# Multi-omics reveals cross-tissue regulatory mechanisms of autism risk loci via gut microbiota-immunity-brain axis

**DOI:** 10.1186/s13568-025-01969-4

**Published:** 2025-10-29

**Authors:** Xingxing Liao, Junzi Long, Xianna Wang, Kaiyue Han, Zhiqing Tang, Jiarou Chen, Yan Zhang, Hao Zhang

**Affiliations:** 1 School of Rehabilitation, Capital Medical University, Beijing, China; 2 Changping Laboratory, Beijing, China; 3 National Autism Rehabilitation Research Center, China Rehabilitation Research Center, Beijing, China; 4 Wenzhou Medical University, Wenzhou, China; 5 Cheeloo College of Medicine, Shandong University, Jinan, China; 6 Beijing Bo’ai Hospital, China Rehabilitation Research Center, School of Rehabilitation, Capital Medical University, Beijing, 100068 China; 7 Division of Brain Sciences, Changping Laboratory, Beijing, 102206 China

**Keywords:** Autism spectrum disorder, GWAS, Multi-omics integration, Gut Microbiota-Immunity-Brain axis

## Abstract

Autism Spectrum Disorder (ASD) involves a multi-system interaction mechanism among genetics, immunity, and gut microbiota, yet its regulatory network remains undefined. This study conducted a meta-analysis on Genome-Wide Association Study data from four independent ASD cohorts to identify potential genetic loci. By integrating Polygenic Priority Score, brain region, and brain cell eQTL enrichment analyses, and combining summary-data-based Mendelian Randomisation (SMR) analyses of brain cis-eQTL and mQTL, bidirectional Mendelian Randomisation analyses of 473 gut microbiota, and SMR analysis of blood eQTL, SNPs such as rs2735307 and rs989134 with significant multi-dimensional associations were identified. These loci exert cross-tissue regulatory effects by participating in gut microbiota regulation, involving immune pathways such as T cell receptor signal activation and neutrophil extracellular trap formation, as well as cis-regulating neurodevelopmental genes (*HMGN1* and *H3C9P*), or synergistically influencing epigenetic methylation modifications to regulate the expression of *BRWD1* and *ABT1*. The cross-scale evidence chain constructed in this study provides a theoretical foundation for precision medicine research in ASD, holding promise to advance the development of innovative therapeutic strategies.

**Supplementary Information:**

The online version contains supplementary material available at 10.1186/s13568-025-01969-4.

## Introduction

Autism spectrum disorder (ASD) is a neurodevelopmental disorder characterised by deficits in social communication, repetitive behaviours, and restricted interests (Association 2013). The aetiology of ASD is profoundly intricate, with genetic factors playing a pivotal role. As early as 1977, Folstein et al. revealed the high heritability of autism through their inaugural study of twins (Folstein and Rutter 1977). Subsequent meta-analyses have estimated heritability values as high as 64% to 91% (Tick et al. 2016). Large-scale whole-genome sequencing studies of patients have now identified numerous highly reliable and recurrent risk loci (Doan et al. 2019; Sanders et al. 2012; Satterstrom et al. 2020; Werling et al. 2018). However, it is important to note that ASD patients also exhibit significant genetic heterogeneity, with genetic mutations detected in approximately 20% of cases, but no single mutation accounts for more than 1% of ASD cases (Jeste and Geschwind 2014). Consequently, contemporary genetic testing remains incapable of accurately predicting or diagnosing ASD.

Recent studies have indicated that the pathophysiological mechanisms of ASD involve a complex network of genetic, environmental, and immune factors, as well as their interactions (Jiang et al. 2022), leading to changes in brain structure and function, which manifest as the core behavioural symptoms of ASD (Zhuang et al. 2024). In recent years, gut microbiota has emerged as a key environmental factor influencing ASD, with non-host genetic material possessing unique vertical transmission characteristics, enabling its transmission to offspring through host reproduction (Moeller et al. 2018). A substantial body of research has demonstrated that gut microbiota exerts bidirectional dynamic regulation on the brain through the gut-brain axis, which encompasses neural, immune, and metabolic pathways (Kraimi et al. 2024; WangYang & Liu 2023). This cross-system interaction may be fundamentally reliant on genetic-level regulation. For instance, neuroactive metabolites produced by gut microbiota (e.g. 5-aminovaleric acid and taurine) have been demonstrated to regulate the alternative splicing of neuronal genes in the brain (Sharon et al. 2019). This, in turn, has been shown to lead to extensive alternative splicing abnormalities in ASD risk genes such as *FMR1*, *Nrxn2*, and *Ank2*, as well as imbalances in GABAergic neuronal excitability (Sharon et al. 2019). These molecular events have been demonstrated to be closely associated with neuronal developmental abnormalities and defects in synaptic plasticity (Sharon et al. 2019). This association has been confirmed through the execution of germ-free mouse colonization experiments, which have shown that gut microbiota can directly induce the emergence of ASD-like behavioural features (Sharon et al. 2019). On the other hand, defects in the ASD high-risk gene *Chd8* can specifically induce abnormal activation of microglia in the brain, while also causing intestinal immune homeostasis imbalance, manifested as disrupted T and B lymphocyte differentiation and significantly reduced immunoglobulin IgA levels, ultimately leading to impaired intestinal barrier structure and function (Ji et al. 2025). These cross-system pathological changes suggest that abnormal gene expression in immune cells of the central nervous system can also remotely regulate the composition and function of the gut microbiota through the gut-brain axis.

Despite mounting evidence indicating that ASD is associated with intricate interactions with other peripheral systems (e.g., the gut microbiota and immune system) and that these interactions play a pivotal role in the onset and progression of the disease (Moradi et al. 2021), conventional genome-wide association studies (GWAS) are frequently constrained to genetic association analyses within a solitary tissue (e.g., brain regions), impeding the capture of the cross-tissue pathogenic characteristics of ASD as a “systemic disease”. A study found that genetic variants associated with ASD are significantly enriched in methylation regulatory sites in brain tissue and peripheral blood. The same study also found that these variants are co-enriched in immune-related pathways (Andrews et al. 2017). However, the present study did not provide explicit evidence of cross-tissue genetic regulation involving peripheral systems such as the gut microbiota. Indeed, genetic variants are likely to act as core drivers, coordinating the dynamic balance of brain neural development, blood-immune responses, and gut microbiota interactions through molecular networks. However, there is currently a paucity of sufficiently systematic research integrating multi-omics data at the genetic level to elucidate how these variants drive ASD pathological processes through cross-tissue regulatory networks.

**Fig. 1:**
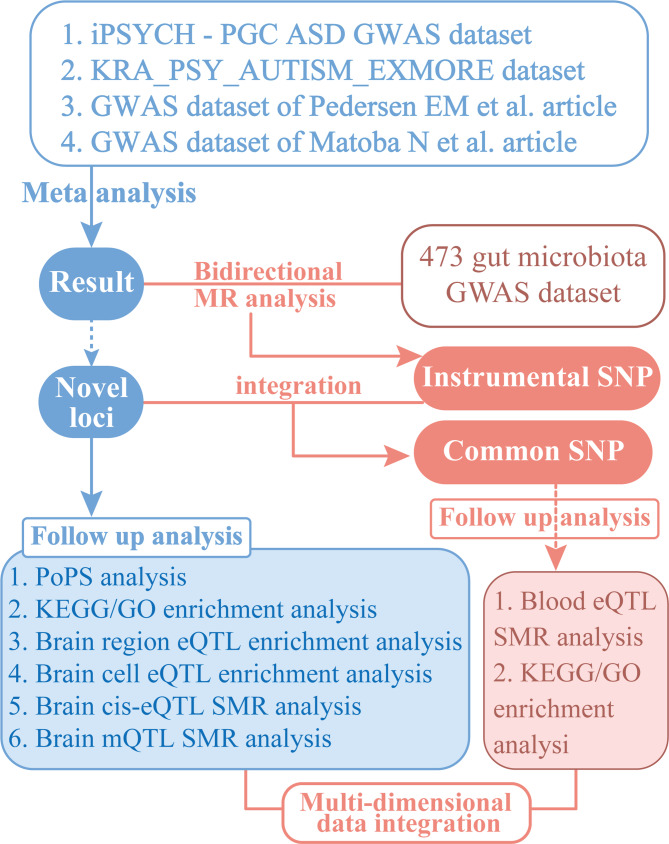
Research flowchart

The objectives of the study are achieved through a multi-stage analysis, the specific process of which is shown in Fig. 1. Firstly, a meta-analysis of multi-source ASD GWAS data was conducted to screen for novel loci significantly associated with ASD. Secondly, we conducted a series of analyses, including Polygenic Priority Score (PoPS) analysis, neuro tissue-specific enrichment analysis of brain region/brain cell expression quantitative trait loci (eQTLs), and Summary-data-based Mendelian Randomization (SMR) analysis based on brain cis-expression quantitative trait loci (cis-eQTL) and methylation quantitative trait loci (mQTL) data. This process involved the screening of single-nucleotide polymorphisms (SNPs) that were significantly associated with multiple-dimensional analyses. Subsequently, we employed Mendelian randomization (MR) analysis to assess the causal association between gut microbiota composition and ASD, and integrated blood eQTL data using the SMR method to screen for variants with immune pathway regulatory effects. The objective of this study is to integrate multi-omics data, employing novel genetic loci in ASD as a point of departure, to elucidate their cross-tissue regulatory mechanisms within the “gut microbiota-immune-brain axis”. This will engender insights for the precise diagnosis and treatment of this disorder.

## Materials and methods

### Data sources

All data involved in this study were publicly accessible and ethically approved in all original publications. Genetic data for ASD were derived from four independent ASD GWAS datasets: ① The European ASD genetics dataset published by the iPSYCH-PGC Consortium (cases: 18,382; controls: 27,969); ② The European autism GWAS dataset from a study by Pedersen EM et al. (Pedersen et al. 2023) (cases: 18,235; controls: 36,741); ③ The KRA_PSY_AUTISM_EXMORE dataset from the Finnish database (R12 version) (cases: 888; controls: 362,304); ④ The European autism GWAS dataset from a study by Matoba N et al. (Matoba et al. 2020) (cases: 22,916; controls: 32,504). Gut microbiota GWAS data were obtained from a study by Qin Y et al. (Qin et al. 2022) (sample size: 5959), which includes genetic information on the abundance of 473 microbial taxonomic groups (from phylum to species).

### Genetic variation integration and meta-analysis

To ensure genomic consistency, CrossMap (v0.6.5) and UCSC chain files (hg19ToHg38.chain) were utilised to convert genomic coordinates from hg19 to hg38 for GWAS datasets released by the iPSYCH-PGC Consortium. Subsequently, PLINK (v1.9) was utilised to align the 1000 Genomes Phase 3 reference panel, to correct allele direction (reverse incorrect chains or exclude mismatched SNPs). The fixed-effects model in METAL (v2023, https://genome.sph.umich.edu/wiki/METAL) was utilised to integrate four ASD datasets, with the SCHEME STDERR and STDERR SE strategies employed to weight the data. The AVERAGEFREQ and MINMAXFREQ options were enabled to exclude SNPs with cross-study eAF differences greater than 0.2. The fixed-effects results were generated using METAL, and Cochran’s Q and I^2^ indices were calculated. Standard errors (SE, $\:SE=\frac{1}{\sqrt{2\times\:P\times\:\left(1-P\right)\times\:\left(weight+{Z}^{2}\right)}}$) and effect sizes (β, $\:\beta\:=SE\times\:Z$) were calculated based on Z-scores, weights, and * P*-values. In instances where substantial heterogeneity is identified (Q test *P* < 0.1 and heterogeneity index I^2^>50%), the R package (metafor) is employed to access the METAL output results, and the DerSimonian-Laird method in the random-effects model is utilised to recalculate the effect size and confidence intervals.

### Novel loci screening

The data obtained from the meta-analysis was merged with the pre-processed data from the four different original sources, and chromosome and position information was extracted. The following steps were taken in the screening of novel loci: ① Exclusion of known loci: Novel loci were defined as SNPs located ≥ 500 kilobases apart from previously reported loci on the same chromosome. Linkage disequilibrium pruning, which involves the retention of independent SNPs with a r^2^ value less than 0.001 within a 10,000-kilobase window, and the selection of variants with a* P*-value less than 5 × 10^− 6^, was performed for each original study dataset. The chromosome and position information of these variants was then extracted for the construction of a known locus database. ② Distance filtering: For each SNP identified in the meta-analysis results, the position of the SNP on the same chromosome must be checked to ensure that it is within ± 500 kilobases of a known locus. SNPs that meet these criteria must then be excluded, and novel loci must finally be obtained.

### Gene enrichment analysis based on pops scores

Gene annotation: Utilising the biomaRt package connected to Ensembl version 109 (GRCh38 genome), genes within 500 kilobases upstream and downstream of each new site were retrieved. The distance between the SNP and the nearest gene was calculated, and all associated genes were recorded.

PoPS analysis: The PoPS method is a novel similarity-based gene prioritisation approach that integrates GWAS summary statistics with gene features (e.g. cell type-specific expression, chromatin interactions, biological pathways) via a supervised learning model to assign a priority score to each protein-coding gene, thereby quantifying its predictive power for the trait (Weeks et al. 2023). In this study, PoPS scores for autism-related genes were extracted from the PoPS dataset. The associated genes for each SNP were matched with their PoPS scores, and the highest PoPS score and gene name corresponding to each SNP were retained. The gene names obtained were then subjected to pathway enrichment analysis using the Gene Ontology (GO) and Kyoto Encyclopedia of Genes and Genomes (KEGG) databases. This process was undertaken to identify significantly enriched pathways.

### Enrichment of brain regions/cell-specific eQTLs

The brain regions’ eQTL data (v10) were obtained from the public data access channel of the GTExPortal website. This data set includes eGenes (genes significantly associated with cis-eQTLs, *P* < 1 × 10^− 12^) and significant SNP-gene pairs. The data about brain cells was obtained from the public data access channel on the Zenodo website (https://zenodo.org/records/7276971). The results of the enrichment analysis, based on PoPS scores, were utilised to assess enrichment in each brain region/cell type. The degree of enrichment was evaluated using the hypergeometric test (phyper function). The null hypothesis posits an absence of association between ASD risk loci and tissue/cell-specific regulatory variants, with* P*-values calculated as follows: $$\:P\left(X\ge\:k\right)=1-{\sum\:}_{i=0}^{k-1}\frac{\left(\begin{array}{c}m\\\:i\end{array}\right)\left(\begin{array}{c}n\\\:r-i\end{array}\right)}{\left(\begin{array}{c}m+n\\\:\gamma\:\end{array}\right)}$$. In this study, m signifies the number of SNPs derived from prior GWAS results, n denotes the number of non-GWAS SNPs distributed across the genome, r represents the number of eQTL SNPs, and k specifies the number of observed overlaps. The Benjamini-Hochberg (BH) method is utilised for the purpose of multiple testing correction, with the objective being the control of the false discovery rate (FDR) across brain regions/cells.

### SMR analysis

SMR analysis constitutes a core method of Mendelian randomization, based on summary data, with the capacity to effectively detect gene expression levels. This method innovatively integrates GWAS and eQTL aggregated data, quantifies the causal effects of genetic variation on gene expression and phenotypes, systematically assesses the potential biological functions of GWAS-associated genes, and prioritises candidate genes (Zhu et al. 2016). Presently, this analysis is chiefly dependent on the SMR software tool that has been developed by the Jian Yang team at the School of Life Sciences, West Lake University (https://yanglab.westlake.edu.cn/software/smr/#Overview). This tool provides efficient and reliable technical support for elucidating the genetic mechanisms of complex diseases through rigorous statistical models and algorithms. It has been widely applied in the field of biomedical research. This study employed two versions of brain cis-eQTL data. One version was the BrainMeta v1 eQTL data in SMR binary (BESD) format, which was downloaded from the SMR official website (Qi et al. 2022). The other version was the BrainMeta v2 cis-eQTL summary data (Qi et al. 2018). The mQTL data were selected from the Brain-mMeta mQTL summary data, which was also downloaded from the same website (Qi et al. 2018).

### MR analysis

In the context of the gut microbiota GWAS data, genetic variants that demonstrated a significant association with particular bacteria were identified through a rigorous screening process, which was based on the following criteria. Specifically, variants with a significance threshold of *P* < 1 × 10^− 5^ were first identified. To ensure independence, an linkage disequilibrium (LD) analysis was performed with clump_r^2^ = 0.1 and clump_kb = 500 to prune variants, with strong instrumental variables retained if F > 10 to reduce the impact of weak instrumental bias. Consequently, each gut microbiota exposure data point is then matched with the ASD meta-analysis results. Following the harmonisation of SNP, allele, and effect size information using the harmonise_data function in the TwoSampleMR package, a two-sample MR analysis is conducted. The inverse variance weighting (IVW) method and the MR Egger regression analysis are utilised to evaluate the causal association between specific bacterial genera and ASD. The odds ratio (OR) and its 95% confidence interval were calculated, and heterogeneity was assessed using Cochran’s Q test, while multi-effect was determined using the MR Egger intercept test.

Reverse MR analysis: Utilising the findings of a meta-analysis of ASD as exposure data, the present study investigated the potential causal effects of ASD on the gut microbiota. The following criteria are to be applied for genetic variant screening: variants with *P* < 5 × 10^− 6^ are to be retained, only strong instrumental variables with F-statistics > 10 are to be included, independence among SNPs is to be ensured by performing LD pruning (clump_r^2^ < 0.001, clump_kb = 10,000), and SNPs already used in the forward MR analysis are to be excluded to avoid overlap bias. For the screened ASD exposure data, harmonisation was performed with the gut microbiota outcome data, with the subsequent methods mirroring those employed in the forward MR analysis.

## Results

### Multidimensional functional annotation and cross-tissue expression analysis of novel genetic loci associated with ASD

#### Genome-wide meta-analysis to screen for novel loci

A meta-analysis of GWAS data from four distinct sources about ASD was conducted, leading to the identification of 12,619,808 SNPs associated with ASD. Following rigorous quality control procedures and a thorough comparison with previously reported raw data, 47 novel significant variant loci were identified. It is noteworthy that these loci are situated more than 500 kilobases away from any previously documented indexed variants. Subsequently, the new significant loci were annotated with gene information, resulting in the identification of the nearest genes to each novel loci and all genes within a 500 kilobase range of each novel loci.

#### Novel loci pops analysis

The PoPS analysis was utilised to identify the highest PoPS score and gene name corresponding to each novel loci (see Table 1 for details). The PoPS score is a multidimensional metric of the strength of the association between a gene and a phenotype. A higher score indicates that the gene plays a more critical causal role in the genetic mechanisms of specific complex traits or diseases. As demonstrated in the analysis results, genes such as *NEDD4L* (PoPS score: 0.99393871, same below), *NTM* (0.87557398), *ATXN1* (0.81590442), *PRKACB* (0.80825122), and *NKAIN3* (0.80155922) have all been shown to have PoPS scores exceeding 0.8. This suggests that they are associated with ASD and may increase the risk of the disease. It is noteworthy that the genes *POU4F1* (− 0.12781026) and *LRP1B* (− 0.05192686) have been identified as having negative scores, which may suggest that they possess a protective effect or that their functional activities are in opposition to the disease mechanism.

**Table 1:** Results of pops analysis of novel loci

SNP	CHR	BP	EA	OA	beta	se	*P*	Nearest_Gene	Distance	ALL_genes	PoPS_gene	PoPS_score
rs10932541	chr2	214,526,141	A	G	− 0.0206	0.0032	1.484e-10	*VWC2L*	0	*VWC2L*	*VWC2L*	0.252953
rs11018825	chr11	88,211,504	A	G	− 0.0286	0.0048	3.518e-09	*MIR3166*	34,911	*CTSC*	*CTSC*	0.490402
rs11742387	chr5	78,518,702	A	G	0.0167	0.003	4.233e-08	*LHFPL2*	0	*LHFPL2*	*LHFPL2*	0.222906
rs12407596	chr1	104,264,068	G	A	− 0.0569	0.0092	5.729e-10	*FTLP17*	110,238	*FTLP17*		
rs12468984	chr2	15,270,984	A	G	− 0.0244	0.0044	2.41e-08	*NBAS*	0	*NBAS*	*NBAS*	0.025553
rs13017968	chr2	40,347,532	T	G	− 0.016	0.0029	4.824e-08	*SLC8A1*	0	*SLC8A1*	*SLC8A1*	0.476399
rs1363105	chr5	104,582,089	C	T	− 0.0581	0.0094	5.581e-10	*NIHCOLE*	175,968	*NIHCOLE*		
rs1431594	chr8	63,422,191	T	C	− 0.018	0.0029	6.112e-10	*IFITM8P*	12,734	*NKAIN3*	*NKAIN3*	0.801559
rs1440786	chr8	52,474,003	T	C	− 0.0206	0.003	1.169e-11	*ST18*	13,044	*RB1CC1*	*RB1CC1*	0.349369
rs150681721	chr7	105,410,099	G	A	− 0.1089	0.0187	5.833e-09	*SRPK2*	10,791	*SRPK2*	*SRPK2*	0.467305
rs152272	chr5	144,779,767	T	G	-0.0168	0.003	1.453e-08	*NAMPTP2*	222,086	*KCTD16*	*KCTD16*	0.293201
rs17439102	chr7	78,588,509	T	C	− 0.0186	0.0029	8.745e-11	*MAGI2*	0	*MAGI2*	*MAGI2*	0.394352
rs17764737	chr15	42,393,436	T	C	− 0.041	0.0073	2.064e-08	*CAPN3*	0	*VPS39*	*VPS39*	0.235908
rs184028657	chr7	4,235,325	A	G	0.0755	0.0138	4.66e-08	*SDK1*	0	*SDK1*	*SDK1*	0.597626
rs199958221	chr2	158,497,317	T	A	0.0613	0.0101	1.111e-09	*PKP4*	0	*UPP2*	*UPP2*	0.188156
rs201608631	chr4	108,504,414	C	T	0.0576	0.0103	2.138e-08	*RPL34-DT*	33,776	*COL25A1*	*COL25A1*	0.265091
rs201867608	chrchr17	81,283,632	G	T	0.1015	0.0177	1.022e-08	*SLC38A10*	0	*ACTG1*	*ACTG1*	0.509806
rs2070939	chr16	72,074,575	G	A	0.053	0.0093	1.33e-08	*TXNL4B*	0	*AP1G1*	*AP1G1*	0.382902
rs2635482	chr8	81,815,280	T	C	− 0.0175	0.003	6.47e-09	*SNX16*	0	*PMP2*	*PMP2*	0.304464
rs2681527	chr5	114,643,490	C	T	0.0641	0.0112	1.023e-08	*LINC01957*	63,520	*KCNN2*	*KCNN2*	0.523066
rs2735307	chr21	39,341,432	T	C	0.0157	0.0027	9.286e-09	*HMGN1*	883	*BRWD1*	*BRWD1*	0.186094
rs2929306	chr8	9,227,399	C	T	0.0635	0.0093	8.701e-12	*PPP1R3B-DT*	0	*MFHAS1*	*MFHAS1*	0.194376
rs3127074	chr10	113,704,373	A	G	0.0171	0.0028	5.554e-10	*CASP7*	0	*CASP7*	*CASP7*	0.378614
rs34029608	chr5	16,848,564	T	C	0.025	0.0041	1.516e-09	*MYO10*	0	*MYO10*	*MYO10*	0.289748
rs4332640	chr13	78,284,001	C	T	− 0.0534	0.0096	2.829e-08	*OBI1-AS1*	0	*POU4F1*	*POU4F1*	-0.12781
rs4673652	chr2	200,038,855	T	C	− 0.038	0.0069	3.502e-08	*MAIP1*	30,315	*TYW5*	*TYW5*	0.232567
rs524890	chr11	131,913,555	A	T	0.0245	0.0039	4.116e-10	*NTM*	0	*NTM*	*NTM*	0.875574
rs56306882	chr3	70,204,542	A	G	0.0185	0.003	1.11e-09	*SAMMSON*	0	*MITF*	*MITF*	0.220207
rs58488829	chr10	81,427,046	T	C	0.0215	0.0037	1.052e-08	*RPA2P2*	289,711	*NRG3*	*NRG3*	0.609343
rs63615960	chr18	58,214,169	C	T	− 0.0627	0.01	3.73e-10	*NEDD4L*	0	*NEDD4L*	*NEDD4L*	0.993939
rs6543221	chr2	103,587,219	C	T	− 0.0503	0.0091	3.421e-08	*CAPZBP1*	36,411	*CRLF3P1*		
rs7044486	chr9	73,389,649	G	A	− 0.057	0.0104	3.727e-08	*DPP3P2*	84,729	*ALDH1A1*	*ALDH1A1*	0.493283
rs72833606	chr6	17,259,402	T	C	0.0287	0.0049	4.669e-09	*RBM24*	21,959	*ATXN1*	*ATXN1*	0.815904
rs72855404	chrchr2	141,569,426	T	A	0.0855	0.0156	4.39e-08	*LRP1B*	0	*LRP1B*	*LRP1B*	-0.05193
rs73073015	chr3	49,465,733	A	G	0.0303	0.0048	2.679e-10	*RNA5SP130*	1382	*RHOA*	*RHOA*	0.616839
rs73909576	chr20	43,164,614	A	G	− 0.0382	0.0069	2.838e-08	*PTPRT*	0	*SRSF6*	*SRSF6*	0.233022
rs7429220	chr3	62,497,213	T	C	− 0.0206	0.0032	6.672e-11	*CADPS*	0	*FEZF2*	*FEZF2*	0.246063
rs75952149	chr14	42,950,959	G	A	− 0.0622	0.0113	3.505e-08	*YWHAQP1*	142,847	*YWHAQP1*		
rs7629432	chr3	158,177,496	C	T	0.0522	0.0093	1.708e-08	*RSRC1*	0	*MLF1*	*MLF1*	0.325999
rs76504400	chr2	1,719,132	A	G	0.0208	0.0037	1.564e-08	*PXDN*	0	*TPO*	*TPO*	0.254077
rs7719580	chr5	51,185,612	T	C	0.0204	0.0034	1.841e-09	*RNU6-1296P*	17,965	*ISL1*	*ISL1*	0.43911
rs77258906	chr1	83,660,321	T	C	− 0.0191	0.0034	2.253e-08	*LINC01725*	0	*PRKACB*	*PRKACB*	0.808251
rs79020737	chr6	23,777,565	C	A	− 0.0582	0.0102	1.207e-08	*SPTLC1P2*	79,133	*NRSN1*	*NRSN1*	0.056713
rs9320913	chr6	98,136,857	C	A	0.063	0.0093	1.064e-11	*EIF4EBP2P3*	42,642	*MIR2113*		
rs9848946	chr3	182,581,060	G	A	− 0.0552	0.01	3.234e-08	*RPL7L1P8*	29,614	*ATP11B*	*ATP11B*	0.136719
rs989134	chr6	26,335,996	C	A	0.0157	0.0028	2.765e-08	*H3C9P*	13,704	*ABT1*	*ABT1*	0.40631
rs9913471	chr17	67,878,379	T	C	0.0186	0.0033	2.113e-08	*BPTF*	0	*BPTF*	*BPTF*	0.312634

SNP: Single Nucleotide Polymorphism; CHR: Chromosome; BP: Base Pair position; EA: Effect Allele; OA: Other Allele; beta: Effect size; se: Standard Error; P: probability value; ALL_genes: All genes corresponding to each novel locis

**Fig. 2:**
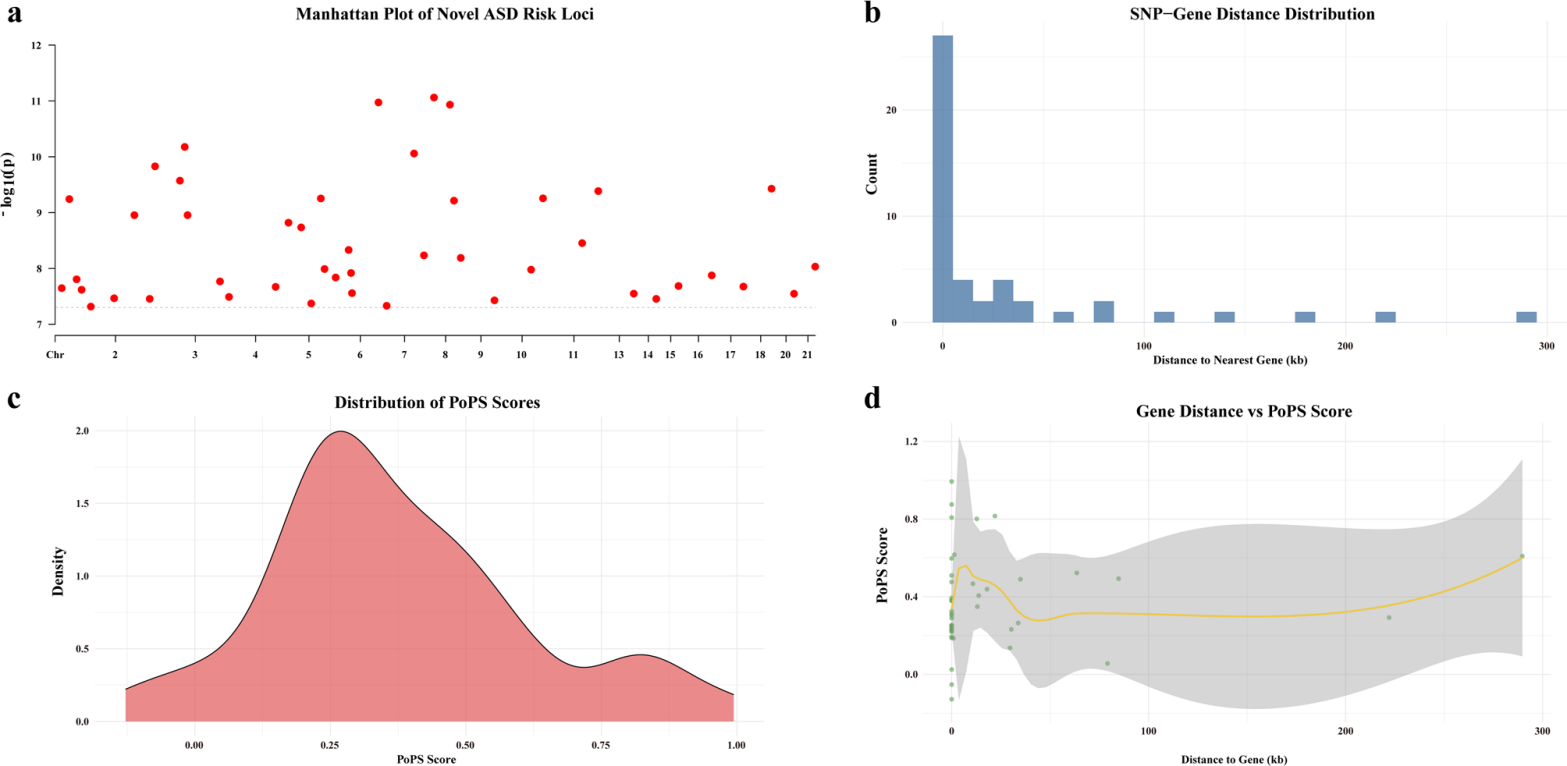
Visualisation of PoPS analysis results for novel ASD risk loci.** a** Manhattan plot of novel ASD risk loci, with the x-axis representing chromosome number and the y-axis representing the − log_10_ transformed value of the* P*; Red dots represent gene loci, with higher vertical coordinates indicating a stronger association with ASD risk. ** b** SNP–Gene Distance Distribution Histogram. The x-axis represents the physical distance from the SNP to the nearest gene, and the y-axis represents the number of SNPs in the corresponding distance interval.** c** PoPS score density distribution plot. The x-axis represents the PoPS score (quantitatively reflecting the strength of association between the gene and ASD genetic mechanisms), and the y-axis represents the probability density of the score. ** d** A scatter plot showing the association between gene distance and PoPS score, used to analyse the relationship between the physical distance from an SNP to a gene and its functional importance (PoPS score). The x-axis represents the distance from the SNP to the nearest gene; the y-axis represents the PoPS score, with higher scores indicating greater functional importance; each point represents an SNP, with coordinates corresponding to its ‘gene distance’ and ‘PoPS score’; The grey area shows the distribution density of PoPS scores at different distances, with wider areas indicating more SNPs in that distance range, and darker colours indicating more data points at that location; the yellow curve is the LOESS fitted curve, smoothly showing the overall association trend between gene distance and PoPS score

Furthermore, the ASD new risk locus Manhattan plot (Fig. 2a) demonstrates that the majority of points are situated below the threshold line, thereby indicating that a limited number of loci within the genome are significantly associated with ASD risk. This finding is consistent with the genetic characteristics of complex diseases characterised by multiple genes and weak effects. The histogram of the distance distribution between SNPs and nearby genes (Fig. 2b) further suggests that the majority of SNPs are located near genes, a circumstance that has the potential to directly affect gene expression (e.g., promoters, enhancer regions). As demonstrated in Fig. 2c, the PoPS score density distribution plot indicates that the majority of data are concentrated in the low-to-medium score range (approximately 0.1–0.6). This observation indicates that a substantial proportion of genes or regions exhibit analogous PoPS scores and consistent selection signal strengths. The association distribution plot of gene distance and PoPS scores (Fig. 2d) reveals that nearby SNPs demonstrate greater functional diversity, while distant SNPs exhibit diminished overall functionality, though exceptions to this pattern do exist. In summary, the study results suggest that we should prioritise high-score, gene-proximal SNPs for further investigation into their mechanisms of action in disease onset and evolutionary processes.

#### Functional enrichment analysis of novel loci

In this study, we further utilised the KEGG and GO databases to perform functional enrichment analysis on the novel loci that obtained PoPS scores. The GO functional enrichment results principally indicate neurodevelopment-related biological processes, including the regulation of neuronal apoptosis, neuronal apoptotic process, cell fate commitment, regulation of neuronal differentiation, and neuronal migration, amongst others. A comprehensive assessment employing − log_10_(*P*.adj) and Gene Count revealed statistically significant enrichment of genes (*P*.adj < 0.01) in the processes of “regulation of neuron differentiation” and “neuron migration”. The KEGG pathway enrichment results indicate that the novel loci were significantly enriched in pathways such as tight junctions, Salmonella infection, pathogenic Escherichia coli infection, proteoglycans in cancer, platelet activation, and the oxytocin signalling pathway. Among these, the tight junction pathway exhibited the highest gene count and statistical significance (*P*.adj < 0.001), suggesting it may play a central role in the cross-tissue pathology of ASD. These findings suggest that these specific processes may be pivotal pathways through which genetic risk variants exert their influence on neurodevelopment in individuals with ASD.

#### Novel loci enriched in brain regions analysis

**Fig. 3:**
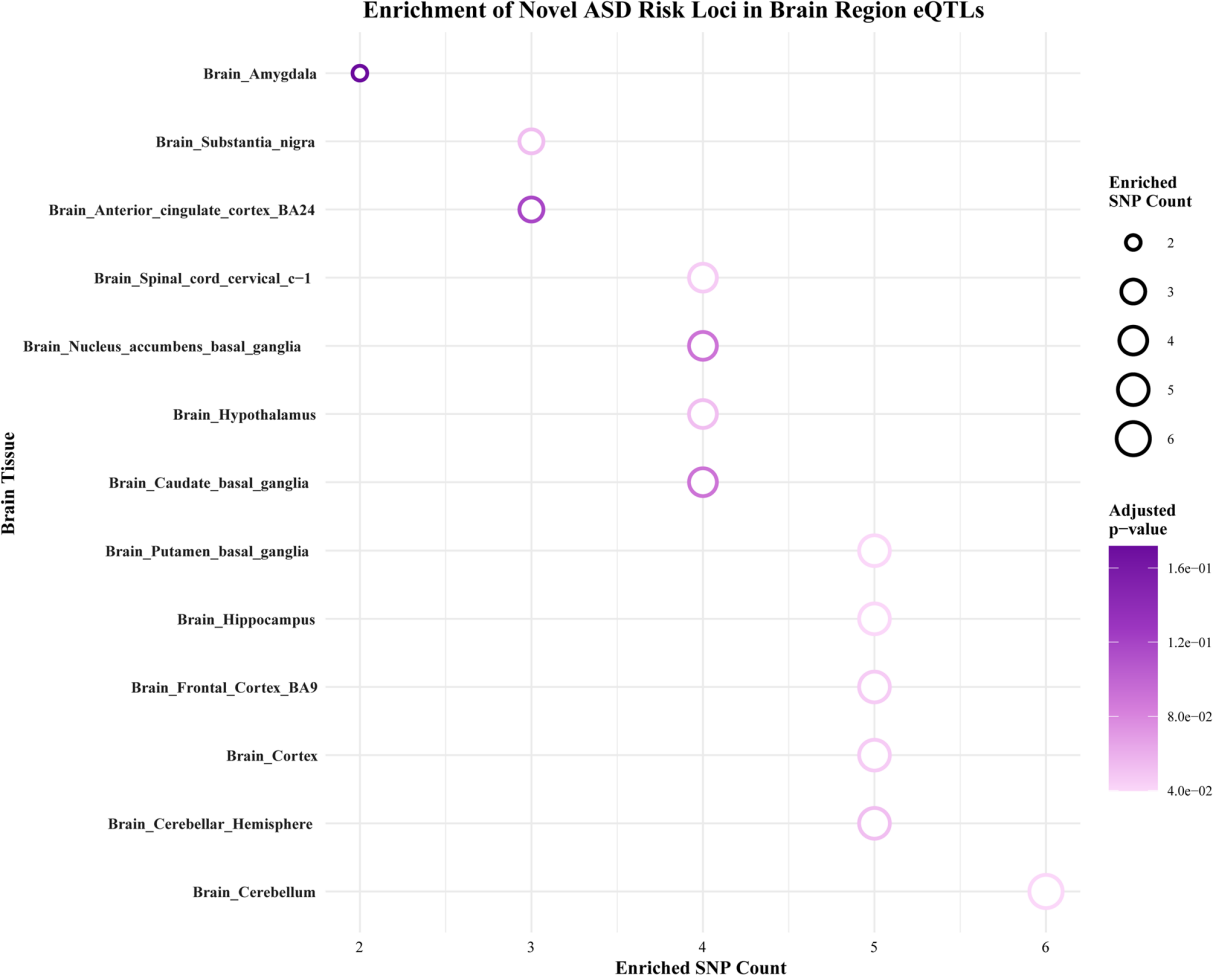
Enrichment level plot of novel loci in brain region eQTLs data. The x-axis represents the number of eQTL-SNP pairs overlapping with novel loci across different brain regions, with a higher number indicating a greater density of ASD-risk SNPs enriched in that brain region; The y-axis represents different brain regions, covering various brain tissues from the cortex to deep nuclei; bubble size is positively correlated with the number of enriched SNPs, with larger bubbles indicating a higher concentration of SNPs in that brain region; The bubble colour represents the adjusted* P*-value, with darker colours indicating stronger enrichment significance

In addition to conventional KEGG and GO functional enrichment analysis, we also performed specific enrichment analysis using novel loci and brain regions’ eQTL data. The eQTL data primarily encompassed thirteen brain regions, including the amygdala, the anterior cingulate cortex (BA24), the caudate basal ganglia, the cerebellar hemisphere, the cerebellum, the cortex, the frontal cortex (BA9), the hippocampus, the hypothalamus, the nucleus accumbens basal ganglia, the putamen basal ganglia, the spinal cord cervical c-1, and the substantia nigra. The significance of the overlap between novel loci and GTEx brain regions eQTL data was calculated using a hypergeometric test. This was done to assess whether specific brain regions are enriched with disease-associated regulatory variants. The results of the study are presented in Fig. 3. The results indicate that new loci for risk of ASD show a trend of enrichment in eQTLs across multiple brain regions, suggesting that abnormal gene expression in these brain regions may be involved in the pathogenesis of ASD. Specifically, the cerebellar hemispheres (5 SNPs), cerebellum (6 SNPs), nucleus accumbens basal ganglia (5 SNPs), and hypothalamus (6 SNPs) were found to be more significant, particularly with adjusted* P*-value as low as 4.0e− 02 in the cerebral cortex. This finding indicates more reliable enrichment signals and suggests that gene expression regulation in the cerebral cortex, as the primary brain region, may be closely related to the pathogenesis of ASD.

#### Novel loci enriched in brain cell analysis

In addition, the objective was to investigate the enrichment of novel loci in specific brain cells. To achieve this objective, we have utilised innovative loci to conduct an enrichment analysis on eight specific brain cells (brain parenchymal cells and glial cells). These cell types comprise key cell types in the central nervous system involved in information transmission and supportive functions, specifically: The following cell types are to be considered: astrocytes, endothelial cells, excitatory neurons, inhibitory neurons, microglia, oligodendrocytes, oligodendrocyte progenitor cells, and pericytes. The hypergeometric test was employed to analyse the significance of the overlap between novel loci and brain cell eQTL data. In summary, novel genetic loci exhibited enrichment factors exceeding 1.0 across all target brain cell types (including astrocytes, endothelial cells, and six other categories), with all results passing the FDR < 0.05 significance threshold. This finding indicates that the novel loci identified in this study exhibit significant enrichment in those above eight specific brain cell types, with this enrichment demonstrating statistical significance. This further supports the hypothesis that these novel loci may be associated with the functional regulation of particular brain cells.

#### Novel loci SMR analysis

**Fig. 4:**
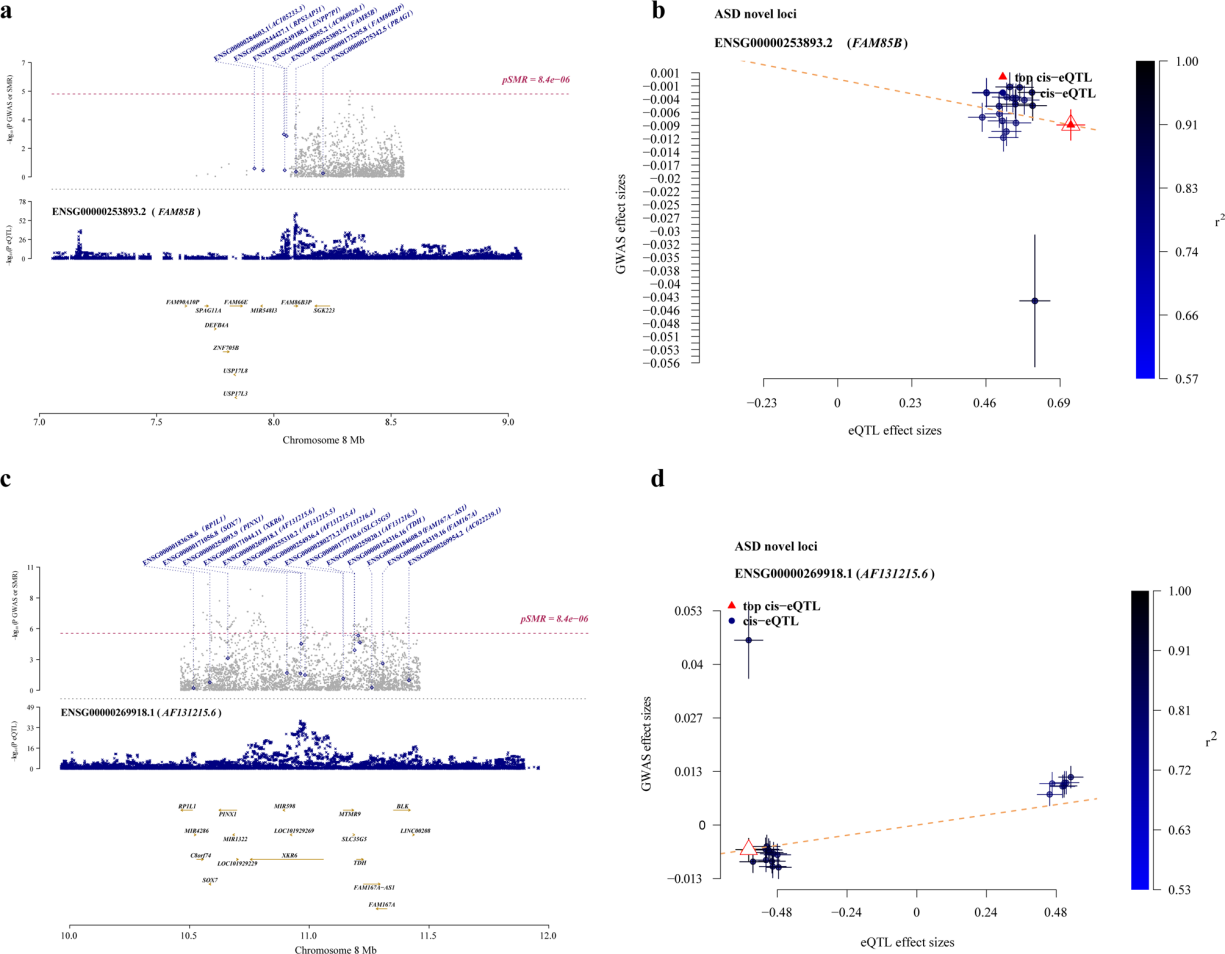
Visualisation of novel SMR locus results. SMR locus plot x-axis: The term “chromosomal physical location” is used to denote the genomic interval in which the target gene is located; The left vertical axis (Y_1_ axis) is defined as follows: log_10_(P GWAS or SMR) is a metric employed to ascertain the significance level of GWAS or SMR analysis, a greater value indicates a smaller * P*-value and stronger significance; The right vertical axis (Y_2_ axis) is defined as follows: The − log_10_(P eQTL) metric is employed to denote the statistical significance of eQTL analysis, which is utilised to evaluate the strength of the correlation between gene expression and SNPs; The distribution of other gene names along the horizontal axis is indicative of the distribution characteristics of gene clusters, aiding in the identification of functionally significant regions of genetic association. Effect sizes plot: Show the association between GWAS effect sizes and eQTL effect sizes to illustrate whether the influence of SNPs on phenotypes is mediated by gene expression. Each point represents a SNP-probe pair, where: x-axis: eQTL effect size (the degree of influence of SNPs on gene expression); y-axis: GWAS effect size (the degree of influence of SNPs on phenotypes); The dashed line aids in judging the magnitude of the effect size; the further the deviation, the stronger the effect; Points marked with “top cis-eQTL” represent the most significant eQTL in that region and are priority candidate sites for validation

In the SMR analysis, we used novel loci and brain eQTL data to examine whether genetic variation affects GWAS results by influencing intermediate phenotypes, thereby validating the causal pathway of “SNP → intermediate phenotype → disease.” The results indicate that all original* P*-value (p_SMR) from the SMR analyses were less than 0.05. To mitigate the risk of false positives arising from multiple testing, further adjustment was performed using the BH method for FDR correction. Following this adjustment, all corrected * P*-value (FDR_BH) remained below 0.05, indicating that SNPs within novel_loci potentially mediate interactions with brain gene expression. Concerning the assessment of horizontal pleiotropy, the restricted number of SNPs within the novel_loci region hinders the computability of p_HEIDI values. However, SNPs in this region have undergone rigorous LD pruning, with the result that only independent SNPs with minimal linkage density have been retained. This maximises the exclusion of signal confusion caused by strong linkage. Furthermore, the maximum and minimum values of beta are ENSG00000269918.1 (b_SMR = 0.18) and ENSG00000253893.2 (b_SMR=-0.17), respectively. The utilisation of a locus plot is employed to visually depict the distribution characteristics of genetic association signals within the genomic region. Among these, the locus plot for ENSG00000253893.2 (*FAM85B*) (Fig. 4a) demonstrates its location in the 7.0–9.0 Mb region of chromosome 8, with neighbouring genes including *FAM86B3P* and *FAM90A10P*, and a pSMR value of 8.4e-06, reaching a significant level. ENSG00000269918.1 (Fig. 4c) is located on chromosome 8 between 10.0 and 12.0 Mb, with neighbouring genes including *BLK* and *PINX1*, and also meets the pSMR significance threshold. The SMR Effect Plot provides a visual representation of the association characteristics. In the Effect Plot for ENSG00000269918.1 (Fig. 4b), the majority of data points are concentrated in the positive correlation region, with some loci exhibiting GWAS effect sizes as high as 0.91, indicating a high degree of reliability for this association. It may be hypothesised that the increased expression of this gene could be a contributing factor to the onset of ASD through regulatory pathways. In the Effect Plot for ENSG00000253893.2 (Fig. 4d), the majority of data points are concentrated in the negative correlation region, and the GWAS effect size at specific loci reaches 0.91, suggesting that this gene may act as a protective gene for ASD, with its increased expression potentially reducing the risk of disease through regulatory pathways. A range of additional results have been presented as locus plots and effect plots, which are available for consultation in “Supplementary Materials–4 Novel loci SMR results”.

#### Joint analysis of results across different dimensions

Further analysis revealed that in the SMR results obtained from V1 version cis-eQTL and mQTL data, the top SNP (i.e., the SNP identifier most strongly associated with gene expression) had two intersections, namely rs2735307 and rs989134; in the SMR results obtained from V2 version cis-eQTL and mQTL data, there was also overlap with rs2735307. When these results were compared with PoPS scores, brain region, and brain cell enrichment analysis results, it was found that rs2735307 showed significant enrichment in twelve brain regions (excluding Brain_Cerebellum) and eight brain cell types, with its nearest neighbour gene being *HMGN1* and the PoPS gene selected by PoPS scoring being *BRWD1*. The rs989134 SNP was found to be significantly enriched in three brain regions (Brain_Cerebellum, Brain_Cortex, and Brain_Frontal_Cortex_BA9) and the aforementioned eight brain cell types. The nearest neighbour gene was determined to be *H3C9P*, and the corresponding PoPS gene was identified as *ABT1*.

The above results suggest that rs2735307 may regulate the expression of *BRWD1* (which plays a key role in neuronal differentiation, migration, and synaptic function maintenance (Fulton et al. 2022) by cis-regulating its neighbouring gene *HMGN1* (which is involved in regulating chromatin structure and gene expression (Yang et al. 2023) or by mediating epigenetic (methylation) effects on the promoter/genome region, ultimately affecting the chromatin dynamics of brain parenchymal cells and glial cells and neuronal functional networks. It is also postulated that rs989134 may interfere with the transcriptional program and the structural stability of chromatin in neurons in brain regions, such as the cerebellum and cerebral cortex, by cis-regulating the epigenetic state of the nearby pseudogene *H3C9P* (histone-related) or by regulating the expression of *ABT1* (involved in basal transcription initiation (Brower et al. 2010). It is evident that both types of variants contribute concurrently to the pathophysiological processes of ASD through the chromatin remodelling-gene expression regulation-neuronal cell function axis, thus providing a cross-scale evidence chain of “genetic variation-epigenetic regulation-cell function” for elucidating the neurogenetic mechanisms of ASD. This finding provides a significant theoretical foundation for subsequent targeted interventions on related pathways and the identification of novel intervention targets for ASD.

### MR analysis of ASD and 473 gut microbiota

#### Forward MR analysis

**Fig. 5:**
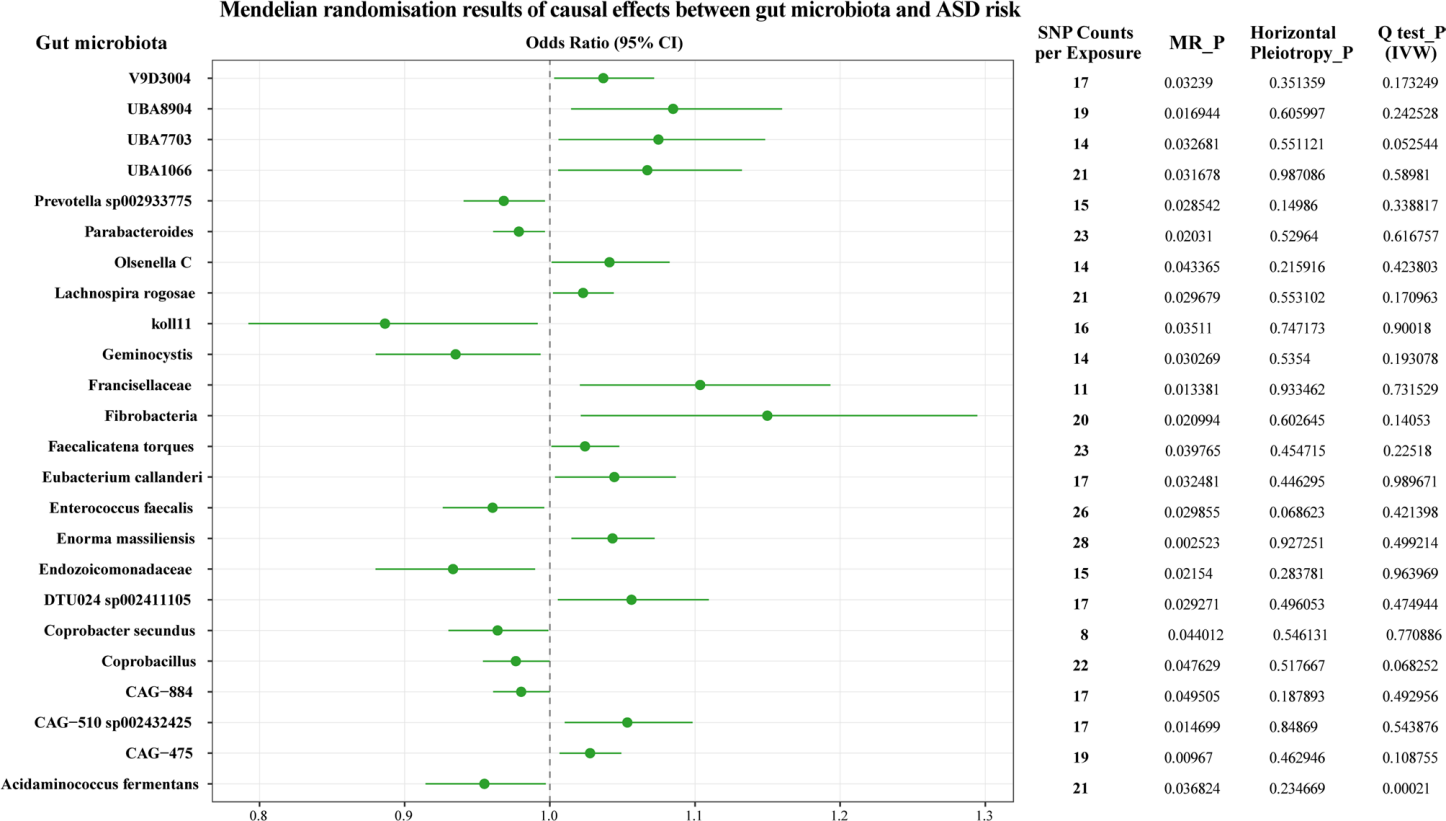
The results plot of the Forward MR analysis. The central part of the figure showcases metrics for measuring the strength of the association between gut microbiota and ASD risk: the x-axis represents the Odds Ratio, with the 95% Confidence Interval (95% CI): the length of the horizontal line indicates the range of the confidence interval, and the y-axis lists the specific gut microbiota exposure factors; “SNP Counts per Exposure” denotes the number of instrumental variables for each exposure; “MR_P” represents the* P*-value results from the MR analysis, while “Q–stat P” indicates the* P*-value for the heterogeneity test

The present study employed a forward MR analysis to investigate the causal association between gut microbiota and ASD. The study utilised 473 gut microbiota species as exposure factors, identified instrumental variables based on strict selection criteria, and then conducted a two-sample MR analysis with the ASD pooled dataset obtained through meta-analysis. Five conventional MR methods were employed to assess the statistical significance of causal effects. As demonstrated in Fig. 5, Following the implementation of an IVW analysis and a BH correction to control for multiple comparison errors, 24 gut microbiota species were identified as having significant causal associations with ASD (*P* < 0.05). In order to validate the reliability of these findings and assess the robustness of causal associations, concurrent validation was performed using MR-Egger regression, leave-one-out validation, and MR-PRESSO analysis. MR-Egger regression intercept* P*-value were consistently > 0.05, and all associations passed leave-one-out validation, suggesting no apparent horizontal pleiotropy. However, the results of Q-tests indicated heterogeneity in some microbial analyses, necessitating a cautious interpretation of causal inferences. The MR-PRESSO global test revealed a sole species (*Acidaminococcus fermentans*) with a *P* < 0.05. Further validation showed its MR-Egger intercept test *P* > 0.05 and passed the leave-one-out validation, indicating that the causal association between this microorganism and ASD remains unaffected by horizontal pleiotropy. Among them, the OR for *Fibrobacteria* was as high as 1.15, suggesting that this microorganism may promote the occurrence of ASD; while the OR value for the *koll11* strain was 0.89, indicating that it may have a potential protective effect against ASD.

**Fig. 6:**
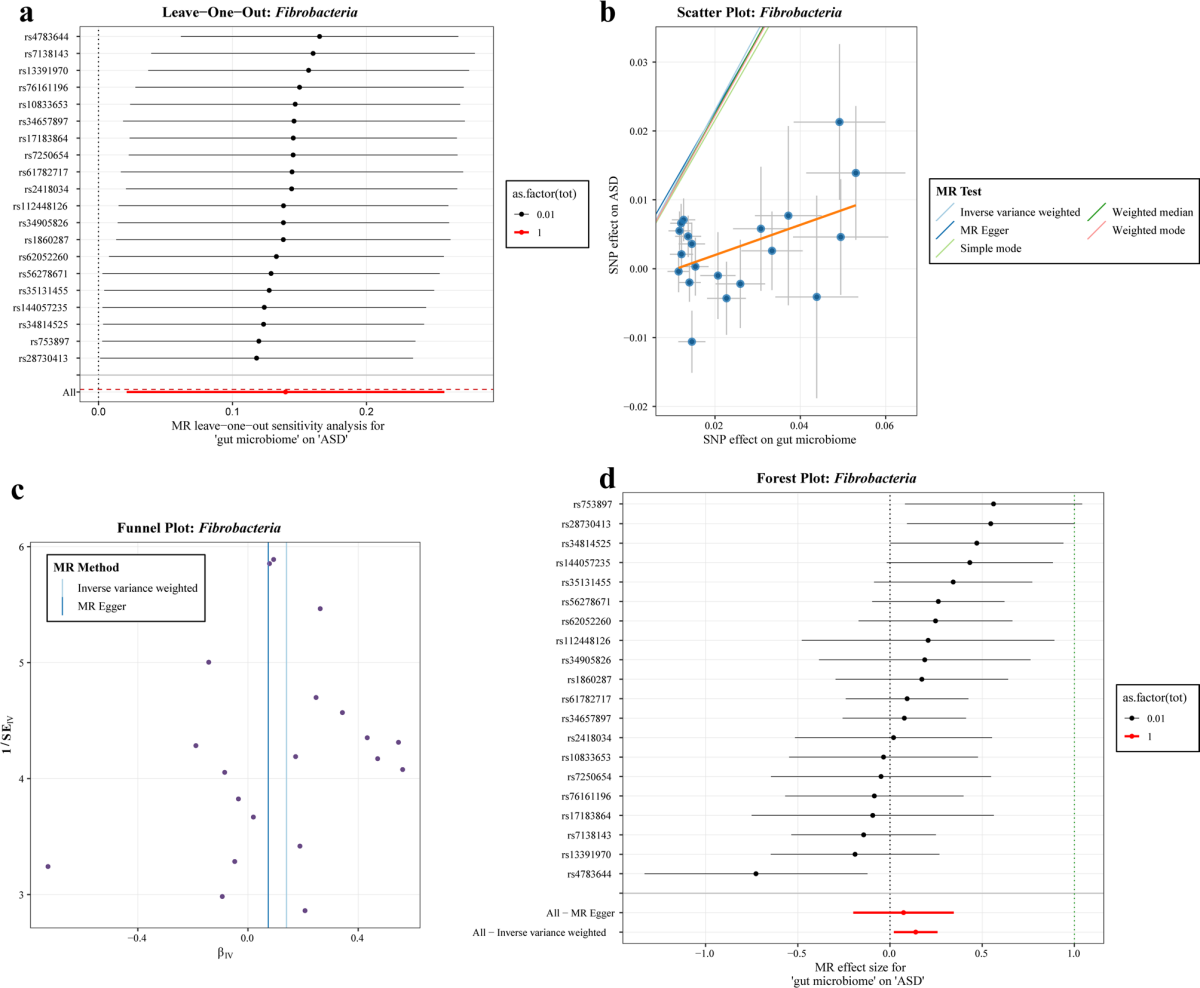
Visualisation of the potential causal association between gut microbiota (*Fibrobacteria*) and ASD.** a** Sensitivity analysis plot showing the trend in Mendelian randomisation effects after removing individual SNPs based on the *Fibrobacteria* abundance instrumental variable set. y-axis: names of SNPs removed one by one; x-axis: effect estimates of exposure → outcome after removing the SNP; red dashed line: MR effect of the entire sample (without removing any SNPs); black dots (as.factor(0.01)), red squares (as.factor(1)): effect points marked with different plotting parameters.** b** Scatter plot showing the distribution of SNP effects on *Fibrobacteria* abundance and ASD, and the causal effect fitting trends of five MR methods: different colours represent different methods.** c** Funnel plot comparing inverse variance-weighted MR methods, showing the distribution of effect estimates (β₍_VW_₎, x-axis) and effect precision (1/SE, y-axis; where 1/SE indicates higher precision of effect estimates), to illustrate the distribution characteristics of instrumental variable effects.** d** A forest plot integrates the ‘individual SNP independent effects’ and ‘aggregated effects of multiple SNPs’ to quantitatively present causal associations. y-axis: names of SNPs included in the analysis one by one; x-axis: MR effect values; black scatter points + horizontal line segments: effect estimates and their 95% confidence intervals when a single SNP is used as an instrumental variable; bottom red horizontal line (All–Inverse variance weighted, All–MR Egger): combined effects of multiple instrumental variables after integrating all SNPs

In order to enhance the reliability of the results, the study utilised scatter plots and forest plots to visually demonstrate the direction and strength of the association between SNP effect values and ASD. Furthermore, funnel plots were employed to assess potential publication bias, and sensitivity analysis was performed using the leave-one-out method. As demonstrated in Fig. 6, the visualisation results for *Fibrobacteria* are presented. Specifically, Fig. 6a indicates that the majority of SNP data points are closely aligned with the bottom red reference line (representing the effect across the entire sample), indicating that the removal of any single SNP does not result in significant fluctuations in the overall association effect estimates. This finding indicates a low reliance on individual instrumental variables and demonstrates a high degree of robustness. As Fig. 6b illustrates, the impact of SNPs on the gut microbiota is characterised by a regular distribution of *Fibrobacteria*, and the fitting lines of various MR models, including IVW and weighted median, demonstrate consistent trends. This suggests that the causal effect estimates from ‘microbiota → ASD’ are stable under multiple statistical strategies. Figure 6c: The point distributions corresponding to the IVW and MR Egger methods are relatively symmetrical and do not exhibit obvious ‘funnel asymmetry’ characteristics, suggesting no significant publication bias or small sample effect bias, and the results are at low risk of interference from systematic bias; Fig. 6d: The confidence intervals for most individual SNPs and the combined effects of the IVW and MR Egger methods are far from zero, quantitatively presenting the magnitude and precision of the effect of ‘*Fibrobacteria* influencing ASD risk’, and further validating the robustness of the association from the ‘single instrumental variable-multiple instrumental variable combination’ dimension. Other significant visualisation results of gut microbiota are detailed in “Supplementary Materials−5 Forward MR analysis results”.

#### Inverse MR analysis

After the conclusion of forward MR analysis, this study undertook to reverse MR to ascertain the directionality of causality. Using ASD data from a meta-analysis as the exposure factor, we rigorously screened and excluded loci that had already been used as instrumental variables in the forward MR analysis, ultimately identifying 53 SNPs for two-sample reverse MR analysis with 473 gut microbiota species. To ensure comparability of results, the reverse MR analysis employed the same methodological framework as the forward analysis, including five classical analytical strategies such as IVW and MR-Egger regression. As the results were analogous to those of the prior forward MR analysis, the full dataset and visualisations are detailed in ‘Supplementary Materials− 6 Inverse MR analysis results’. Following the validation of the IVW method, 18 species of gut microbes were found to have statistically significant associations with ASD (*P* < 0.05), thus indicating that ASD may exert a causal effect on the abundance of these microbes. The application of MR-Egger regression, leave-one-out validation, and MR-PRESSO analysis yielded the following findings: both the MR-Egger regression intercept* P*-value and MR-PRESSO test* P*-value exceeded 0.05, while all associations successfully passed leave-one-out validation. This indicates an absence of apparent horizontal pleiotropy; however, the Q-test indicated heterogeneity in some analyses, necessitating a cautious interpretation of the results. The OR for *Lactobacillus* B *ruminis* was 1.11, suggesting that ASD may promote an increase in the abundance of this microorganism; while the OR value for *CAG-302* was 0.82, implying that ASD may lead to a decrease in its abundance.

**Fig. 7:**
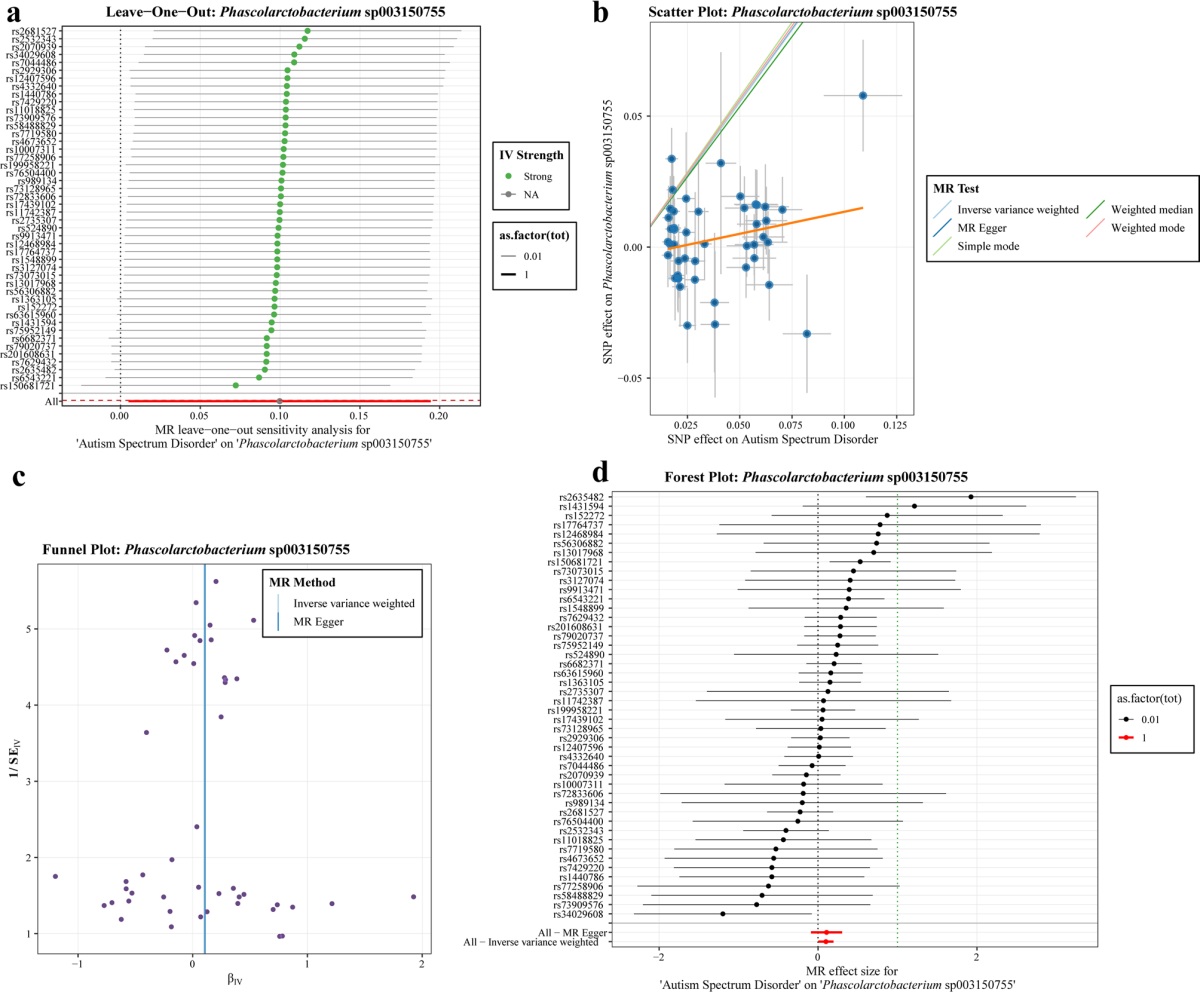
Visualisation results of reverse MR between gut microbiota (*Phascolarctobacterium* sp003150755) and ASD. For detailed explanations, please refer to the legends in Fig. 6

The reverse MR analysis employs the same visualization method as the forward MR analysis. As demonstrated in Fig. 7, the reverse MR analysis between *Phascolarctobacterium* sp003150755 and ASD yielded visualisation results. Specifically, Fig. 7a demonstrates that following the removal of a single SNP, the effect points become closer to the red line representing the entire sample. No significant interference from the single instrumental variable is evident, resulting in robust results. Figure 7b: The fitting lines of the five MR methods demonstrate consistent trends, and the causal effect estimates for ‘ASD → *Phascolarctobacterium* sp003150755’ remain stable under multiple statistical strategies. Figure 7c: The point distributions of the IVW and MR Egger’s tests demonstrate a high degree of symmetry, indicating an absence of discernible publication bias or bias due to small sample size. Figure 7d: The confidence intervals for the majority of SNPs and combined effects are not close to zero, and both single and multiple instrumental variable dimensions support stable associations. Overall, these findings indicate that the causal association between this bacterial and ASD is robust after multi-dimensional testing. Detailed data and visualisation results for other gut microbiota are available in “Supplementary Materials−6 Inverse MR analysis results”.

### Blood eQTL association analysis and pathway enrichment study of ASD-related novel loci and gut microbiota instrumental variables with common SNPs

#### Screening of common SNPs

Utilising a comprehensive approach encompassing both ASD novel loci and gut microbiota instrumental variables, along with the incorporation of significant SNPs in bidirectional MR analysis, an intersection analysis was conducted to elucidate the genetic effects that overlap. The results of the reverse MR analysis (ASD as exposure, gut microbiota as outcome) indicated the presence of 41 common SNPs (named common SNP) that were identified between the instrumental variable SNPs and novel loci; however, no overlap was detected in the forward MR analysis (gut microbiota as exposure, ASD as outcome), suggesting that the genetic effects of ASD on gut microbiota may be independent of the signals from novel loci.

#### Common SNP SMR analysis

Subsequent SMR analysis was conducted utilising common SNPs and blood eQTL data, yielding 26 significant associations (see Table 2). The original SMR* P*-value (p_SMR) and the* P*-value adjusted using the BH method (FDR_BH) were both less than 0.05, indicating a potential causal mediation effect between these variants and blood gene expression. Given that common SNPs had been pre-processed using LD pruning, pleiotropy interference could be reasonably excluded. Furthermore, SMR locus plots were generated to visualise the distribution of genetic association signals across genomic regions (see Supplementary Materials–7 Common SNP SMR results Plot).

**Table 2:** Common SNP SMR analysis results

probeID	Gene	ProbeChr	Probe_bp	topSNP	topSNP_chr	topSNP_bp	Freq	b_SMR	se_SMR	p_SMR	FDR_BH
ENSG00000091127	*PUS7*	7	105,121,411	rs150681721	7	105,050,546	0.0755467	0.788793	0.169944	3.4592E-06	5.7654E-06
ENSG00000111801	*BTN3A3*	6	26,447,171	rs989134	6	26,336,224	0.545726	0.244763	0.05325	4.2968E-06	6.7137E-06
ENSG00000112343	*TRIM38*	6	25,974,189	rs989134	6	26,336,224	0.545726	0.268453	0.0605902	9.3958E-06	1.1745E-05
ENSG00000112763	*BTN2A1*	6	26,467,499	rs989134	6	26,336,224	0.545726	− 0.356753	0.0909864	8.82E-05	8.82E-05
ENSG00000124508	*BTN2A2*	6	26,389,213	rs989134	6	26,336,224	0.545726	− 0.251219	0.0551958	5.3289E-06	7.8366E-06
ENSG00000157578	*LCA5L*	21	40,797,750	rs2735307	21	40,713,358	0.404573	0.113251	0.0205662	3.6572E-08	1.9033E-07
ENSG00000158373	*H2BC5*	6	26,164,963	rs989134	6	26,336,224	0.545726	− 0.0705567	0.0128304	3.8156E-08	1.9033E-07
ENSG00000158406	*H4C8*	6	26,283,522	rs989134	6	26,336,224	0.545726	− 0.0403858	0.0072471	2.5085E-08	1.9033E-07
ENSG00000168274		6	26,217,438	rs989134	6	26,336,224	0.545726	− 0.26184	0.0583014	7.0842E-06	9.3666E-06
ENSG00000168827	*GFM1*	3	158,386,215	rs7629432	3	157,895,285	0.389662	− 0.640163	0.132339	1.3163E-06	2.5313E-06
ENSG00000174891	*RSRC1*	3	158,043,581	rs7629432	3	157,895,285	0.389662	− 0.191757	0.0354961	6.5835E-08	2.1133E-07
ENSG00000182093	*GET1*	21	40,776,312	rs2735307	21	40,713,358	0.404573	0.0394364	0.00682604	7.5886E-09	9.4858E-08
ENSG00000182952	*HMGN4*	6	26,542,557	rs989134	6	26,336,224	0.545726	− 0.134601	0.0276464	1.1235E-06	2.3407E-06
ENSG00000183527	*PSMG1*	21	40,551,236	rs2735307	21	40,713,358	0.404573	0.228506	0.0476737	1.6421E-06	2.9323E-06
ENSG00000185658	*BRWD1*	21	40,624,793	rs2735307	21	40,713,358	0.404573	− 0.200483	0.040257	6.3564E-07	1.4446E-06
ENSG00000186470	*BTN3A2*	6	26,371,966	rs989134	6	26,336,224	0.545726	0.0804045	0.0147063	4.5678E-08	1.9033E-07
ENSG00000196966		6	26,225,613	rs989134	6	26,336,224	0.545726	0.239263	0.0555148	1.6333E-05	1.7754E-05
ENSG00000197846		6	26,200,345	rs989134	6	26,336,224	0.545726	− 0.0977073	0.0181028	6.7627E-08	2.1133E-07
ENSG00000197903	*H2BC12*	6	27,110,346	rs989134	6	26,336,224	0.545726	0.262004	0.0583513	7.1186E-06	9.3666E-06
ENSG00000198518		6	26,205,562	rs989134	6	26,336,224	0.545726	− 0.115743	0.0217319	1.0042E-07	2.7893E-07
ENSG00000205581	*HMGN1*	21	40,717,907	rs2735307	21	40,713,358	0.404573	− 0.0313818	0.00542019	7.0471E-09	9.4858E-08
ENSG00000217275		6	26,202,633	rs989134	6	26,336,224	0.545726	− 0.117888	0.0269871	1.2521E-05	1.4906E-05
ENSG00000228223	*HCG11*	6	26,524,441	rs989134	6	26,336,224	0.545726	0.15061	0.0371111	4.9422E-05	5.1482E-05
ENSG00000228223	*HCG11*	6	26,524,441	rs989134	6	26,336,224	0.545726	0.15061	0.0371111	4.9422E-05	5.1482E-05
ENSG00000241549	*GUSBP2*	6	26,881,798	rs989134	6	26,336,224	0.545726	− 0.119927	0.0276434	1.4354E-05	1.6311E-05
ENSG00000261584		6	26,687,330	rs989134	6	26,336,224	0.545726	− 0.0686969	0.0135111	3.6861E-07	9.2152E-07

ProbeChr denotes the chromosome on which the probe is located, while Probe_bp designates the base position of the probe on the chromosome. topSNP denotes the SNP most significantly associated with gene expression levels. topSNP_chr designates the chromosome where topSNP is located. topSNP_bp represents the base position of topSNP on the chromosome. Freq denotes its allele frequency. b_SMR represents the effect value in the SMR analysis, se_SMR represents the standard error of the effect value, and p_SMR represents the* P*-value of the SMR analysis. FDR_BH represents the* P*-value obtained using the BH method

#### Functional enrichment analysis of common SNPs after SMR

**Fig. 8:**
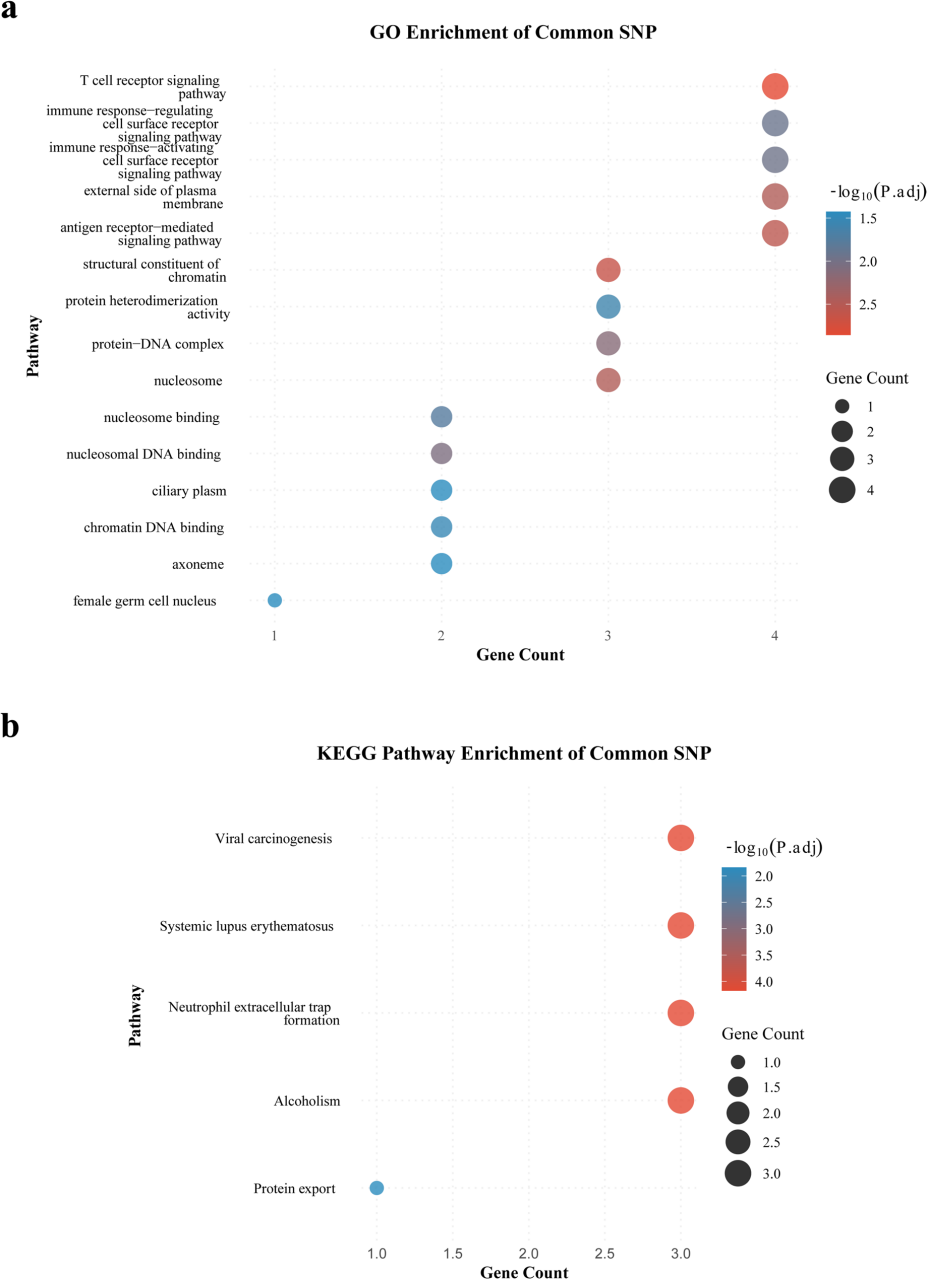
GO (**a**) and KEGG (**b**) enrichment results for common SNPs. The x-axis represents the number of genes enriched in the corresponding pathway; the higher the value, the more risk genes are associated with that function; The bubble colour represents the − log_10_(*P*.adj) value of enrichment significance (*P*.adj is the* P*-value after multiple testing correction), with darker colours indicating a more significant association between the functional entry and ASD risk; bubble size is positively correlated with the number of enriched genes, with larger bubbles representing more genes involved

GO and KEGG pathway enrichment analysis was performed on the SMR results obtained above to reveal their potential biological functions. The results are displayed in the accompanying Fig. 8. The GO enrichment pathways that demonstrate significant enrichment are predominantly concentrated in the “T cell receptor signalling pathway” and “immune response - activation of cell surface receptor signalling pathway” pathways, as indicated by the use of darker colours and a larger number of genes. This finding suggests that common SNPs are highly enriched in immune cell activation and signal transduction. The results of the KEGG analysis indicated that common SNPs were predominantly enriched in pathways such as “Viral carcinogenesis,” “Systemic lupus erythematosus,” “Neutrophil extracellular trap formation,” and “Alcoholism.” The findings from both analyses consistently demonstrate that common SNPs are predominantly enriched in immune regulation and immune response pathways, thereby indicating a potential influence on the pathogenesis of ASD. This influence may be exerted through the following pathways: gut microbiota → abnormal blood immune pathways (e.g., T cell activation, neutrophil dysfunction) → neuroimmune interactions → ASD pathophysiological processes. This finding provides genetic evidence for the role of the “gut-immune-brain axis” in ASD, suggesting that targeting immune pathways may offer a new intervention strategy linking gut microbiota to neurodevelopmental abnormalities.

### Key findings: dual validation and pleiotropic mechanisms of cross-organizational regulatory hubs

It is noteworthy that the two SNPs, rs2735307 and rs989134, manifest pleiotropic characteristics of trans-tissue regulation. They not only demonstrate significant genetic effects in the validation analysis of brain function dimensions, but both have also been confirmed to directly participate in neuron differentiation, synapse formation, and chromatin dynamic regulation through cis-regulation of neighbouring genes or epigenetic modifications, which is highly consistent with the pathological features of abnormal neural circuit connections in the brains of ASD patients. Furthermore, these loci have also been identified as key regulatory sites in immune pathways in the SMR analysis of common SNPs and blood eQTL. Among the identified variants, rs2735307 is significantly enriched in the autoimmune disease pathway Systemic lupus erythematosus (hsa05322). This finding suggests the potential for rs2735307 to induce abnormal immune attacks by disrupting the immune tolerance mechanism. It is evident that rs989134 is successively enriched in a number of significant signalling pathways, including the T cell receptor signalling pathway (GO:0050852), the antigen receptor-mediated signalling pathway (GO:0050851), the immune response-regulating cell surface receptor signalling pathway (GO:0002768), and neutrophil extracellular trap formation (hsa04613). This finding thus reveals a comprehensive immune activation chain, which spans the entire process from the initiation of T-cell antigen recognition signals to the formation of neutrophil extracellular traps. The two pathways synergistically activate immune cell signalling, regulate pro-inflammatory cytokine expression, and modulate neutrophil function, thereby triggering systemic inflammatory responses and exacerbating neuroinflammation through the blood-brain barrier. This significant brain-immune dual-system association signal was validated through rigorous LD pruning and multi-dataset cross-validation, thereby eliminating false-positive biases from single-organ tissue testing. Consequently, a cross-system pathogenic network linking “neurodevelopment-immune regulation” at the genetic level was established. Combined with the genetic association between gut microbiota and ASD identified in MR analysis, this suggests that the two variants may act as common genetic drivers of the “gut-immune-brain axis,” forming a pathological cascade of “microbiota imbalance → immune activation → neural damage” through cross-tissue regulatory networks. This provides an integrated explanatory framework for the multidimensional pathogenesis of ASD.

## Discussion

The present study identified two potential variants associated with an increased risk of ASD, namely rs2735307 and rs989134, through a multi-omics cross-organ integration analysis. This analysis revealed the synergistic regulatory networks of these variants in the brain, gut, and immune system. These key variants exert their effects through a triple mechanism: regulating the expression of neurodevelopmental genes, modulating gut microbiota composition, and activating immune pathways, thereby forming a positive feedback loop within the “gut-immune-brain axis” that drives the pathological progression of ASD. This finding unveils a systemic pathogenic mechanism that conventional single-brain-tissue GWAS studies are unable to capture.

Firstly, following the execution of PoPS analysis on novel loci in the present study, key clues were identified through GO and KEGG functional enrichment analysis: The results of the KEGG pathway analysis indicated that the “tight junction” pathway exhibited the highest gene count and the most significant statistical evidence (*P*.adj < 0.001). This finding suggests the possibility that genetic variations associated with ASD may mediate cross-tissue pathologies by impacting the function of intercellular barriers. This finding is in close alignment with the clinical characteristics of ASD. Epidemiological studies have indicated that individuals diagnosed with ASD frequently exhibit gastrointestinal symptoms, including altered bowel habits and abdominal discomfort (Horvath and Perman 2002; Ristori et al. 2019; WangTancredi & Thomas 2011). Defects in intestinal epithelial tight junctions have been identified as one of the core mechanisms underlying gastrointestinal dysfunction (Gupta et al. 2017). From the perspective of cross-system interaction mechanisms, impaired intestinal tight junctions have been demonstrated to trigger microbiota translocation, which logically correlates with the KEGG-annotated pathways “Salmonella infection” and “pathogenic Escherichia coli infection”: excessive proliferation of intestinal pathogens has been shown to directly damage the intestinal mucosal barrier and to release pro-inflammatory substances such as lipopolysaccharides into the circulatory system via the “leaky gut-inflammation” pathway (Srikantha and Mohajeri 2019). Concurrently, aberrations in tight junction proteins of the blood-brain barrier may permit peripheral inflammatory factors (e.g. IL-6 and TNF-α) to penetrate the barrier, activate microglia in the brain, and induce neuroinflammation (Greene et al. 2022; Takata et al. 2021). This, in turn, can trigger GO enrichment analysis-related biological processes such as “regulation of neuronal apoptosis” and “abnormal neuronal migration,” thereby establishing a complete causal chain. It is noteworthy that the inflammatory cascade may further influence the “oxytocin signalling pathway” in GO enrichment analysis. The hypothesis is that insufficient oxytocin in the brain may weaken the amygdala-prefrontal cortex pathway’s response to social stimuli, thereby affecting the recognition and processing of social cues in individuals with ASD (Fineberg and Ross 2017). The dual role of tight junction pathways in the intestinal barrier and blood-brain barrier, driven by genetic variations, forms a cross-system pathogenic loop of “intestinal barrier damage → peripheral inflammation → brain barrier damage → neurodevelopmental disorders.” The significant enrichment of novel loci in the aforementioned pathways in this study provides direct genetic evidence for the “gut-immune-brain axis” synergistic pathogenic mechanism in ASD, suggesting that restoring tight junction function through drugs or probiotics may be a key intervention strategy to block the pathological process.

Next, in the subsequent analysis of eQTL-specific enrichment at novel loci across brain regions and cell types, the key variant rs2735307 exhibited significant cross-brain region and cell type distribution characteristics. This variant was found to be significantly enriched in 12 brain regions (with the exception of the cerebellum) and 8 brain cell types. This finding suggests that its regulatory effects exhibit characteristics of cross-brain network properties. This finding provides a genetic explanation for the phenotypic diversity of ASD, by demonstrating that the functional effects of the same genetic variant in different brain regions (e.g. the prefrontal cortex and striatum) may lead to heterogeneous clinical manifestations in patients. Those with prefrontal cortex involvement primarily exhibit social interaction deficits, while those with striatal dysfunction are more likely to exhibit repetitive and stereotyped behaviours (AglinskasHartshorne & Anzellotti 2022). The results presented herein provide direct genetic evidence for endophenotypic stratification in ASD, thereby supporting the feasibility of classifying patients through brain region-specific molecular markers. In comparison, the rs989134 variant exhibits a distinct brain region-specific enrichment pattern, primarily concentrated in the Brain_Cerebellum, Brain_Cortex, and Brain_Frontal_Cortex_BA9. These three brain regions have been identified as hubs for motor-cognitive regulation, the core of higher neural functions, and the centre for social behaviour control, respectively. Their synergistic abnormalities may collectively drive functional impairments in ASD patients across multiple dimensions, including motor coordination, language expression, and social behavior, through defects in the “cerebellum-cortex-frontal cortex” neural circuit. The specific enrichment of rs989134 in these key brain regions suggests that related genetic variations may disrupt the normal development and functional homeostasis of neural circuits by regulating region-specific gene expression or epigenetic modifications. The coexistence of “brain-wide regulation” and “region-specific effects” further reveals the complex interplay between “common foundations” and “individual differences” in the genetic mechanisms of ASD, providing a multidimensional perspective for elucidating disease heterogeneity.

Then, following the integration of relevant research data through meta-analysis, a bidirectional MR analysis of gut microbiota and ASD was conducted. In the forward MR analysis, gut microbiota was used as the exposure factor to explore its causal influence on ASD onset; in the reverse MR analysis, ASD was used as the starting point to explore the effect of ASD on the structure and abundance of the gut microbiota. The results of the forward MR analysis demonstrated that certain prevalent microbial communities manifested statistically significant effect values. For instance, the OR for *Enterococcus faecalis*, *Parabacteroides*, and *Coprobacillus* were less than 1. This finding suggests that, under normal physiological conditions, an increase in the abundance of these microorganisms may be associated with a reduced risk of ASD onset. It has been hypothesised that they may play a protective role in ASD through physiological functions such as producing short-chain fatty acids, regulating metabolism, maintaining intestinal microecological balance, and regulating immune function. Conversely, the OR value for *Francisellaceae* (*Francisella*) exceeds 1, signifying that an augmentation in its prevalence could potentially elevate the risk of ASD emergence, possibly via mechanisms such as instigating intestinal inflammation that contributes to the pathological progression of ASD. The results of the reverse MR analysis are equally intriguing, with *Lactobacillus* B (a specific strain of the *Lactobacillus* genus), *Enterococcus* A, *Enterococcus* B (strains related to the *Enterococcus* genus), and *Faecalibacterium* sp002160895 (a specific strain of the *Faecalibacterium* genus) identified as gut microbiota with a significant causal relationship to ASD, and their OR values all exceeding 1. This suggests that the presence of ASD is associated with an increase in the abundance of these microorganisms. The hypothesis that this phenomenon occurs as a result of the distinctive physiological and pathological characteristics exhibited by individuals diagnosed with ASD, including gastrointestinal dysfunction and immune-inflammatory imbalance, is a subject of current research. These conditions are believed to engender an environment more conducive to the proliferation of these microorganisms, ultimately resulting in their overabundance within the gastrointestinal tract. Furthermore, changes in the abundance of these microorganisms may in turn feed back and influence the progression of ASD symptoms, forming a complex interactive network. Furthermore, it is noteworthy that the significant gut microbial OR values identified in this study generally approached 1. This phenomenon aligns closely with the ecological characteristics of the gut microbiome: the gut microbiota constitutes a highly interconnected network, where compensatory actions from other microbial communities often dilute the effects of individual microbes. For instance, lactic acid production by *Enterococcus faecalis* may be reduced due to competitive substrate utilisation by symbiotic bacteria such as *Bifidobacterium*, resulting in a statistically weaker protective effect when considered in isolation. Furthermore, individual variations such as host genetic background and dietary habits may further diminish the association strength of individual microbial communities at the population level, rendering them less likely to exhibit strong effects. Nevertheless, this “weak association” retains significant public health and clinical importance. In the case of *Fibrobacteria* (OR = 1.15), when combined with its prevalence of approximately 1% among children with high ASD incidence, the population attributable risk is calculated to be around 0.11%. This equates to approximately 1100 ASD cases per million target children being attributable to the abnormal abundance of this bacterium. While the impact of individual microbiota appears negligible, from an intervention perspective, such microbiota are precisely suited for regulation through low-cost measures (such as targeted prebiotics or composite probiotics). This could achieve a moderate reduction in ASD risk at the population level, providing a viable entry point for primary prevention.

Subsequently, the core variants rs2735307 and rs989134 were identified, exhibiting significant effects across multiple dimensions. These variants have been shown to reveal the cross-system pathogenic mechanisms of ASD from the dimensions of chromatin regulation-immune interaction and transcriptional regulation-immune drive, respectively. In particular, rs2735307 has been identified as a crossroads hub between brain chromatin regulation and blood immune pathways, influencing neural development through the following dual pathways: on one hand, this variant modulates the spatial expression of the nearby gene *HMGN1* (a chromatin structure regulator (Yang et al. 2023) via cis-regulation, indirectly regulating the spatiotemporal expression of *BRWD1* (a gene involved in neuronal differentiation and synaptic function (Fulton et al. 2022), disrupting neuronal-specific transcriptional programs, exacerbating synaptic pruning defects, and inducing abnormal neural circuit connections, which highly aligns with the typical pathological features of ASD (Chen et al. 2018); On the other hand, SMR analysis of blood eQTLs revealed that this variant is enriched in the systemic lupus erythematosus pathway, suggesting potential immune tolerance imbalance and abnormal production of autoantibodies, which may induce antibody attacks on synaptic-related proteins in the brain (DaiFan & Zhao 2025). In combination with the previously identified abnormalities in the tight junction pathway, the resultant damage to the intestinal and blood-brain barriers enables the translocation of intestinal microbiota and their derivatives into the brain. This process then engenders continuous activation of chronic neuroinflammation, thereby establishing a vicious cycle of “intestinal leakage - immune attack - neuroinflammation”. Similarly, rs989134 can be regarded as a dual driver of neural transcriptional regulation and systemic immune abnormalities. The rs989134 has been hypothesised to participate in the pathological process of ASD through the “neural transcription-systemic immunity” interaction mechanism. In the brain, the variant modulates the epigenetic state of the cis-regulatory pseudogene *H3C9P* (a histone modification mimic) by recruiting chromatin remodelling complexes. This, in turn, affects the promoter activity of the adjacent functional gene *ABT1* (basic transcription initiation factor (Brower et al. 2010). The consequence of this imbalance is a loss of transcriptional homeostasis in neurons of the prefrontal cortex BA9 region. This is characterised by the repression of genes associated with synaptic plasticity and irregularities in genes involved in neuronal migration, consequently impairing social cognitive neural circuits and disrupting the layered distribution of cortical neurons. In the context of the blood immune pathway, this variant has been found to be significantly associated with both the T cell receptor signalling pathway and the neutrophil extracellular trap formation pathway. It is speculated that it may promote the expression of pro-inflammatory factors such as IL-6 by regulating the chromatin state of CD4^+^ T cells, thereby breaching the blood-brain barrier and triggering neuroinflammation (Williams et al. 2021). Additionally, it may induce neutrophils to release excessive extracellular traps, exacerbating immune attacks on neural synapses (Tang et al. 2024), thereby forming a vicious cycle of neuroimmune imbalance in ASD patients. As core variants of novel loci, rs2735307 and rs989134 respectively elucidate the molecular mechanisms underlying the “gut-immune-brain” cross-system pathogenesis of ASD from the dimensions of “chromatin regulation - immune attack” and “transcriptional homeostasis–immune dysfunction”. The former is characterised by the driving of neuroinflammation through the disruption of barriers and autoimmune attacks, while the latter is marked by the amplification of pathological damage through transcriptional disruption and immune-neural interactions. Collectively, these findings suggest that genetic variations may contribute to the multifaceted presentation of ASD through synergistic effects across multiple pathways and tissues. This provides molecular targets and theoretical underpinnings for subsequent precision intervention strategies, focusing on epigenetic regulation and immune modulation.

In addition to the two aforementioned specific potential ASD risk variants, the novel loci identified in this study through PoPS scoring (> 0.08) also involve multiple potential genetic risk loci and corresponding genes. The association of these genes with ASD can be systematically interpreted from multiple perspectives, including molecular mechanisms, neurodevelopment, and cross-species studies. Specifically, although *NEDD4L* (rs63615960) has not been directly proven to be associated with ASD, its core function as an E3 ubiquitin ligase provides indirect clues for elucidating ASD mechanisms: Abnormalities in the ubiquitin pathway have been reported to be involved in ASD-related deficits in synaptic plasticity (Kasherman et al. 2020), and *NEDD4L* participates in neurodevelopment by regulating neuronal axon pruning and dendritic spine maturation (Rodrigues et al. 2020). Its functional abnormalities may influence brain network formation through mechanisms that are similar to those previously described. The potential role of *NTM* (rs524890) has been linked to chromosomal micro-rearrangements and cross-species expression patterns. While no significant association has been identified between *NTM* single nucleotide polymorphisms and ASD, micro-rearrangements in the 11q24.2-25 region (including *NTM*) have been associated with increased subcortical volume, reduced occipital grey matter volume, and other brain structural abnormalities in ASD patients (Maruani et al. 2015). Furthermore, as a member of the IgLON family, the zebrafish homologue of *NTM* is specifically expressed in conserved brain regions and ASD-related brain regions (Habicher et al. 2024), suggesting a potential role in pathogenesis through the regulation of neural circuit development. Further validation of the synergistic effects among genes in this region is required. The mechanism of *ATXN1* (rs72833606) has been elucidated to a greater extent: the complex formed with the CIC protein plays a pivotal role in the maturation of upper cortical neurons and the regulation of social behaviour. Mice afflicted with *ATXN1* deficiency have been observed to manifest symptoms of hyperactivity, social abnormalities, and deficits in learning and memory. In human subjects, the presence of heterozygous truncating mutations in CIC frequently gives rise to ASD and other neurodevelopmental disorders, thereby indicating that abnormalities within the ATXN1-CIC pathway may constitute a pathogenic pathway for ASD (Lu et al. 2017). In comparison, the existing evidence for *PRKACB* (rs77258906) and *NKAIN3* (rs1431594) is relatively limited. The former, as a core component of the cAMP signalling pathway, is involved in regulating synaptic plasticity (Mo et al. 2024; Tchilikidi 2024). Abnormal synaptic plasticity is one of the core pathological features of ASD. This suggests that *PRKACB* may indirectly contribute to ASD pathogenesis via this pathway, though direct associative evidence is currently lacking. The latter (*NKAIN3*) has not yet been clearly linked to ASD and requires further support from cross-omics data. In summary, the aforementioned genes collectively delineate a cross-molecular network of genetic risk for ASD through multiple mechanisms, including ubiquitination regulation (*NEDD4L*), neural circuit development *(NTM*, *ATXN1*), and signal pathway transmission (*PRKACB*). Although the association of certain genes (e.g. *NKAIN3*) still requires validation, this study provides significant candidate directions for subsequent mechanism validation (e.g. cell models or animal models) and drug target screening by employing an advanced similarity-based gene prioritisation PoPS scoring method for annotation.

Furthermore, the study revealed that the PoPS scores for *POU4F1* (rs4332640) and *LRP1B* (rs72855404) were negative, suggesting a potential protective effect or function by antagonising the pathological mechanisms of ASD. The *POU4F* gene family, to which *POU4F1* belongs, plays a central role in neural development: its homologous gene *POU4F2* (*Brn3b*) acts as a key transcription factor, regulating the fate specification, differentiation, and survival of retinal ganglion cells (Badea et al. 2009; Wu et al. 2015). The functional loss of *POU4F2* has been demonstrated to result in neuronal cells undergoing a transition to a non-neuronal fate, accompanied by the process of apoptosis and degeneration (Xiang et al. 2011). Despite the indirect nature of the association between *POU4F1* and ASD, the evidence from studies of its conserved functions in neural circuit formation, axon growth, and cell fate determination (Xiang et al. 2011) suggests a potential for this family to contribute to ASD genetic susceptibility by influencing early neural developmental networks. *LRP1B*, a member of the low-density lipoprotein receptor family, has been demonstrated to possess several biological functions. Not only is it involved in endocytosis, signal transduction and neural development processes, but it also functions as a tumour suppressor gene and can exert neuroprotective effects by inhibiting Aβ production (Príncipe et al. 2021). Despite the ambiguity surrounding the precise mechanisms of *LRP1B* in ASD, a study of monozygotic/dizygotic twins demonstrated a statistical correlation between ASD-related SNPs and the *LRP1B* gene (HuDevlin & Debski 2019), thereby implying a potential indirect influence on ASD risk through the regulation of neuronal migration or synaptic plasticity. It is noteworthy that the negative score of *LRP1B* corresponds to its neuroprotective function, indicating that enhanced activity may counteract ASD-related neurodegenerative or synaptic developmental abnormalities. In summary, the negative scores of *POU4F1* and *LRP1B* reveal the bidirectional nature of ASD genetic mechanisms—the former potentially exerts a protective effect by maintaining the integrity of neural developmental programs, while the latter may antagonize pathological processes through multiple neuroprotective mechanisms. These findings provide new evidence for a polygenic regulatory model of ASD. Future studies should combine single-cell transcriptomics analysis and functional knockout/overexpression experiments to further elucidate their specific roles in the neuro-immune-gut cross-system network.

### Limitations

Firstly, due to the relatively limited sample size of the case group in existing autism GWAS data, this study integrated four independent GWAS datasets for meta-analysis to enhance statistical power and the reliability of results. Nevertheless, it is important to note that imbalances in sample size may still have a detrimental effect on the ability to detect rare variants.

Secondly, in terms of gut microbiome analysis, this study utilised a recently published comprehensive database covering 473 gut microbiome species to comprehensively assess microbiome-host interactions. However, due to the current limitations in the completeness of microbial genome annotations, it is possible that certain low-abundance or understudied microbial communities may not have been included in the analysis. This necessitates further study to expand the database coverage.

Thirdly, all genetic and microbiome data used in this study were derived exclusively from individuals of European ancestry. This demographic homogeneity may limit the generalizability of the findings to populations of other racial/ethnic backgrounds (e.g., East Asian, African, or Hispanic populations), as genetic architectures, gut microbiome compositions, and their associations with autism can vary across ancestral groups. Future studies should incorporate multi-ancestry datasets to validate the cross-population consistency of microbiome-disease associations, thereby improving the universality of the conclusions.

Furthermore, in exploring functional mechanisms, this study exclusively conducted enrichment and SMR analyses based on eQTL data from a solitary source of brain tissue and blood, which may have resulted in the potential oversight of biases introduced by tissue specificity or technical batches. It is therefore recommended that future studies integrate eQTL data from multiple centres and platforms in order to enhance the generalizability of conclusions.

Finally, the results of this study were primarily derived from bioinformatics analysis. Further validation of these findings is required through the implementation of in vitro or in vivo experiments.

## Conclusions

This study addresses certain limitations of single-organ association analyses by integrating GWAS and multi-omics data. The constructed model was validated through multi-dimensional verification, thereby supporting the polygenic hypothesis and identifying potential regulatory loci, including rs2735307 and rs989134. The results of the study suggest that the pathogenesis of ASD may be associated with the interaction of systems, including neurodevelopment, immune regulation, and gut microbiota. These findings provide new insights into the pathogenesis of neuropsychiatric disorders and offer theoretical references for future precision medicine research by preliminarily elucidating the association between “genetic variation-cross-tissue regulation-pathological process.”

## Supplementary Information

Below is the link to the electronic supplementary material.

Supplementary Material 1

Supplementary Material 2

## Data Availability

The public data utilised in this study are delineated in the preceding section entitled “Methods”. Furthermore, all data analysed in this study is available upon reasonable request to the corresponding author.

## Abbreviations

ASD: Autism spectrum disorder
GWAS: Genome-wide association studies
PoPS: Polygenic priority score
SMR: Summary-data-based mendelian randomization
SNPs: Single-nucleotide polymorphisms
MR: Mendelian randomization
GO: Gene ontology
KEGG: Kyoto encyclopedia of genes and genomes
FDR: False discovery rate
BH: Benjamini-Hochberg
eQTL: Expression quantitative trait locus
cis-eQTL: Cis-expression quantitative trait loci
mQTL: Methylation quantitative trait loci
LD: Linkage disequilibrium
IVW: Inverse variance weighting
OR: Odds ratio
