# Correction: Multi-omics reveals cross-tissue regulatory mechanisms of autism risk loci via gut microbiota-immunity-brain axis

**DOI:** 10.1186/s13568-025-01992-5

**Published:** 2025-12-10

**Authors:** Xingxing Liao, Junzi Long, Xianna Wang, Kaiyue Han, Zhiqing Tang, Jiarou Chen, Yan Zhang, Hao Zhang

**Affiliations:** 1 School of Rehabilitation, Capital Medical University, Beijing, China; 2 Beijing Bo’ai Hospital, China Rehabilitation Research Center, Beijing, China; 3 Changping Laboratory, Beijing, China; 4 National Autism Rehabilitation Research Center, China Rehabilitation Research Center, Beijing, China; 5 Wenzhou Medical University, Wenzhou, China; 6 Cheeloo College of Medicine, Shandong University, Jinan, China; 7 University of Health and Rehabilitation Sciences, Qingdao, China

**Correction to: AMB Express (2025) 15:161** 10.1186/s13568-025-01969-4

In this article (Liao et al. 2025), the authors wish to correct an error in the author affiliations. The complete affiliation details for all authors (Xingxing Liao, Junzi Long, Xianna Wang, Kaiyue Han, Zhiqing Tang, Jiarou Chen, Yan Zhang, Hao Zhang) have been updated with this correction which does not affect the scientific conclusions of the article.
