# Supplementary material for: Multi-omics reveals cross-tissue regulatory mechanisms of autism risk loci via gut microbiota-immunity-brain axis
Source: AMB Express. 2025 Oct 29;15:161. doi: 10.1186/s13568-025-01969-4 (PMC12572420; doi:10.1186/s13568-025-01969-4)
Supplement: Supplementary file 2 — Supplementary Material 2 [file 13568_2025_1969_MOESM2_ESM.zip › Revised supplementary materials/1 GO and KEGG enrichment results for novel loci of ASD/1 GO and KEGG enrichment results for novel loci of ASD.docx]

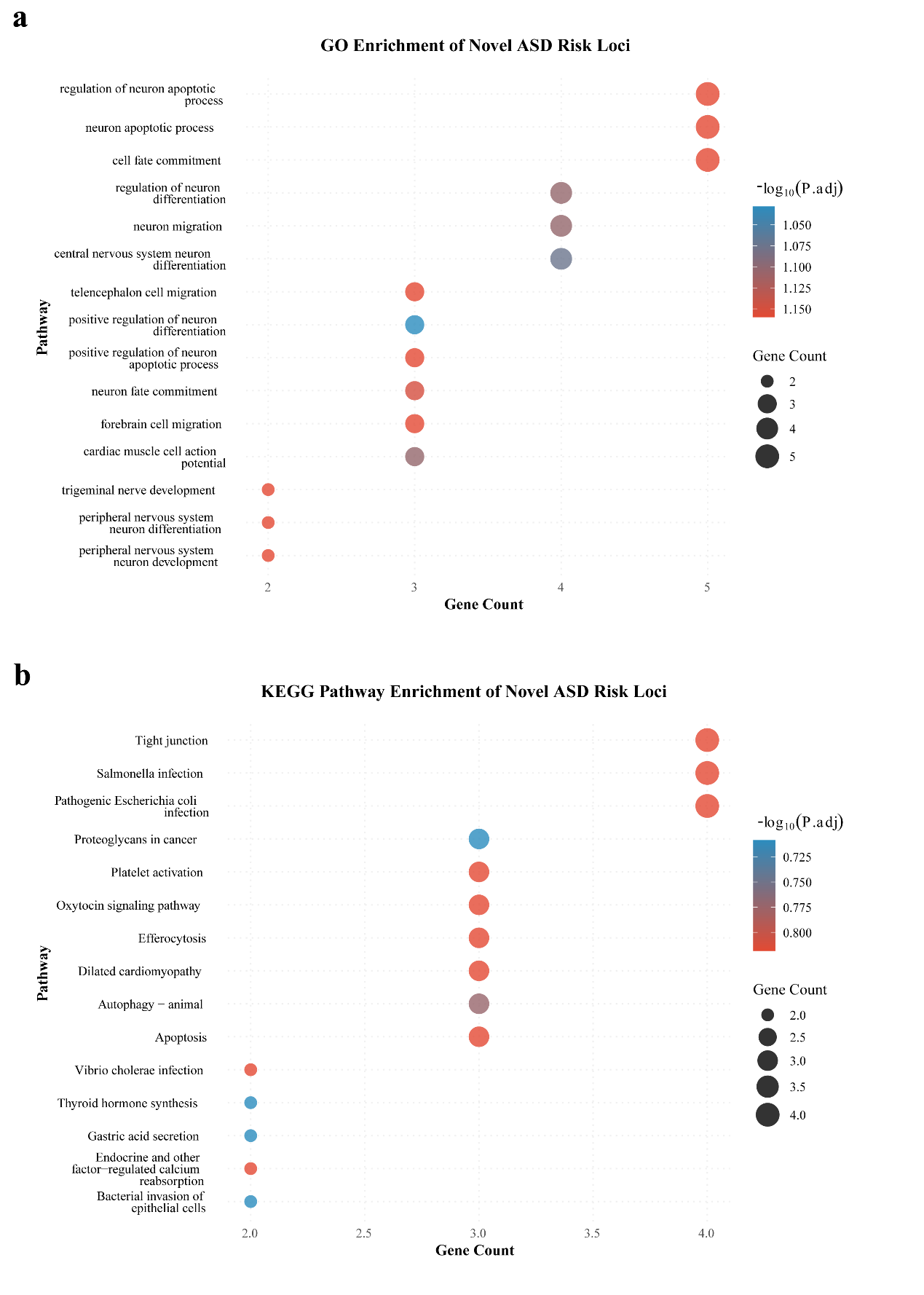


GO (a) and KEGG (b) enrichment results for novel loci of ASD. The x-axis represents the number of genes enriched in the corresponding pathway; the higher the value, the more risk genes are associated with that function; The bubble colour represents the -log₁₀(P.adj) value of enrichment significance (P.adj is the P-value after multiple testing correction), with darker colours indicating a more significant association between the functional entry and ASD risk; bubble size is positively correlated with the number of enriched genes, with larger bubbles representing more genes involved.
