# Supplementary material for: Multi-omics reveals cross-tissue regulatory mechanisms of autism risk loci via gut microbiota-immunity-brain axis
Source: AMB Express. 2025 Oct 29;15:161. doi: 10.1186/s13568-025-01969-4 (PMC12572420; doi:10.1186/s13568-025-01969-4)
Supplement: Supplementary file 2 — Supplementary Material 2 [file 13568_2025_1969_MOESM2_ESM.zip › Revised supplementary materials/3 enrichment results of novel loci in the brain cell eQTL data/Enrichment level plot of novel loci in brain cell eQTLs data.docx]

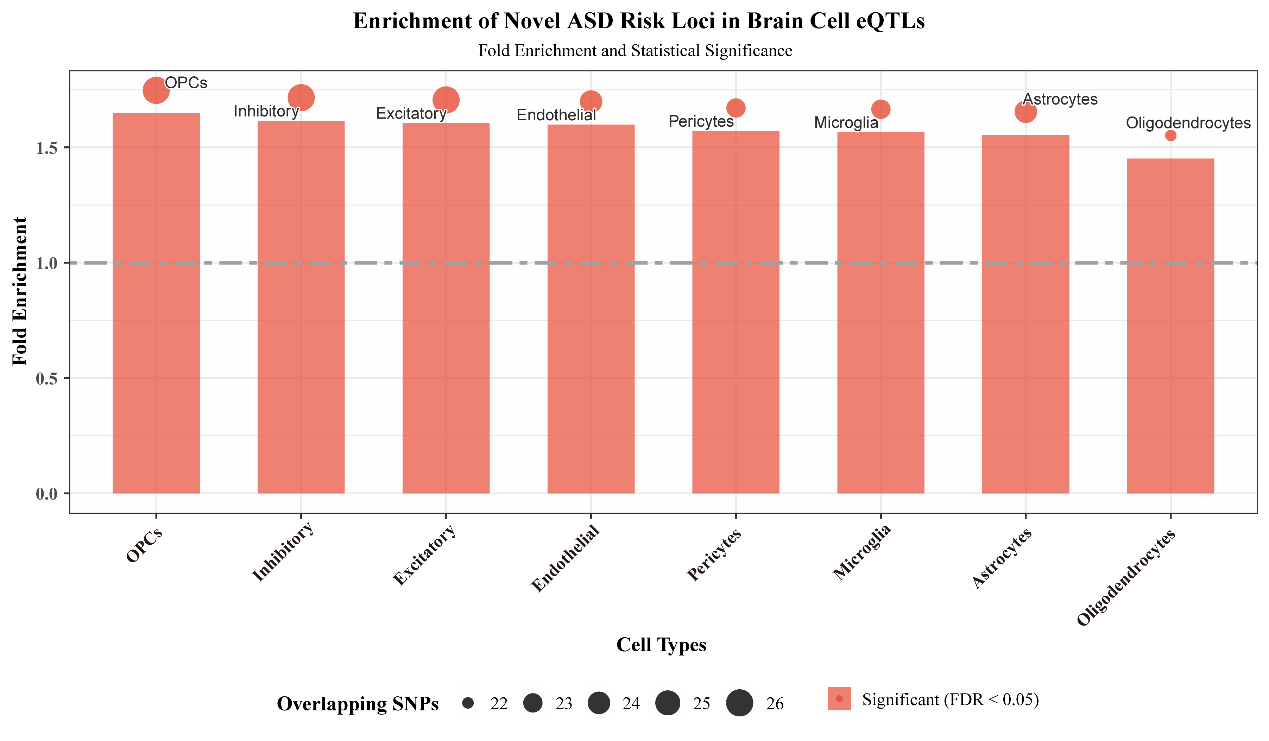


Enrichment level plot of novel loci in brain cell eQTLs data. The x-axis represents cell types within the brain; The y-axis represents enrichment multiples, reflecting the enrichment level of novel loci in eQTLs of corresponding cell types (>1 indicates enrichment, with higher values indicating more significant enrichment); Bar chart colours: Red indicates significant enrichment (FDR < 0.05), meaning that the association between eQTLs in that cell type and ASD novel loci is statistically significant; Different-sized dots correspond to the number of overlapping SNPs; larger dots indicate more eQTL-SNP overlaps with novel loci in that cell type.
