# Supplementary material for: Multi-omics reveals cross-tissue regulatory mechanisms of autism risk loci via gut microbiota-immunity-brain axis
Source: AMB Express. 2025 Oct 29;15:161. doi: 10.1186/s13568-025-01969-4 (PMC12572420; doi:10.1186/s13568-025-01969-4)
Supplement: Supplementary file 2 — Supplementary Material 2 [file 13568_2025_1969_MOESM2_ESM.zip › Revised supplementary materials/4 Novel loci SMR results/plot/legends.docx]

SMR locus plot: x-axis: The term "chromosomal physical location" is used to denote the genomic interval in which the target gene is located; The left vertical axis (Y_1_ axis) is defined as follows: log_10_(P GWAS or SMR) is a metric employed to ascertain the significance level of GWAS or SMR analysis, a greater value indicates a smaller P-value and stronger significance; The right vertical axis (Y_2_ axis) is defined as follows: The -log_10_(P eQTL) metric is employed to denote the statistical significance of eQTL analysis, which is utilised to evaluate the strength of the correlation between gene expression and SNPs; The distribution of other gene names along the horizontal axis is indicative of the distribution characteristics of gene clusters, aiding in the identification of functionally significant regions of genetic association. Effect sizes plot: Show the association between GWAS effect sizes and eQTL effect sizes to illustrate whether the influence of SNPs on phenotypes is mediated by gene expression. Each point represents a SNP-probe pair, where: x-axis: eQTL effect size (the degree of influence of SNPs on gene expression); y-axis: GWAS effect size (the degree of influence of SNPs on phenotypes); The dashed line aids in judging the magnitude of the effect size; the further the deviation, the stronger the effect; Points marked with “top cis-eQTL” represent the most significant eQTL in that region and are priority candidate sites for validation.
