# Supplementary figures and images for: Multi-omics reveals cross-tissue regulatory mechanisms of autism risk loci via gut microbiota-immunity-brain axis (part 2 of 2)
Source: AMB Express. 2025 Oct 29;15:161. doi: 10.1186/s13568-025-01969-4 (PMC12572420; doi:10.1186/s13568-025-01969-4)

# Leave-One-Out (OR): CAG-884

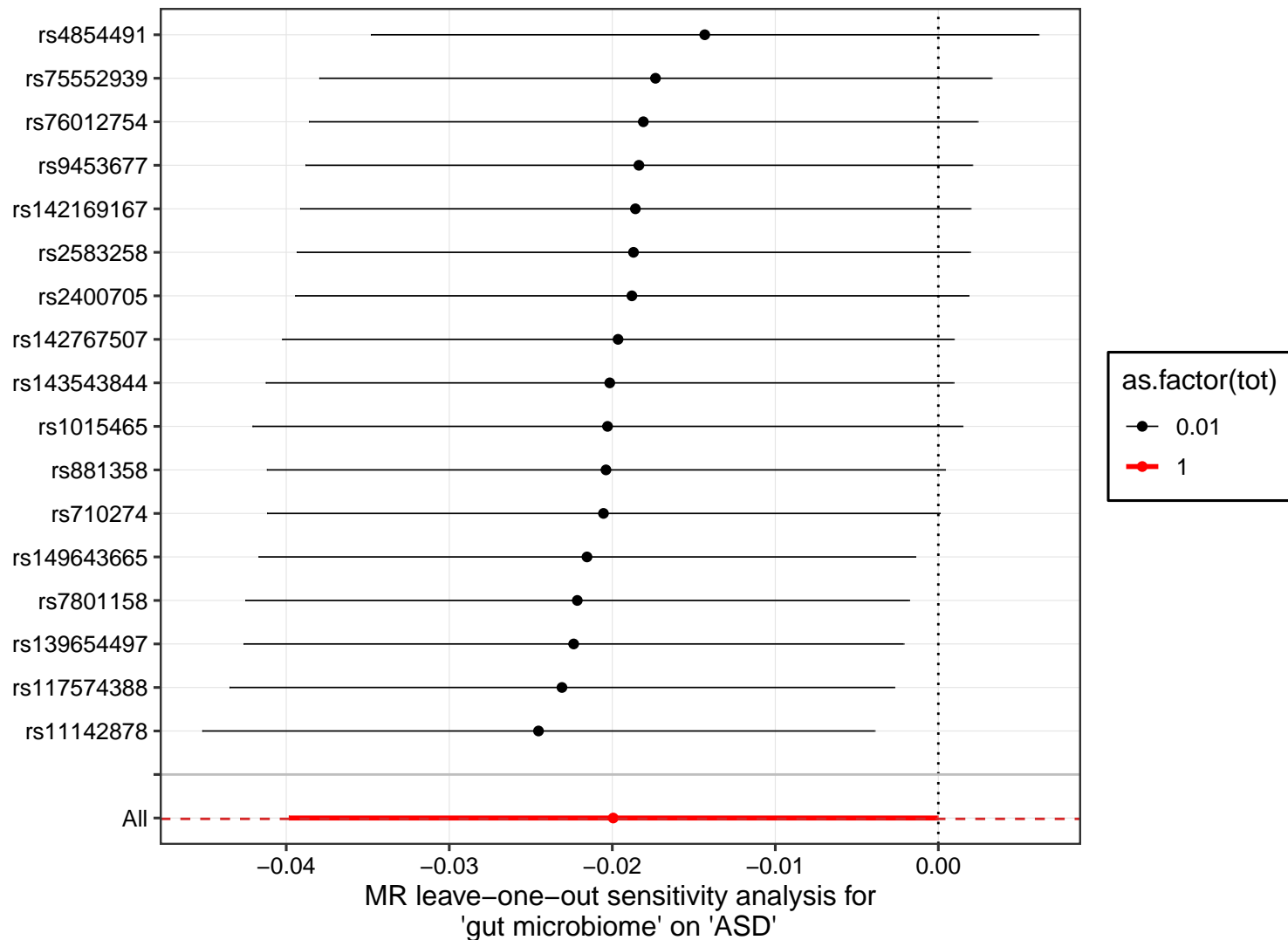

Supplement: Supplementary file 2 — Supplementary Material 2 [file 13568_2025_1969_MOESM2_ESM.zip › Revised supplementary materials/5 Forward MR analysis results/plot/leaveoneout_or_CAG-884.pdf]

# Leave-One-Out (OR): Coprobacillus

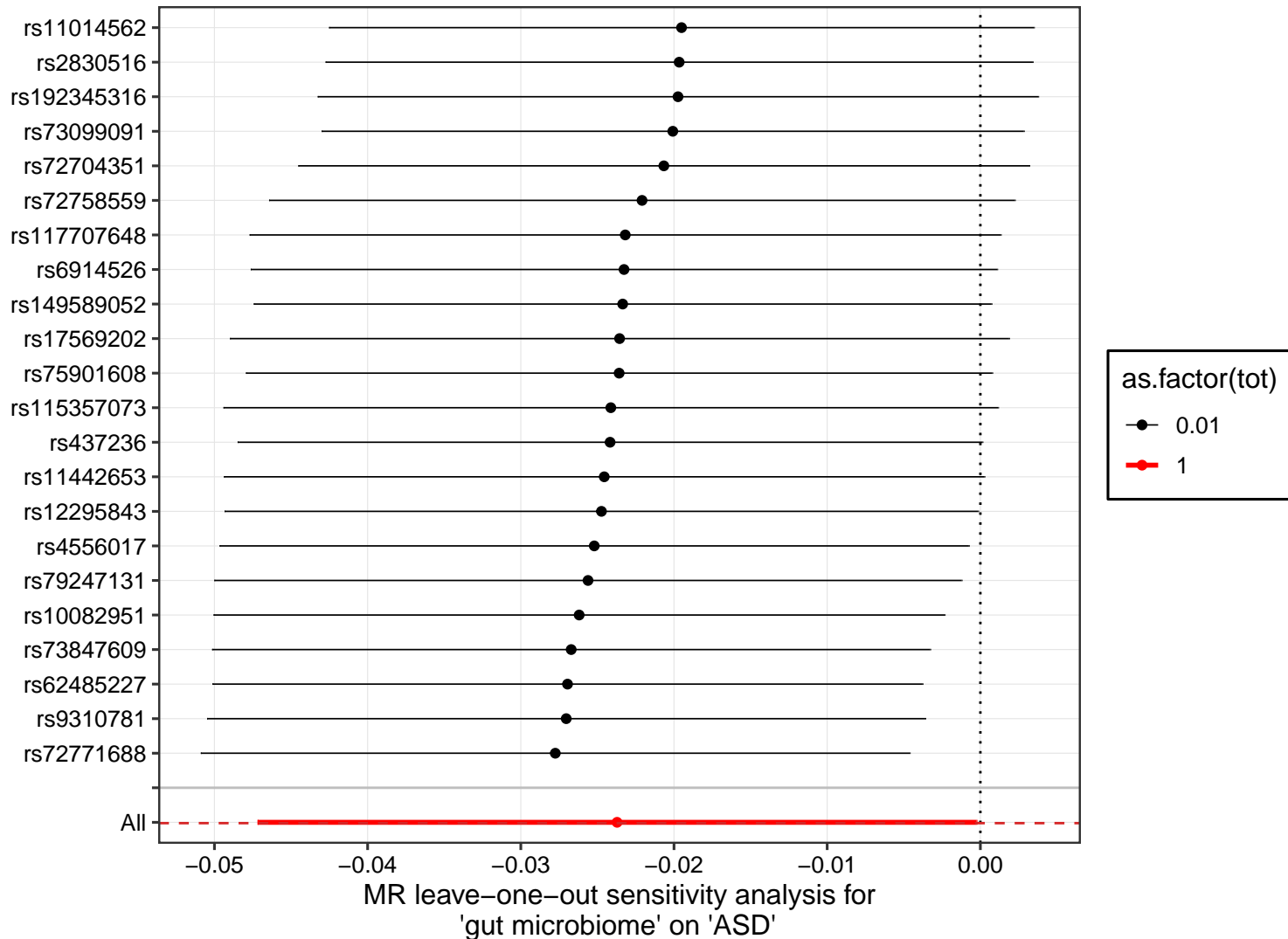

Supplement: Supplementary file 2 — Supplementary Material 2 [file 13568_2025_1969_MOESM2_ESM.zip › Revised supplementary materials/5 Forward MR analysis results/plot/leaveoneout_or_Coprobacillus.pdf]

# Leave-One-Out (OR): *Coprobacter secundus*

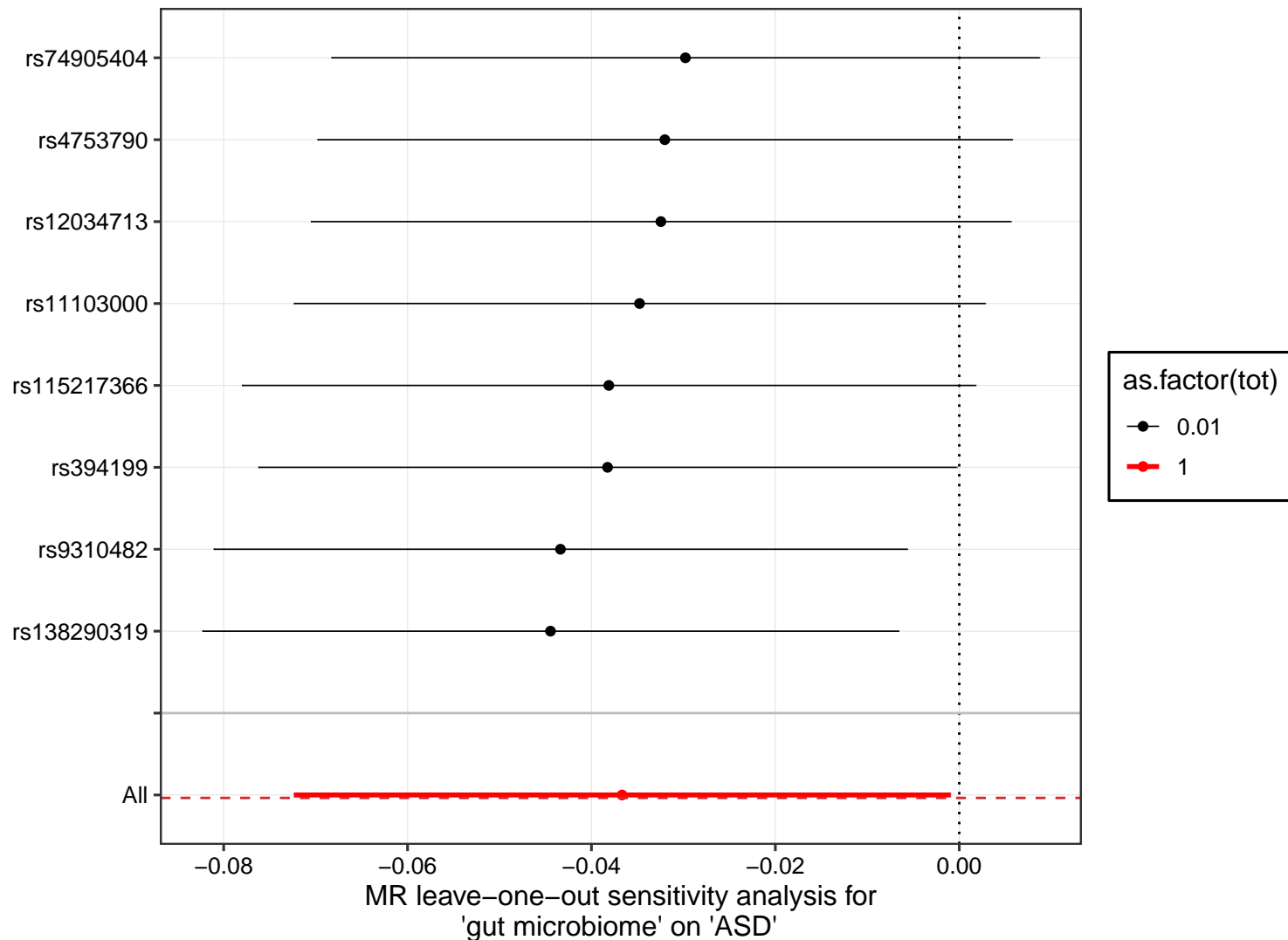

Supplement: Supplementary file 2 — Supplementary Material 2 [file 13568_2025_1969_MOESM2_ESM.zip › Revised supplementary materials/5 Forward MR analysis results/plot/leaveoneout_or_Coprobacter secundus.pdf]

# Leave-One-Out (OR): DTU024 sp002411105

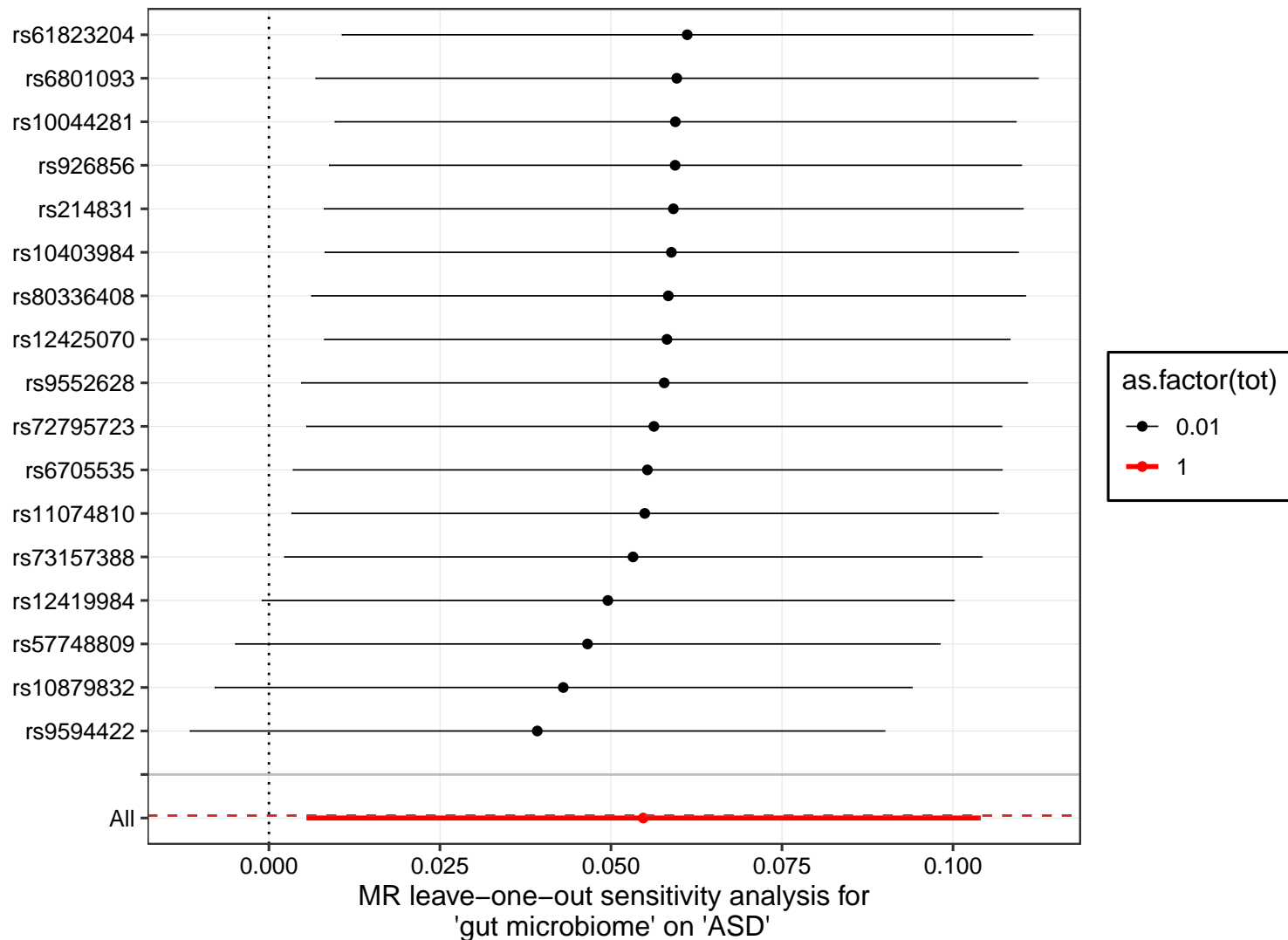

Supplement: Supplementary file 2 — Supplementary Material 2 [file 13568_2025_1969_MOESM2_ESM.zip › Revised supplementary materials/5 Forward MR analysis results/plot/leaveoneout_or_DTU024 sp002411105.pdf]

# Leave-One-Out (OR): Endozoicomonadaceae

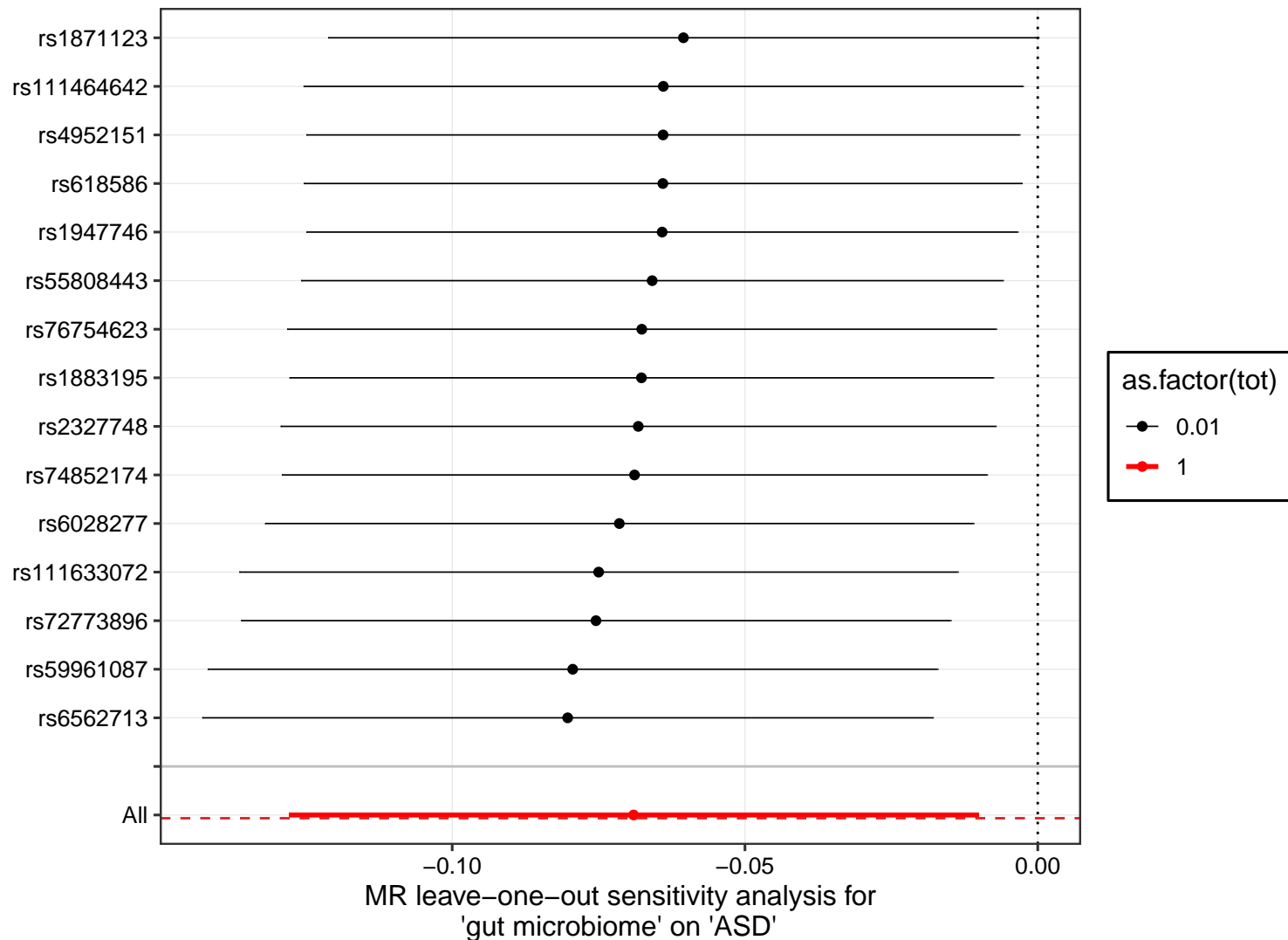

Supplement: Supplementary file 2 — Supplementary Material 2 [file 13568_2025_1969_MOESM2_ESM.zip › Revised supplementary materials/5 Forward MR analysis results/plot/leaveoneout_or_Endozoicomonadaceae.pdf]

# Leave-One-Out (OR): Enorma massiliensis

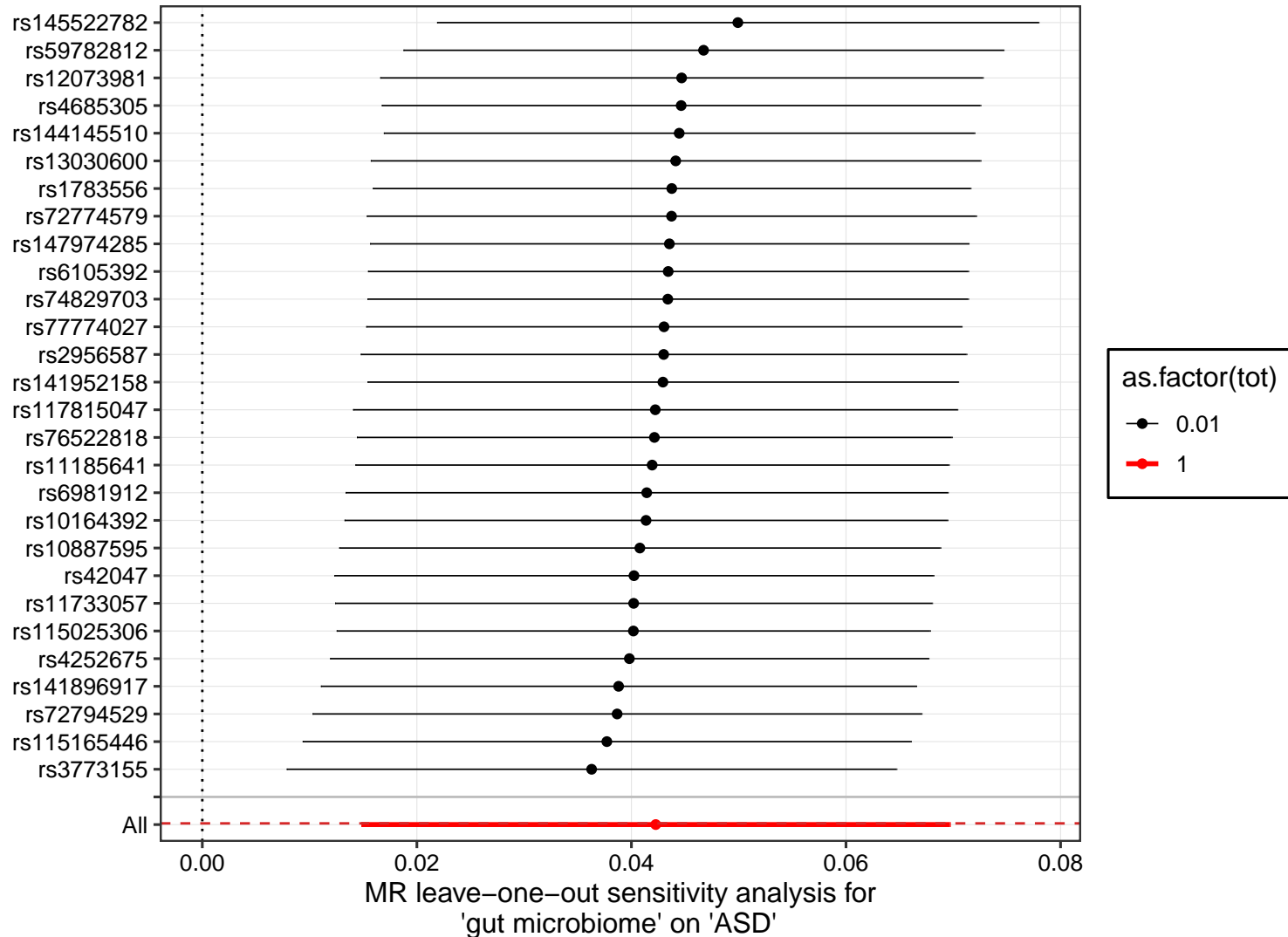

Supplement: Supplementary file 2 — Supplementary Material 2 [file 13568_2025_1969_MOESM2_ESM.zip › Revised supplementary materials/5 Forward MR analysis results/plot/leaveoneout_or_Enorma massiliensis.pdf]

# Leave-One-Out (OR): Enterococcus faecalis

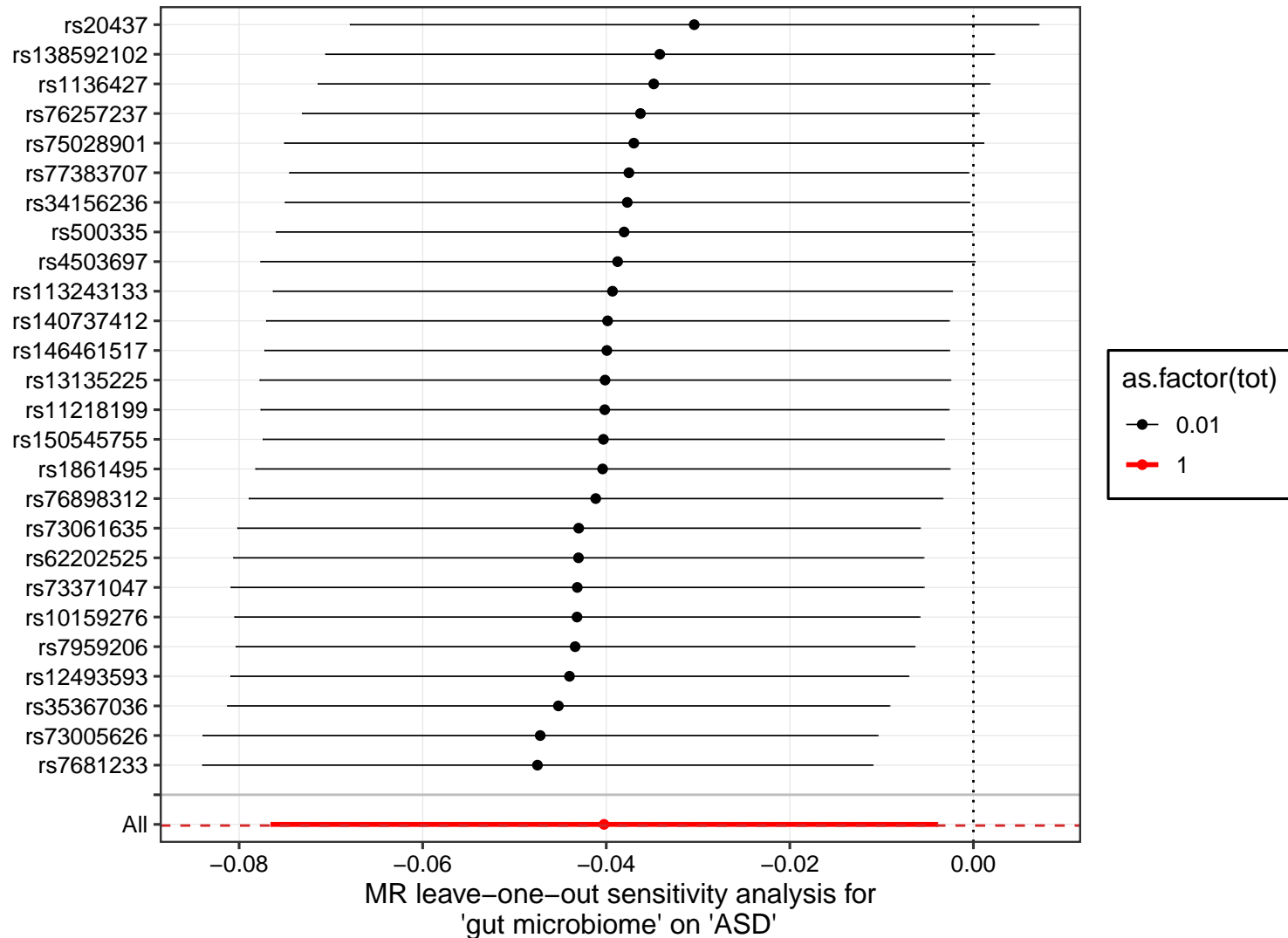

Supplement: Supplementary file 2 — Supplementary Material 2 [file 13568_2025_1969_MOESM2_ESM.zip › Revised supplementary materials/5 Forward MR analysis results/plot/leaveoneout_or_Enterococcus faecalis.pdf]

# Leave-One-Out (OR): *Eubacterium callanderi*

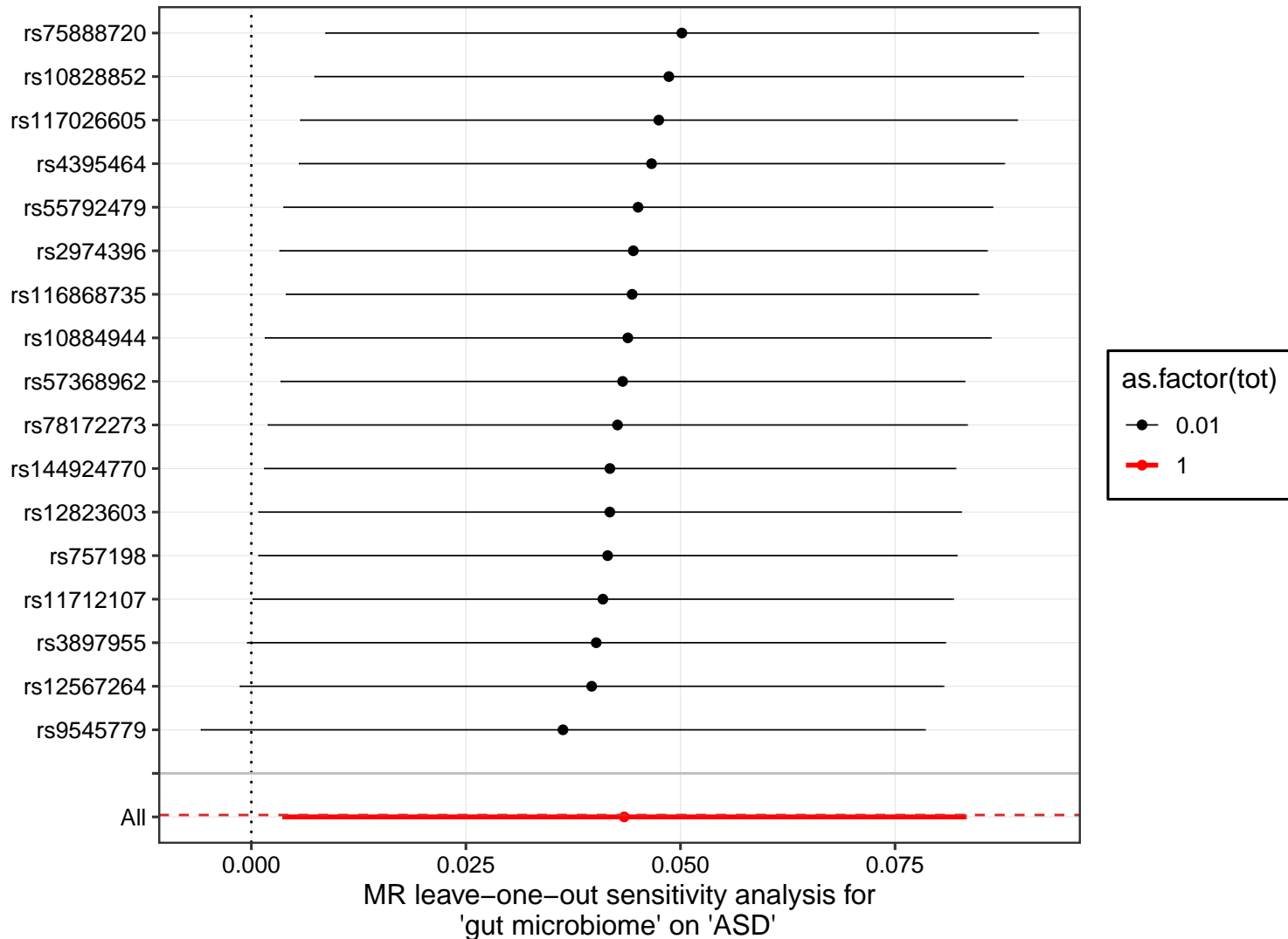

Supplement: Supplementary file 2 — Supplementary Material 2 [file 13568_2025_1969_MOESM2_ESM.zip › Revised supplementary materials/5 Forward MR analysis results/plot/leaveoneout_or_Eubacterium callanderi.pdf]

# Leave-One-Out (OR): *Faecalicatena torques*

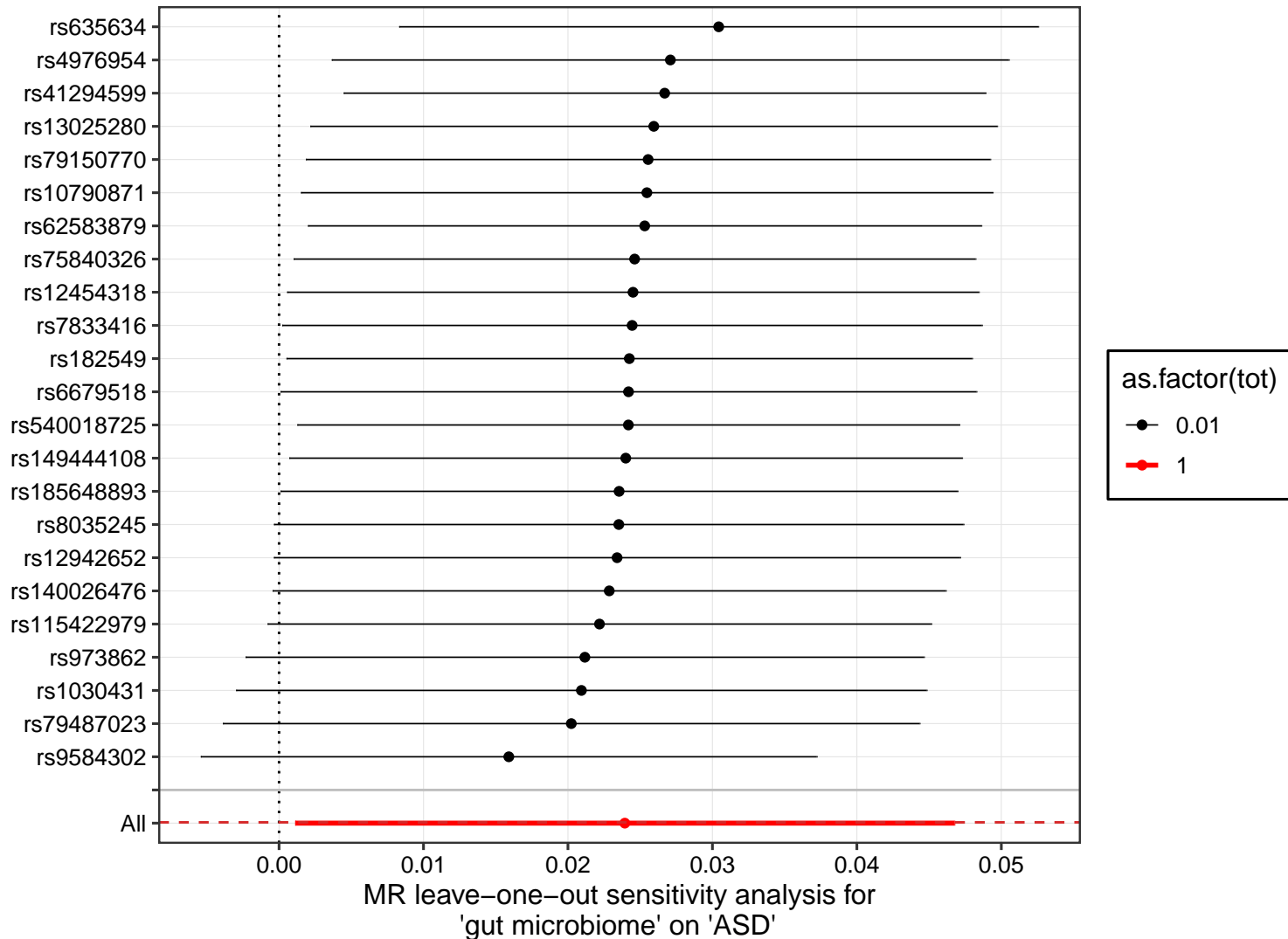

Supplement: Supplementary file 2 — Supplementary Material 2 [file 13568_2025_1969_MOESM2_ESM.zip › Revised supplementary materials/5 Forward MR analysis results/plot/leaveoneout_or_Faecalicatena torques.pdf]

# Leave-One-Out (OR): Fibrobacteria

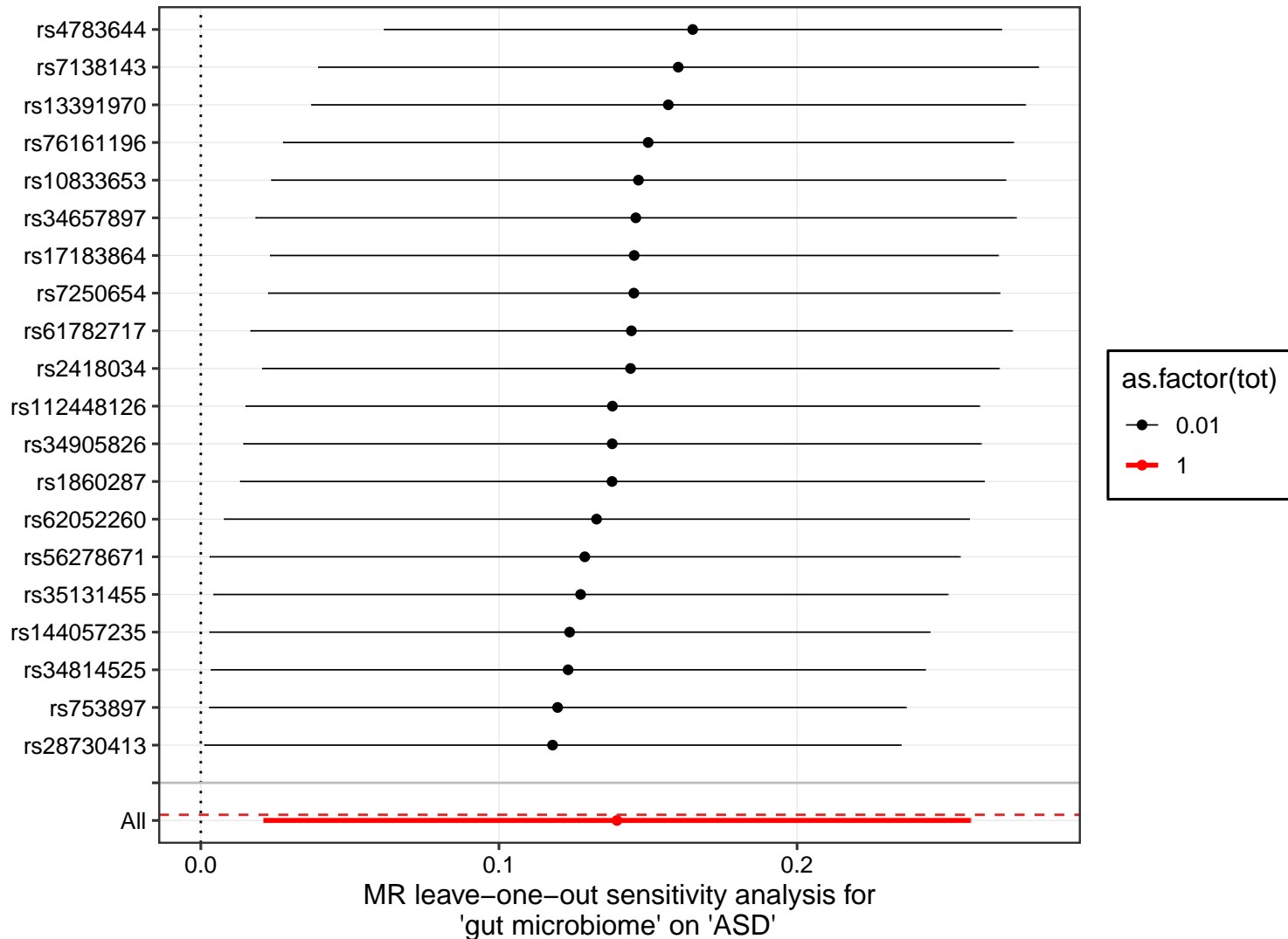

Supplement: Supplementary file 2 — Supplementary Material 2 [file 13568_2025_1969_MOESM2_ESM.zip › Revised supplementary materials/5 Forward MR analysis results/plot/leaveoneout_or_Fibrobacteria.pdf]

# Leave-One-Out (OR): Francisellaceae

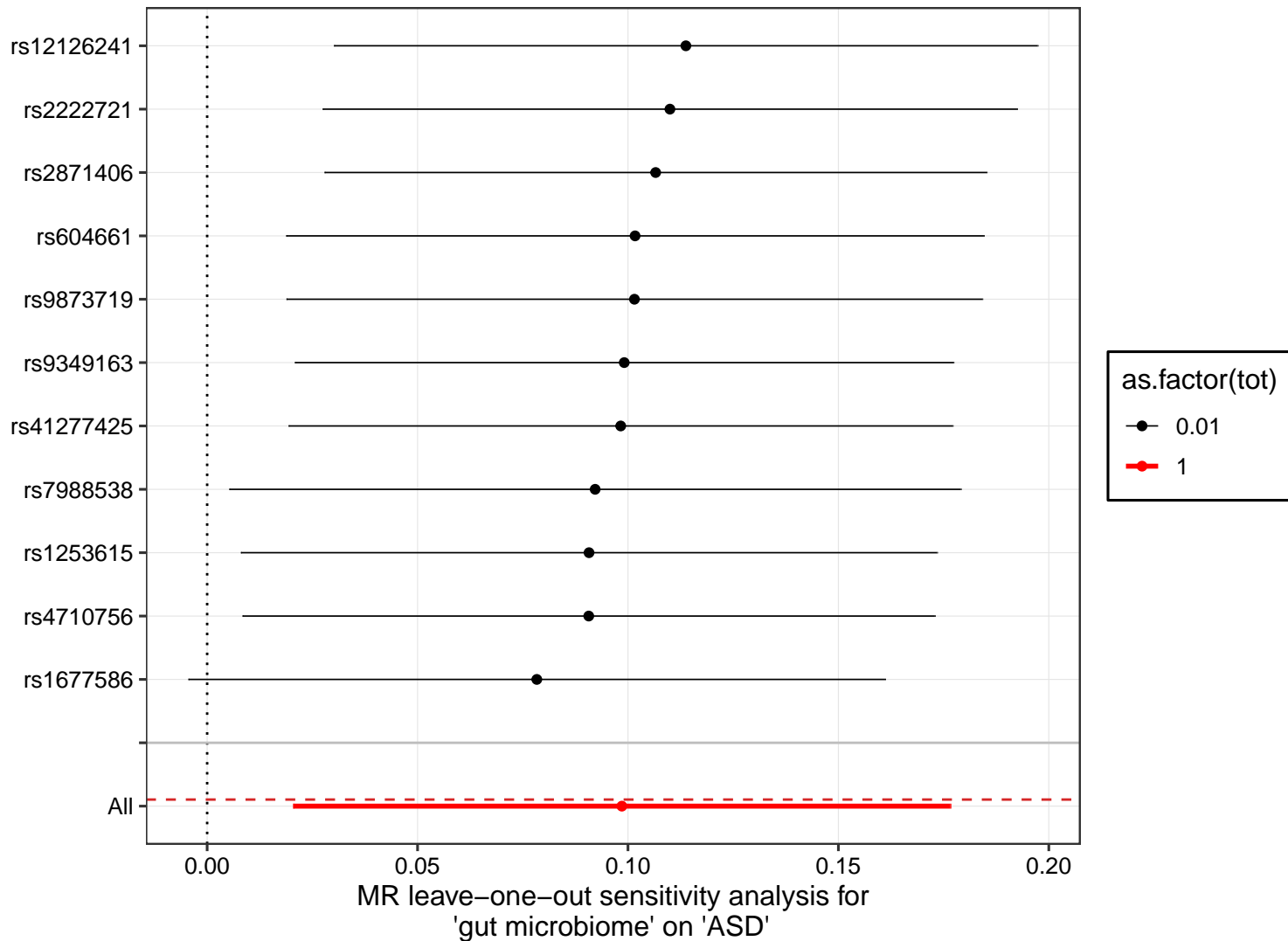

Supplement: Supplementary file 2 — Supplementary Material 2 [file 13568_2025_1969_MOESM2_ESM.zip › Revised supplementary materials/5 Forward MR analysis results/plot/leaveoneout_or_Francisellaceae.pdf]

# Leave-One-Out (OR): Geminocystis

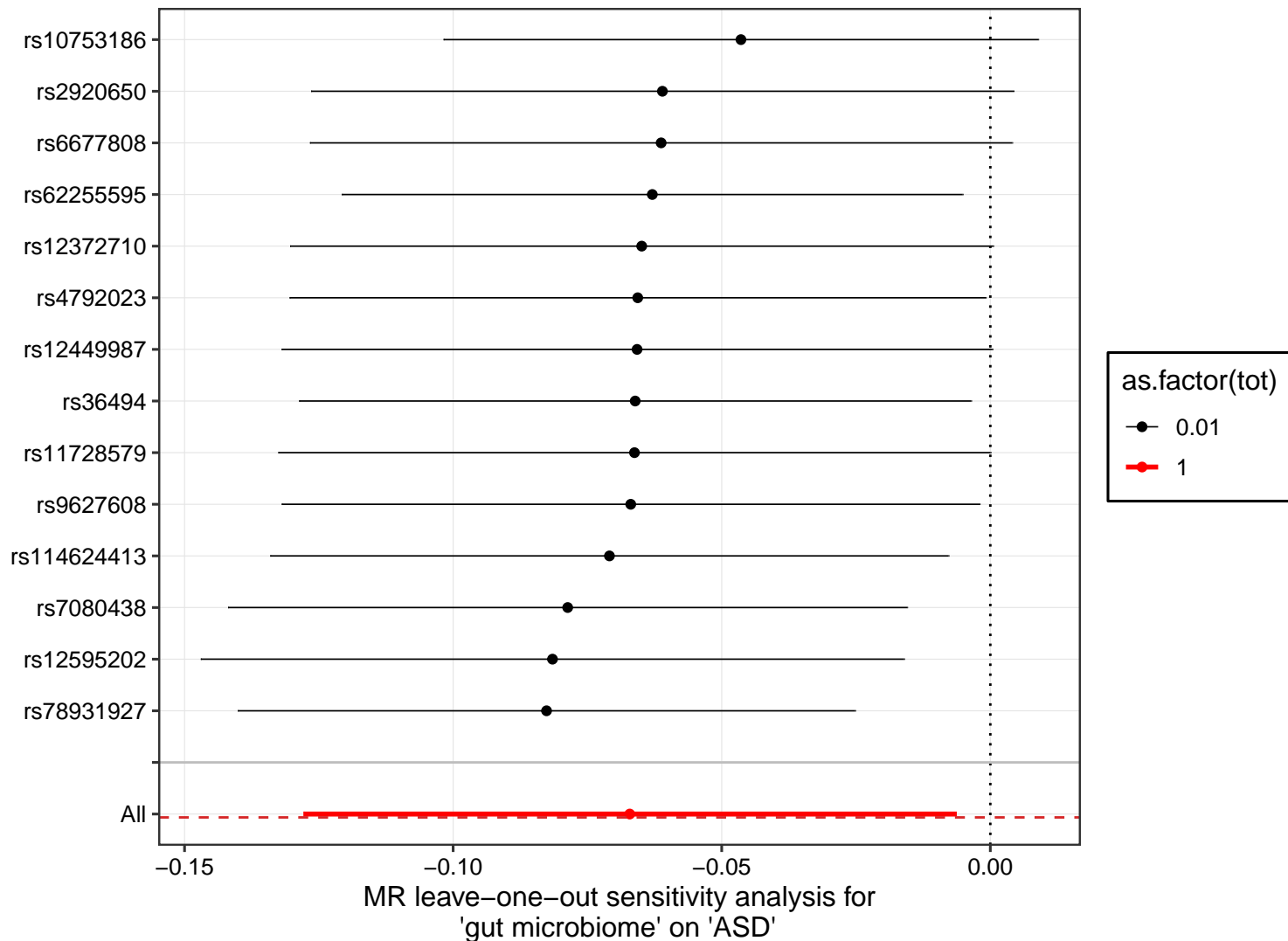

Supplement: Supplementary file 2 — Supplementary Material 2 [file 13568_2025_1969_MOESM2_ESM.zip › Revised supplementary materials/5 Forward MR analysis results/plot/leaveoneout_or_Geminocystis.pdf]

# Leave-One-Out (OR): koll11

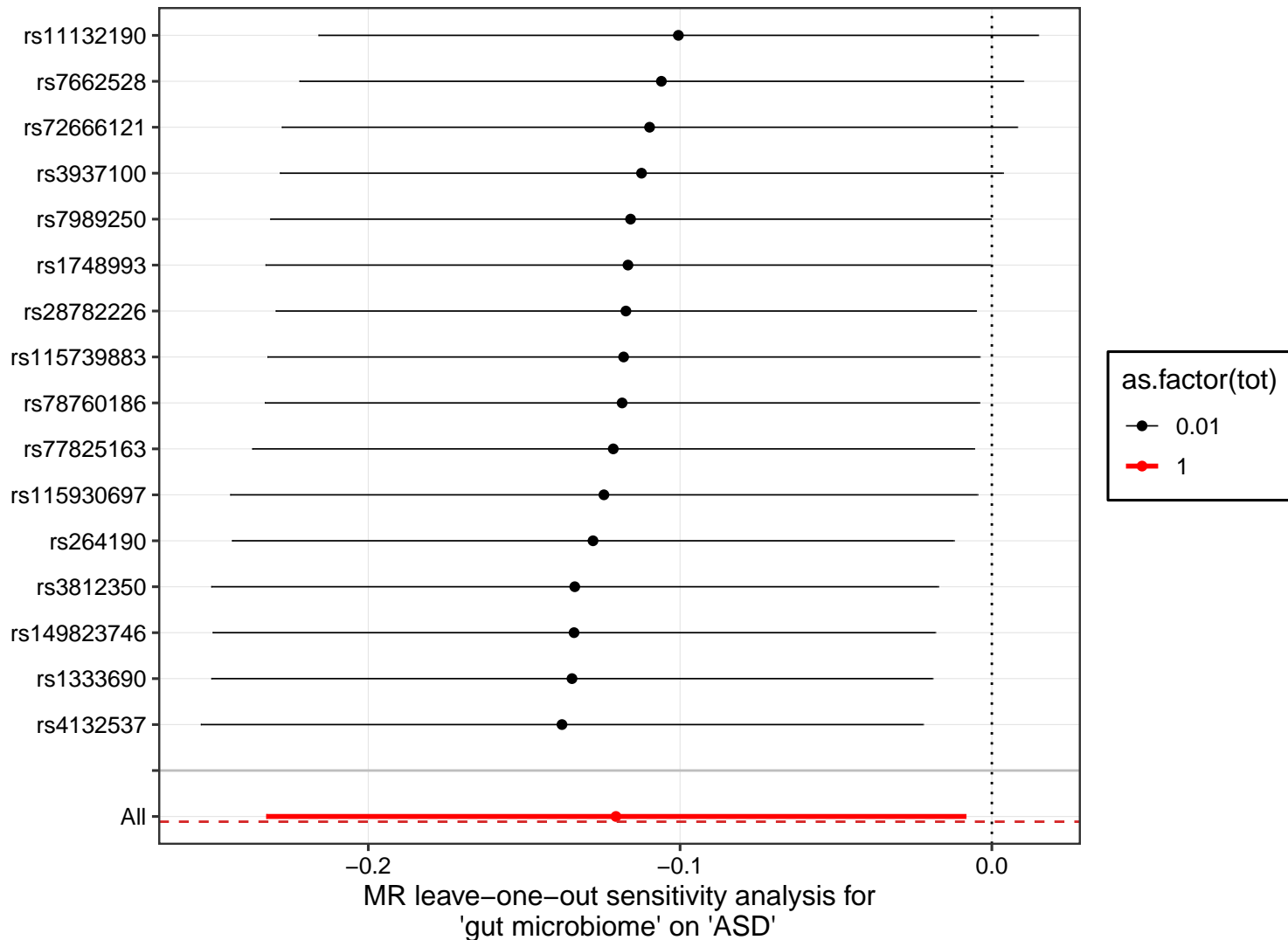

Supplement: Supplementary file 2 — Supplementary Material 2 [file 13568_2025_1969_MOESM2_ESM.zip › Revised supplementary materials/5 Forward MR analysis results/plot/leaveoneout_or_koll11.pdf]

# Leave-One-Out (OR): *Lachnospira rogosae*

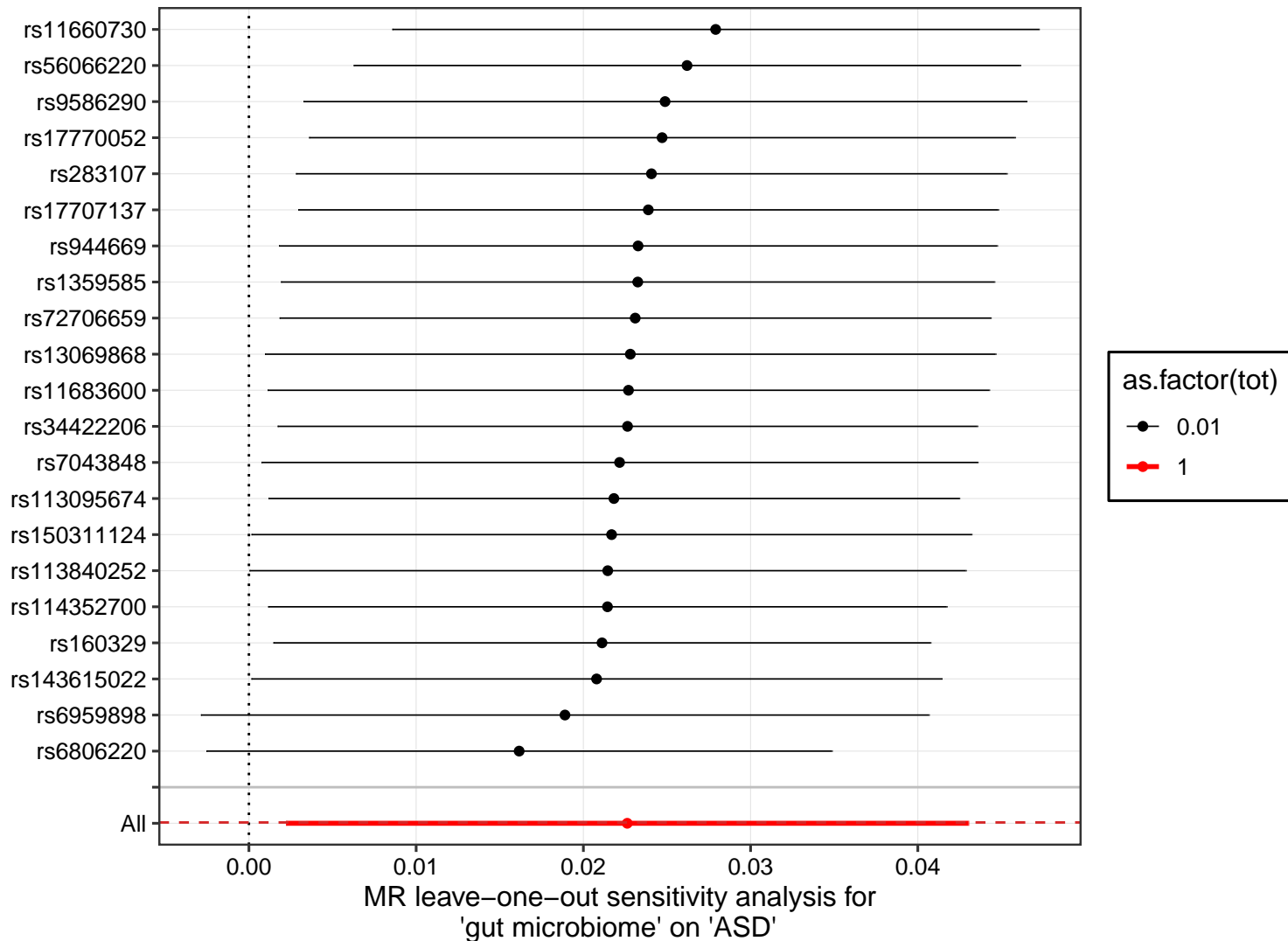

Supplement: Supplementary file 2 — Supplementary Material 2 [file 13568_2025_1969_MOESM2_ESM.zip › Revised supplementary materials/5 Forward MR analysis results/plot/leaveoneout_or_Lachnospira rogosae.pdf]

## Leave-One-Out (OR): Olsenella C

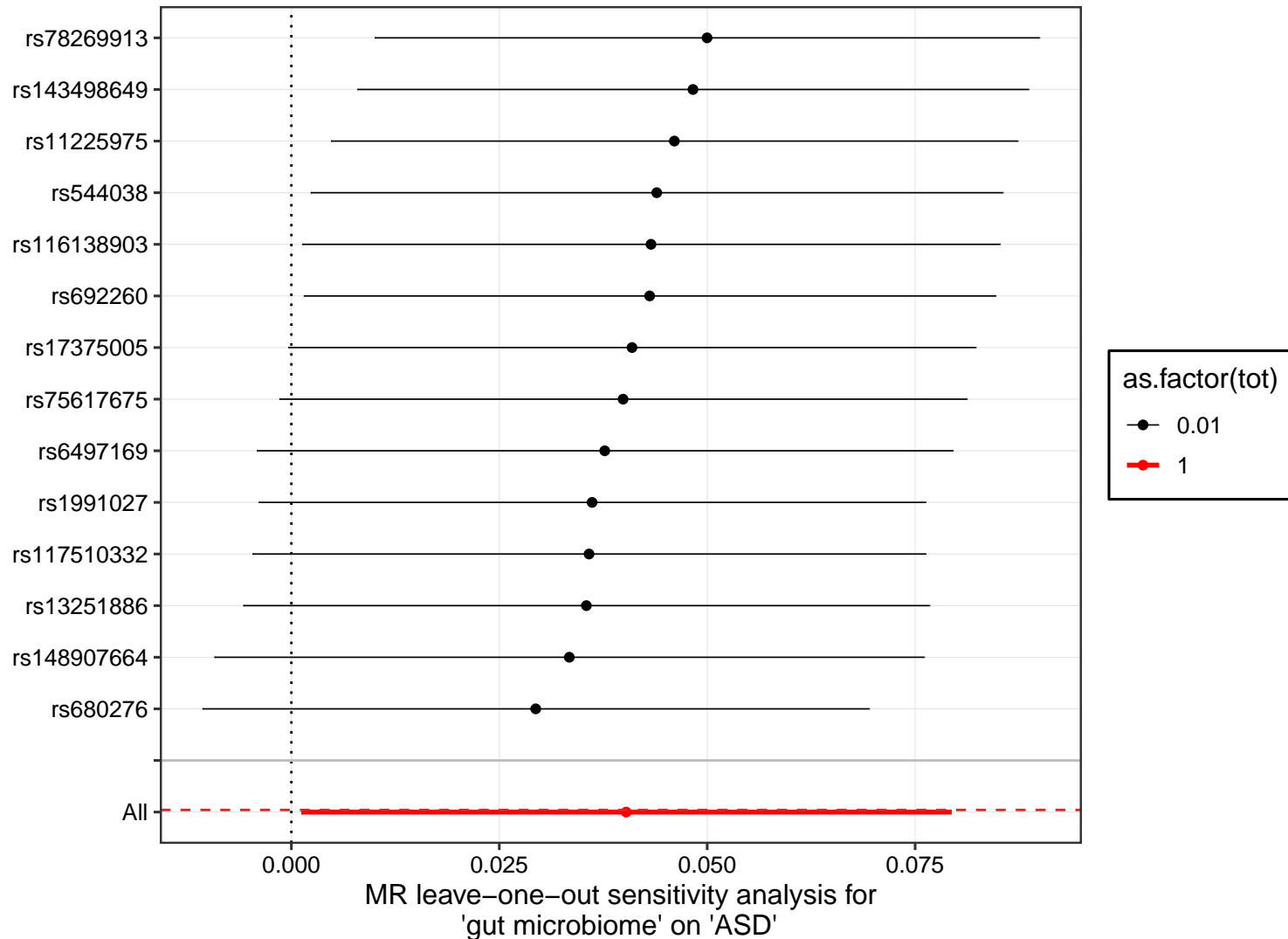

Supplement: Supplementary file 2 — Supplementary Material 2 [file 13568_2025_1969_MOESM2_ESM.zip › Revised supplementary materials/5 Forward MR analysis results/plot/leaveoneout_or_Olsenella C.pdf]

# Leave-One-Out (OR): Parabacteroides

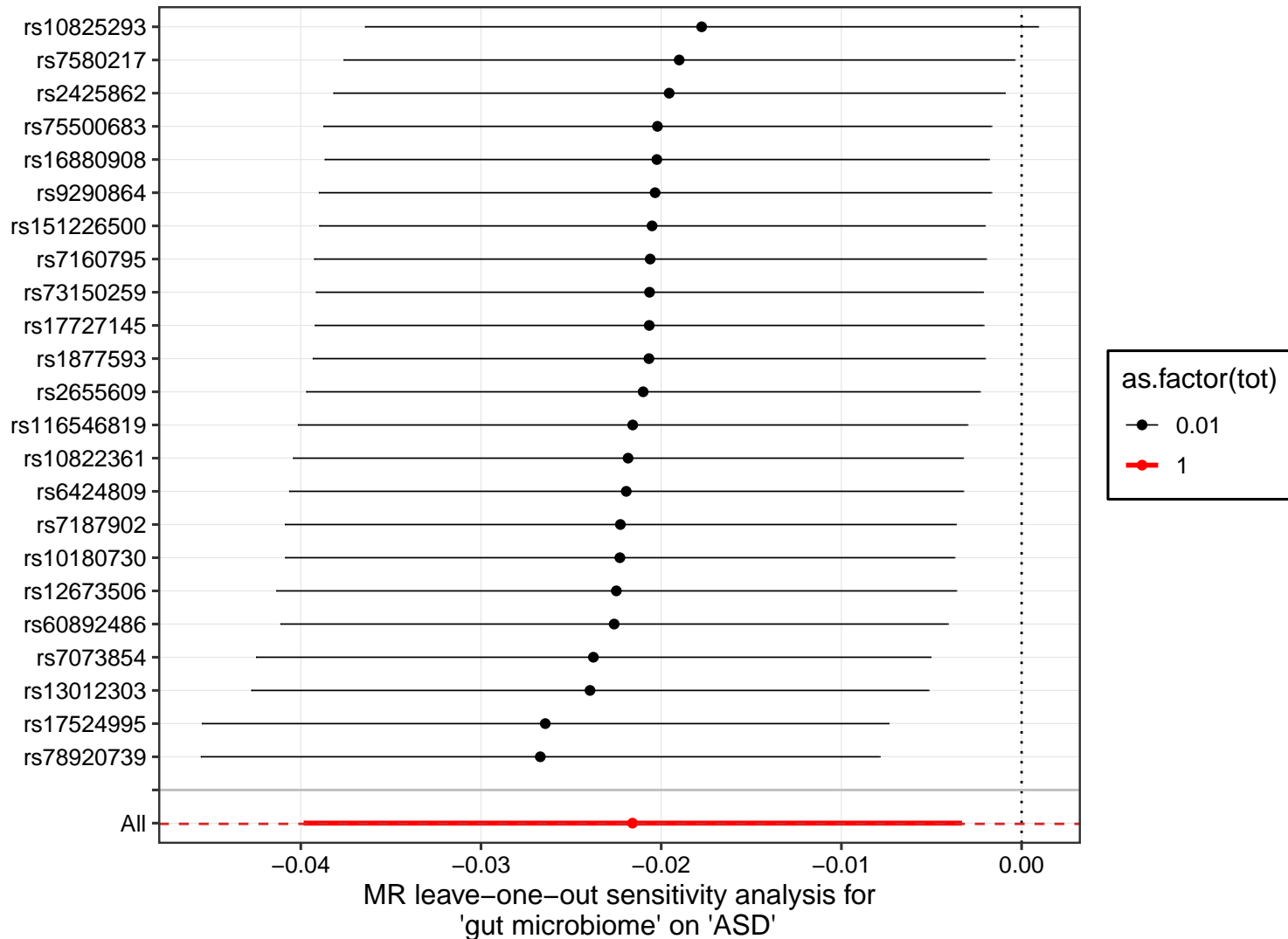

Supplement: Supplementary file 2 — Supplementary Material 2 [file 13568_2025_1969_MOESM2_ESM.zip › Revised supplementary materials/5 Forward MR analysis results/plot/leaveoneout_or_Parabacteroides.pdf]

# Leave-One-Out (OR): Prevotella sp002933775

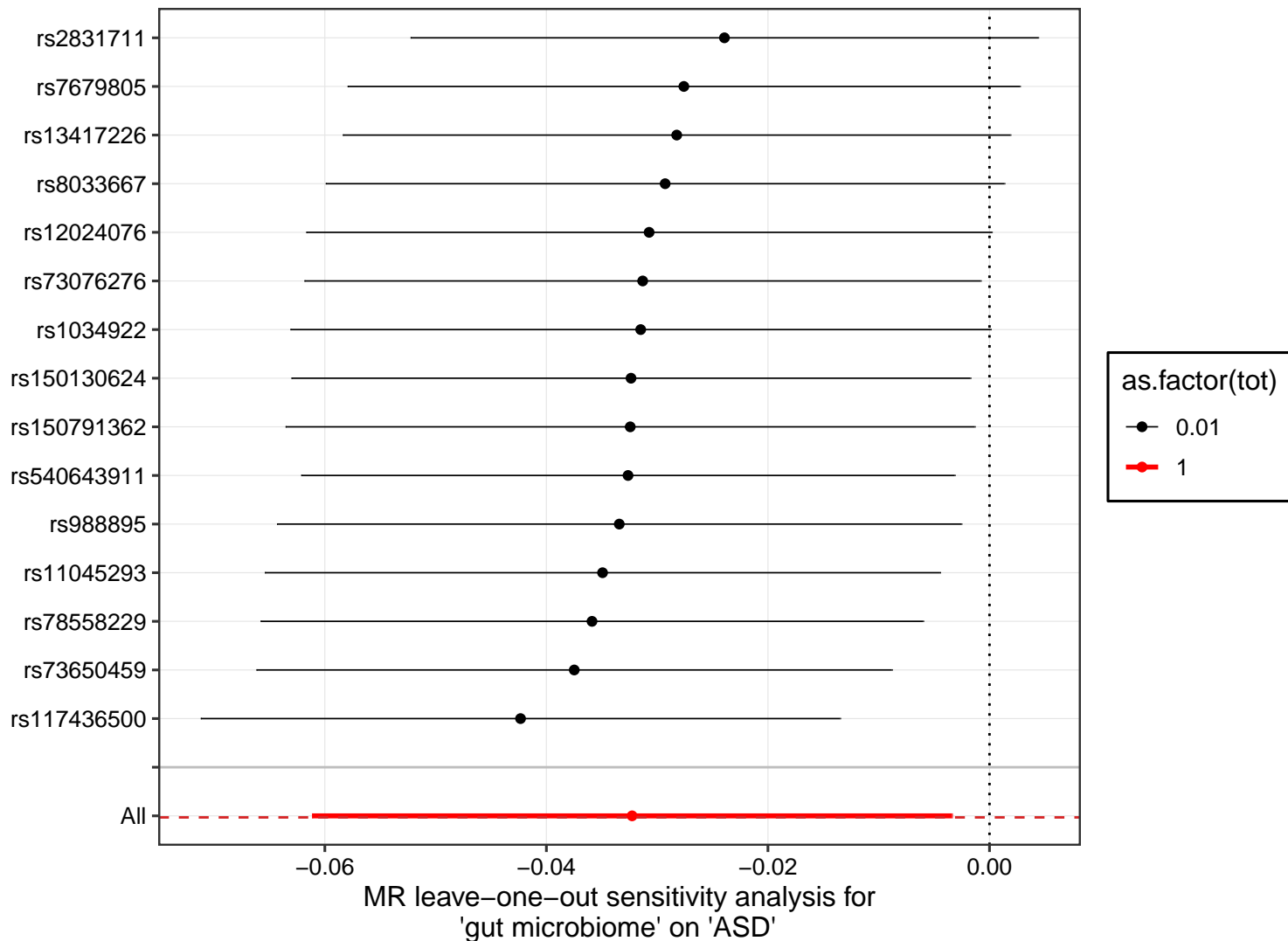

Supplement: Supplementary file 2 — Supplementary Material 2 [file 13568_2025_1969_MOESM2_ESM.zip › Revised supplementary materials/5 Forward MR analysis results/plot/leaveoneout_or_Prevotella sp002933775.pdf]

# Leave-One-Out (OR): UBA1066

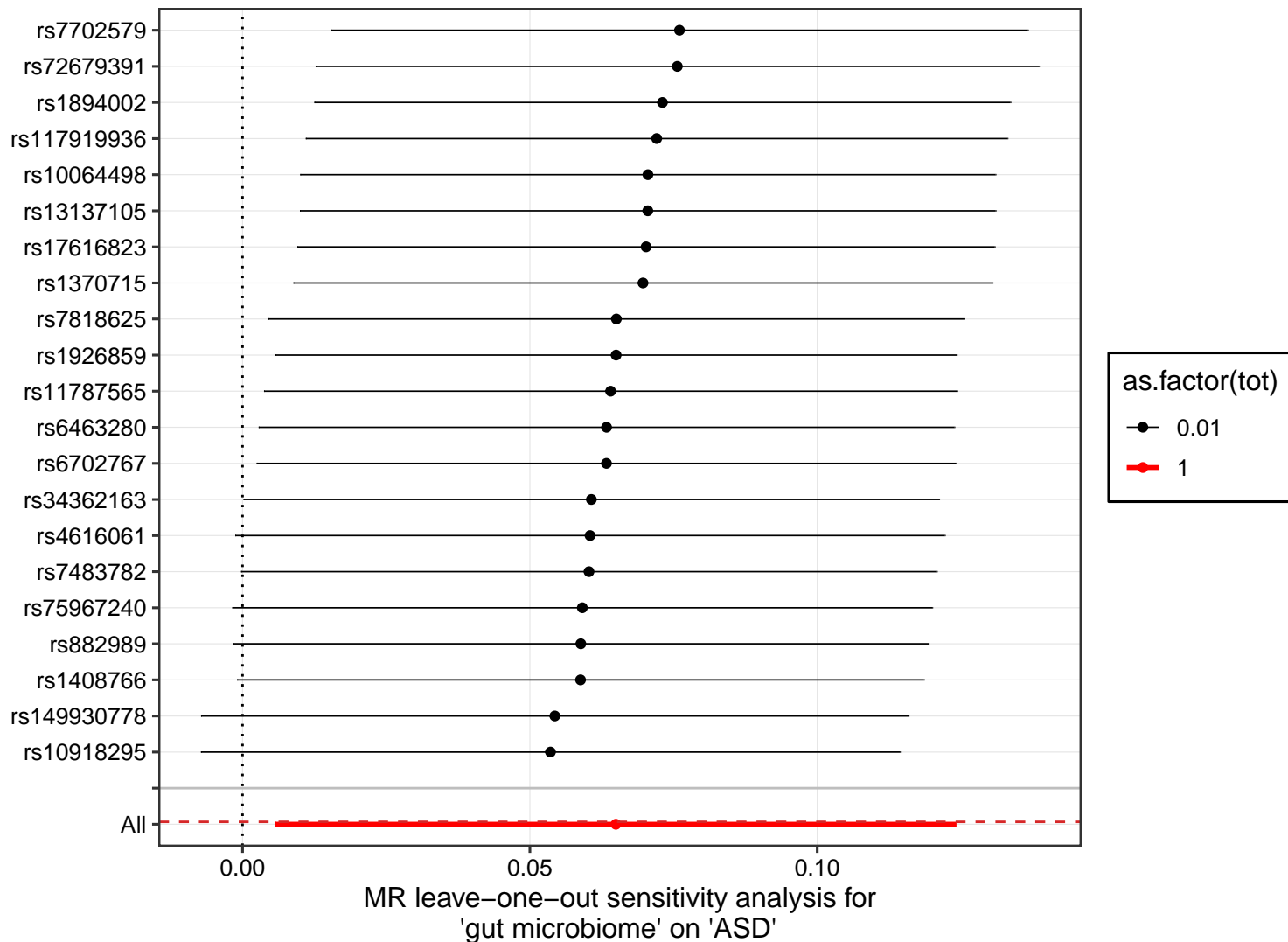

Supplement: Supplementary file 2 — Supplementary Material 2 [file 13568_2025_1969_MOESM2_ESM.zip › Revised supplementary materials/5 Forward MR analysis results/plot/leaveoneout_or_UBA1066.pdf]

# Leave-One-Out (OR): UBA7703

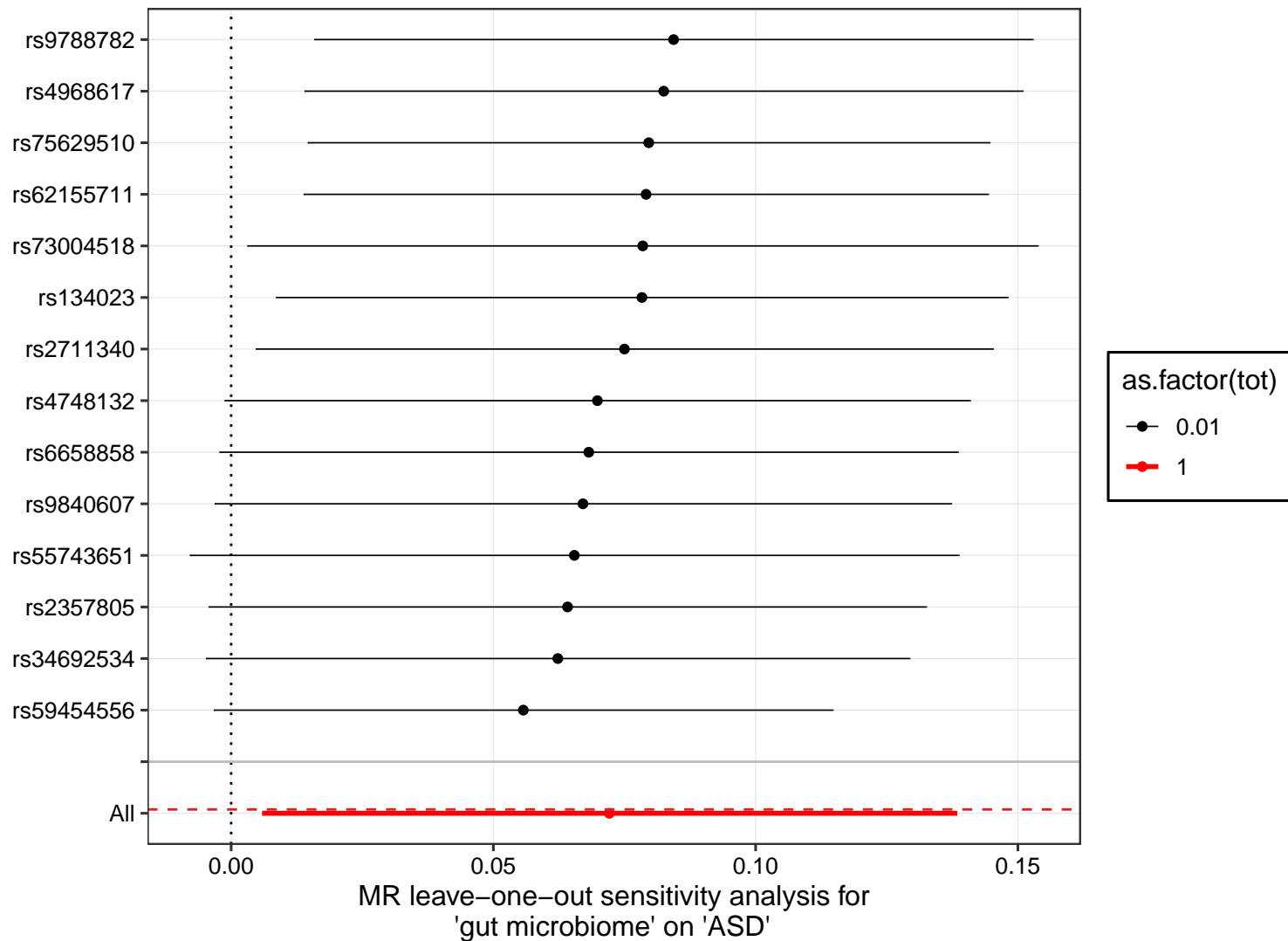

Supplement: Supplementary file 2 — Supplementary Material 2 [file 13568_2025_1969_MOESM2_ESM.zip › Revised supplementary materials/5 Forward MR analysis results/plot/leaveoneout_or_UBA7703.pdf]

# Leave-One-Out (OR): UBA8904

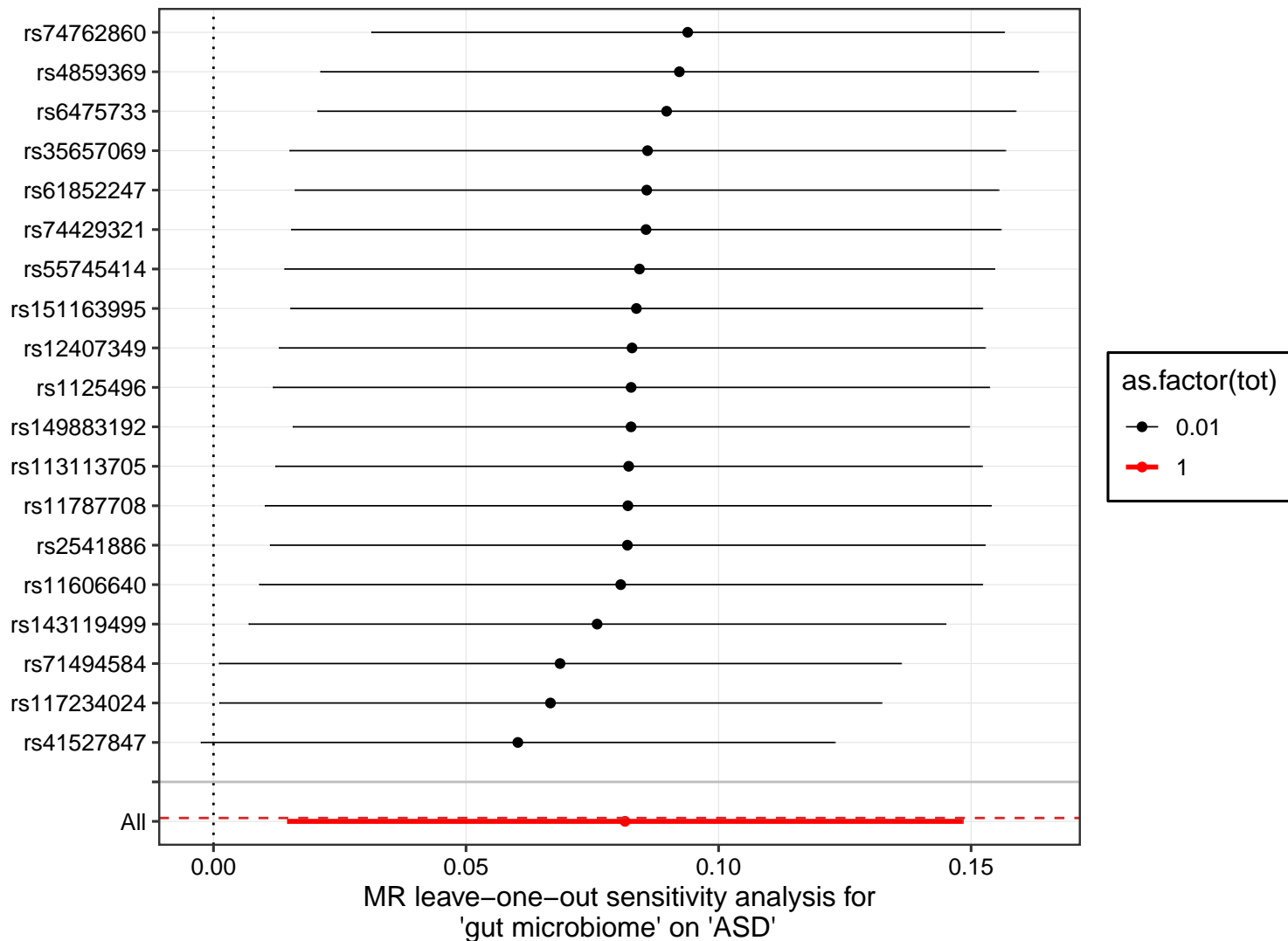

Supplement: Supplementary file 2 — Supplementary Material 2 [file 13568_2025_1969_MOESM2_ESM.zip › Revised supplementary materials/5 Forward MR analysis results/plot/leaveoneout_or_UBA8904.pdf]

# Leave-One-Out (OR): V9D3004

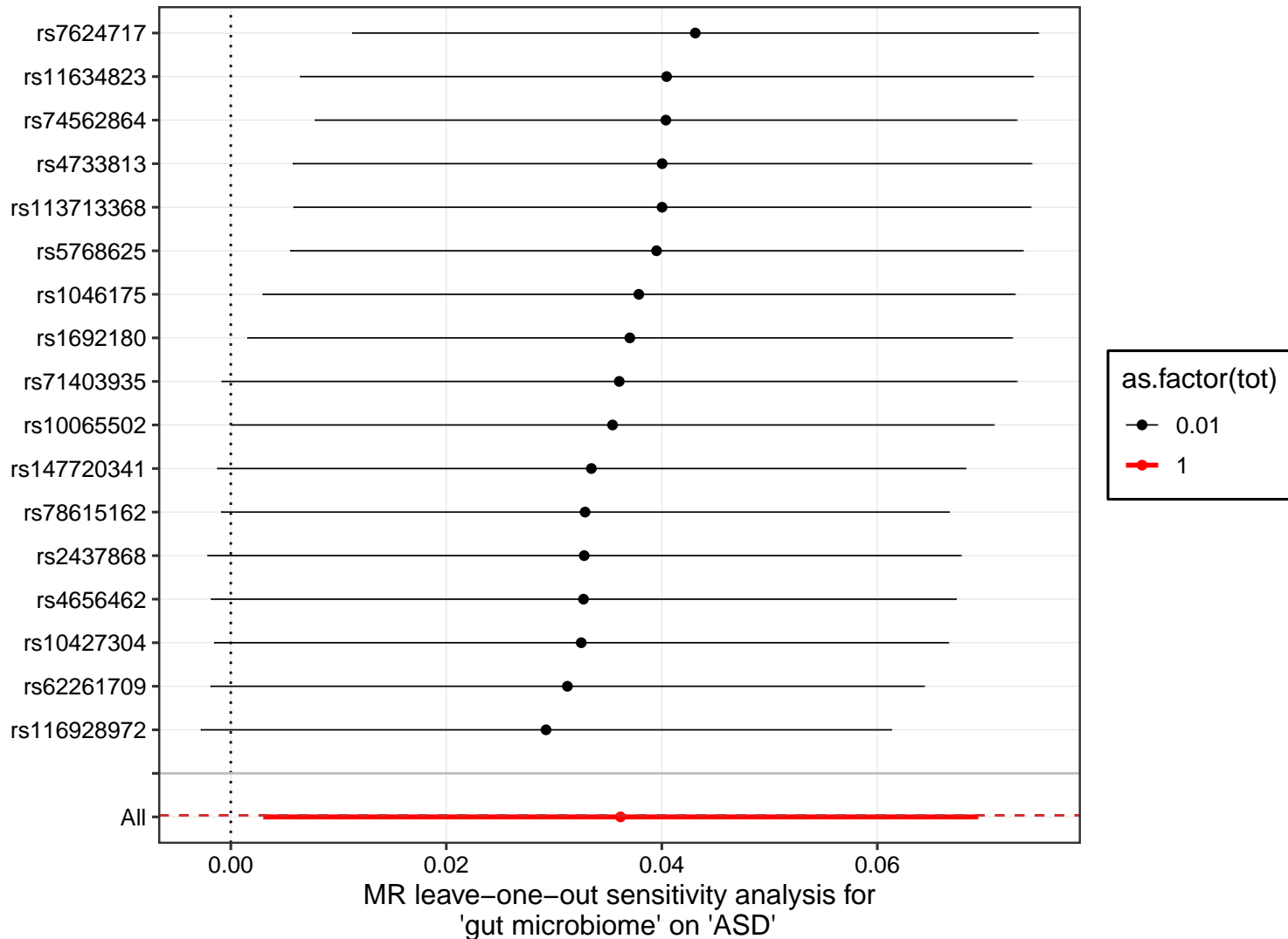

Supplement: Supplementary file 2 — Supplementary Material 2 [file 13568_2025_1969_MOESM2_ESM.zip › Revised supplementary materials/5 Forward MR analysis results/plot/leaveoneout_or_V9D3004.pdf]

Scatter Plot (OR): Acidaminococcus fermentans

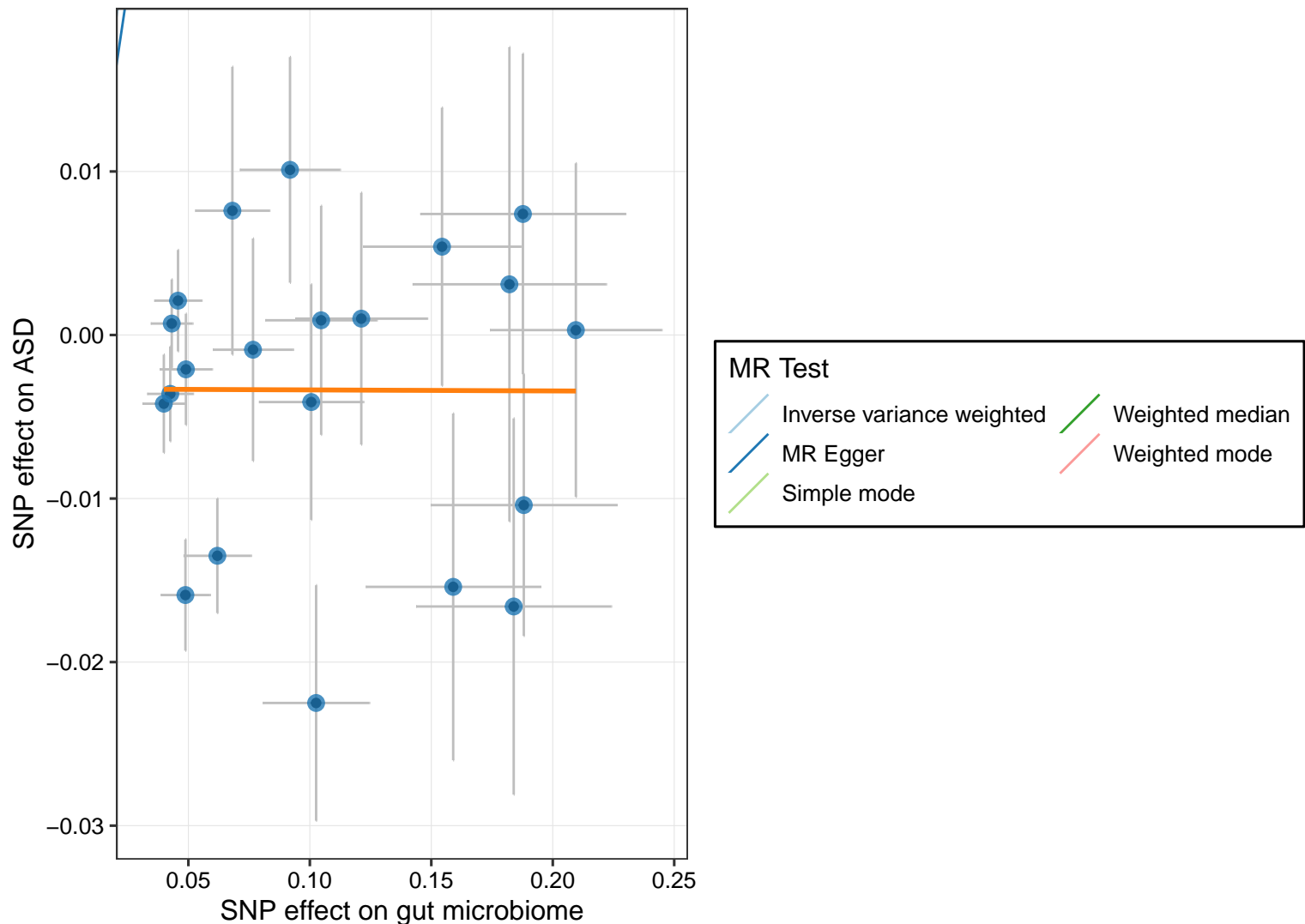

Supplement: Supplementary file 2 — Supplementary Material 2 [file 13568_2025_1969_MOESM2_ESM.zip › Revised supplementary materials/5 Forward MR analysis results/plot/scatter_or_Acidaminococcus fermentans.pdf]

**Scatter Plot (OR): CAG-475**

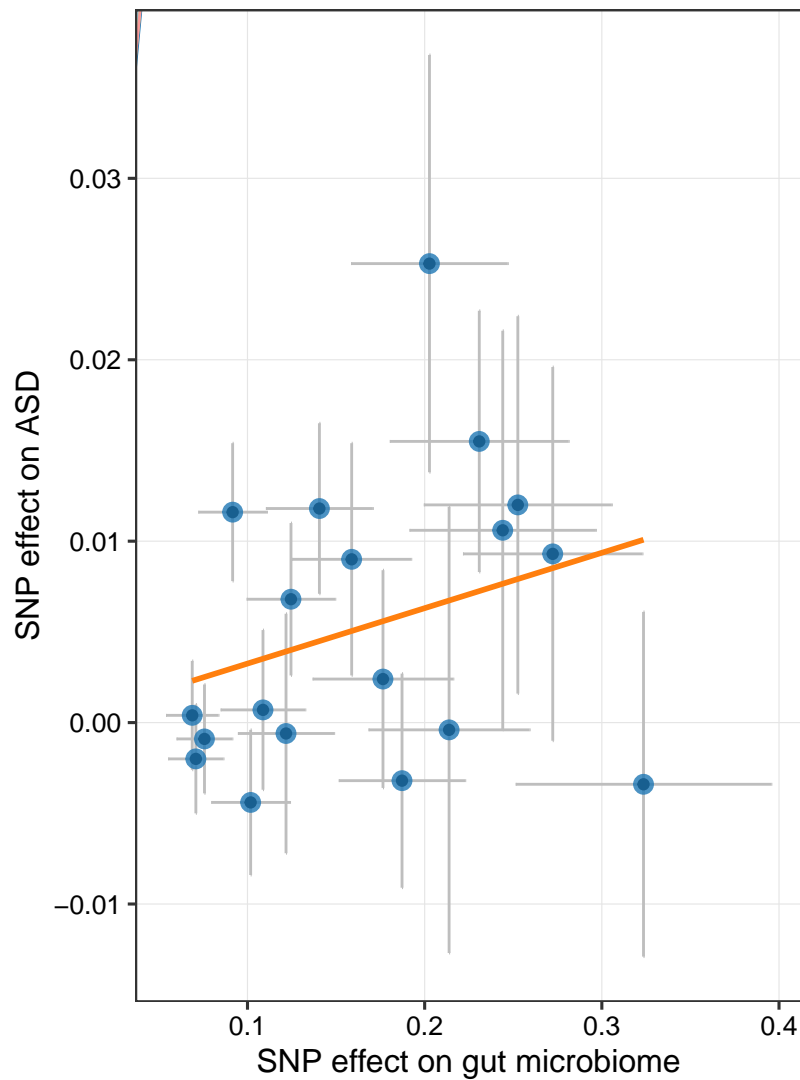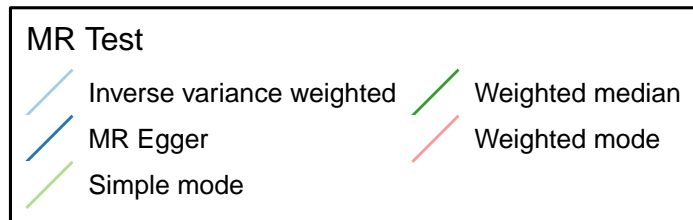

Supplement: Supplementary file 2 — Supplementary Material 2 [file 13568_2025_1969_MOESM2_ESM.zip › Revised supplementary materials/5 Forward MR analysis results/plot/scatter_or_CAG-475.pdf]

Scatter Plot (OR): CAG-510 sp002432425

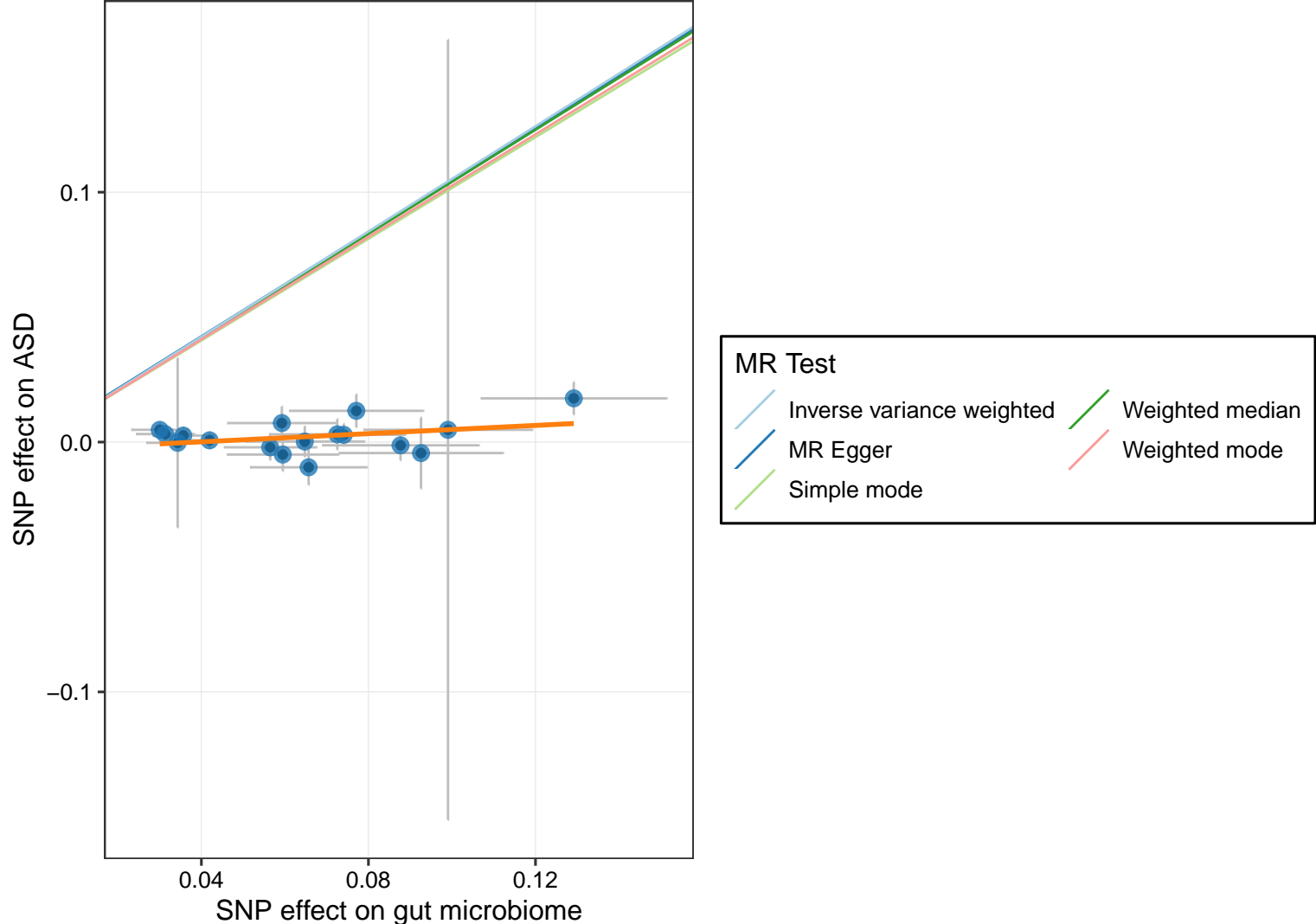

Supplement: Supplementary file 2 — Supplementary Material 2 [file 13568_2025_1969_MOESM2_ESM.zip › Revised supplementary materials/5 Forward MR analysis results/plot/scatter_or_CAG-510 sp002432425.pdf]

**Scatter Plot (OR): CAG-884**

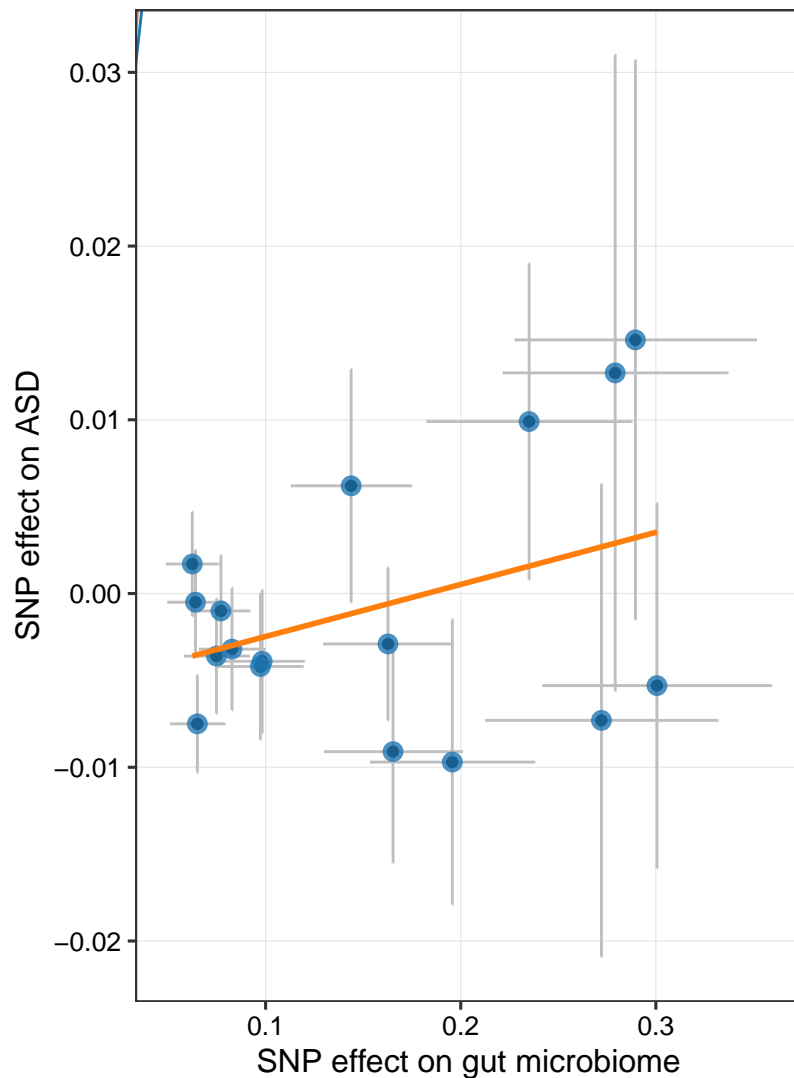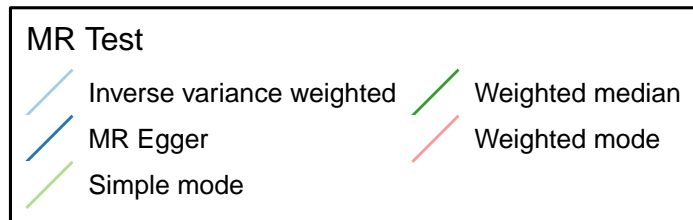

Supplement: Supplementary file 2 — Supplementary Material 2 [file 13568_2025_1969_MOESM2_ESM.zip › Revised supplementary materials/5 Forward MR analysis results/plot/scatter_or_CAG-884.pdf]

**Scatter Plot (OR): Coprobacillus**

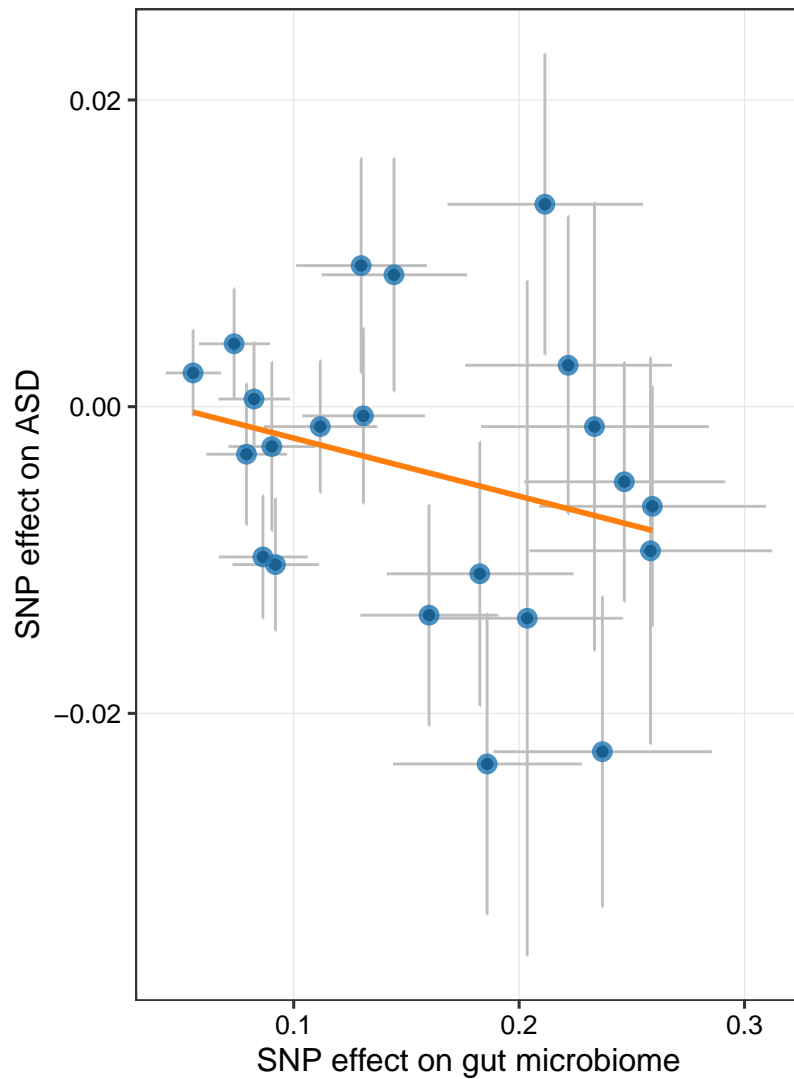

Supplement: Supplementary file 2 — Supplementary Material 2 [file 13568_2025_1969_MOESM2_ESM.zip › Revised supplementary materials/5 Forward MR analysis results/plot/scatter_or_Coprobacillus.pdf]

## Scatter Plot (OR): *Coprobacter secundus*

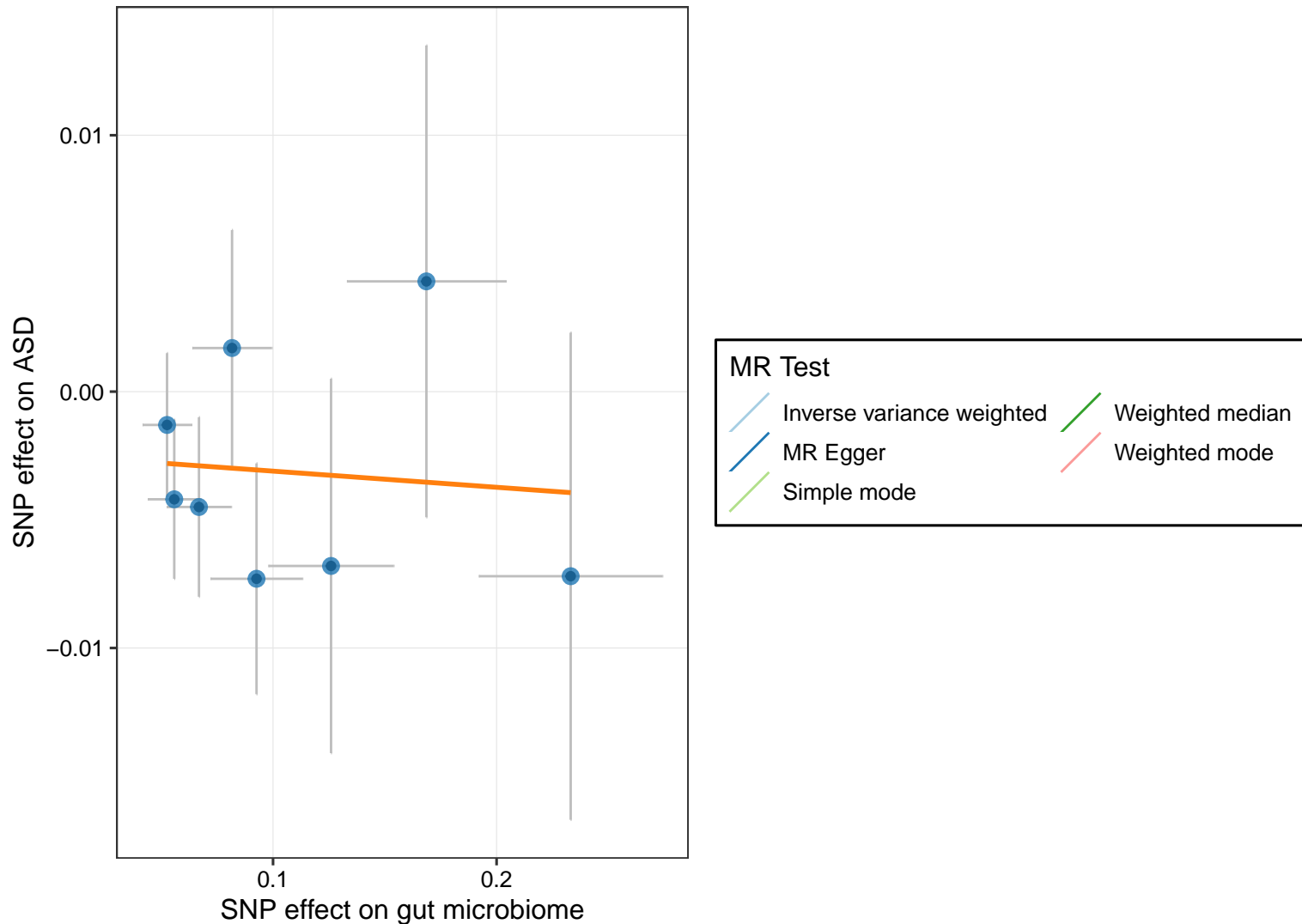

Supplement: Supplementary file 2 — Supplementary Material 2 [file 13568_2025_1969_MOESM2_ESM.zip › Revised supplementary materials/5 Forward MR analysis results/plot/scatter_or_Coprobacter secundus.pdf]

**Scatter Plot (OR): DTU024 sp002411105**

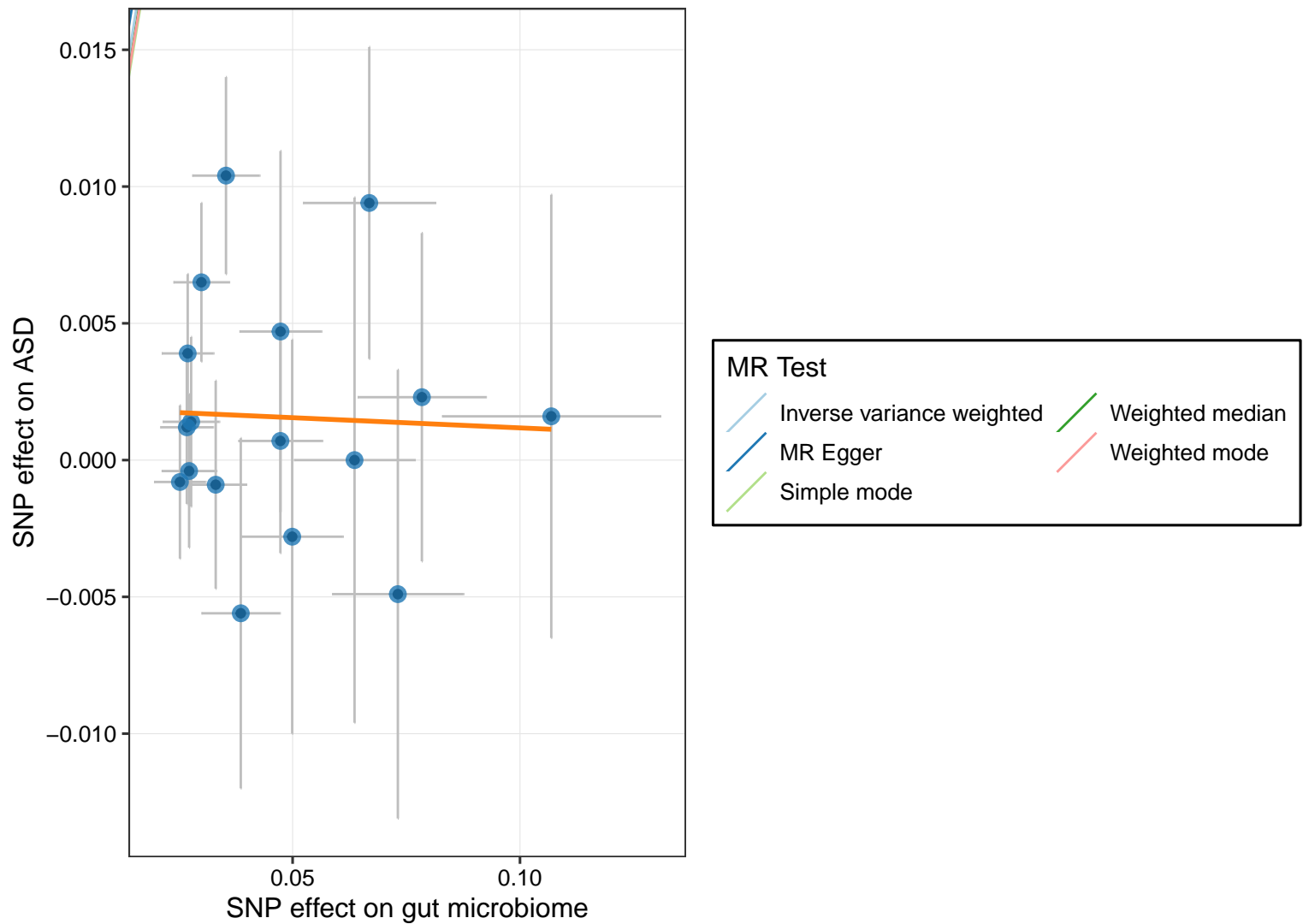

Supplement: Supplementary file 2 — Supplementary Material 2 [file 13568_2025_1969_MOESM2_ESM.zip › Revised supplementary materials/5 Forward MR analysis results/plot/scatter_or_DTU024 sp002411105.pdf]

**Scatter Plot (OR): Endozoicomonadaceae**

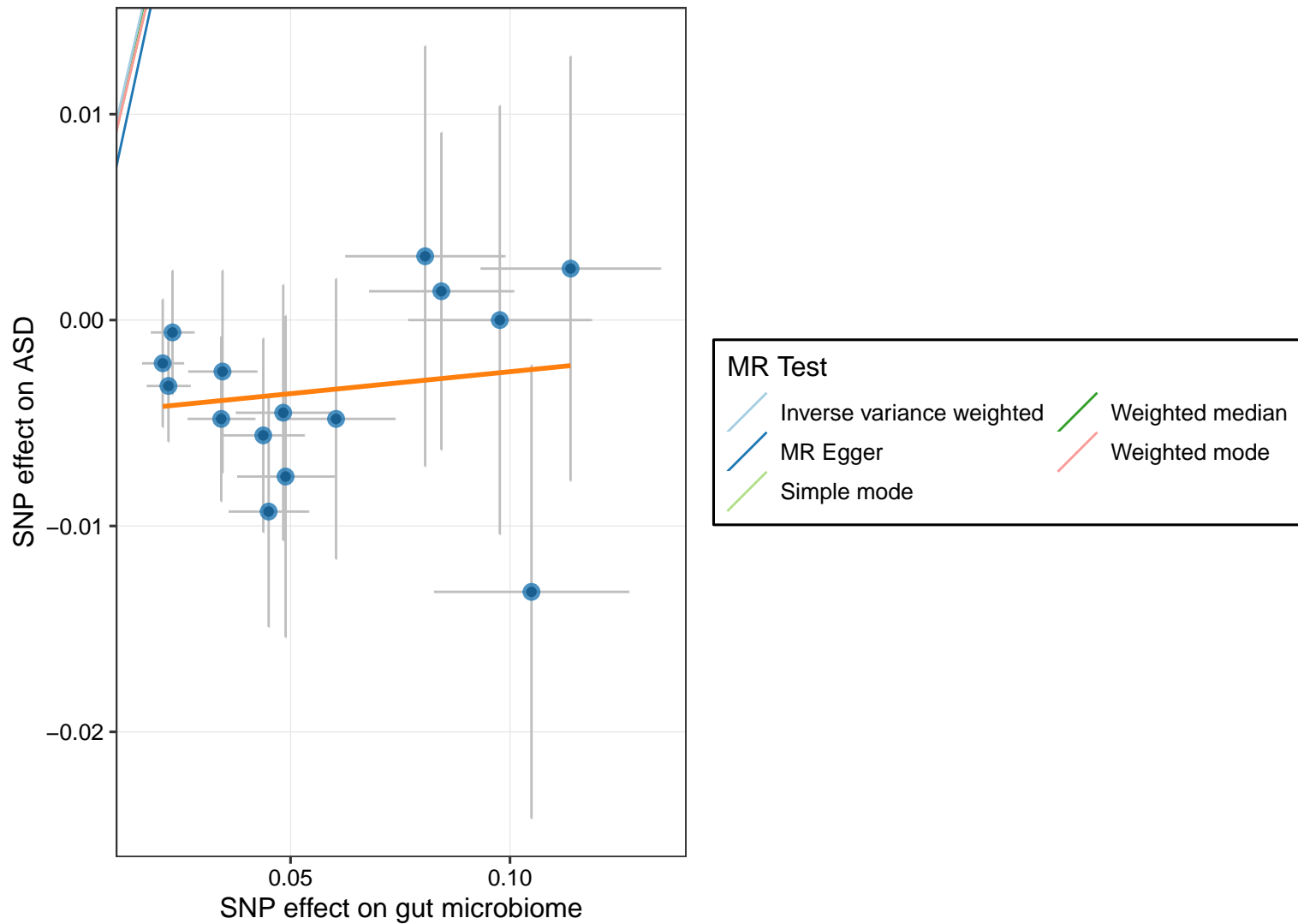

Supplement: Supplementary file 2 — Supplementary Material 2 [file 13568_2025_1969_MOESM2_ESM.zip › Revised supplementary materials/5 Forward MR analysis results/plot/scatter_or_Endozoicomonadaceae.pdf]

**Scatter Plot (OR): Enorma massiliensis**

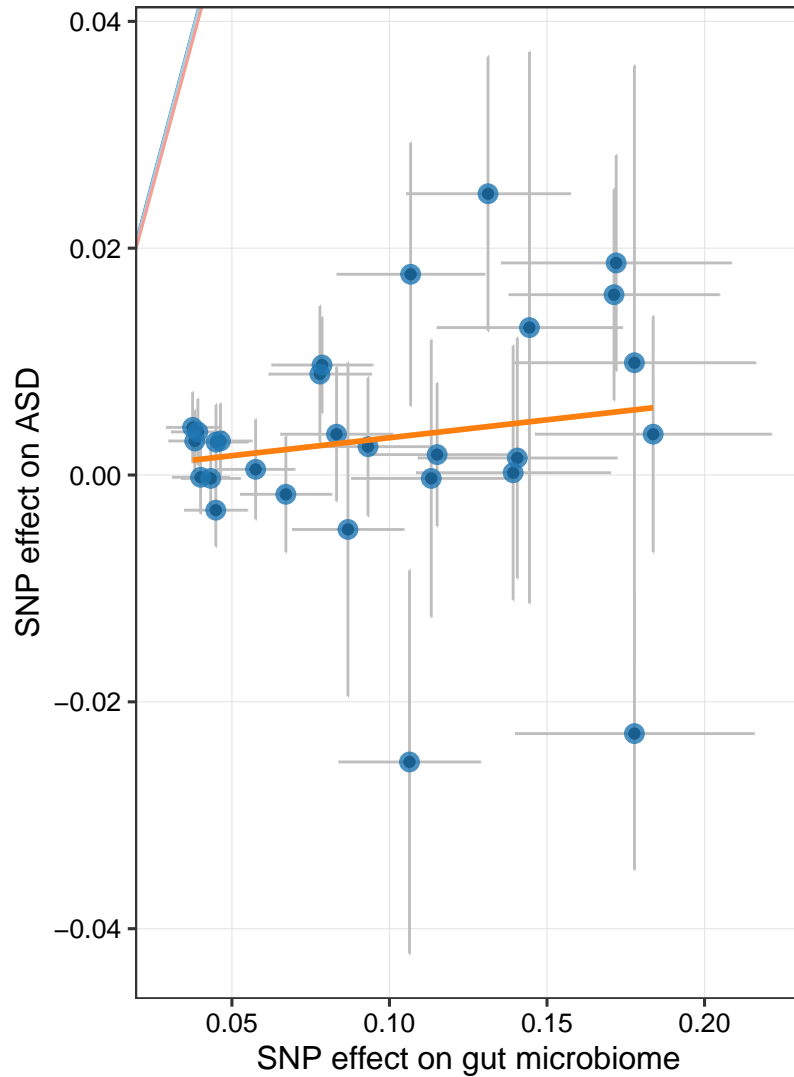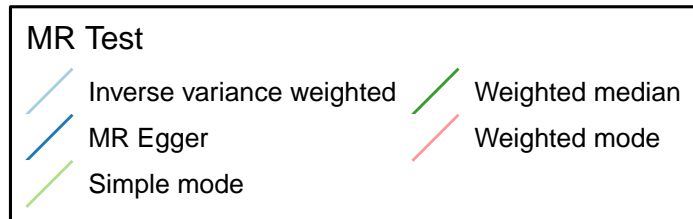

Supplement: Supplementary file 2 — Supplementary Material 2 [file 13568_2025_1969_MOESM2_ESM.zip › Revised supplementary materials/5 Forward MR analysis results/plot/scatter_or_Enorma massiliensis.pdf]

**Scatter Plot (OR): Enterococcus faecalis**

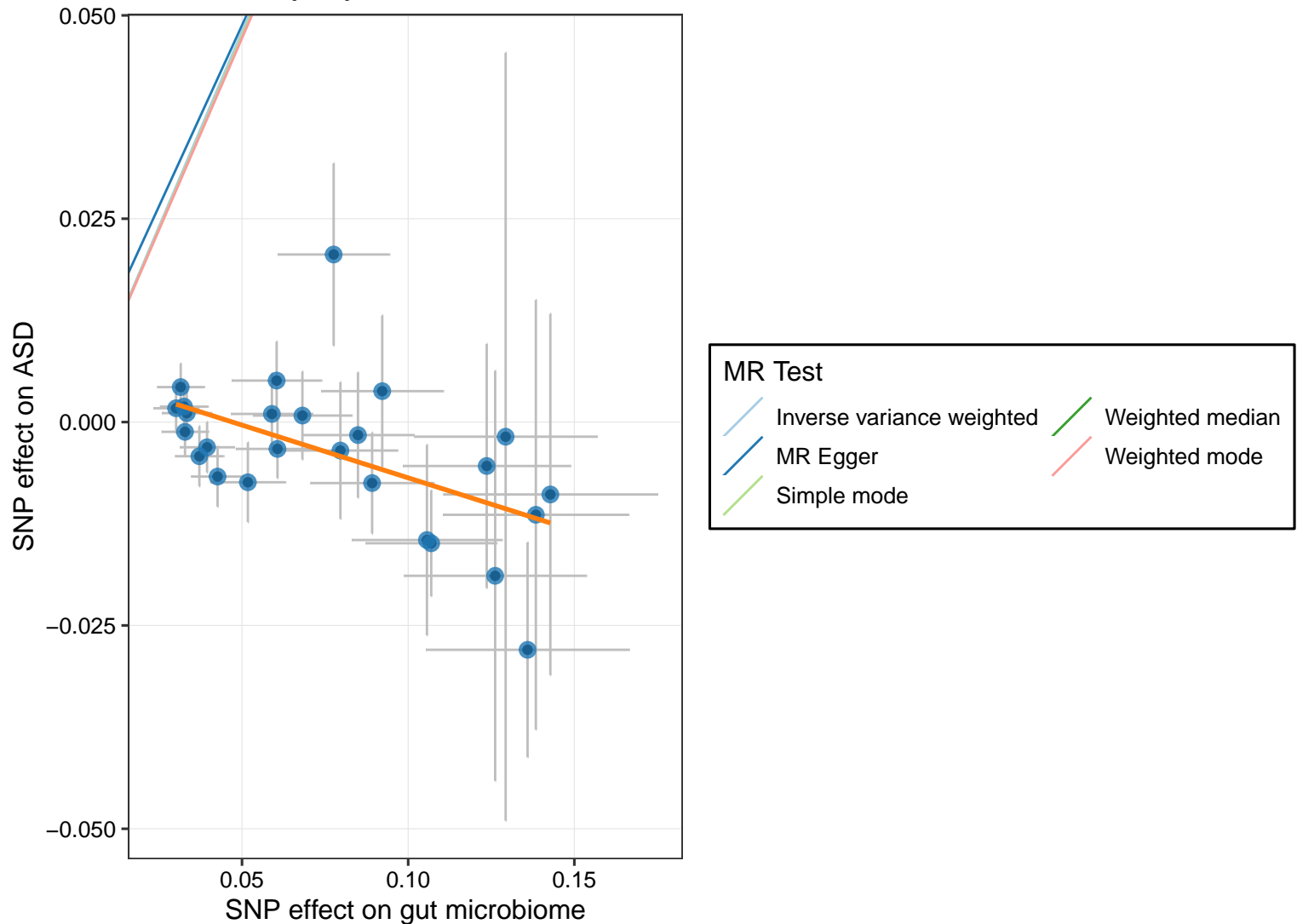

Supplement: Supplementary file 2 — Supplementary Material 2 [file 13568_2025_1969_MOESM2_ESM.zip › Revised supplementary materials/5 Forward MR analysis results/plot/scatter_or_Enterococcus faecalis.pdf]

# Scatter Plot (OR): *Eubacterium callanderi*

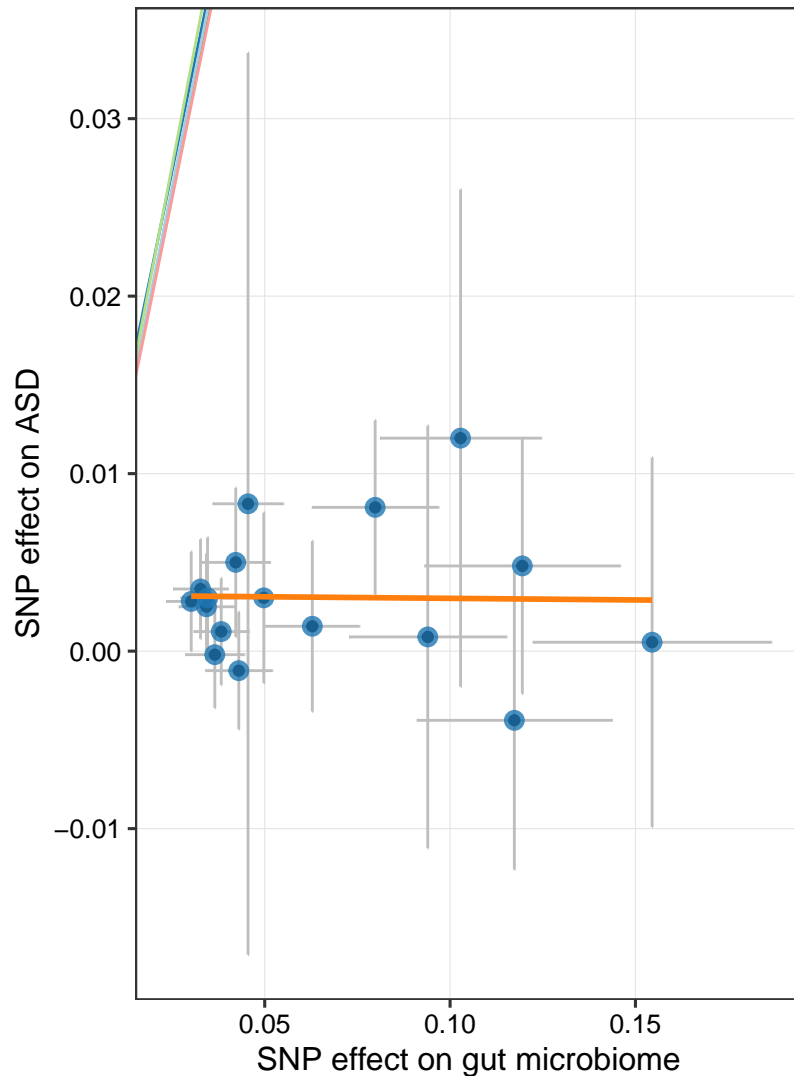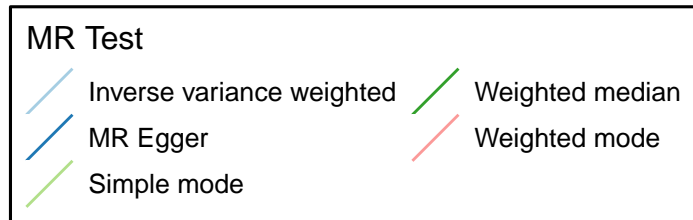

Supplement: Supplementary file 2 — Supplementary Material 2 [file 13568_2025_1969_MOESM2_ESM.zip › Revised supplementary materials/5 Forward MR analysis results/plot/scatter_or_Eubacterium callanderi.pdf]

# Scatter Plot (OR): *Faecalicatena torques*

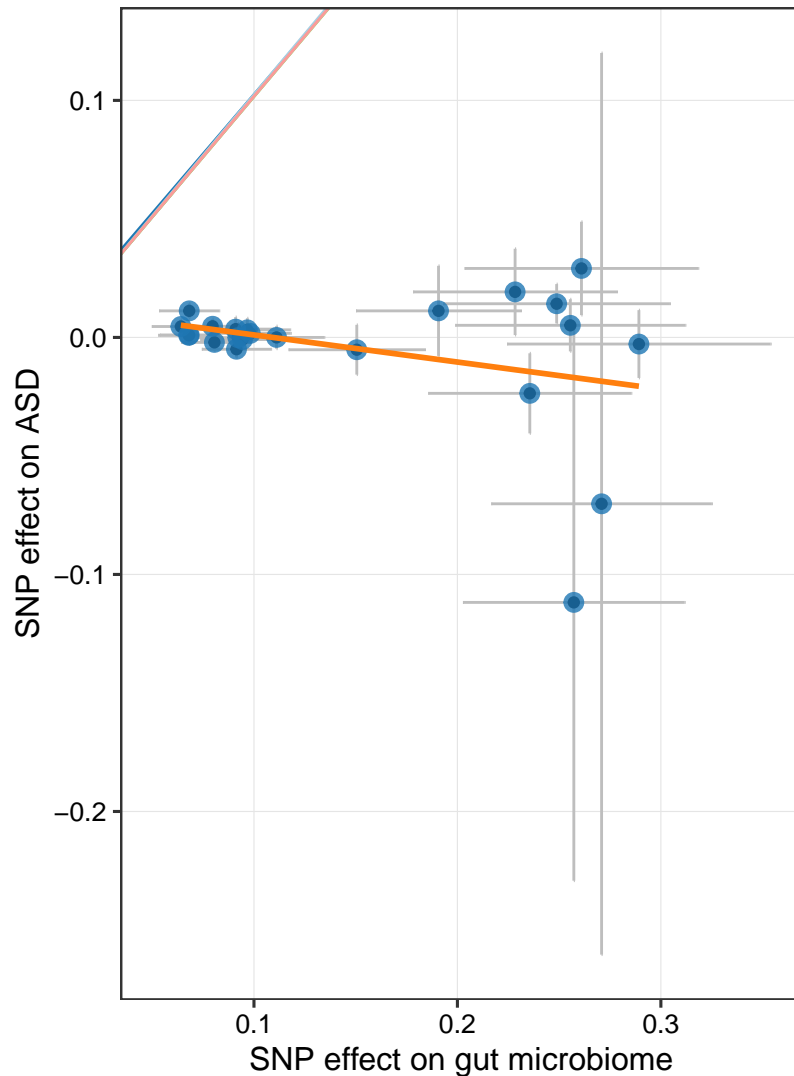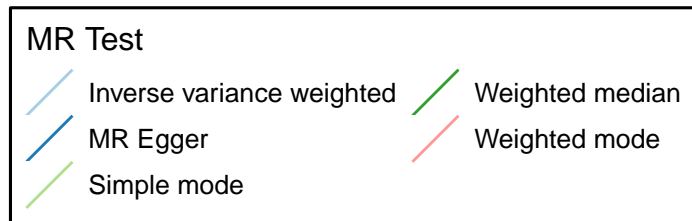

Supplement: Supplementary file 2 — Supplementary Material 2 [file 13568_2025_1969_MOESM2_ESM.zip › Revised supplementary materials/5 Forward MR analysis results/plot/scatter_or_Faecalicatena torques.pdf]

## Scatter Plot (OR): Fibrobacteria

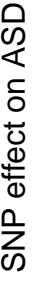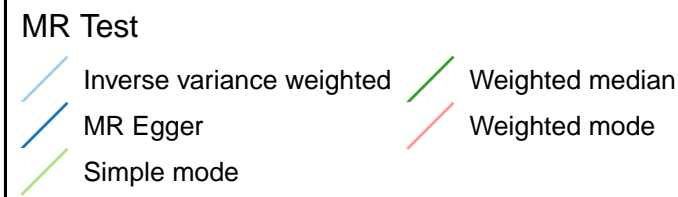

Supplement: Supplementary file 2 — Supplementary Material 2 [file 13568_2025_1969_MOESM2_ESM.zip › Revised supplementary materials/5 Forward MR analysis results/plot/scatter_or_Fibrobacteria.pdf]

**Scatter Plot (OR): Francisellaceae**

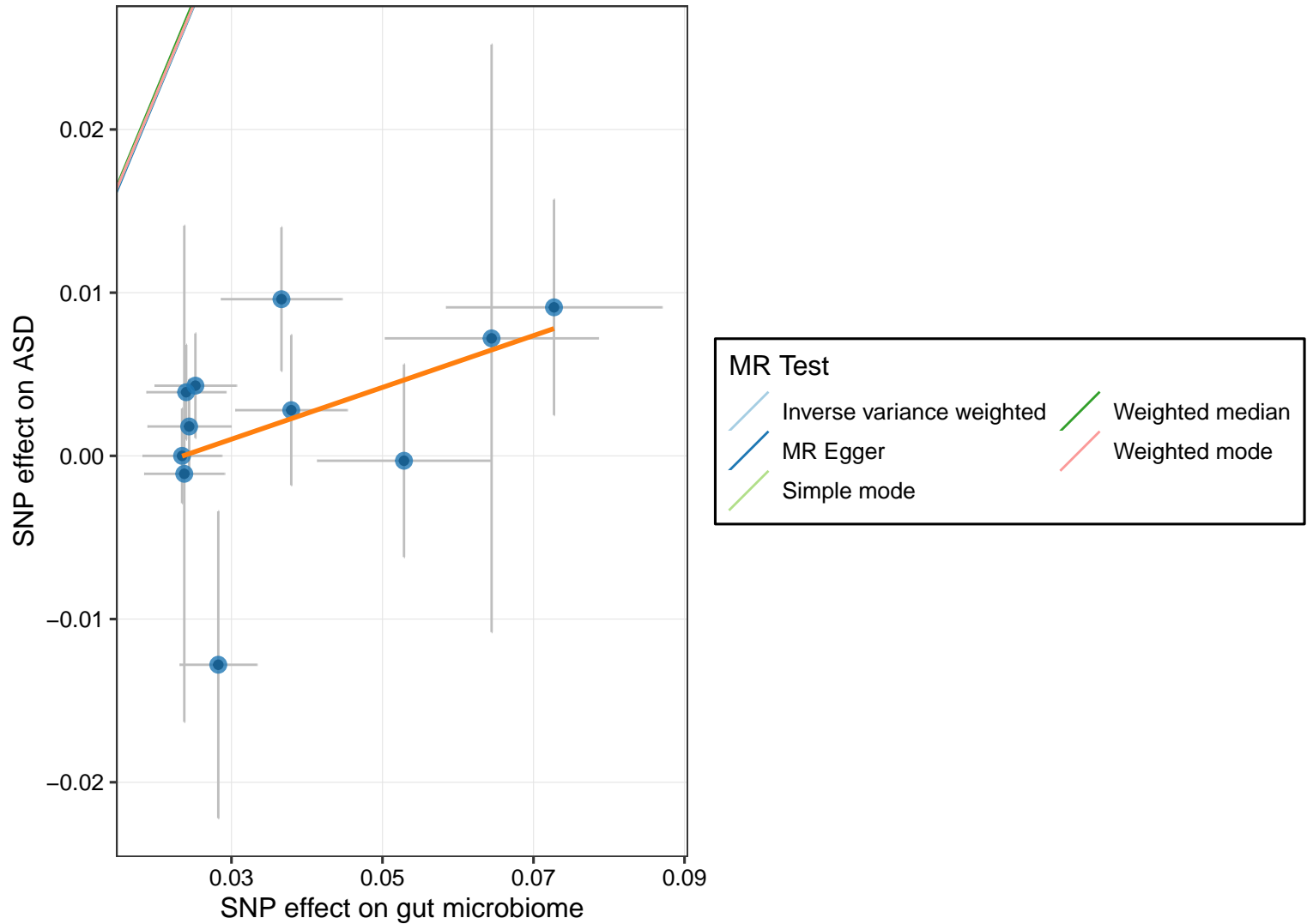

Supplement: Supplementary file 2 — Supplementary Material 2 [file 13568_2025_1969_MOESM2_ESM.zip › Revised supplementary materials/5 Forward MR analysis results/plot/scatter_or_Francisellaceae.pdf]

**Scatter Plot (OR): Geminocystis**

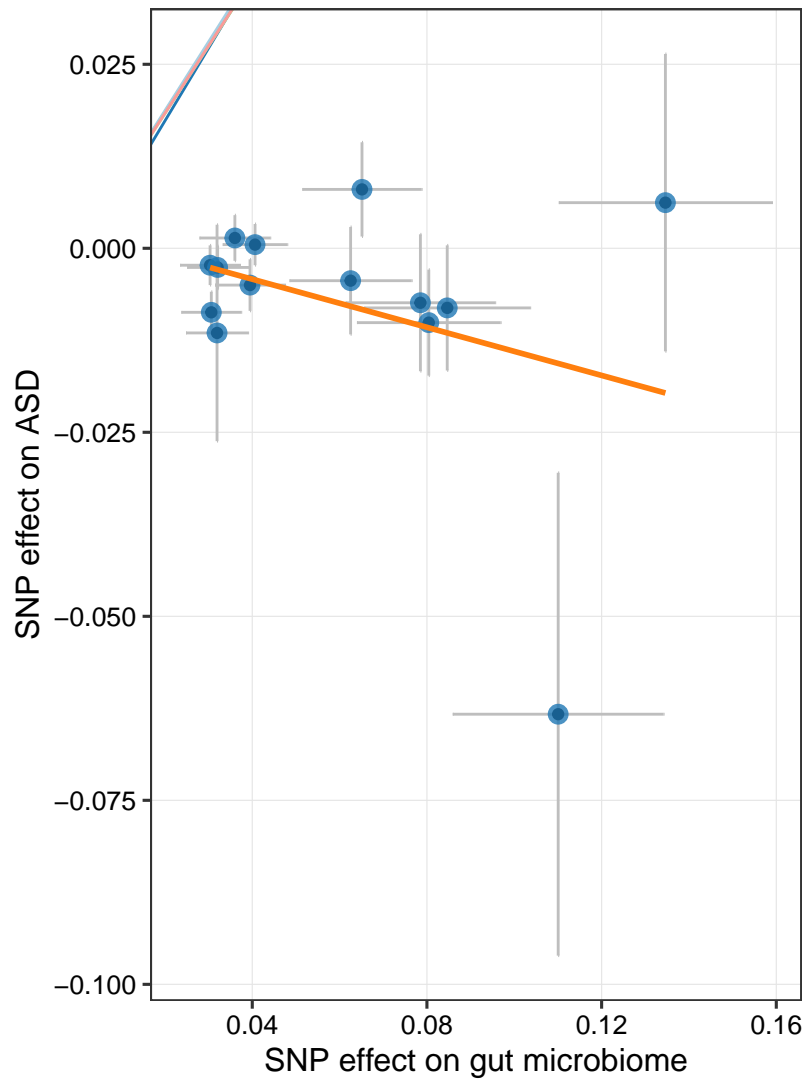

Supplement: Supplementary file 2 — Supplementary Material 2 [file 13568_2025_1969_MOESM2_ESM.zip › Revised supplementary materials/5 Forward MR analysis results/plot/scatter_or_Geminocystis.pdf]

**Scatter Plot (OR): koll11**

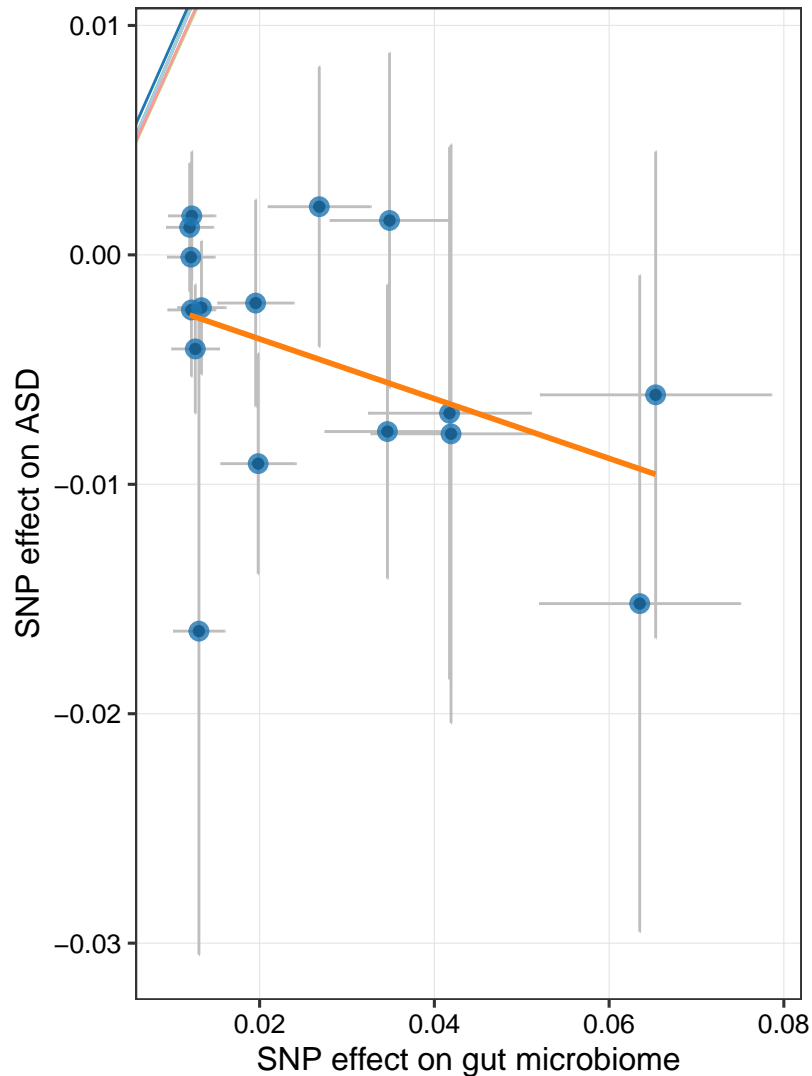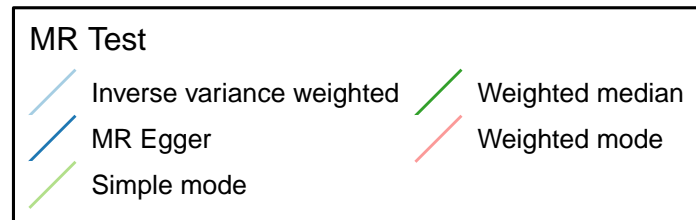

Supplement: Supplementary file 2 — Supplementary Material 2 [file 13568_2025_1969_MOESM2_ESM.zip › Revised supplementary materials/5 Forward MR analysis results/plot/scatter_or_koll11.pdf]

**Scatter Plot (OR): *Lachnospira rogosae***

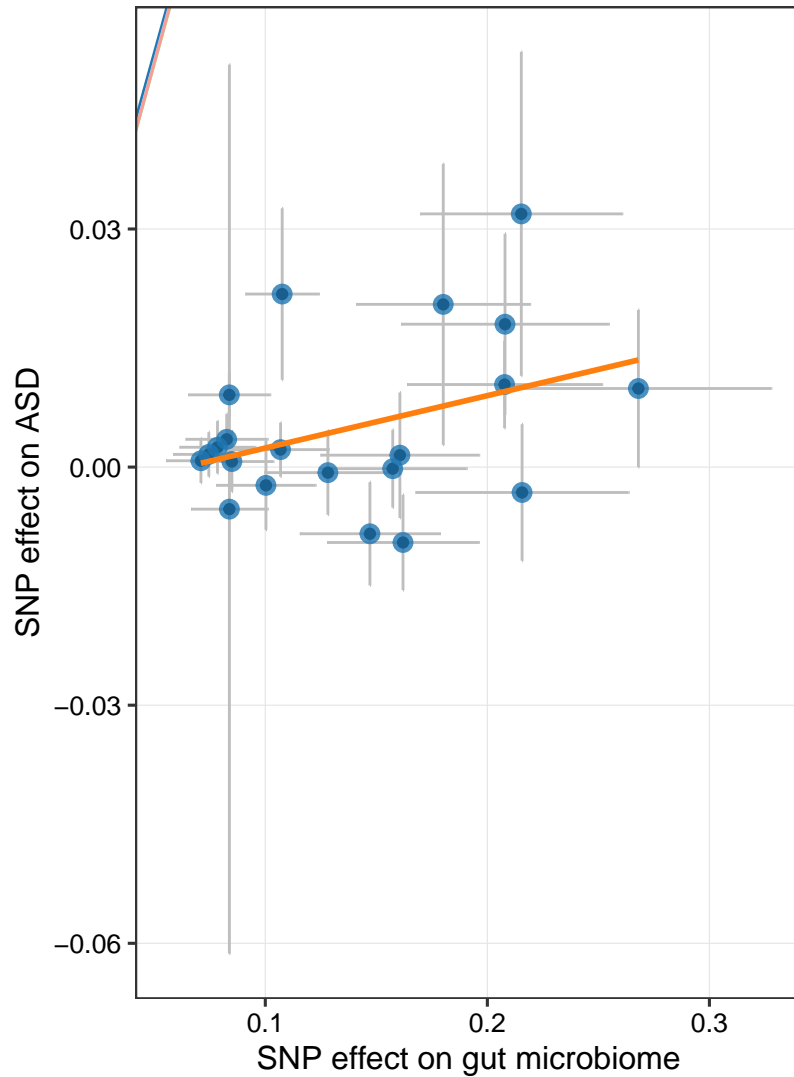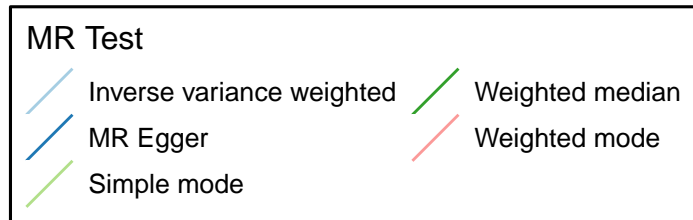

Supplement: Supplementary file 2 — Supplementary Material 2 [file 13568_2025_1969_MOESM2_ESM.zip › Revised supplementary materials/5 Forward MR analysis results/plot/scatter_or_Lachnospira rogosae.pdf]

**Scatter Plot (OR): Olsenella C**

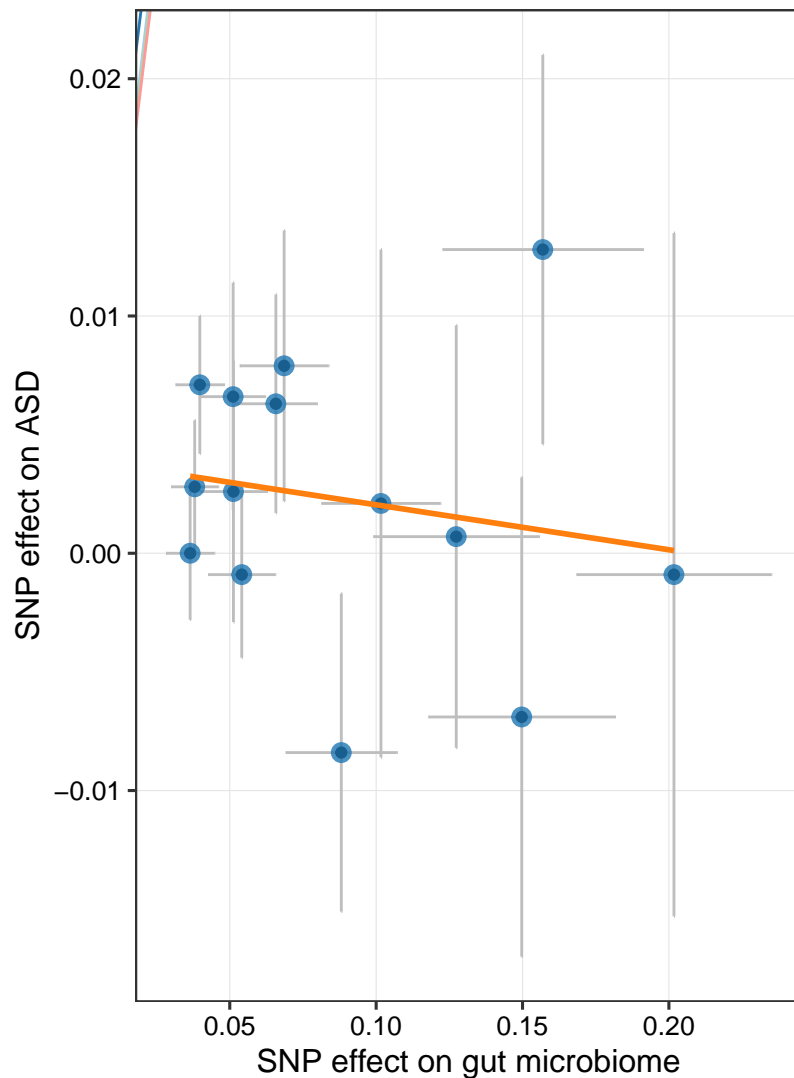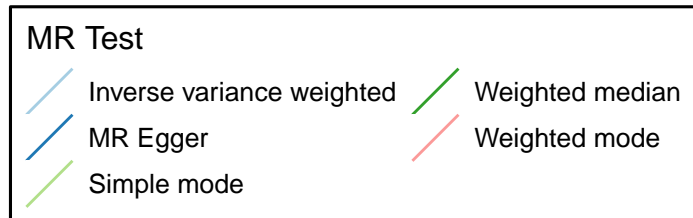

Supplement: Supplementary file 2 — Supplementary Material 2 [file 13568_2025_1969_MOESM2_ESM.zip › Revised supplementary materials/5 Forward MR analysis results/plot/scatter_or_Olsenella C.pdf]

**Scatter Plot (OR): Parabacteroides**

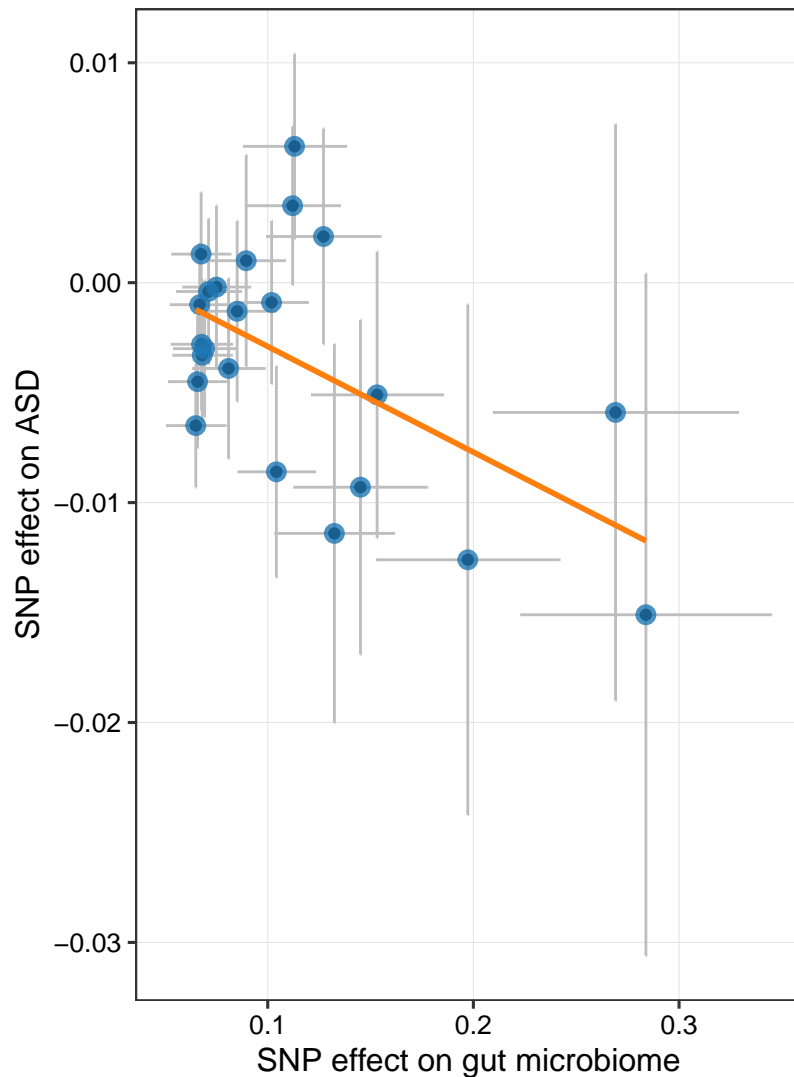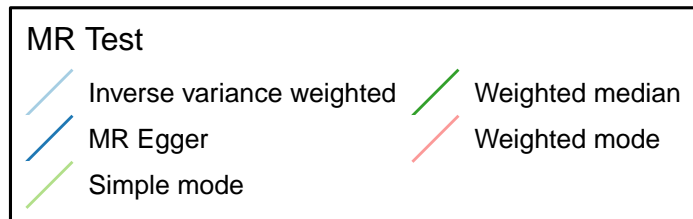

Supplement: Supplementary file 2 — Supplementary Material 2 [file 13568_2025_1969_MOESM2_ESM.zip › Revised supplementary materials/5 Forward MR analysis results/plot/scatter_or_Parabacteroides.pdf]

**Scatter Plot (OR): Prevotella sp002933775**

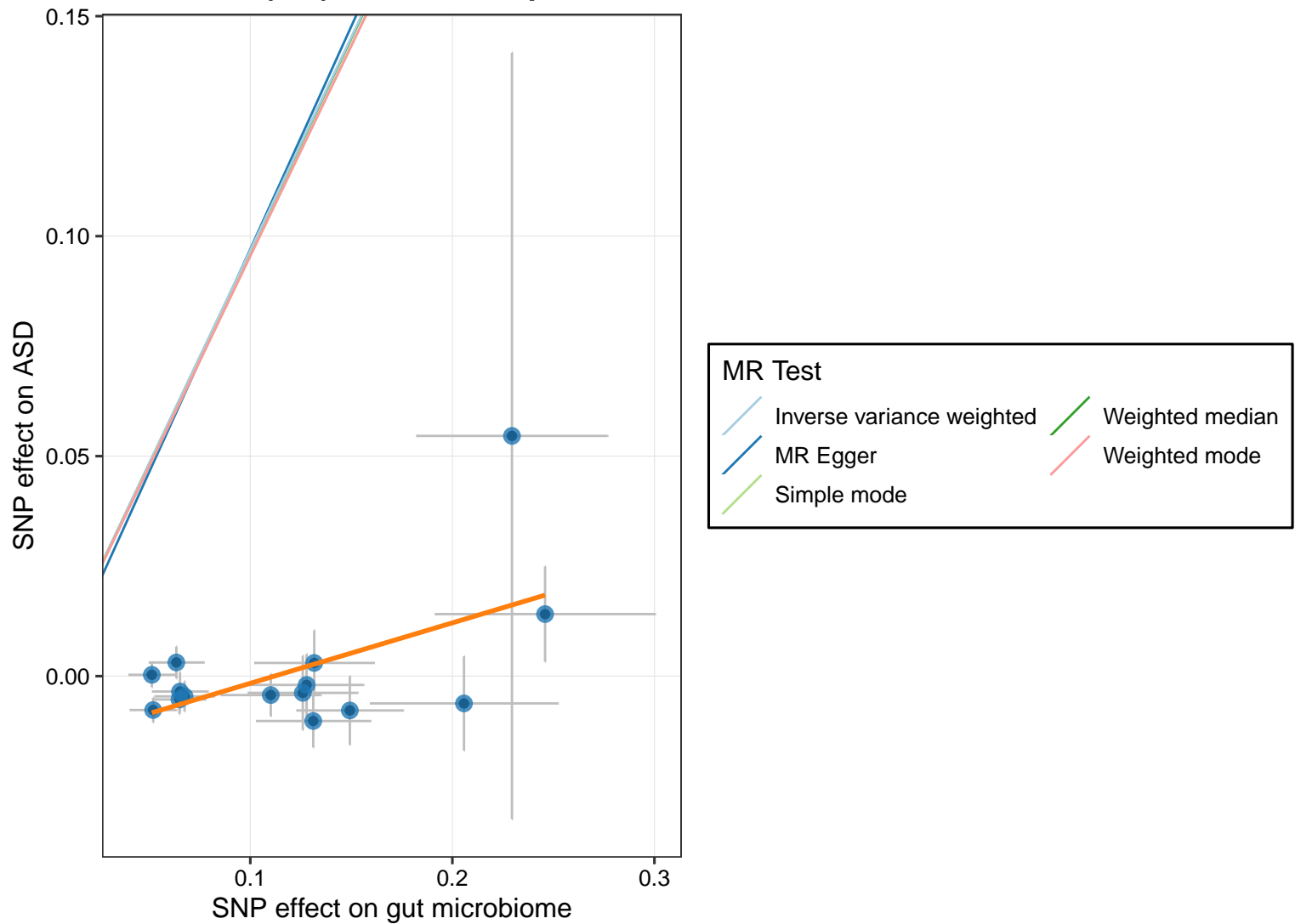

Supplement: Supplementary file 2 — Supplementary Material 2 [file 13568_2025_1969_MOESM2_ESM.zip › Revised supplementary materials/5 Forward MR analysis results/plot/scatter_or_Prevotella sp002933775.pdf]

**Scatter Plot (OR): UBA1066**

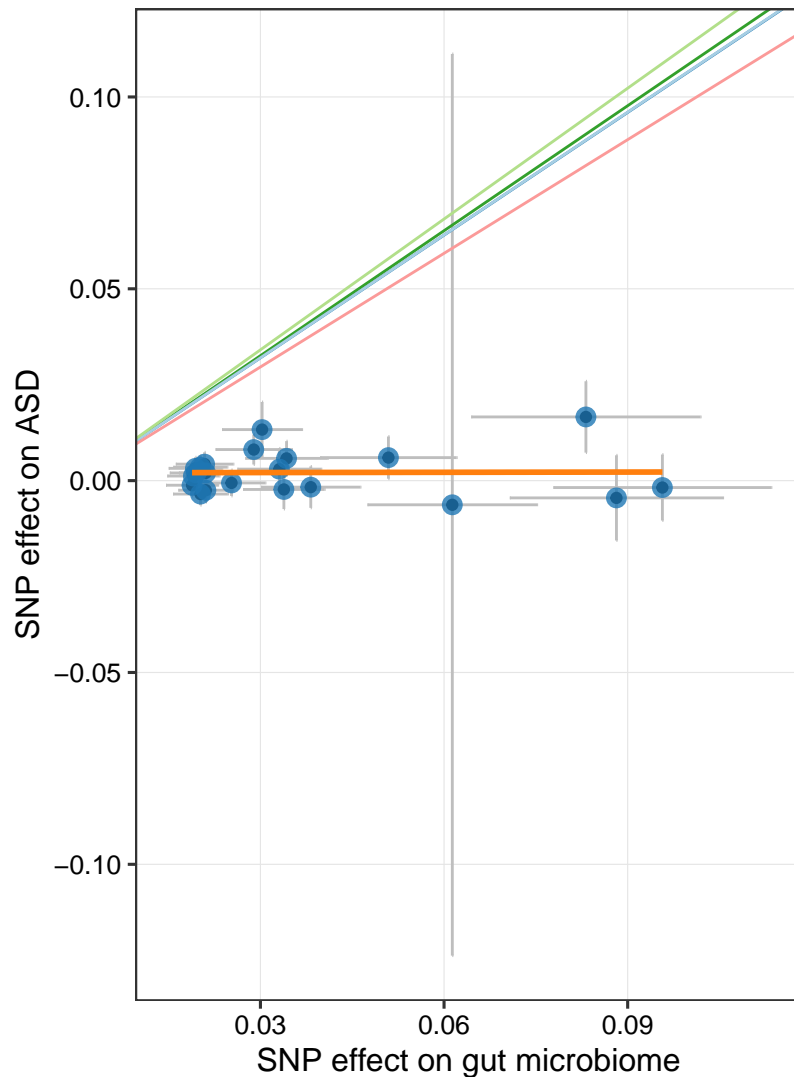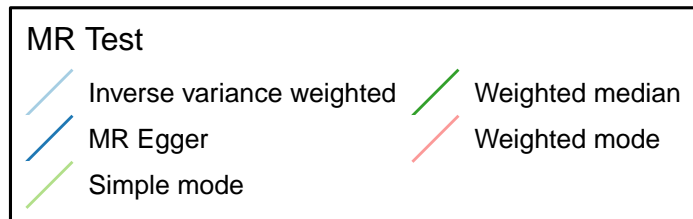

Supplement: Supplementary file 2 — Supplementary Material 2 [file 13568_2025_1969_MOESM2_ESM.zip › Revised supplementary materials/5 Forward MR analysis results/plot/scatter_or_UBA1066.pdf]

**Scatter Plot (OR): UBA7703**

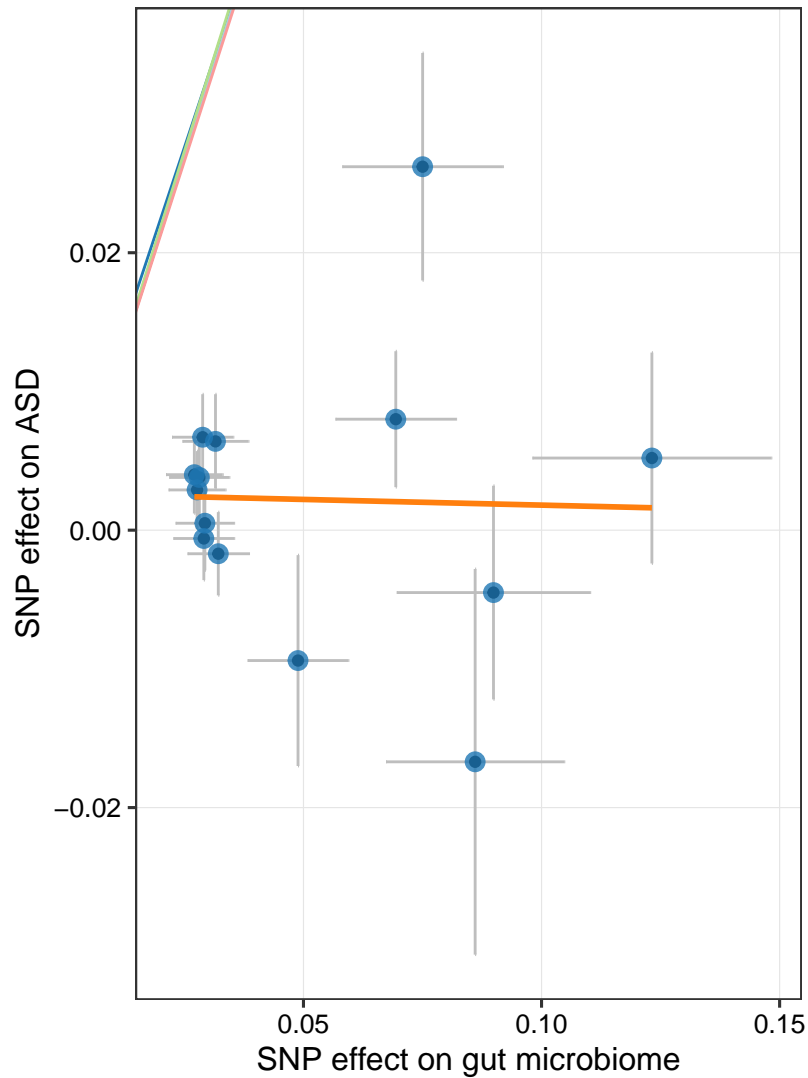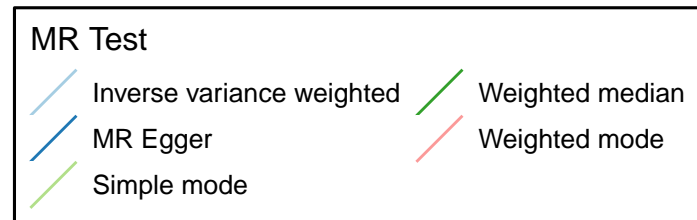

Supplement: Supplementary file 2 — Supplementary Material 2 [file 13568_2025_1969_MOESM2_ESM.zip › Revised supplementary materials/5 Forward MR analysis results/plot/scatter_or_UBA7703.pdf]

**Scatter Plot (OR): UBA8904**

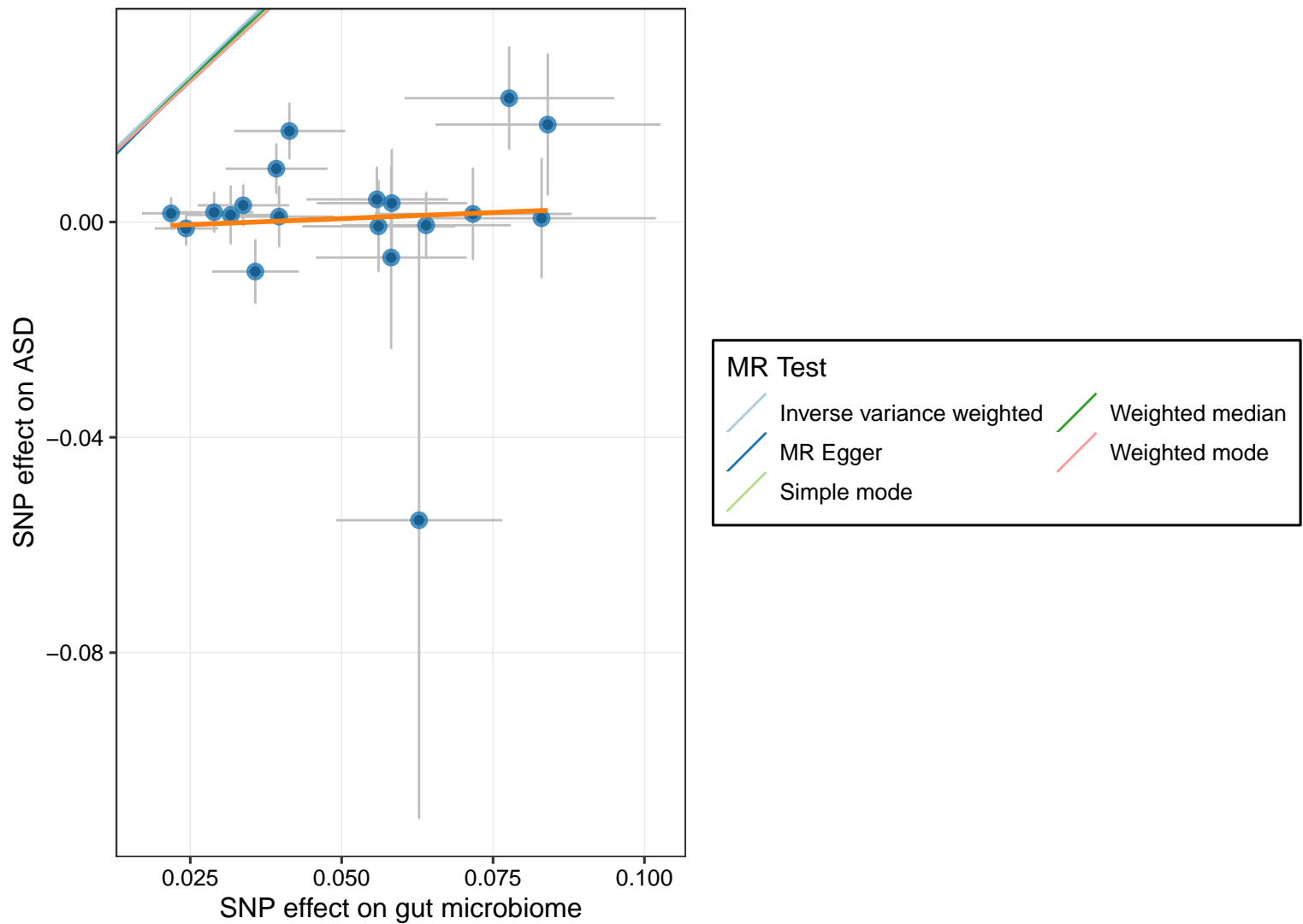

Supplement: Supplementary file 2 — Supplementary Material 2 [file 13568_2025_1969_MOESM2_ESM.zip › Revised supplementary materials/5 Forward MR analysis results/plot/scatter_or_UBA8904.pdf]

**Scatter Plot (OR): V9D3004**

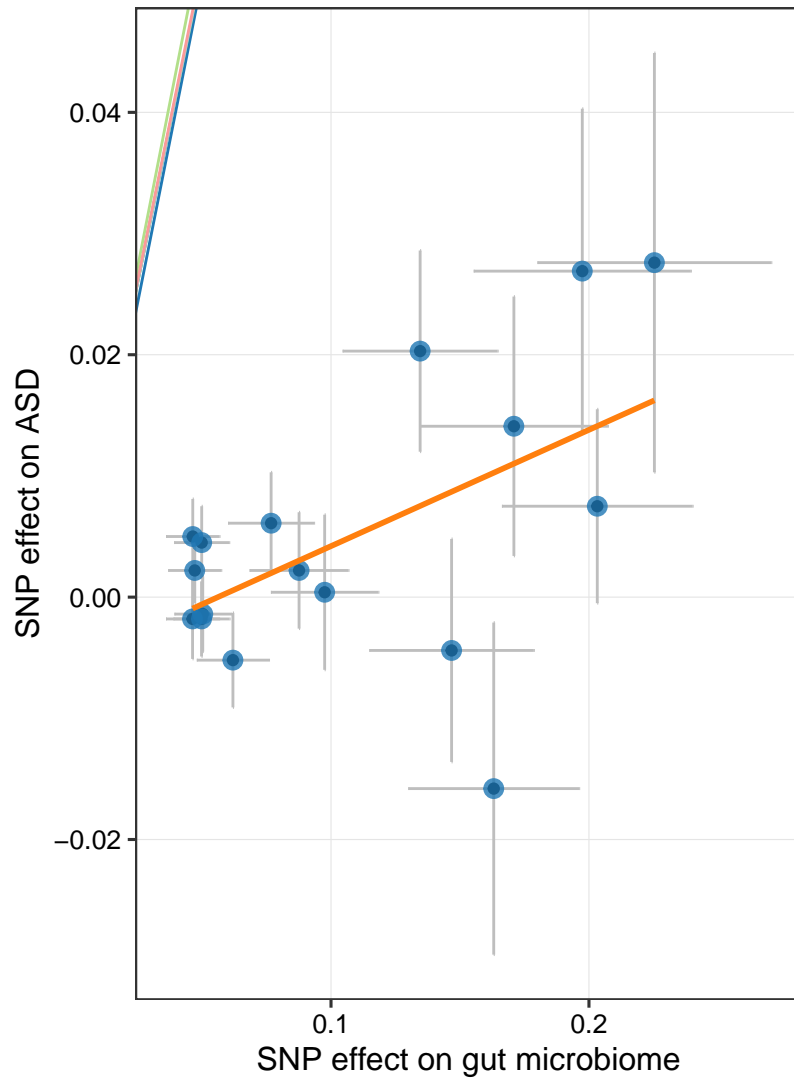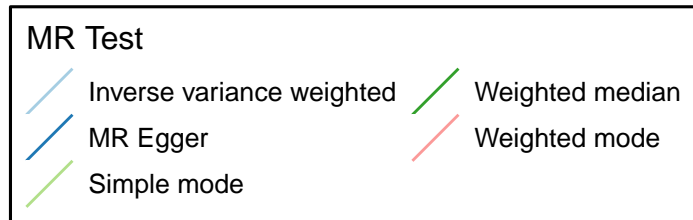

Supplement: Supplementary file 2 — Supplementary Material 2 [file 13568_2025_1969_MOESM2_ESM.zip › Revised supplementary materials/5 Forward MR analysis results/plot/scatter_or_V9D3004.pdf]

# Forest Plot (OR): Alistipes shahii

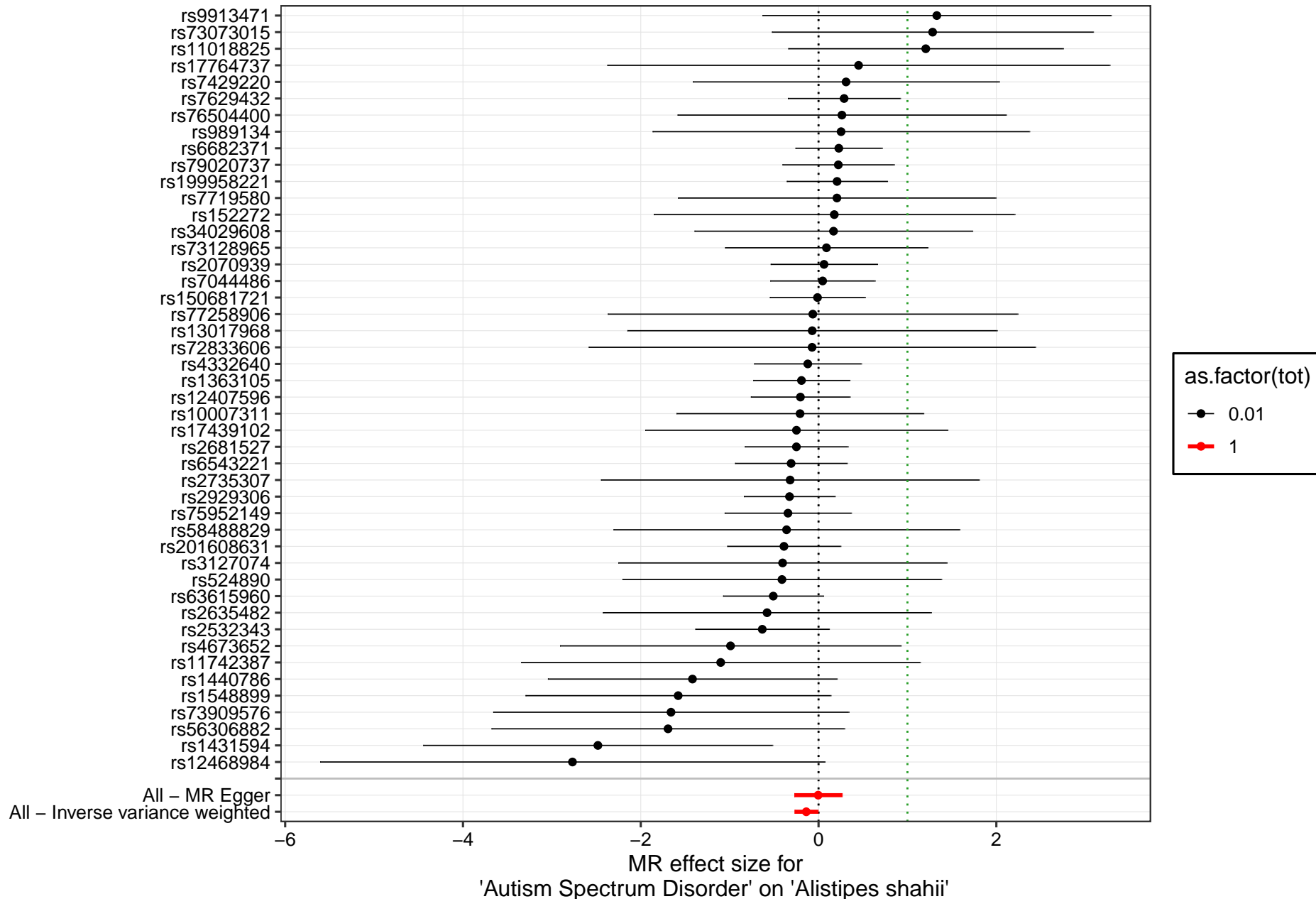

Supplement: Supplementary file 2 — Supplementary Material 2 [file 13568_2025_1969_MOESM2_ESM.zip › Revised supplementary materials/6 Inverse MR analysis results/plot/forest_or_Alistipes shahii.pdf]

# Forest Plot (OR): An181

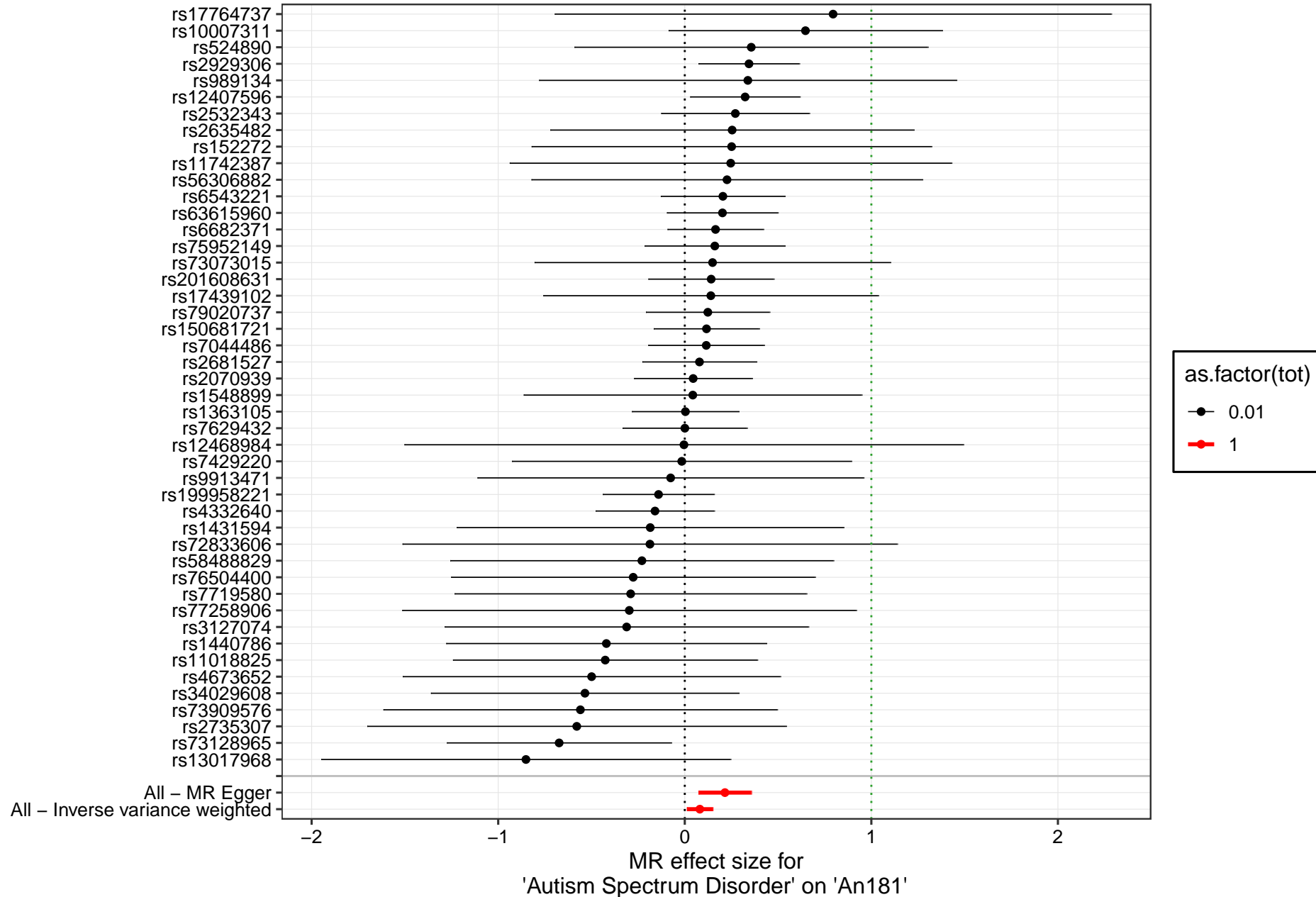

Supplement: Supplementary file 2 — Supplementary Material 2 [file 13568_2025_1969_MOESM2_ESM.zip › Revised supplementary materials/6 Inverse MR analysis results/plot/forest_or_An181.pdf]

# Forest Plot (OR): Aureimonas

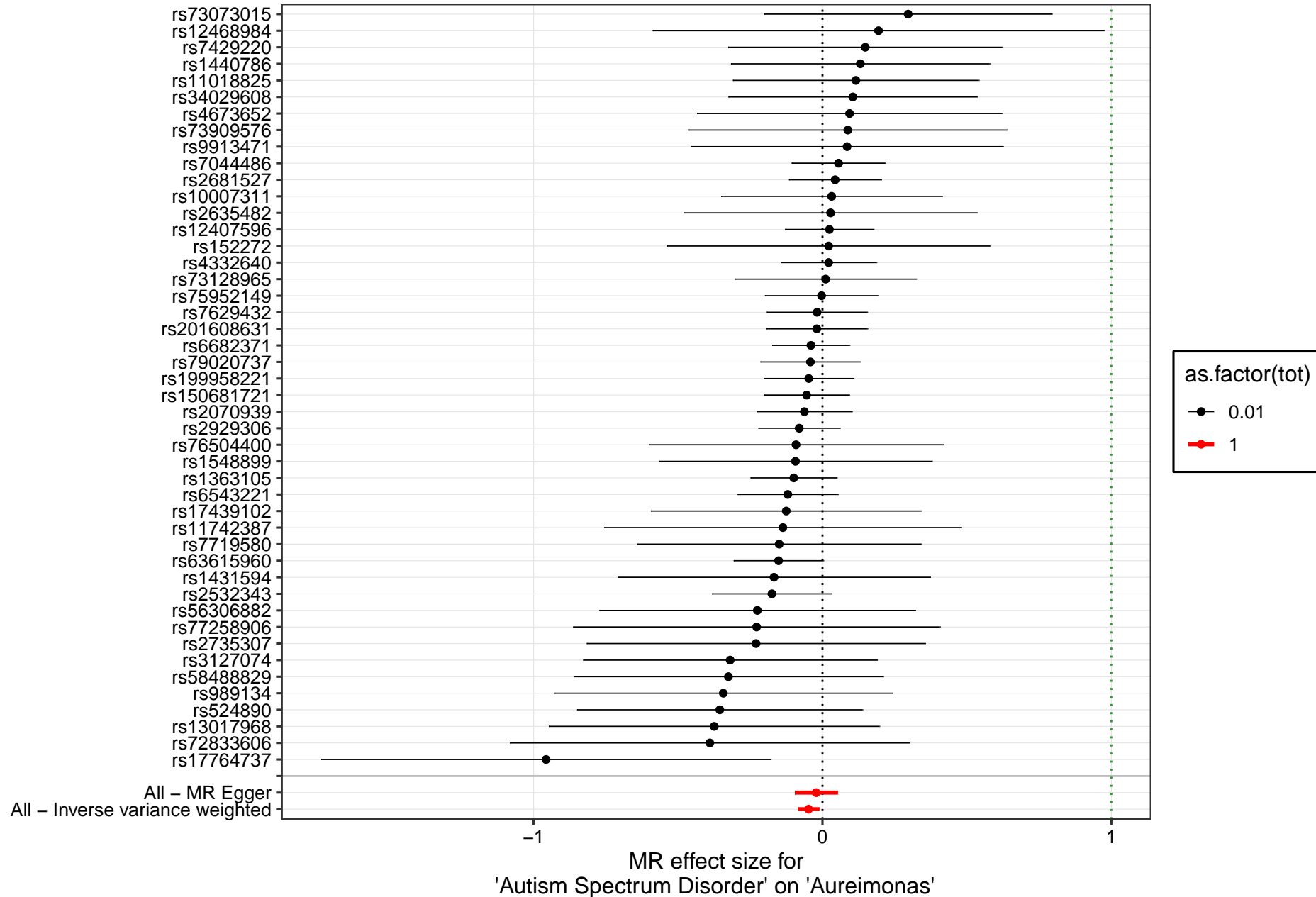

Supplement: Supplementary file 2 — Supplementary Material 2 [file 13568_2025_1969_MOESM2_ESM.zip › Revised supplementary materials/6 Inverse MR analysis results/plot/forest_or_Aureimonas.pdf]

# Forest Plot (OR): Blautia A sp002159835

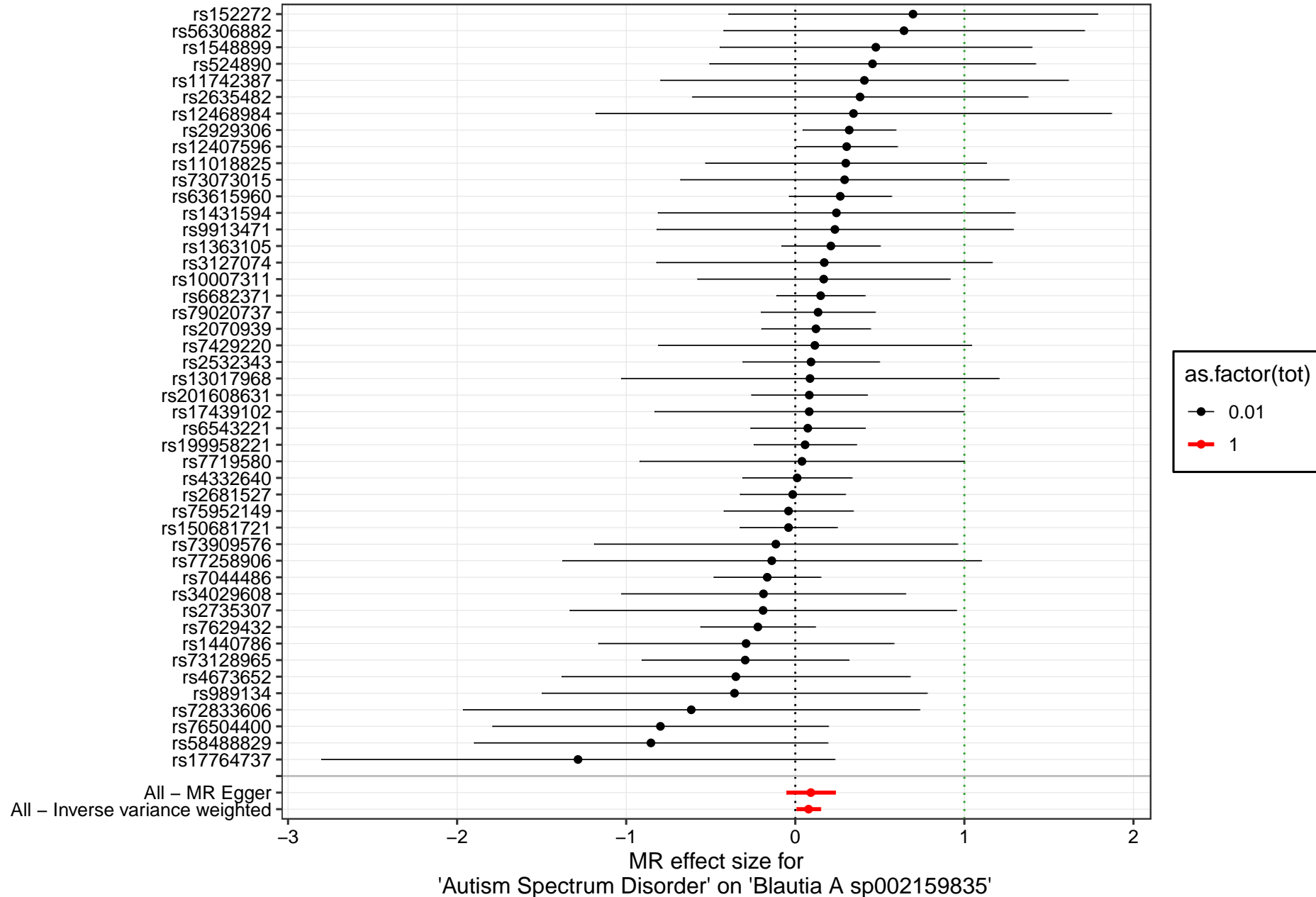

Supplement: Supplementary file 2 — Supplementary Material 2 [file 13568_2025_1969_MOESM2_ESM.zip › Revised supplementary materials/6 Inverse MR analysis results/plot/forest_or_Blautia A sp002159835.pdf]

# Forest Plot (OR): CAG-302

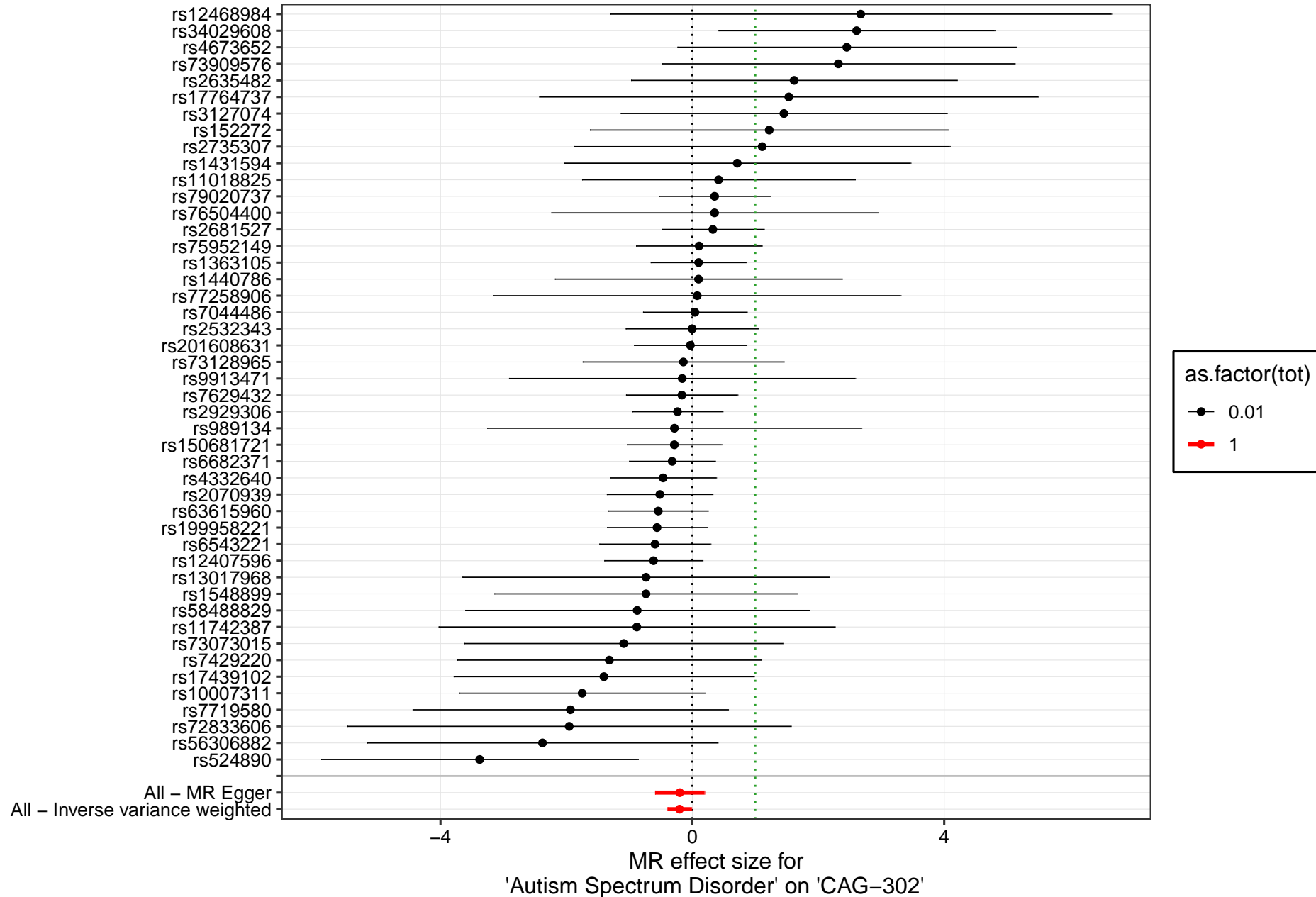

Supplement: Supplementary file 2 — Supplementary Material 2 [file 13568_2025_1969_MOESM2_ESM.zip › Revised supplementary materials/6 Inverse MR analysis results/plot/forest_or_CAG-302.pdf]

# Forest Plot (OR): CAG-485 sp002404675

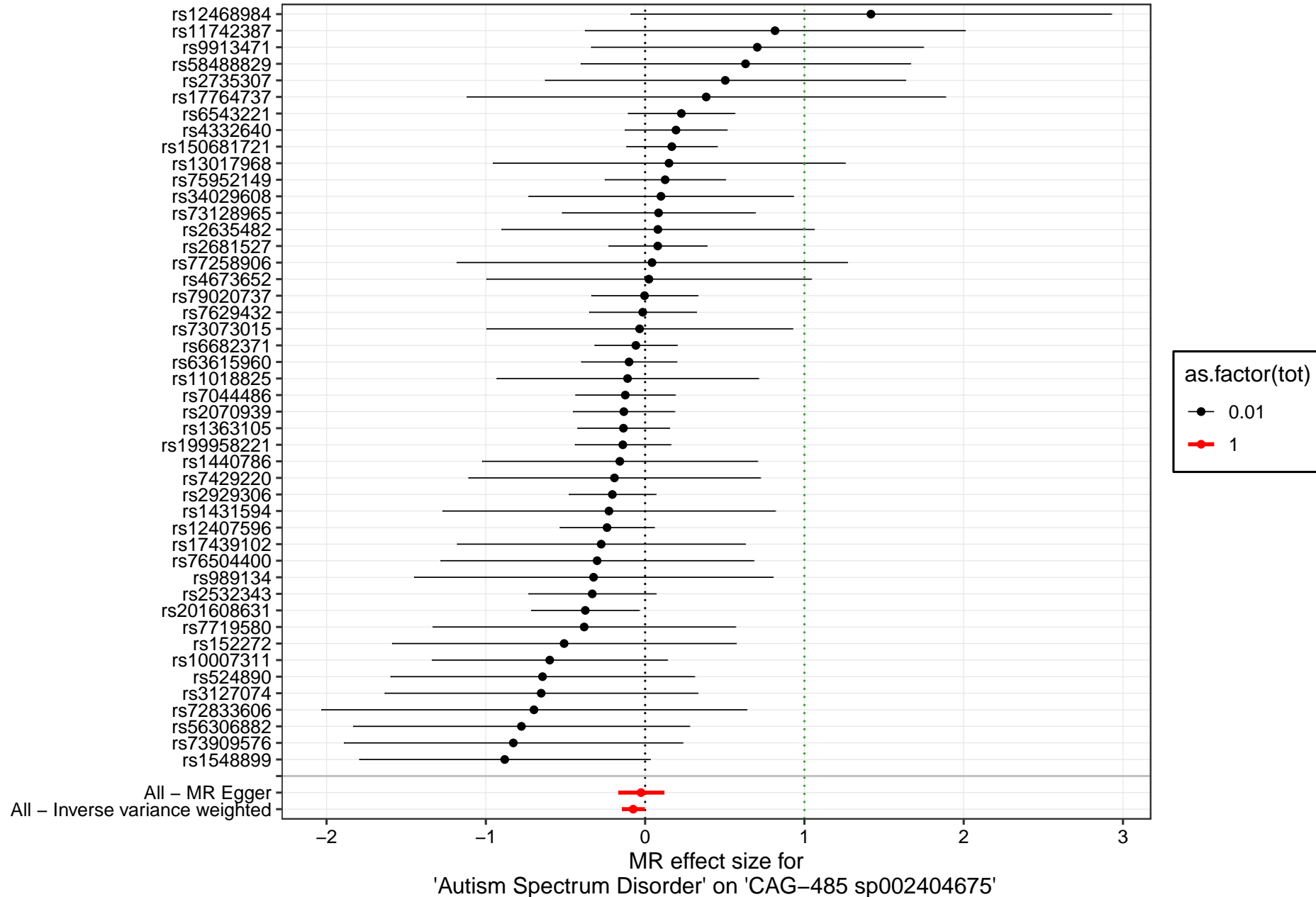

Supplement: Supplementary file 2 — Supplementary Material 2 [file 13568_2025_1969_MOESM2_ESM.zip › Revised supplementary materials/6 Inverse MR analysis results/plot/forest_or_CAG-485 sp002404675.pdf]

# Forest Plot (OR): CAG-83 sp002392625

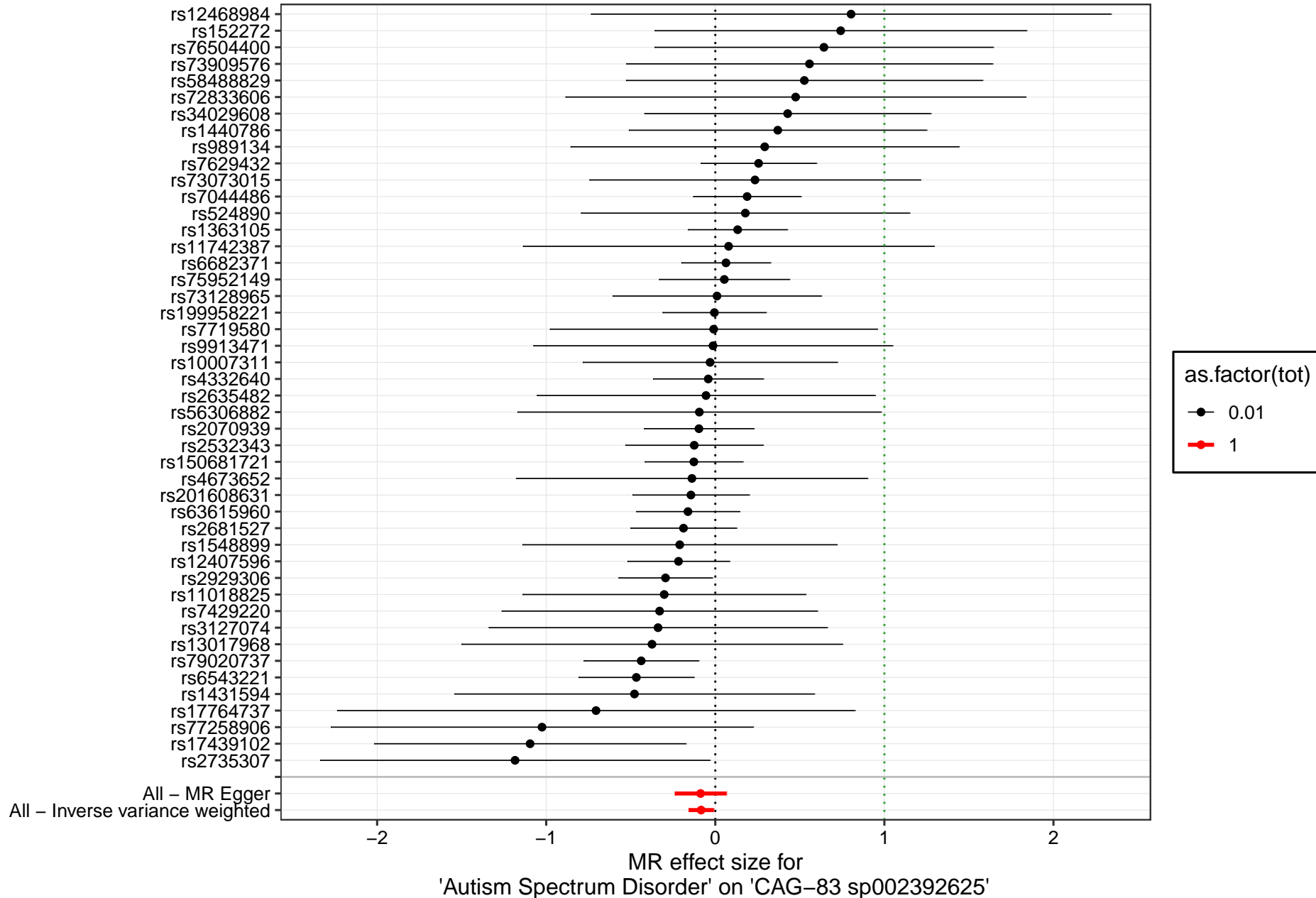

Supplement: Supplementary file 2 — Supplementary Material 2 [file 13568_2025_1969_MOESM2_ESM.zip › Revised supplementary materials/6 Inverse MR analysis results/plot/forest_or_CAG-83 sp002392625.pdf]

# Forest Plot (OR): Enterococcus A

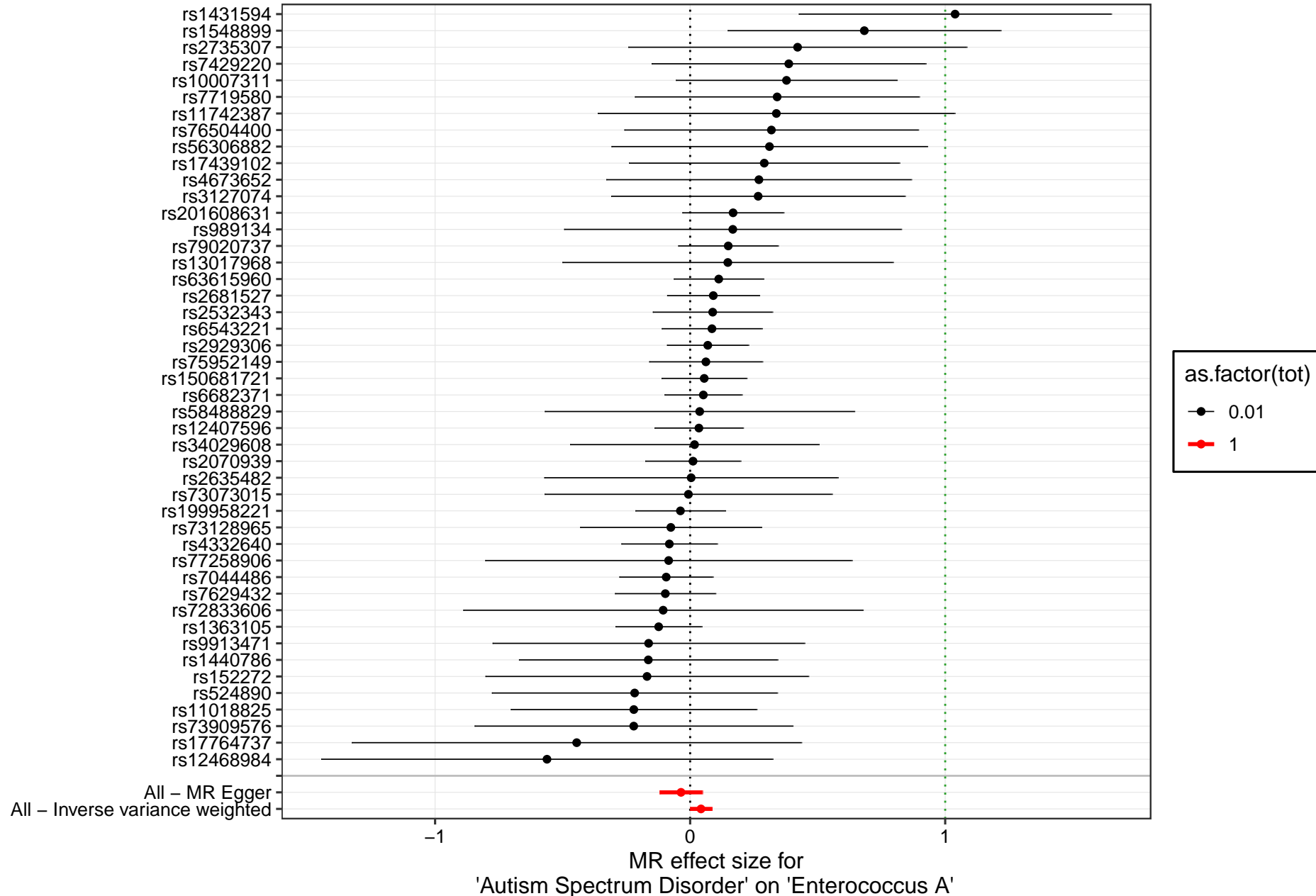

Supplement: Supplementary file 2 — Supplementary Material 2 [file 13568_2025_1969_MOESM2_ESM.zip › Revised supplementary materials/6 Inverse MR analysis results/plot/forest_or_Enterococcus A.pdf]

# Forest Plot (OR): Enterococcus B

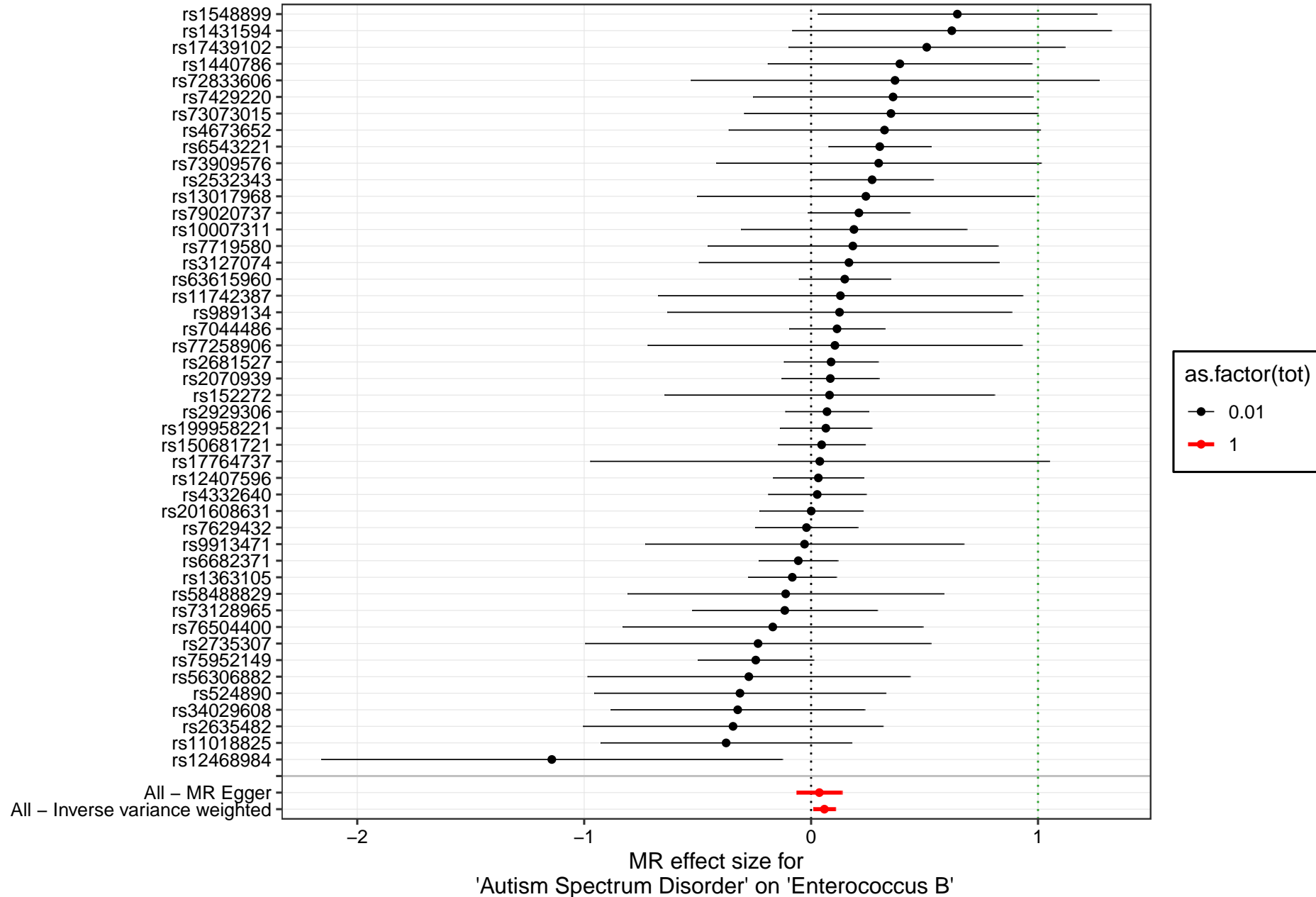

Supplement: Supplementary file 2 — Supplementary Material 2 [file 13568_2025_1969_MOESM2_ESM.zip › Revised supplementary materials/6 Inverse MR analysis results/plot/forest_or_Enterococcus B.pdf]

# Forest Plot (OR): Faecalibacterium sp002160895

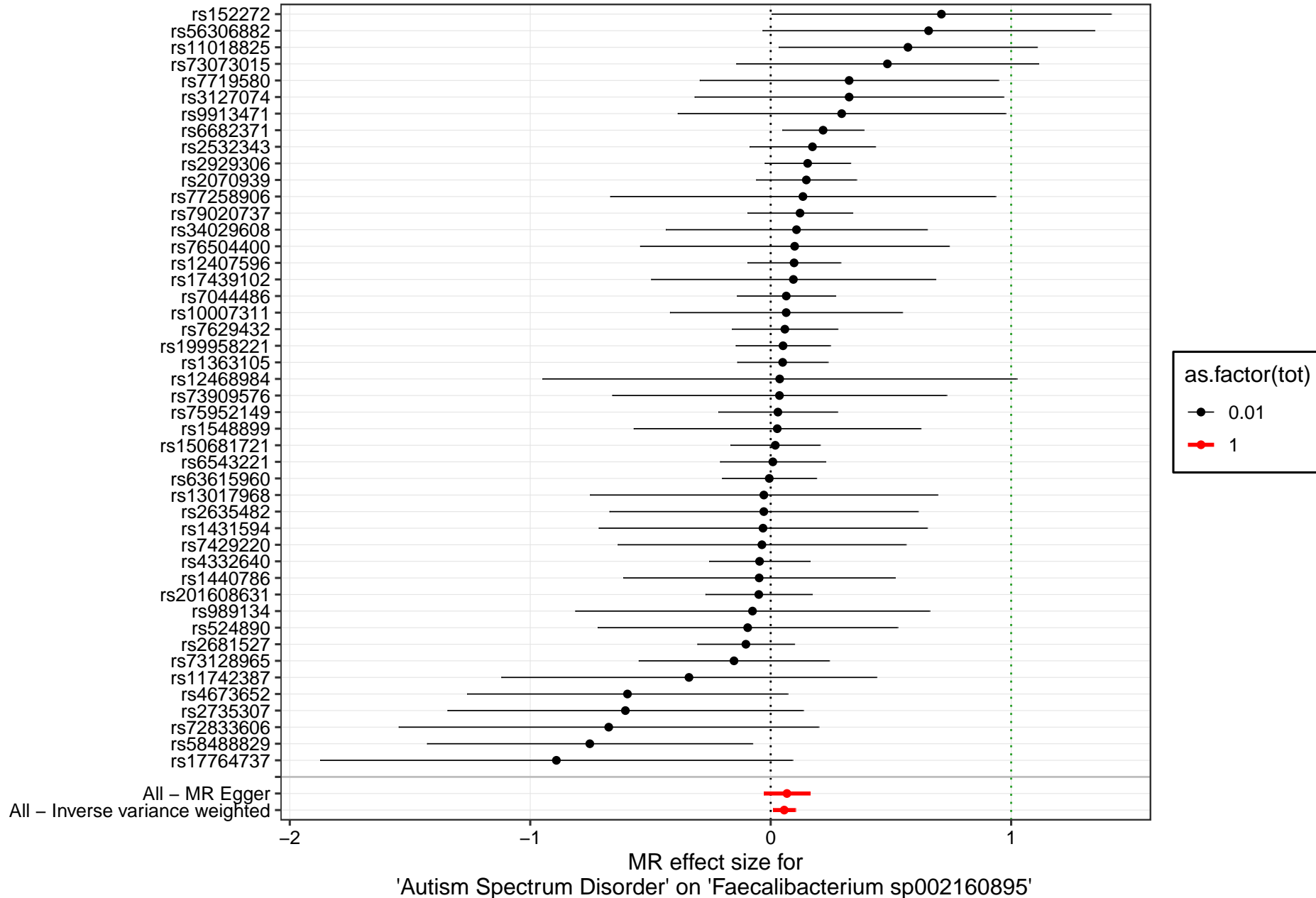

Supplement: Supplementary file 2 — Supplementary Material 2 [file 13568_2025_1969_MOESM2_ESM.zip › Revised supplementary materials/6 Inverse MR analysis results/plot/forest_or_Faecalibacterium sp002160895.pdf]

# Forest Plot (OR): Gemmatimonadaceae

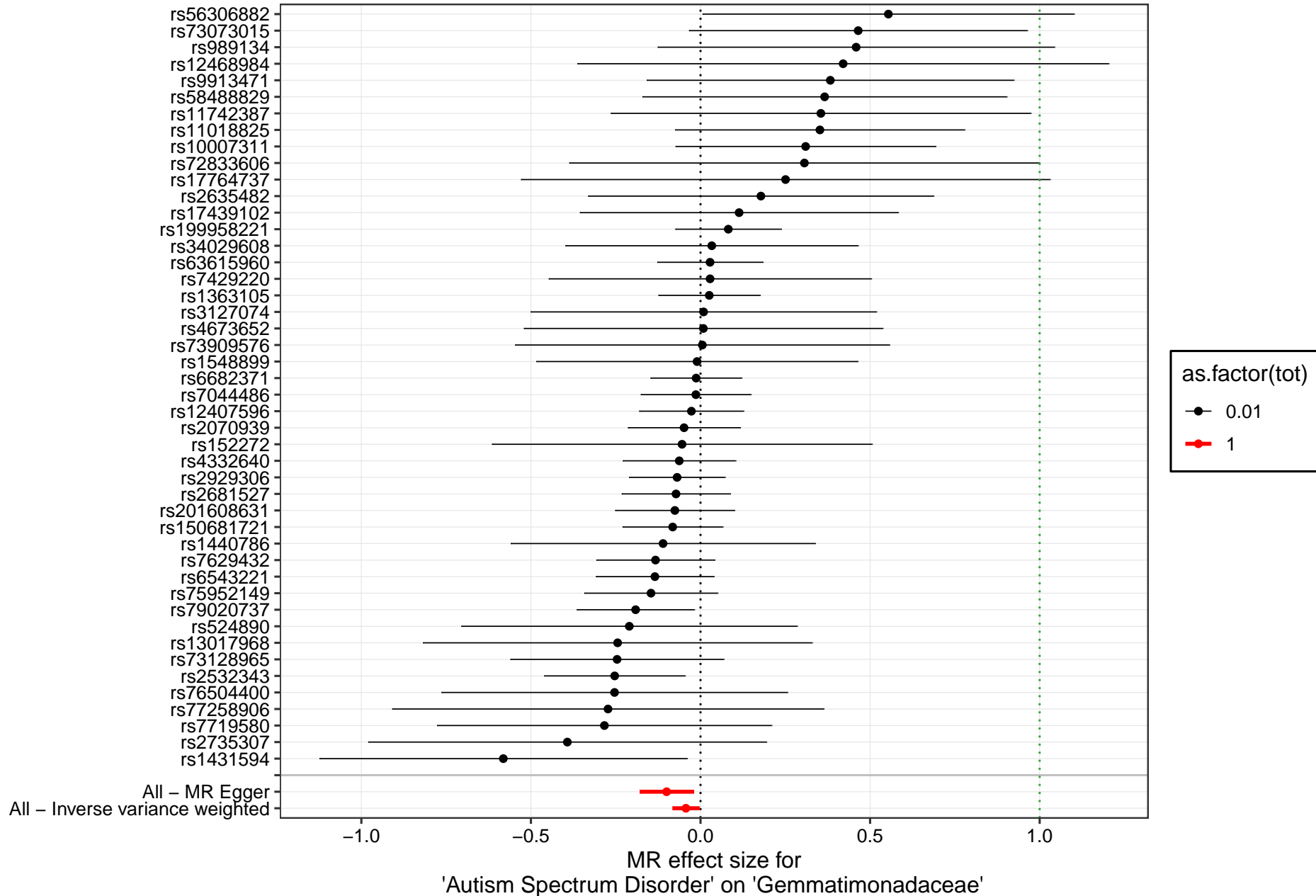

Supplement: Supplementary file 2 — Supplementary Material 2 [file 13568_2025_1969_MOESM2_ESM.zip › Revised supplementary materials/6 Inverse MR analysis results/plot/forest_or_Gemmatimonadaceae.pdf]

# Forest Plot (OR): Lactobacillus B

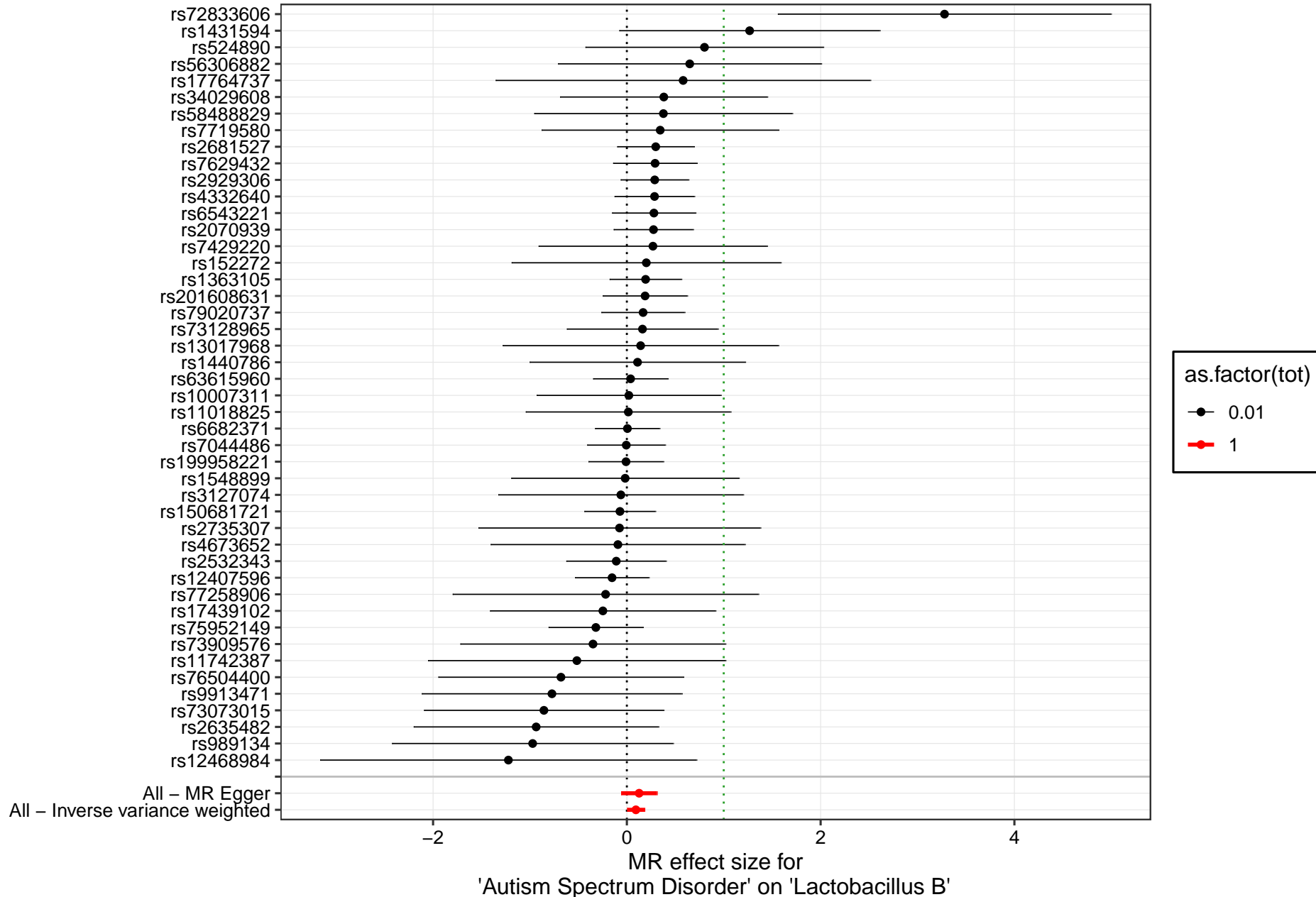

Supplement: Supplementary file 2 — Supplementary Material 2 [file 13568_2025_1969_MOESM2_ESM.zip › Revised supplementary materials/6 Inverse MR analysis results/plot/forest_or_Lactobacillus B.pdf]

# Forest Plot (OR): Microvirga

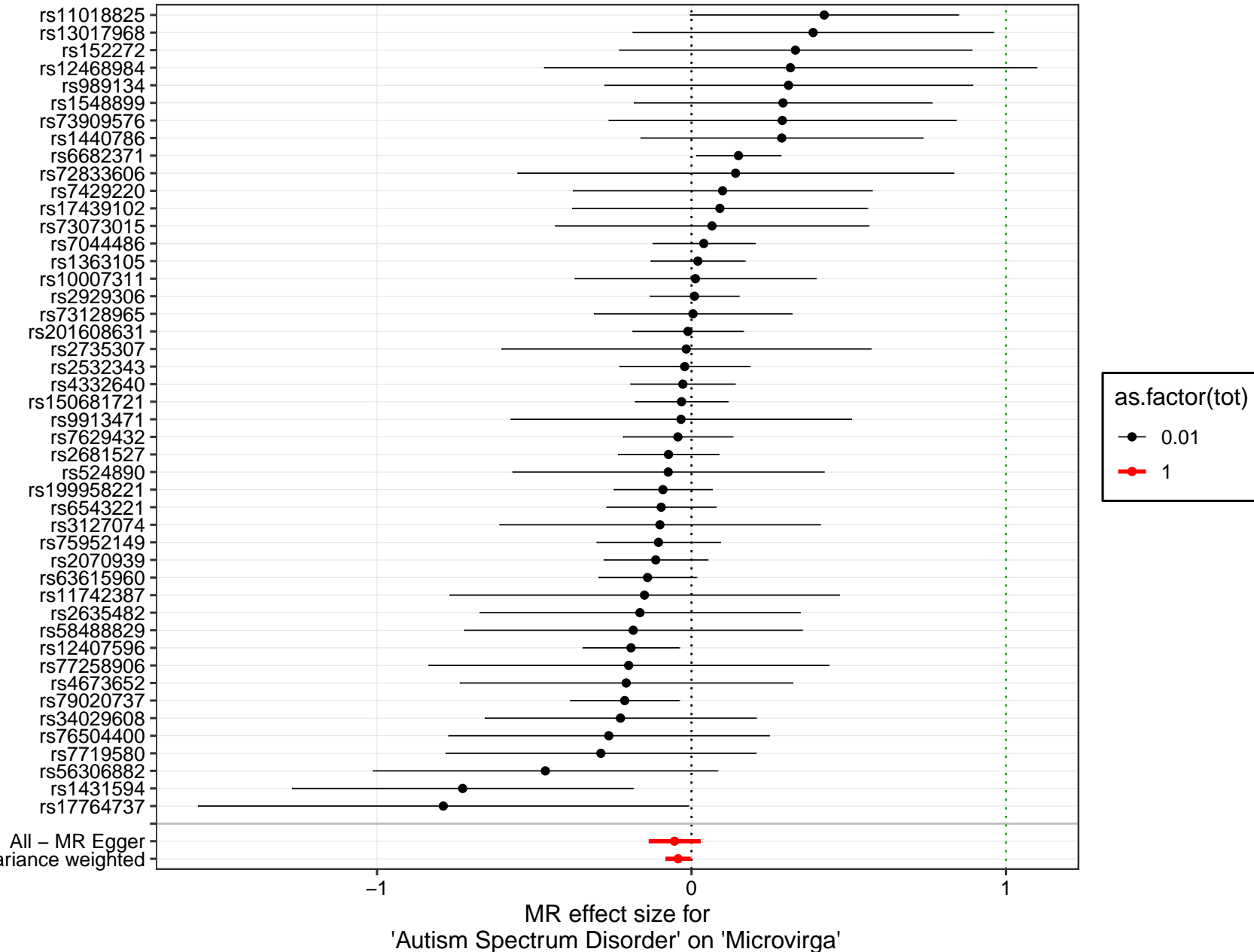

Supplement: Supplementary file 2 — Supplementary Material 2 [file 13568_2025_1969_MOESM2_ESM.zip › Revised supplementary materials/6 Inverse MR analysis results/plot/forest_or_Microvirga.pdf]

# Forest Plot (OR): Phascolarctobacterium sp003150755

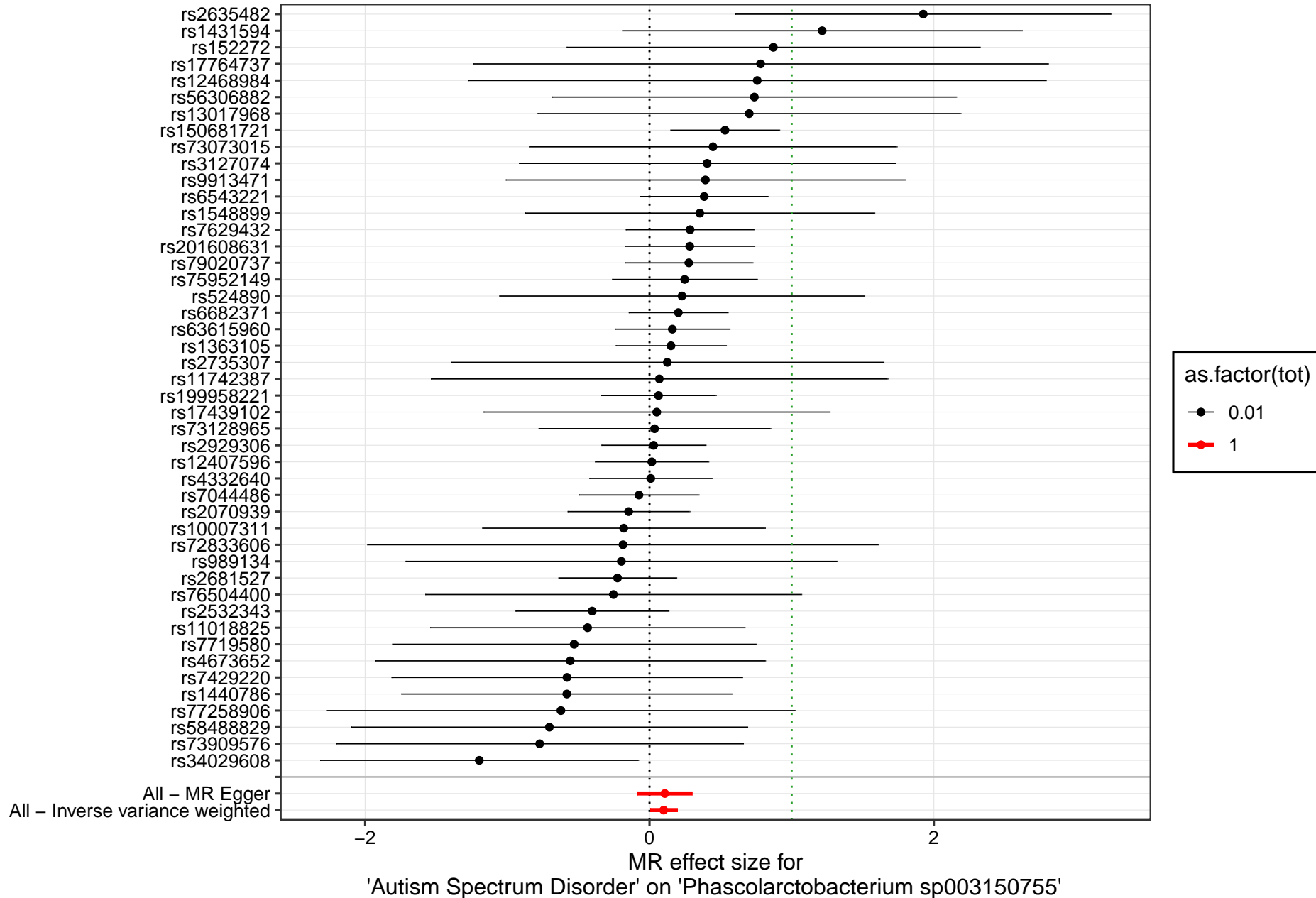

Supplement: Supplementary file 2 — Supplementary Material 2 [file 13568_2025_1969_MOESM2_ESM.zip › Revised supplementary materials/6 Inverse MR analysis results/plot/forest_or_Phascolarctobacterium sp003150755.pdf]

# Forest Plot (OR): RUG472

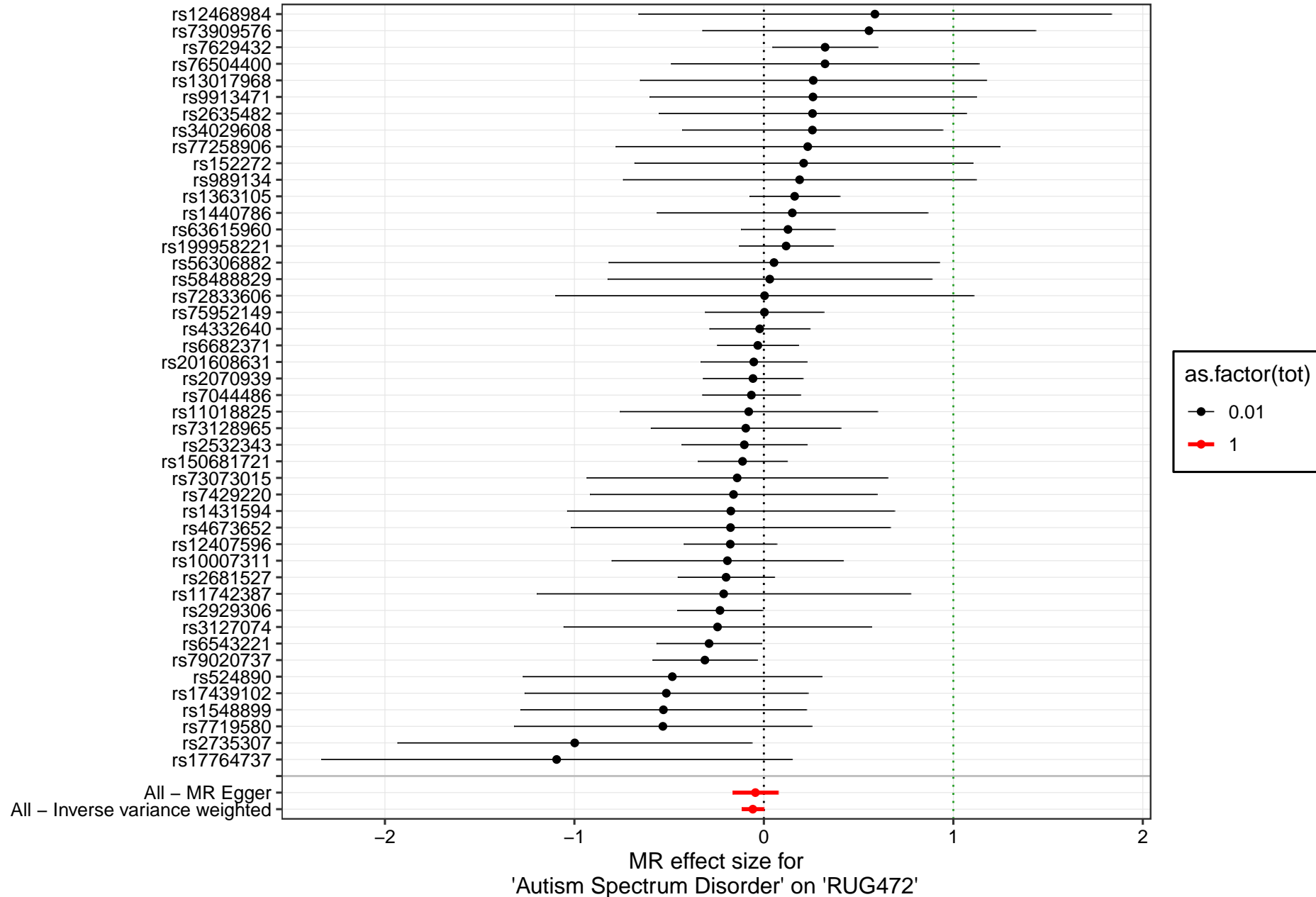

Supplement: Supplementary file 2 — Supplementary Material 2 [file 13568_2025_1969_MOESM2_ESM.zip › Revised supplementary materials/6 Inverse MR analysis results/plot/forest_or_RUG472.pdf]

# Forest Plot (OR): Spirillospora

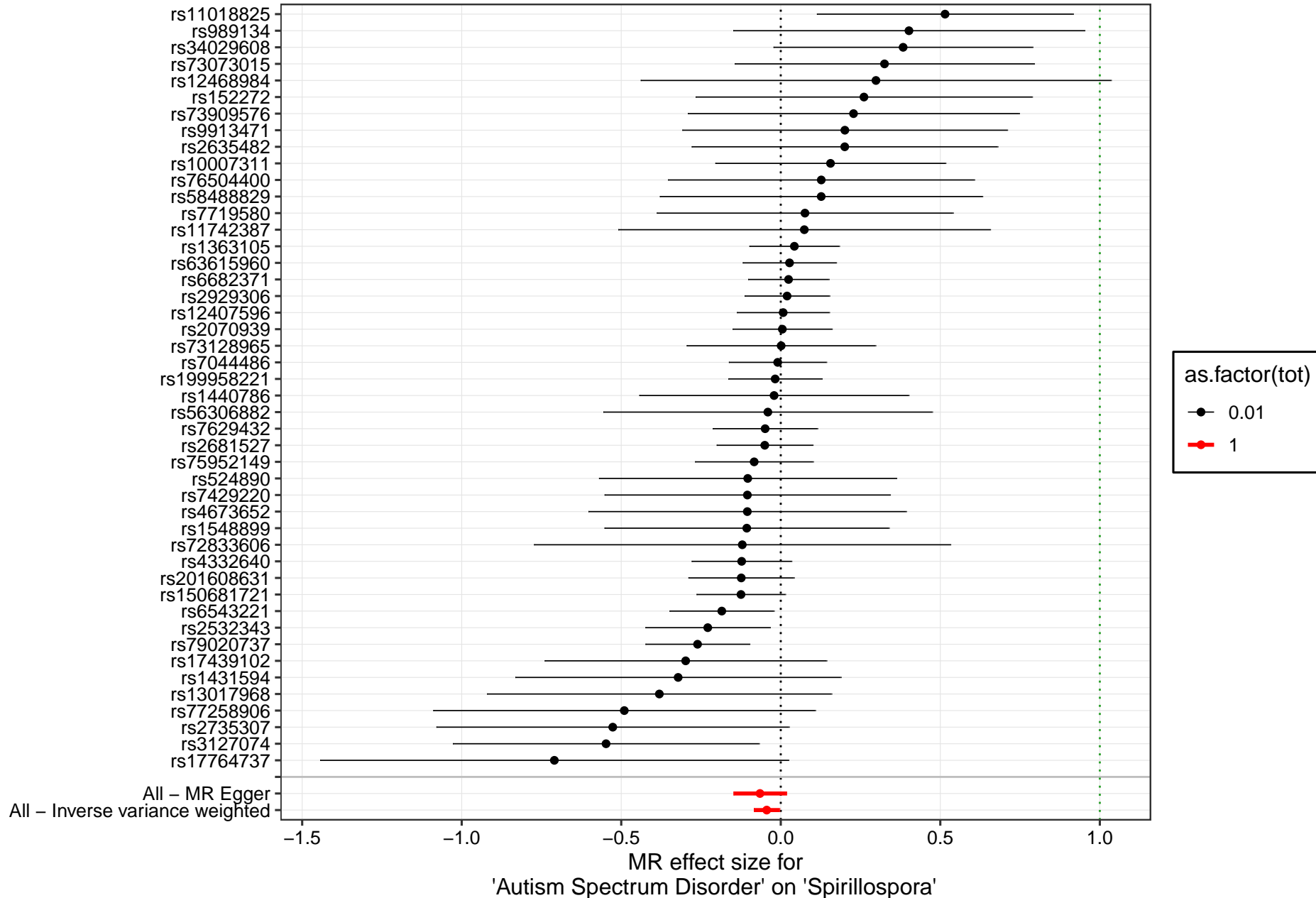

Supplement: Supplementary file 2 — Supplementary Material 2 [file 13568_2025_1969_MOESM2_ESM.zip › Revised supplementary materials/6 Inverse MR analysis results/plot/forest_or_Spirillospora.pdf]

# Forest Plot (OR): UBA1777 sp900316255

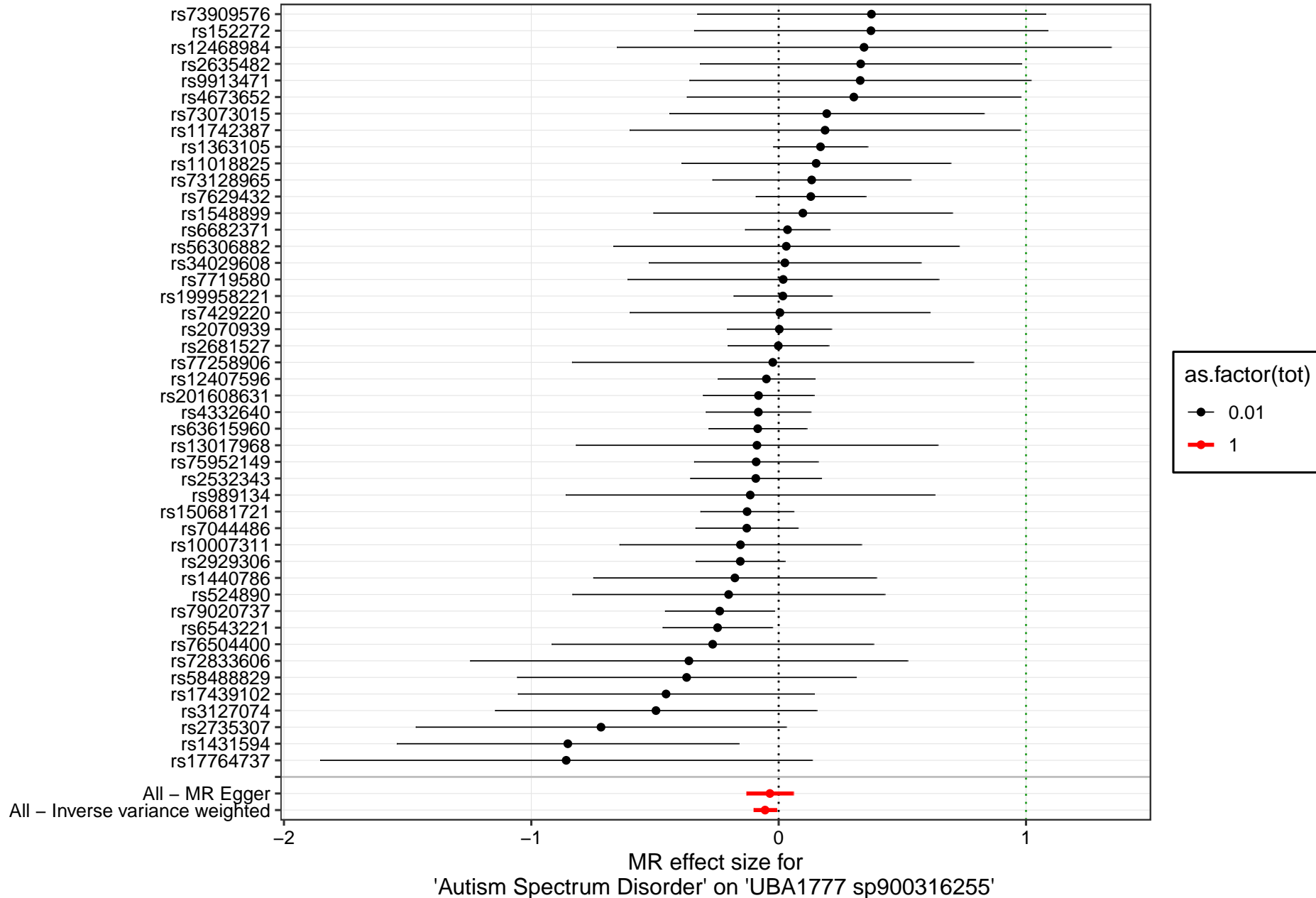

Supplement: Supplementary file 2 — Supplementary Material 2 [file 13568_2025_1969_MOESM2_ESM.zip › Revised supplementary materials/6 Inverse MR analysis results/plot/forest_or_UBA1777 sp900316255.pdf]

# Forest Plot (OR): UBA7703

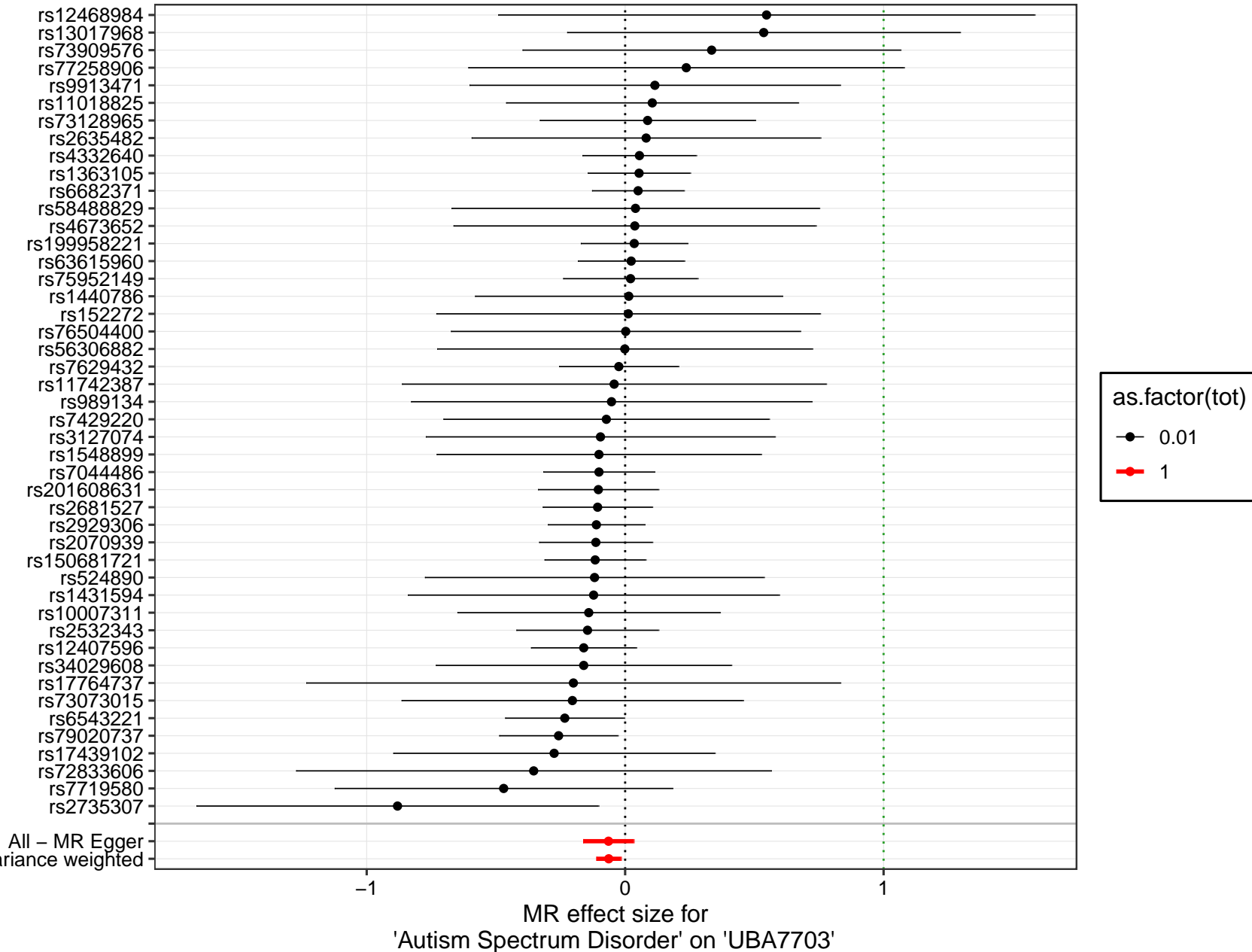

Supplement: Supplementary file 2 — Supplementary Material 2 [file 13568_2025_1969_MOESM2_ESM.zip › Revised supplementary materials/6 Inverse MR analysis results/plot/forest_or_UBA7703.pdf]

**Funnel Plot (OR): *Alistipes shahii***

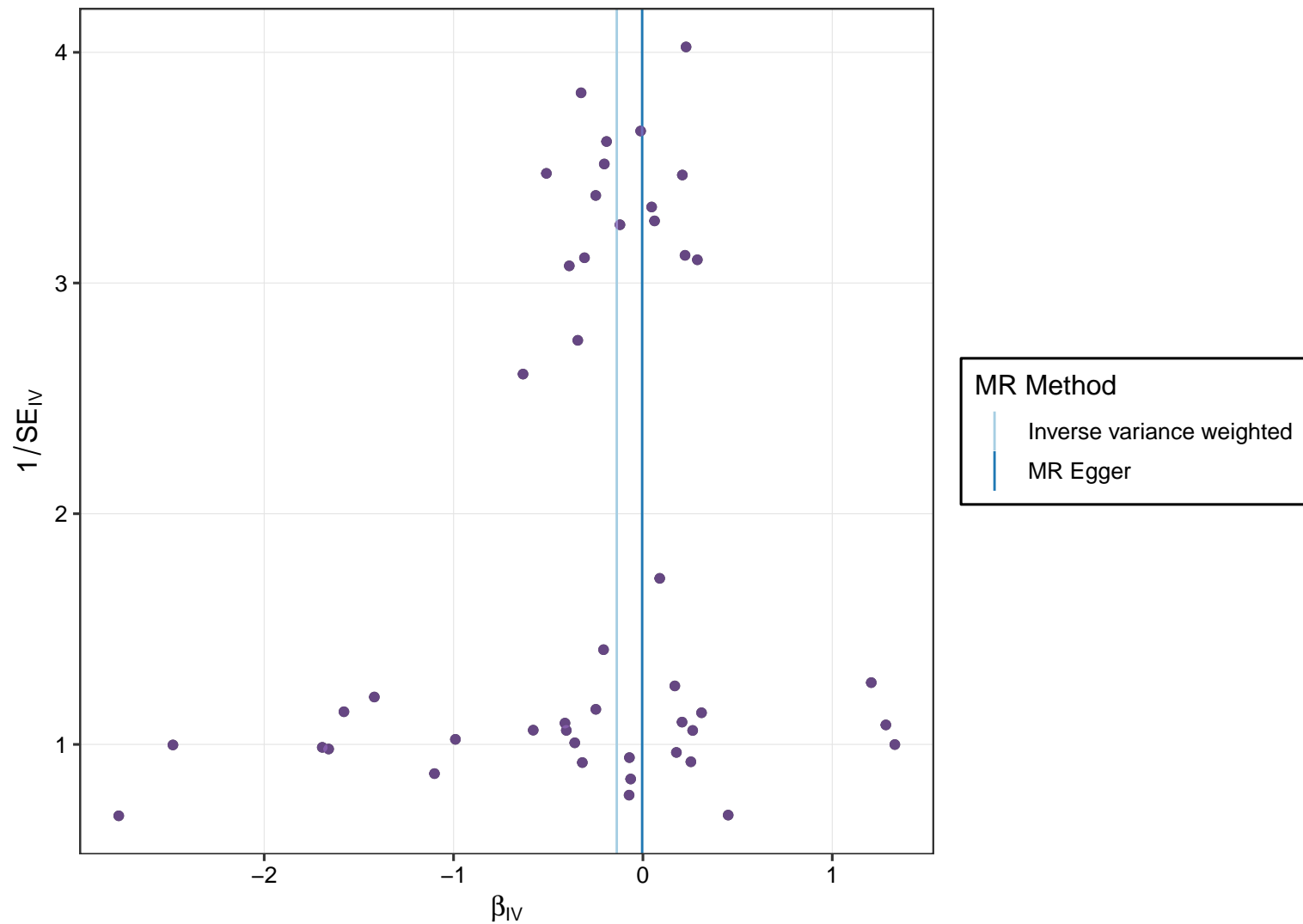

Supplement: Supplementary file 2 — Supplementary Material 2 [file 13568_2025_1969_MOESM2_ESM.zip › Revised supplementary materials/6 Inverse MR analysis results/plot/funnel_or_Alistipes shahii.pdf]

Funnel Plot (OR): An181

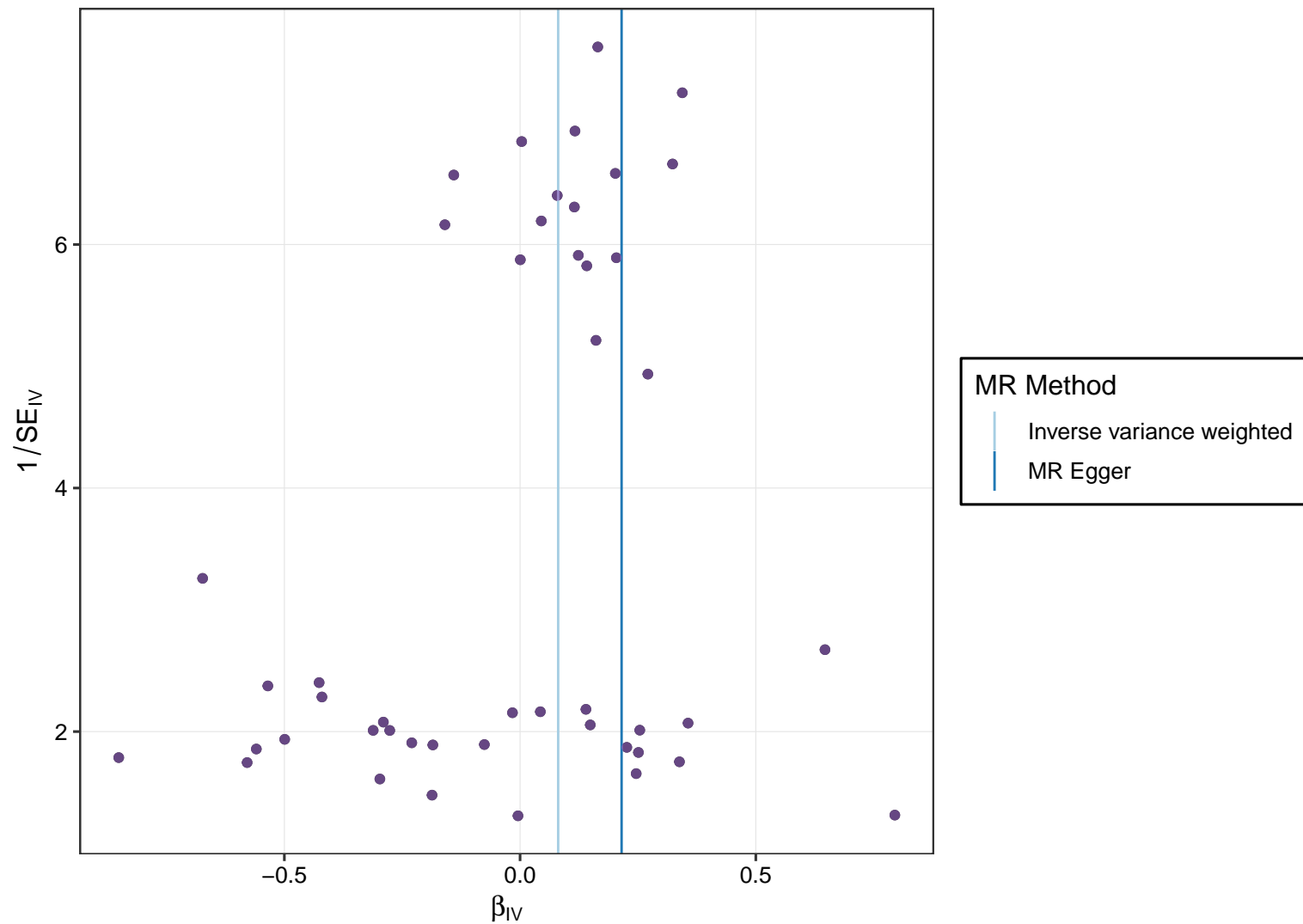

Supplement: Supplementary file 2 — Supplementary Material 2 [file 13568_2025_1969_MOESM2_ESM.zip › Revised supplementary materials/6 Inverse MR analysis results/plot/funnel_or_An181.pdf]

**Funnel Plot (OR): Aureimonas**

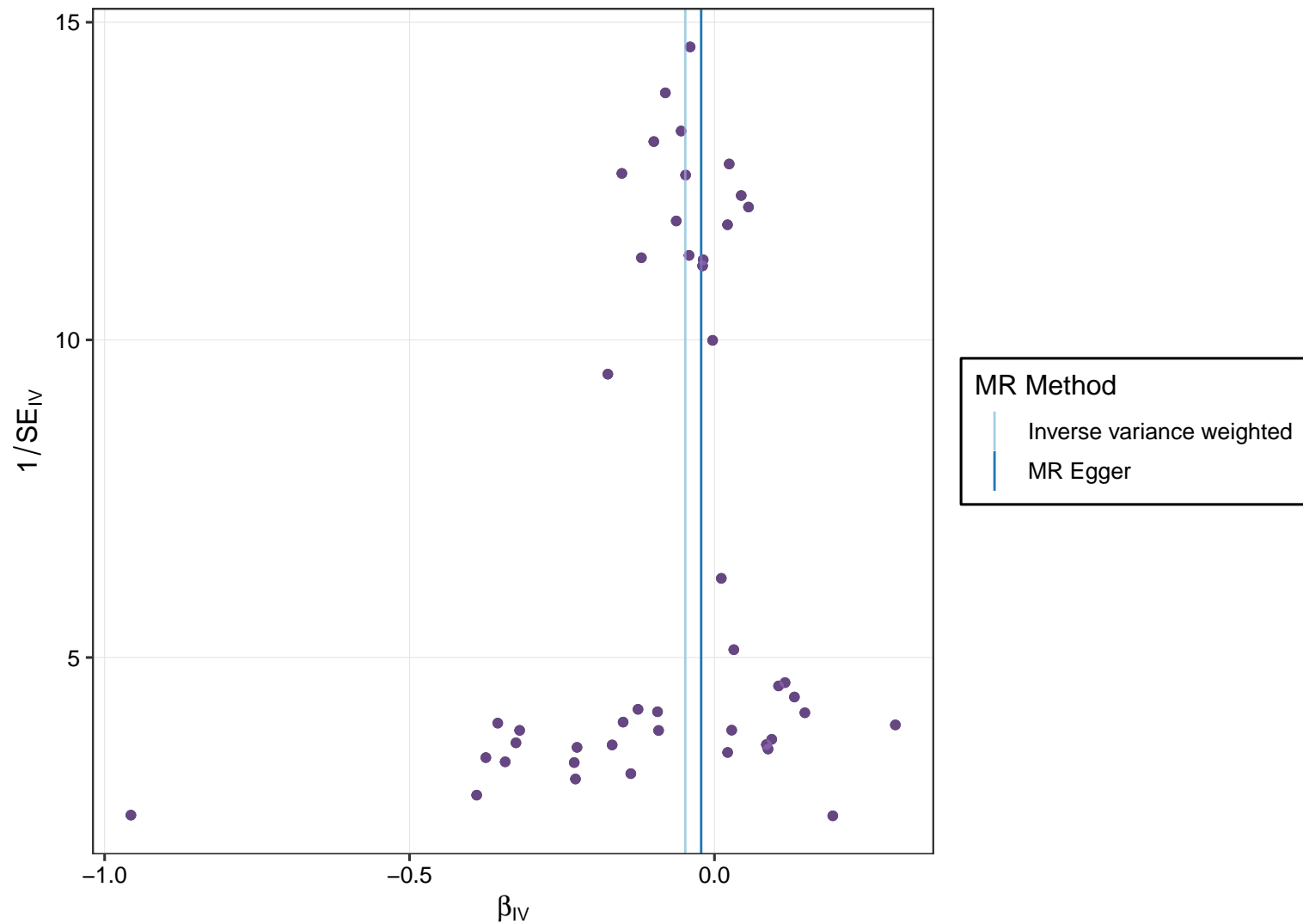

Supplement: Supplementary file 2 — Supplementary Material 2 [file 13568_2025_1969_MOESM2_ESM.zip › Revised supplementary materials/6 Inverse MR analysis results/plot/funnel_or_Aureimonas.pdf]

# Funnel Plot (OR): Blautia A sp002159835

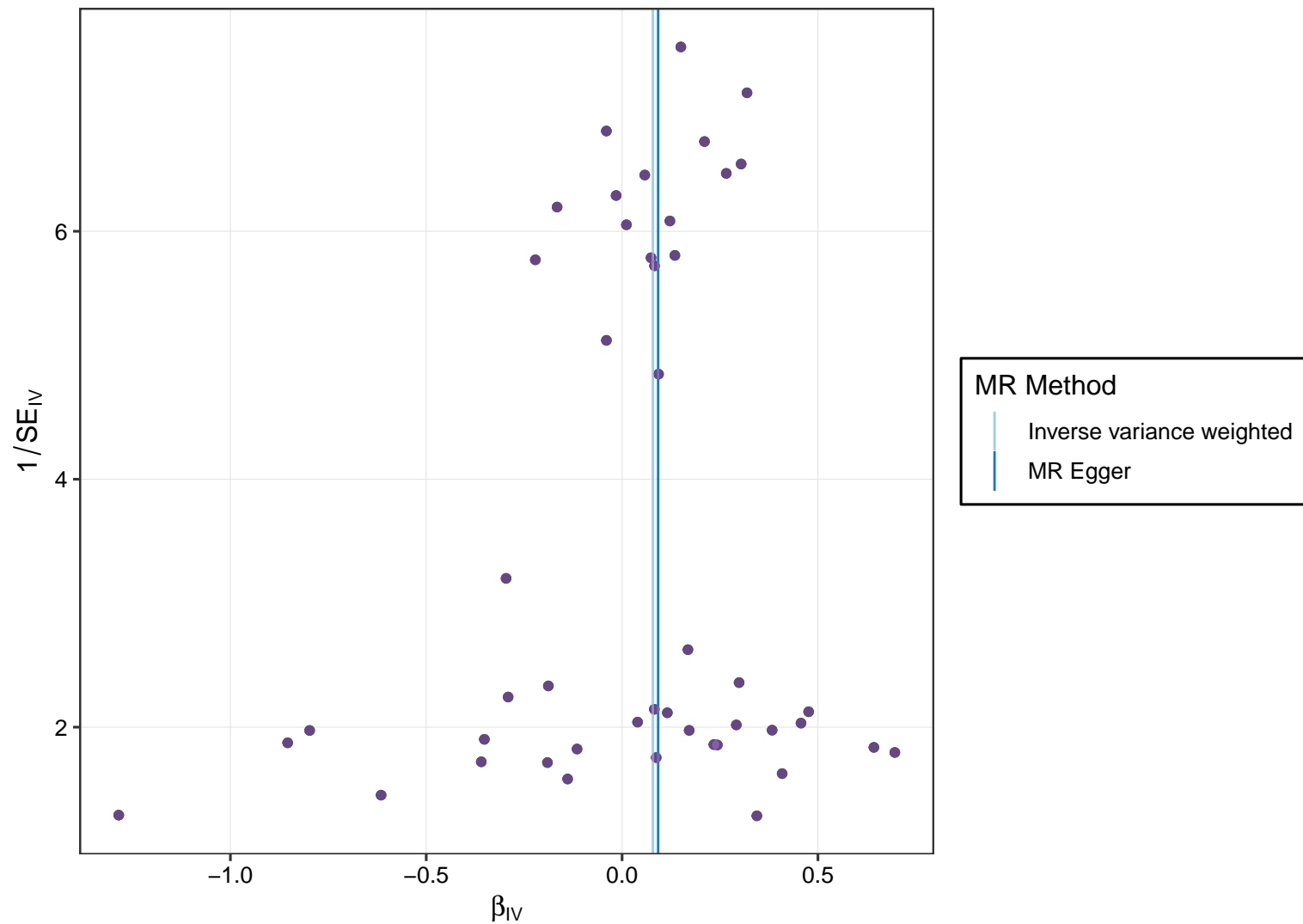

Supplement: Supplementary file 2 — Supplementary Material 2 [file 13568_2025_1969_MOESM2_ESM.zip › Revised supplementary materials/6 Inverse MR analysis results/plot/funnel_or_Blautia A sp002159835.pdf]

**Funnel Plot (OR): CAG-302**

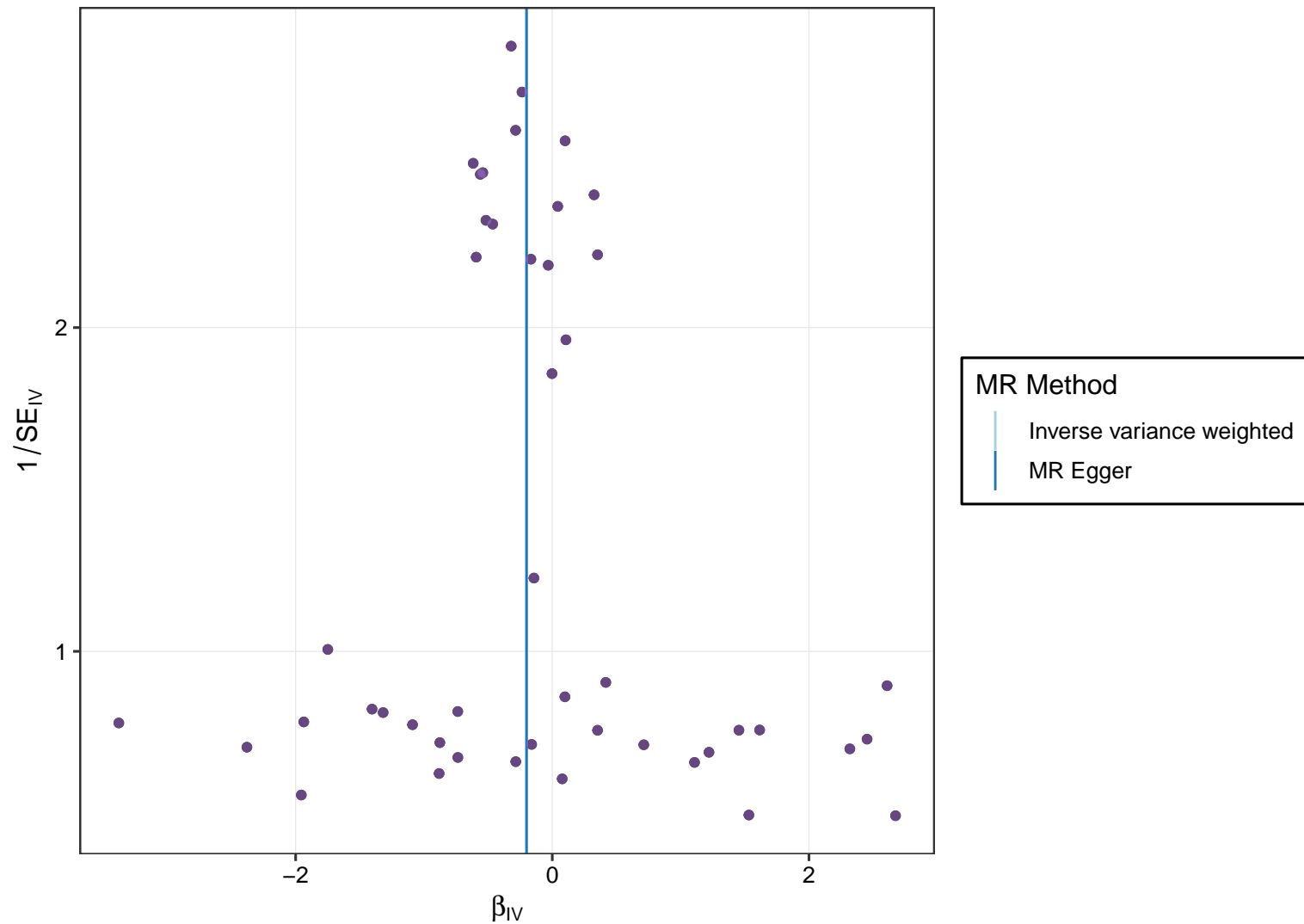

Supplement: Supplementary file 2 — Supplementary Material 2 [file 13568_2025_1969_MOESM2_ESM.zip › Revised supplementary materials/6 Inverse MR analysis results/plot/funnel_or_CAG-302.pdf]

**Funnel Plot (OR): CAG-485 sp002404675**

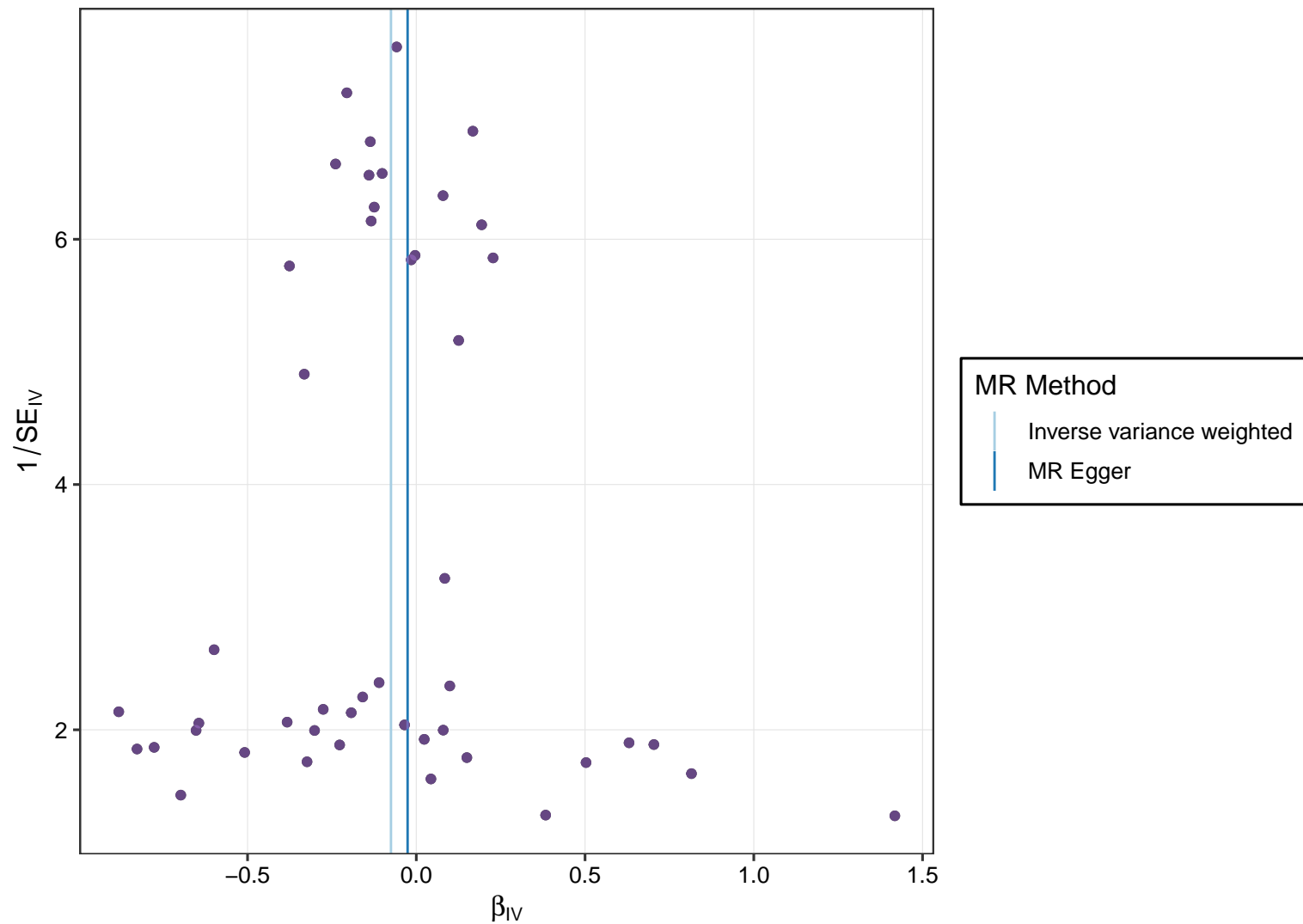

Supplement: Supplementary file 2 — Supplementary Material 2 [file 13568_2025_1969_MOESM2_ESM.zip › Revised supplementary materials/6 Inverse MR analysis results/plot/funnel_or_CAG-485 sp002404675.pdf]

**Funnel Plot (OR): CAG-83 sp002392625**

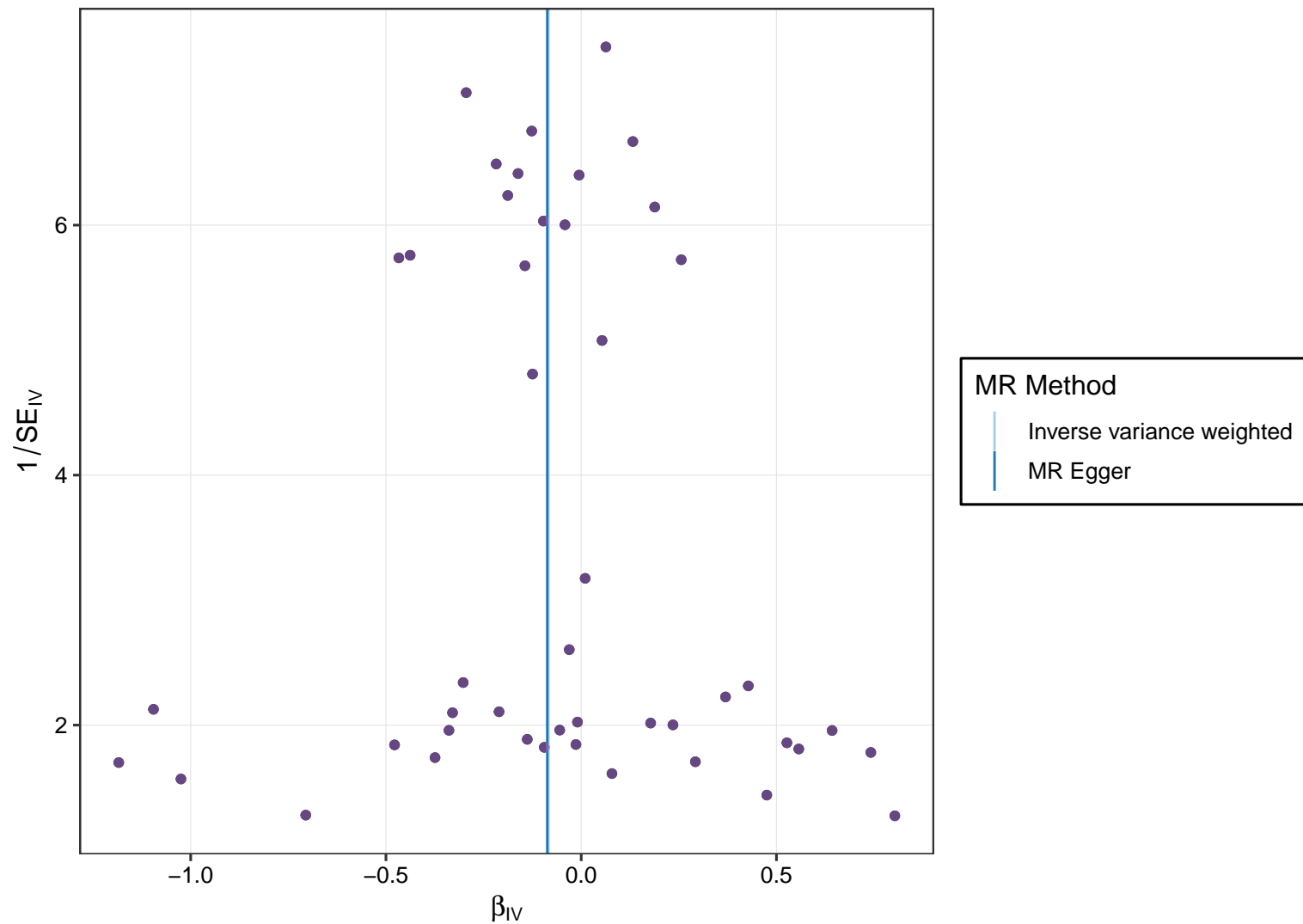

Supplement: Supplementary file 2 — Supplementary Material 2 [file 13568_2025_1969_MOESM2_ESM.zip › Revised supplementary materials/6 Inverse MR analysis results/plot/funnel_or_CAG-83 sp002392625.pdf]

**Funnel Plot (OR): Enterococcus A**

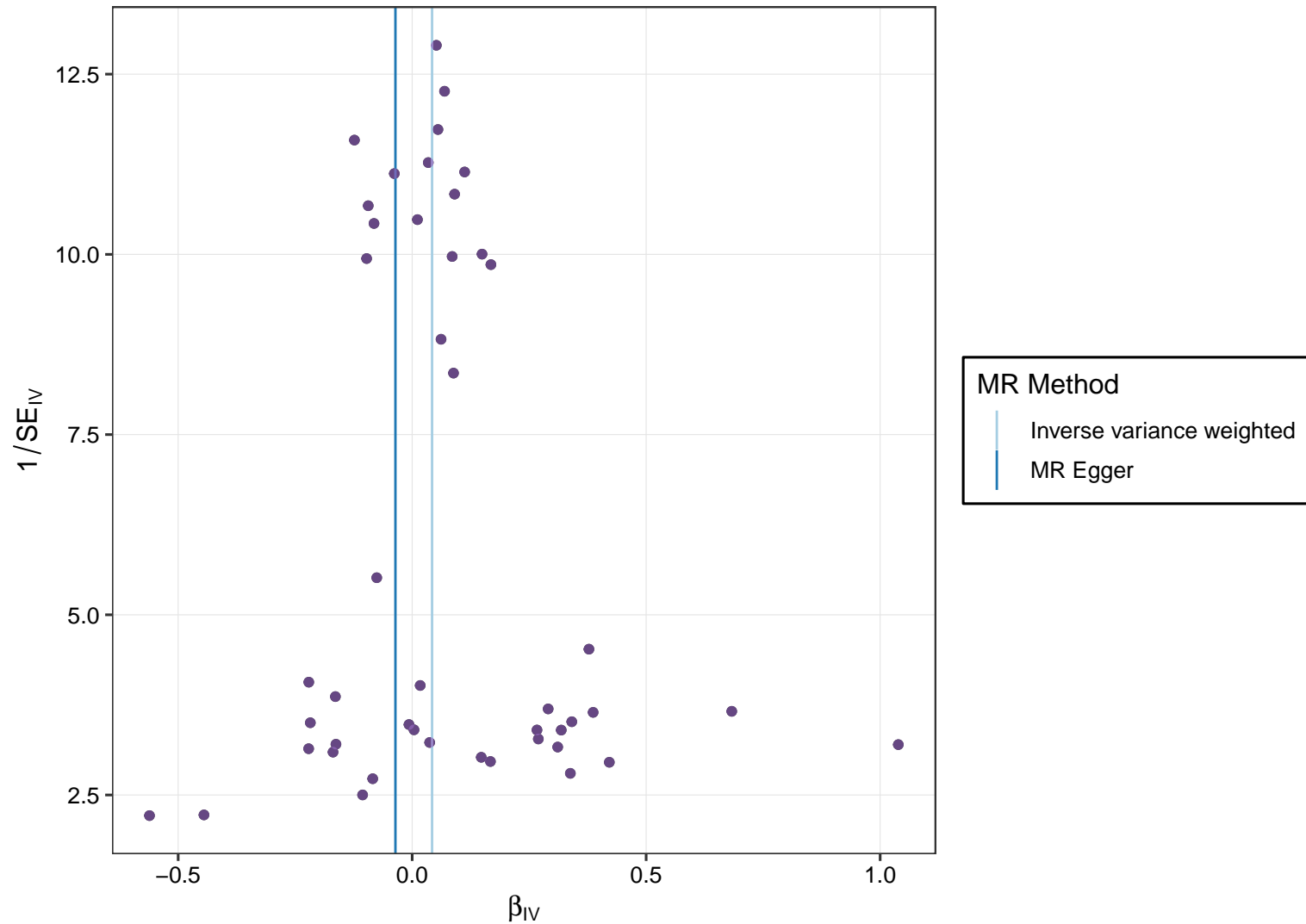

Supplement: Supplementary file 2 — Supplementary Material 2 [file 13568_2025_1969_MOESM2_ESM.zip › Revised supplementary materials/6 Inverse MR analysis results/plot/funnel_or_Enterococcus A.pdf]

# Funnel Plot (OR): Enterococcus B

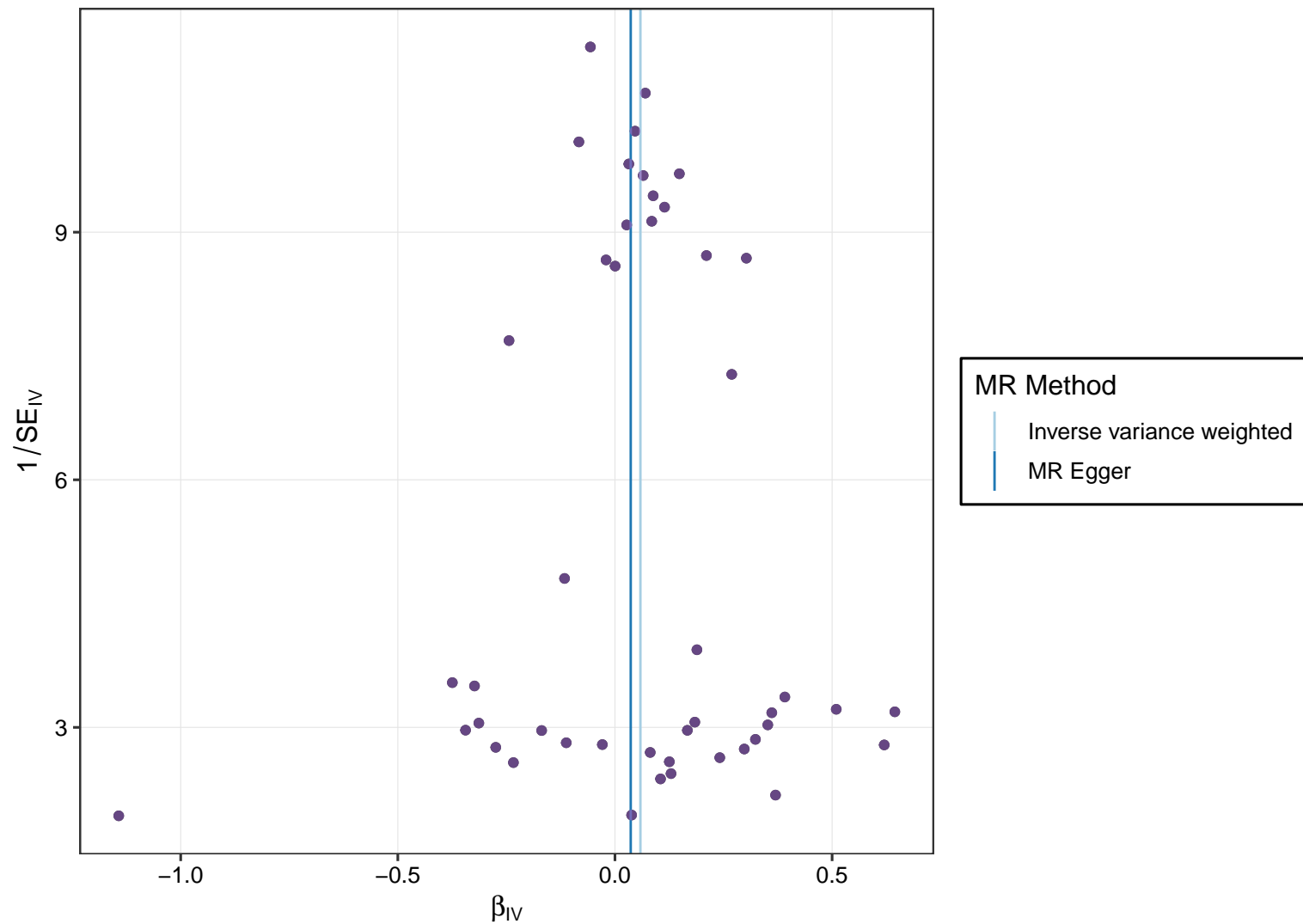

Supplement: Supplementary file 2 — Supplementary Material 2 [file 13568_2025_1969_MOESM2_ESM.zip › Revised supplementary materials/6 Inverse MR analysis results/plot/funnel_or_Enterococcus B.pdf]

# Funnel Plot (OR): Faecalibacterium sp002160895

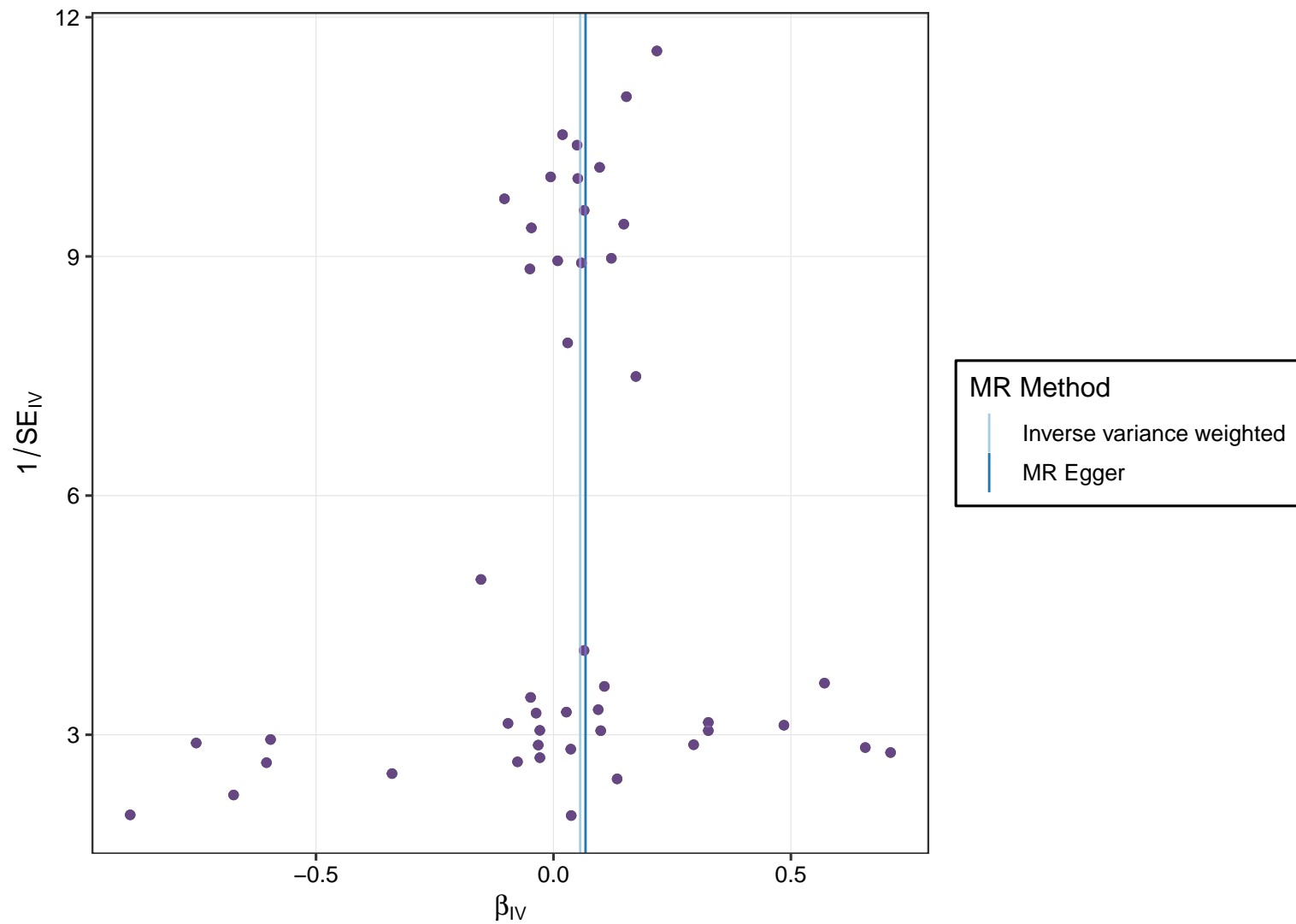

Supplement: Supplementary file 2 — Supplementary Material 2 [file 13568_2025_1969_MOESM2_ESM.zip › Revised supplementary materials/6 Inverse MR analysis results/plot/funnel_or_Faecalibacterium sp002160895.pdf]

**Funnel Plot (OR): Gemmatimonadaceae**

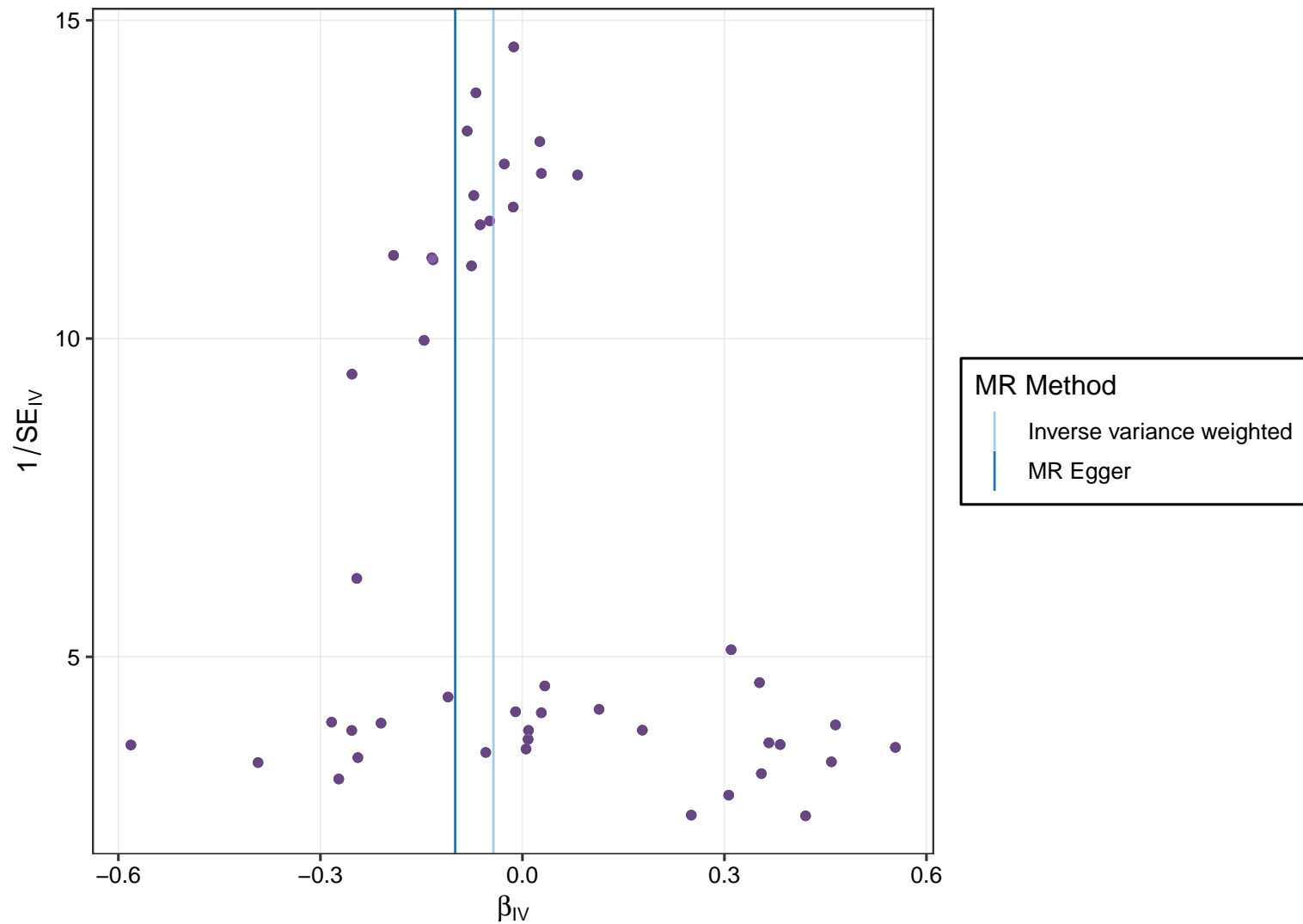

Supplement: Supplementary file 2 — Supplementary Material 2 [file 13568_2025_1969_MOESM2_ESM.zip › Revised supplementary materials/6 Inverse MR analysis results/plot/funnel_or_Gemmatimonadaceae.pdf]

### Funnel Plot (OR): Lactobacillus B

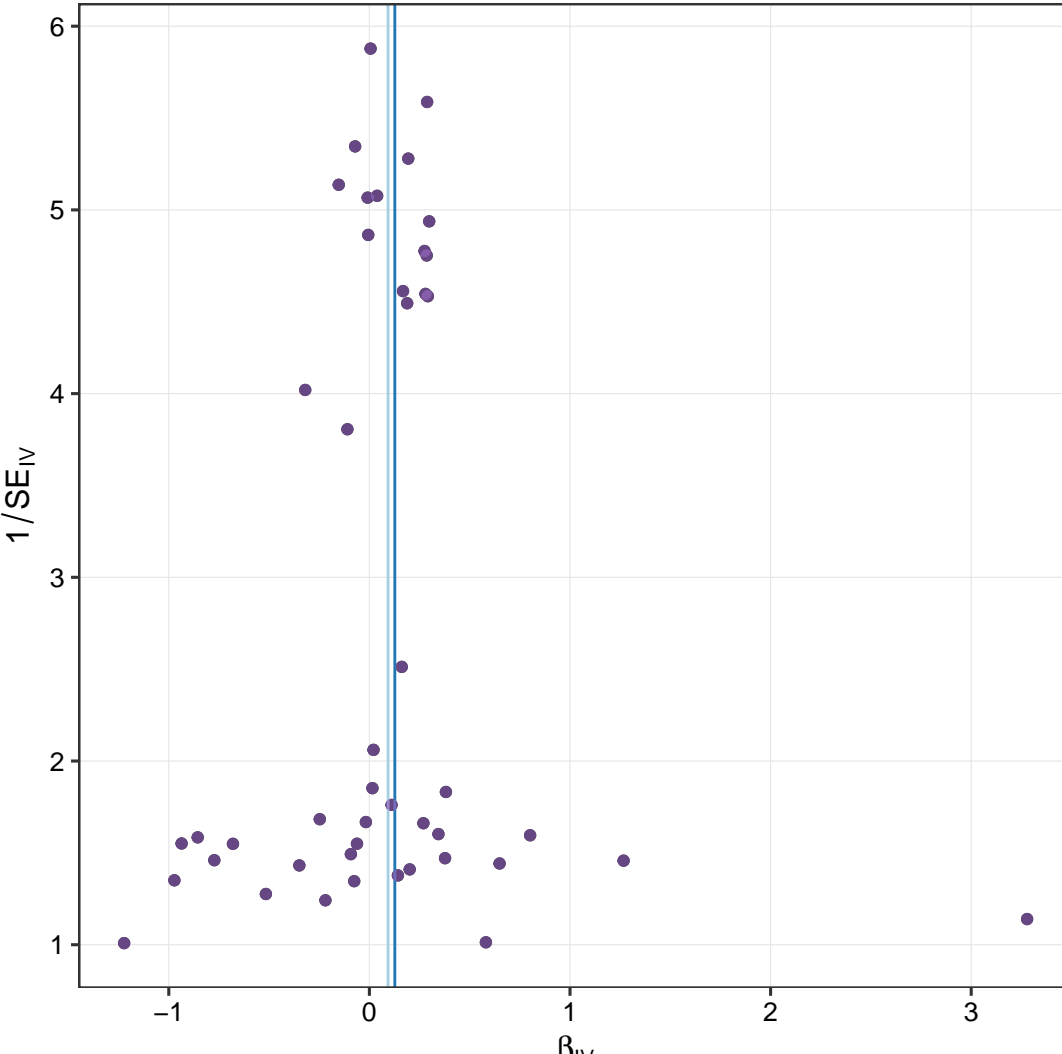

Supplement: Supplementary file 2 — Supplementary Material 2 [file 13568_2025_1969_MOESM2_ESM.zip › Revised supplementary materials/6 Inverse MR analysis results/plot/funnel_or_Lactobacillus B.pdf]

**Funnel Plot (OR): Microvirga**

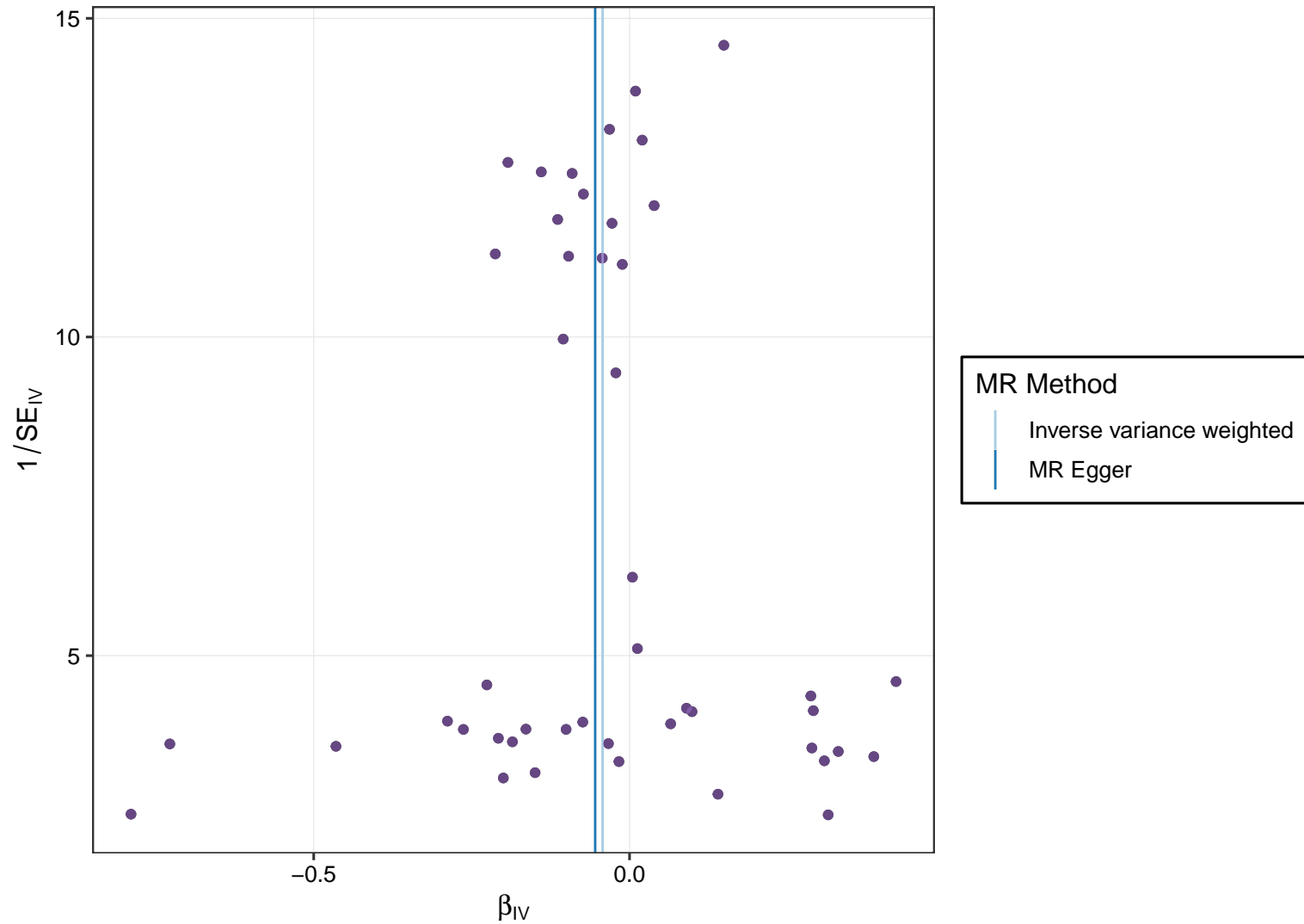

Supplement: Supplementary file 2 — Supplementary Material 2 [file 13568_2025_1969_MOESM2_ESM.zip › Revised supplementary materials/6 Inverse MR analysis results/plot/funnel_or_Microvirga.pdf]

# Funnel Plot (OR): *Phascolarctobacterium* sp003150755

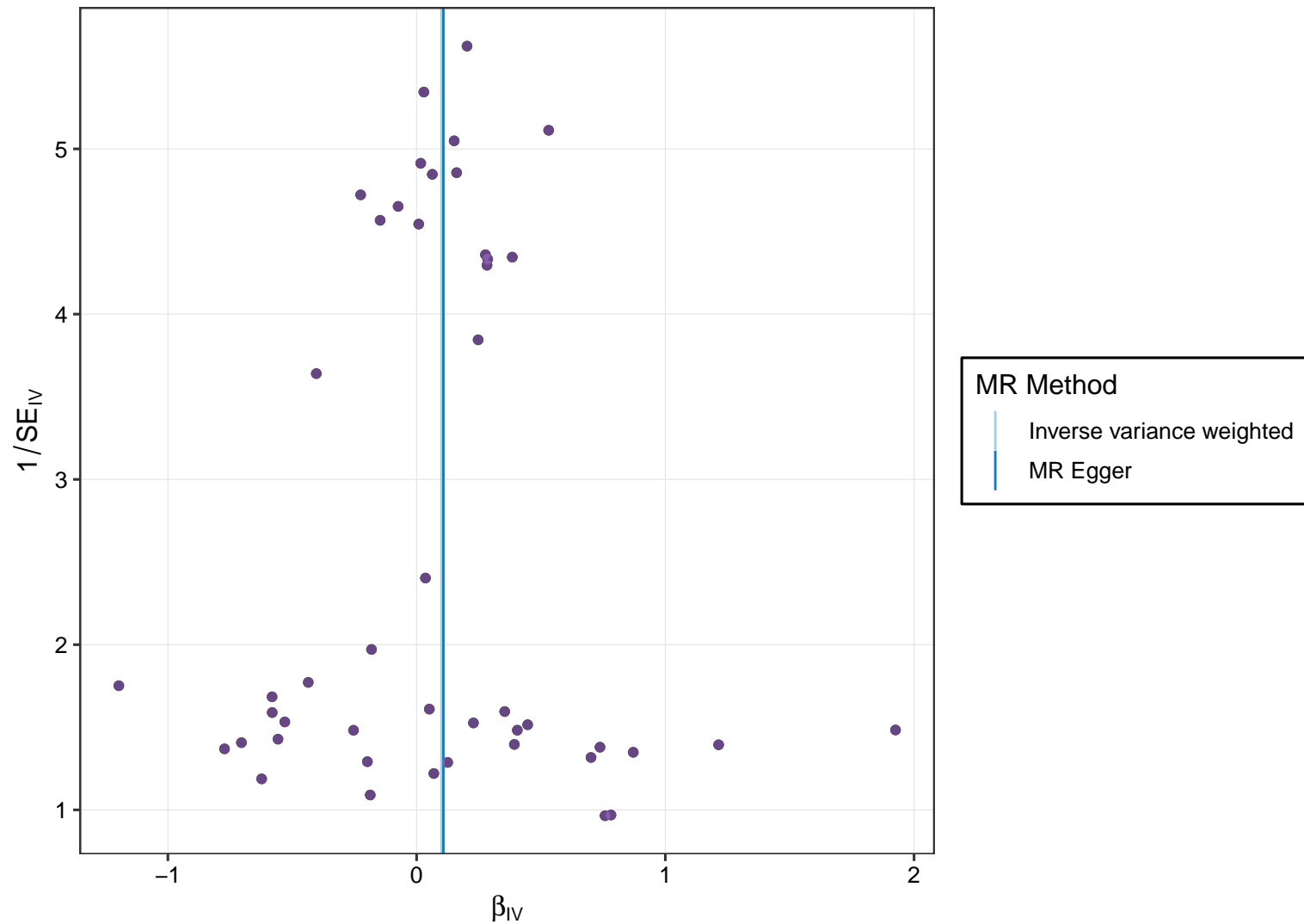

Supplement: Supplementary file 2 — Supplementary Material 2 [file 13568_2025_1969_MOESM2_ESM.zip › Revised supplementary materials/6 Inverse MR analysis results/plot/funnel_or_Phascolarctobacterium sp003150755.pdf]

## Funnel Plot (OR): RUG472

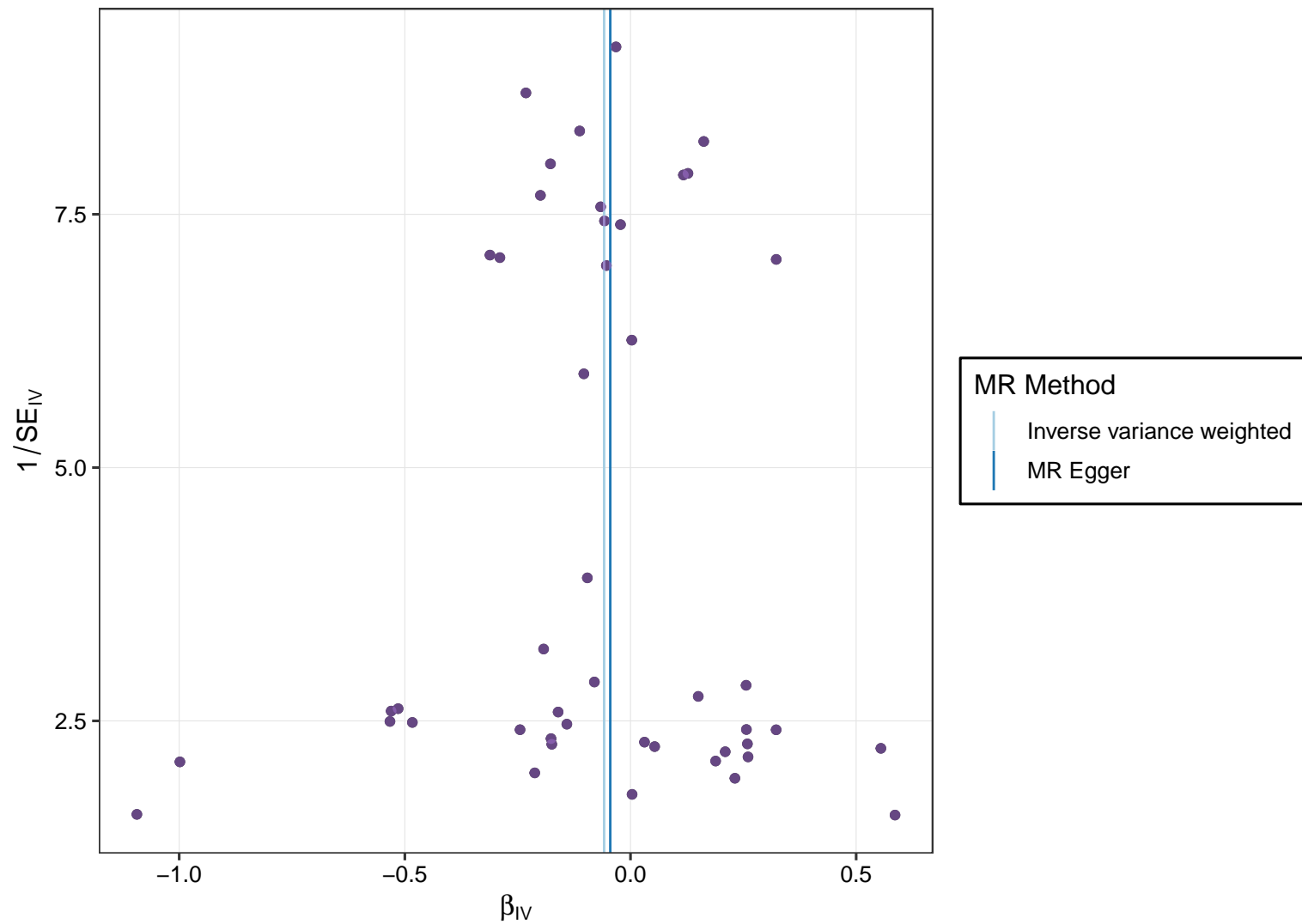

Supplement: Supplementary file 2 — Supplementary Material 2 [file 13568_2025_1969_MOESM2_ESM.zip › Revised supplementary materials/6 Inverse MR analysis results/plot/funnel_or_RUG472.pdf]

**Funnel Plot (OR): Spirillospora**

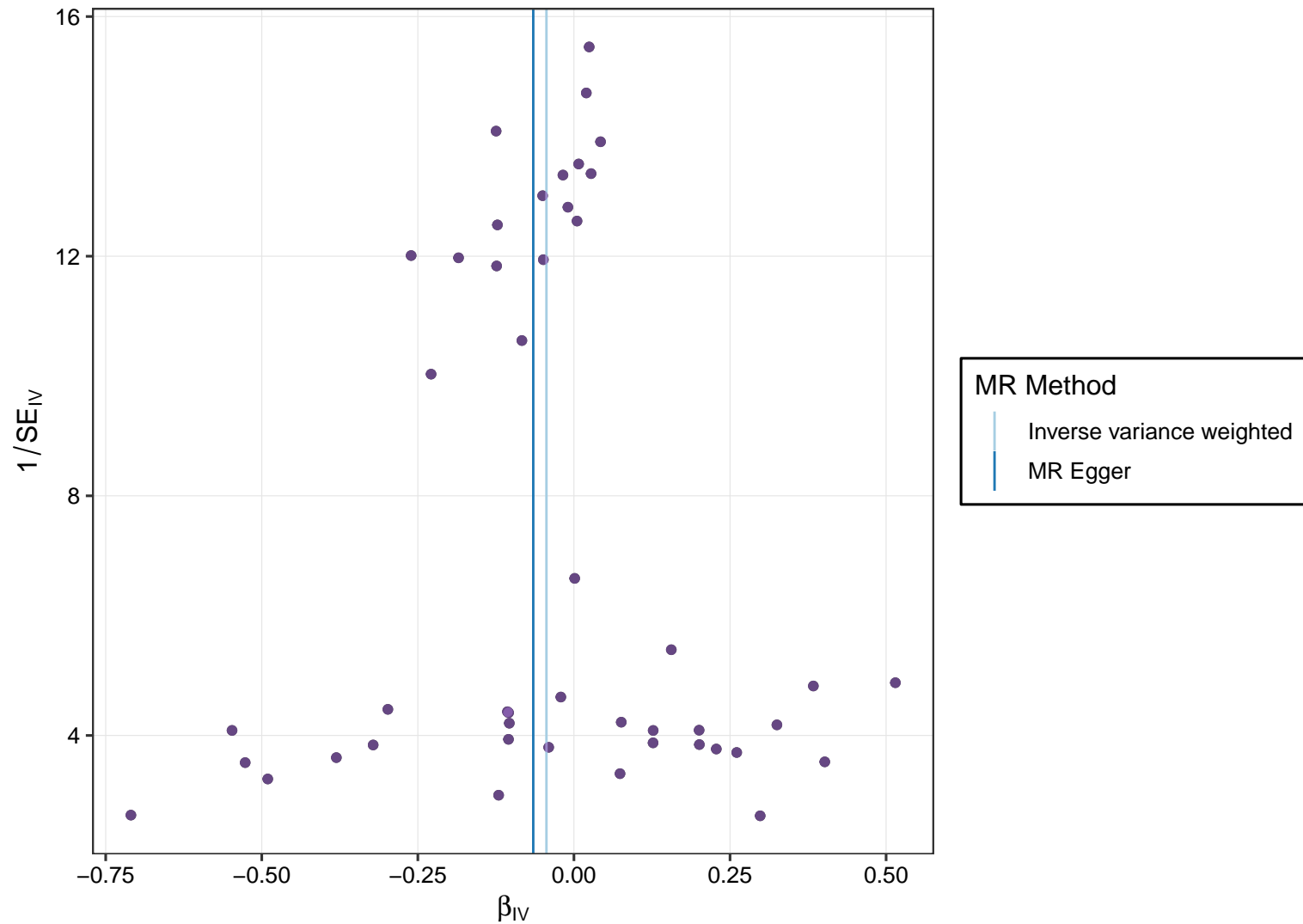

Supplement: Supplementary file 2 — Supplementary Material 2 [file 13568_2025_1969_MOESM2_ESM.zip › Revised supplementary materials/6 Inverse MR analysis results/plot/funnel_or_Spirillospora.pdf]

Funnel Plot (OR): UBA1777 sp900316255

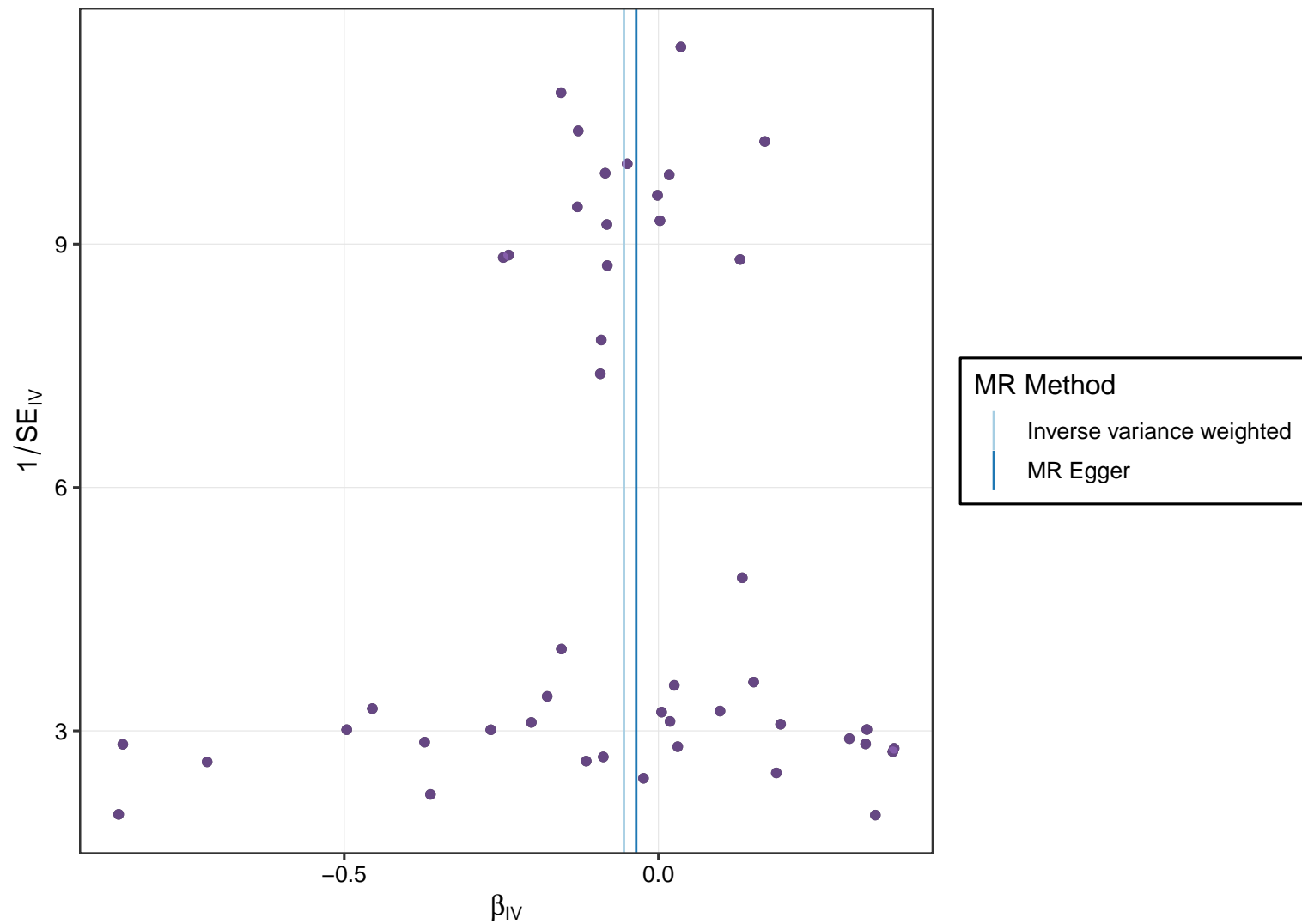

Supplement: Supplementary file 2 — Supplementary Material 2 [file 13568_2025_1969_MOESM2_ESM.zip › Revised supplementary materials/6 Inverse MR analysis results/plot/funnel_or_UBA1777 sp900316255.pdf]

# Funnel Plot (OR): UBA7703

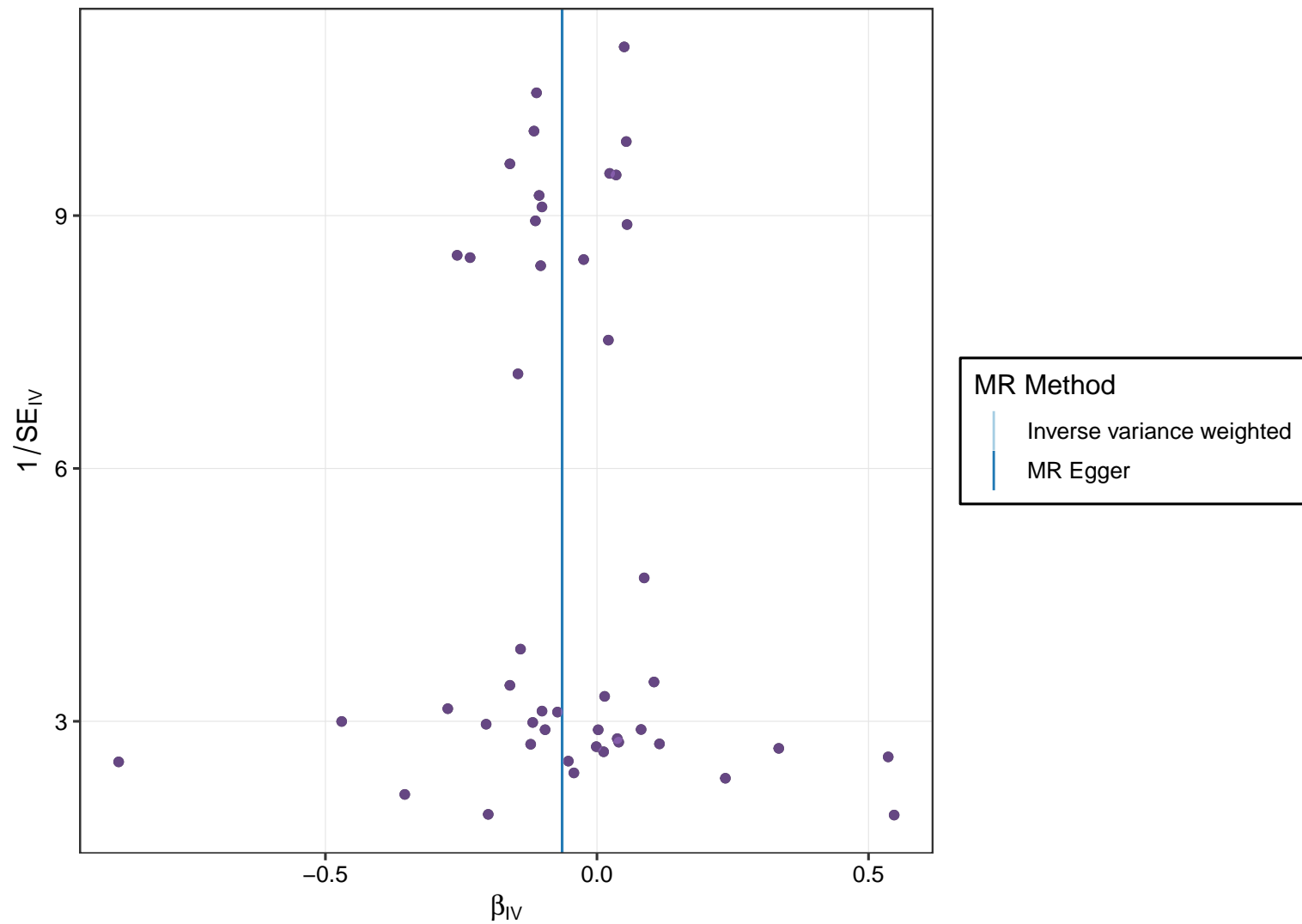

Supplement: Supplementary file 2 — Supplementary Material 2 [file 13568_2025_1969_MOESM2_ESM.zip › Revised supplementary materials/6 Inverse MR analysis results/plot/funnel_or_UBA7703.pdf]

# Leave-One-Out (Sorted): Alistipes shahii

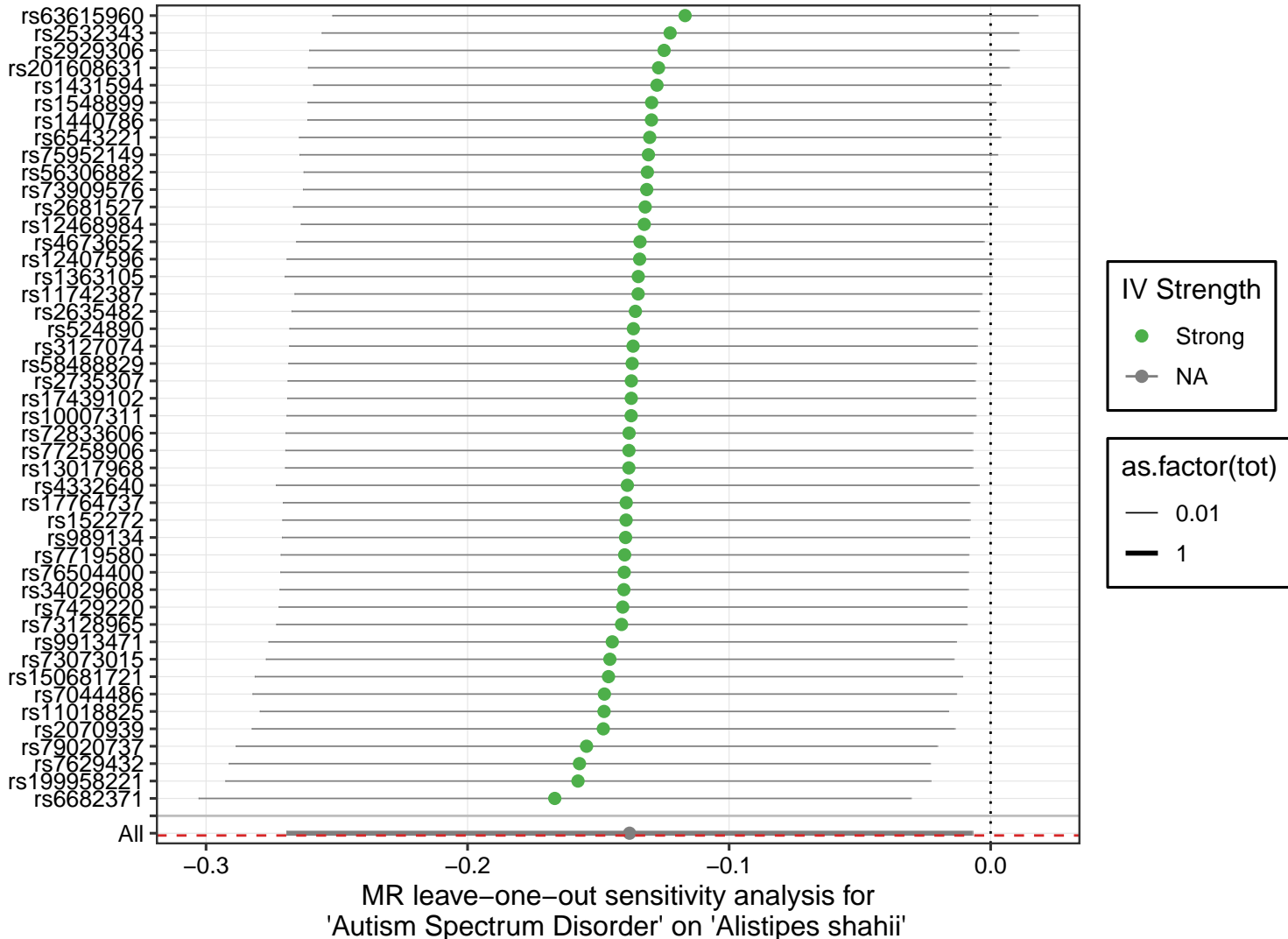

Supplement: Supplementary file 2 — Supplementary Material 2 [file 13568_2025_1969_MOESM2_ESM.zip › Revised supplementary materials/6 Inverse MR analysis results/plot/leaveoneout_or_Alistipes shahii.pdf]

# Leave-One-Out (Sorted): An181

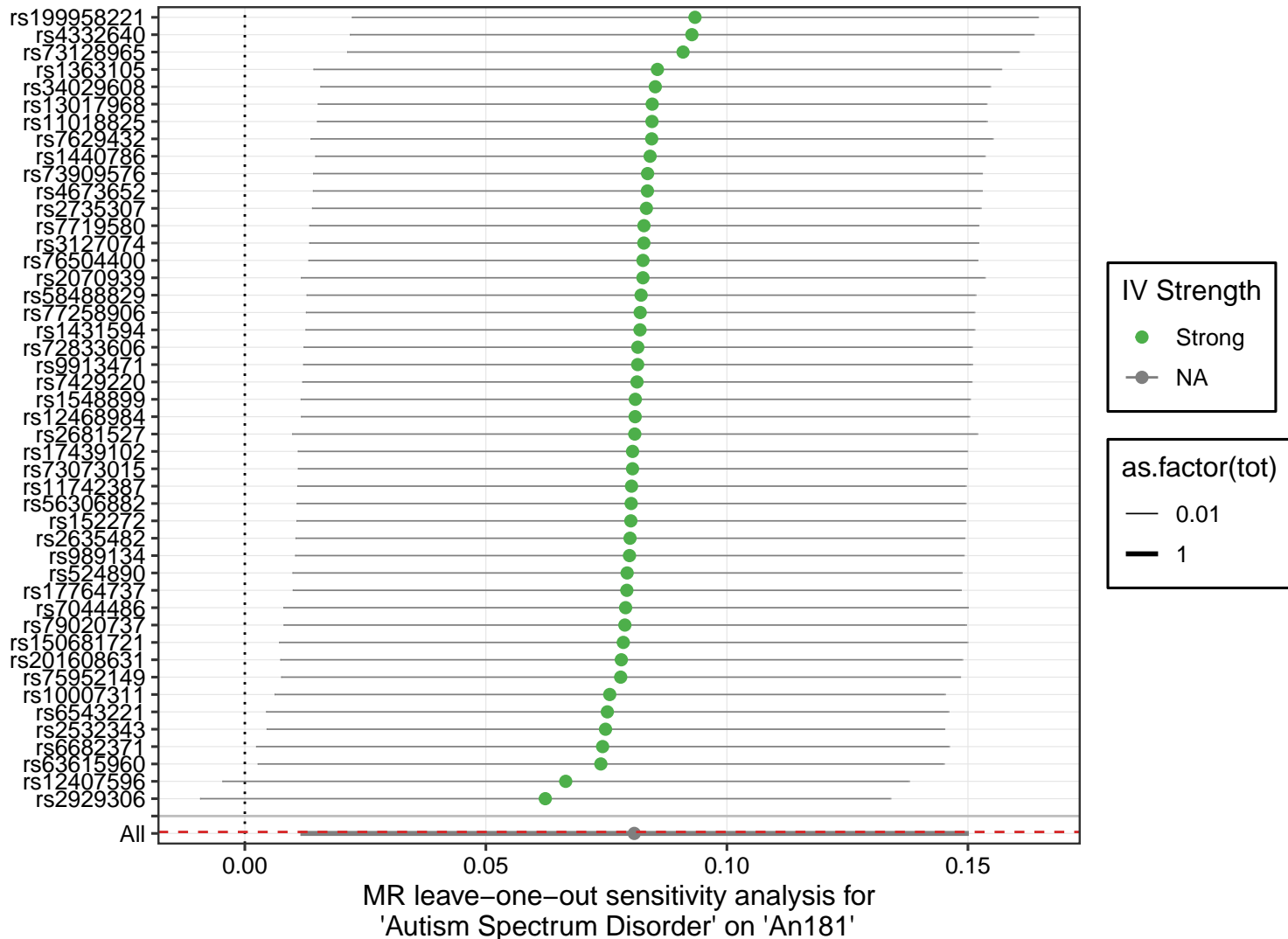

Supplement: Supplementary file 2 — Supplementary Material 2 [file 13568_2025_1969_MOESM2_ESM.zip › Revised supplementary materials/6 Inverse MR analysis results/plot/leaveoneout_or_An181.pdf]

# Leave-One-Out (Sorted): Aureimonas

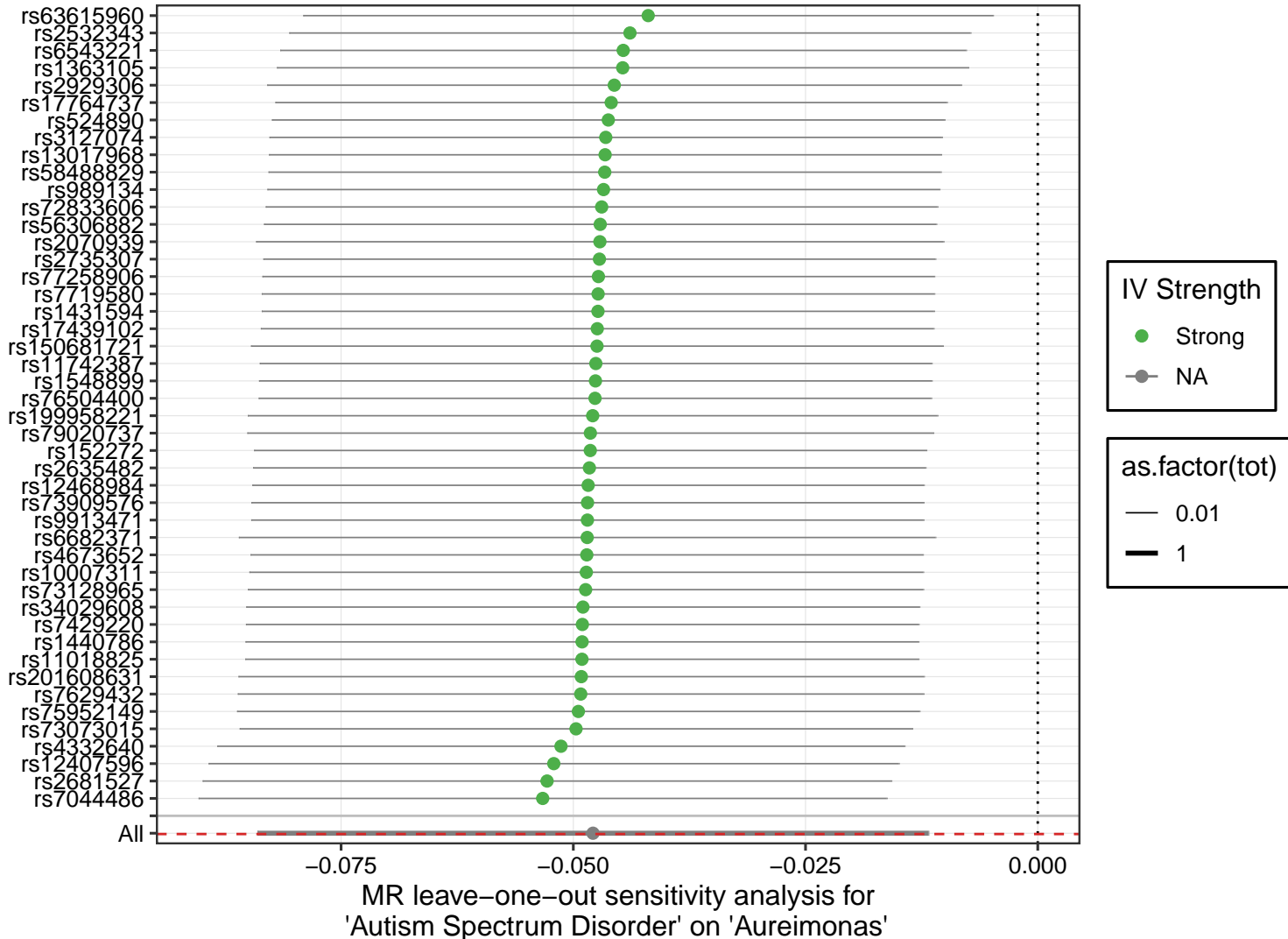

Supplement: Supplementary file 2 — Supplementary Material 2 [file 13568_2025_1969_MOESM2_ESM.zip › Revised supplementary materials/6 Inverse MR analysis results/plot/leaveoneout_or_Aureimonas.pdf]

# Leave-One-Out (Sorted): Blautia A sp002159835

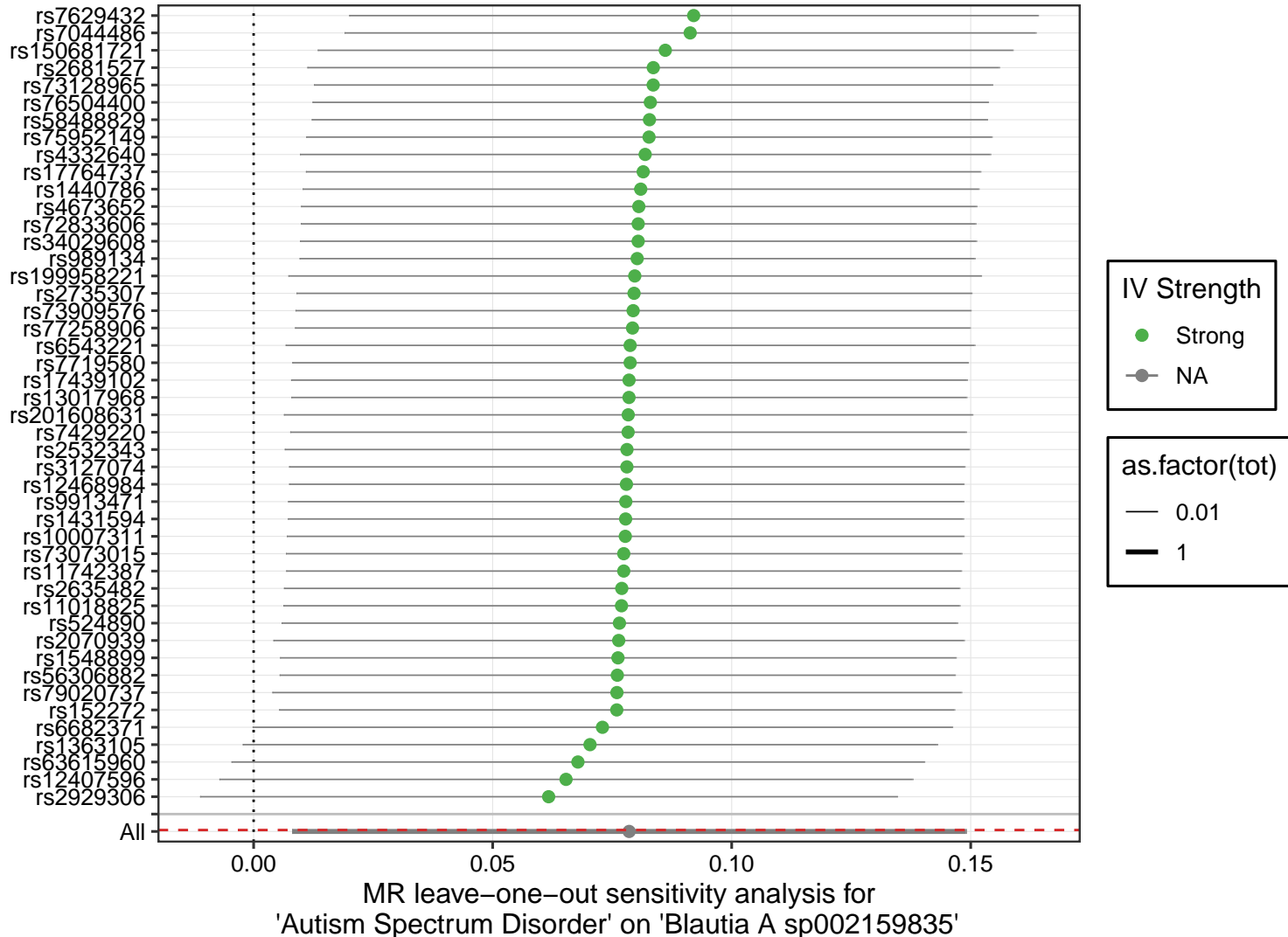

Supplement: Supplementary file 2 — Supplementary Material 2 [file 13568_2025_1969_MOESM2_ESM.zip › Revised supplementary materials/6 Inverse MR analysis results/plot/leaveoneout_or_Blautia A sp002159835.pdf]

# Leave-One-Out (Sorted): CAG-302

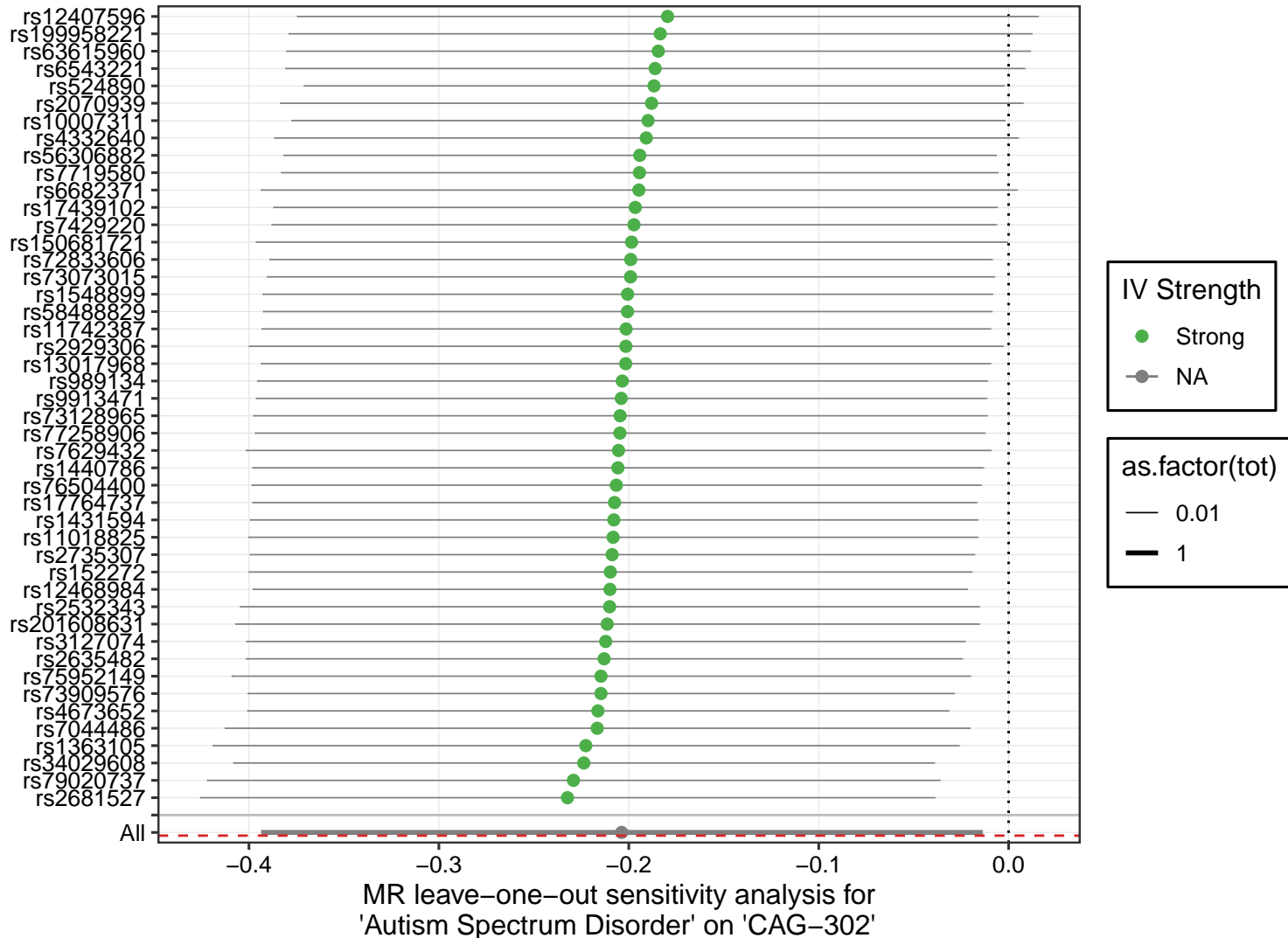

Supplement: Supplementary file 2 — Supplementary Material 2 [file 13568_2025_1969_MOESM2_ESM.zip › Revised supplementary materials/6 Inverse MR analysis results/plot/leaveoneout_or_CAG-302.pdf]

# Leave-One-Out (Sorted): CAG-485 sp002404675

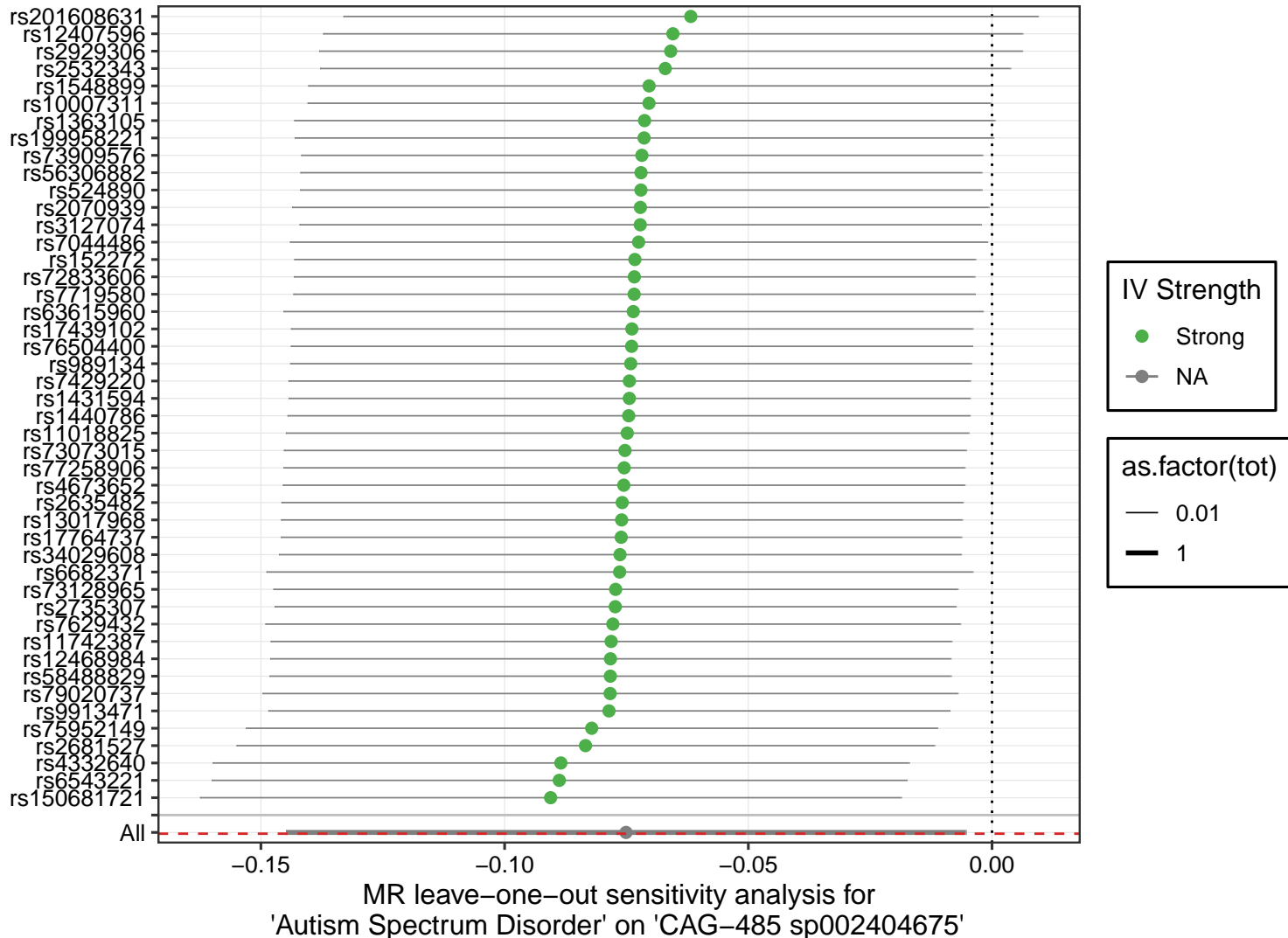

Supplement: Supplementary file 2 — Supplementary Material 2 [file 13568_2025_1969_MOESM2_ESM.zip › Revised supplementary materials/6 Inverse MR analysis results/plot/leaveoneout_or_CAG-485 sp002404675.pdf]

# Leave-One-Out (Sorted): CAG-83 sp002392625

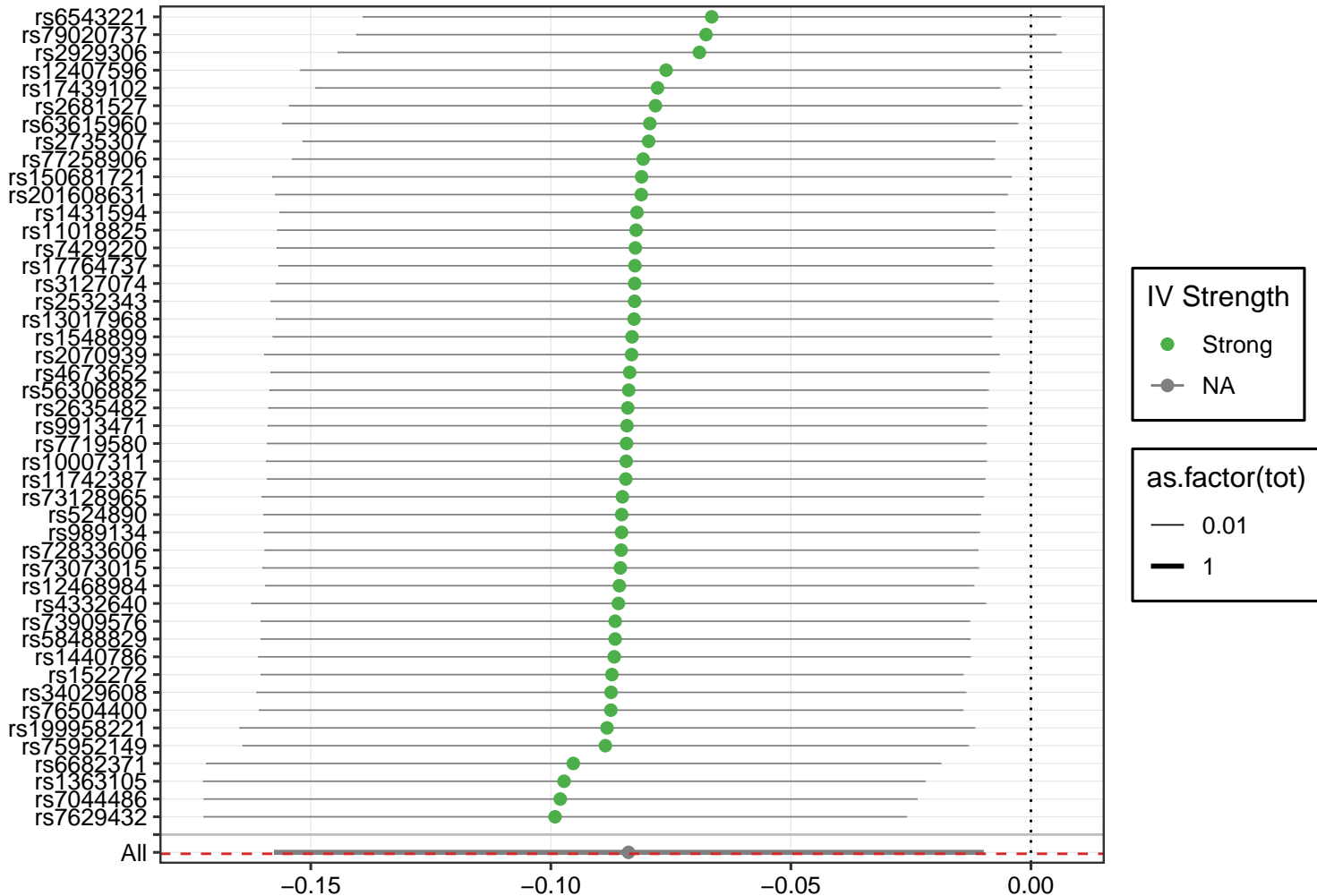

Supplement: Supplementary file 2 — Supplementary Material 2 [file 13568_2025_1969_MOESM2_ESM.zip › Revised supplementary materials/6 Inverse MR analysis results/plot/leaveoneout_or_CAG-83 sp002392625.pdf]

# Leave-One-Out (Sorted): Enterococcus A

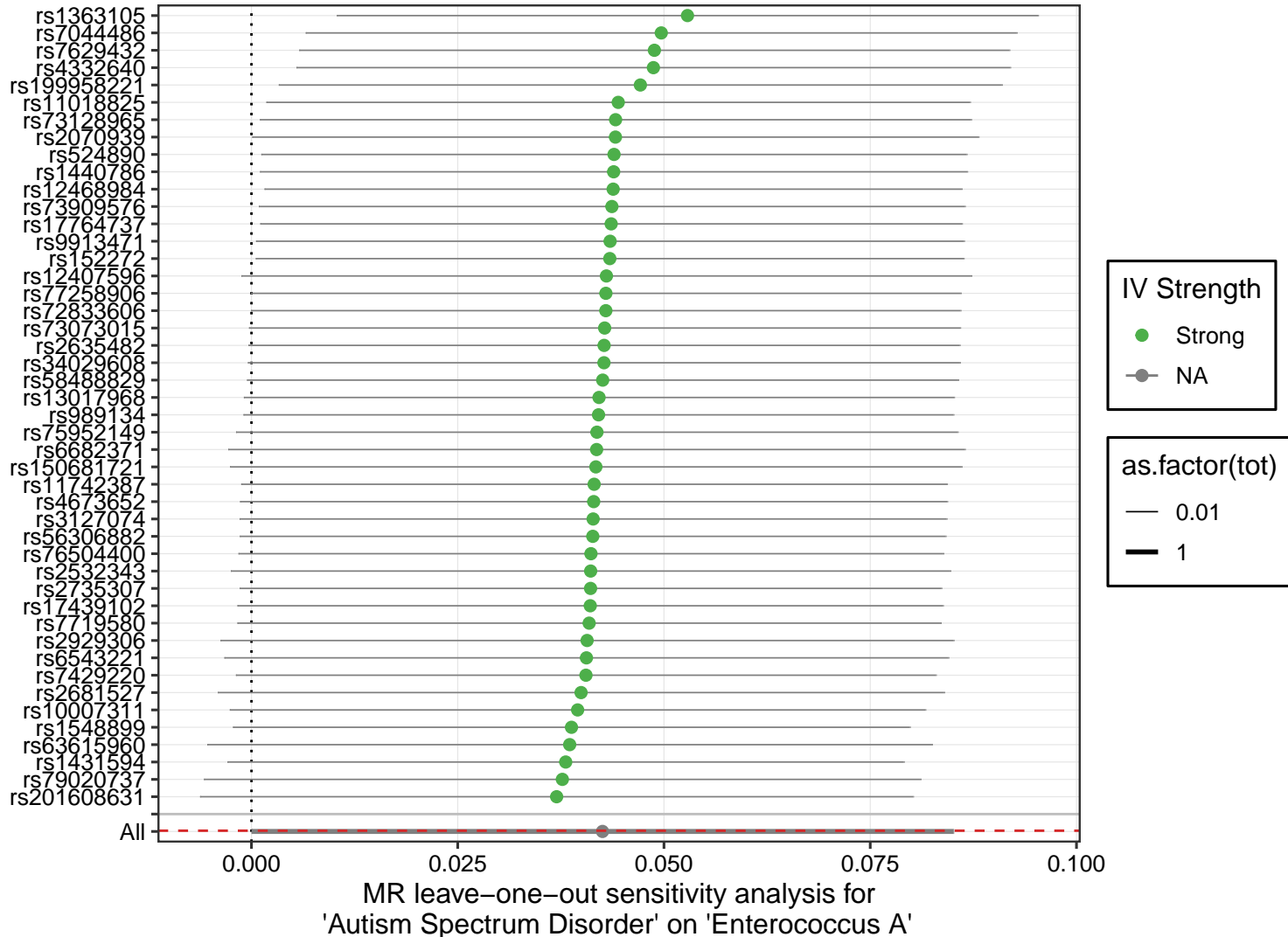

Supplement: Supplementary file 2 — Supplementary Material 2 [file 13568_2025_1969_MOESM2_ESM.zip › Revised supplementary materials/6 Inverse MR analysis results/plot/leaveoneout_or_Enterococcus A.pdf]

# Leave-One-Out (Sorted): Enterococcus B

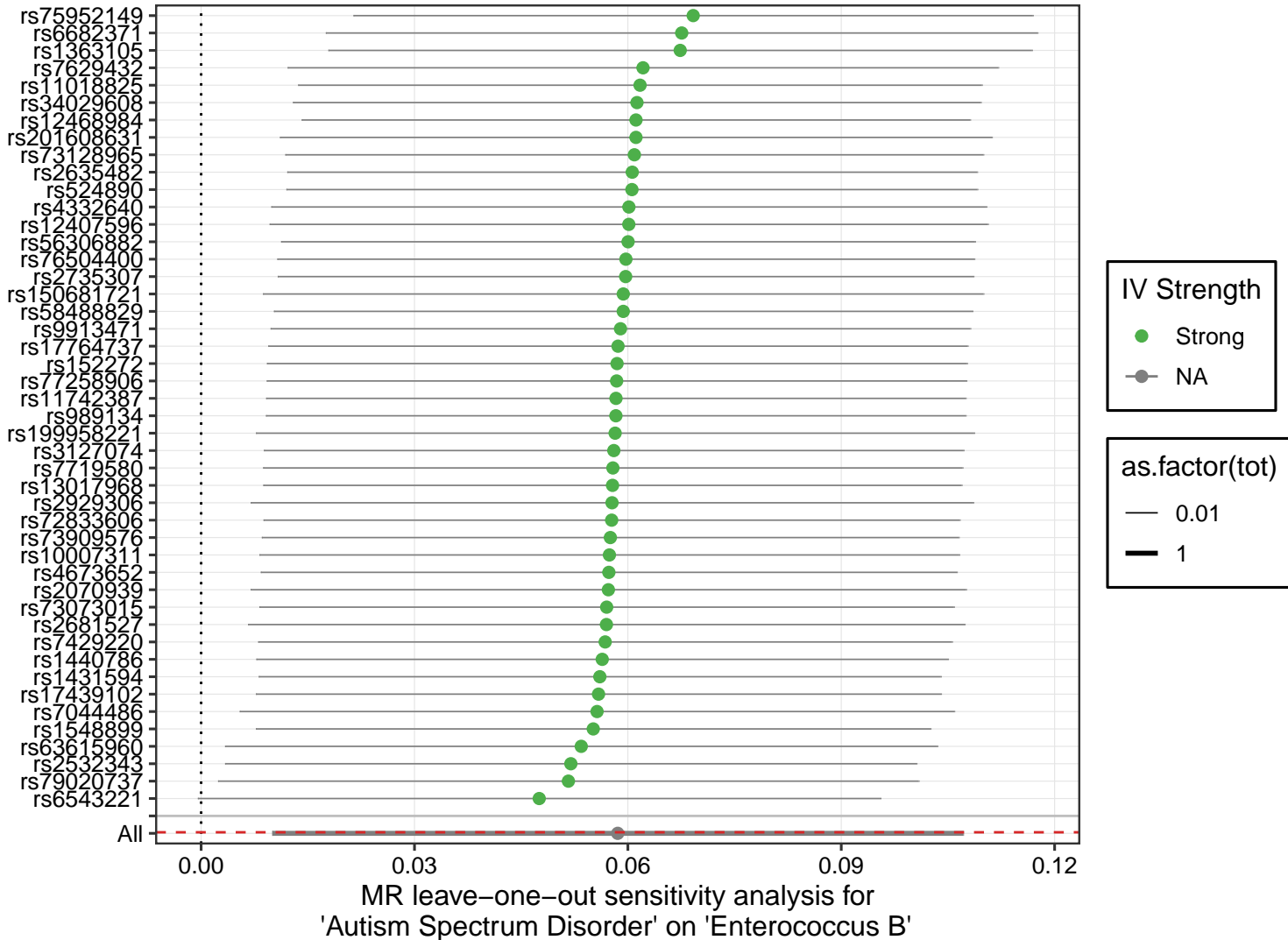

Supplement: Supplementary file 2 — Supplementary Material 2 [file 13568_2025_1969_MOESM2_ESM.zip › Revised supplementary materials/6 Inverse MR analysis results/plot/leaveoneout_or_Enterococcus B.pdf]

# Leave-One-Out (Sorted): Faecalibacterium sp002160895

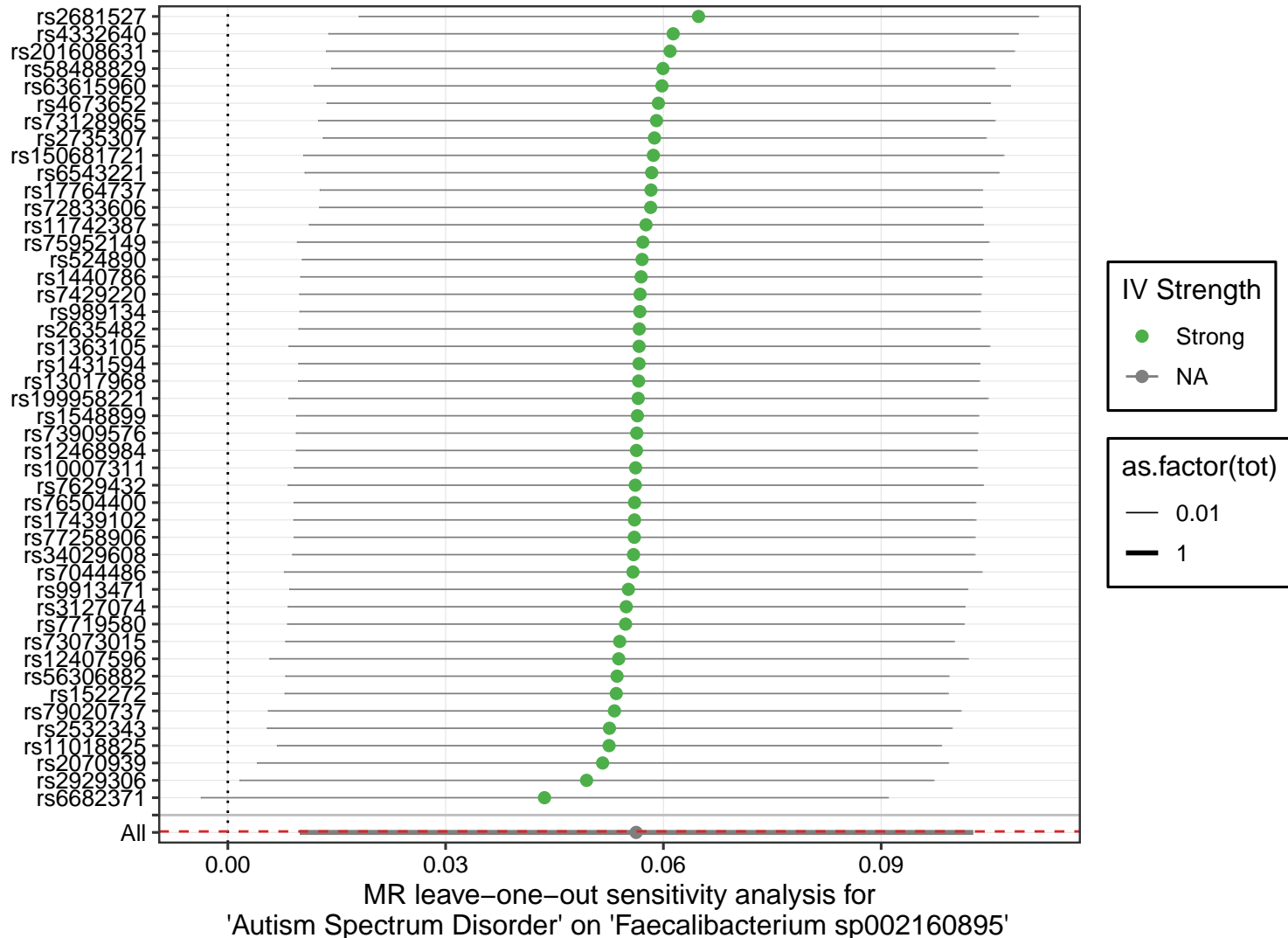

Supplement: Supplementary file 2 — Supplementary Material 2 [file 13568_2025_1969_MOESM2_ESM.zip › Revised supplementary materials/6 Inverse MR analysis results/plot/leaveoneout_or_Faecalibacterium sp002160895.pdf]
