# Supplementary figures and images for: Multi-omics reveals cross-tissue regulatory mechanisms of autism risk loci via gut microbiota-immunity-brain axis (part 1 of 2)
Source: AMB Express. 2025 Oct 29;15:161. doi: 10.1186/s13568-025-01969-4 (PMC12572420; doi:10.1186/s13568-025-01969-4)

ASD novel loci

cg00631329 (NA)

▲ top cis-eQTL

● cis-eQTL

GWAS effect sizes

0.018  
0  
-0.018  
-0.036  
-0.054

-0.35 0 0.35 0.7 1.05

eQTL effect sizes

$r^2$

1.00  
0.81  
0.62  
0.43  
0.25  
0.06

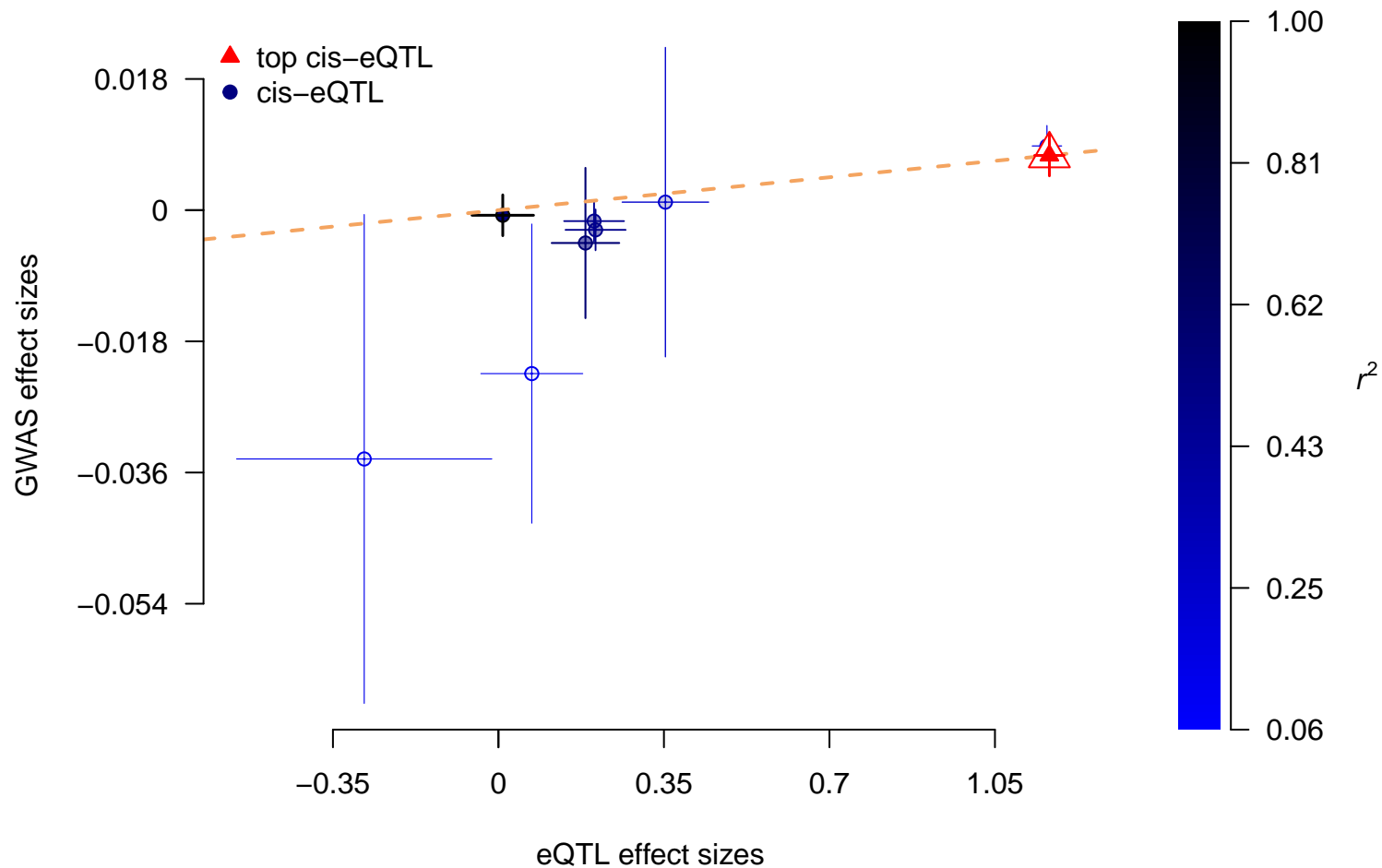

Supplement: Supplementary file 2 — Supplementary Material 2 [file 13568_2025_1969_MOESM2_ESM.zip › Revised supplementary materials/4 Novel loci SMR results/plot/cg00631329_EffectPlot.pdf]

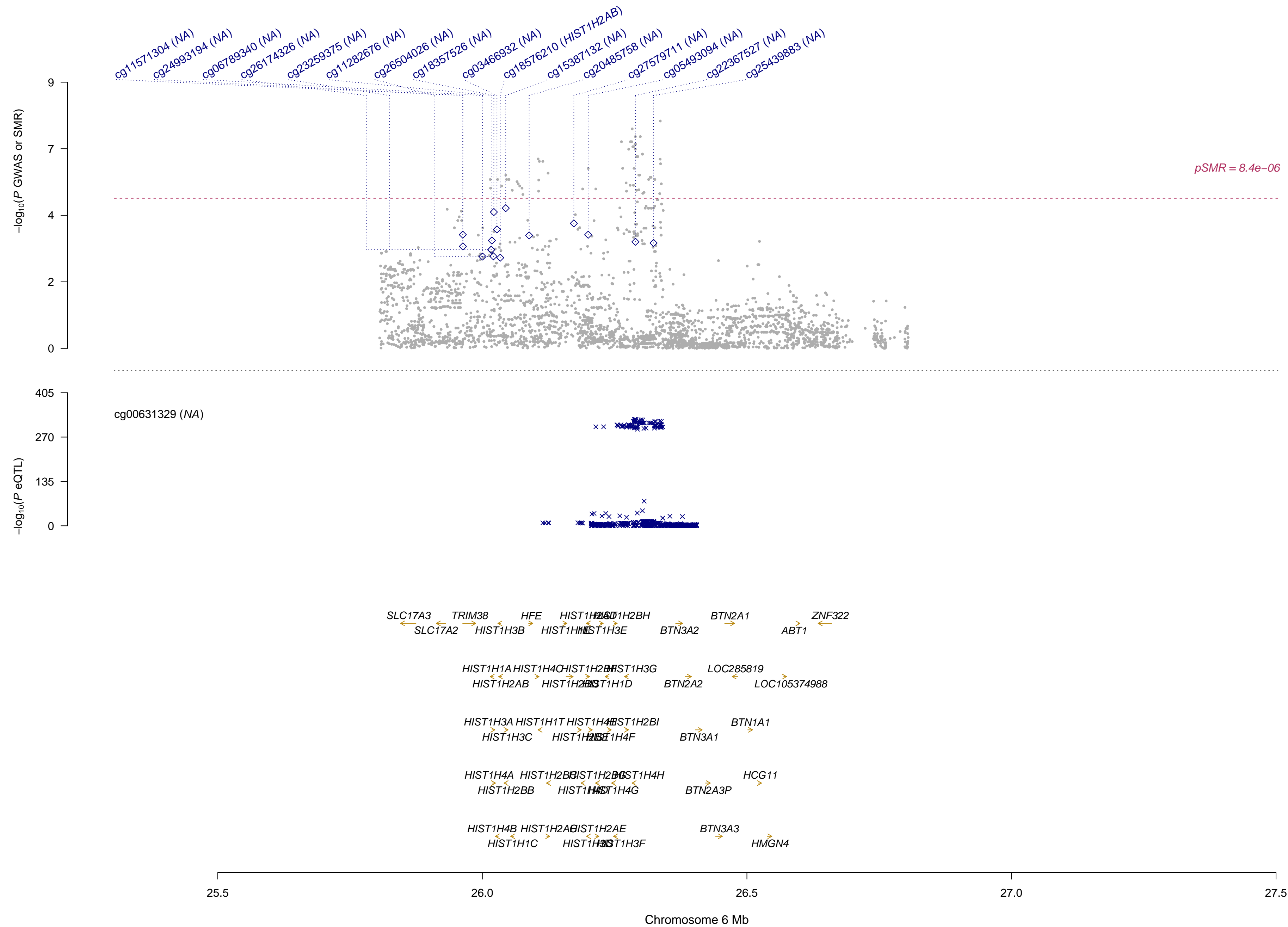

Supplement: Supplementary file 2 — Supplementary Material 2 [file 13568_2025_1969_MOESM2_ESM.zip › Revised supplementary materials/4 Novel loci SMR results/plot/cg00631329_LocusPlot.pdf]

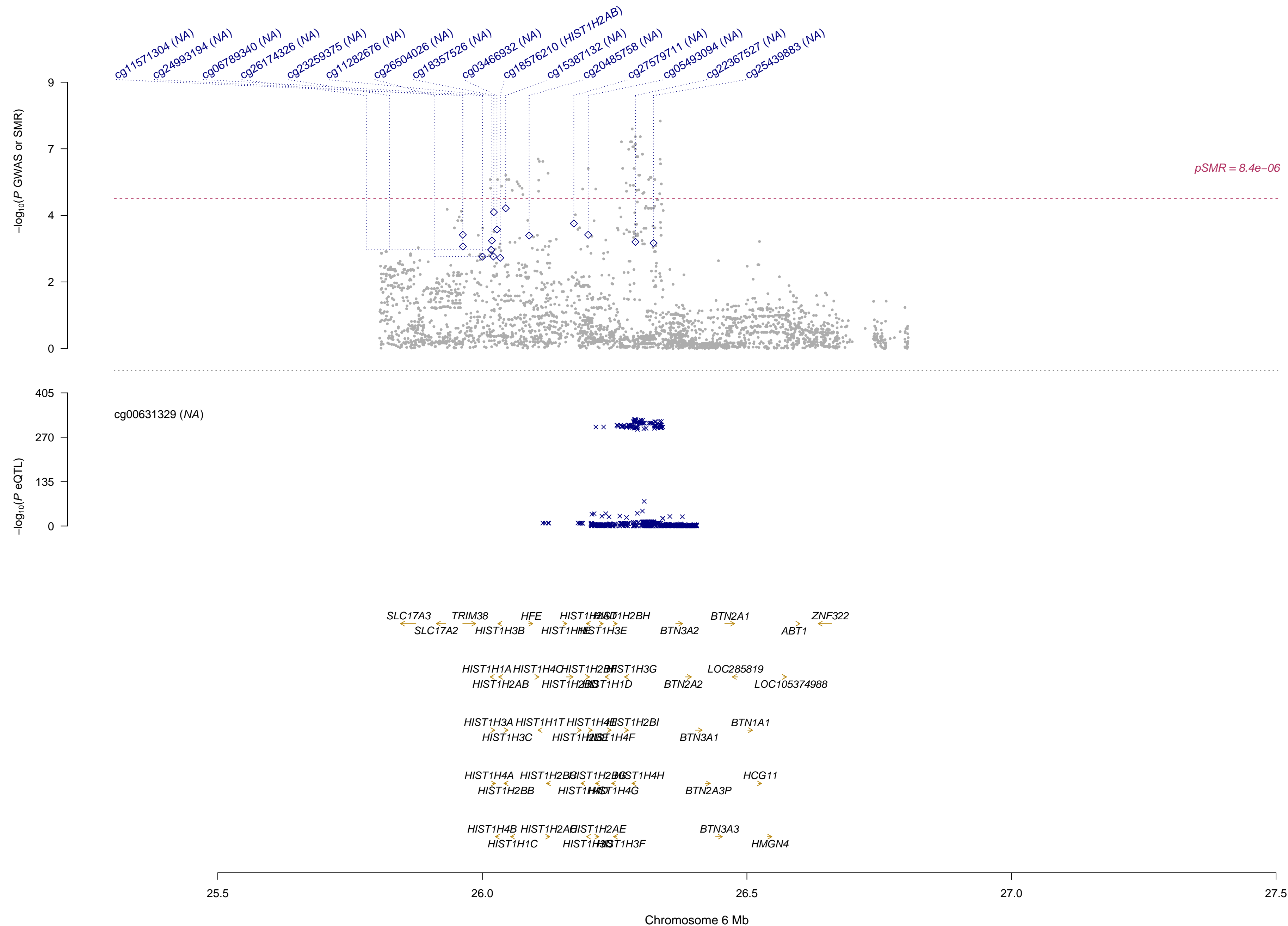

Supplement: Supplementary file 2 — Supplementary Material 2 [file 13568_2025_1969_MOESM2_ESM.zip › Revised supplementary materials/4 Novel loci SMR results/plot/cg02269231_LocusPlot.pdf]

ASD novel loci

cg11644478 (NA)

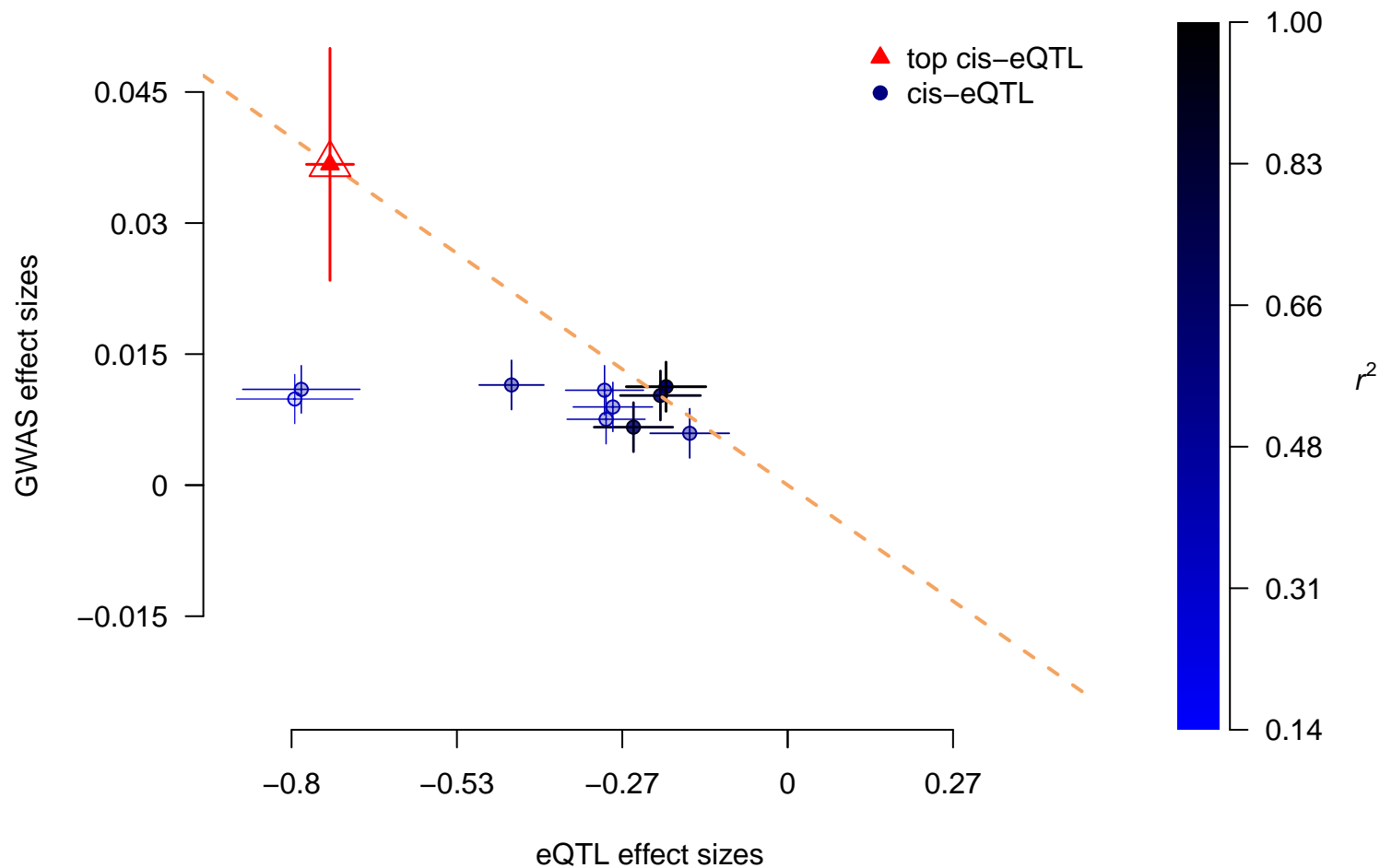

Supplement: Supplementary file 2 — Supplementary Material 2 [file 13568_2025_1969_MOESM2_ESM.zip › Revised supplementary materials/4 Novel loci SMR results/plot/cg11644478_EffectPlot.pdf]

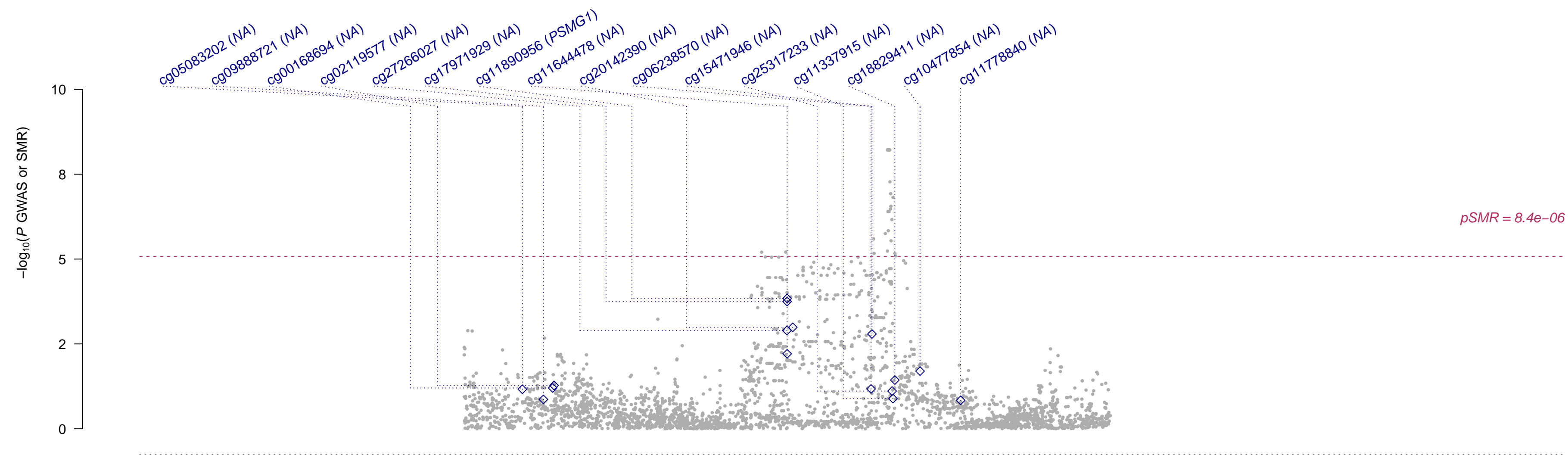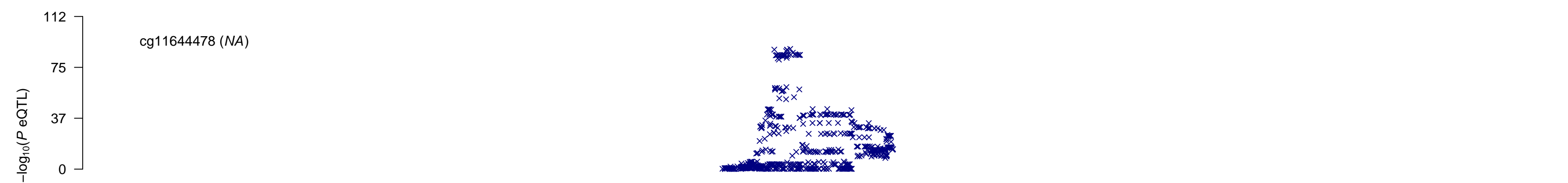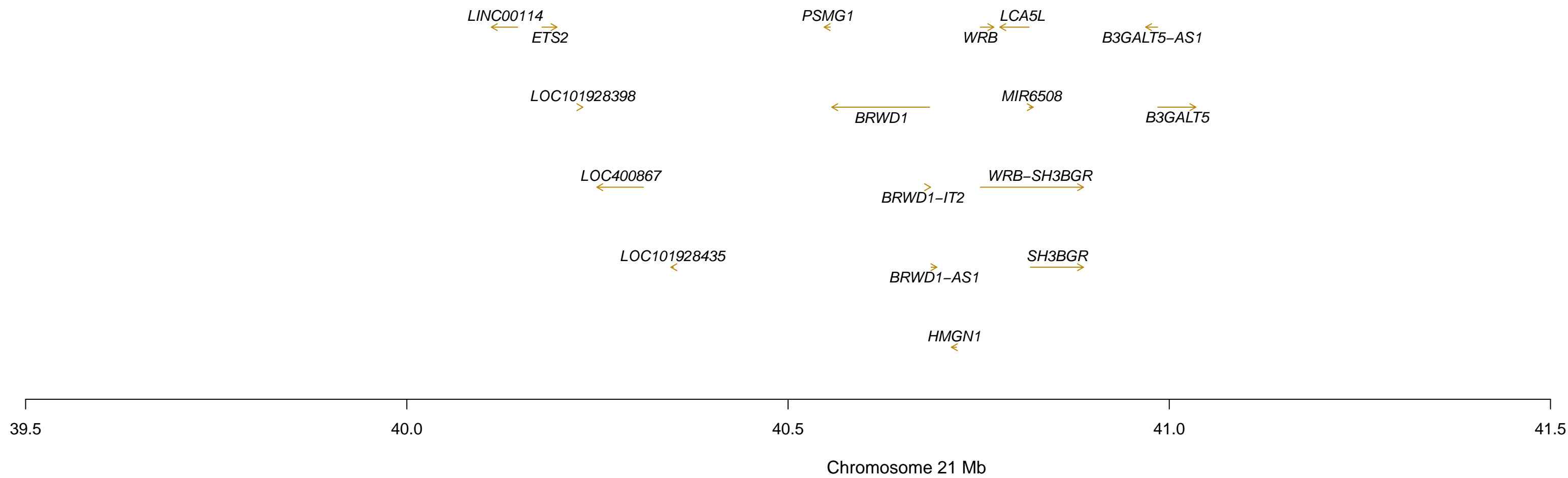

Supplement: Supplementary file 2 — Supplementary Material 2 [file 13568_2025_1969_MOESM2_ESM.zip › Revised supplementary materials/4 Novel loci SMR results/plot/cg11644478_LocusPlot.pdf]

ASD novel loci

cg11890956 (*PSMG1*)

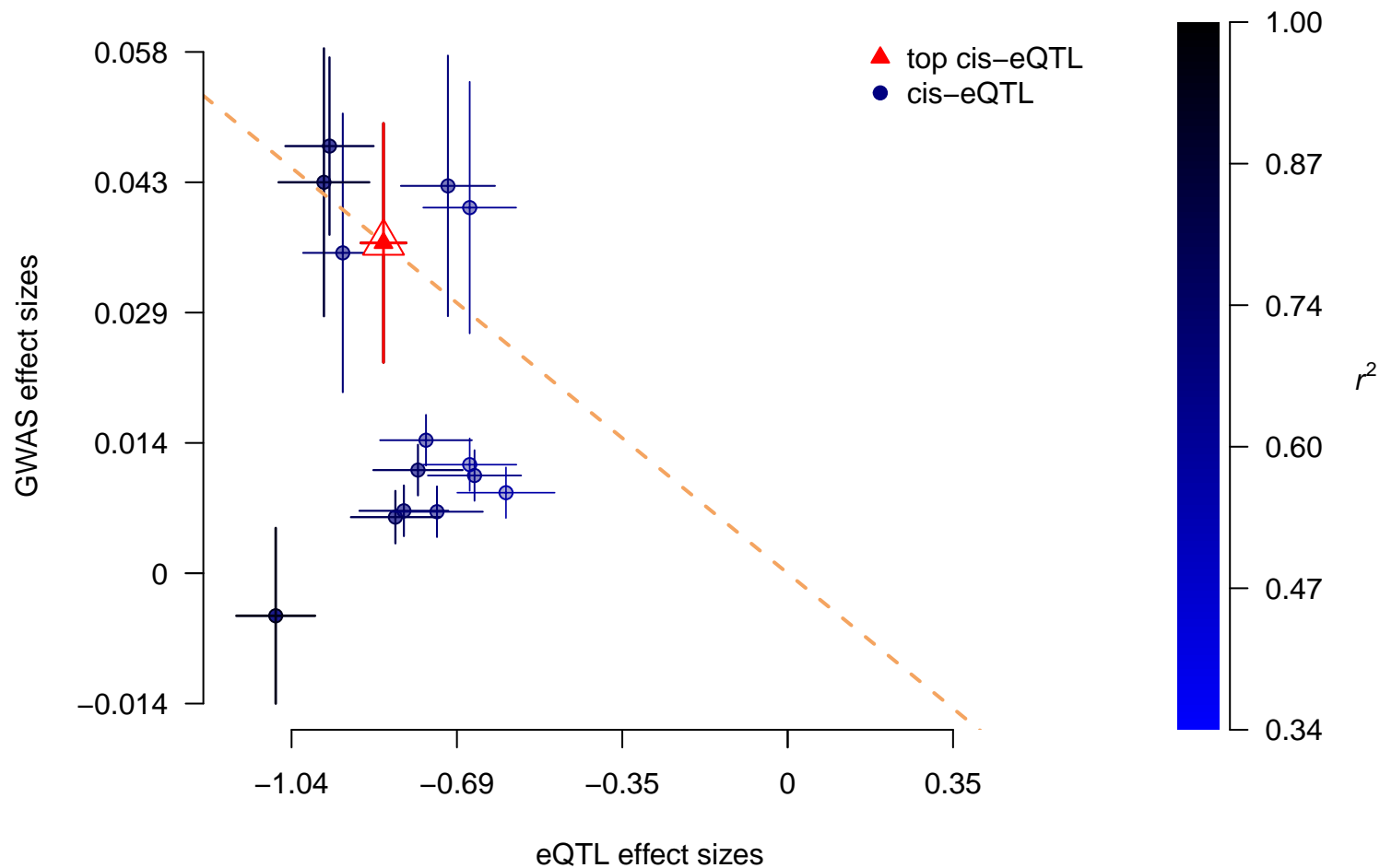

Supplement: Supplementary file 2 — Supplementary Material 2 [file 13568_2025_1969_MOESM2_ESM.zip › Revised supplementary materials/4 Novel loci SMR results/plot/cg11890956_EffectPlot.pdf]

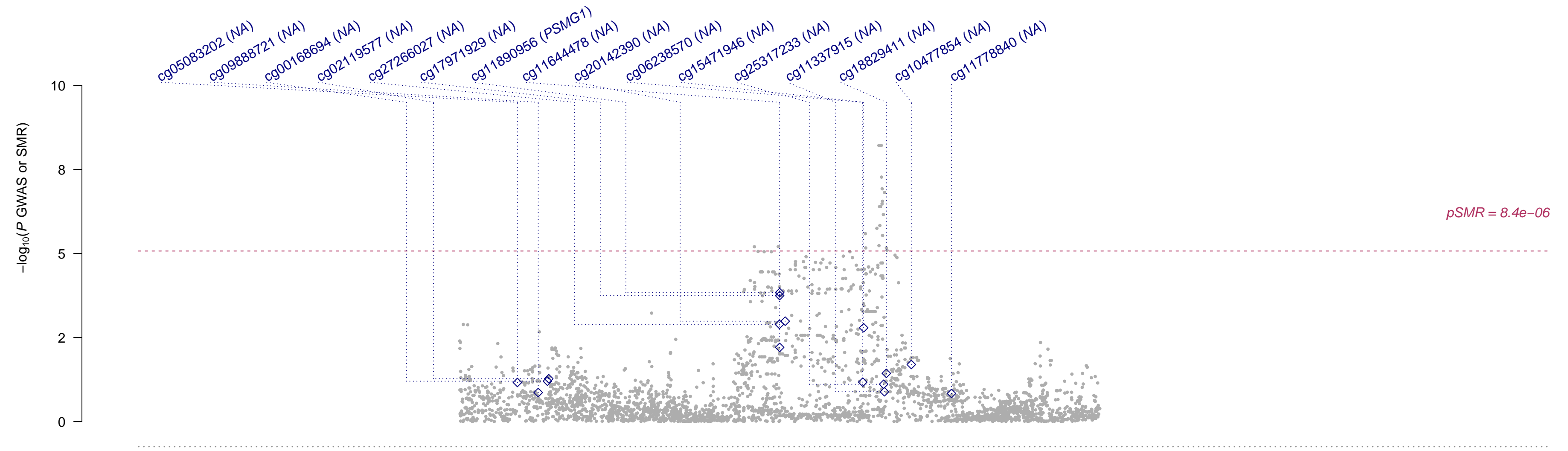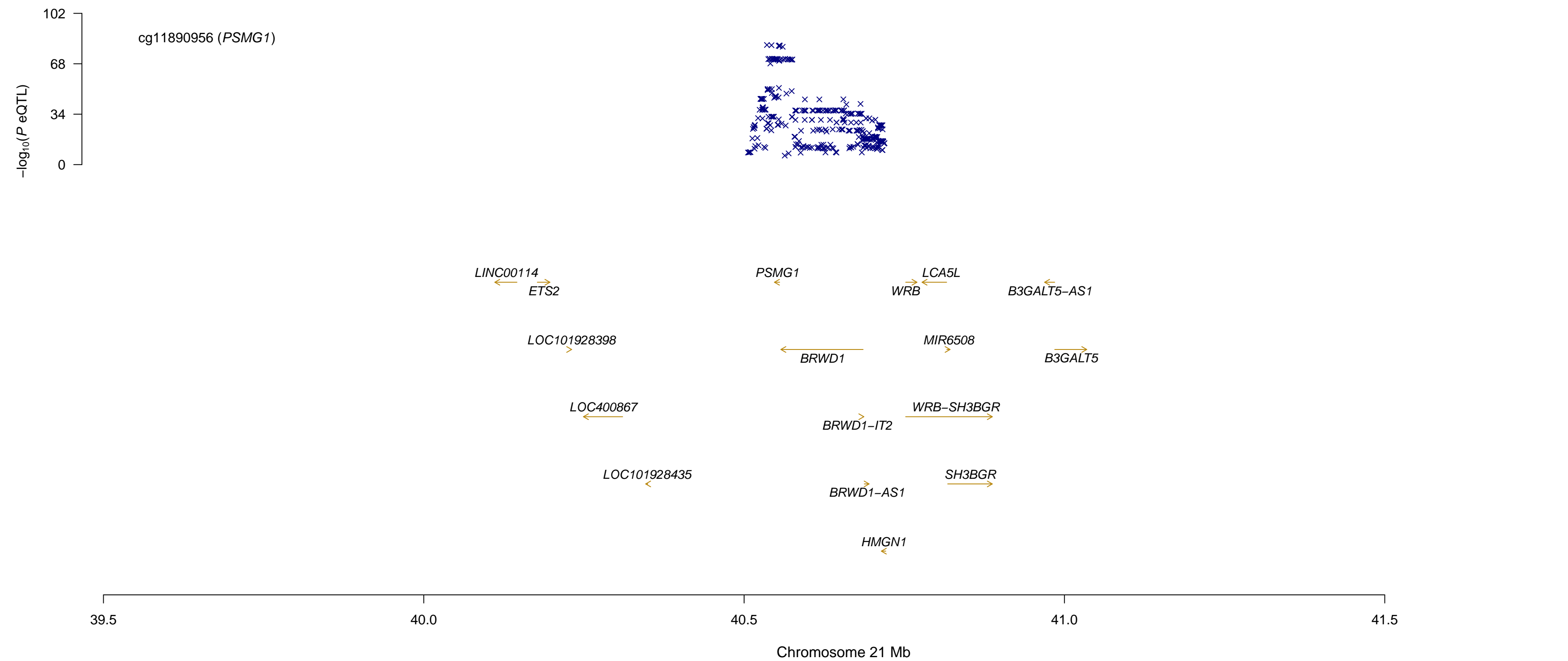

Supplement: Supplementary file 2 — Supplementary Material 2 [file 13568_2025_1969_MOESM2_ESM.zip › Revised supplementary materials/4 Novel loci SMR results/plot/cg11890956_LocusPlot.pdf]

ASD novel loci

cg13736514 (NA)

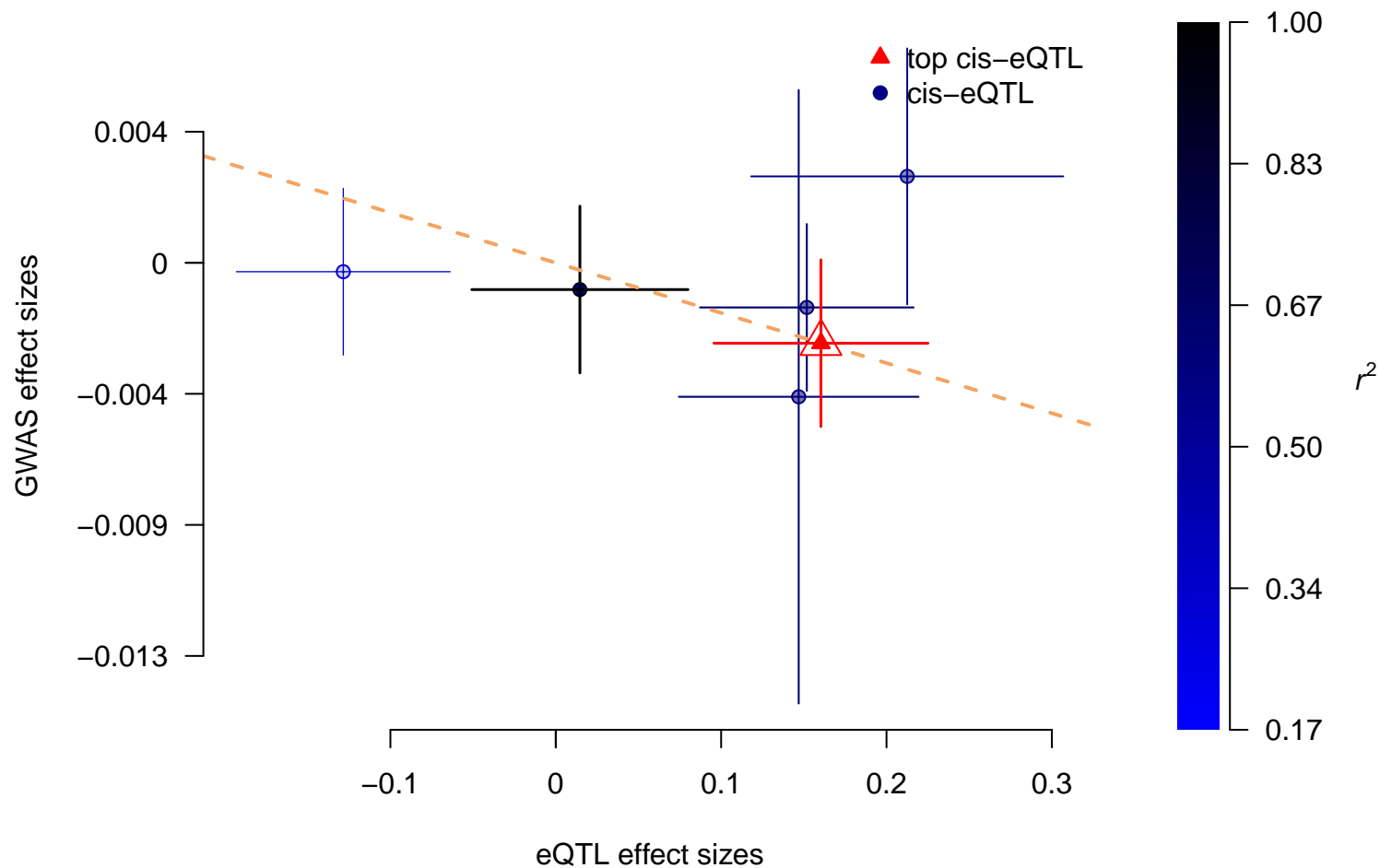

Supplement: Supplementary file 2 — Supplementary Material 2 [file 13568_2025_1969_MOESM2_ESM.zip › Revised supplementary materials/4 Novel loci SMR results/plot/cg13736514_EffectPlot.pdf]

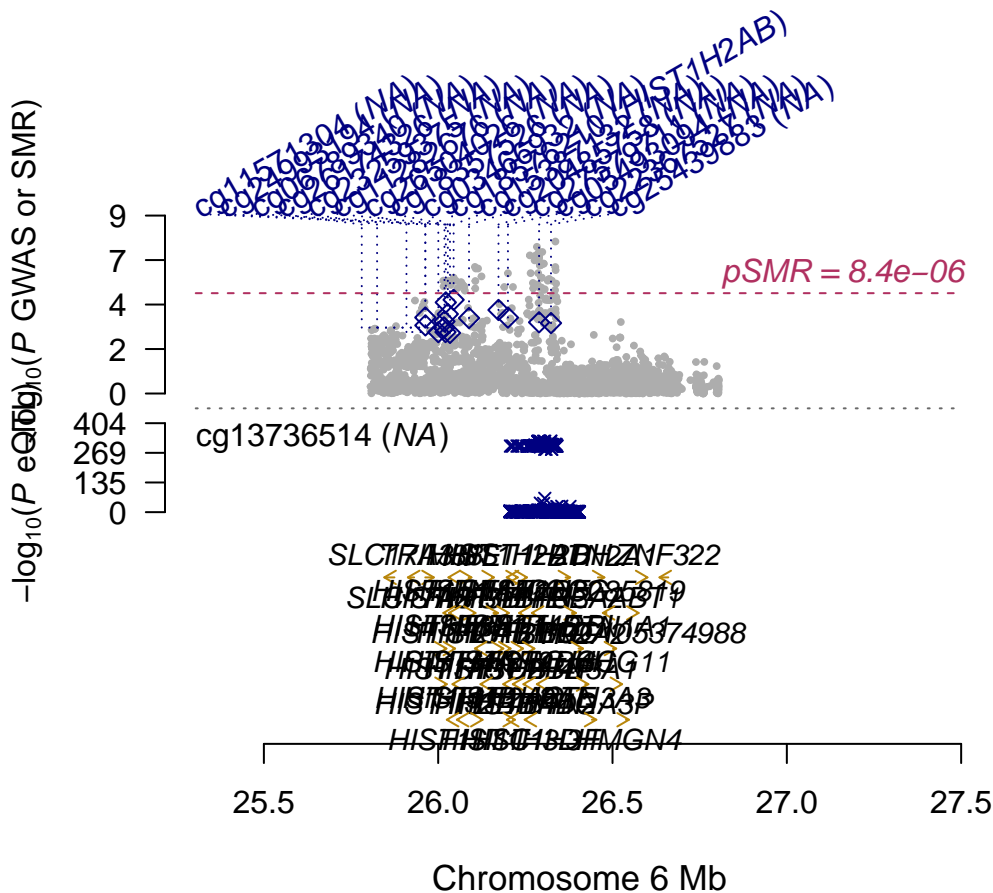

Supplement: Supplementary file 2 — Supplementary Material 2 [file 13568_2025_1969_MOESM2_ESM.zip › Revised supplementary materials/4 Novel loci SMR results/plot/cg13736514_LocusPlot.pdf]

ASD novel loci

cg15471946 (NA)

▲ top cis-eQTL

● cis-eQTL

GWAS effect sizes

0.008  
0.005  
0.003  
0  
-0.003

-0.01 0.04 0.09 0.15 0.2 0.24 0.29 0.35 0.4

eQTL effect sizes

$r^2$

1.00

0.83

0.65

0.48

0.31

0.13

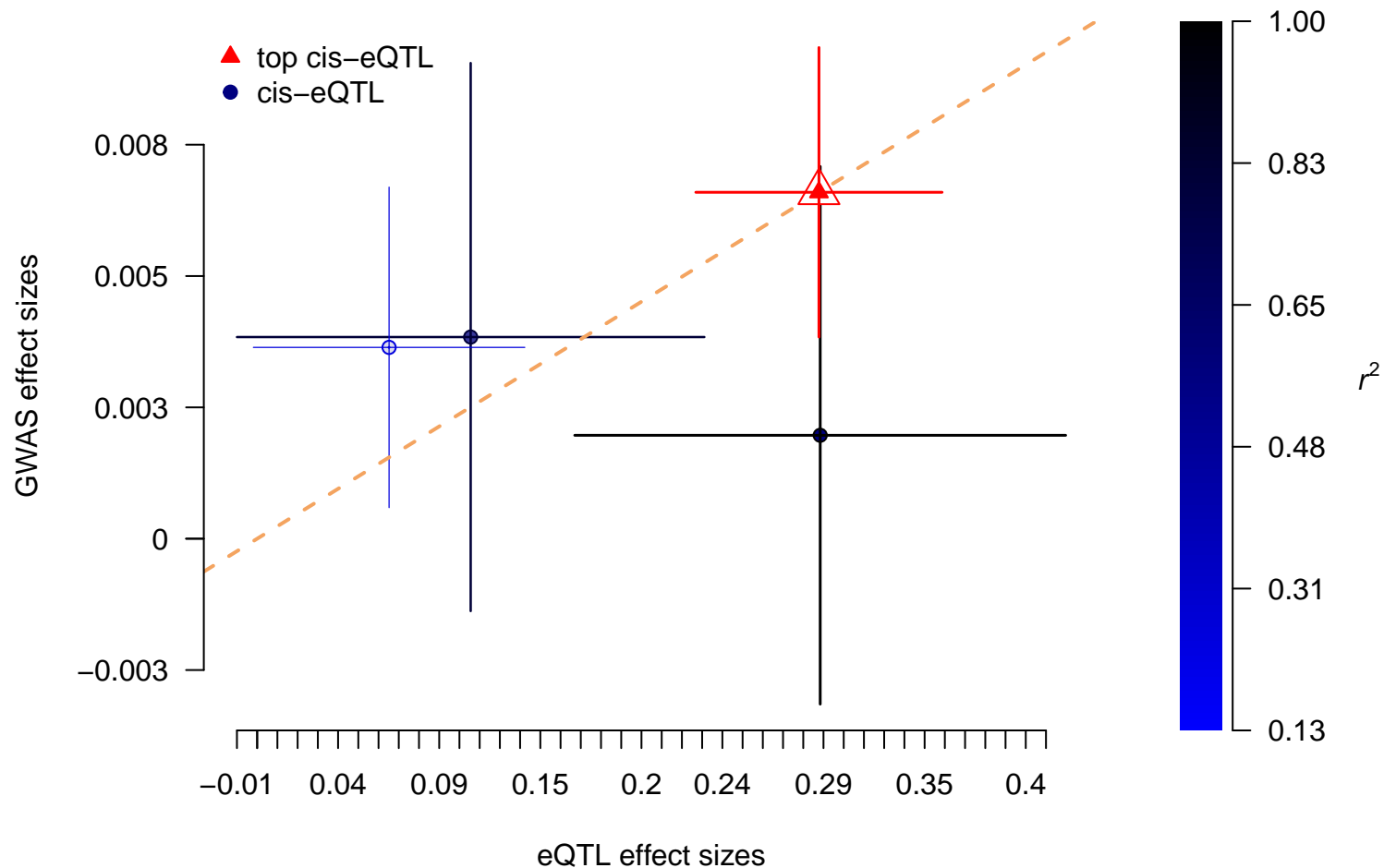

Supplement: Supplementary file 2 — Supplementary Material 2 [file 13568_2025_1969_MOESM2_ESM.zip › Revised supplementary materials/4 Novel loci SMR results/plot/cg15471946_EffectPlot.pdf]

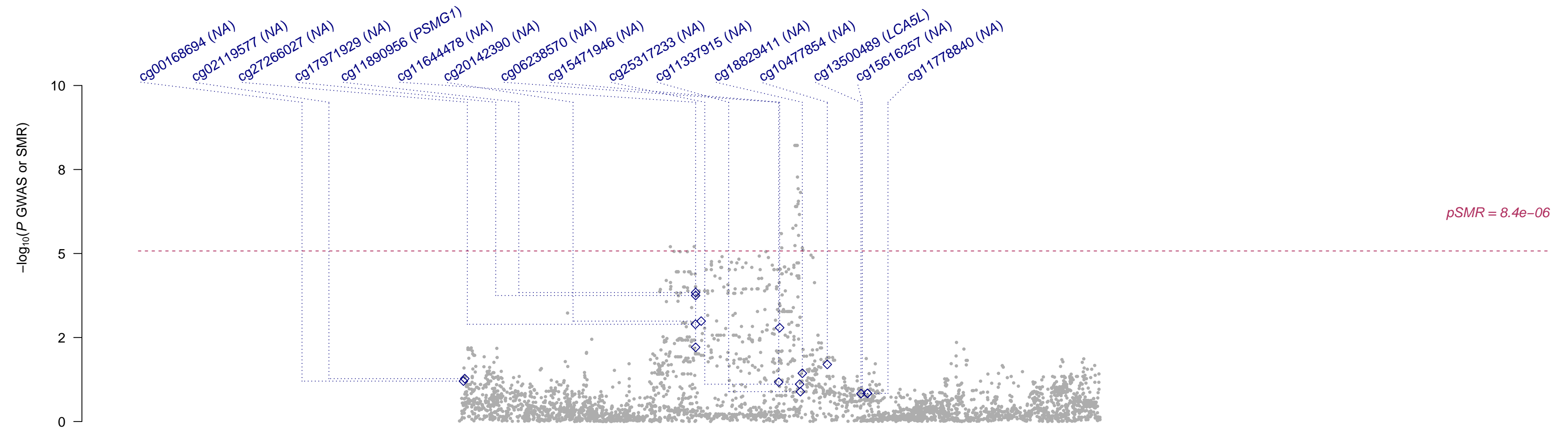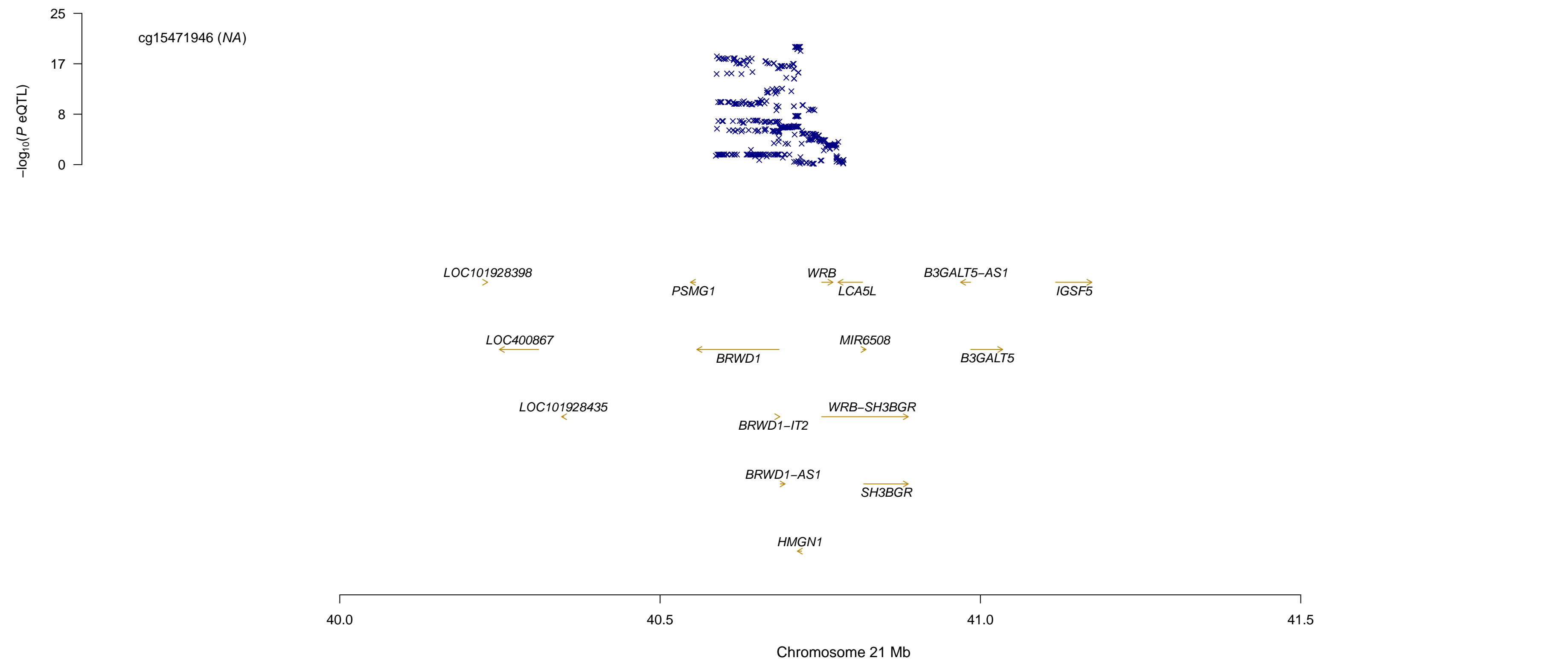

Supplement: Supplementary file 2 — Supplementary Material 2 [file 13568_2025_1969_MOESM2_ESM.zip › Revised supplementary materials/4 Novel loci SMR results/plot/cg15471946_LocusPlot.pdf]

ASD novel loci

cg16579770 (NA)

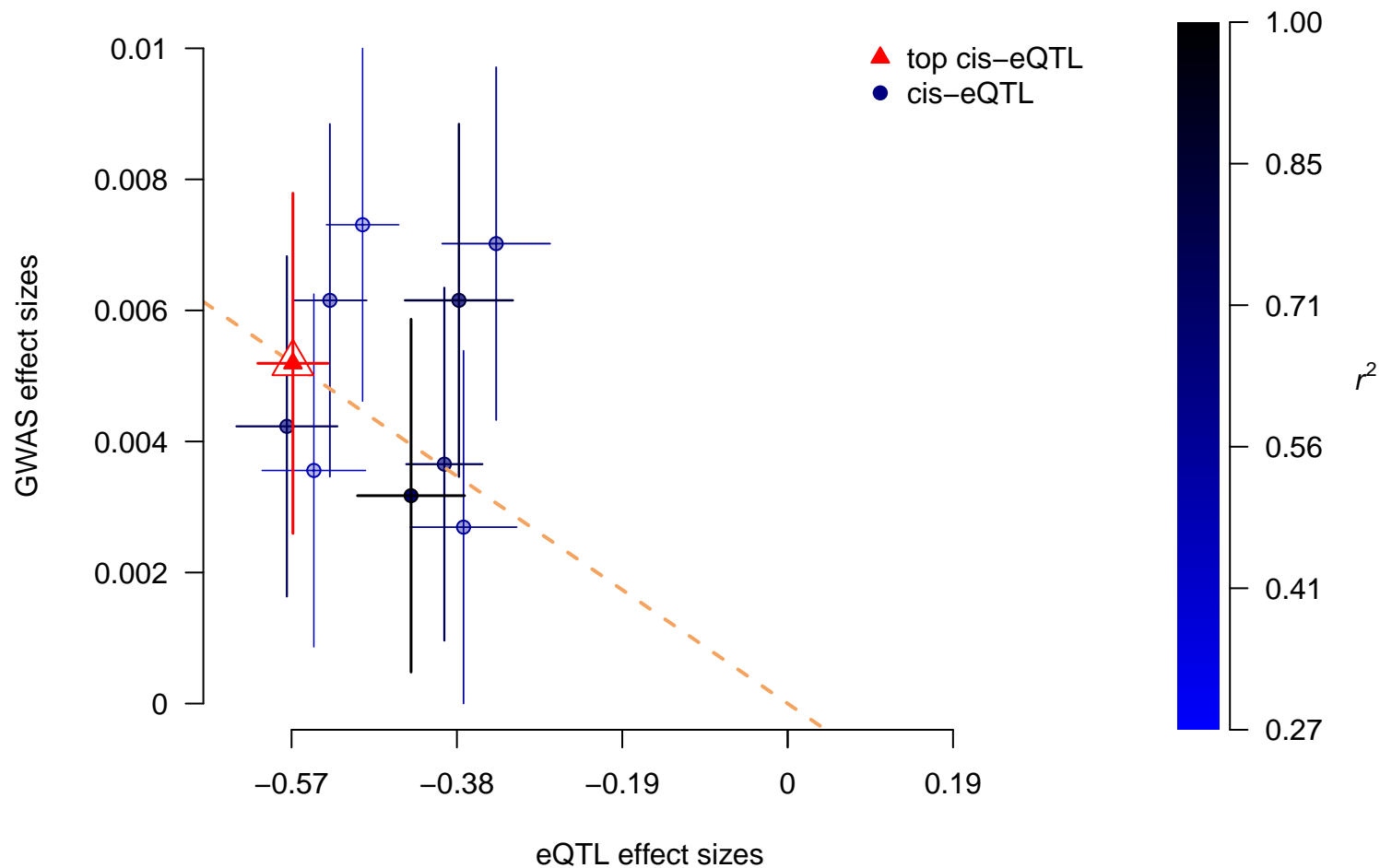

Supplement: Supplementary file 2 — Supplementary Material 2 [file 13568_2025_1969_MOESM2_ESM.zip › Revised supplementary materials/4 Novel loci SMR results/plot/cg16579770_EffectPlot.pdf]

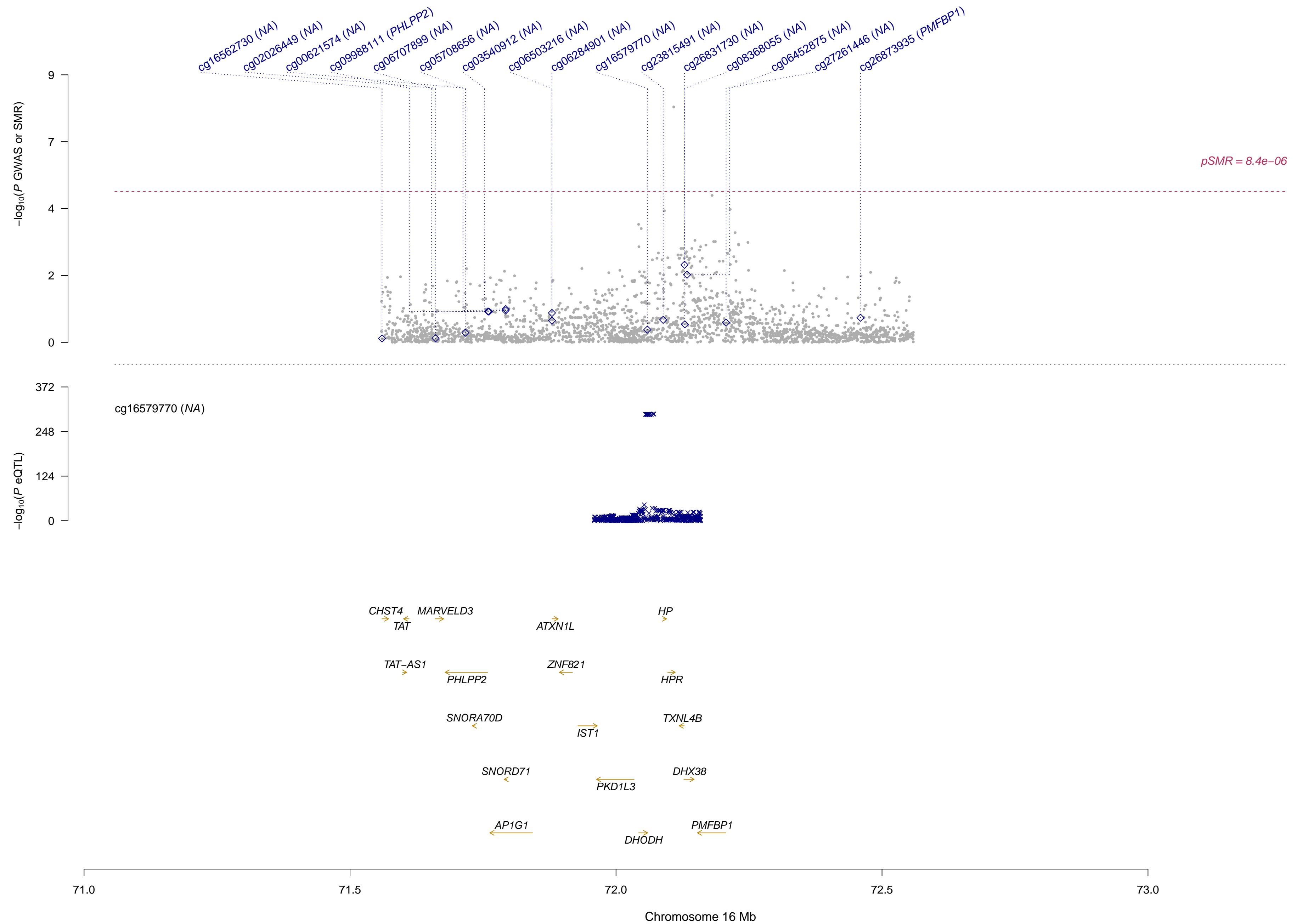

Supplement: Supplementary file 2 — Supplementary Material 2 [file 13568_2025_1969_MOESM2_ESM.zip › Revised supplementary materials/4 Novel loci SMR results/plot/cg16579770_LocusPlot.pdf]

ASD novel loci

cg17971929 (NA)

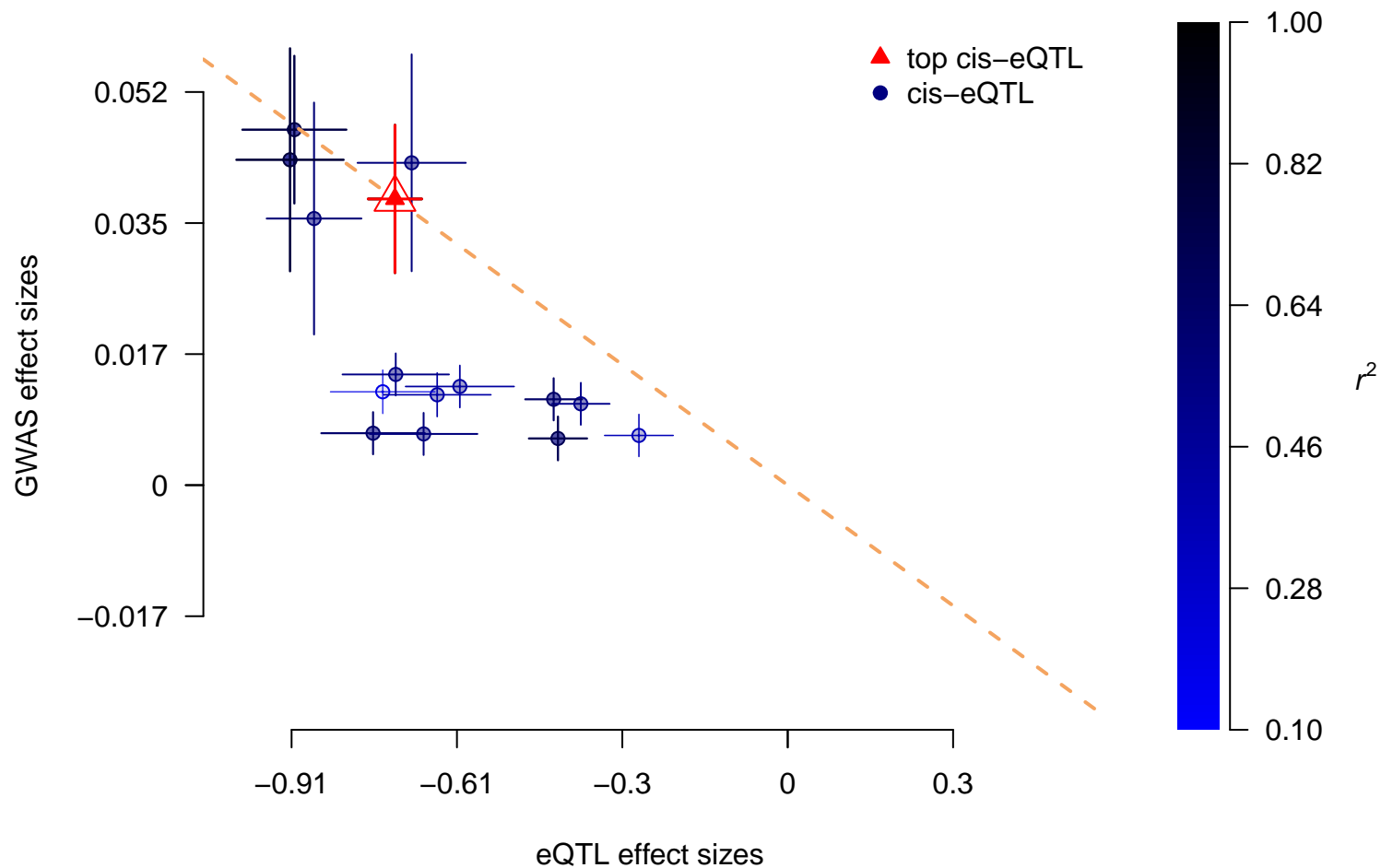

Supplement: Supplementary file 2 — Supplementary Material 2 [file 13568_2025_1969_MOESM2_ESM.zip › Revised supplementary materials/4 Novel loci SMR results/plot/cg17971929_EffectPlot.pdf]

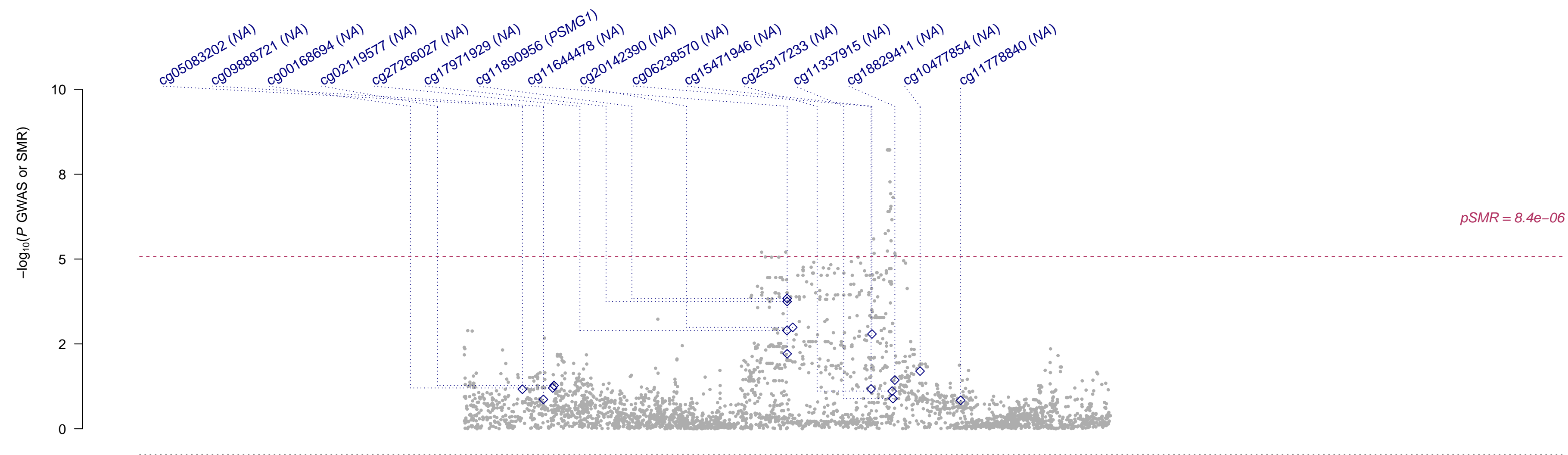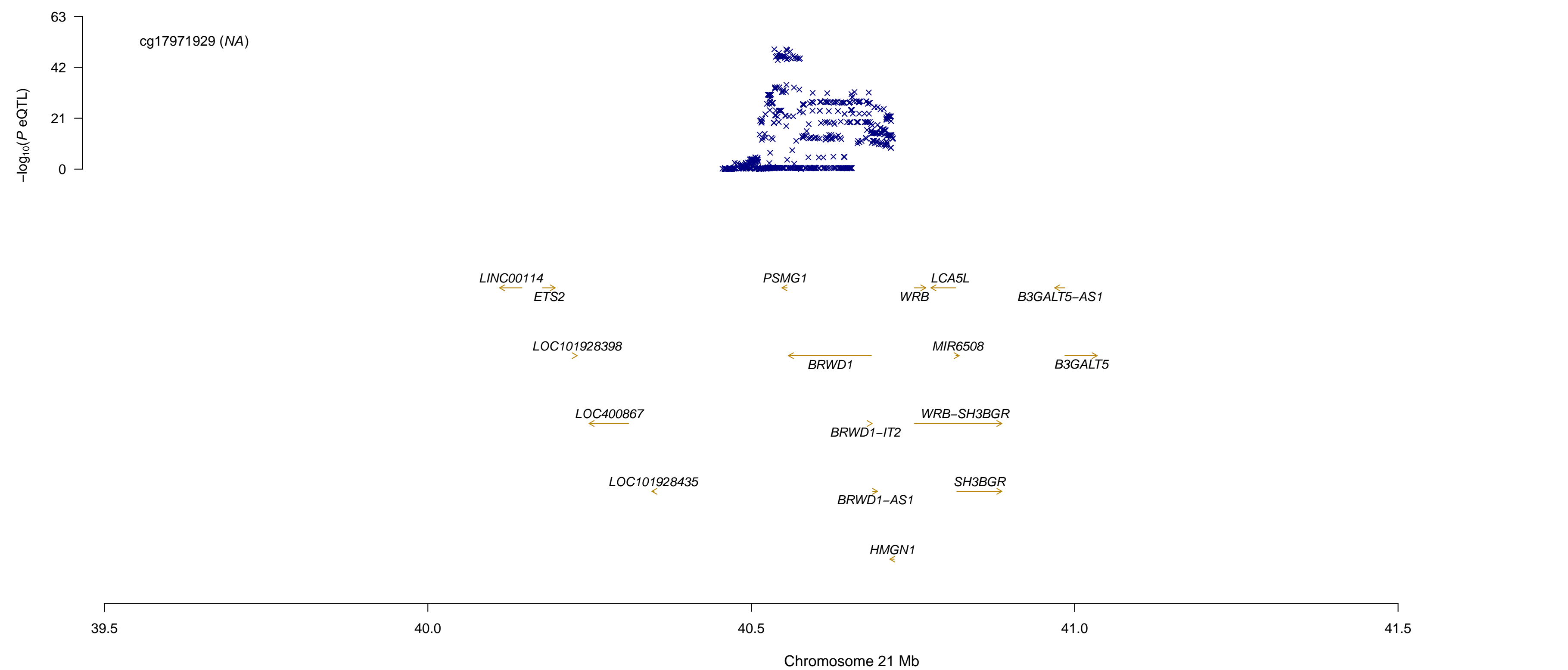

Supplement: Supplementary file 2 — Supplementary Material 2 [file 13568_2025_1969_MOESM2_ESM.zip › Revised supplementary materials/4 Novel loci SMR results/plot/cg17971929_LocusPlot.pdf]

ASD novel loci

cg22367527 (NA)

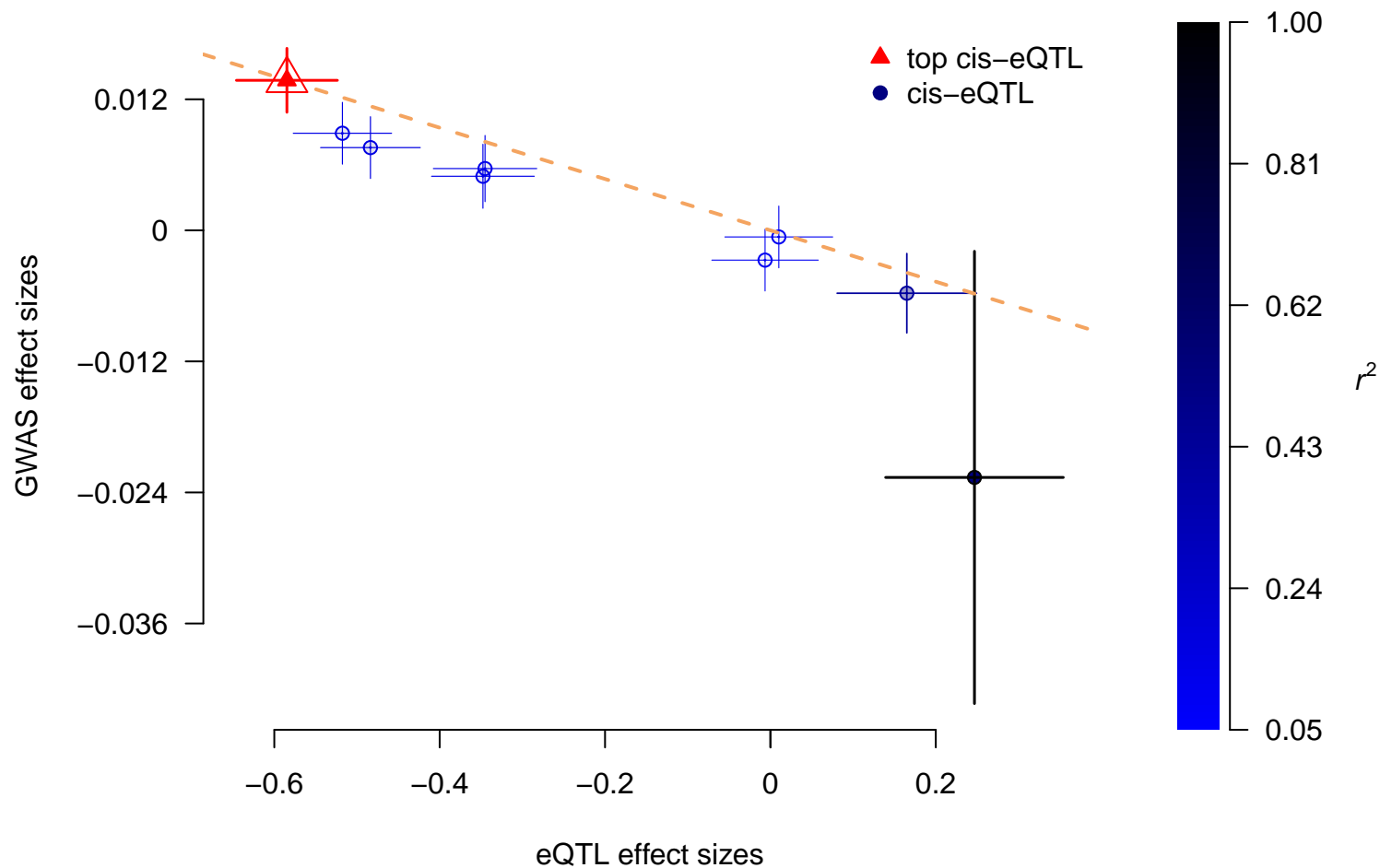

Supplement: Supplementary file 2 — Supplementary Material 2 [file 13568_2025_1969_MOESM2_ESM.zip › Revised supplementary materials/4 Novel loci SMR results/plot/cg22367527_EffectPlot.pdf]

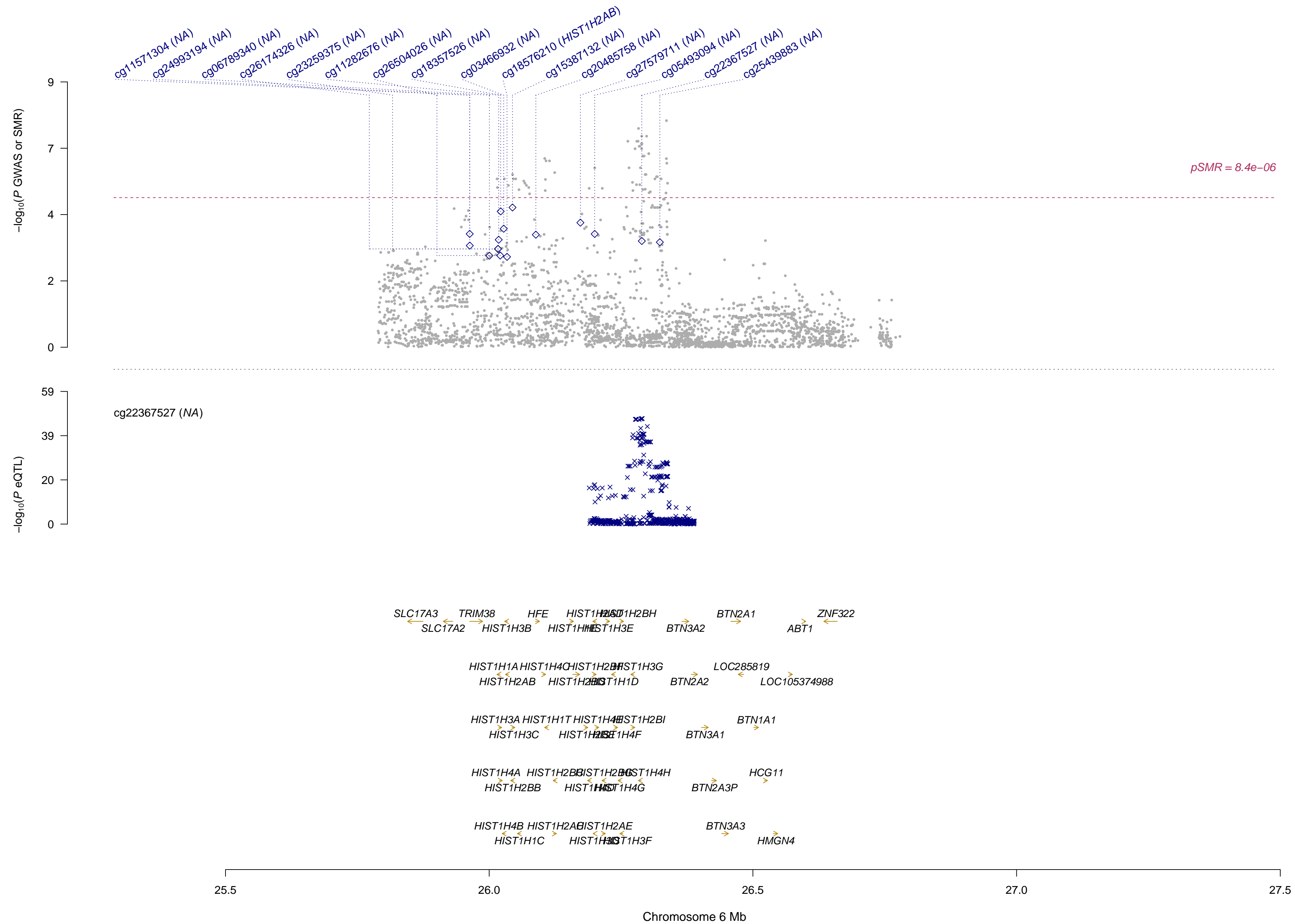

Supplement: Supplementary file 2 — Supplementary Material 2 [file 13568_2025_1969_MOESM2_ESM.zip › Revised supplementary materials/4 Novel loci SMR results/plot/cg22367527_LocusPlot.pdf]

ASD novel loci

cg23815491 (NA)

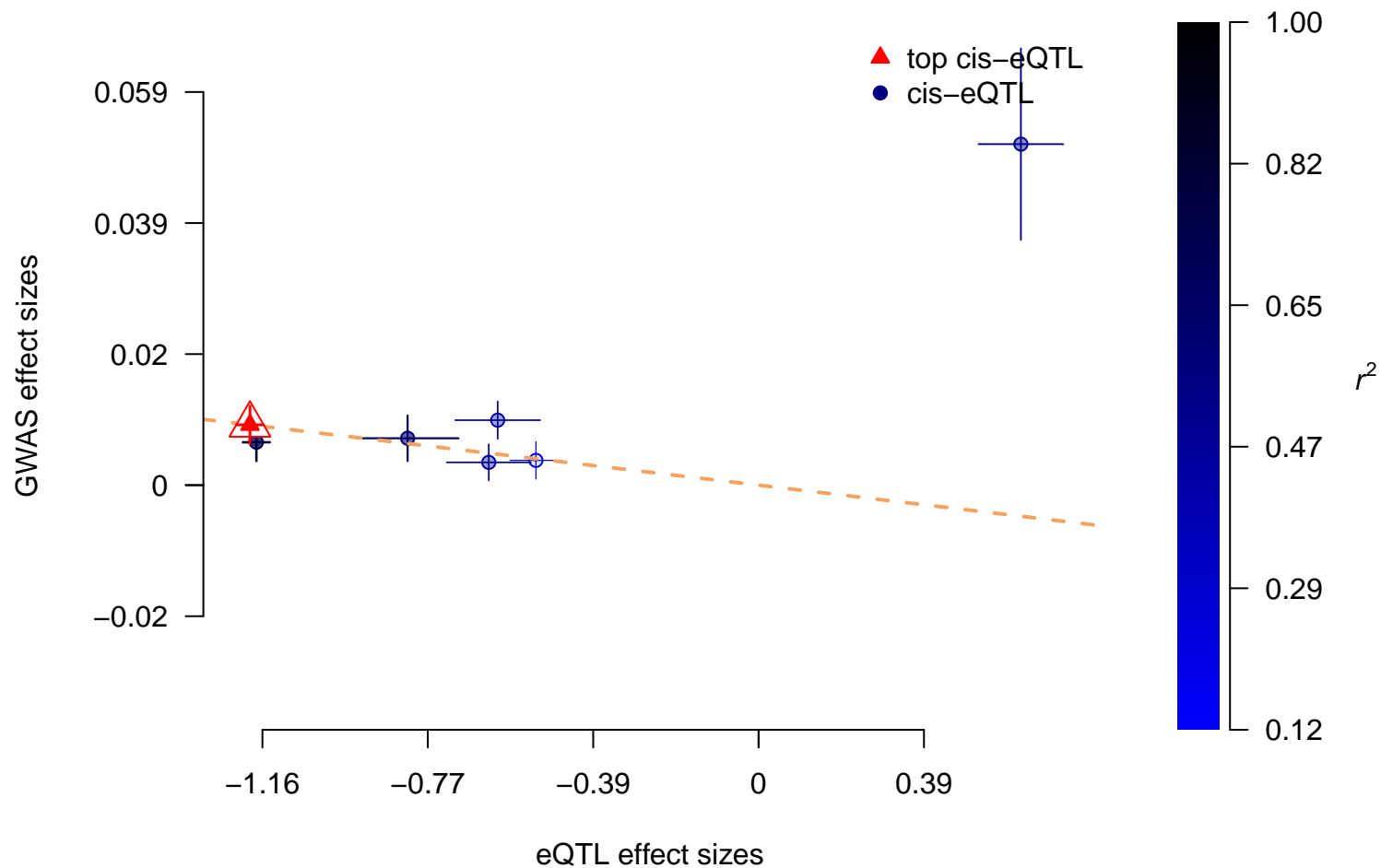

Supplement: Supplementary file 2 — Supplementary Material 2 [file 13568_2025_1969_MOESM2_ESM.zip › Revised supplementary materials/4 Novel loci SMR results/plot/cg23815491_EffectPlot.pdf]

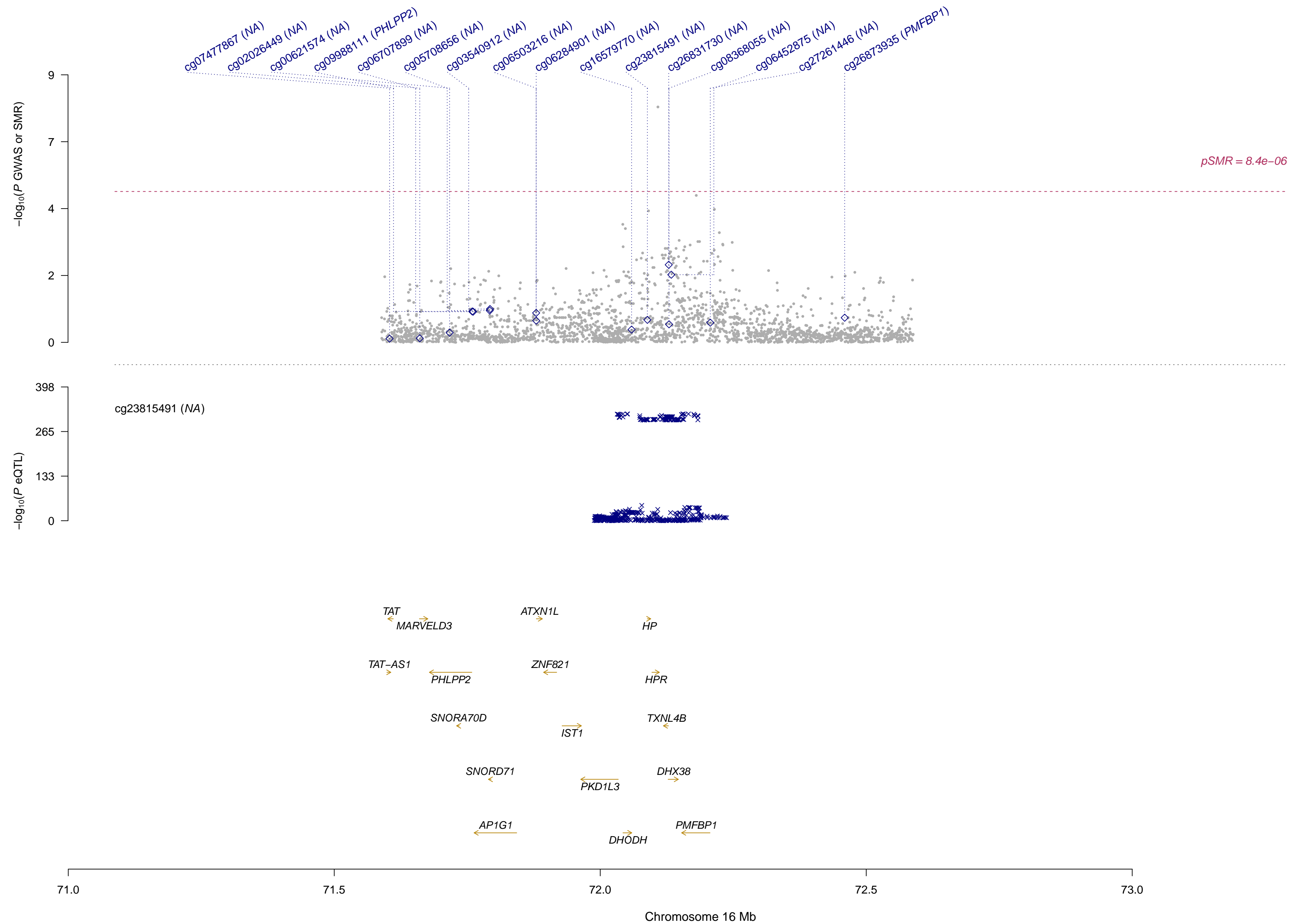

Supplement: Supplementary file 2 — Supplementary Material 2 [file 13568_2025_1969_MOESM2_ESM.zip › Revised supplementary materials/4 Novel loci SMR results/plot/cg23815491_LocusPlot.pdf]

ASD novel loci

cg25972943 (NA)

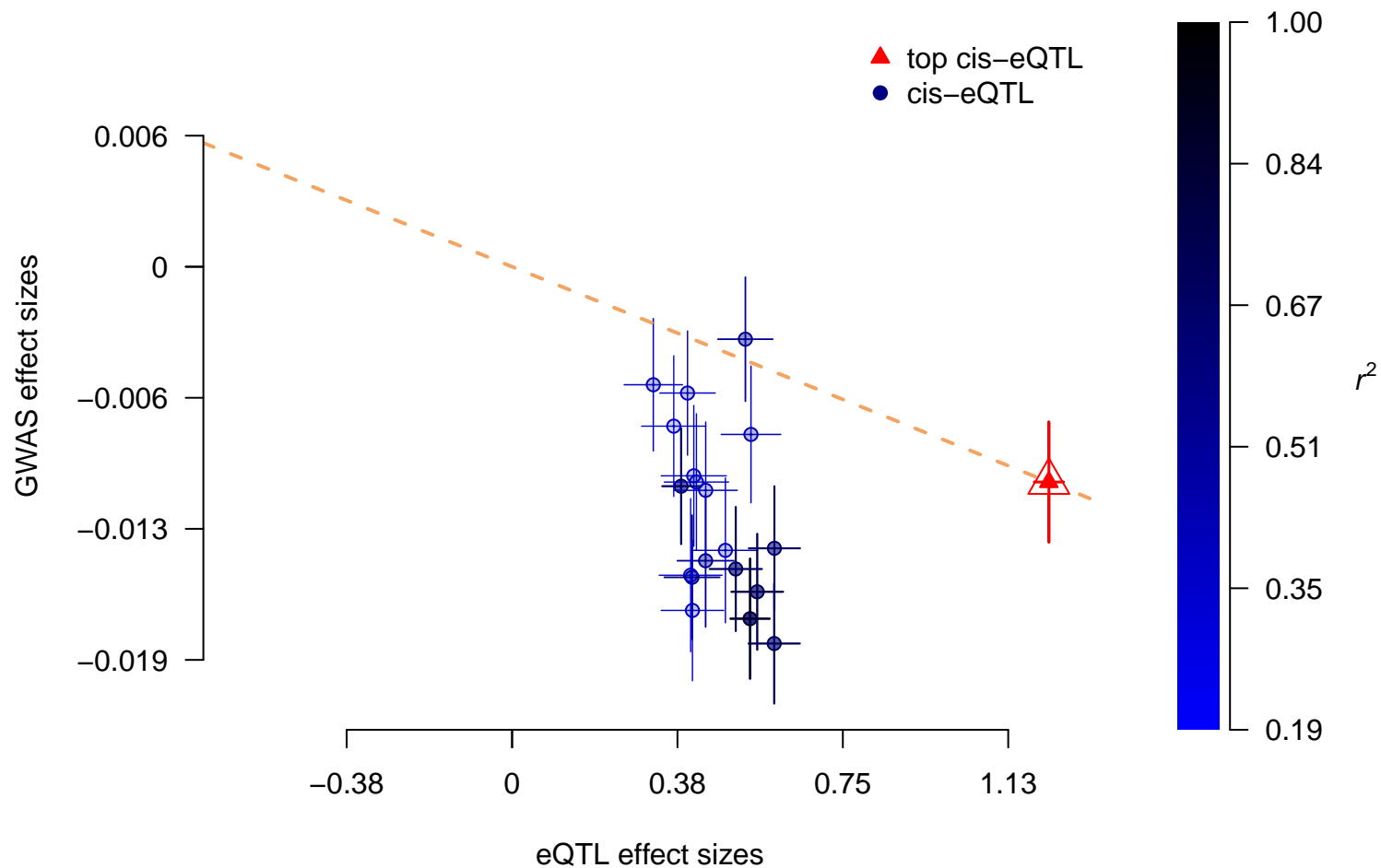

Supplement: Supplementary file 2 — Supplementary Material 2 [file 13568_2025_1969_MOESM2_ESM.zip › Revised supplementary materials/4 Novel loci SMR results/plot/cg25972943_EffectPlot.pdf]

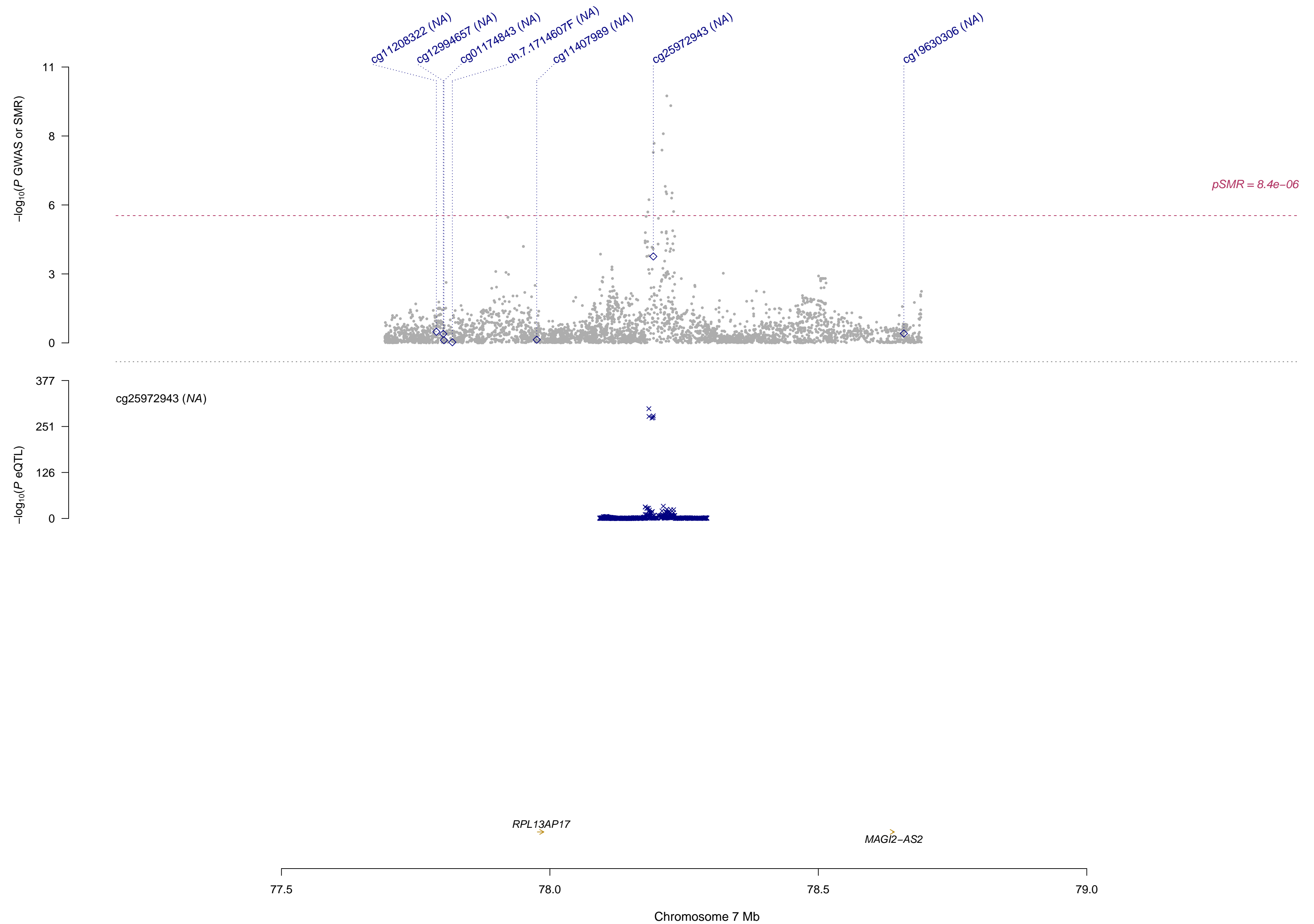

Supplement: Supplementary file 2 — Supplementary Material 2 [file 13568_2025_1969_MOESM2_ESM.zip › Revised supplementary materials/4 Novel loci SMR results/plot/cg25972943_LocusPlot.pdf]

ASD novel loci

cg26831730 (NA)

▲ top cis-eQTL

● cis-eQTL

GWAS effect sizes

0.012  
0.01  
0.009  
0.007  
0.005  
0.003  
0.002  
0  
-0.002

-0.33

-0.16

0

0.16

0.33

eQTL effect sizes

1.00

0.92

0.84

$r^2$

0.76

0.68

0.60

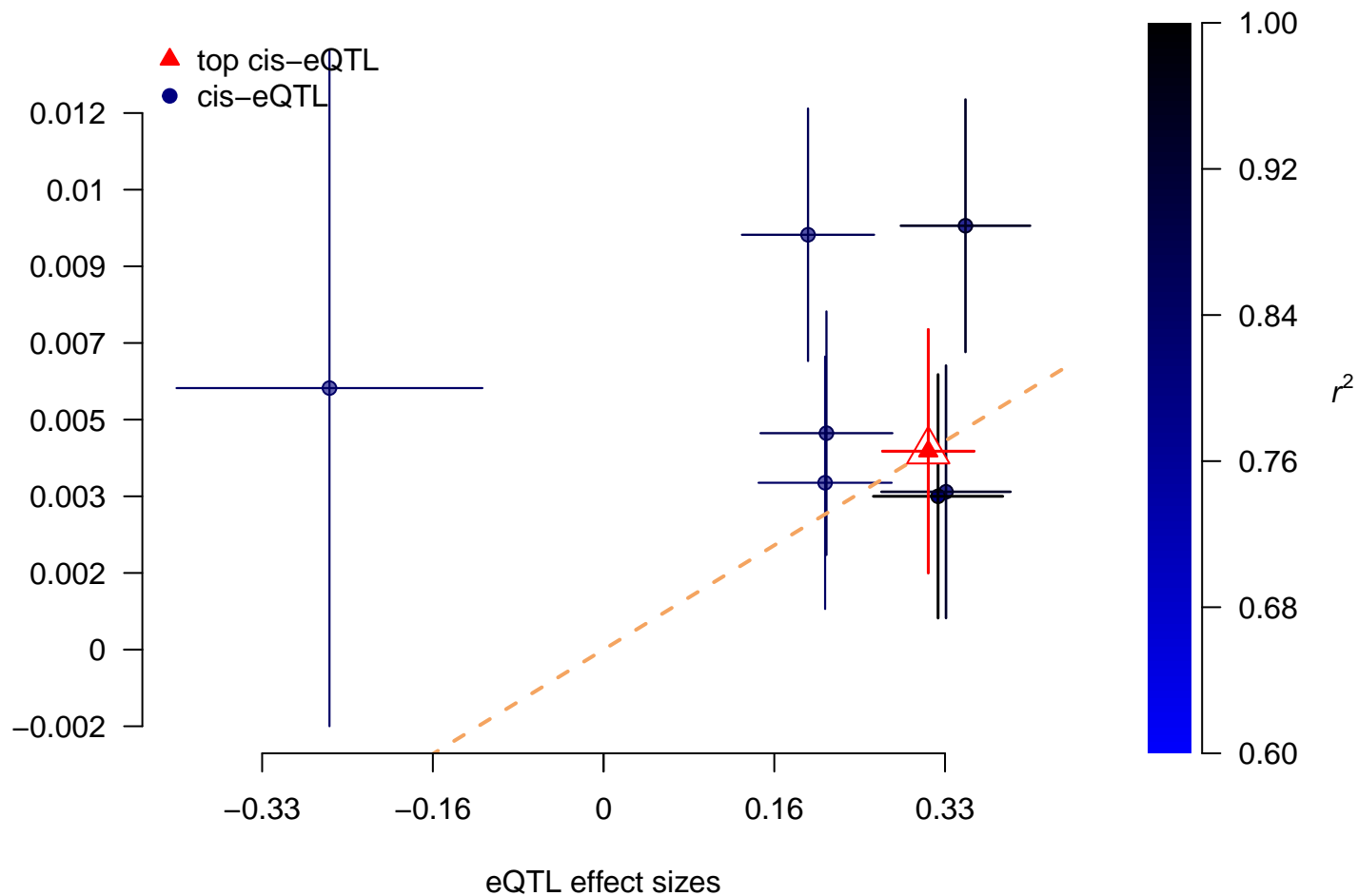

Supplement: Supplementary file 2 — Supplementary Material 2 [file 13568_2025_1969_MOESM2_ESM.zip › Revised supplementary materials/4 Novel loci SMR results/plot/cg26831730_EffectPlot.pdf]

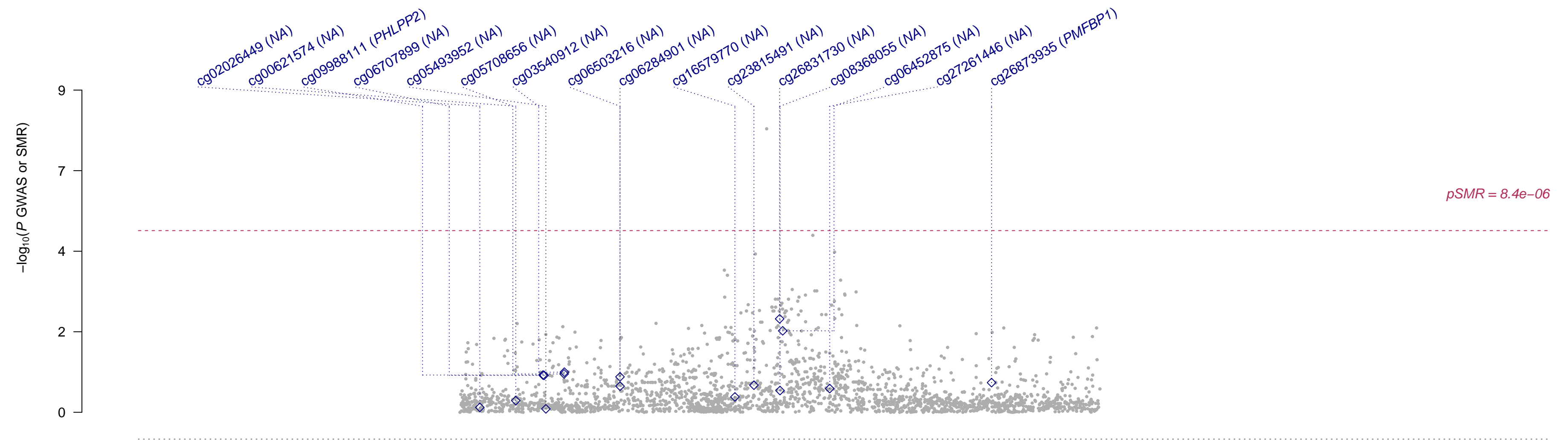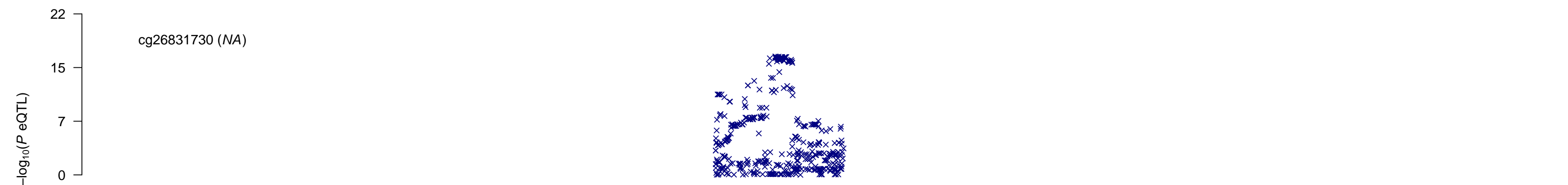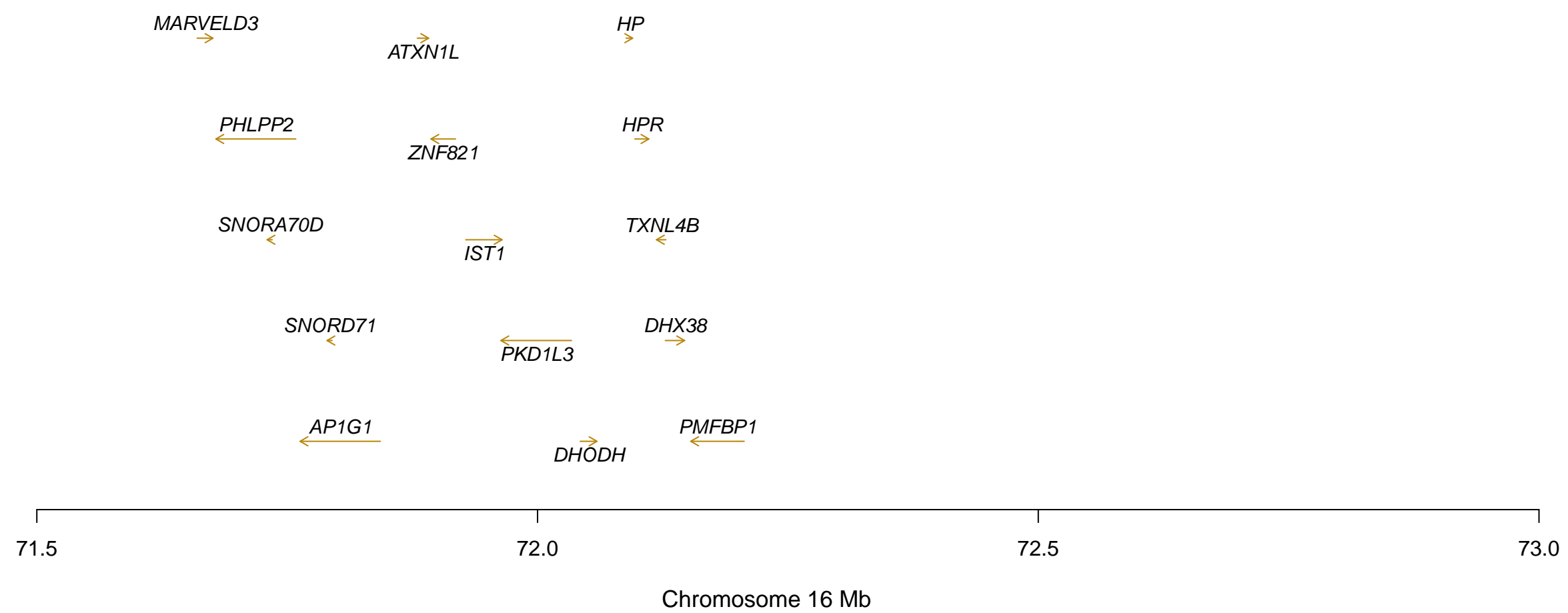

Supplement: Supplementary file 2 — Supplementary Material 2 [file 13568_2025_1969_MOESM2_ESM.zip › Revised supplementary materials/4 Novel loci SMR results/plot/cg26831730_LocusPlot.pdf]

ASD novel loci

ENSG00000153237.18 (*CCDC148*)

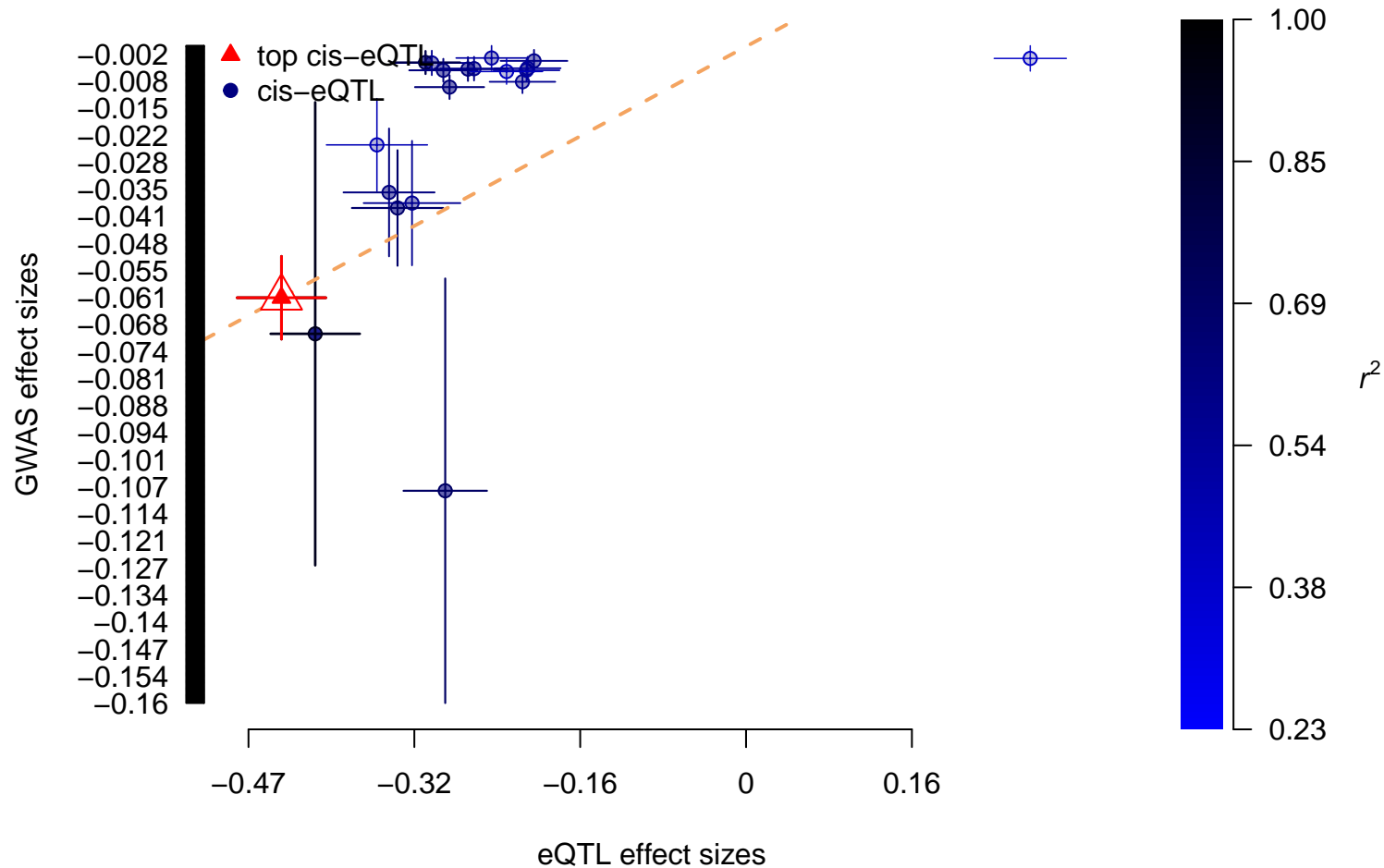

Supplement: Supplementary file 2 — Supplementary Material 2 [file 13568_2025_1969_MOESM2_ESM.zip › Revised supplementary materials/4 Novel loci SMR results/plot/ENSG00000153237.18_EffectPlot.pdf]

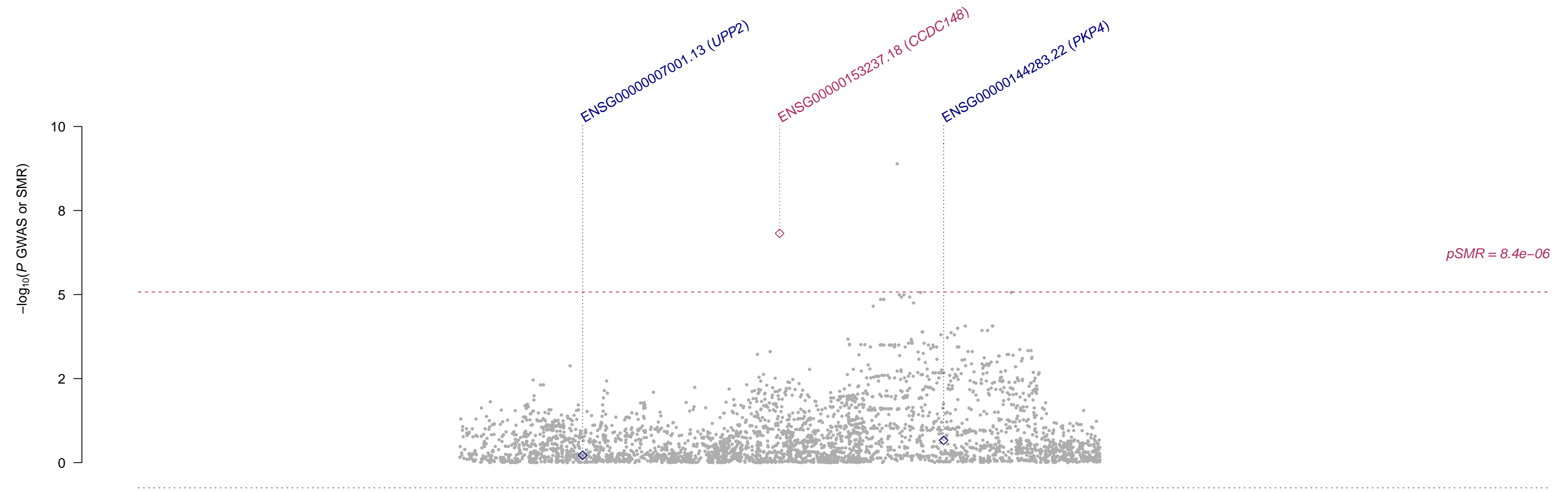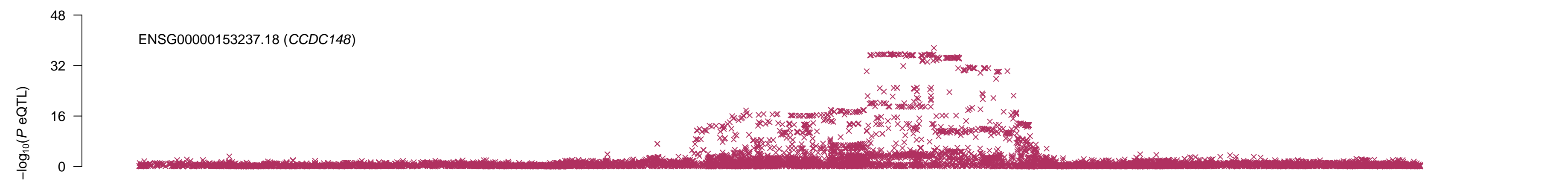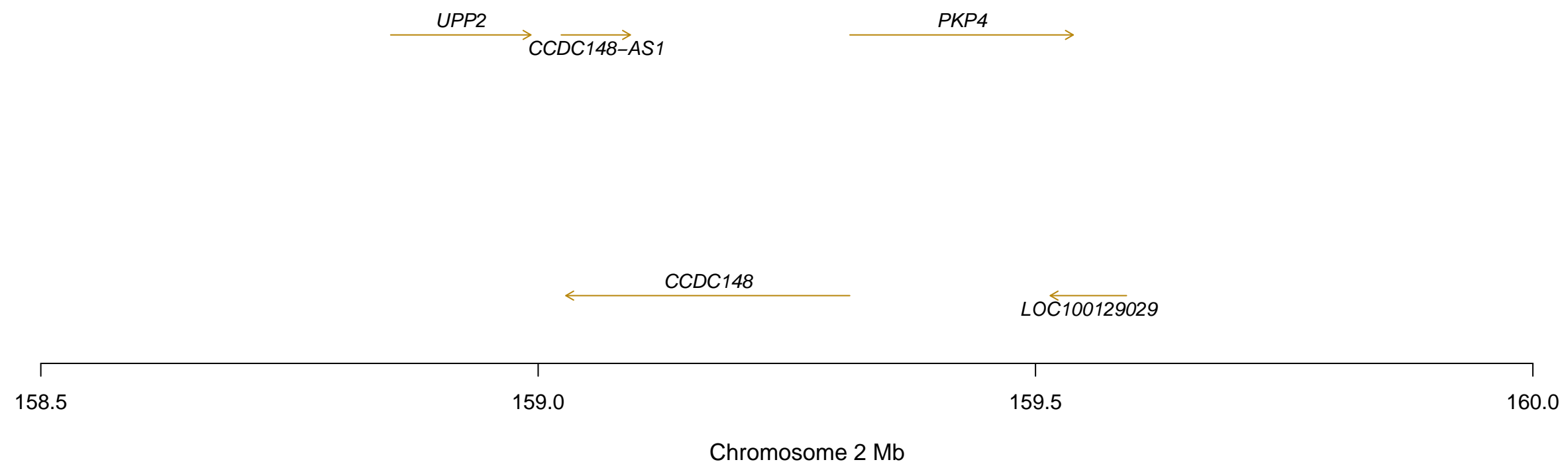

Supplement: Supplementary file 2 — Supplementary Material 2 [file 13568_2025_1969_MOESM2_ESM.zip › Revised supplementary materials/4 Novel loci SMR results/plot/ENSG00000153237.18_LocusPlot.pdf]

ASD novel loci

ENSG00000157578 (*LCA5L*)

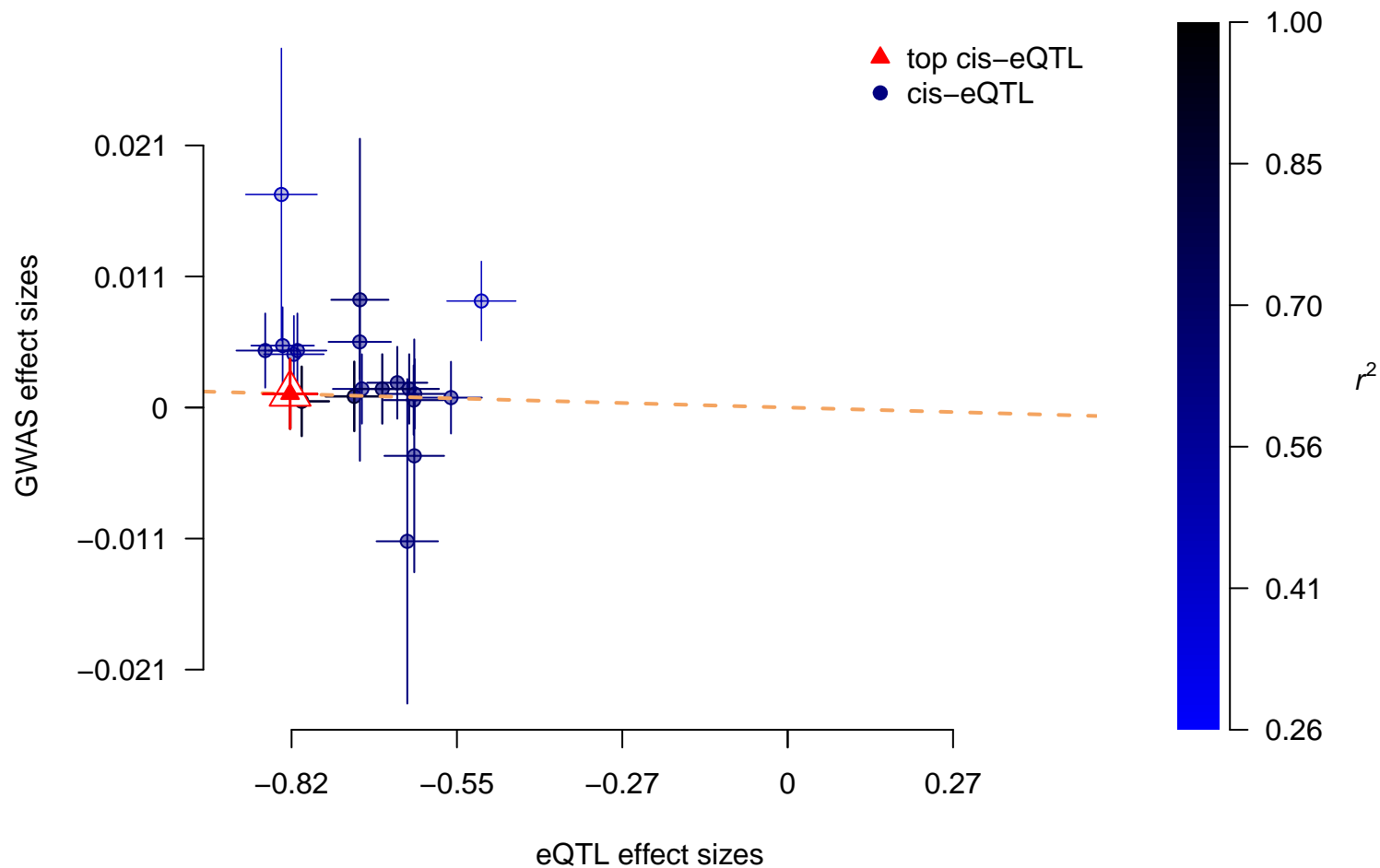

Supplement: Supplementary file 2 — Supplementary Material 2 [file 13568_2025_1969_MOESM2_ESM.zip › Revised supplementary materials/4 Novel loci SMR results/plot/ENSG00000157578_EffectPlot.pdf]

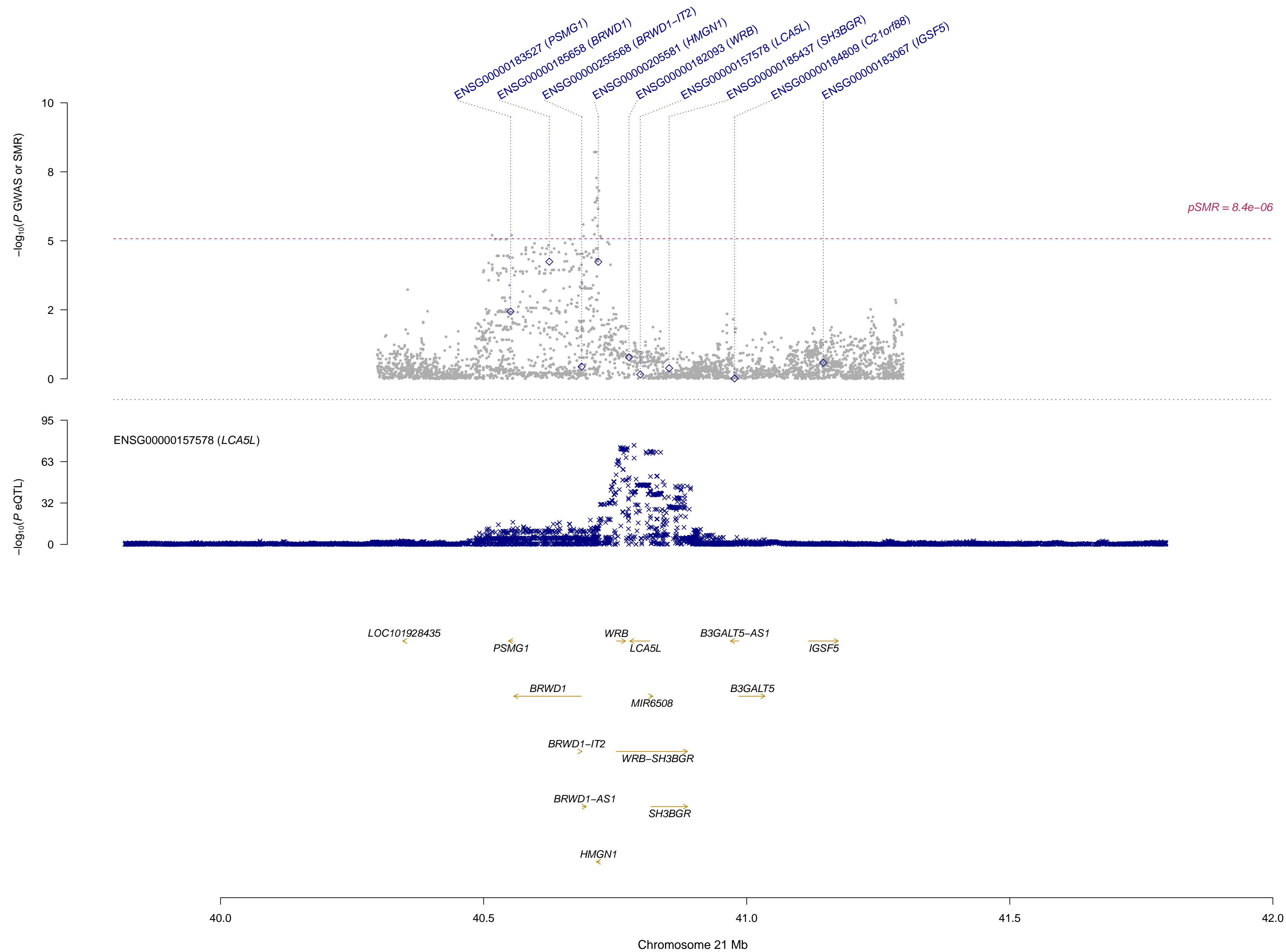

Supplement: Supplementary file 2 — Supplementary Material 2 [file 13568_2025_1969_MOESM2_ESM.zip › Revised supplementary materials/4 Novel loci SMR results/plot/ENSG00000157578_LocusPlot.pdf]

ASD novel loci

ENSG00000158406.5 (*H4C8*)

▲ top cis-eQTL

● cis-eQTL

GWAS effect sizes

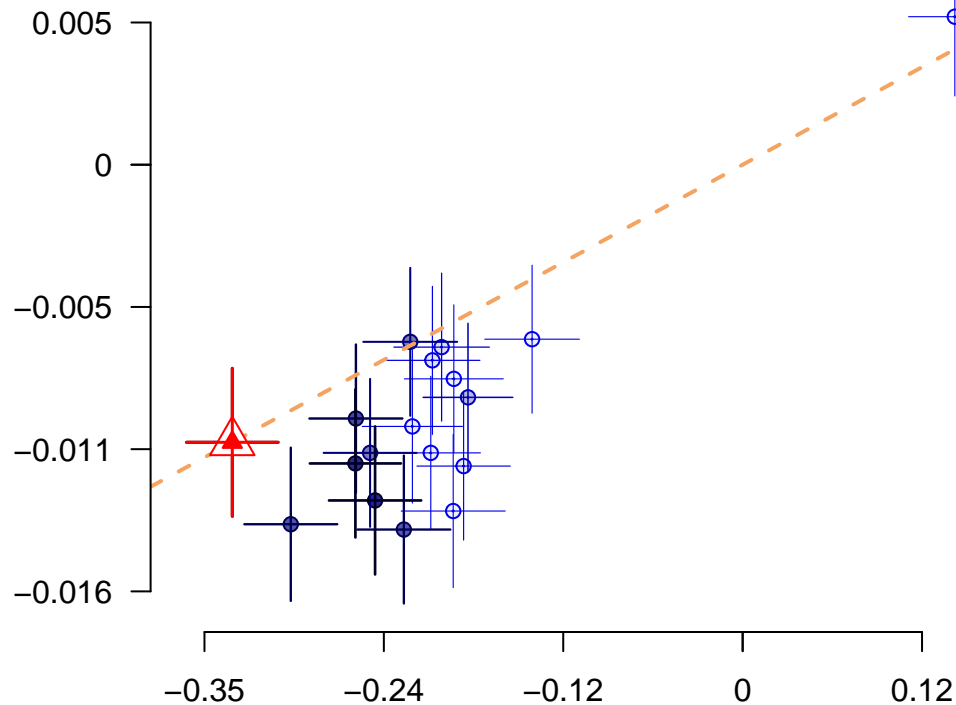

$r^2$

1.00

0.81

0.62

0.43

0.24

0.06

Supplement: Supplementary file 2 — Supplementary Material 2 [file 13568_2025_1969_MOESM2_ESM.zip › Revised supplementary materials/4 Novel loci SMR results/plot/ENSG00000158406.5_EffectPlot.pdf]

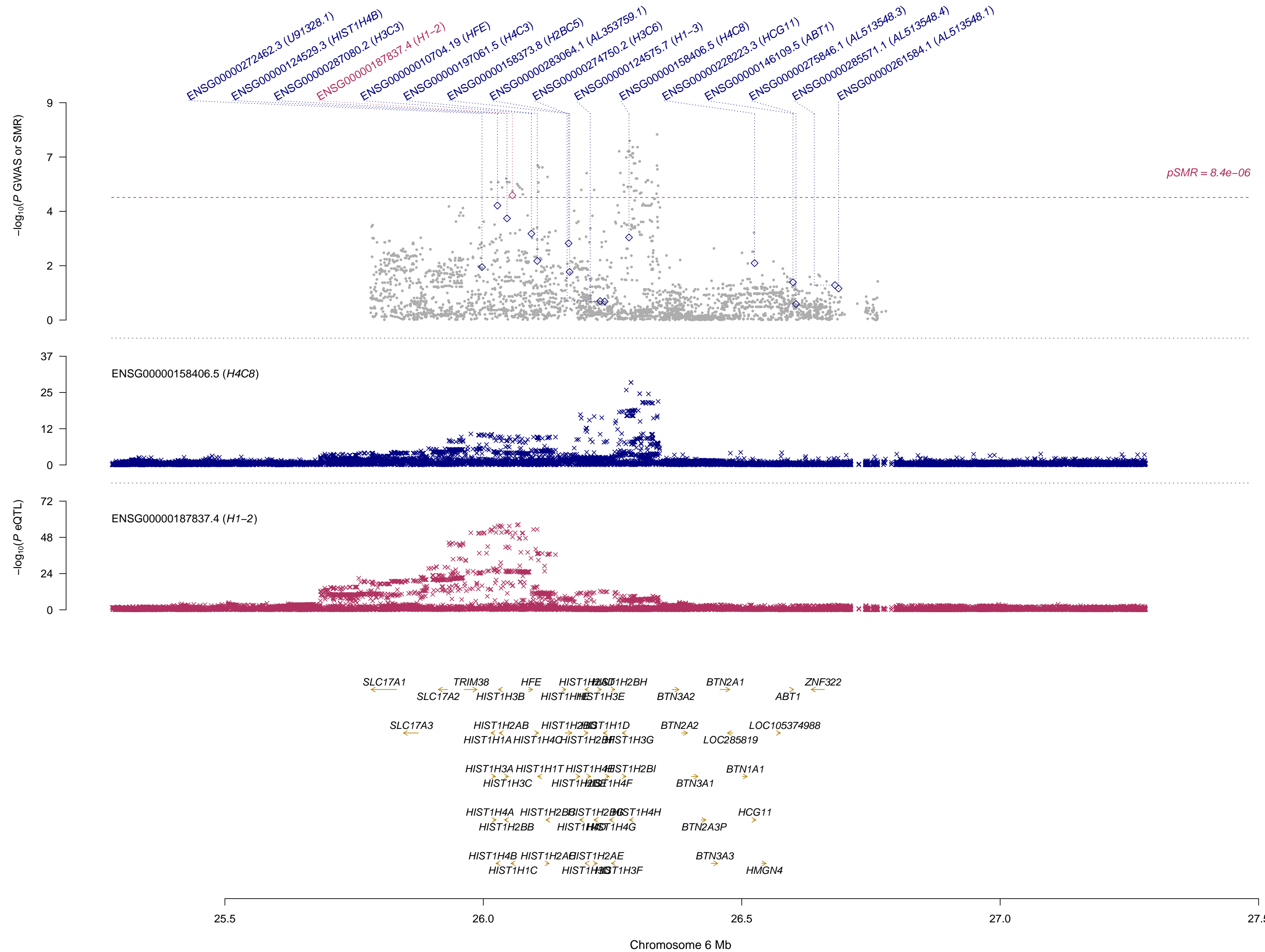

Supplement: Supplementary file 2 — Supplementary Material 2 [file 13568_2025_1969_MOESM2_ESM.zip › Revised supplementary materials/4 Novel loci SMR results/plot/ENSG00000158406.5_LocusPlot.pdf]

ASD novel loci

ENSG00000173295.8 (*FAM86B3P*)

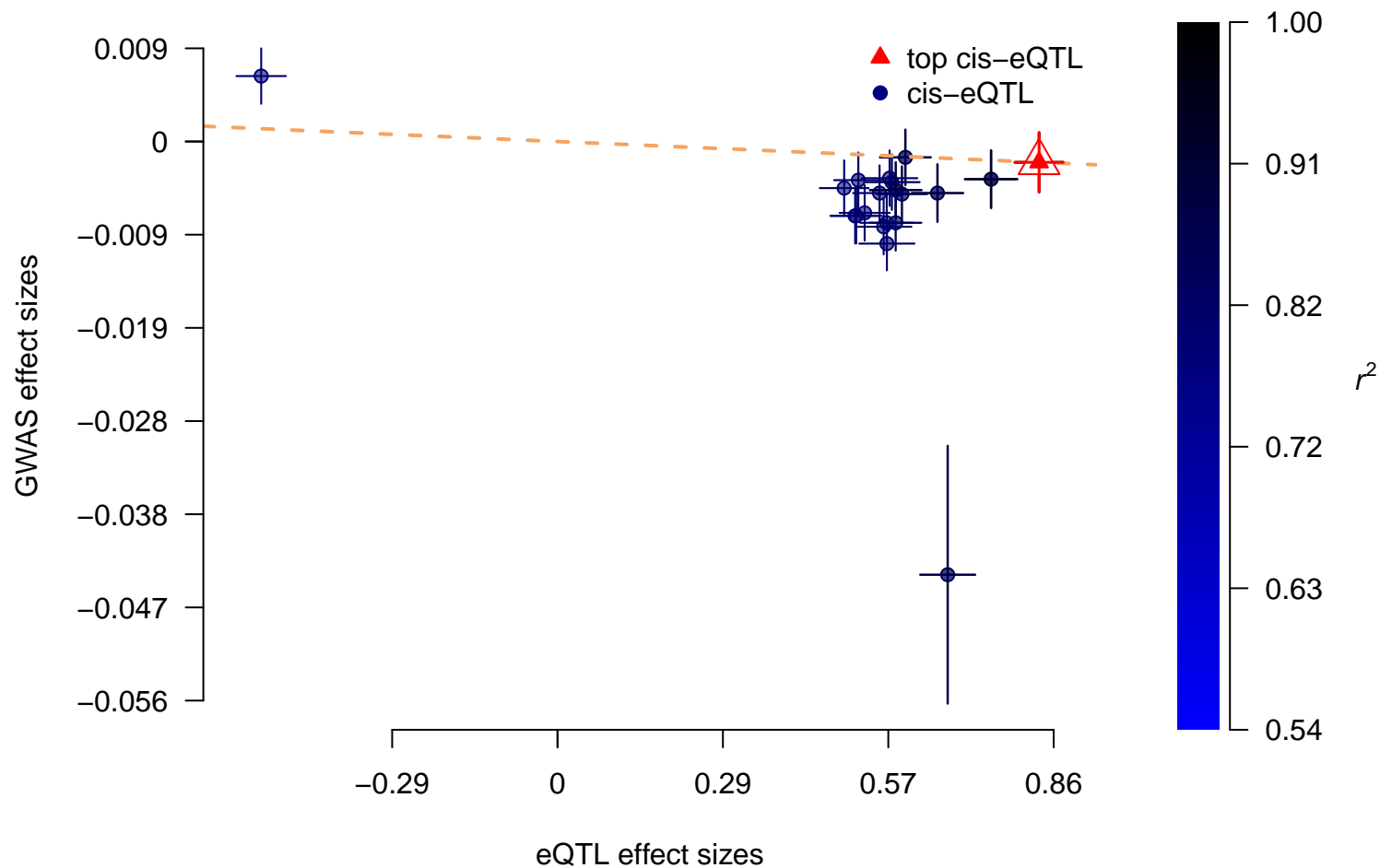

Supplement: Supplementary file 2 — Supplementary Material 2 [file 13568_2025_1969_MOESM2_ESM.zip › Revised supplementary materials/4 Novel loci SMR results/plot/ENSG00000173295.8_EffectPlot.pdf]

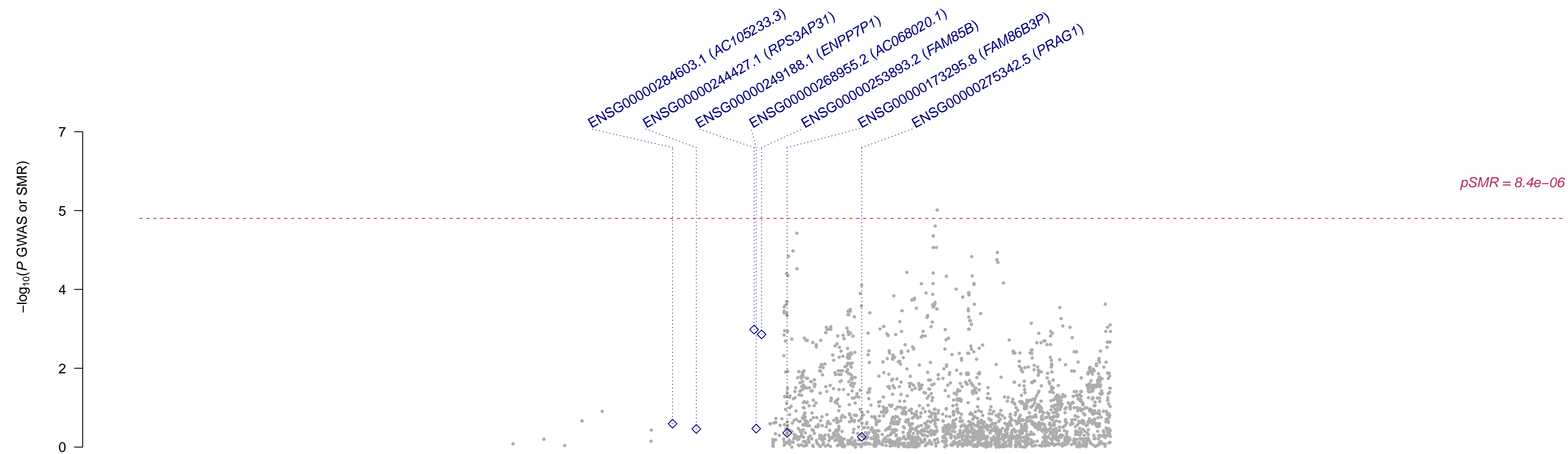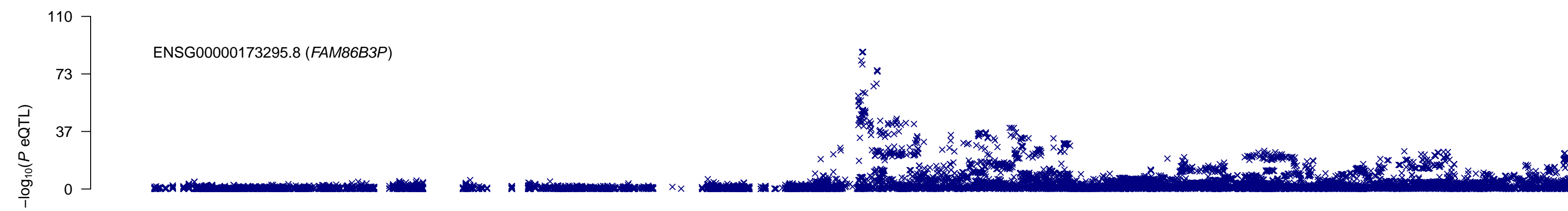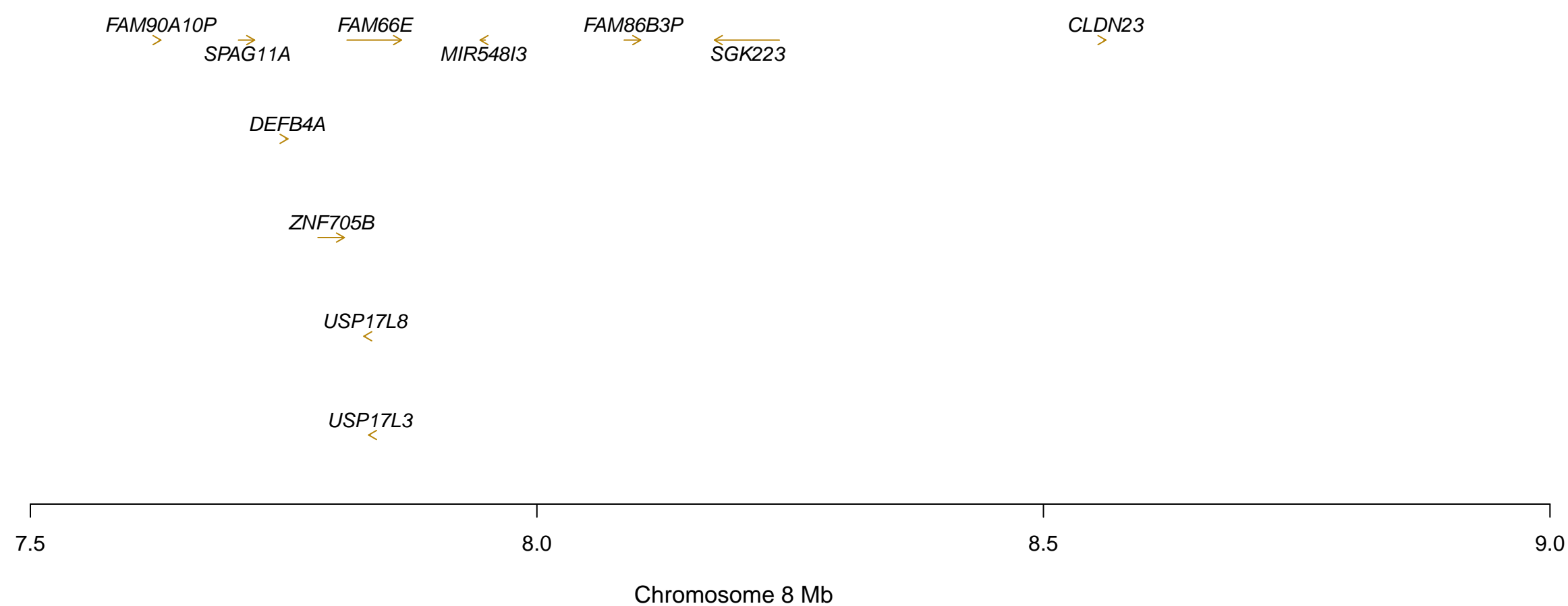

Supplement: Supplementary file 2 — Supplementary Material 2 [file 13568_2025_1969_MOESM2_ESM.zip › Revised supplementary materials/4 Novel loci SMR results/plot/ENSG00000173295.8_LocusPlot.pdf]

ENSG00000182093 (*WRB*)

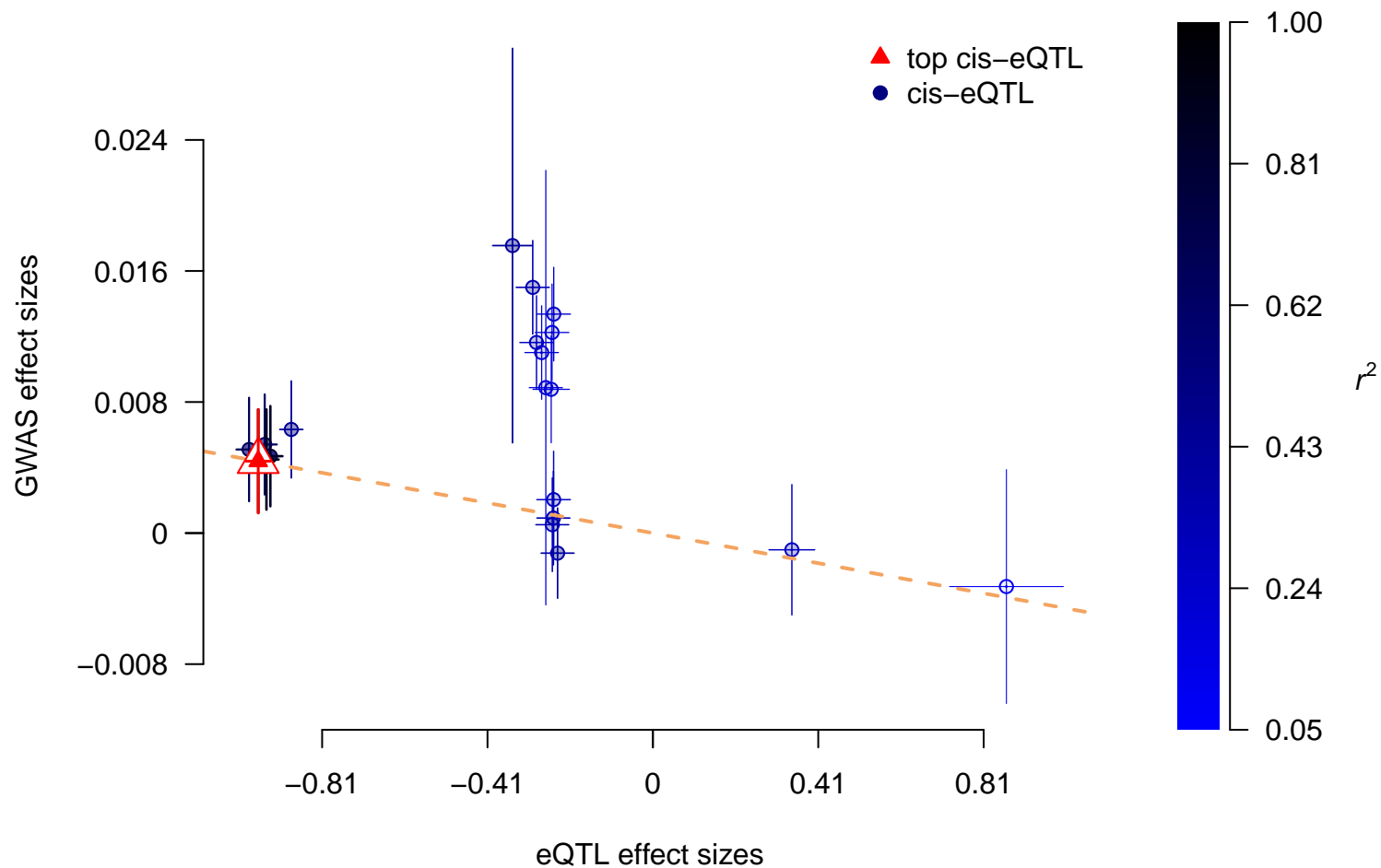

Supplement: Supplementary file 2 — Supplementary Material 2 [file 13568_2025_1969_MOESM2_ESM.zip › Revised supplementary materials/4 Novel loci SMR results/plot/ENSG00000182093_EffectPlot.pdf]

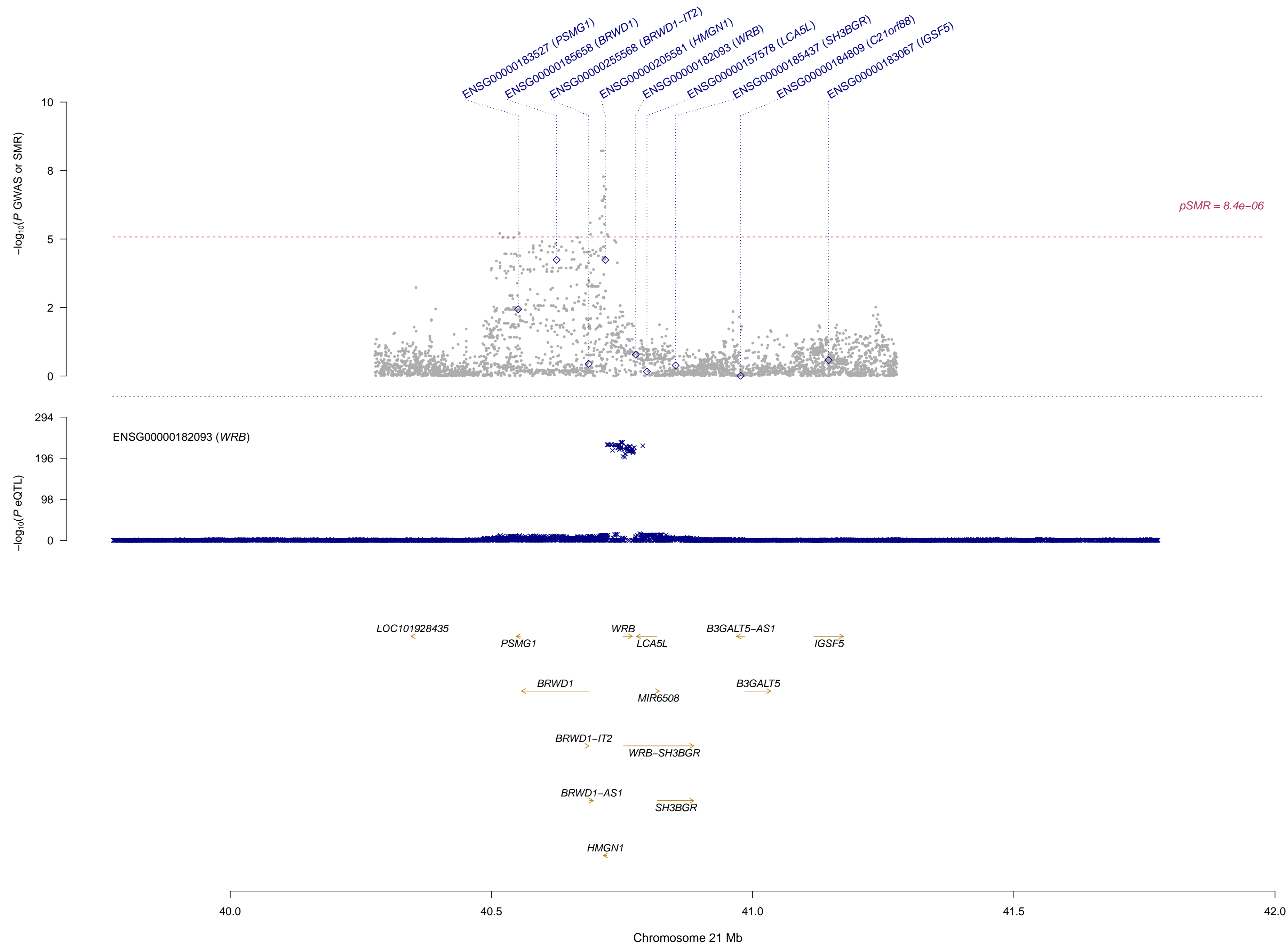

Supplement: Supplementary file 2 — Supplementary Material 2 [file 13568_2025_1969_MOESM2_ESM.zip › Revised supplementary materials/4 Novel loci SMR results/plot/ENSG00000182093_LocusPlot.pdf]

ASD novel loci

ENSG00000183527 (*PSMG1*)

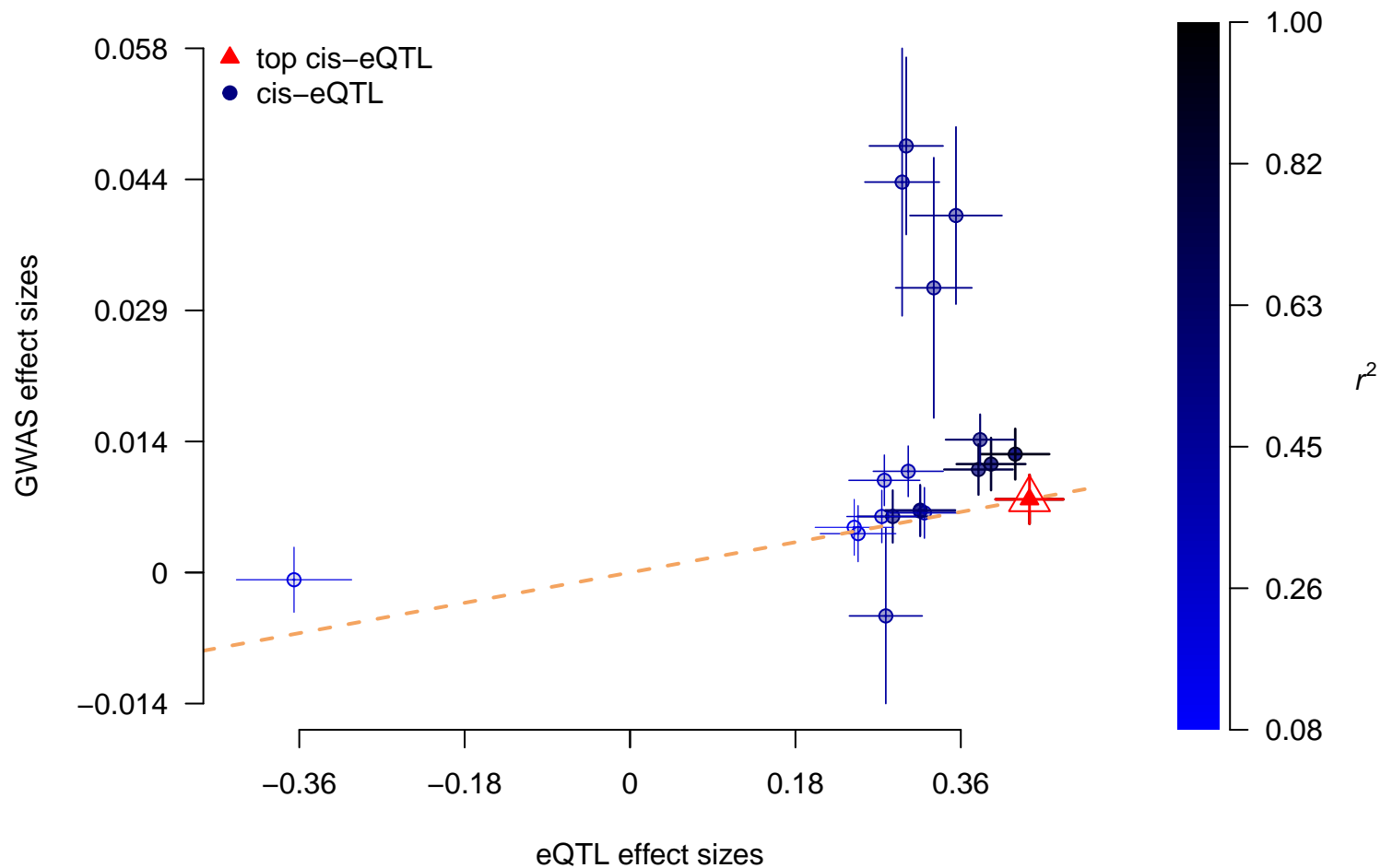

Supplement: Supplementary file 2 — Supplementary Material 2 [file 13568_2025_1969_MOESM2_ESM.zip › Revised supplementary materials/4 Novel loci SMR results/plot/ENSG00000183527_EffectPlot.pdf]

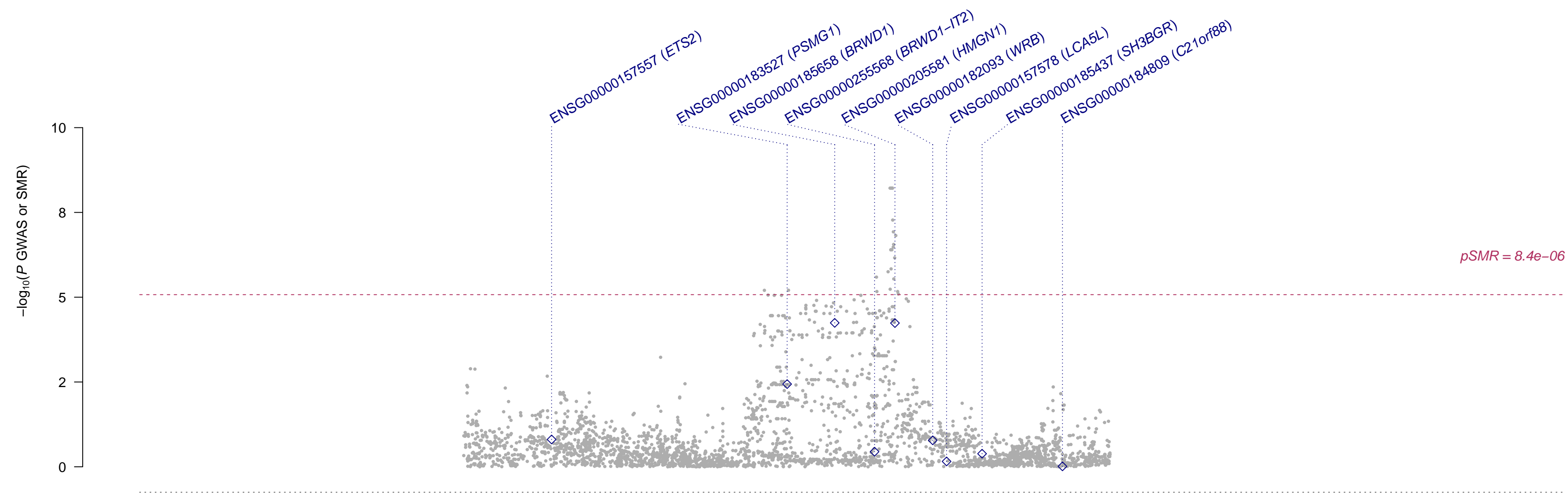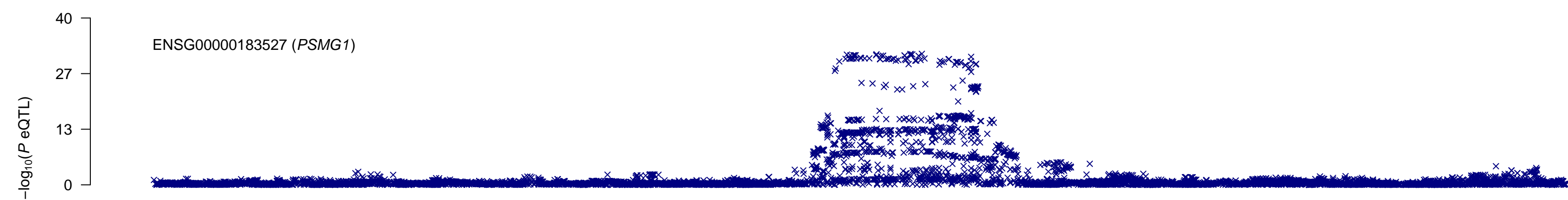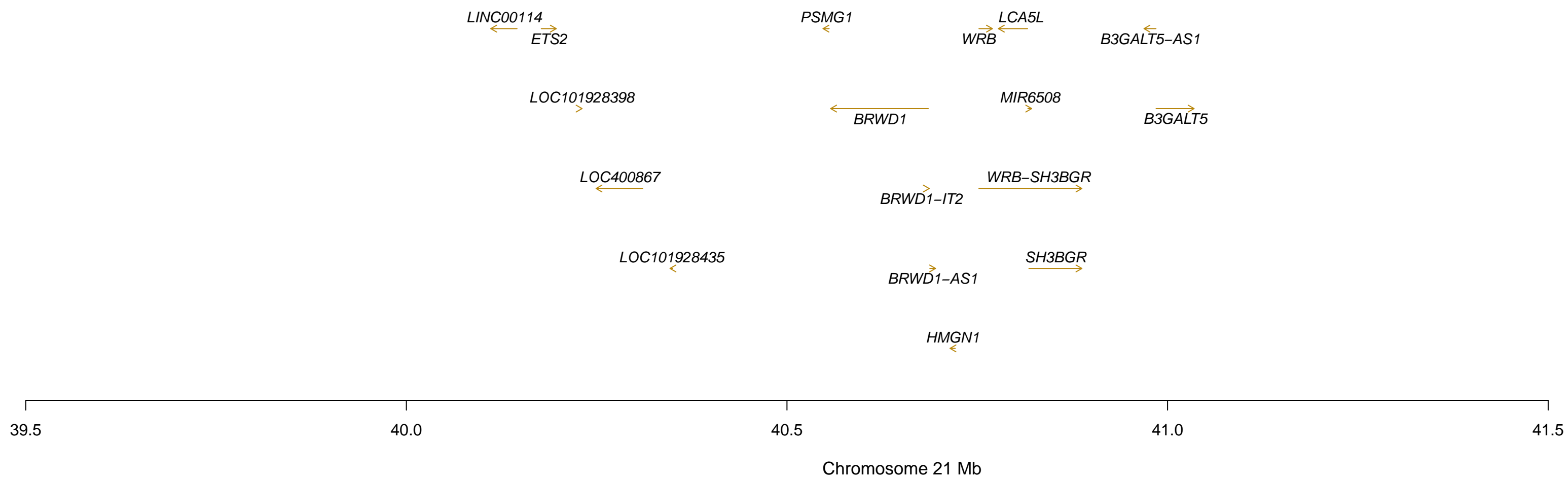

Supplement: Supplementary file 2 — Supplementary Material 2 [file 13568_2025_1969_MOESM2_ESM.zip › Revised supplementary materials/4 Novel loci SMR results/plot/ENSG00000183527_LocusPlot.pdf]

ENSG00000185658 (*BRWD1*)

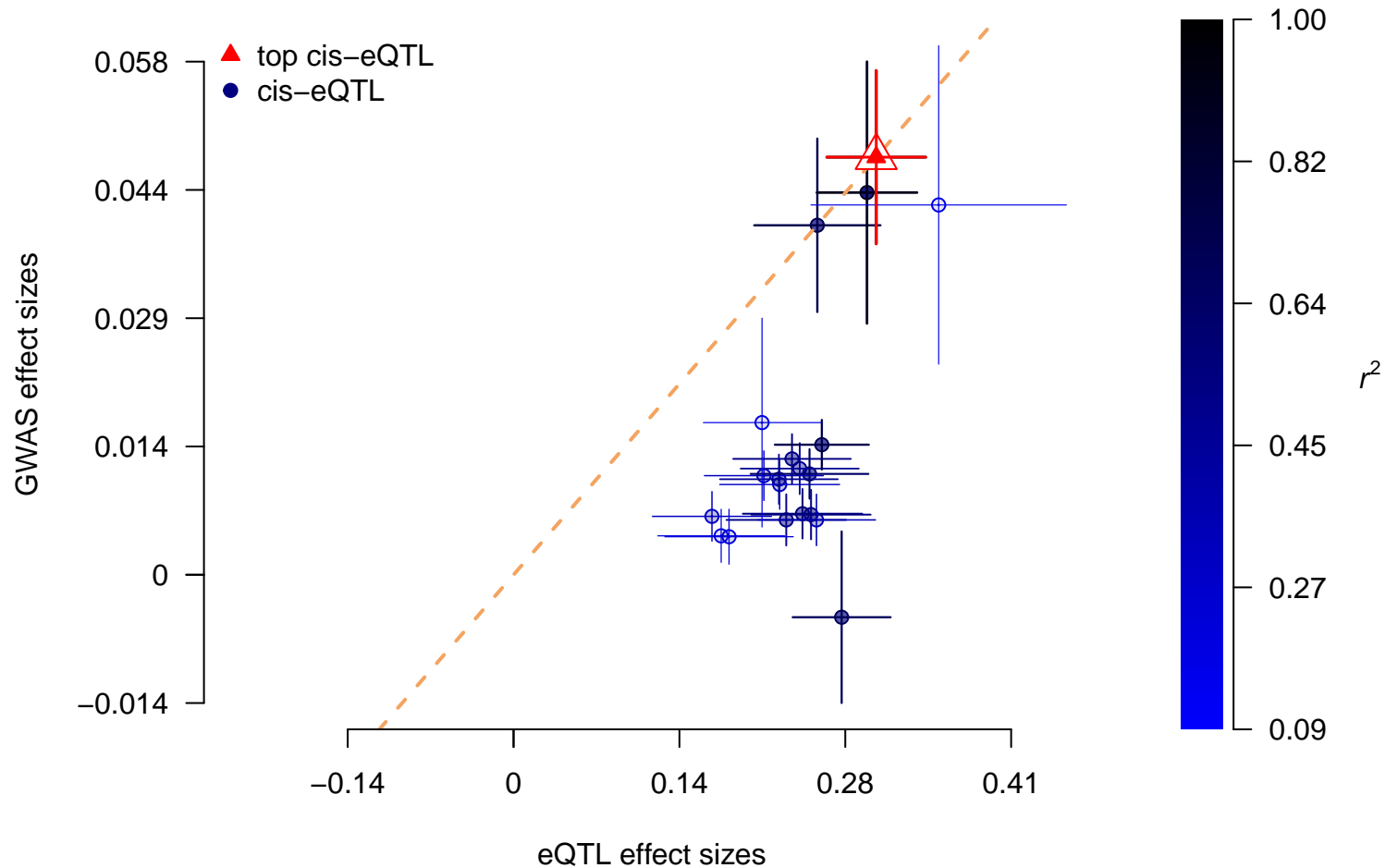

Supplement: Supplementary file 2 — Supplementary Material 2 [file 13568_2025_1969_MOESM2_ESM.zip › Revised supplementary materials/4 Novel loci SMR results/plot/ENSG00000185658_EffectPlot.pdf]

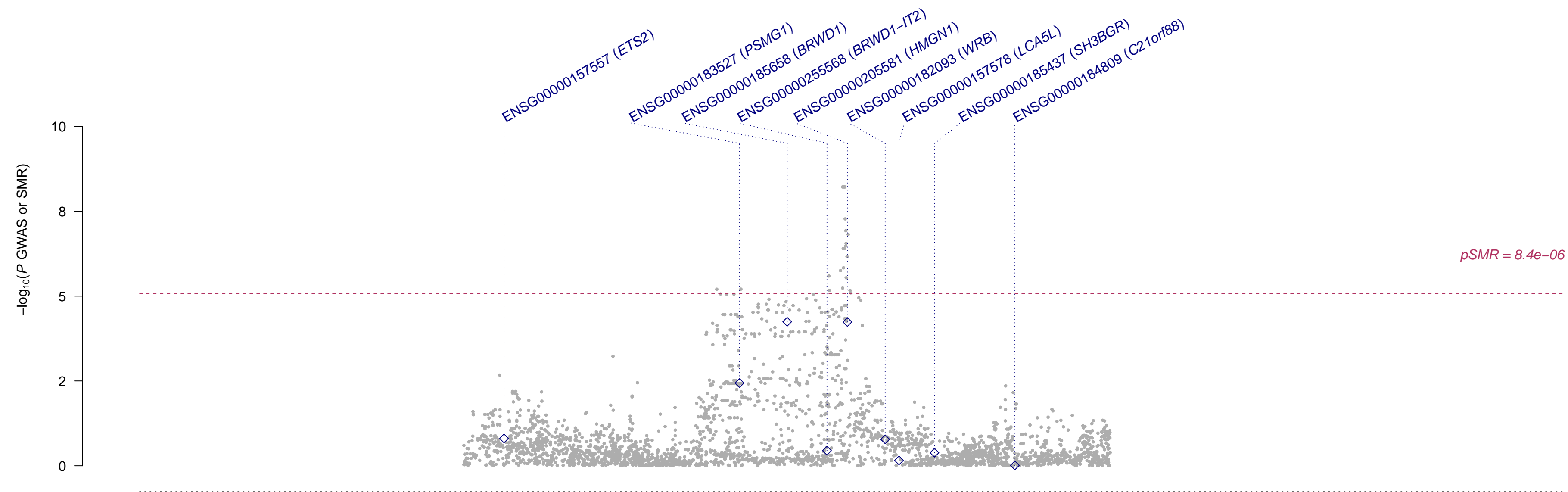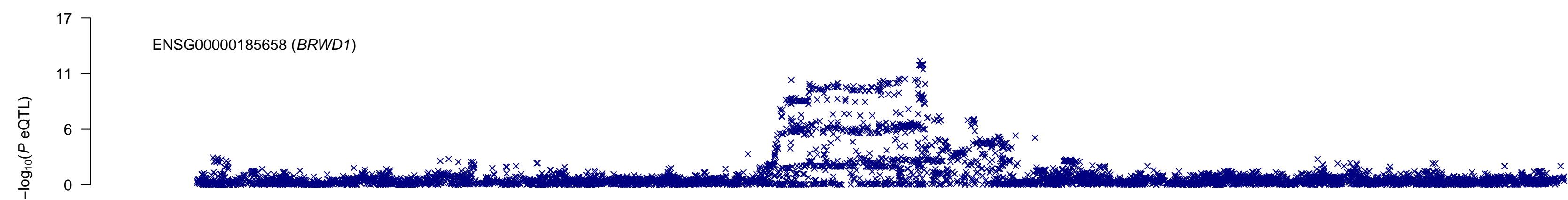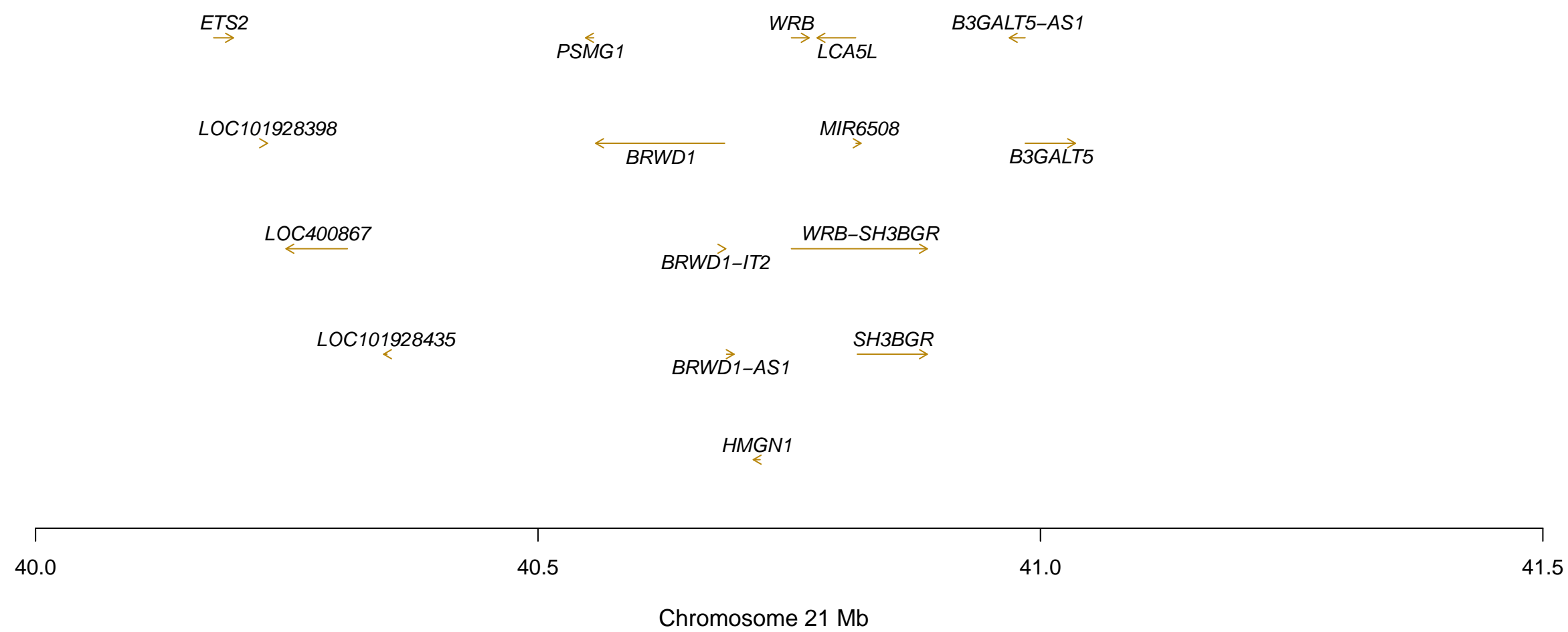

Supplement: Supplementary file 2 — Supplementary Material 2 [file 13568_2025_1969_MOESM2_ESM.zip › Revised supplementary materials/4 Novel loci SMR results/plot/ENSG00000185658_LocusPlot.pdf]

ASD novel loci

ENSG00000205581 (*HMGN1*)

▲ top cis-eQTL

● cis-eQTL

GWAS effect sizes

0.045  
0.022  
0  
-0.022  
-0.045

-0.47 -0.24 0 0.24 0.47

eQTL effect sizes

$r^2$

1.00

0.83

0.66

0.49

0.32

0.14

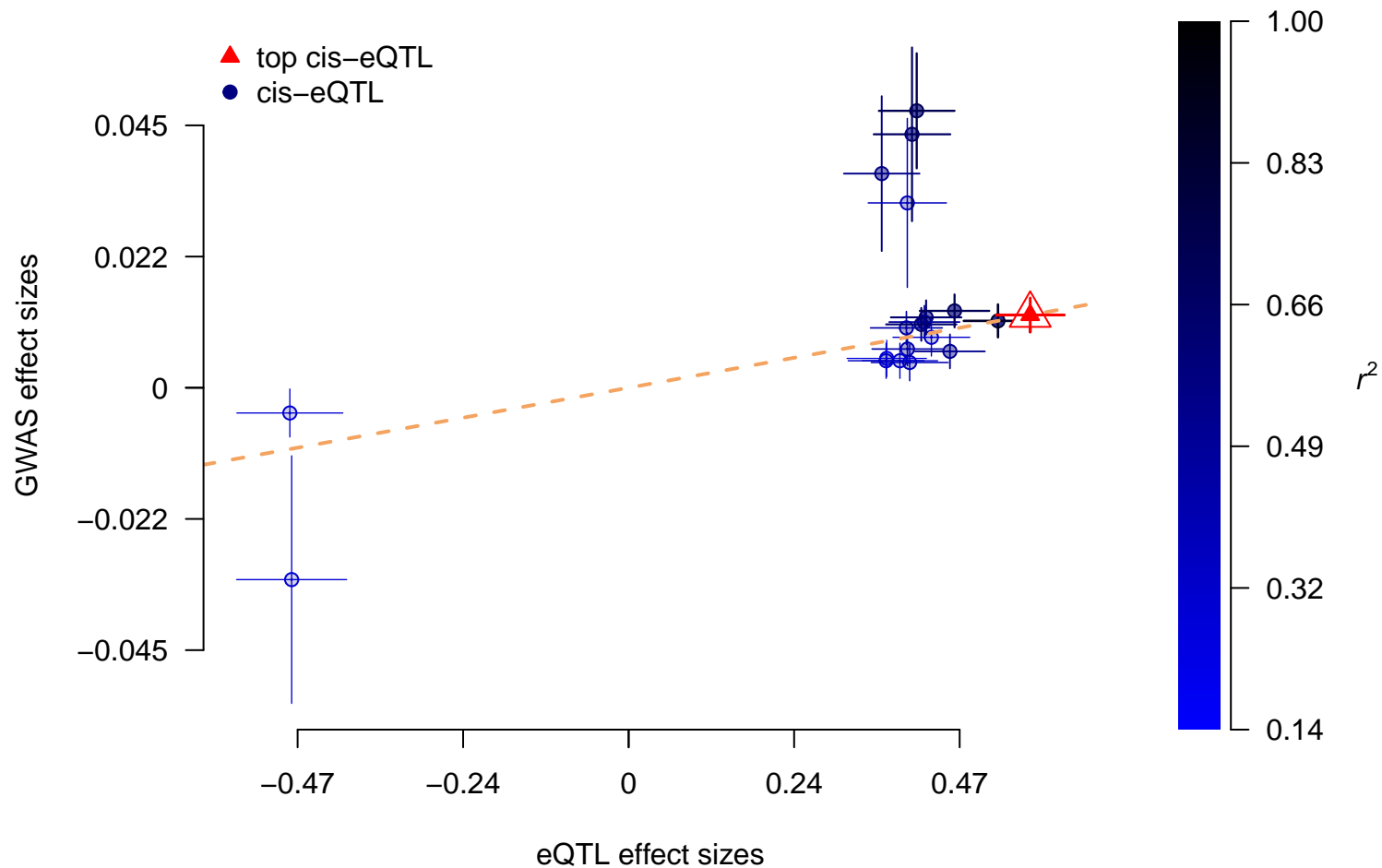

Supplement: Supplementary file 2 — Supplementary Material 2 [file 13568_2025_1969_MOESM2_ESM.zip › Revised supplementary materials/4 Novel loci SMR results/plot/ENSG00000205581_EffectPlot.pdf]

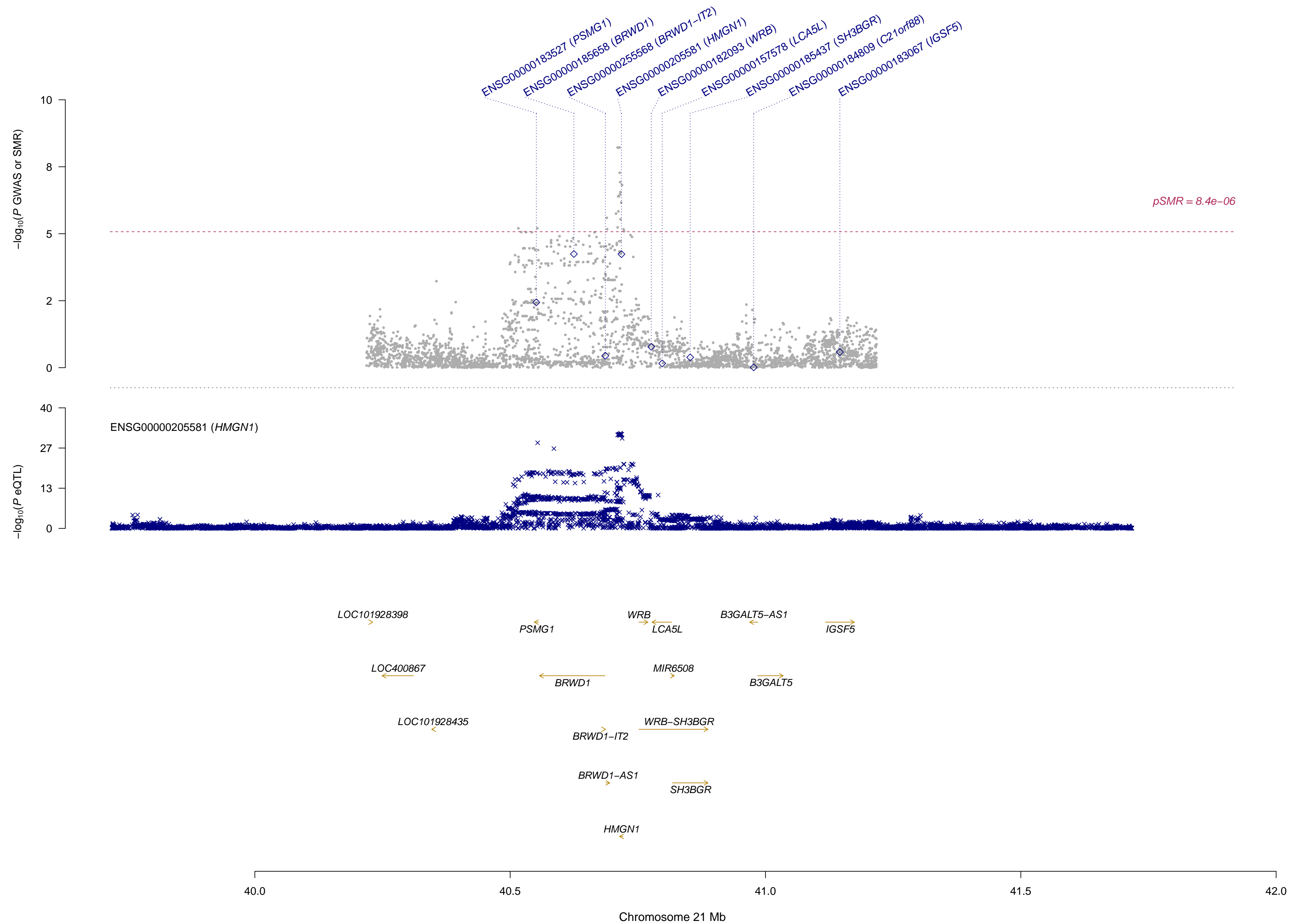

Supplement: Supplementary file 2 — Supplementary Material 2 [file 13568_2025_1969_MOESM2_ESM.zip › Revised supplementary materials/4 Novel loci SMR results/plot/ENSG00000205581_LocusPlot.pdf]

ASD novel loci

ENSG00000233609.3 (*RPL10P19*)

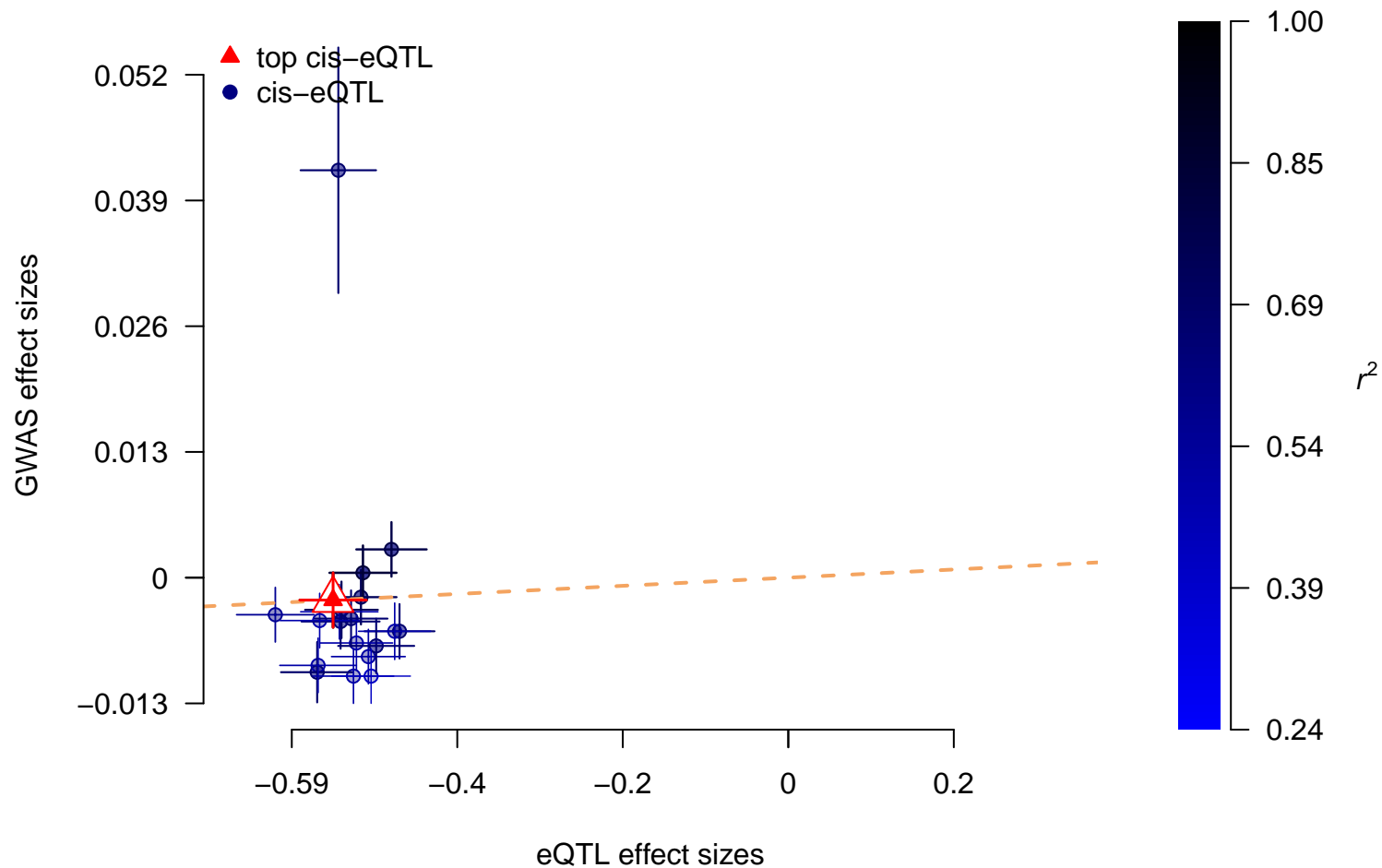

Supplement: Supplementary file 2 — Supplementary Material 2 [file 13568_2025_1969_MOESM2_ESM.zip › Revised supplementary materials/4 Novel loci SMR results/plot/ENSG00000233609.3_EffectPlot.pdf]

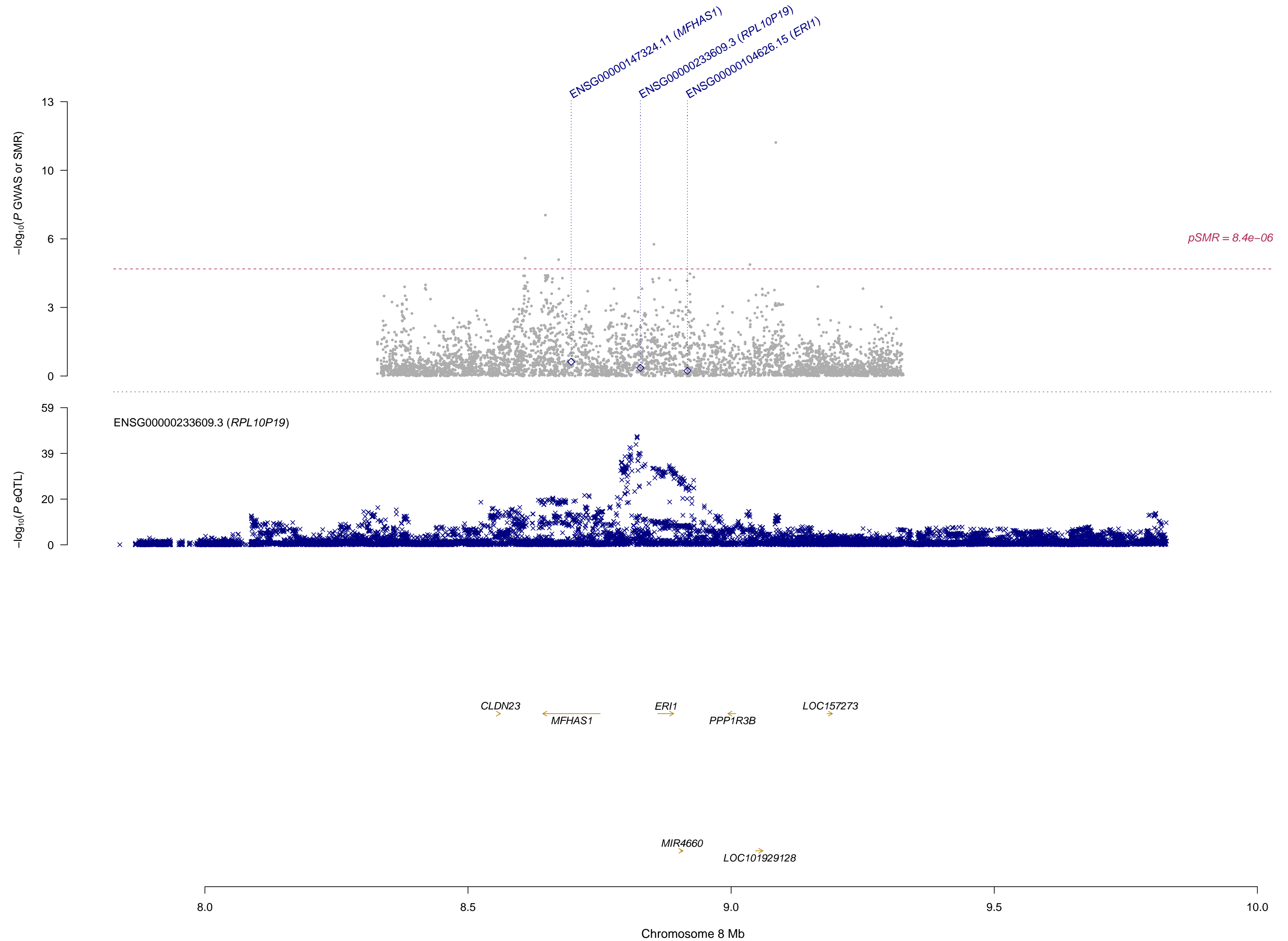

Supplement: Supplementary file 2 — Supplementary Material 2 [file 13568_2025_1969_MOESM2_ESM.zip › Revised supplementary materials/4 Novel loci SMR results/plot/ENSG00000233609.3_LocusPlot.pdf]

ASD novel loci

ENSG00000253893.2 (*FAM85B*)

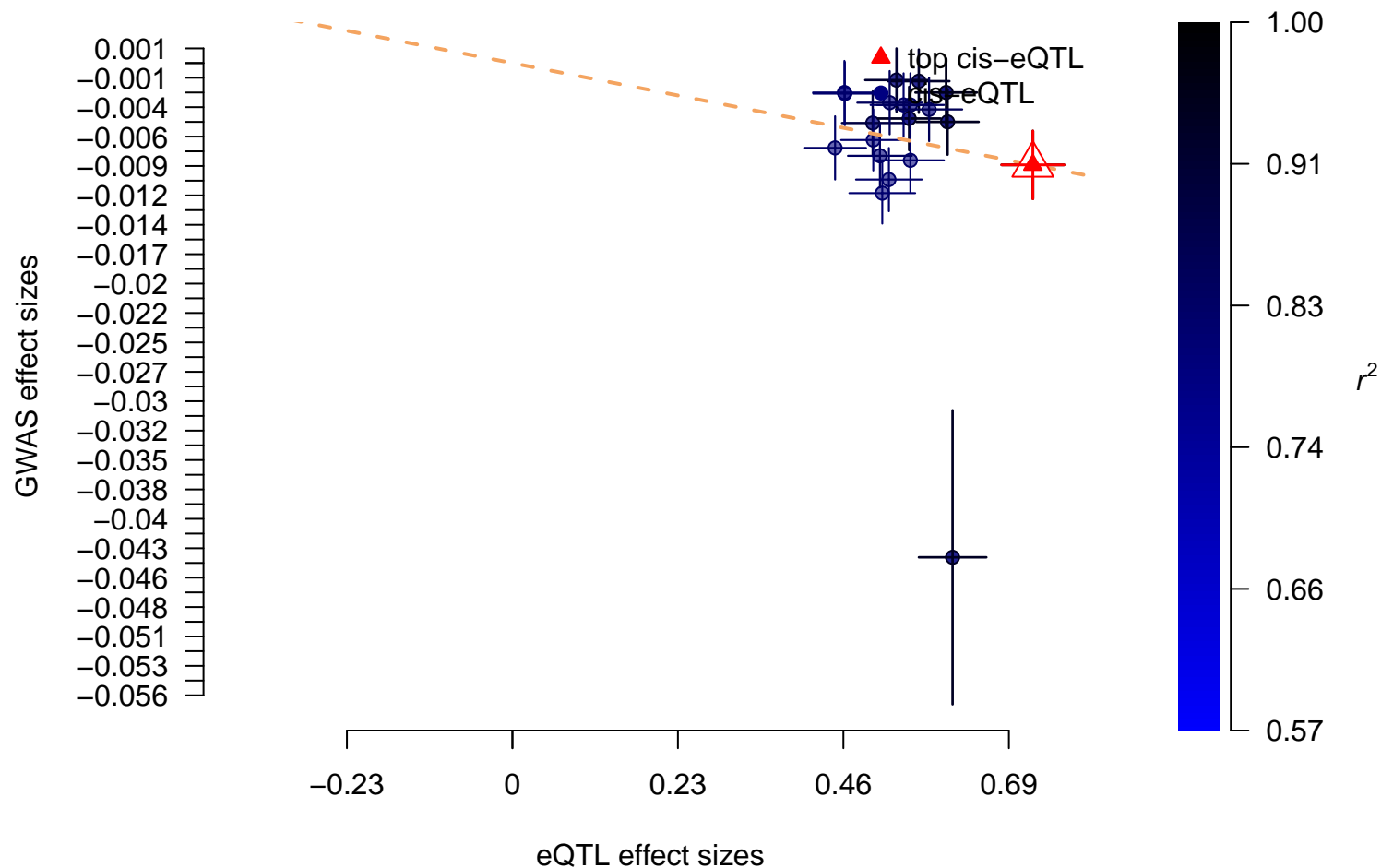

Supplement: Supplementary file 2 — Supplementary Material 2 [file 13568_2025_1969_MOESM2_ESM.zip › Revised supplementary materials/4 Novel loci SMR results/plot/ENSG00000253893.2_EffectPlot.pdf]

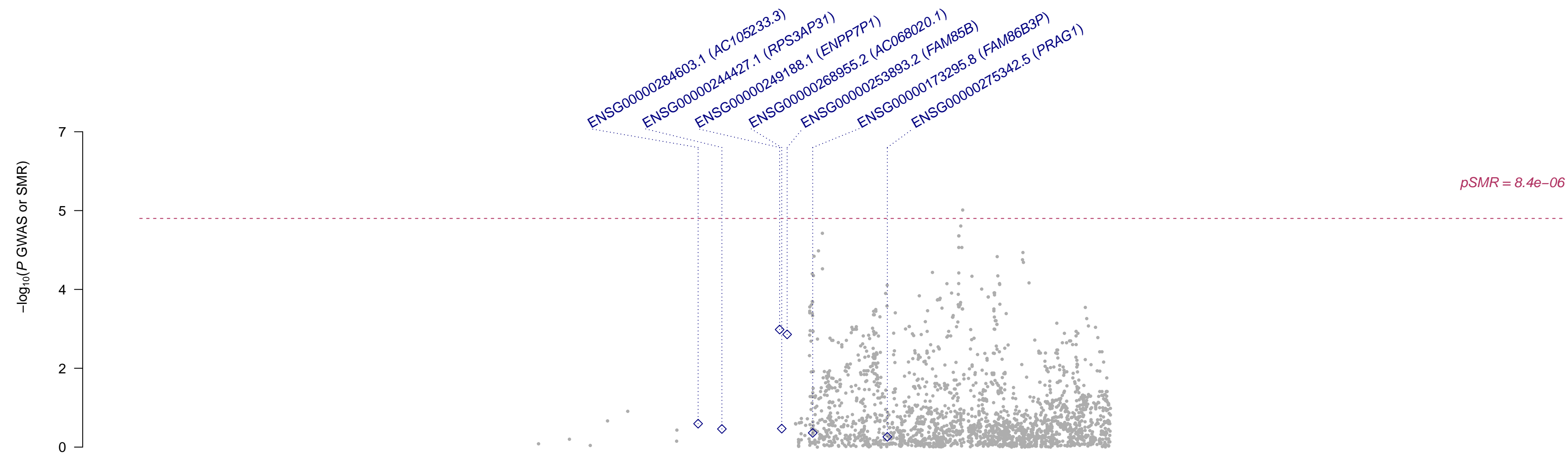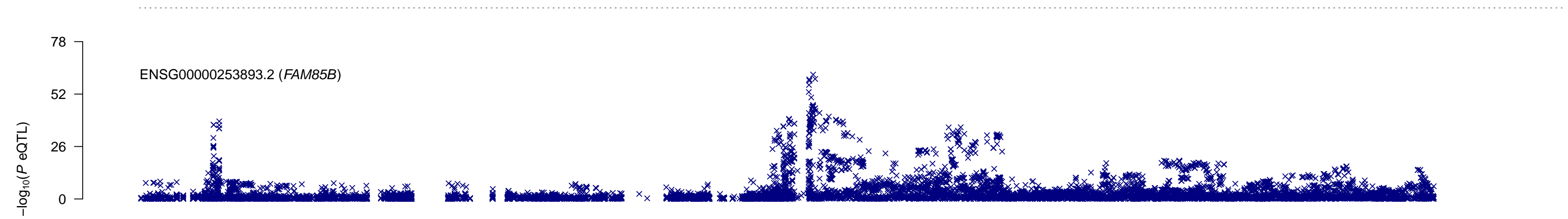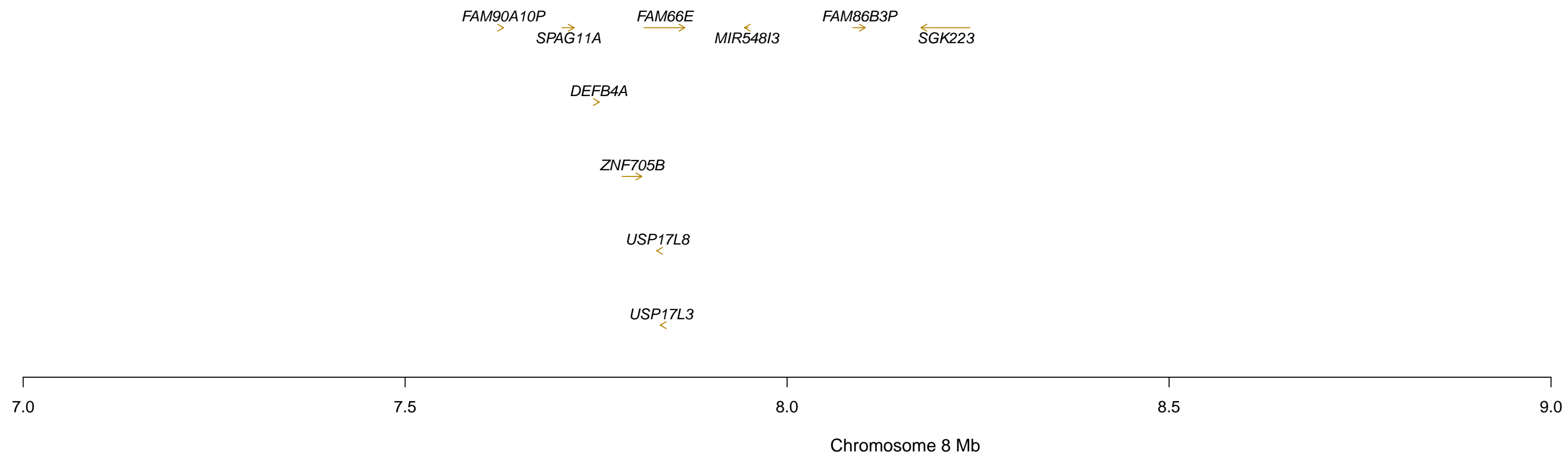

Supplement: Supplementary file 2 — Supplementary Material 2 [file 13568_2025_1969_MOESM2_ESM.zip › Revised supplementary materials/4 Novel loci SMR results/plot/ENSG00000253893.2_LocusPlot.pdf]

ASD novel loci

ENSG00000257017.4 (*HP*)

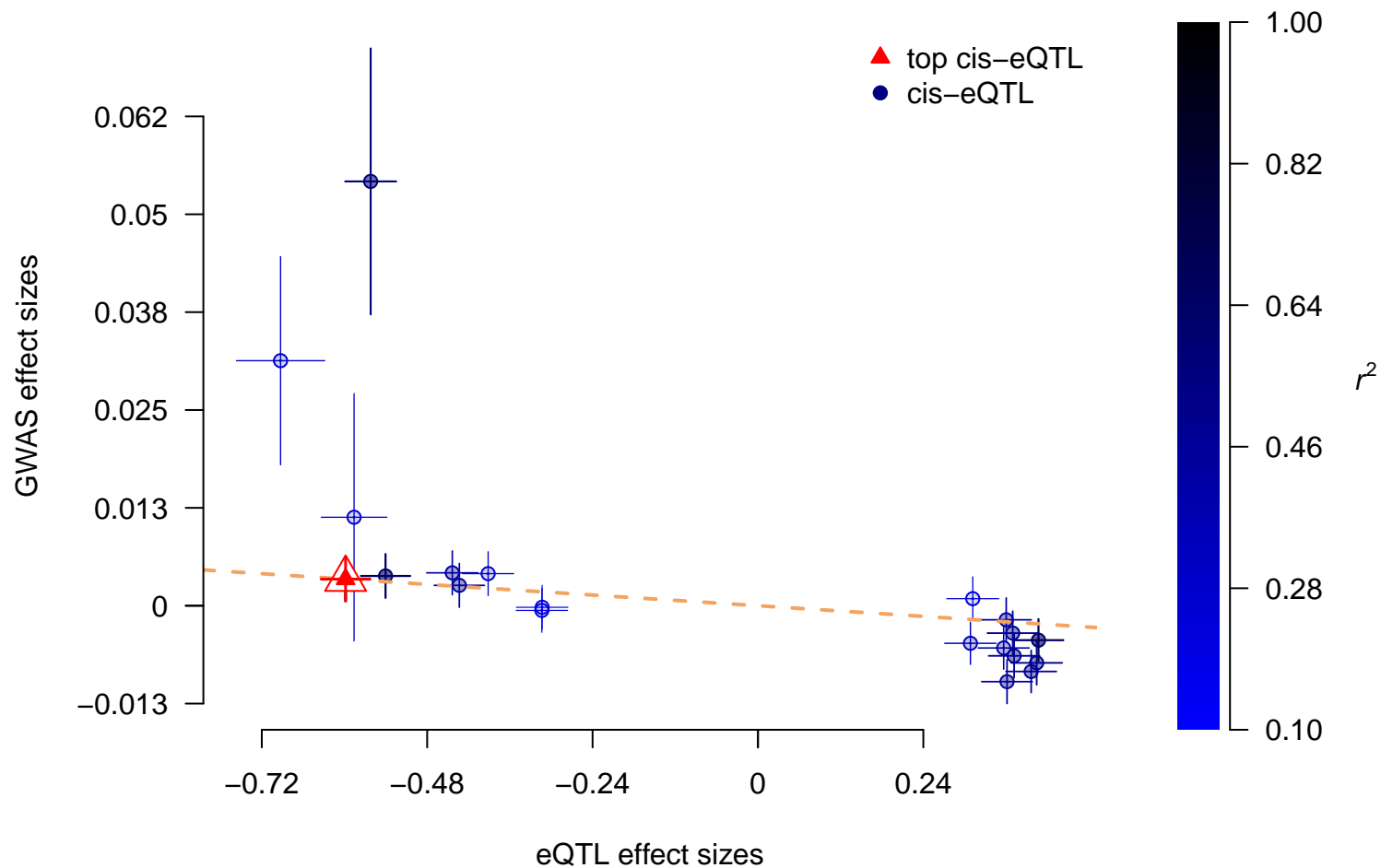

Supplement: Supplementary file 2 — Supplementary Material 2 [file 13568_2025_1969_MOESM2_ESM.zip › Revised supplementary materials/4 Novel loci SMR results/plot/ENSG00000257017.4_EffectPlot.pdf]

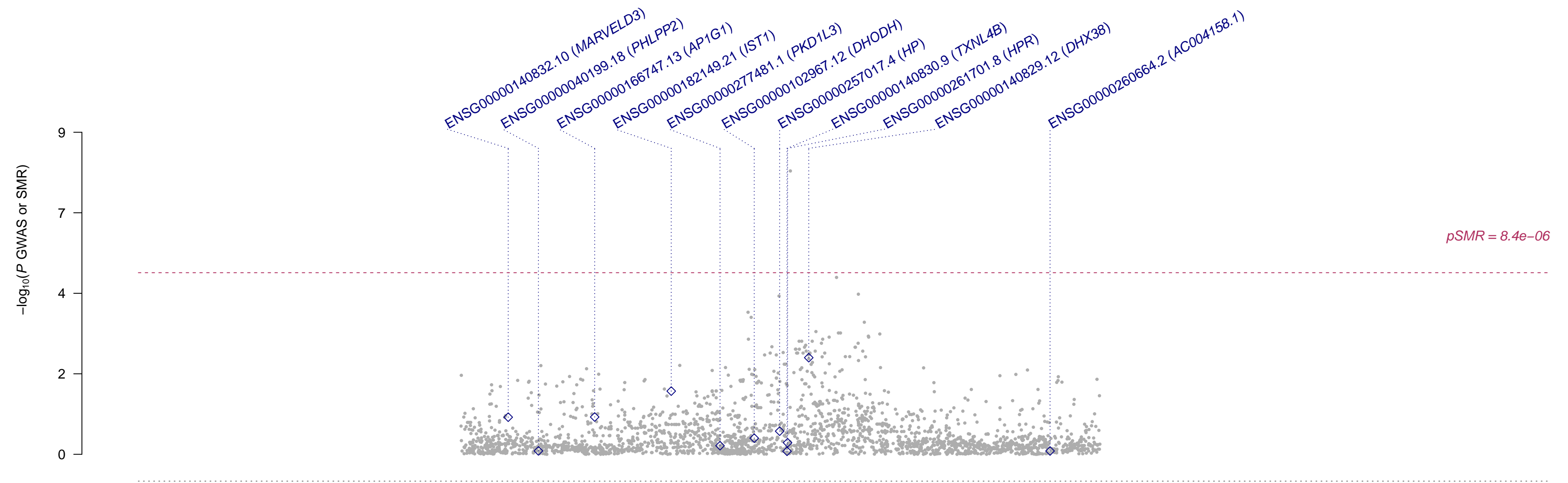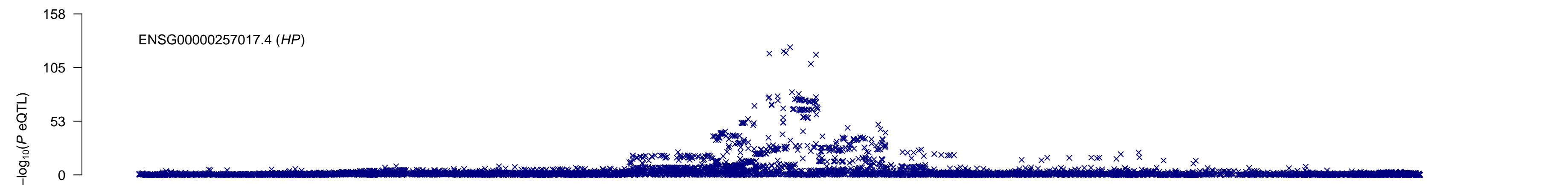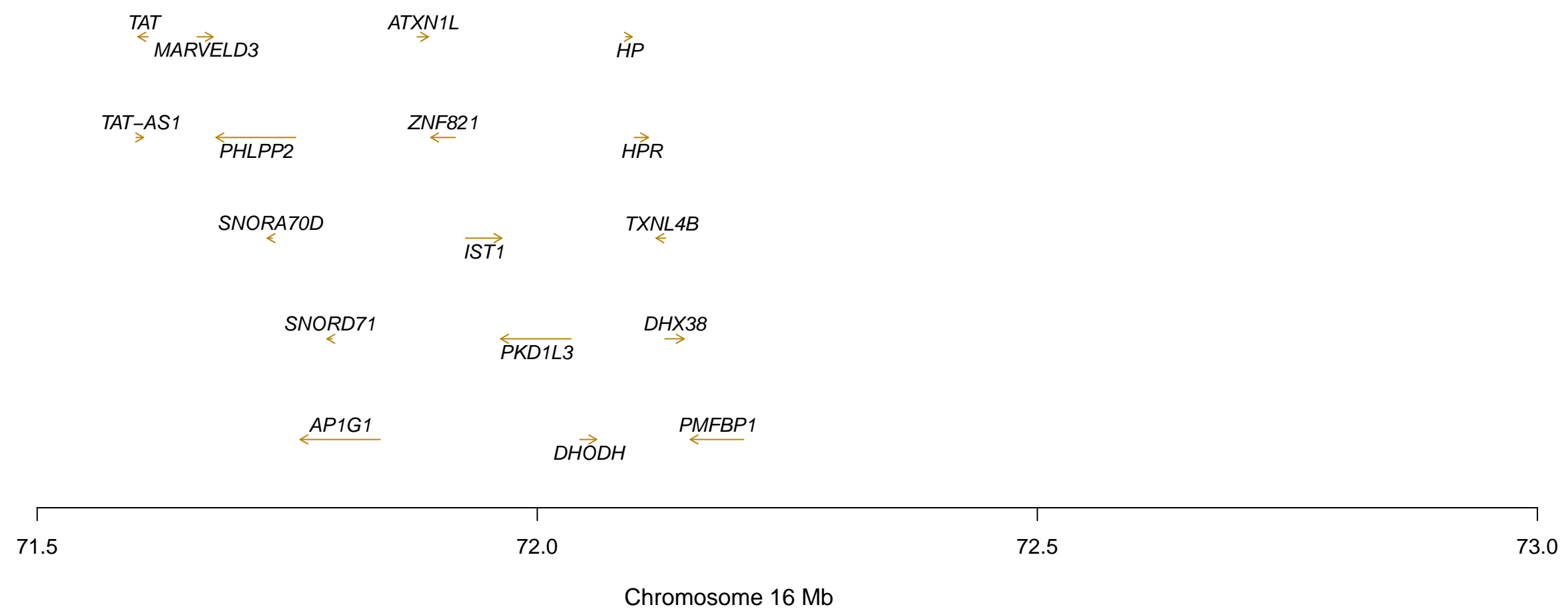

Supplement: Supplementary file 2 — Supplementary Material 2 [file 13568_2025_1969_MOESM2_ESM.zip › Revised supplementary materials/4 Novel loci SMR results/plot/ENSG00000257017.4_LocusPlot.pdf]

ASD novel loci

ENSG00000261701.8 (*HPR*)

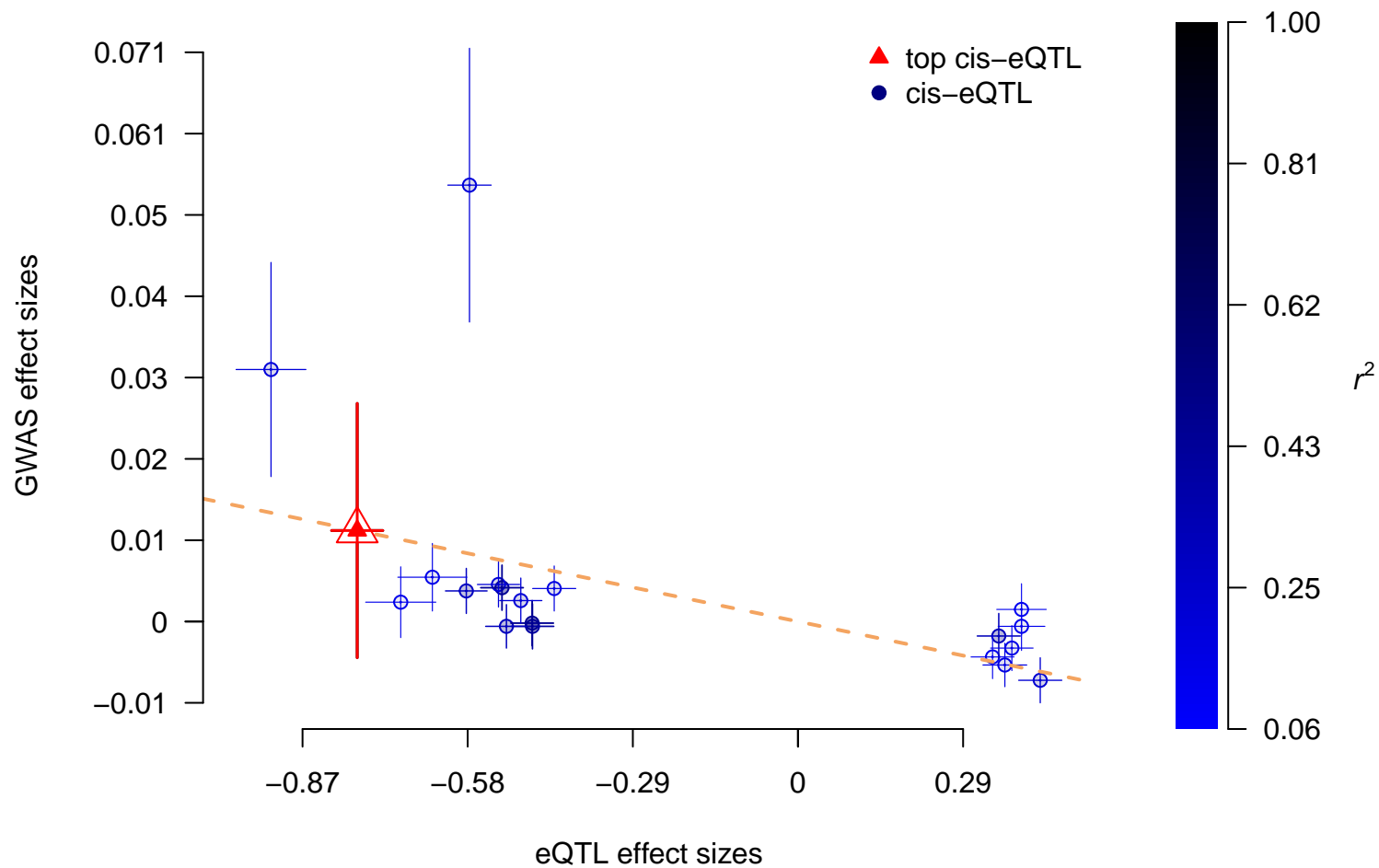

Supplement: Supplementary file 2 — Supplementary Material 2 [file 13568_2025_1969_MOESM2_ESM.zip › Revised supplementary materials/4 Novel loci SMR results/plot/ENSG00000261701.8_EffectPlot.pdf]

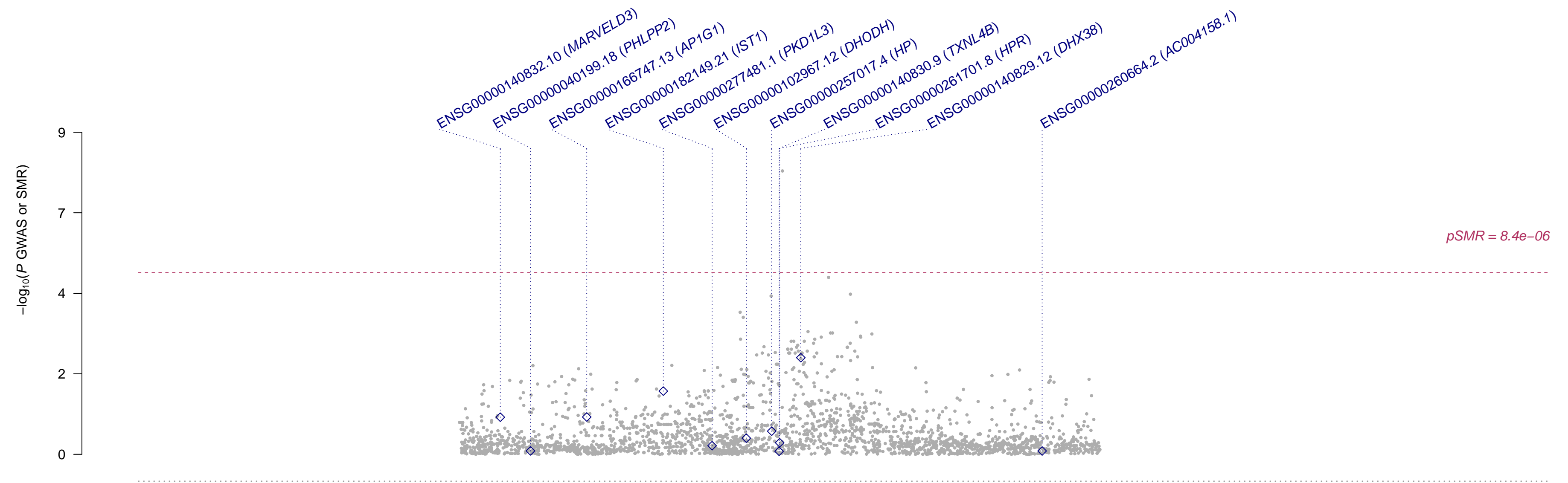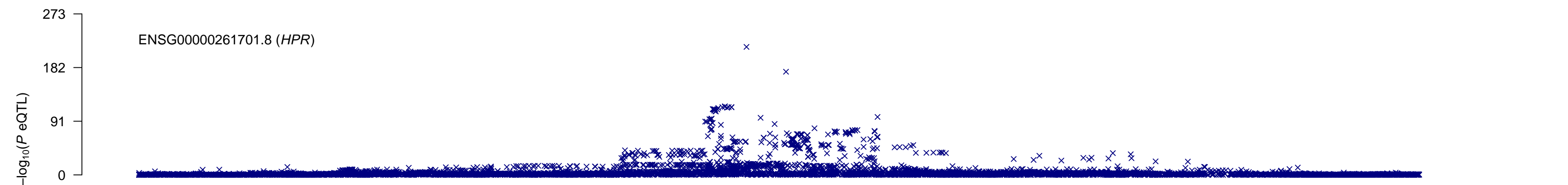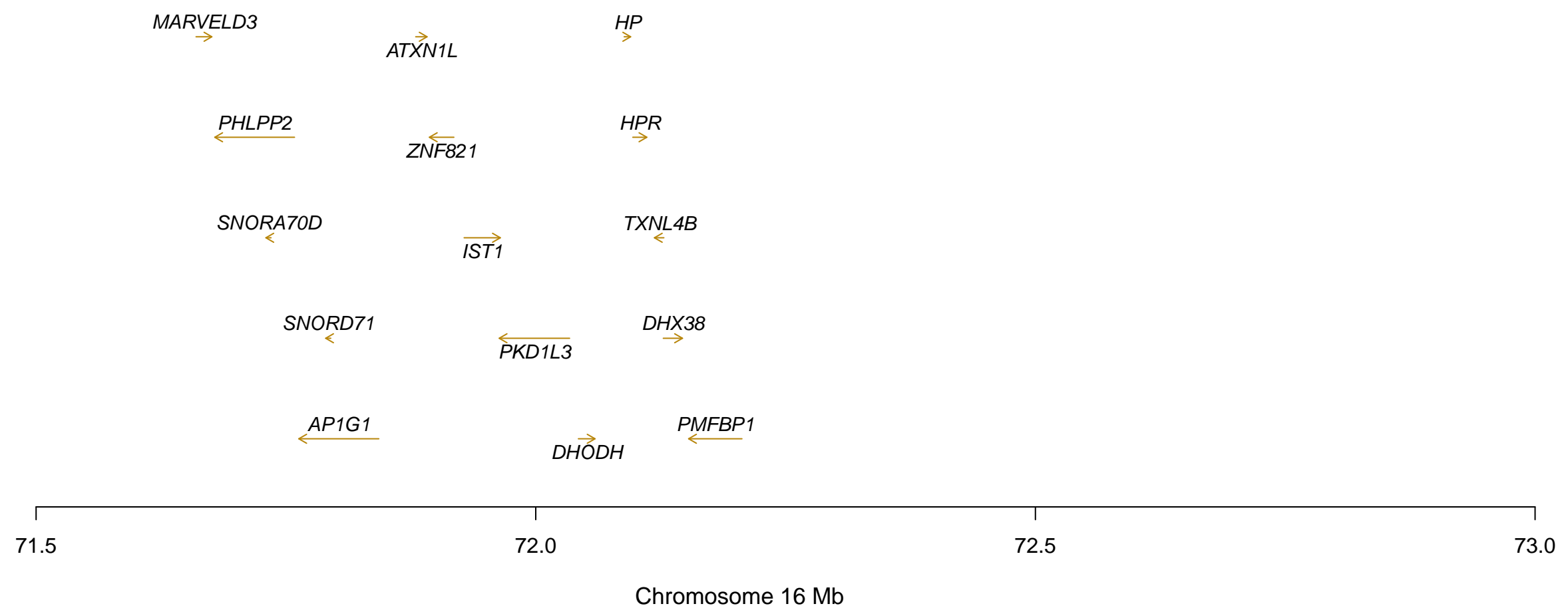

Supplement: Supplementary file 2 — Supplementary Material 2 [file 13568_2025_1969_MOESM2_ESM.zip › Revised supplementary materials/4 Novel loci SMR results/plot/ENSG00000261701.8_LocusPlot.pdf]

ASD novel loci

ENSG00000269918.1 (*AF131215.6*)

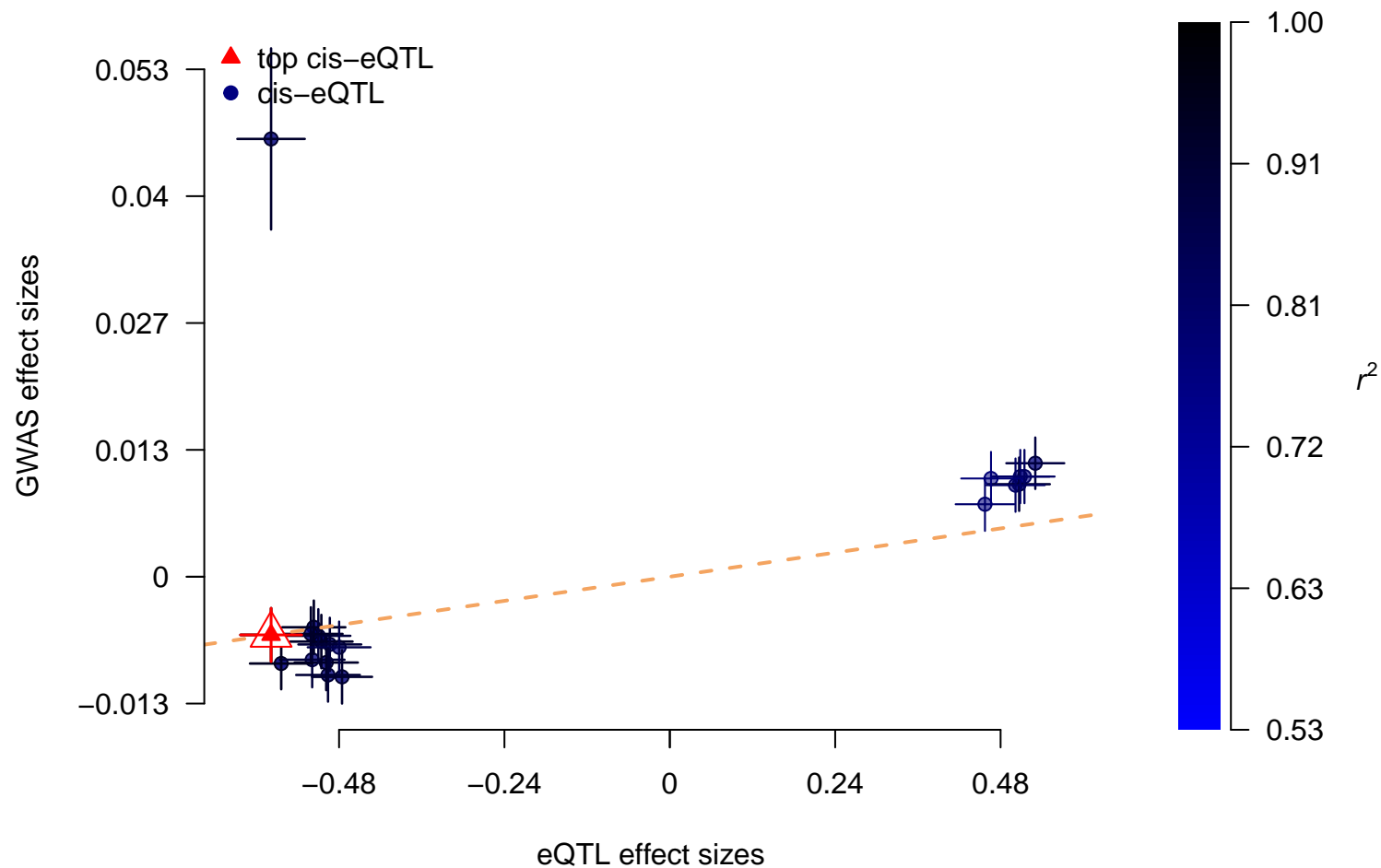

Supplement: Supplementary file 2 — Supplementary Material 2 [file 13568_2025_1969_MOESM2_ESM.zip › Revised supplementary materials/4 Novel loci SMR results/plot/ENSG00000269918.1_EffectPlot.pdf]

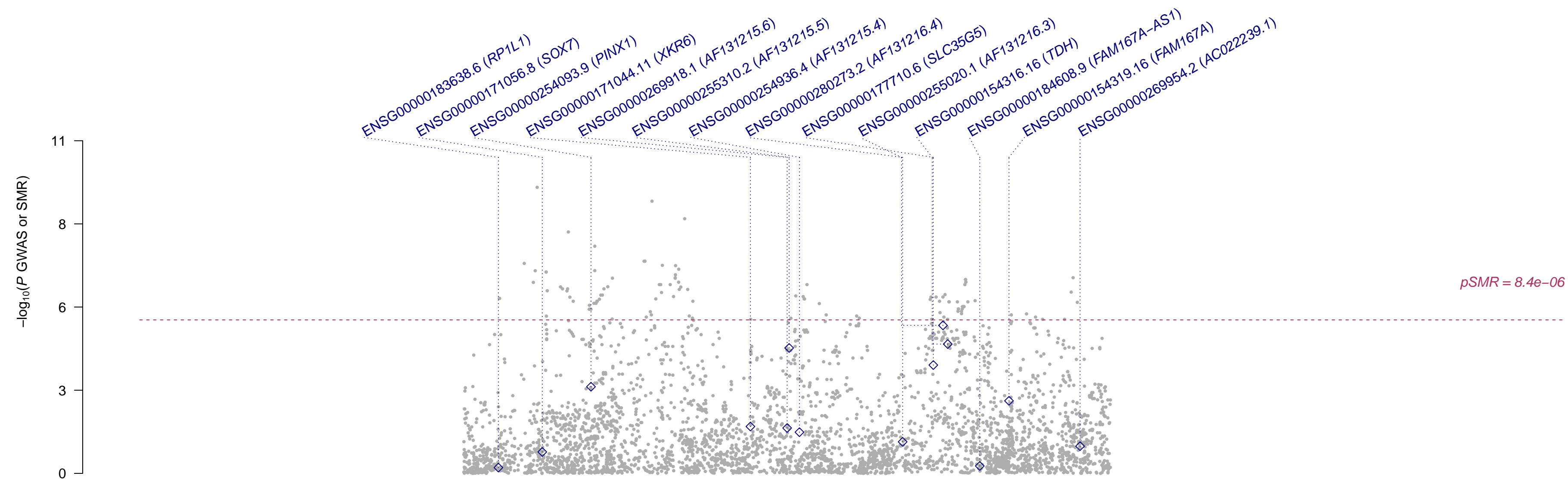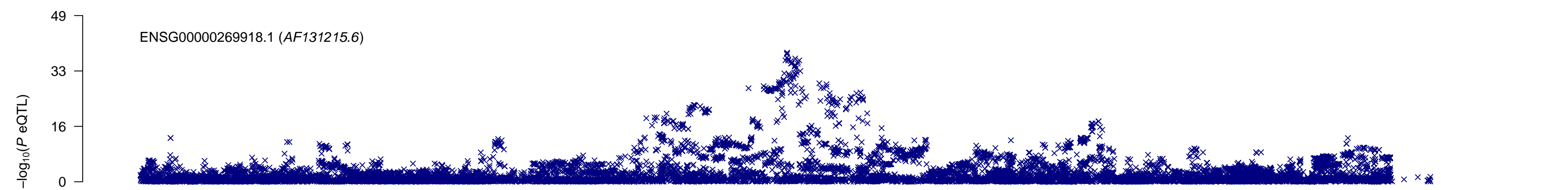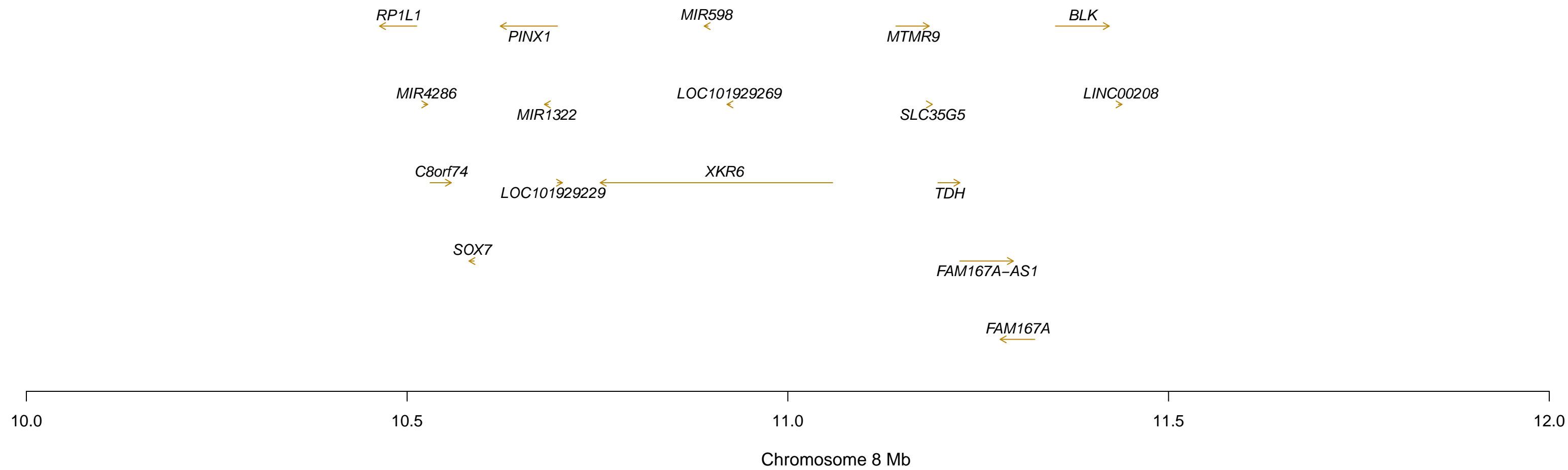

Supplement: Supplementary file 2 — Supplementary Material 2 [file 13568_2025_1969_MOESM2_ESM.zip › Revised supplementary materials/4 Novel loci SMR results/plot/ENSG00000269918.1_LocusPlot.pdf]

# Forest Plot (OR): *Acidaminococcus fermentans*

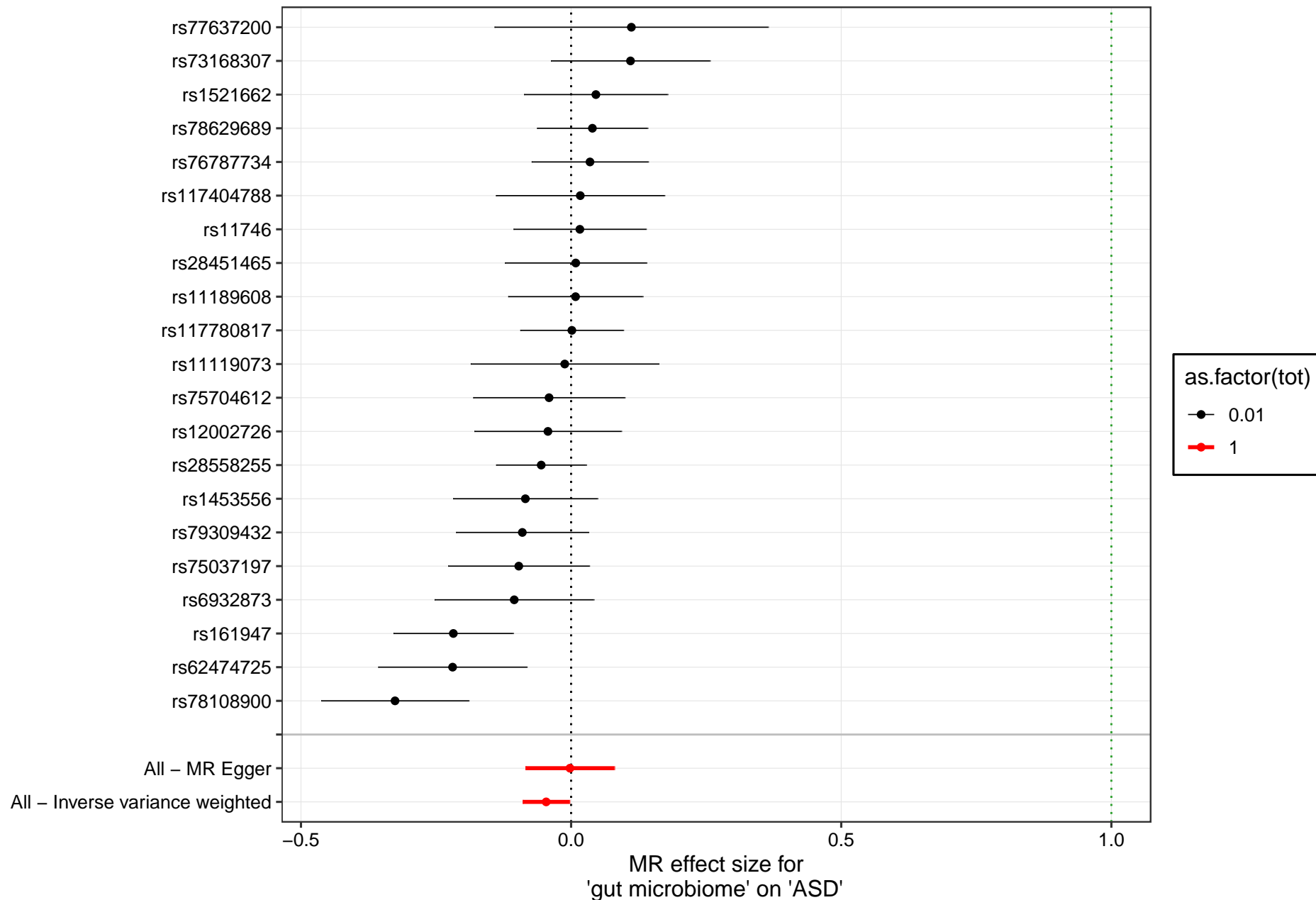

Supplement: Supplementary file 2 — Supplementary Material 2 [file 13568_2025_1969_MOESM2_ESM.zip › Revised supplementary materials/5 Forward MR analysis results/plot/forest_or_Acidaminococcus fermentans.pdf]

# Forest Plot (OR): CAG-475

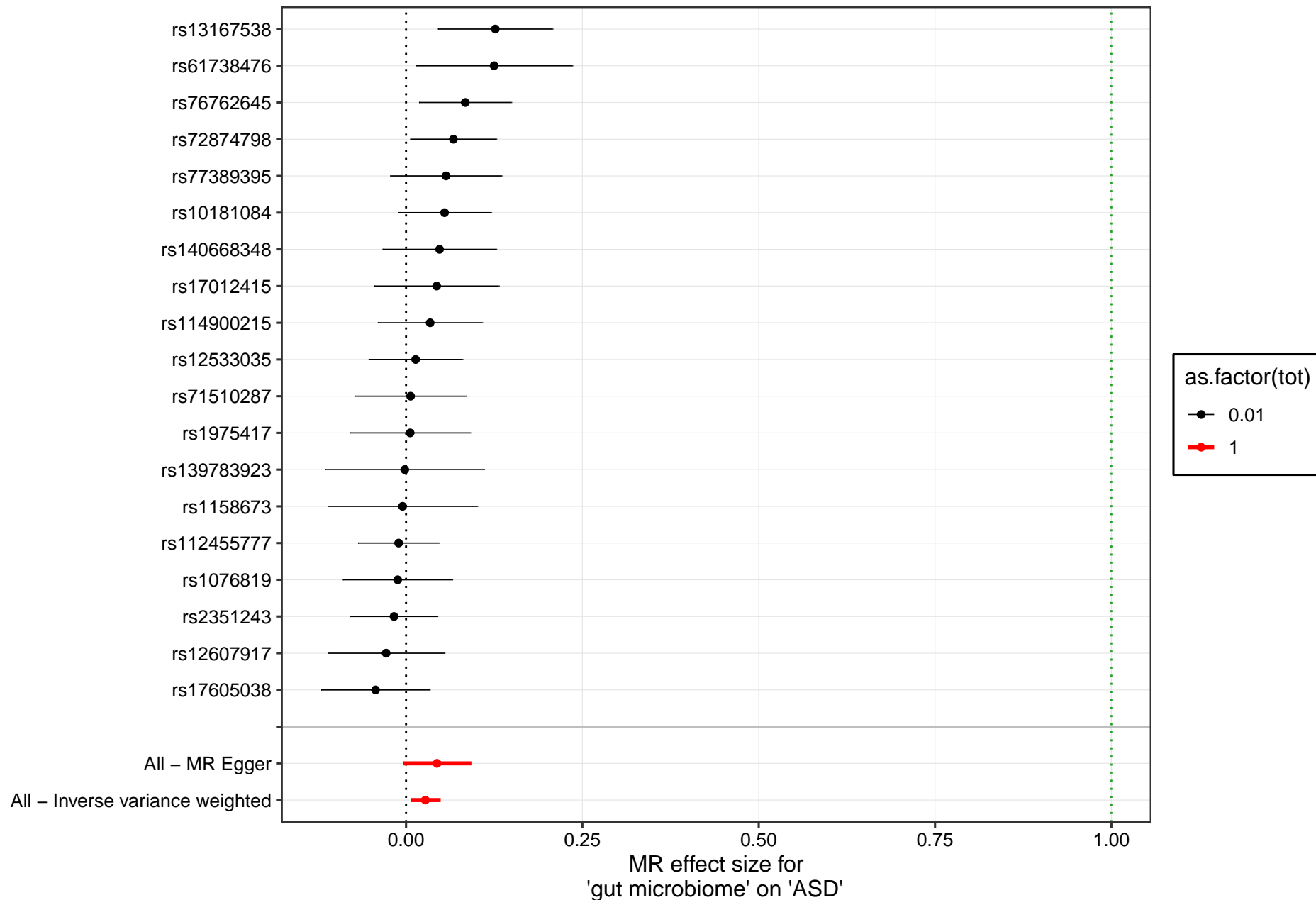

Supplement: Supplementary file 2 — Supplementary Material 2 [file 13568_2025_1969_MOESM2_ESM.zip › Revised supplementary materials/5 Forward MR analysis results/plot/forest_or_CAG-475.pdf]

# Forest Plot (OR): CAG-510 sp002432425

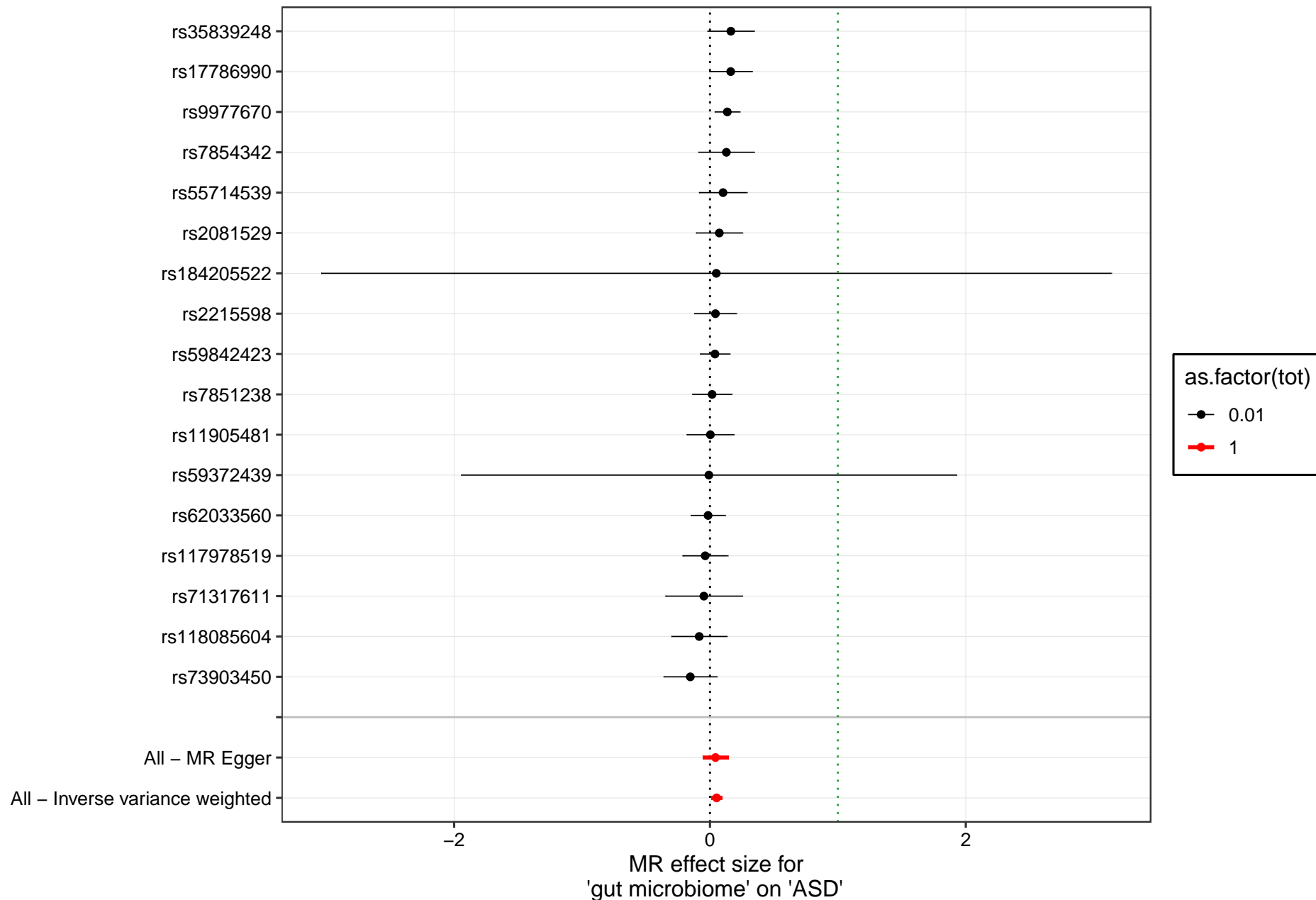

Supplement: Supplementary file 2 — Supplementary Material 2 [file 13568_2025_1969_MOESM2_ESM.zip › Revised supplementary materials/5 Forward MR analysis results/plot/forest_or_CAG-510 sp002432425.pdf]

# Forest Plot (OR): CAG-884

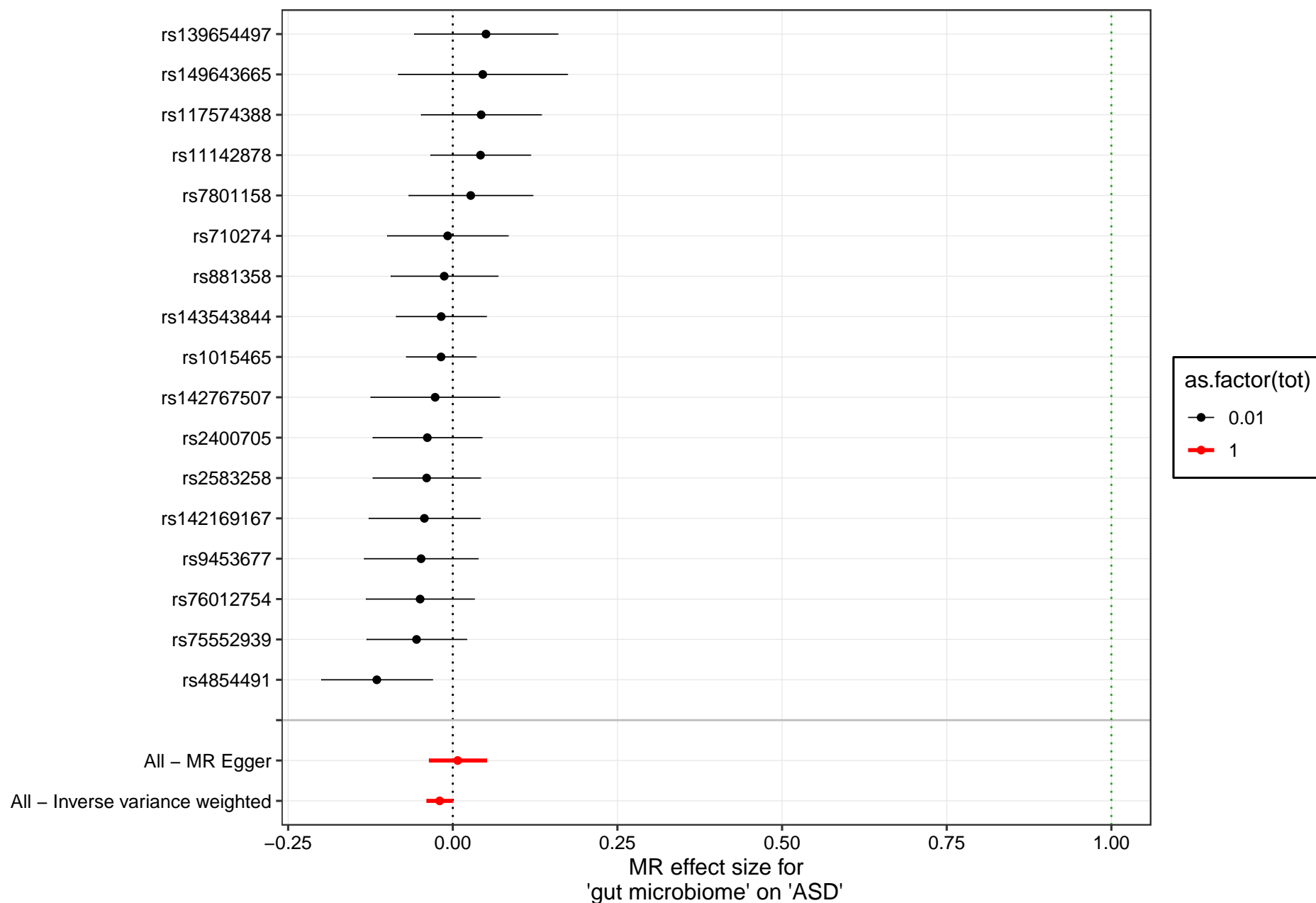

Supplement: Supplementary file 2 — Supplementary Material 2 [file 13568_2025_1969_MOESM2_ESM.zip › Revised supplementary materials/5 Forward MR analysis results/plot/forest_or_CAG-884.pdf]

# Forest Plot (OR): Coprobacillus

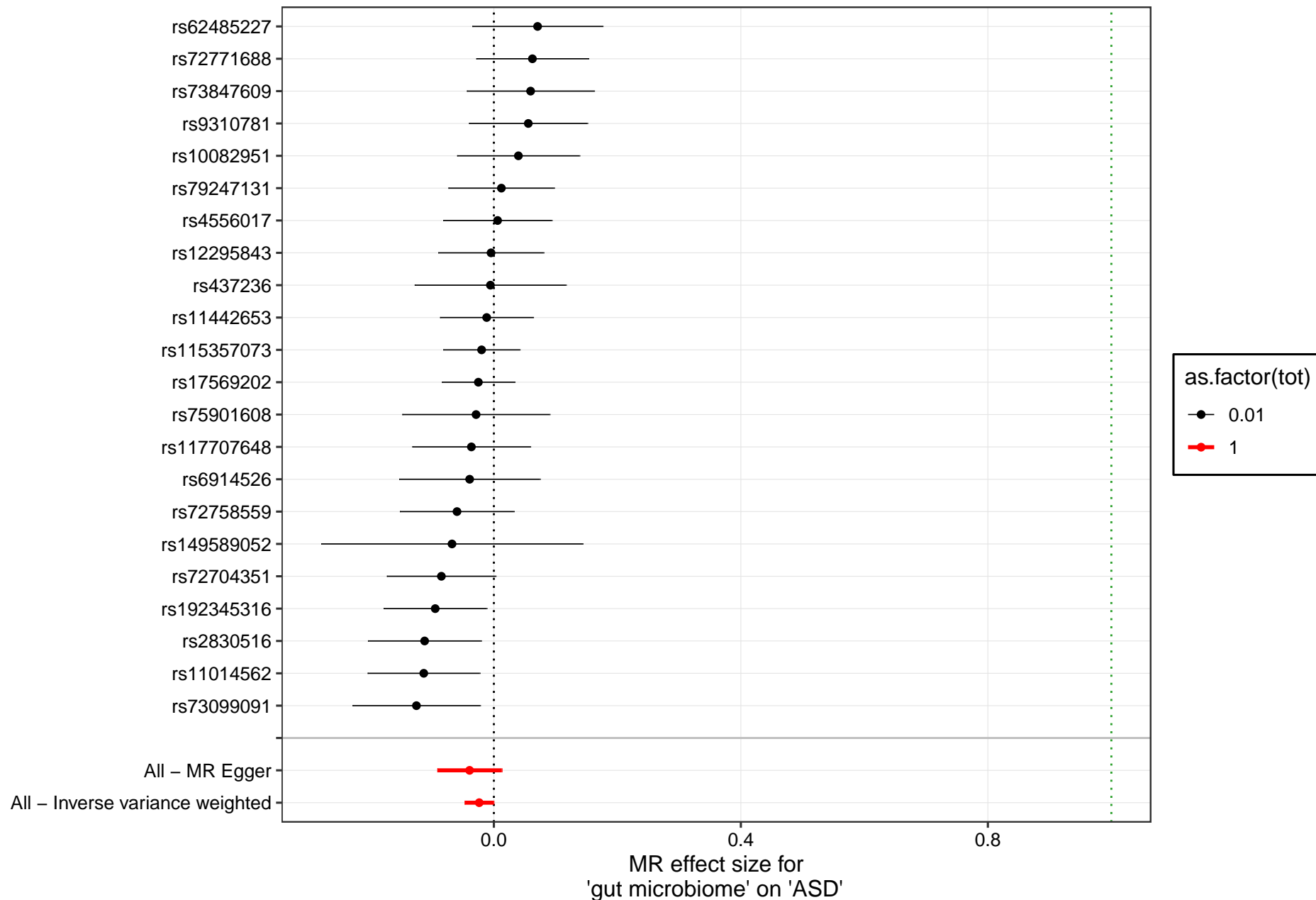

Supplement: Supplementary file 2 — Supplementary Material 2 [file 13568_2025_1969_MOESM2_ESM.zip › Revised supplementary materials/5 Forward MR analysis results/plot/forest_or_Coprobacillus.pdf]

# Forest Plot (OR): Coprobacter secundus

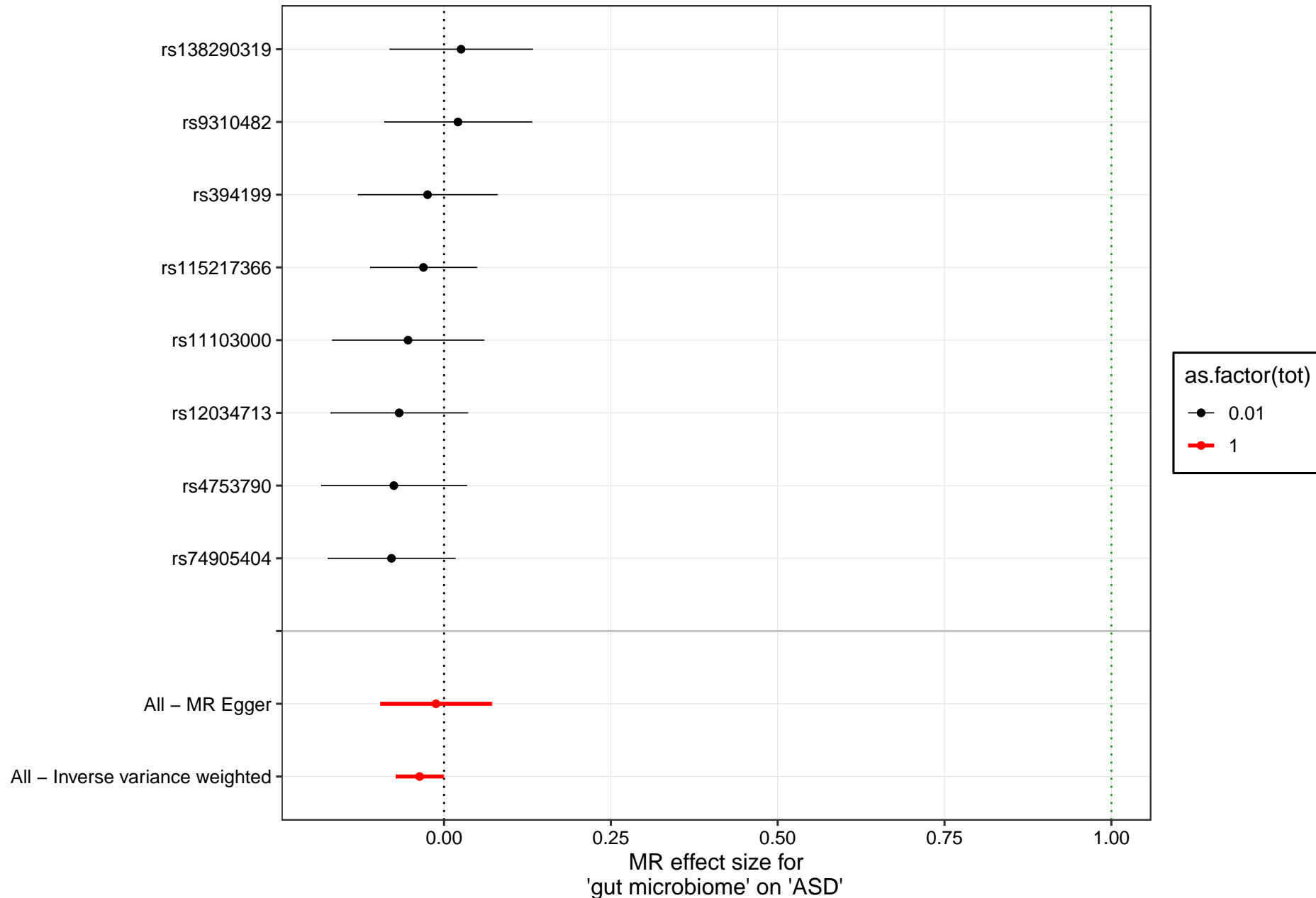

Supplement: Supplementary file 2 — Supplementary Material 2 [file 13568_2025_1969_MOESM2_ESM.zip › Revised supplementary materials/5 Forward MR analysis results/plot/forest_or_Coprobacter secundus.pdf]

# Forest Plot (OR): DTU024 sp002411105

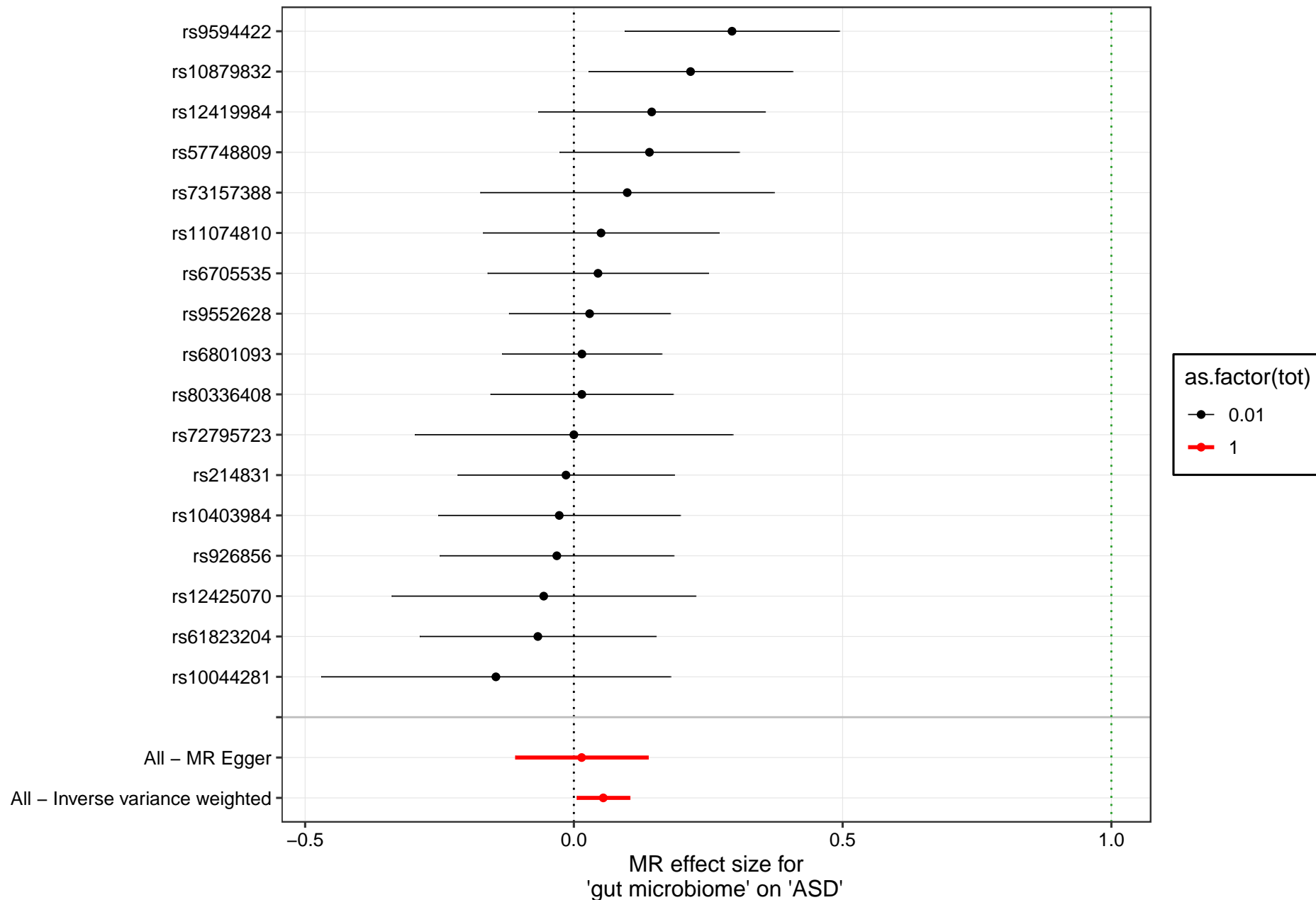

Supplement: Supplementary file 2 — Supplementary Material 2 [file 13568_2025_1969_MOESM2_ESM.zip › Revised supplementary materials/5 Forward MR analysis results/plot/forest_or_DTU024 sp002411105.pdf]

# Forest Plot (OR): Endozoicomonadaceae

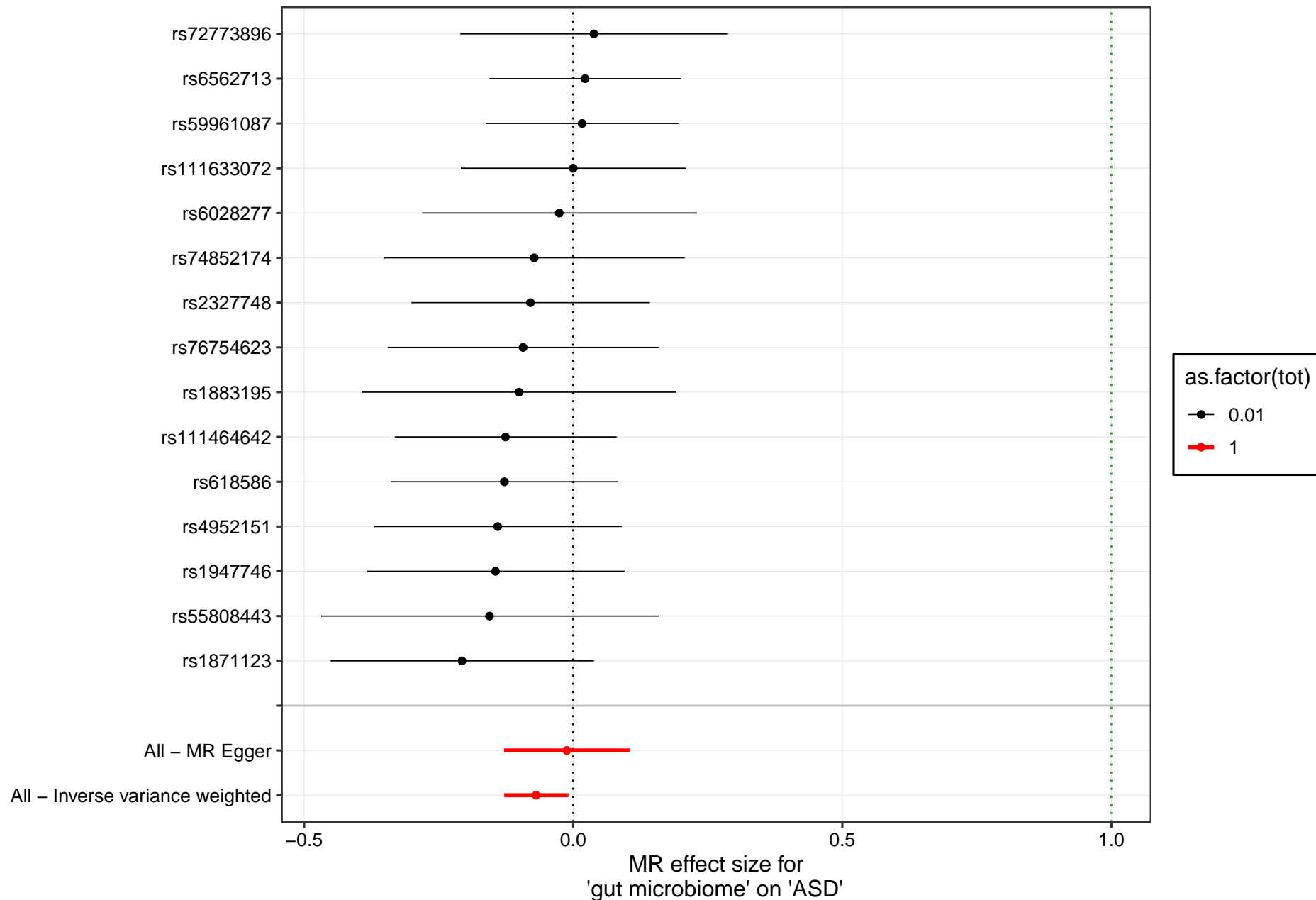

Supplement: Supplementary file 2 — Supplementary Material 2 [file 13568_2025_1969_MOESM2_ESM.zip › Revised supplementary materials/5 Forward MR analysis results/plot/forest_or_Endozoicomonadaceae.pdf]

# Forest Plot (OR): Enorma massiliensis

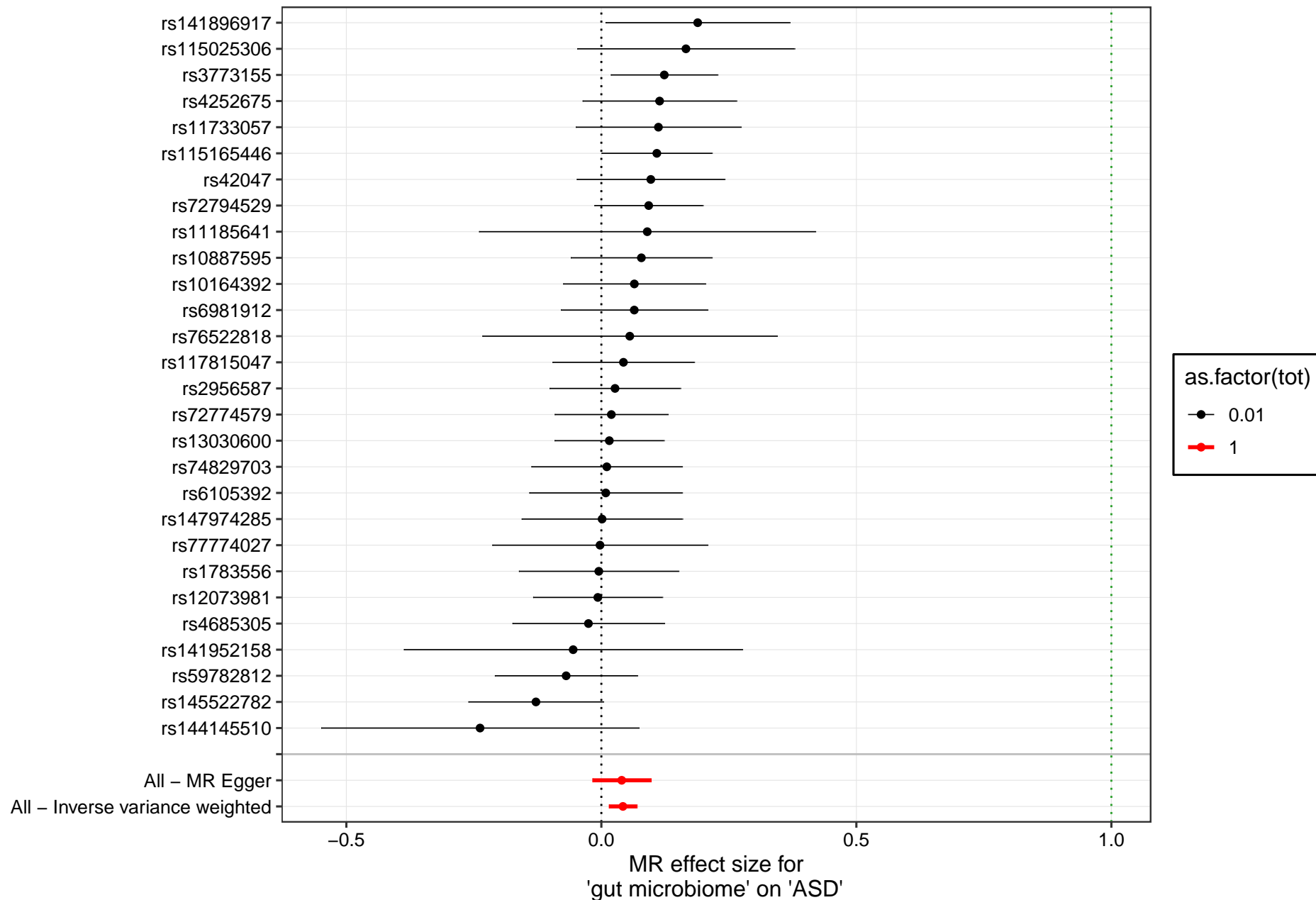

Supplement: Supplementary file 2 — Supplementary Material 2 [file 13568_2025_1969_MOESM2_ESM.zip › Revised supplementary materials/5 Forward MR analysis results/plot/forest_or_Enorma massiliensis.pdf]

# Forest Plot (OR): Enterococcus faecalis

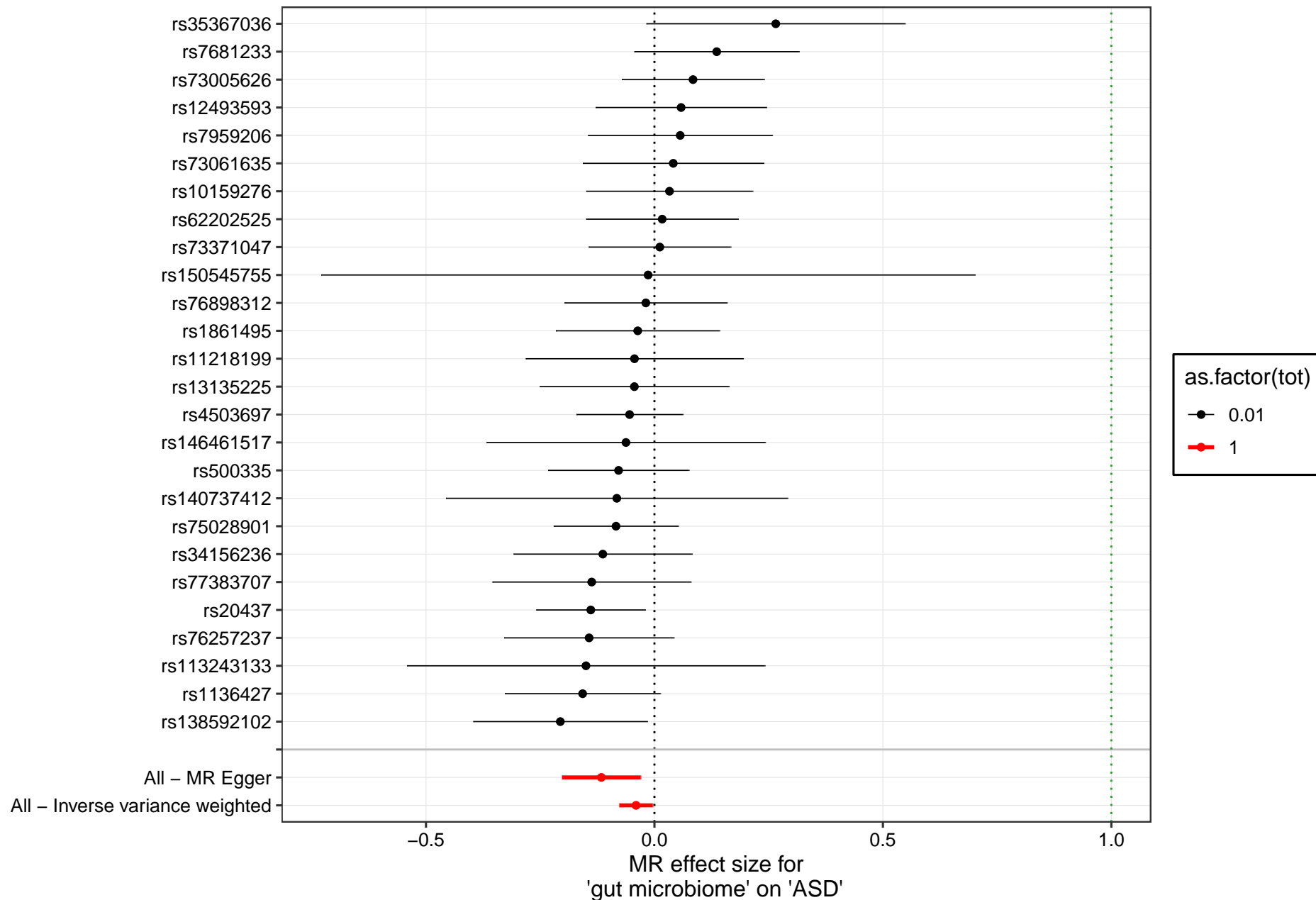

Supplement: Supplementary file 2 — Supplementary Material 2 [file 13568_2025_1969_MOESM2_ESM.zip › Revised supplementary materials/5 Forward MR analysis results/plot/forest_or_Enterococcus faecalis.pdf]

# Forest Plot (OR): Eubacterium callanderi

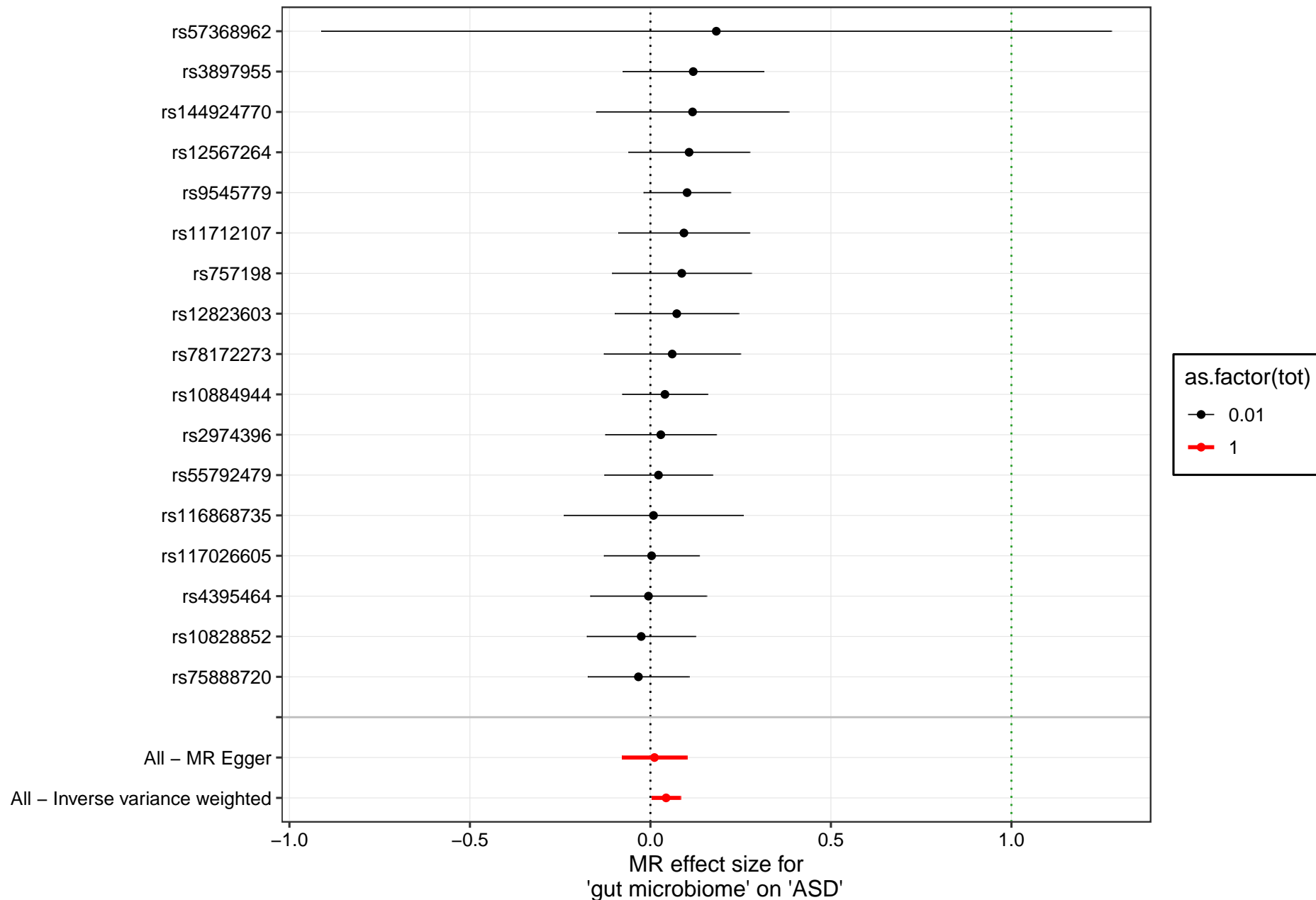

Supplement: Supplementary file 2 — Supplementary Material 2 [file 13568_2025_1969_MOESM2_ESM.zip › Revised supplementary materials/5 Forward MR analysis results/plot/forest_or_Eubacterium callanderi.pdf]

# Forest Plot (OR): *Faecalicatena torques*

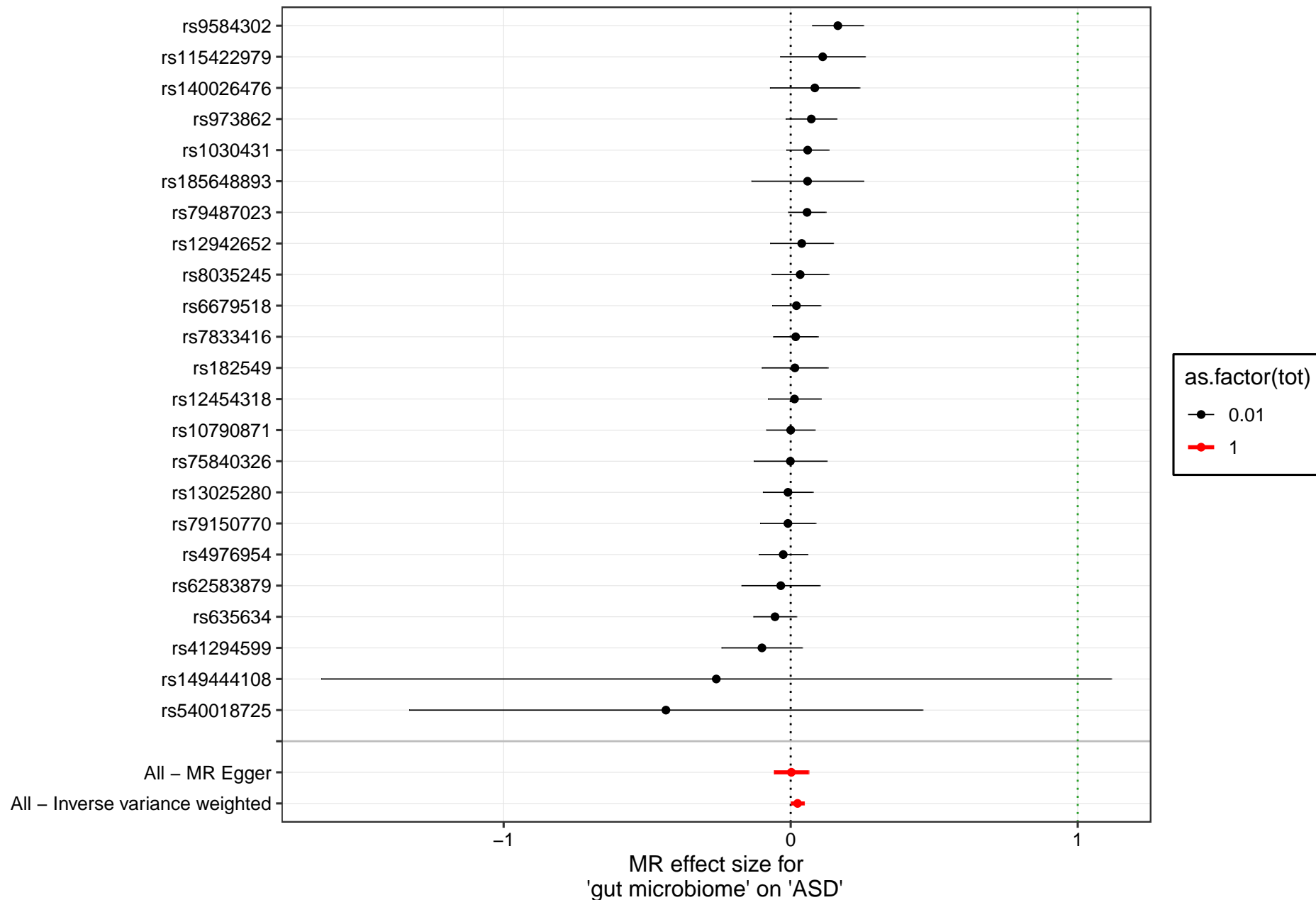

Supplement: Supplementary file 2 — Supplementary Material 2 [file 13568_2025_1969_MOESM2_ESM.zip › Revised supplementary materials/5 Forward MR analysis results/plot/forest_or_Faecalicatena torques.pdf]

# Forest Plot (OR): Fibrobacteria

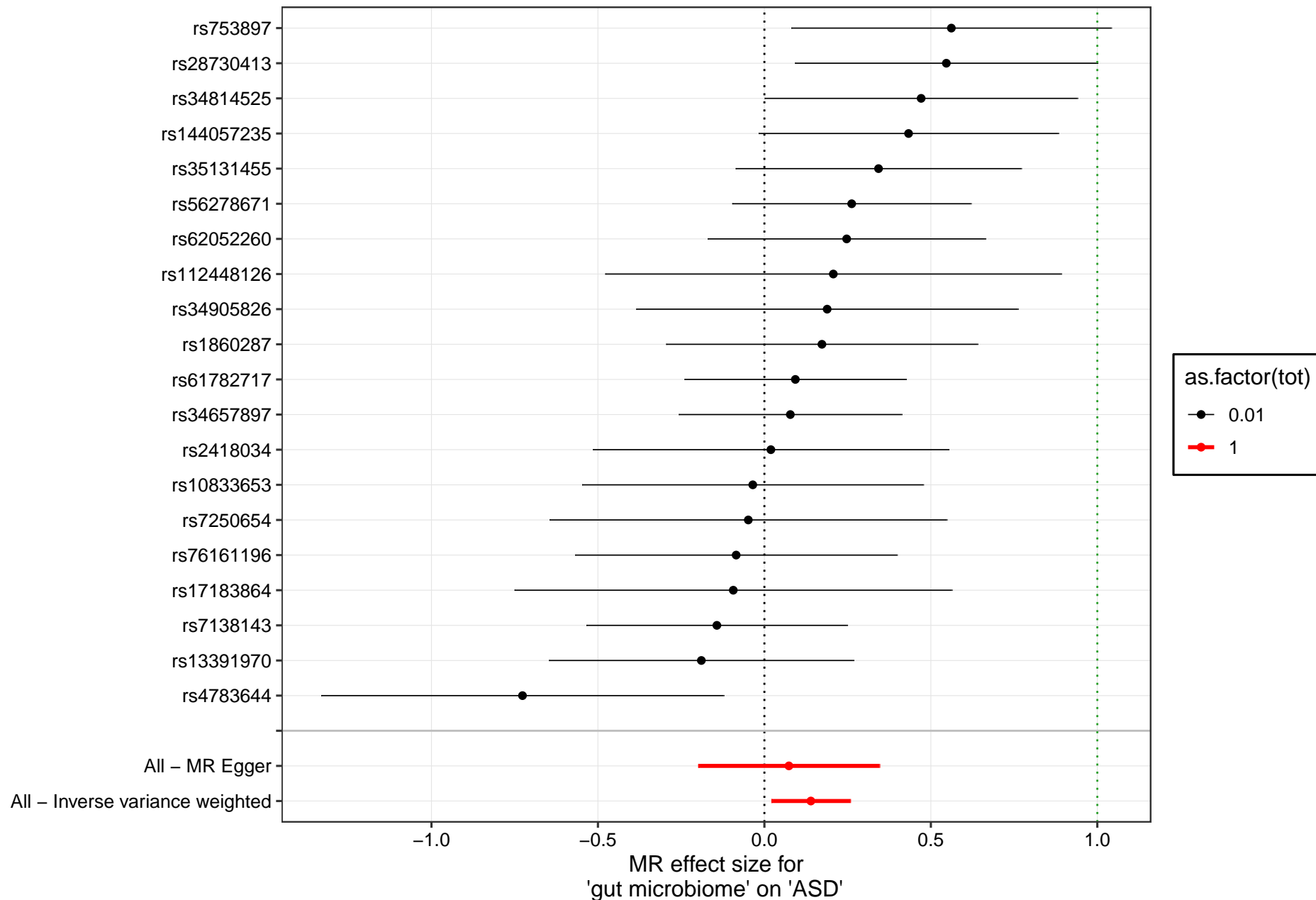

Supplement: Supplementary file 2 — Supplementary Material 2 [file 13568_2025_1969_MOESM2_ESM.zip › Revised supplementary materials/5 Forward MR analysis results/plot/forest_or_Fibrobacteria.pdf]

# Forest Plot (OR): Francisellaceae

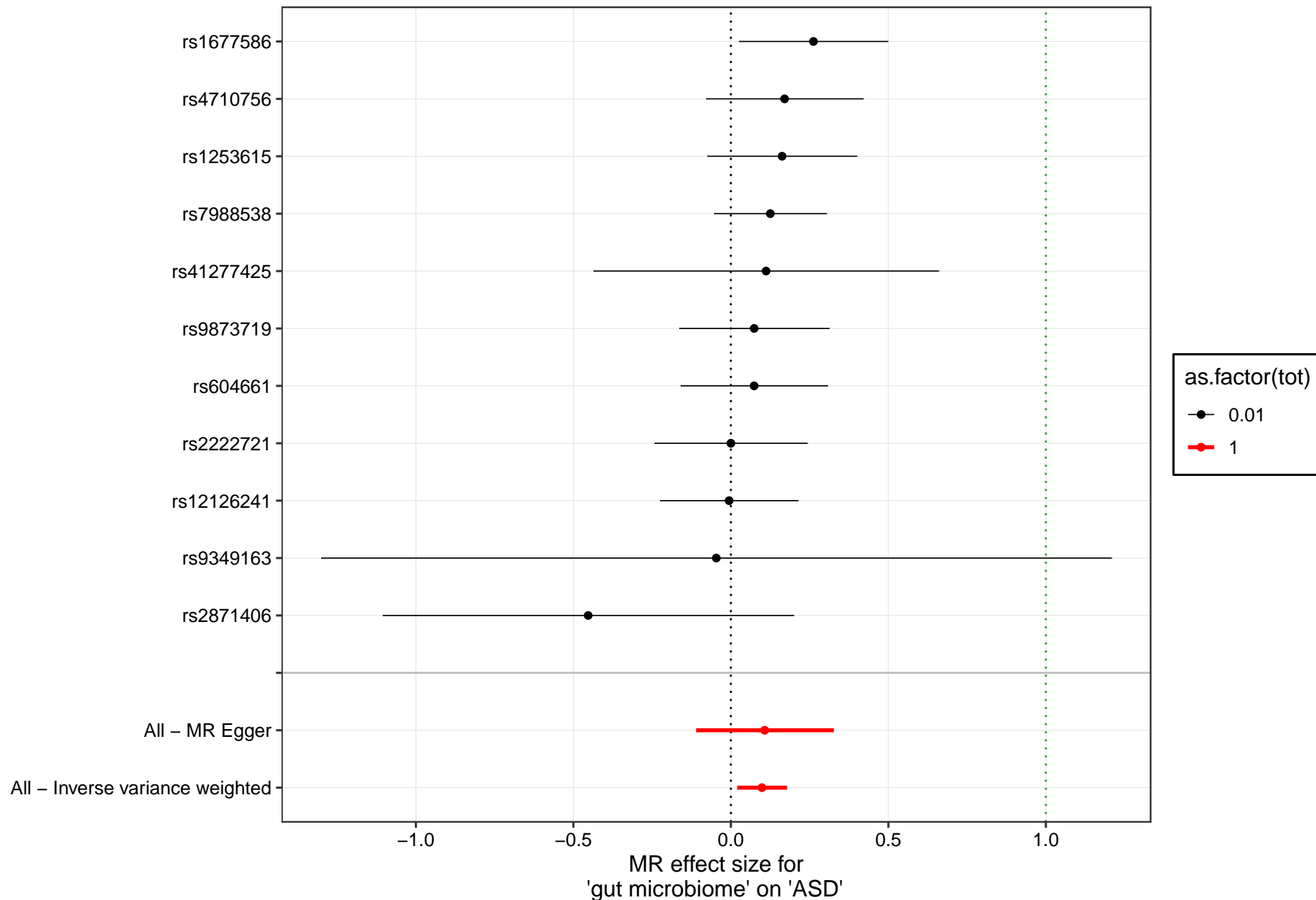

Supplement: Supplementary file 2 — Supplementary Material 2 [file 13568_2025_1969_MOESM2_ESM.zip › Revised supplementary materials/5 Forward MR analysis results/plot/forest_or_Francisellaceae.pdf]

# Forest Plot (OR): Geminocystis

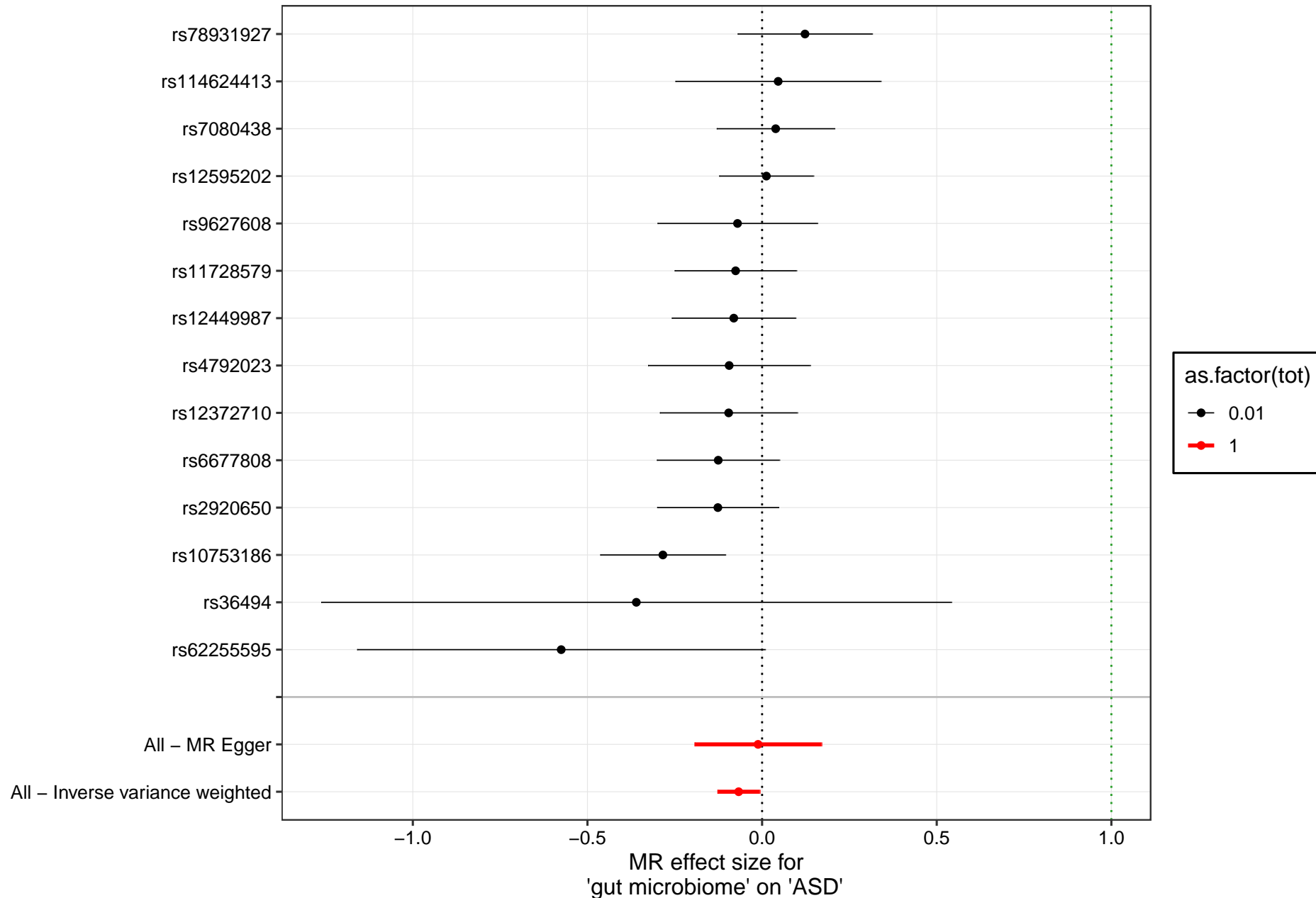

Supplement: Supplementary file 2 — Supplementary Material 2 [file 13568_2025_1969_MOESM2_ESM.zip › Revised supplementary materials/5 Forward MR analysis results/plot/forest_or_Geminocystis.pdf]

# Forest Plot (OR): koll11

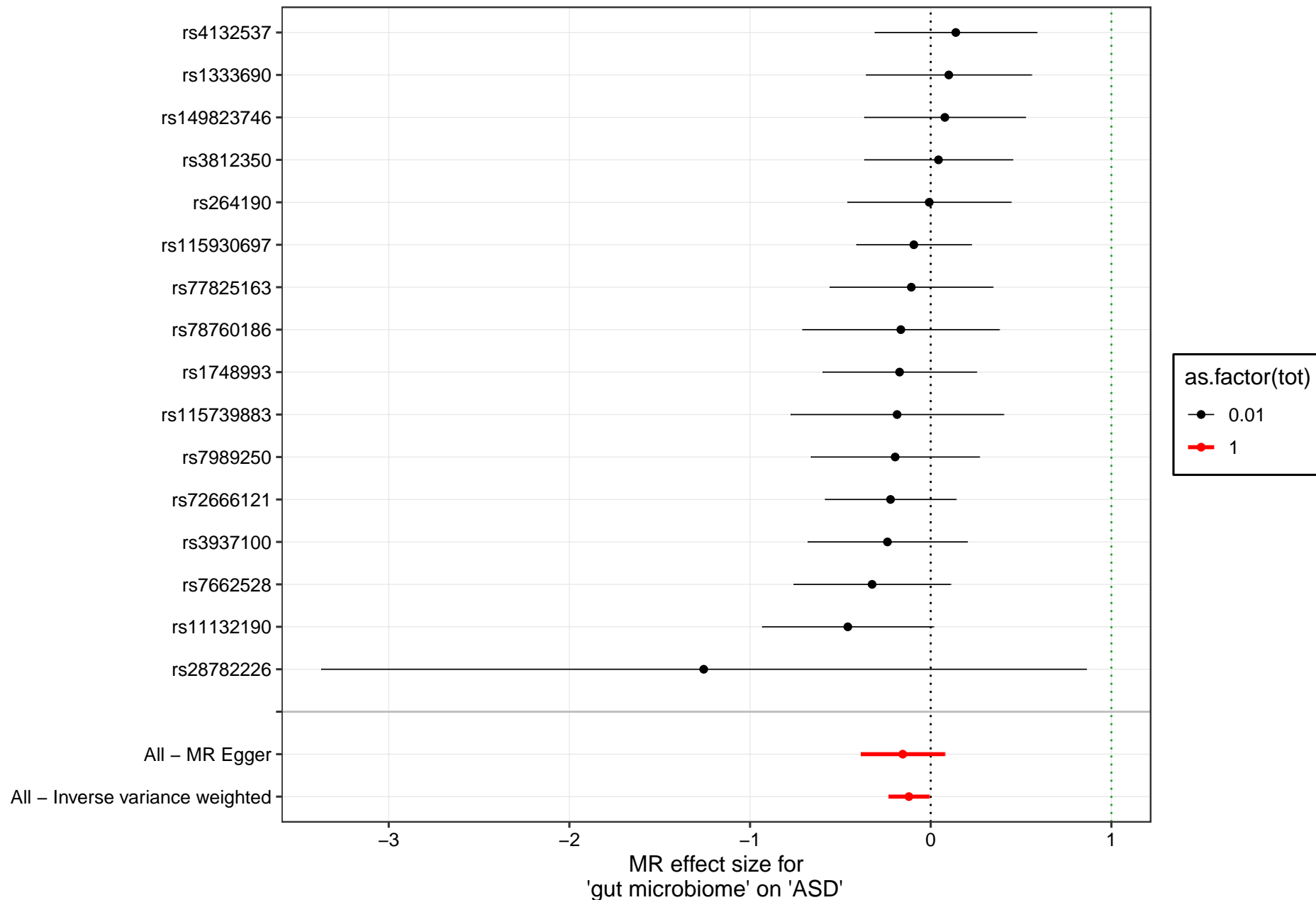

Supplement: Supplementary file 2 — Supplementary Material 2 [file 13568_2025_1969_MOESM2_ESM.zip › Revised supplementary materials/5 Forward MR analysis results/plot/forest_or_koll11.pdf]

# Forest Plot (OR): *Lachnospira rogosae*

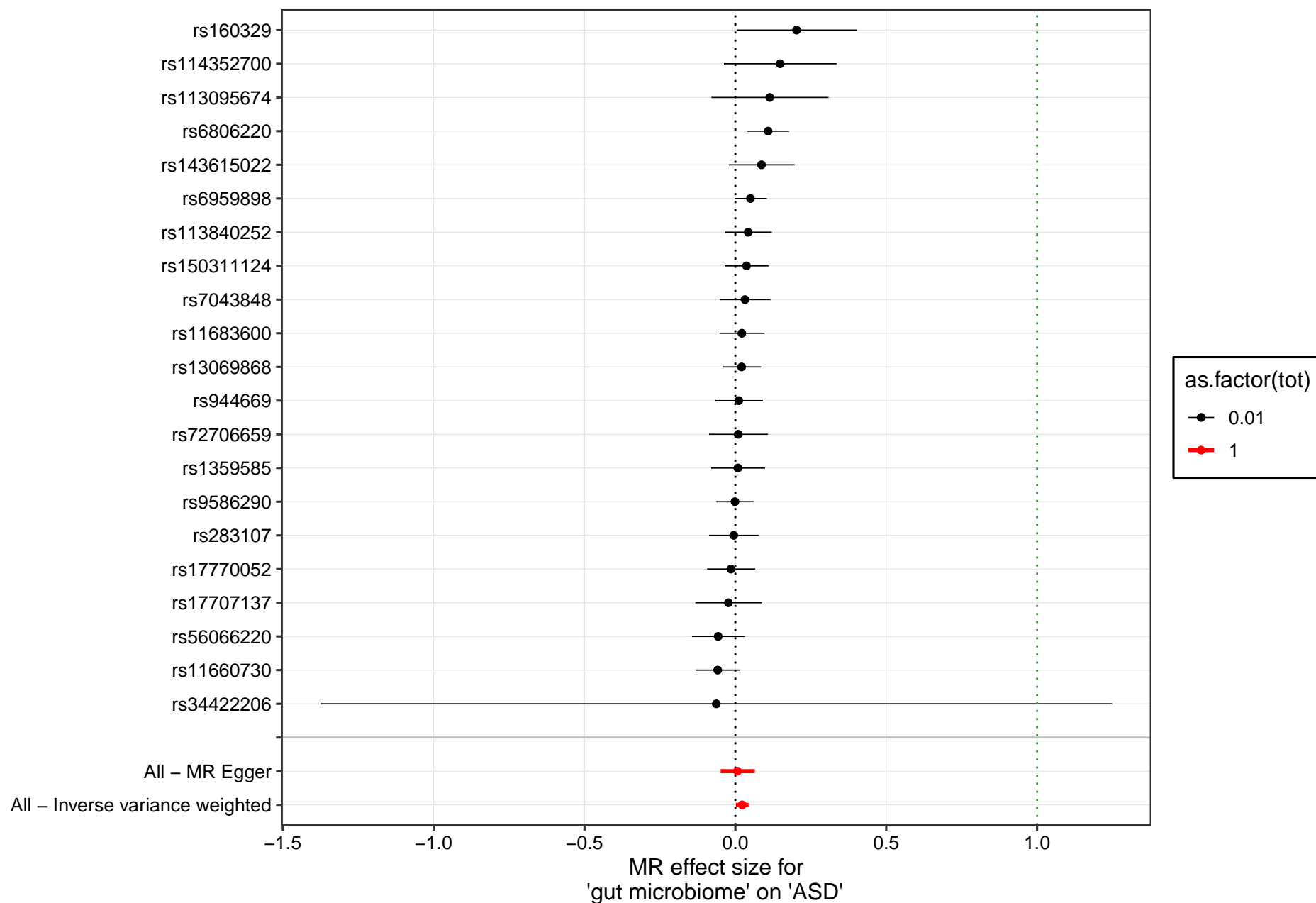

Supplement: Supplementary file 2 — Supplementary Material 2 [file 13568_2025_1969_MOESM2_ESM.zip › Revised supplementary materials/5 Forward MR analysis results/plot/forest_or_Lachnospira rogosae.pdf]

# Forest Plot (OR): Olsenella C

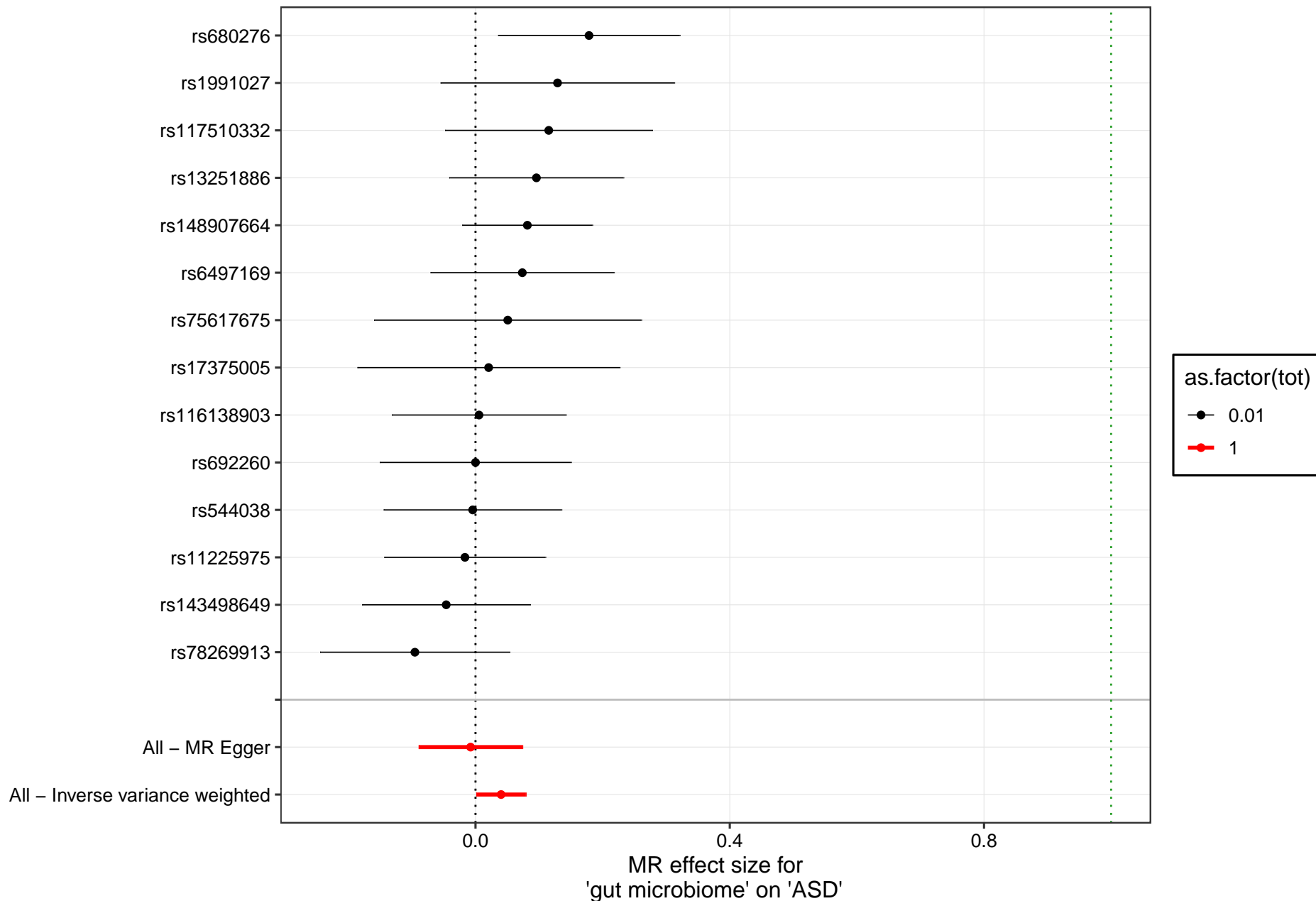

Supplement: Supplementary file 2 — Supplementary Material 2 [file 13568_2025_1969_MOESM2_ESM.zip › Revised supplementary materials/5 Forward MR analysis results/plot/forest_or_Olsenella C.pdf]

# Forest Plot (OR): Parabacteroides

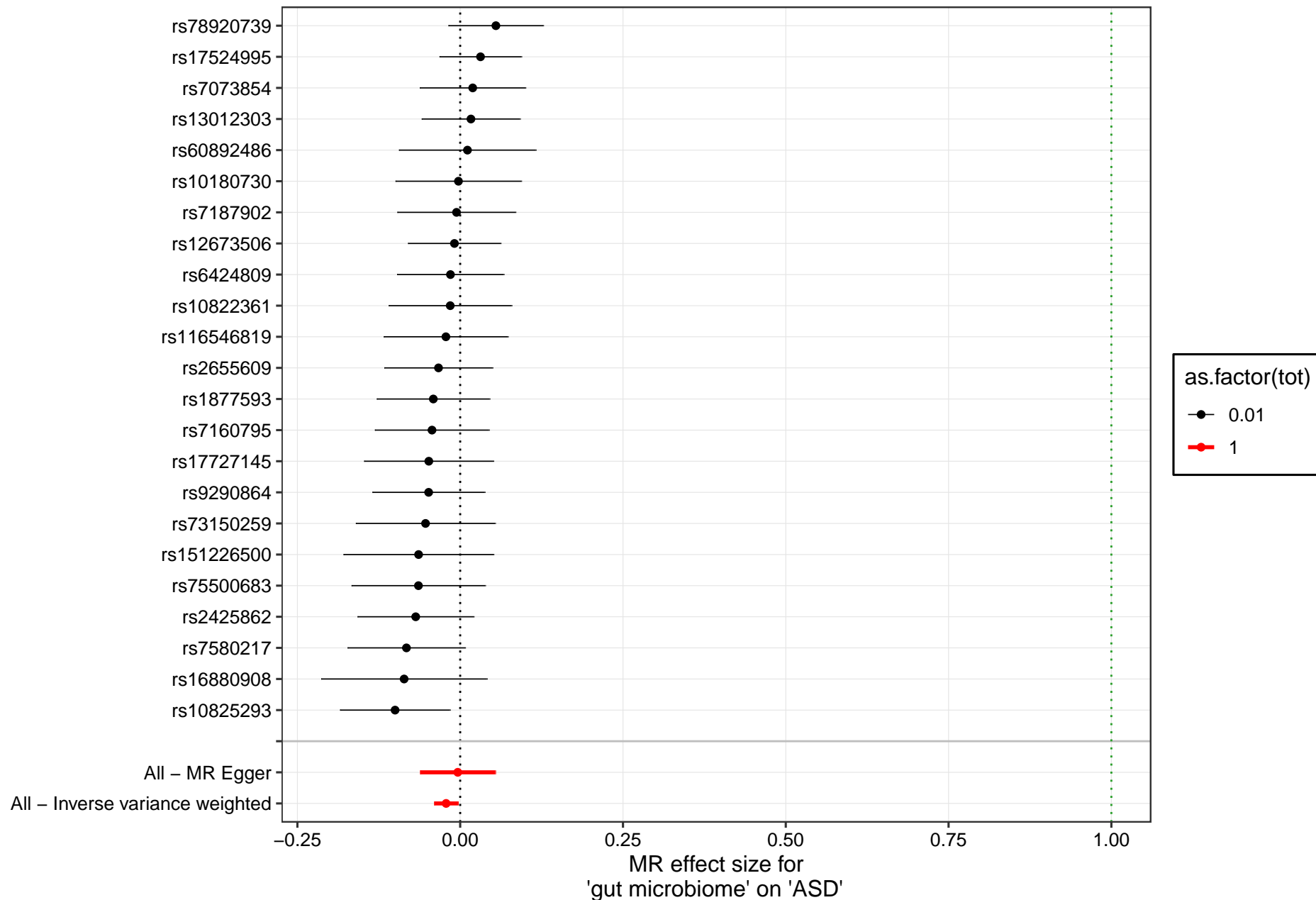

Supplement: Supplementary file 2 — Supplementary Material 2 [file 13568_2025_1969_MOESM2_ESM.zip › Revised supplementary materials/5 Forward MR analysis results/plot/forest_or_Parabacteroides.pdf]

# Forest Plot (OR): Prevotella sp002933775

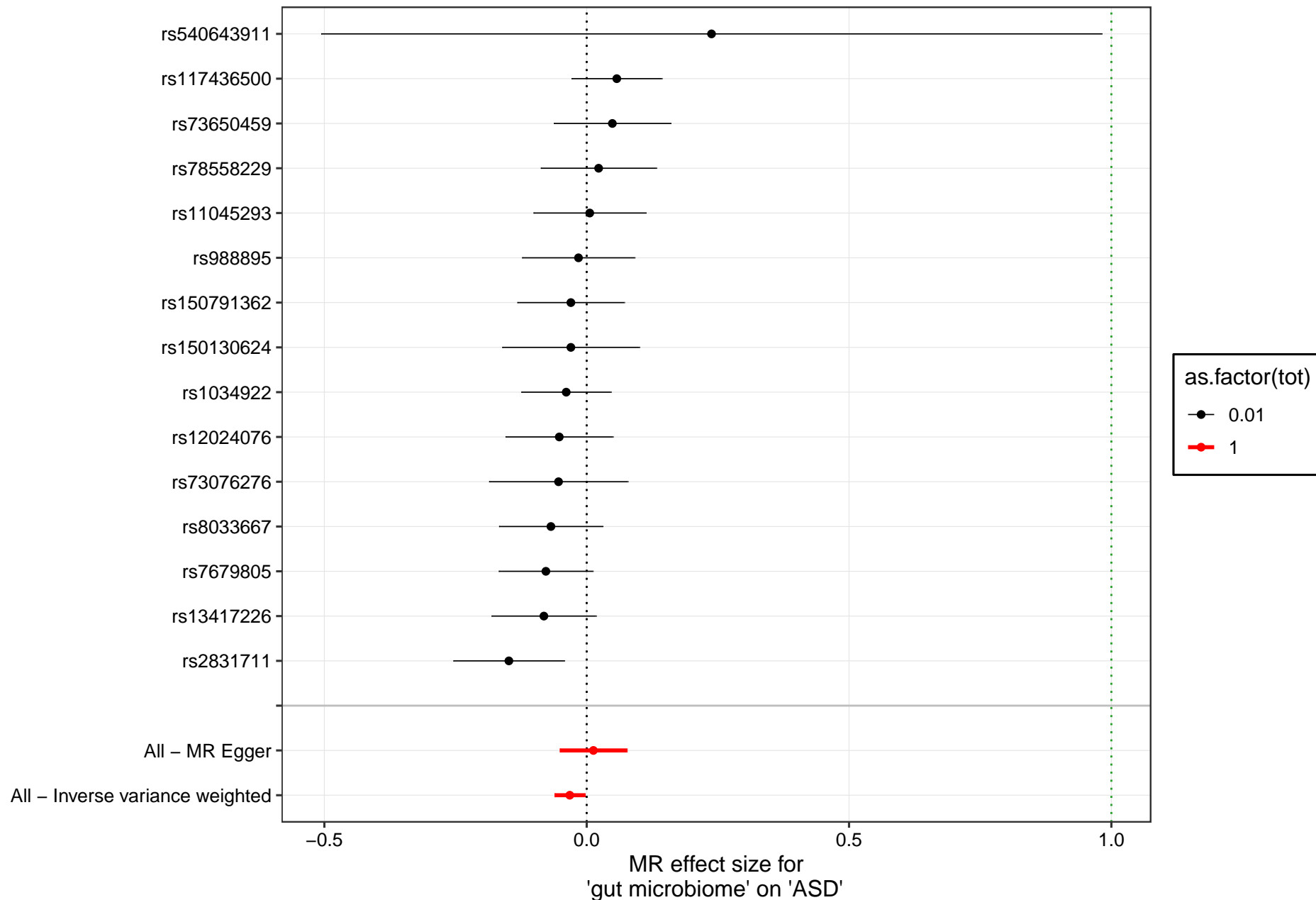

Supplement: Supplementary file 2 — Supplementary Material 2 [file 13568_2025_1969_MOESM2_ESM.zip › Revised supplementary materials/5 Forward MR analysis results/plot/forest_or_Prevotella sp002933775.pdf]

# Forest Plot (OR): UBA1066

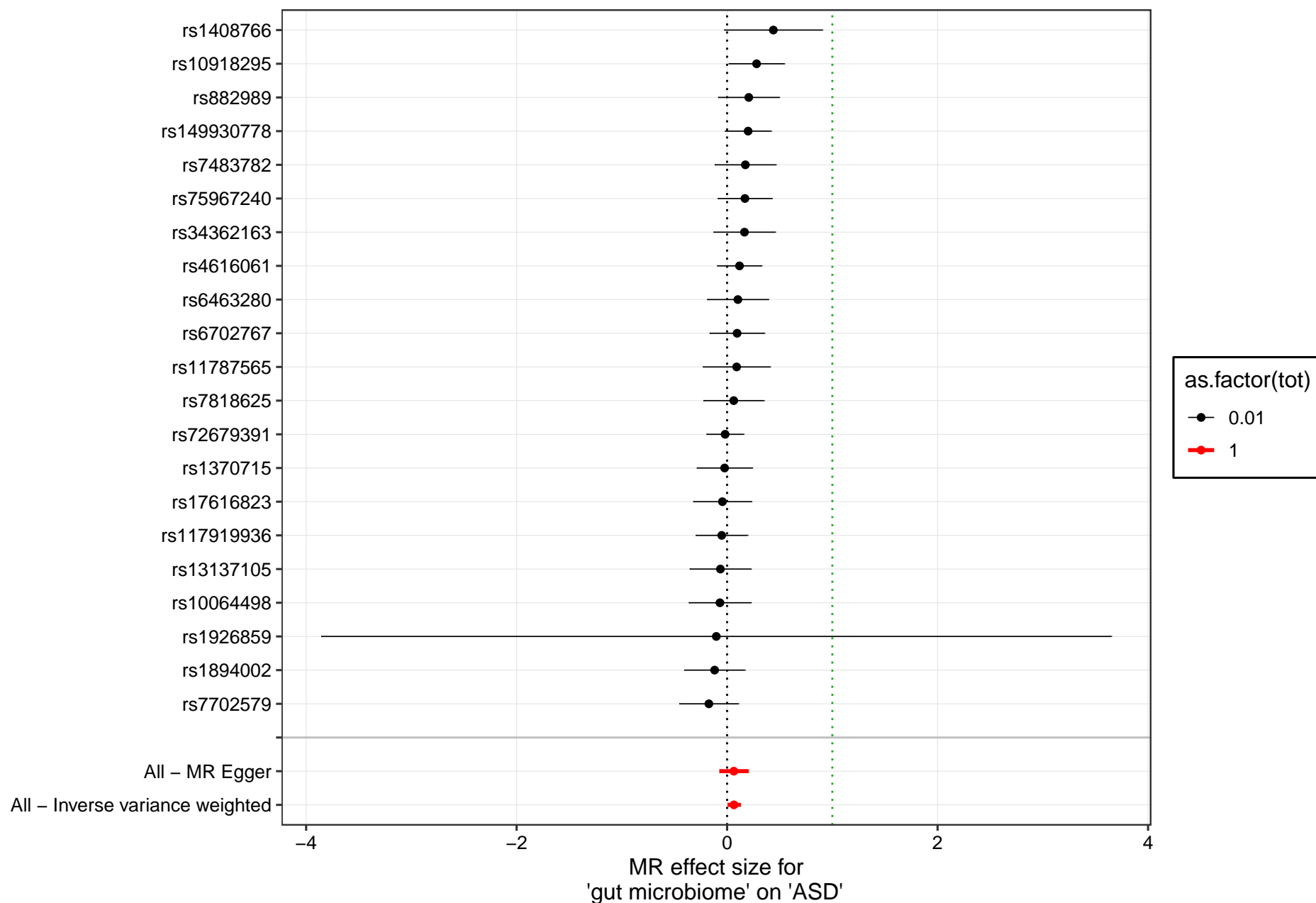

Supplement: Supplementary file 2 — Supplementary Material 2 [file 13568_2025_1969_MOESM2_ESM.zip › Revised supplementary materials/5 Forward MR analysis results/plot/forest_or_UBA1066.pdf]

# Forest Plot (OR): UBA7703

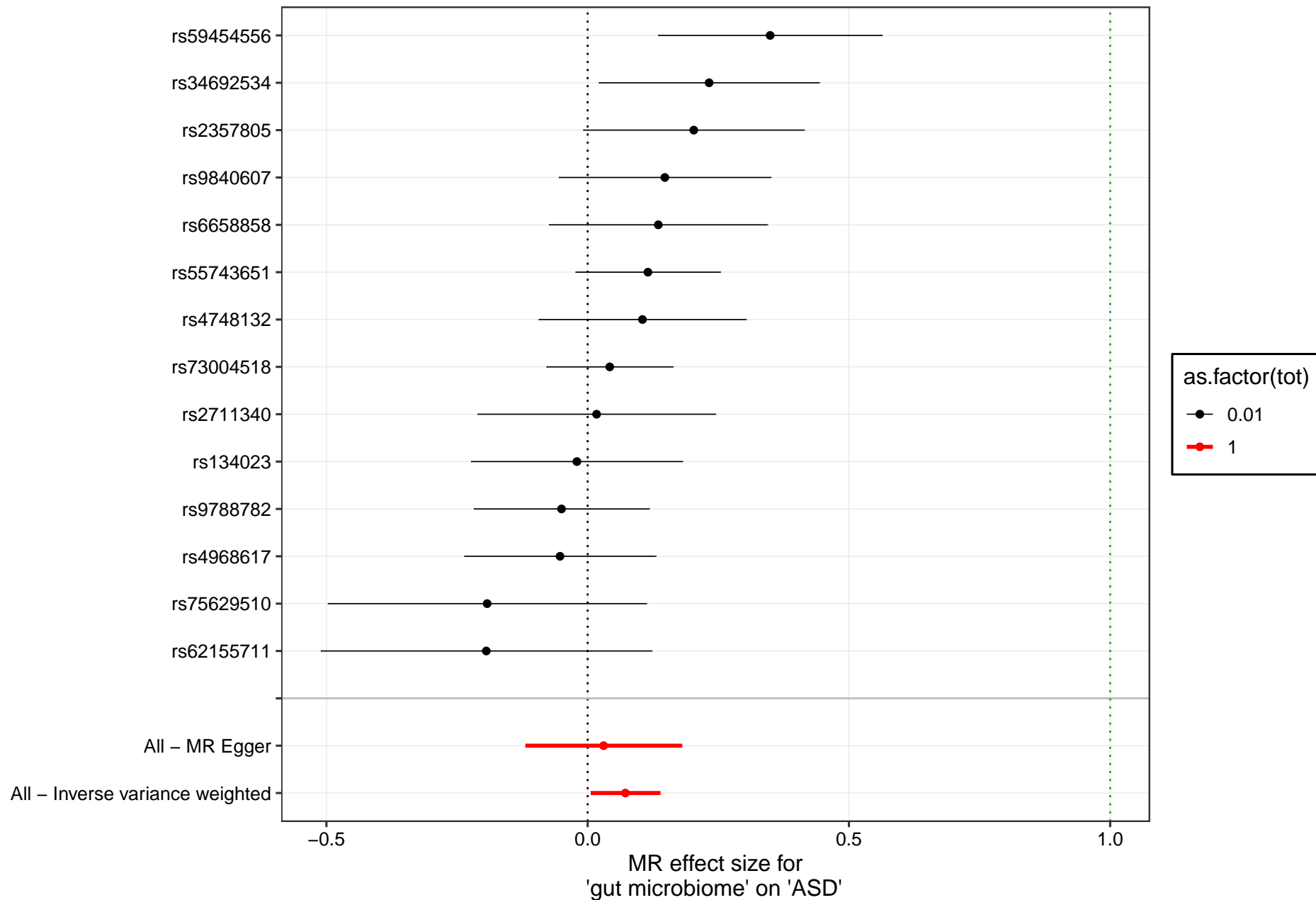

Supplement: Supplementary file 2 — Supplementary Material 2 [file 13568_2025_1969_MOESM2_ESM.zip › Revised supplementary materials/5 Forward MR analysis results/plot/forest_or_UBA7703.pdf]

# Forest Plot (OR): UBA8904

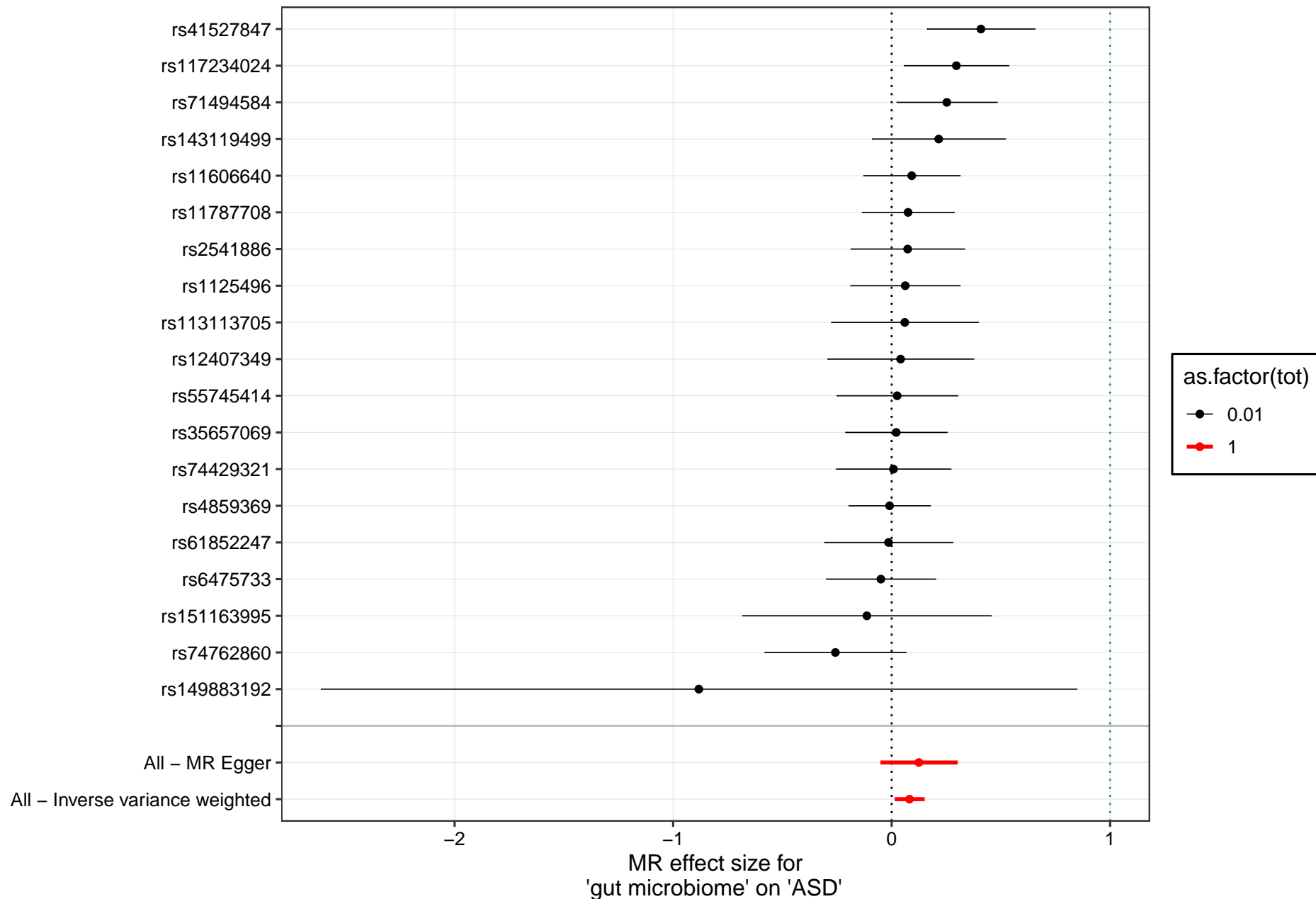

Supplement: Supplementary file 2 — Supplementary Material 2 [file 13568_2025_1969_MOESM2_ESM.zip › Revised supplementary materials/5 Forward MR analysis results/plot/forest_or_UBA8904.pdf]

# Forest Plot (OR): V9D3004

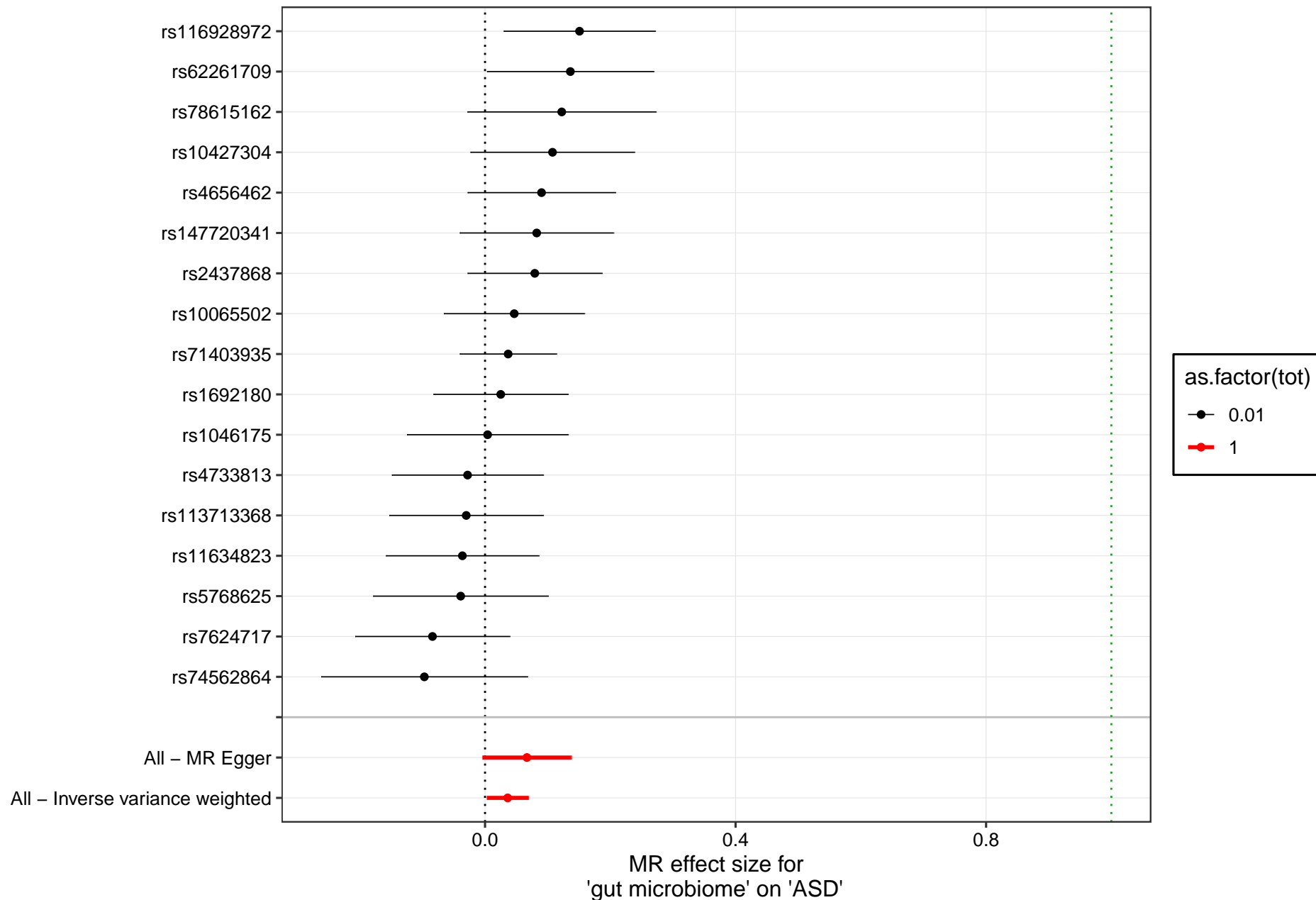

Supplement: Supplementary file 2 — Supplementary Material 2 [file 13568_2025_1969_MOESM2_ESM.zip › Revised supplementary materials/5 Forward MR analysis results/plot/forest_or_V9D3004.pdf]

# Funnel Plot (OR): *Acidaminococcus fermentans*

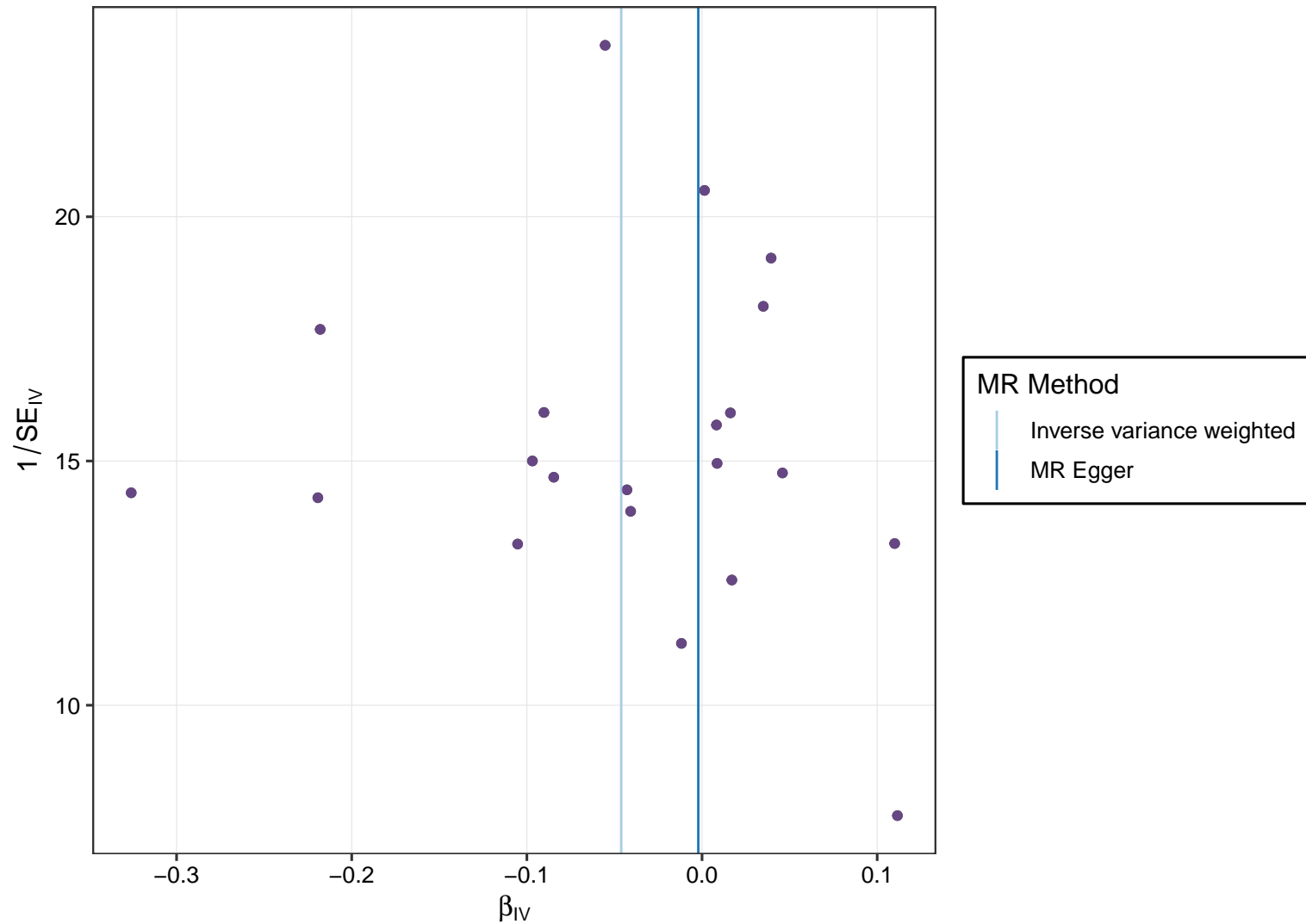

Supplement: Supplementary file 2 — Supplementary Material 2 [file 13568_2025_1969_MOESM2_ESM.zip › Revised supplementary materials/5 Forward MR analysis results/plot/funnel_or_Acidaminococcus fermentans.pdf]

**Funnel Plot (OR): CAG-475**

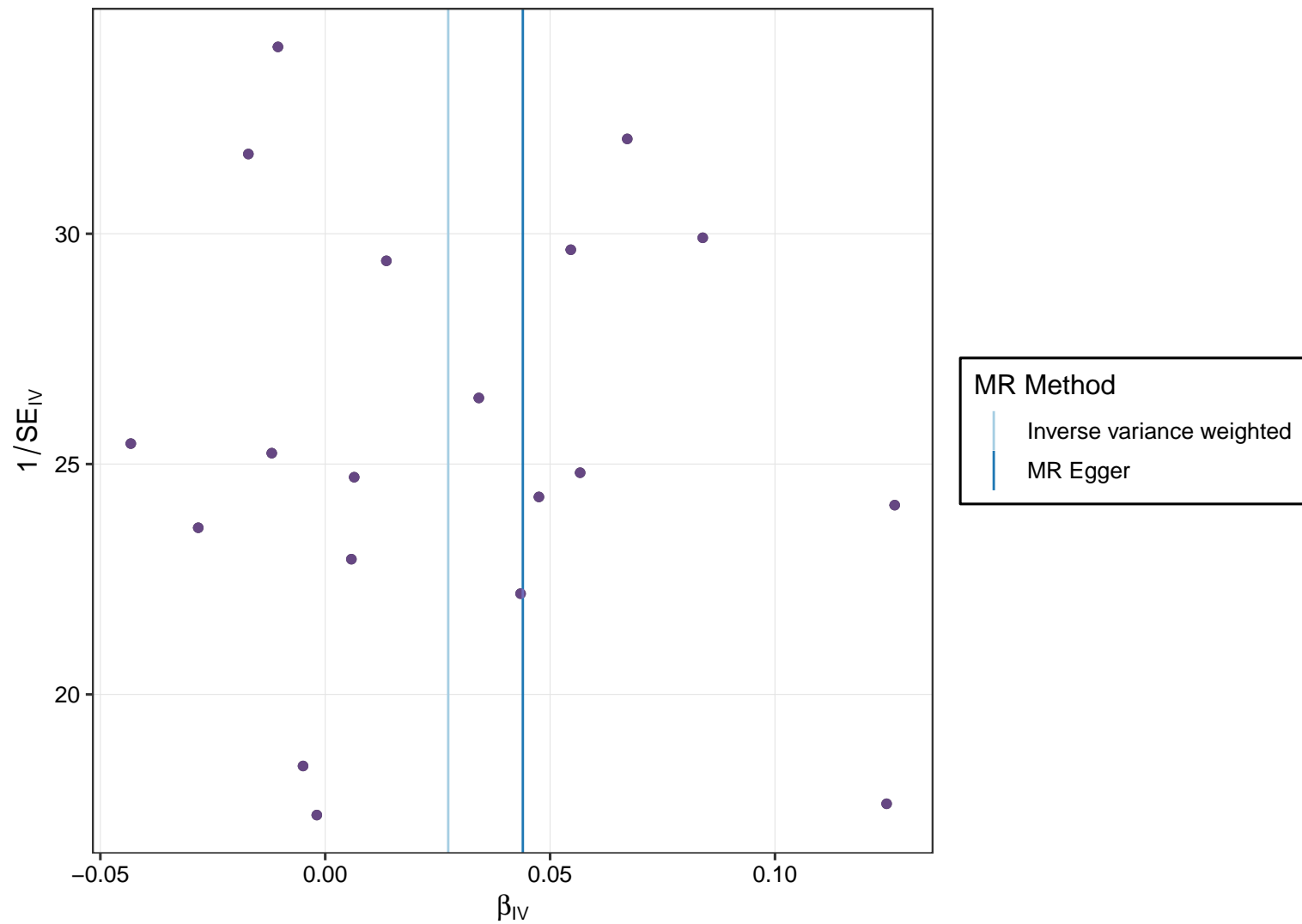

Supplement: Supplementary file 2 — Supplementary Material 2 [file 13568_2025_1969_MOESM2_ESM.zip › Revised supplementary materials/5 Forward MR analysis results/plot/funnel_or_CAG-475.pdf]

# Funnel Plot (OR): CAG-510 sp002432425

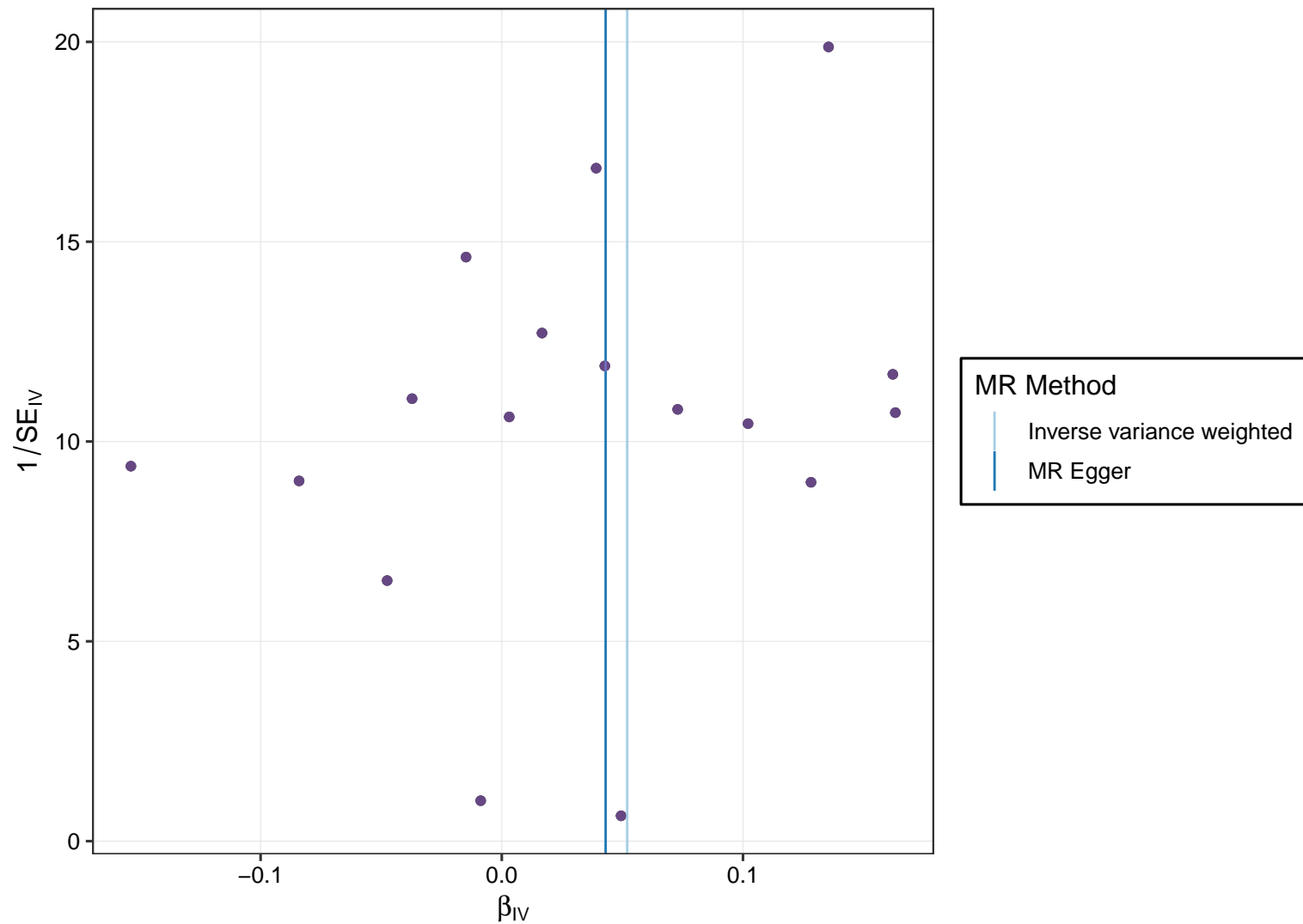

Supplement: Supplementary file 2 — Supplementary Material 2 [file 13568_2025_1969_MOESM2_ESM.zip › Revised supplementary materials/5 Forward MR analysis results/plot/funnel_or_CAG-510 sp002432425.pdf]

**Funnel Plot (OR): CAG-884**

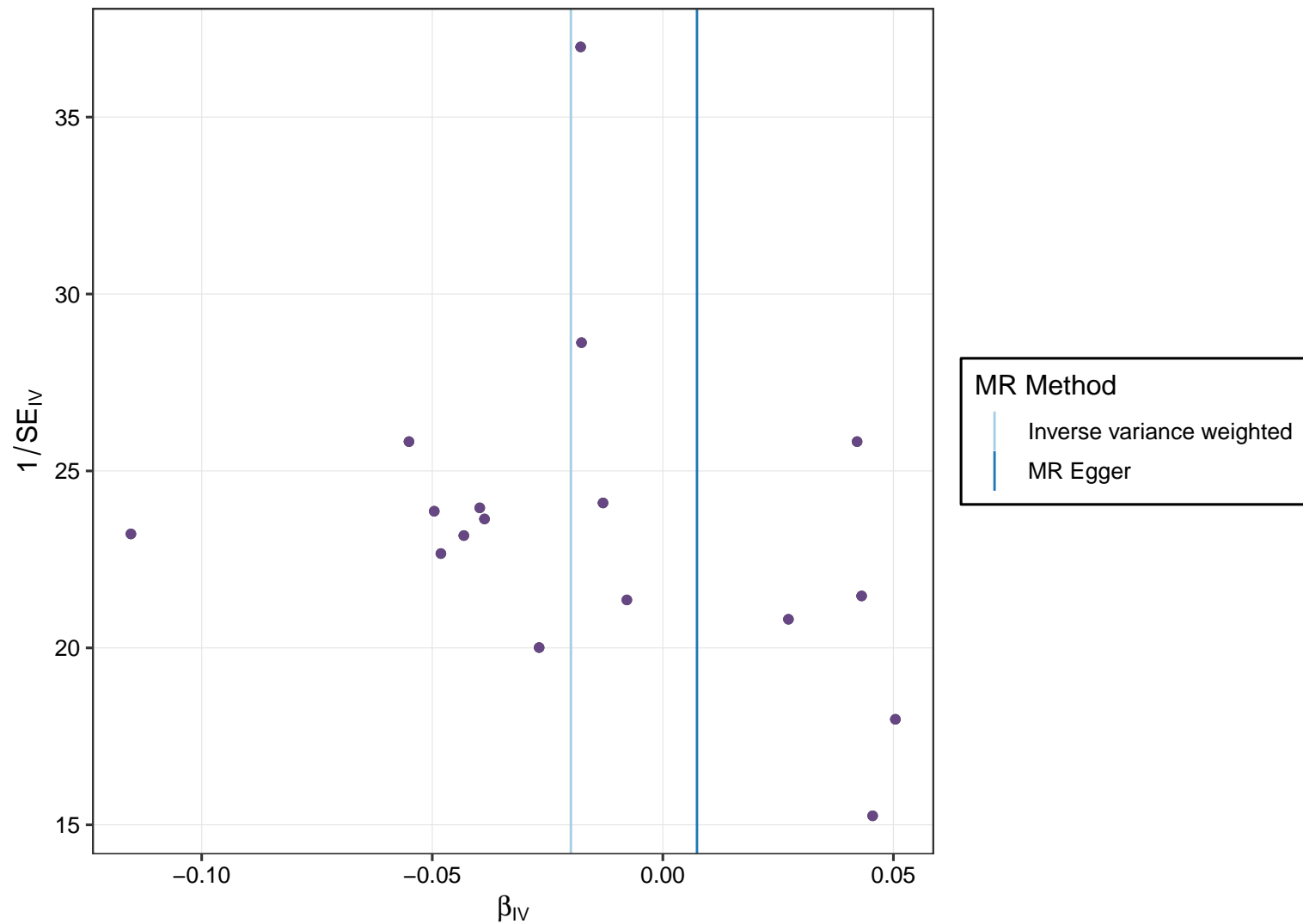

Supplement: Supplementary file 2 — Supplementary Material 2 [file 13568_2025_1969_MOESM2_ESM.zip › Revised supplementary materials/5 Forward MR analysis results/plot/funnel_or_CAG-884.pdf]

**Funnel Plot (OR): Coprobacillus**

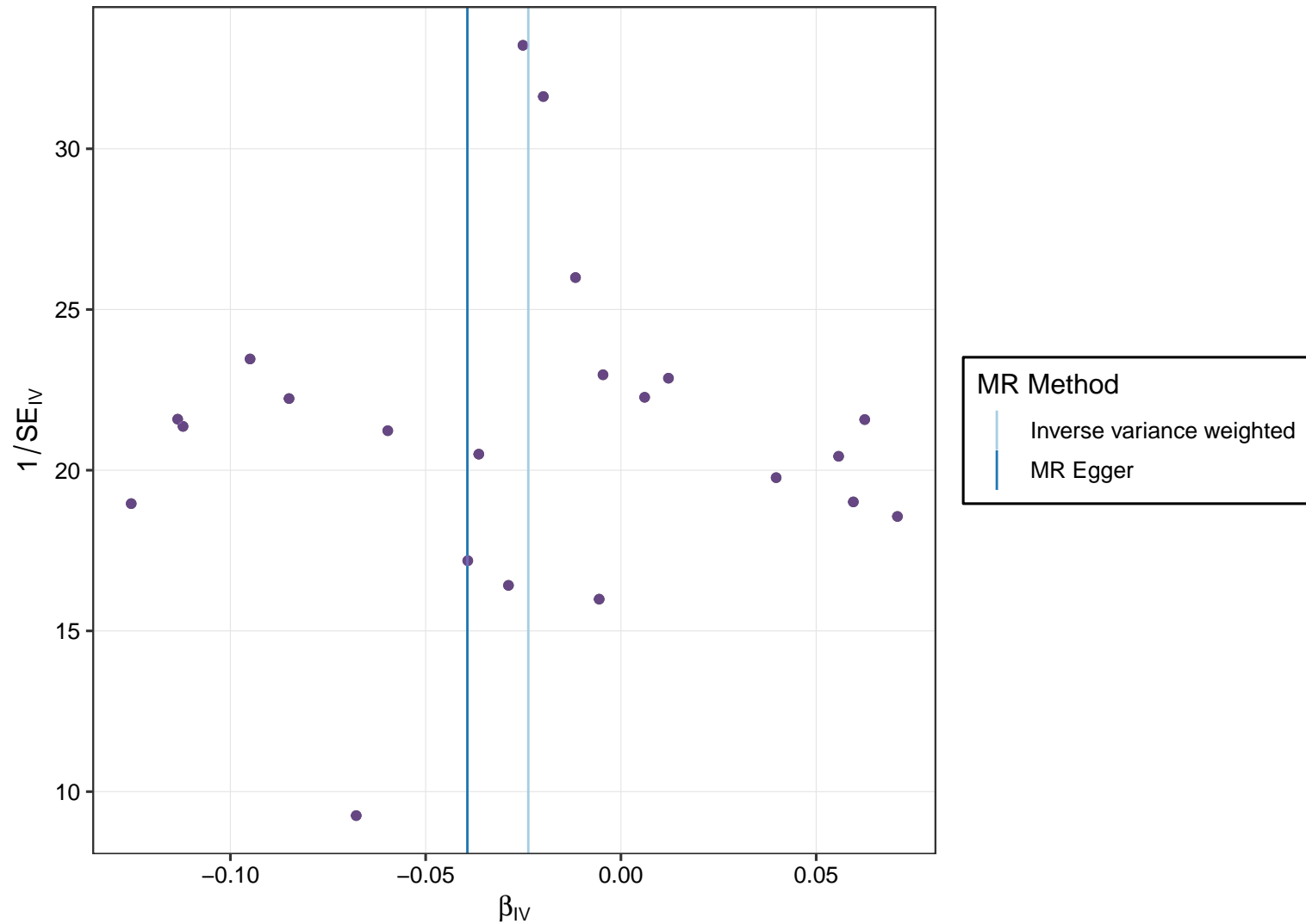

Supplement: Supplementary file 2 — Supplementary Material 2 [file 13568_2025_1969_MOESM2_ESM.zip › Revised supplementary materials/5 Forward MR analysis results/plot/funnel_or_Coprobacillus.pdf]

# Funnel Plot (OR): *Coprobacter secundus*

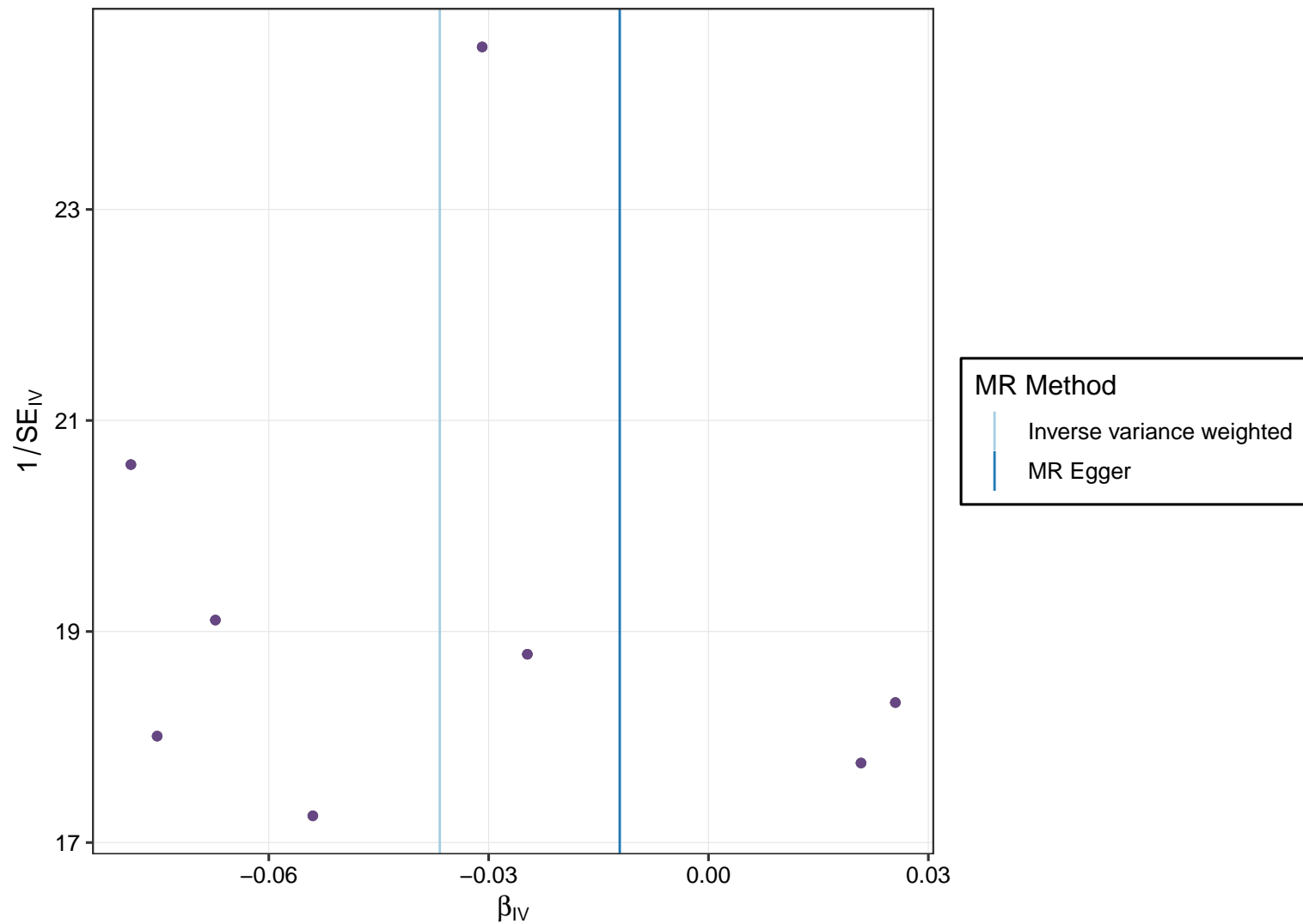

Supplement: Supplementary file 2 — Supplementary Material 2 [file 13568_2025_1969_MOESM2_ESM.zip › Revised supplementary materials/5 Forward MR analysis results/plot/funnel_or_Coprobacter secundus.pdf]

Funnel Plot (OR): DTU024 sp002411105

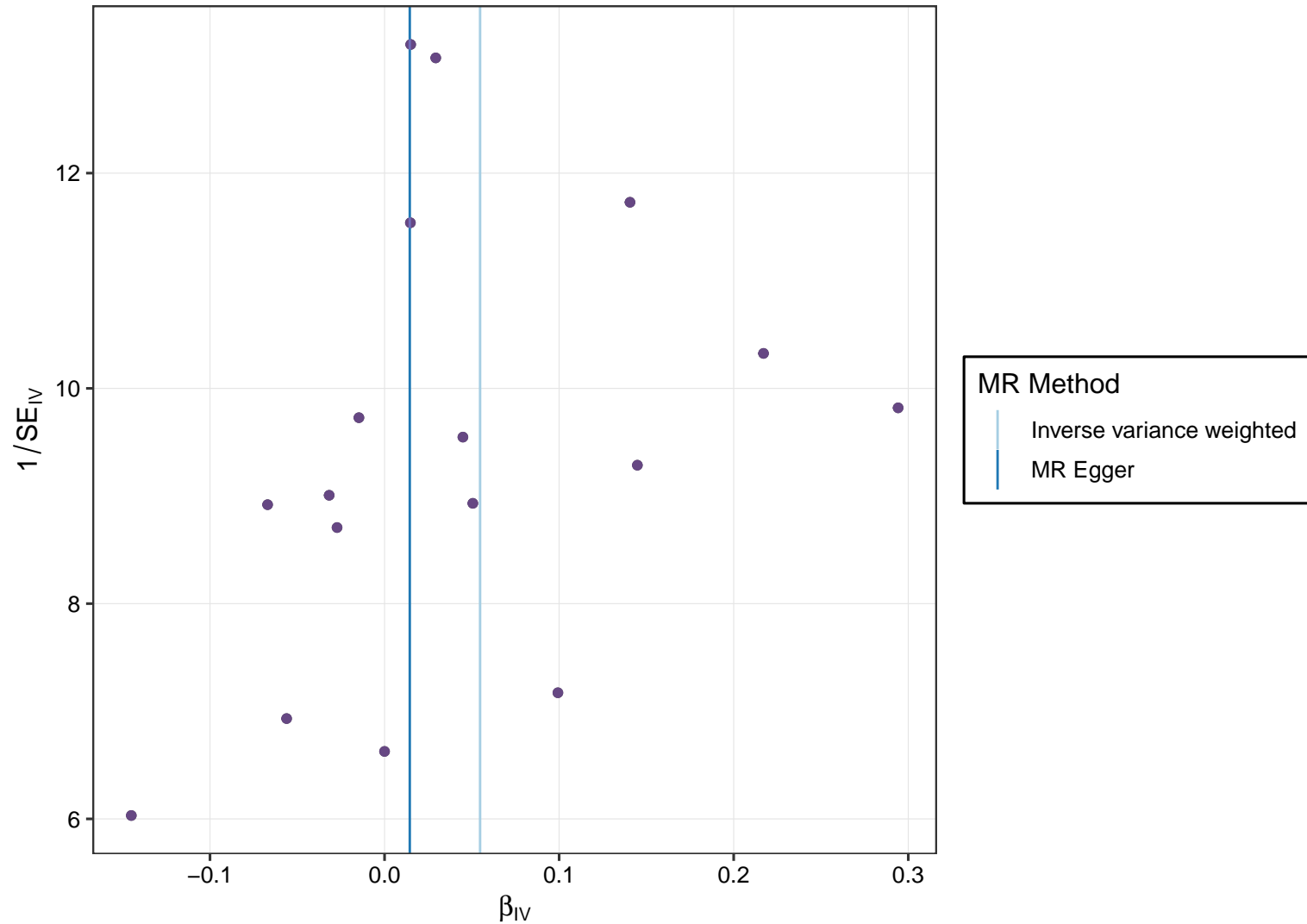

Supplement: Supplementary file 2 — Supplementary Material 2 [file 13568_2025_1969_MOESM2_ESM.zip › Revised supplementary materials/5 Forward MR analysis results/plot/funnel_or_DTU024 sp002411105.pdf]

# Funnel Plot (OR): Endozoicomonadaceae

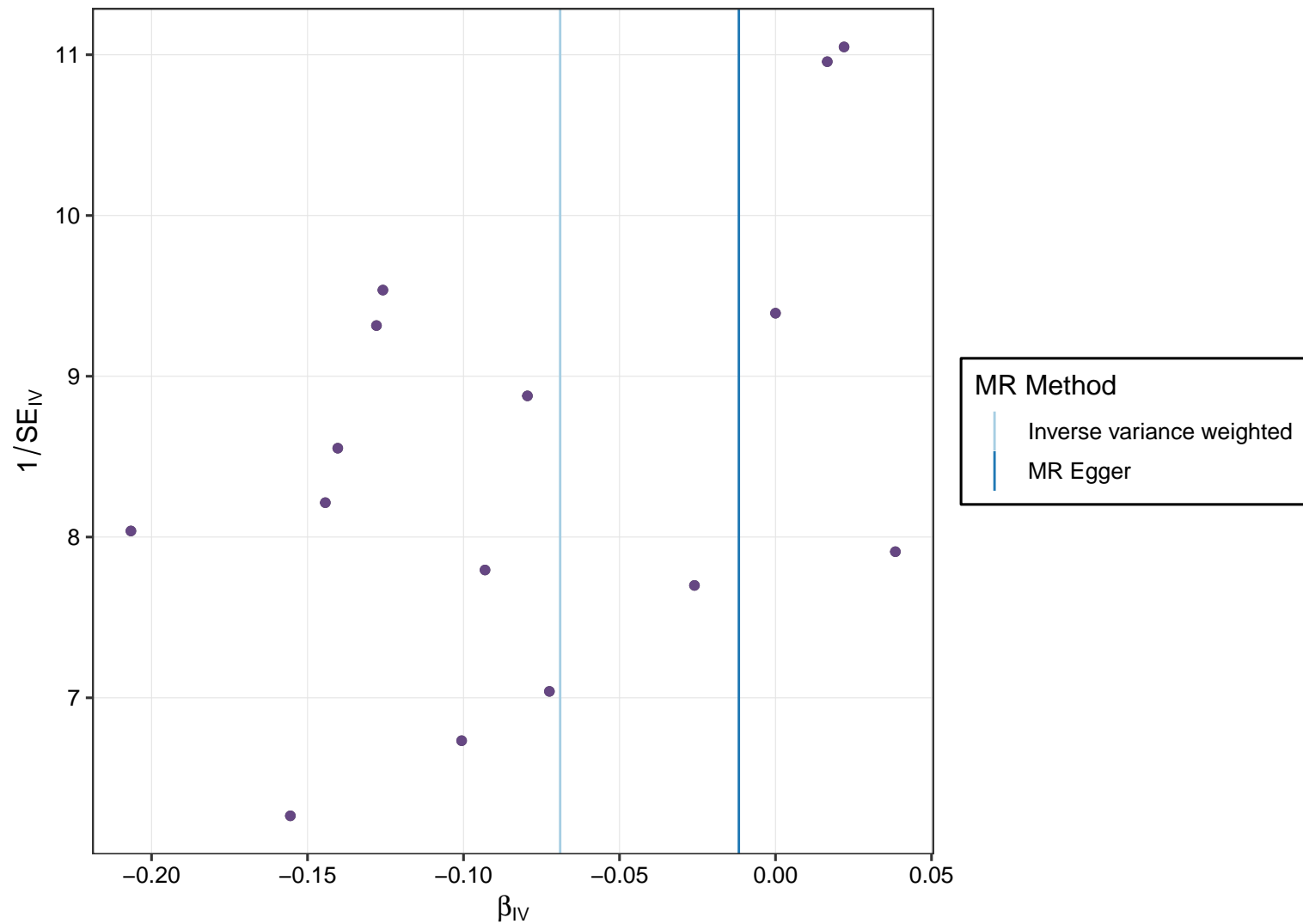

Supplement: Supplementary file 2 — Supplementary Material 2 [file 13568_2025_1969_MOESM2_ESM.zip › Revised supplementary materials/5 Forward MR analysis results/plot/funnel_or_Endozoicomonadaceae.pdf]

**Funnel Plot (OR): Enorma massiliensis**

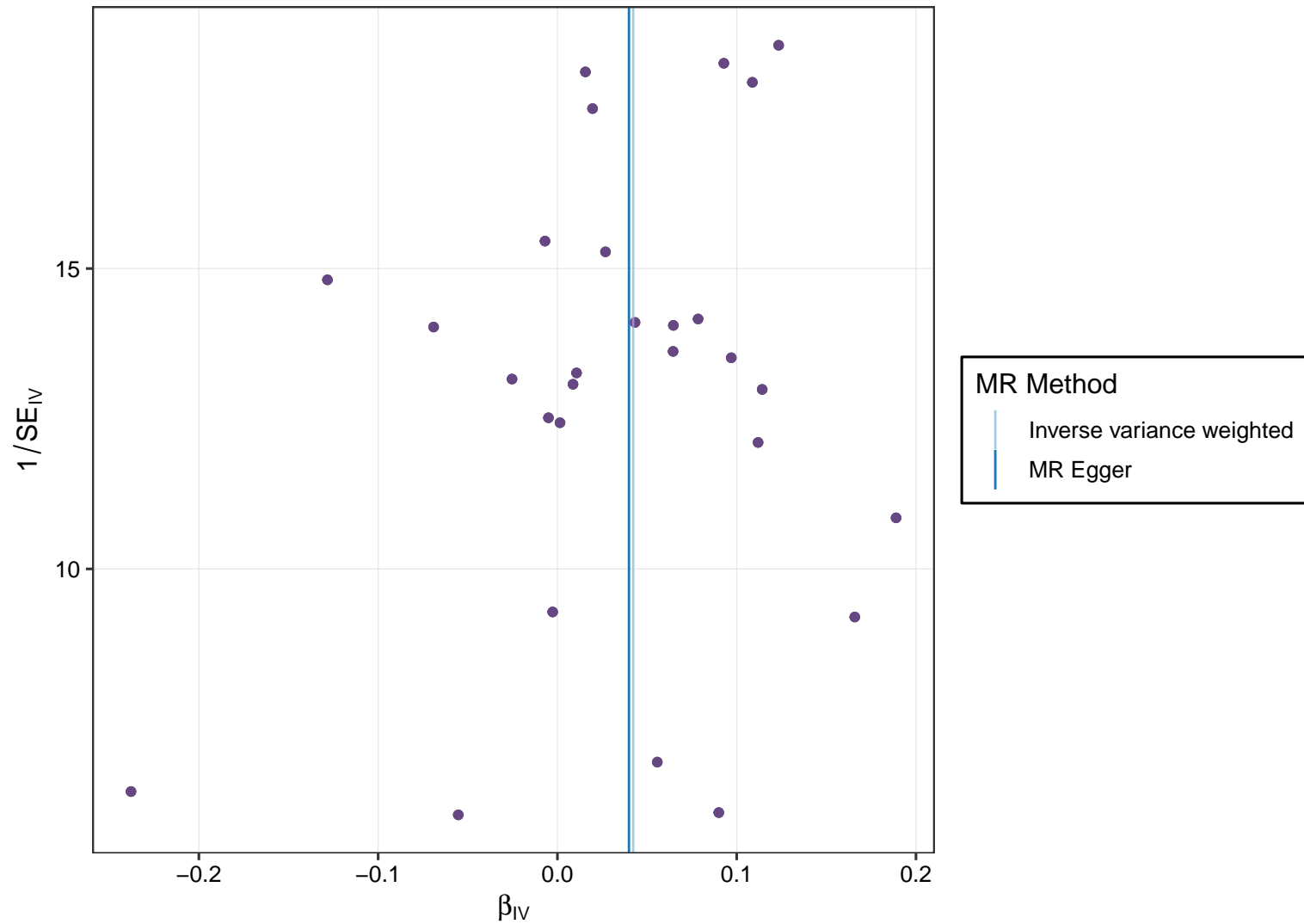

Supplement: Supplementary file 2 — Supplementary Material 2 [file 13568_2025_1969_MOESM2_ESM.zip › Revised supplementary materials/5 Forward MR analysis results/plot/funnel_or_Enorma massiliensis.pdf]

**Funnel Plot (OR): Enterococcus faecalis**

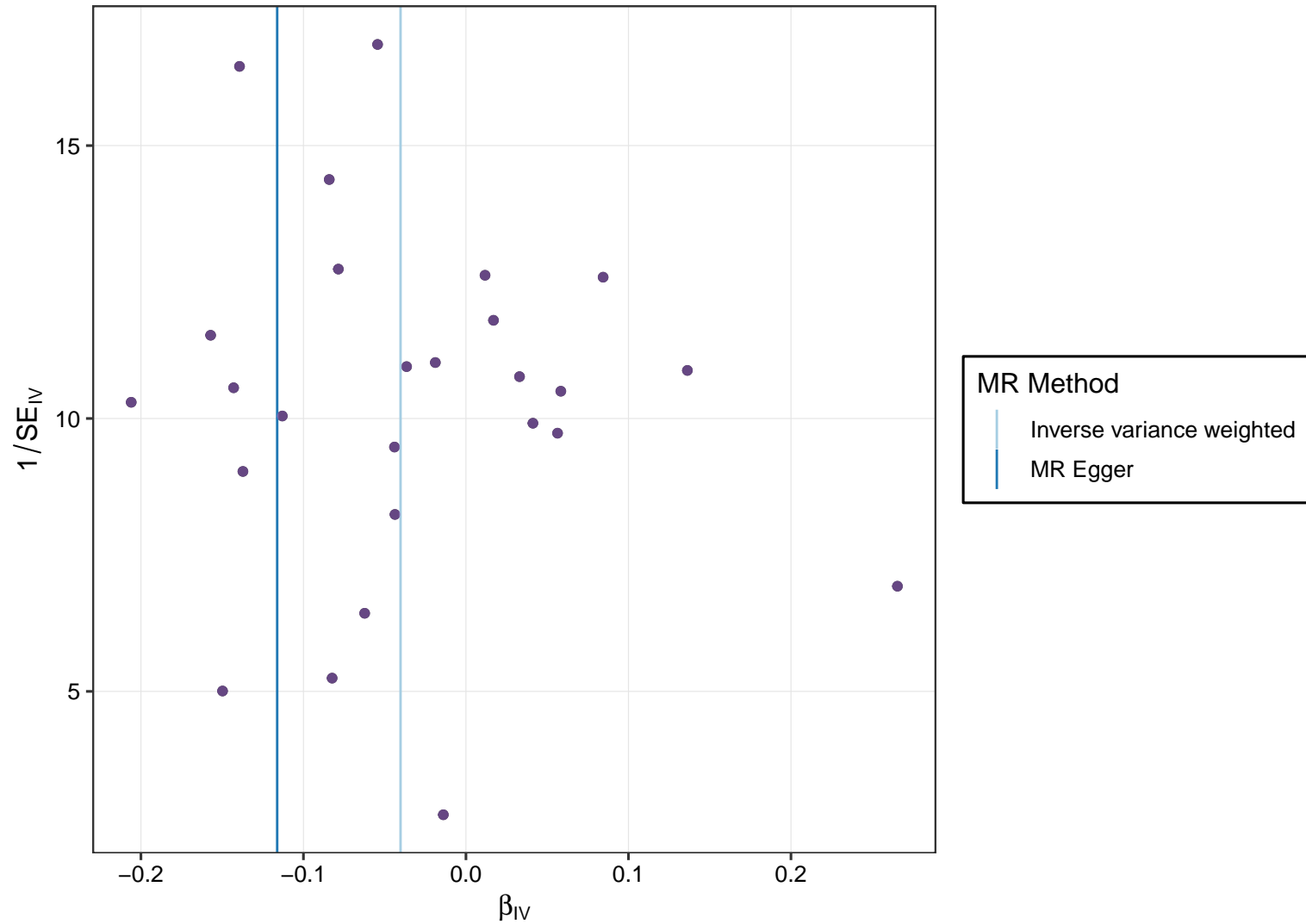

Supplement: Supplementary file 2 — Supplementary Material 2 [file 13568_2025_1969_MOESM2_ESM.zip › Revised supplementary materials/5 Forward MR analysis results/plot/funnel_or_Enterococcus faecalis.pdf]

**Funnel Plot (OR): *Eubacterium callanderi***

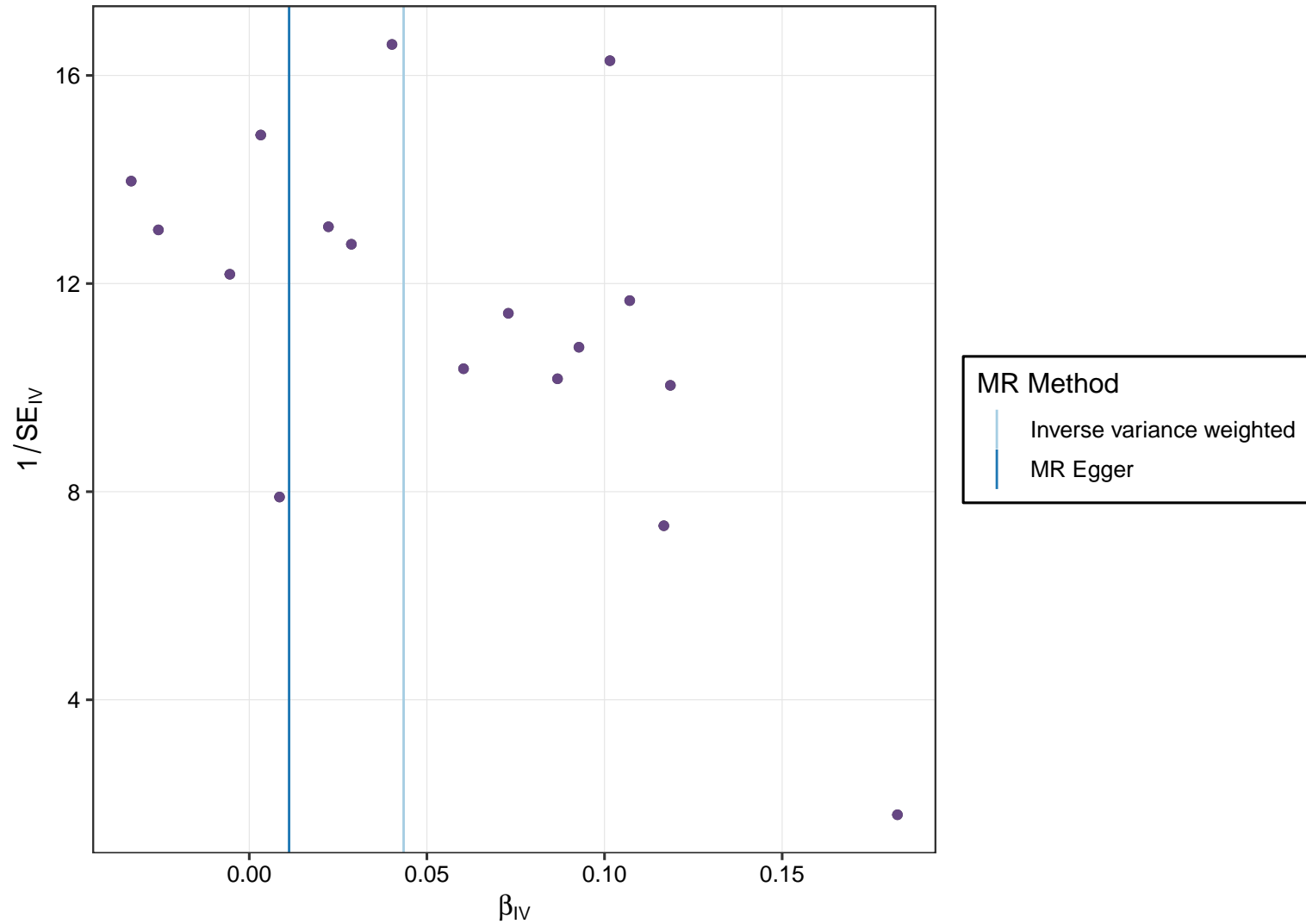

Supplement: Supplementary file 2 — Supplementary Material 2 [file 13568_2025_1969_MOESM2_ESM.zip › Revised supplementary materials/5 Forward MR analysis results/plot/funnel_or_Eubacterium callanderi.pdf]

**Funnel Plot (OR): *Faecalicatena torques***

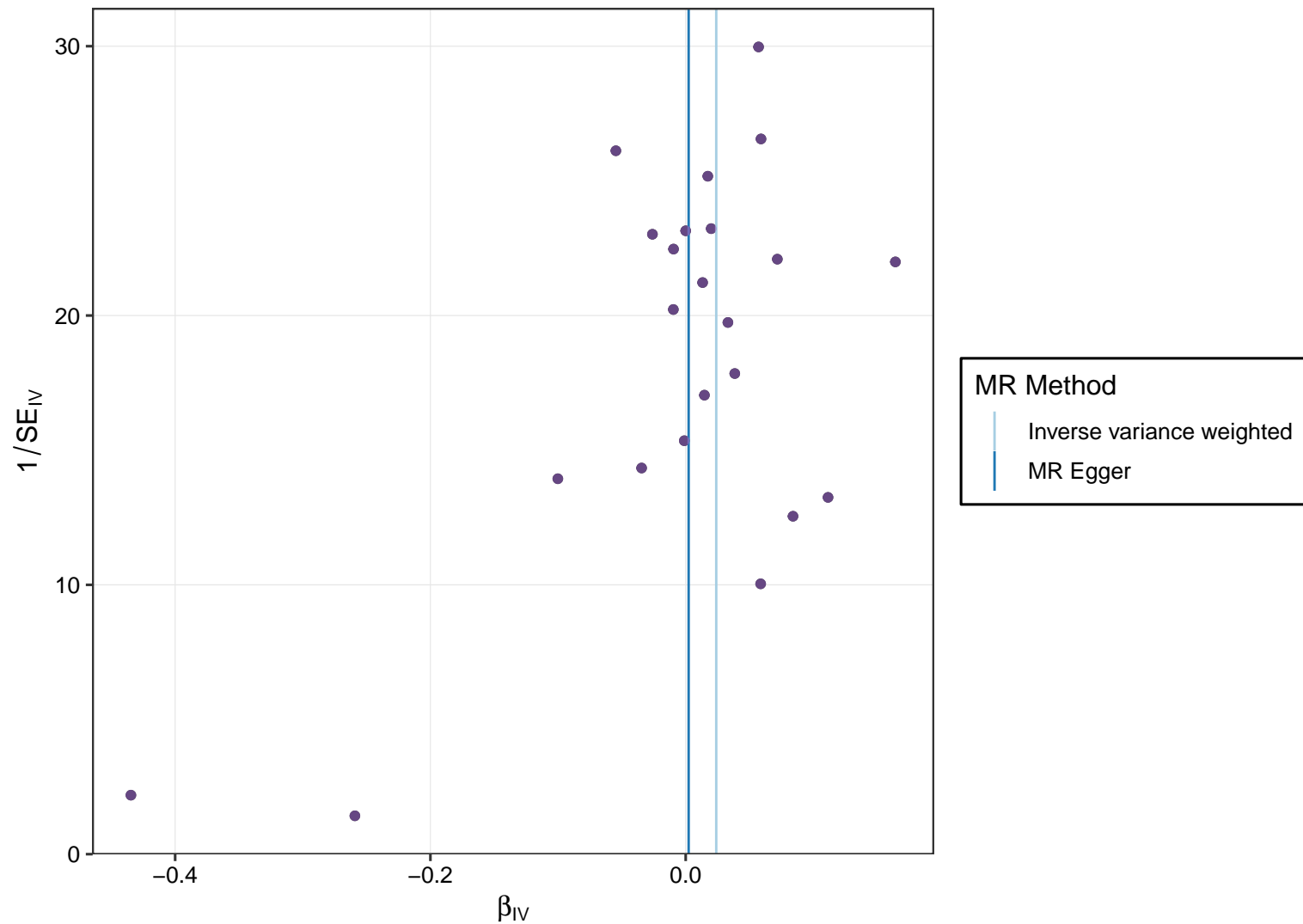

Supplement: Supplementary file 2 — Supplementary Material 2 [file 13568_2025_1969_MOESM2_ESM.zip › Revised supplementary materials/5 Forward MR analysis results/plot/funnel_or_Faecalicatena torques.pdf]

**Funnel Plot (OR): Fibrobacteria**

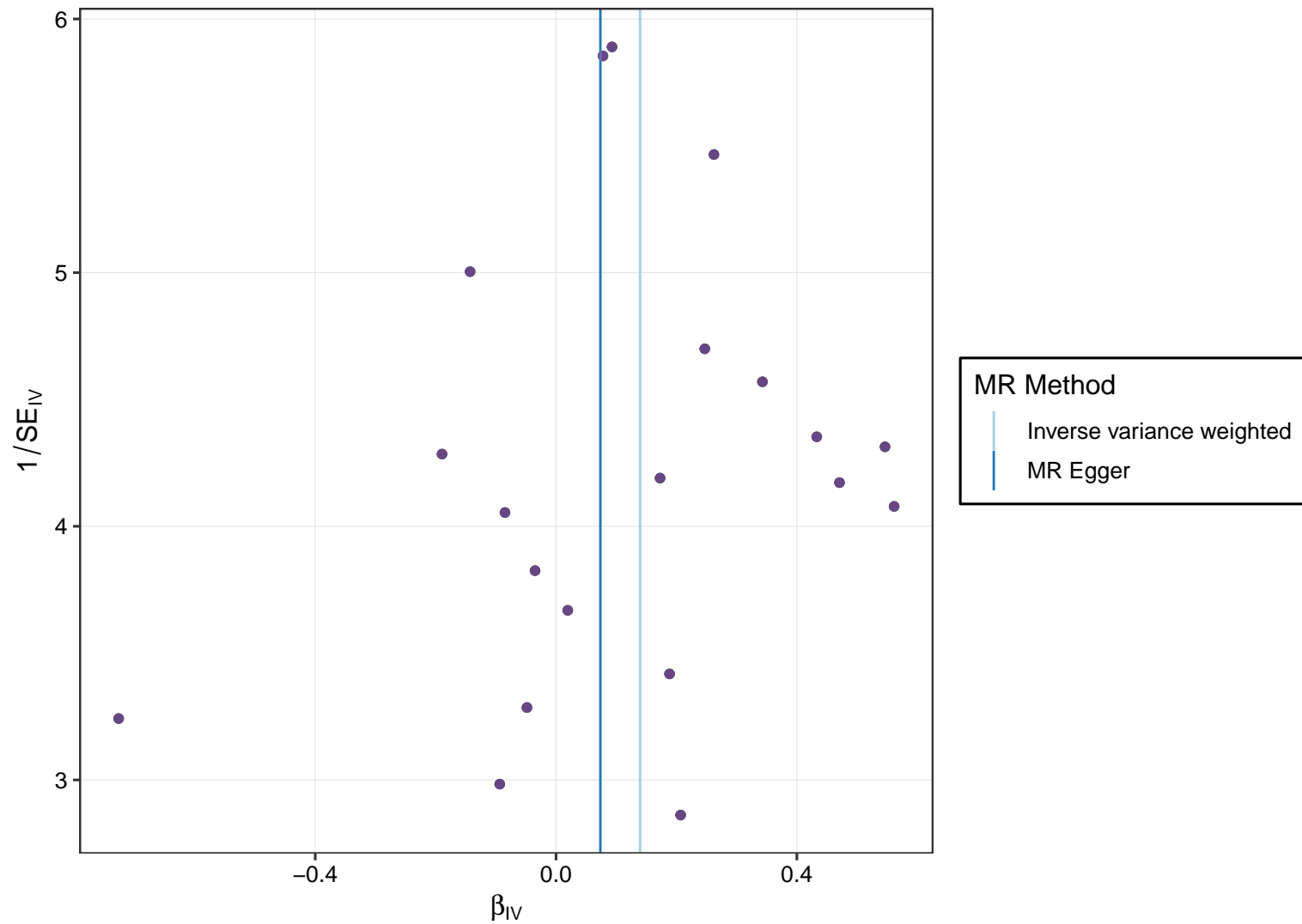

Supplement: Supplementary file 2 — Supplementary Material 2 [file 13568_2025_1969_MOESM2_ESM.zip › Revised supplementary materials/5 Forward MR analysis results/plot/funnel_or_Fibrobacteria.pdf]

**Funnel Plot (OR): Francisellaceae**

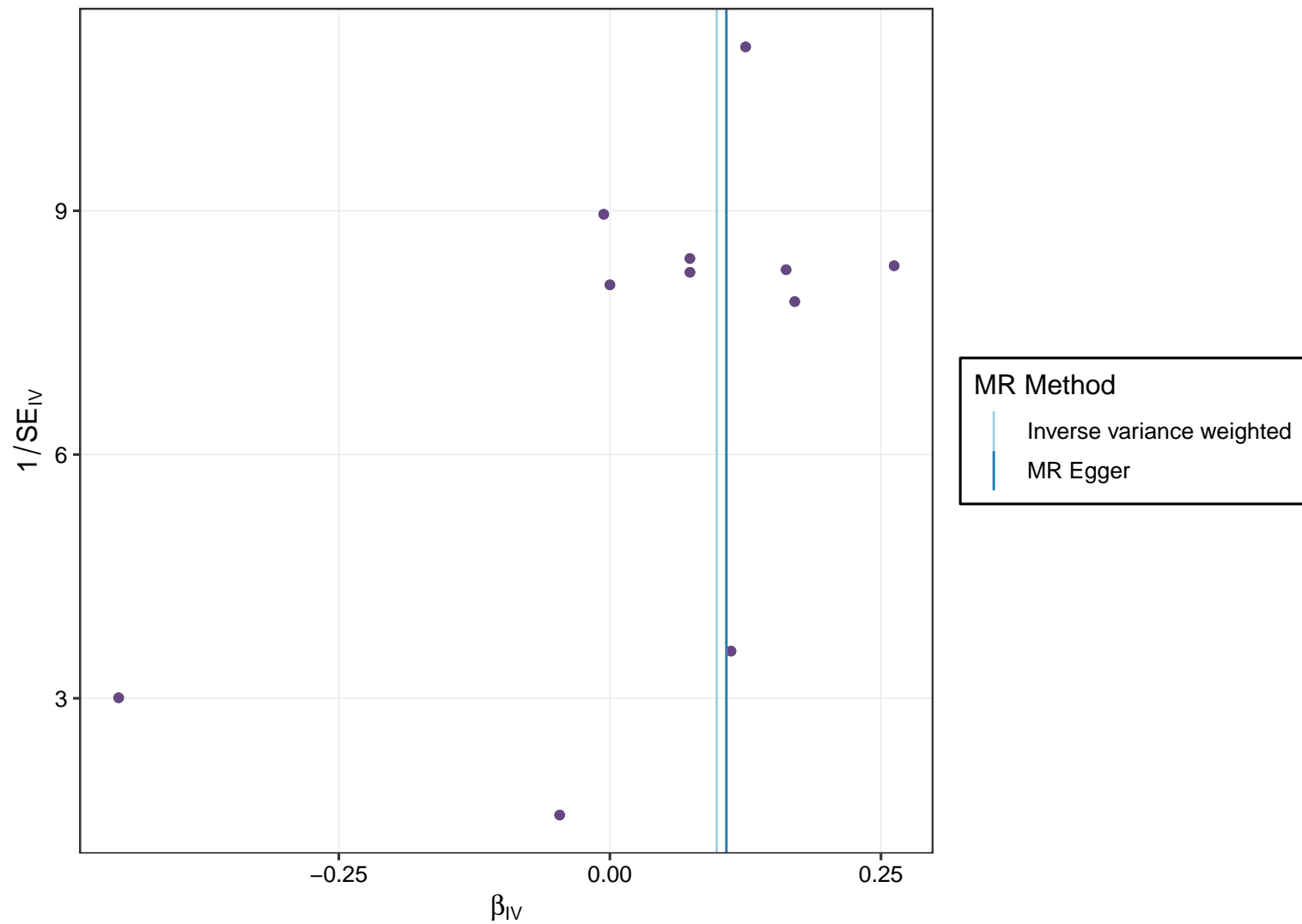

Supplement: Supplementary file 2 — Supplementary Material 2 [file 13568_2025_1969_MOESM2_ESM.zip › Revised supplementary materials/5 Forward MR analysis results/plot/funnel_or_Francisellaceae.pdf]

**Funnel Plot (OR): Geminocystis**

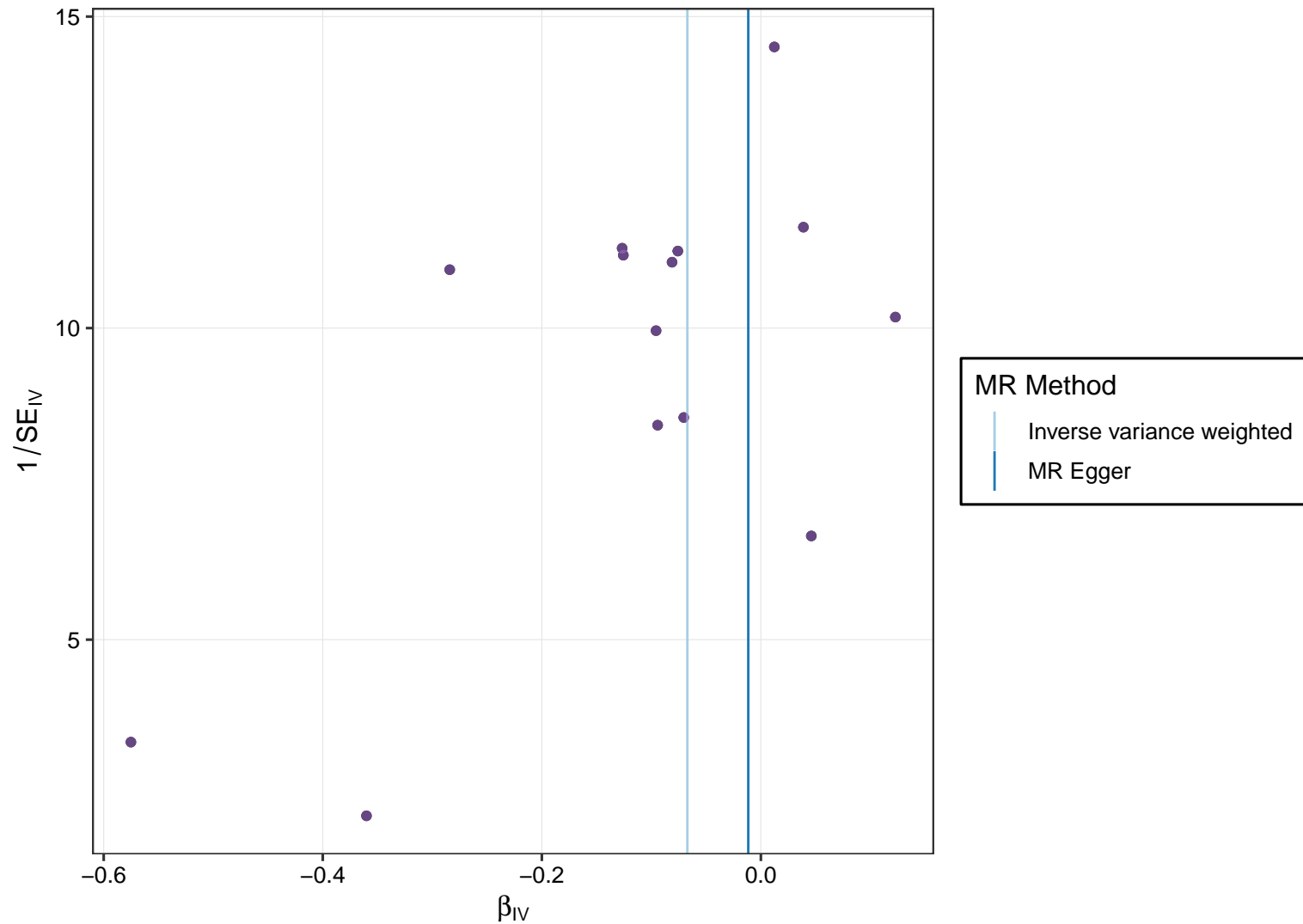

Supplement: Supplementary file 2 — Supplementary Material 2 [file 13568_2025_1969_MOESM2_ESM.zip › Revised supplementary materials/5 Forward MR analysis results/plot/funnel_or_Geminocystis.pdf]

**Funnel Plot (OR): koll11**

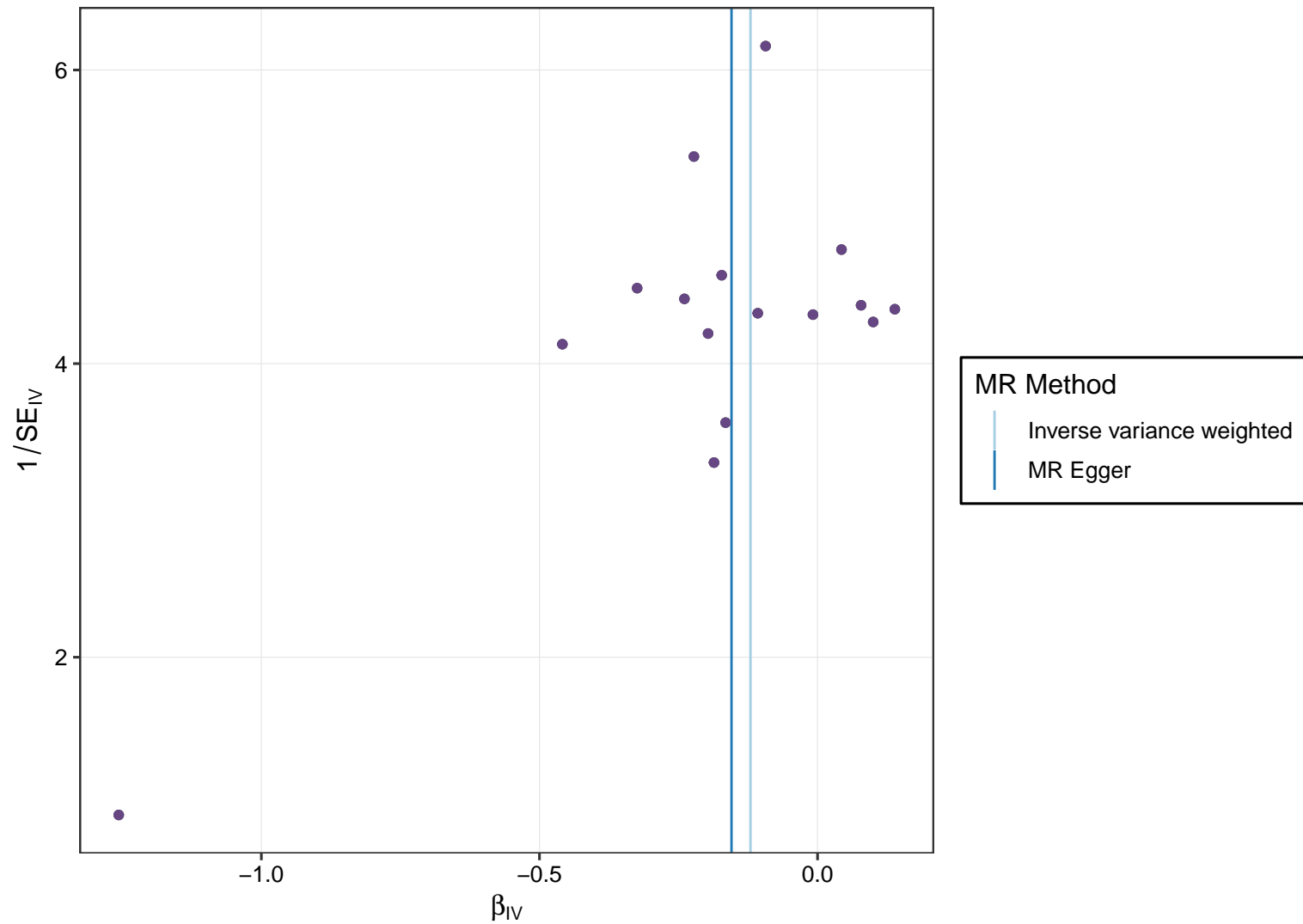

Supplement: Supplementary file 2 — Supplementary Material 2 [file 13568_2025_1969_MOESM2_ESM.zip › Revised supplementary materials/5 Forward MR analysis results/plot/funnel_or_koll11.pdf]

**Funnel Plot (OR): *Lachnospira rogosae***

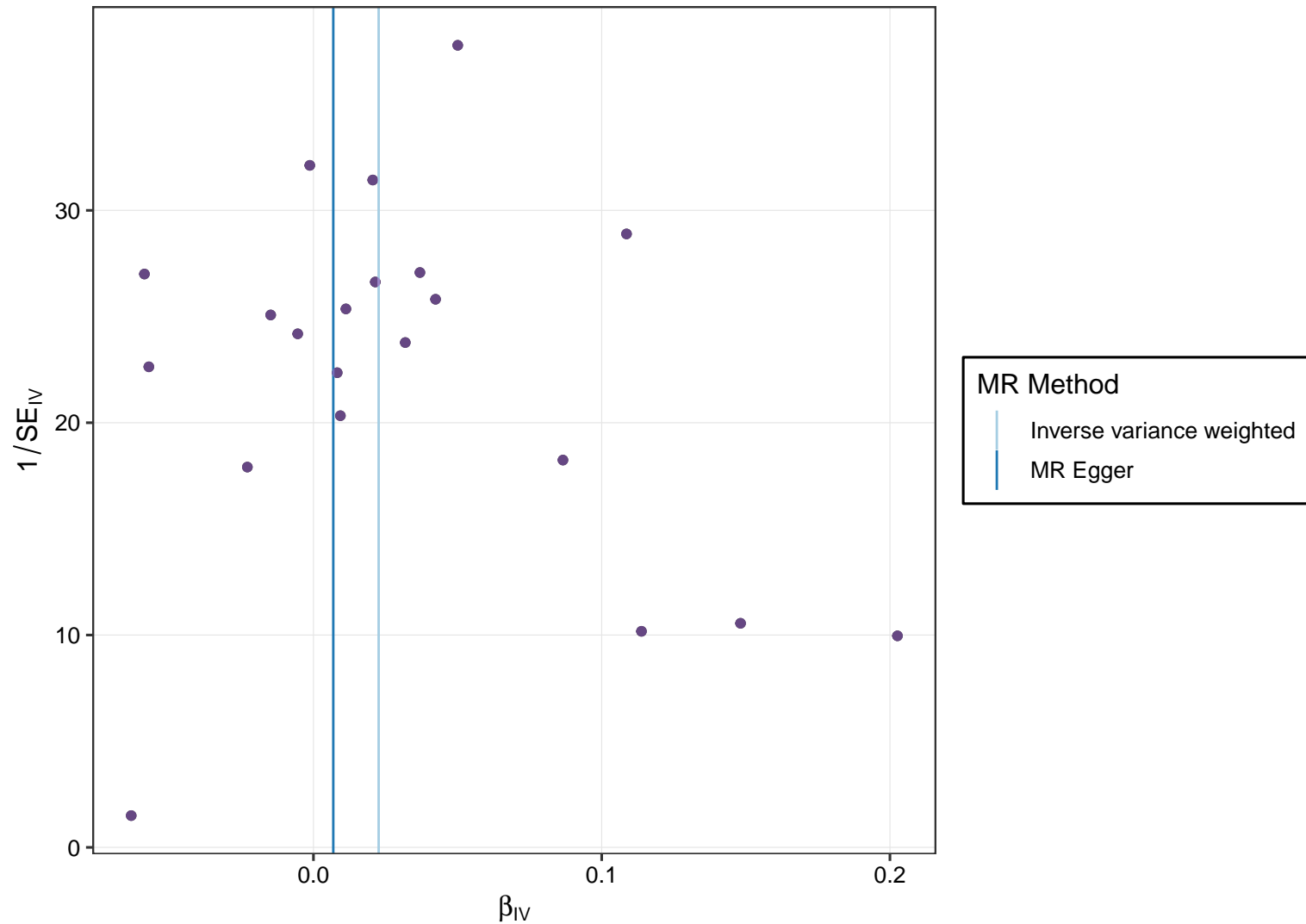

Supplement: Supplementary file 2 — Supplementary Material 2 [file 13568_2025_1969_MOESM2_ESM.zip › Revised supplementary materials/5 Forward MR analysis results/plot/funnel_or_Lachnospira rogosae.pdf]

**Funnel Plot (OR): Olsenella C**

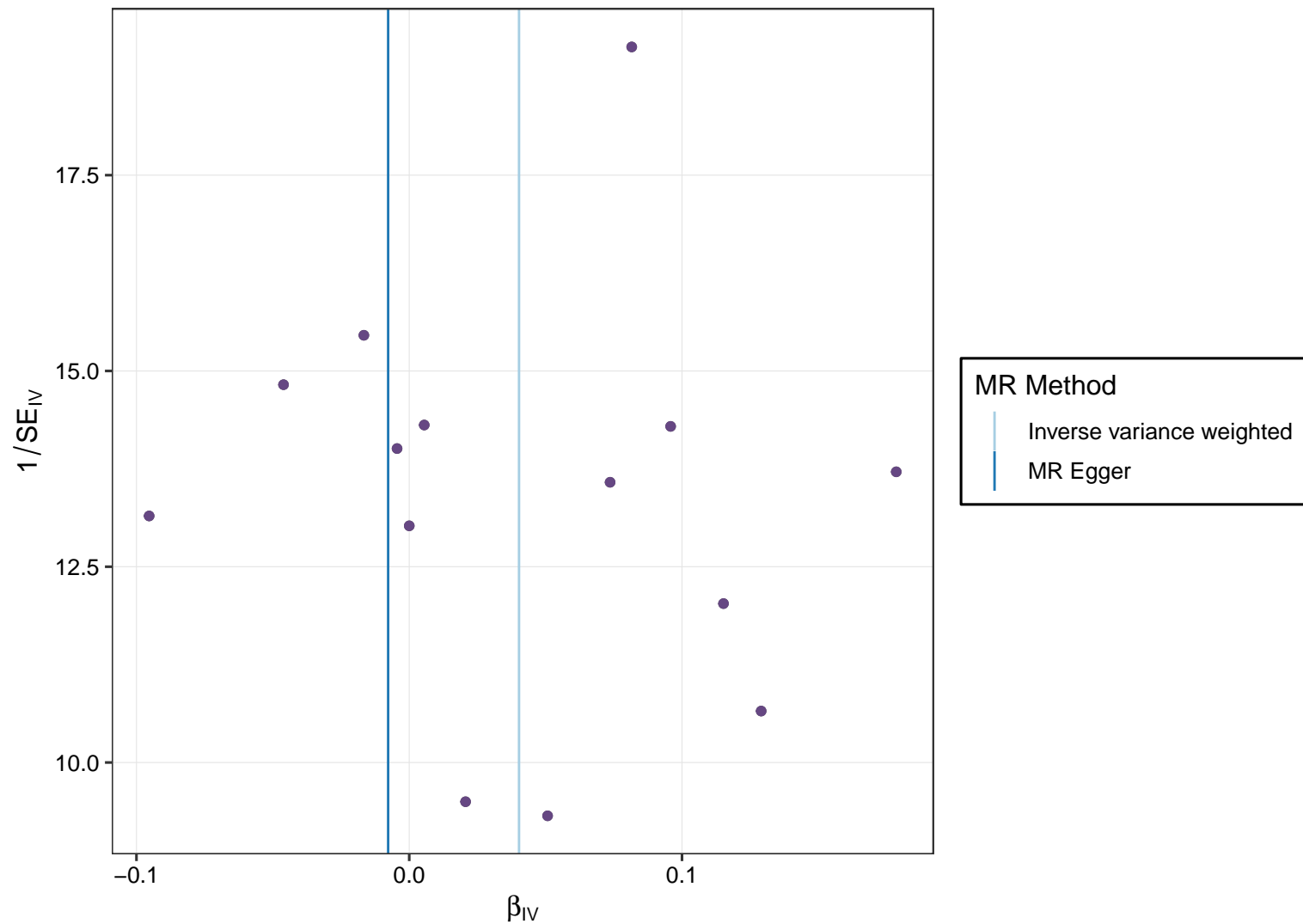

Supplement: Supplementary file 2 — Supplementary Material 2 [file 13568_2025_1969_MOESM2_ESM.zip › Revised supplementary materials/5 Forward MR analysis results/plot/funnel_or_Olsenella C.pdf]

**Funnel Plot (OR): Parabacteroides**

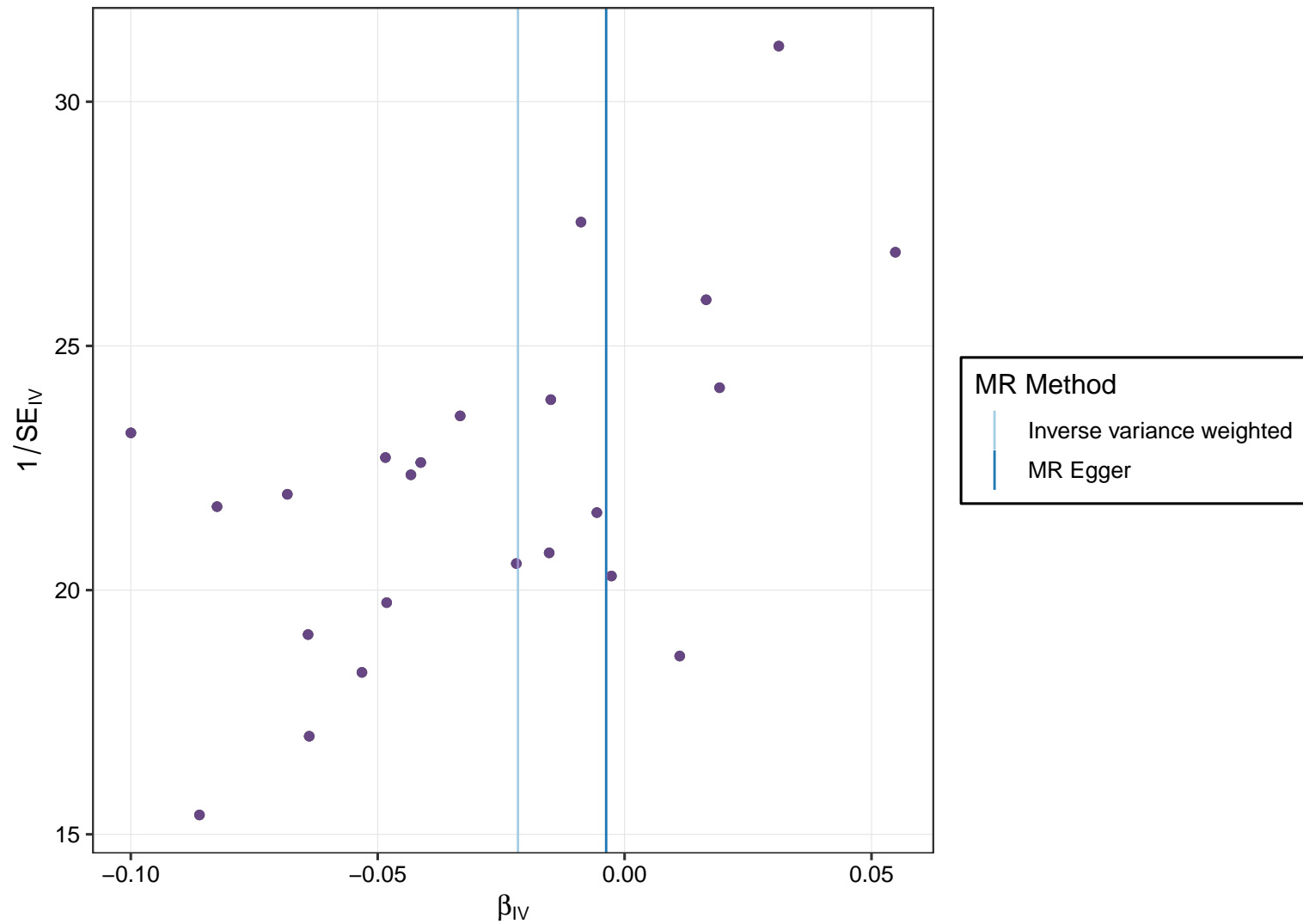

Supplement: Supplementary file 2 — Supplementary Material 2 [file 13568_2025_1969_MOESM2_ESM.zip › Revised supplementary materials/5 Forward MR analysis results/plot/funnel_or_Parabacteroides.pdf]

# Funnel Plot (OR): Prevotella sp002933775

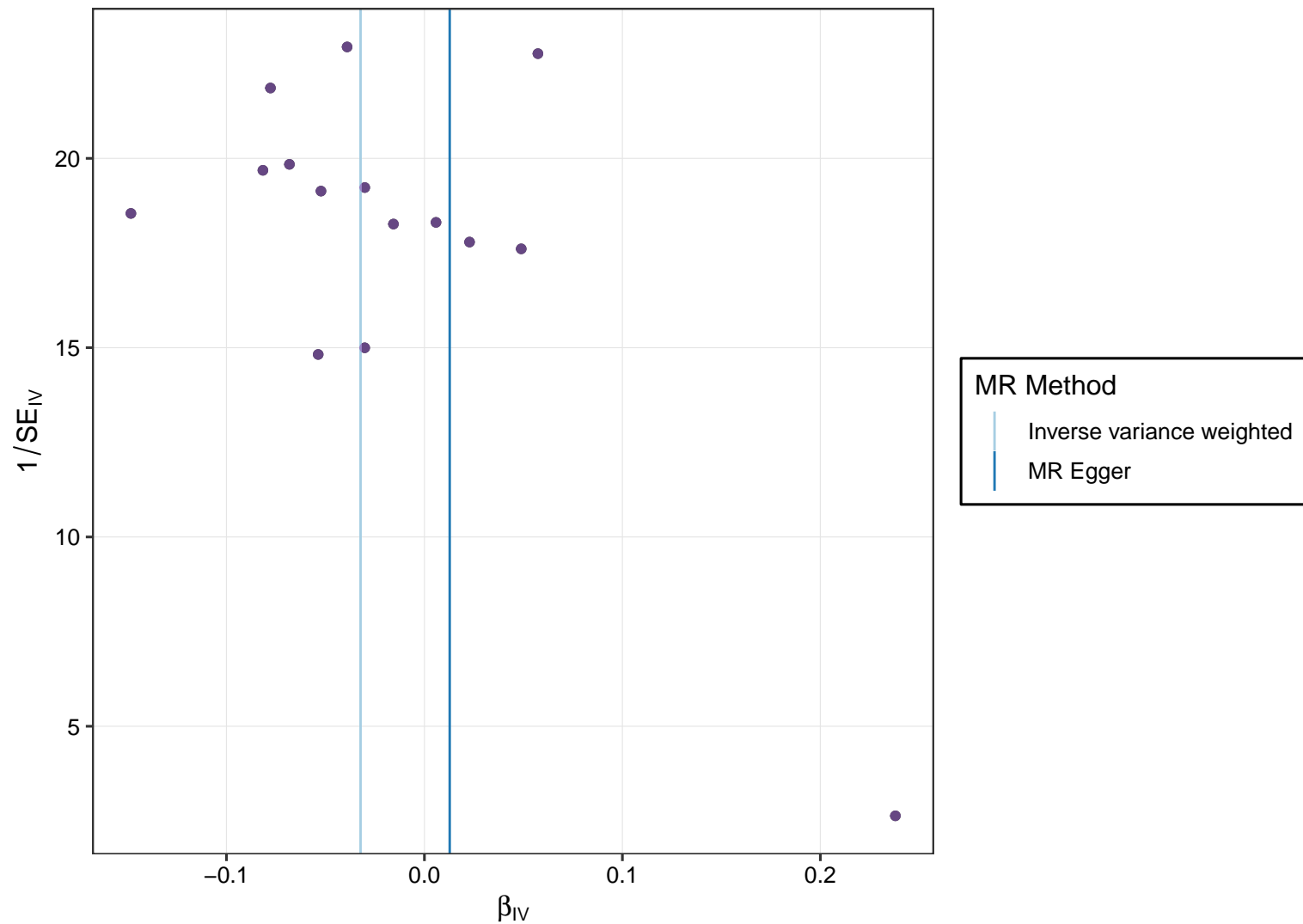

Supplement: Supplementary file 2 — Supplementary Material 2 [file 13568_2025_1969_MOESM2_ESM.zip › Revised supplementary materials/5 Forward MR analysis results/plot/funnel_or_Prevotella sp002933775.pdf]

**Funnel Plot (OR): UBA1066**

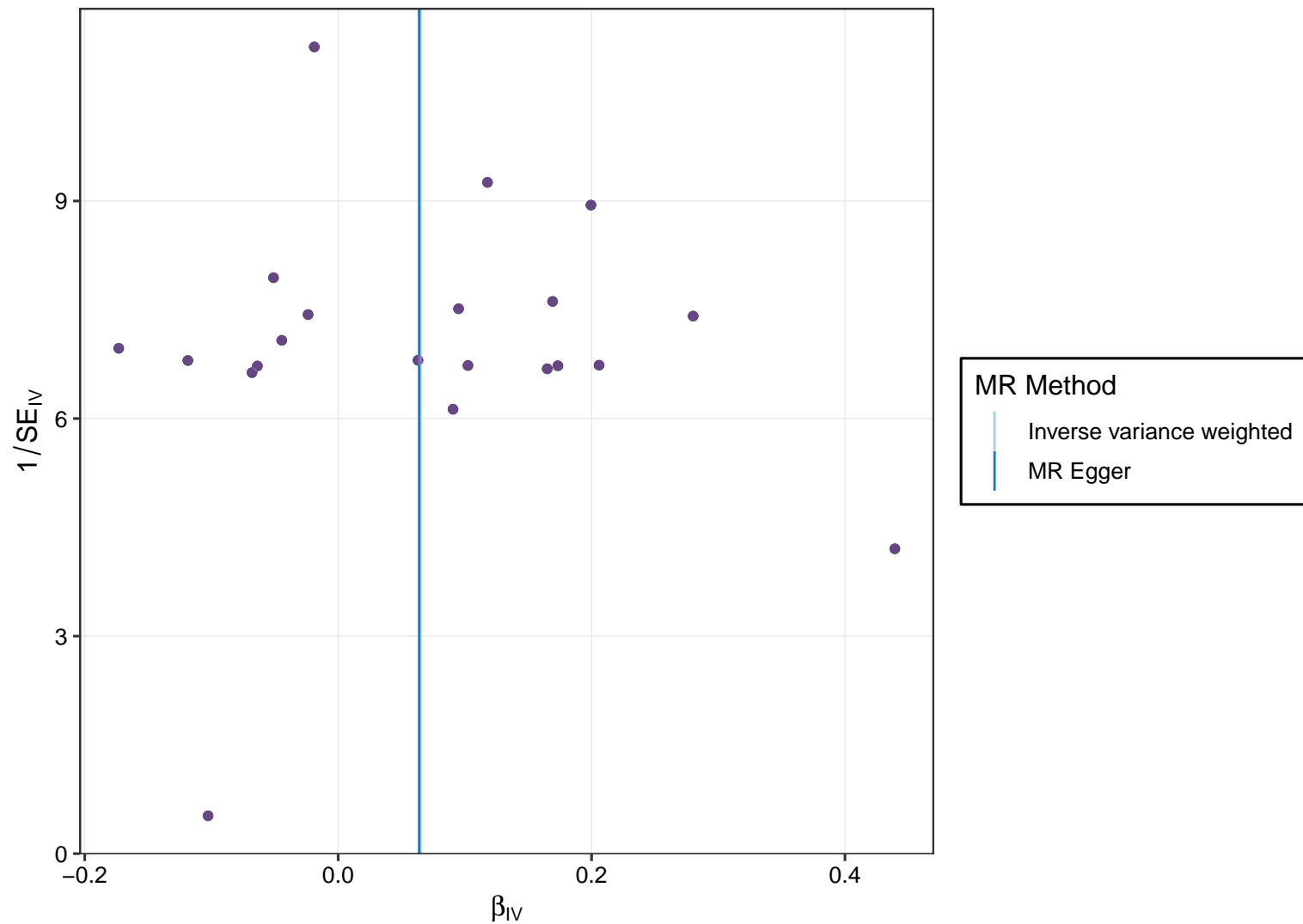

Supplement: Supplementary file 2 — Supplementary Material 2 [file 13568_2025_1969_MOESM2_ESM.zip › Revised supplementary materials/5 Forward MR analysis results/plot/funnel_or_UBA1066.pdf]

Funnel Plot (OR): UBA7703

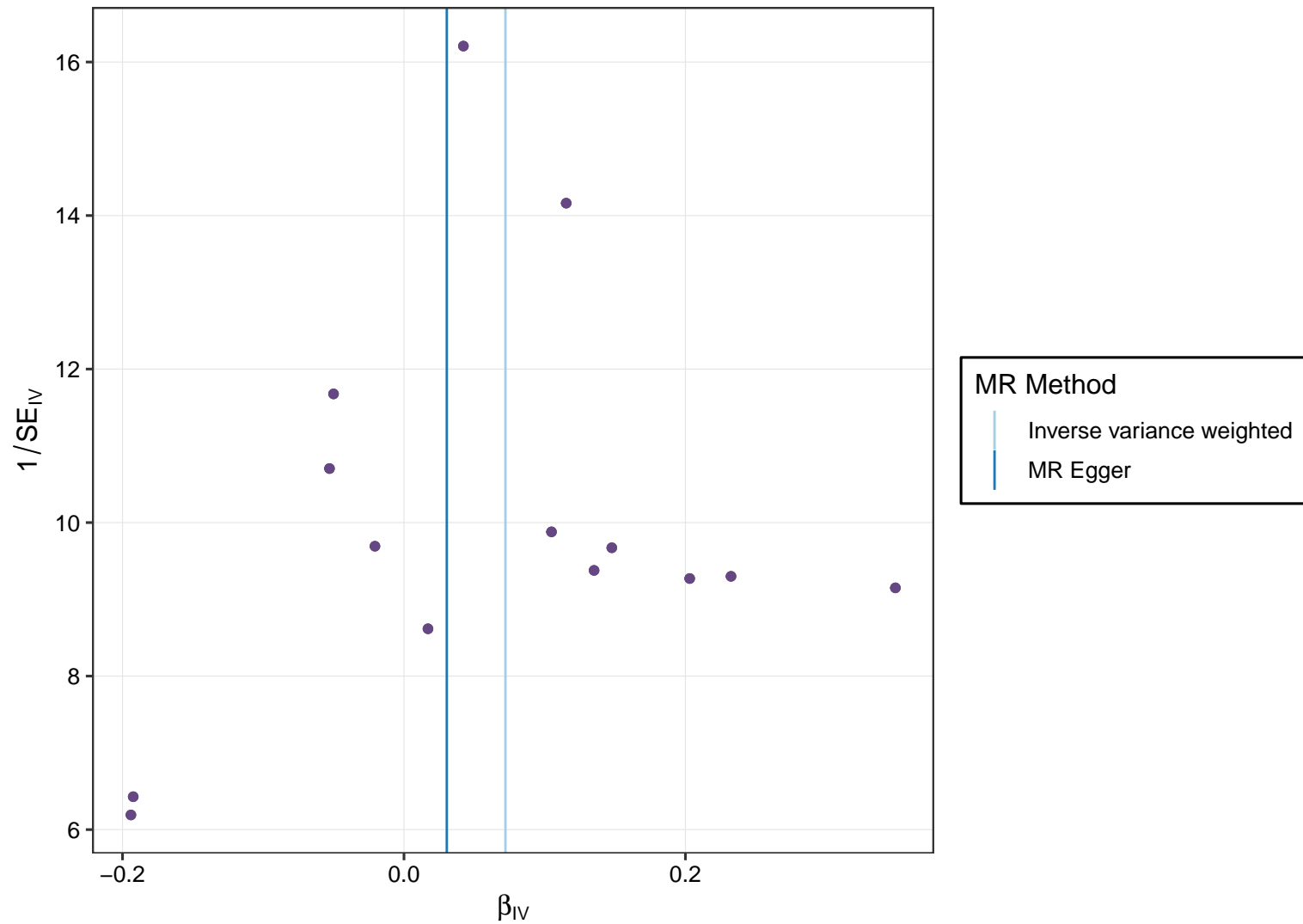

Supplement: Supplementary file 2 — Supplementary Material 2 [file 13568_2025_1969_MOESM2_ESM.zip › Revised supplementary materials/5 Forward MR analysis results/plot/funnel_or_UBA7703.pdf]

**Funnel Plot (OR): UBA8904**

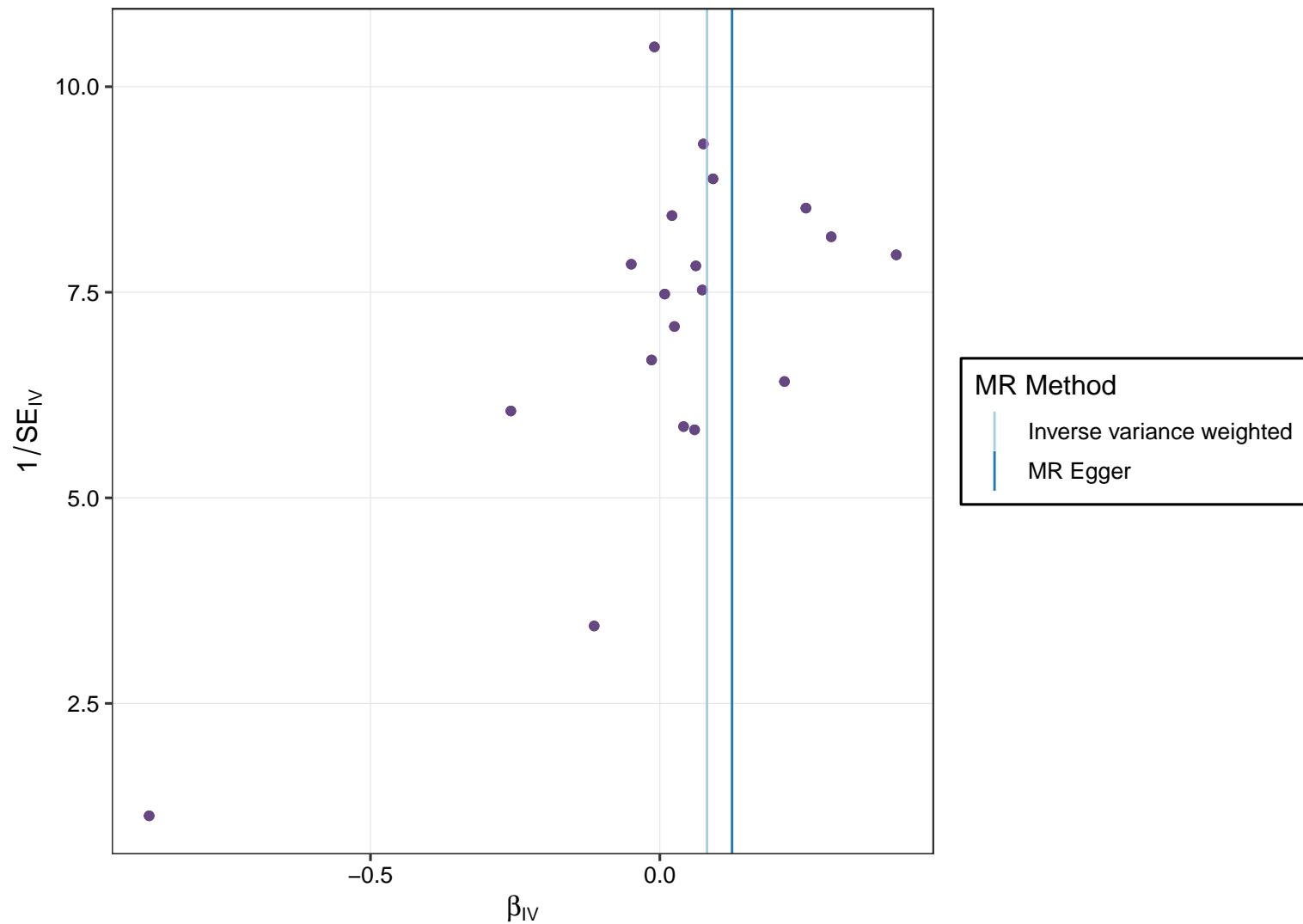

Supplement: Supplementary file 2 — Supplementary Material 2 [file 13568_2025_1969_MOESM2_ESM.zip › Revised supplementary materials/5 Forward MR analysis results/plot/funnel_or_UBA8904.pdf]

**Funnel Plot (OR): V9D3004**

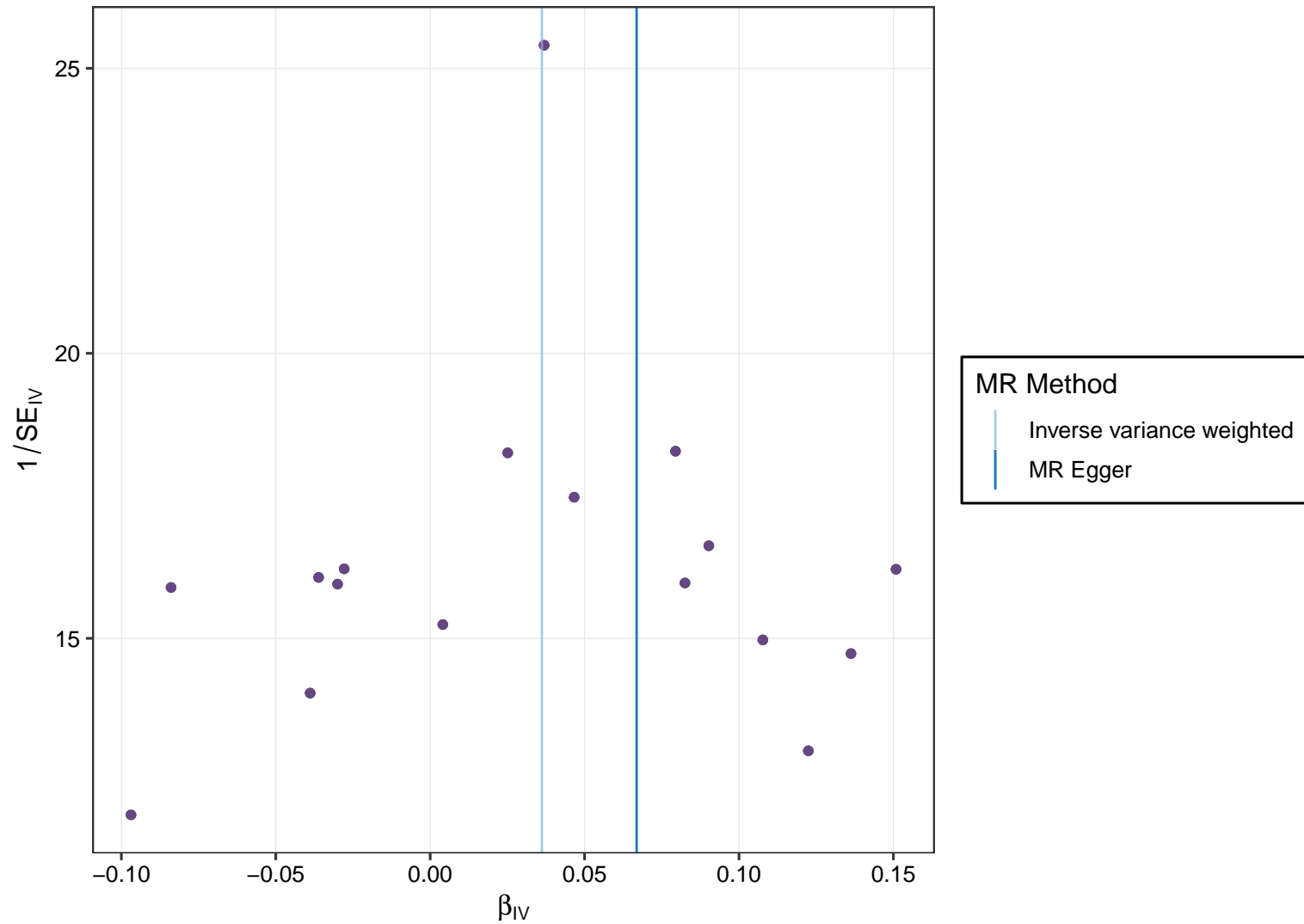

Supplement: Supplementary file 2 — Supplementary Material 2 [file 13568_2025_1969_MOESM2_ESM.zip › Revised supplementary materials/5 Forward MR analysis results/plot/funnel_or_V9D3004.pdf]

# Leave-One-Out (OR): *Acidaminococcus fermentans*

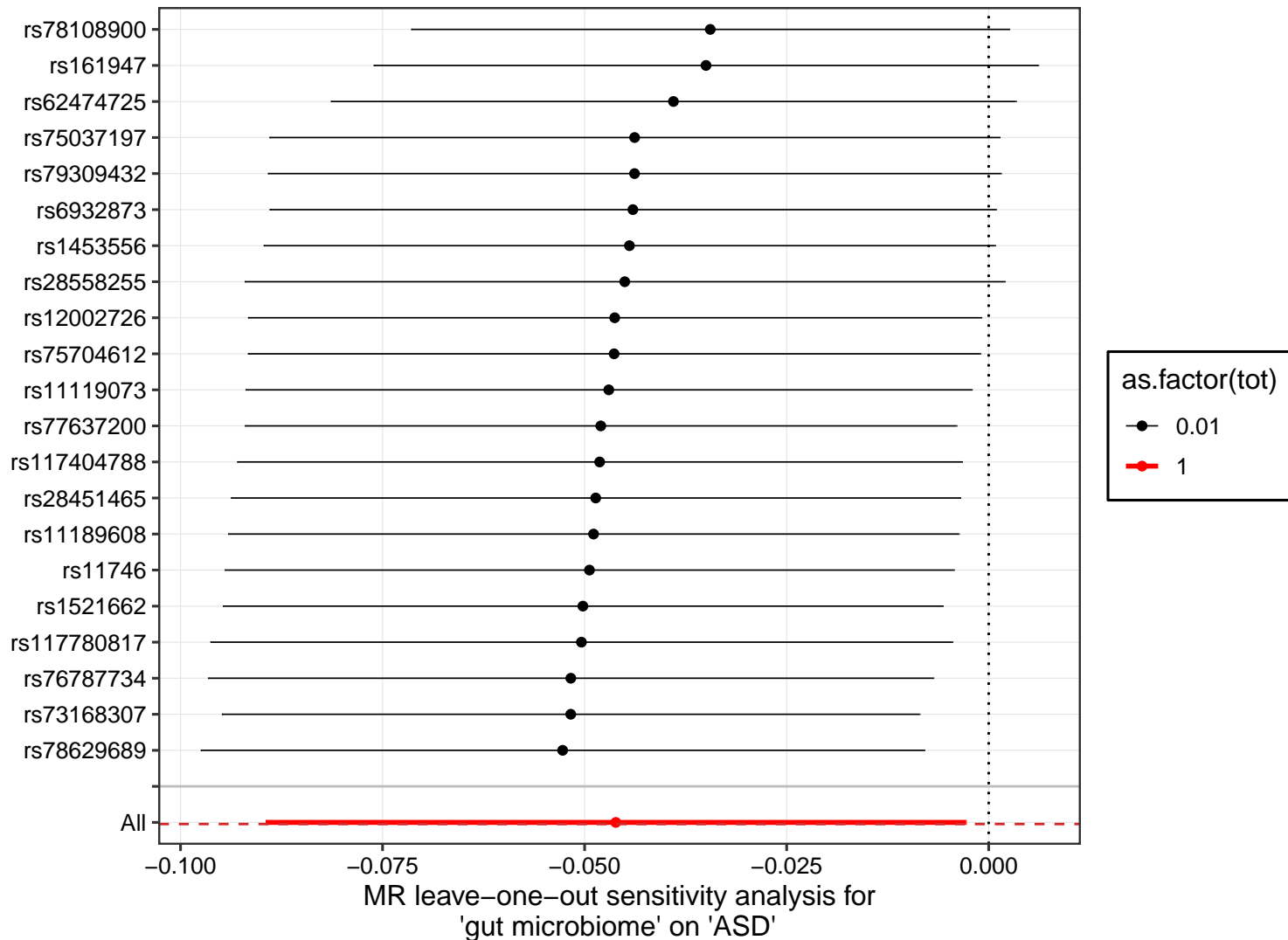

Supplement: Supplementary file 2 — Supplementary Material 2 [file 13568_2025_1969_MOESM2_ESM.zip › Revised supplementary materials/5 Forward MR analysis results/plot/leaveoneout_or_Acidaminococcus fermentans.pdf]

# Leave-One-Out (OR): CAG-475

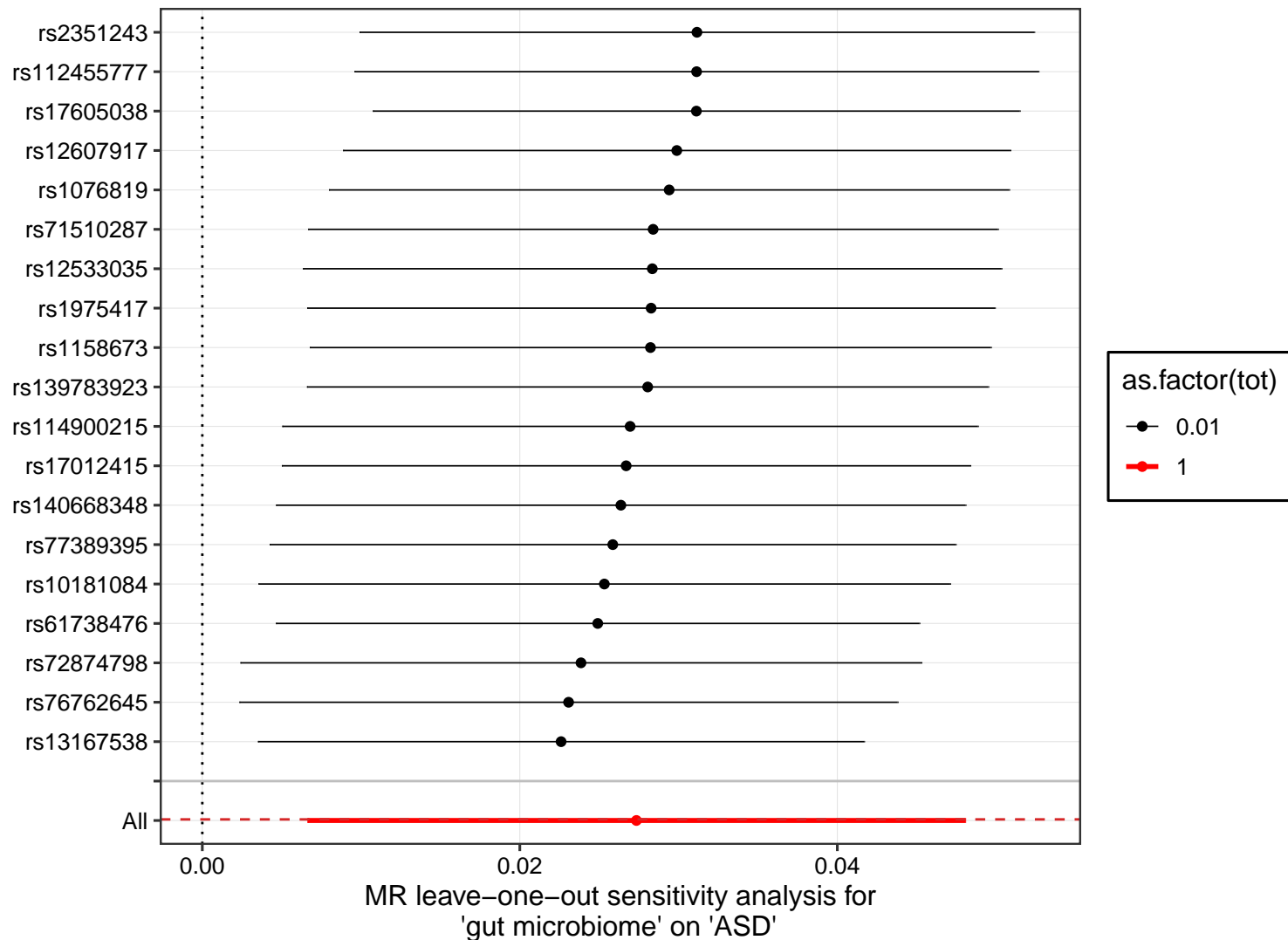

Supplement: Supplementary file 2 — Supplementary Material 2 [file 13568_2025_1969_MOESM2_ESM.zip › Revised supplementary materials/5 Forward MR analysis results/plot/leaveoneout_or_CAG-475.pdf]

# Leave-One-Out (OR): CAG-510 sp002432425

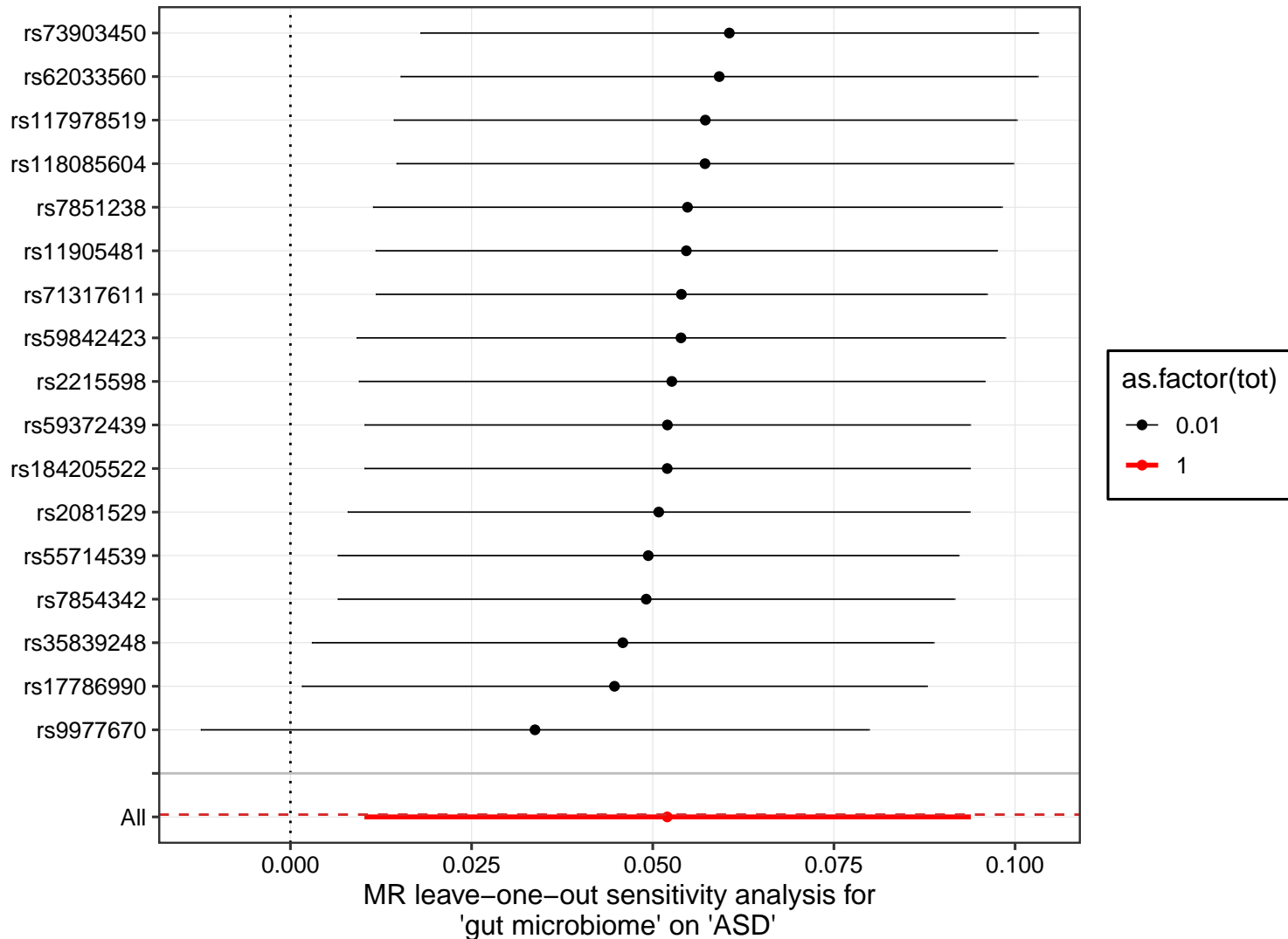

Supplement: Supplementary file 2 — Supplementary Material 2 [file 13568_2025_1969_MOESM2_ESM.zip › Revised supplementary materials/5 Forward MR analysis results/plot/leaveoneout_or_CAG-510 sp002432425.pdf]
