# Supplementary material for: Multi-omics reveals cross-tissue regulatory mechanisms of autism risk loci via gut microbiota-immunity-brain axis
Source: AMB Express. 2025 Oct 29;15:161. doi: 10.1186/s13568-025-01969-4 (PMC12572420; doi:10.1186/s13568-025-01969-4)
Supplement: Supplementary file 2 — Supplementary Material 2 [file 13568_2025_1969_MOESM2_ESM.zip › Revised supplementary materials/6 Inverse MR analysis results/plot/leaveoneout_or_Microvirga.pdf]

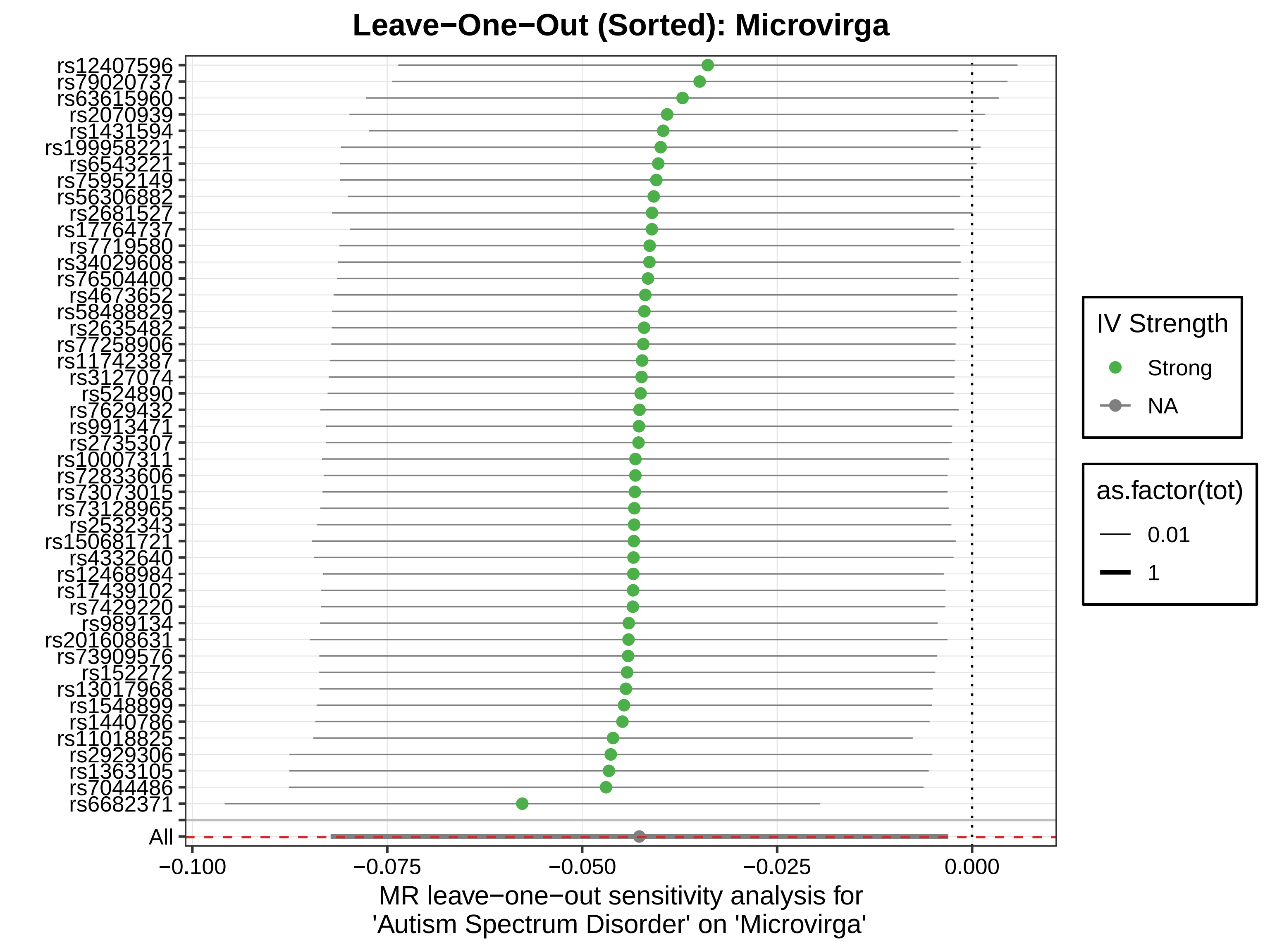
Leave−One−Out (Sorted): Microvirga
rs12407596
rs79020737
rs63615960
rs2070939
rs1431594
rs199958221
rs6543221
rs75952149
rs56306882
rs2681527
rs17764737
rs7719580
rs34029608
rs76504400
rs4673652
rs58488829
rs2635482
rs77258906
rs11742387
rs3127074
rs524890
rs7629432
rs9913471
rs2735307
rs10007311
rs72833606
rs73073015
rs73128965
rs2532343
rs150681721
rs4332640
rs12468984
rs17439102
rs7429220
rs989134
rs201608631
rs73909576
rs152272
rs13017968
rs1548899
rs1440786
rs11018825
rs2929306
rs1363105
rs7044486
rs6682371
All
−0.100
−0.075
−0.050
−0.025
0.000
MR leave−one−out sensitivity analysis for
'Autism Spectrum Disorder' on 'Microvirga'
IV Strength
Strong
NA
as.factor(tot)
0.01
1
