# Supplementary material for: Multi-omics reveals cross-tissue regulatory mechanisms of autism risk loci via gut microbiota-immunity-brain axis
Source: AMB Express. 2025 Oct 29;15:161. doi: 10.1186/s13568-025-01969-4 (PMC12572420; doi:10.1186/s13568-025-01969-4)
Supplement: Supplementary file 2 — Supplementary Material 2 [file 13568_2025_1969_MOESM2_ESM.zip › Revised supplementary materials/6 Inverse MR analysis results/plot/leaveoneout_or_Phascolarctobacterium sp003150755.pdf]

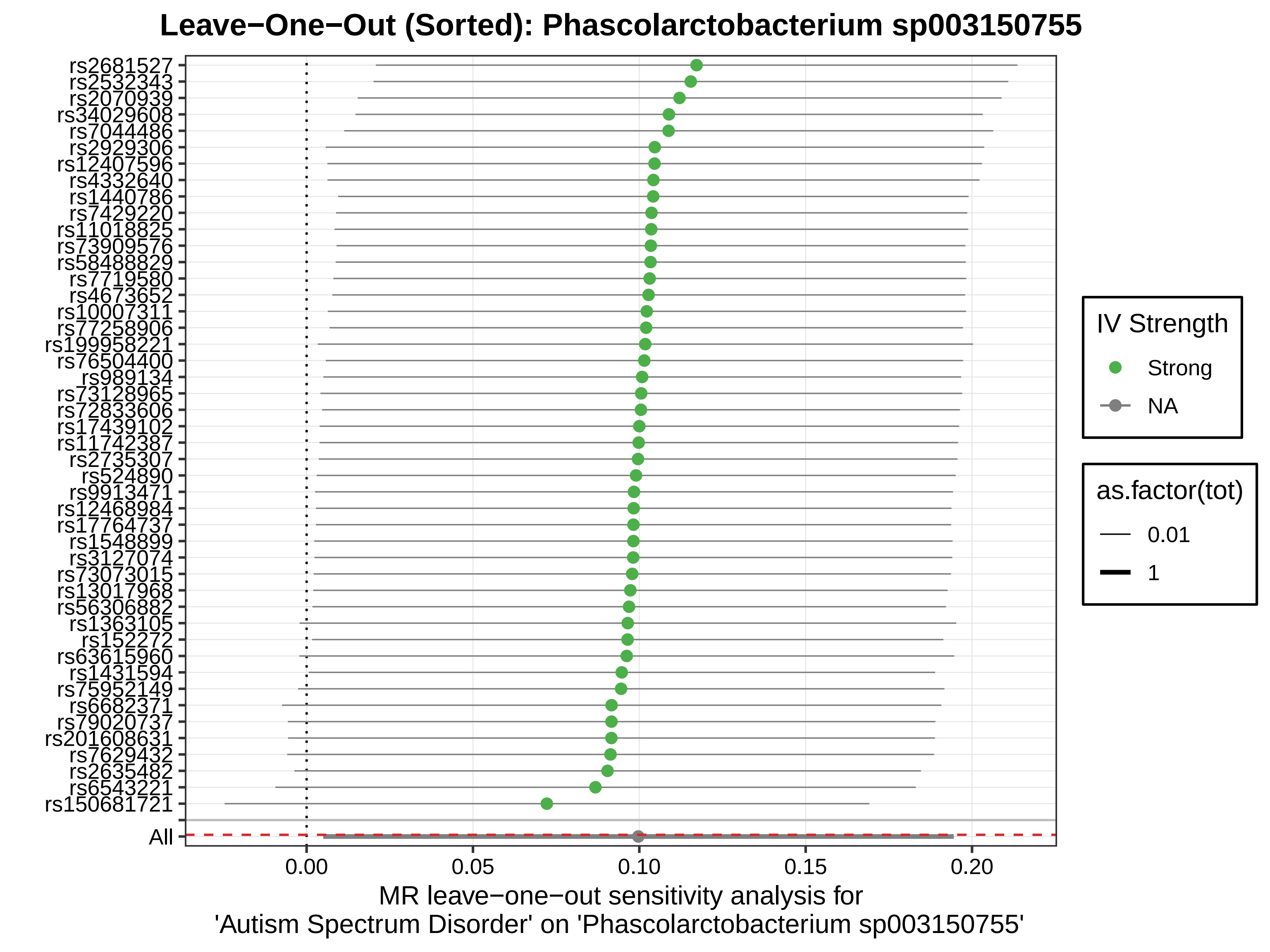
Leave−One−Out (Sorted): Phascolarctobacterium sp003150755
rs2681527
rs2532343
rs2070939
rs34029608
rs7044486
rs2929306
rs12407596
rs4332640
rs1440786
rs7429220
rs11018825
rs73909576
rs58488829
rs7719580
rs4673652
rs10007311
rs77258906
rs199958221
rs76504400
rs989134
rs73128965
rs72833606
rs17439102
rs11742387
rs2735307
rs524890
rs9913471
rs12468984
rs17764737
rs1548899
rs3127074
rs73073015
rs13017968
rs56306882
rs1363105
rs152272
rs63615960
rs1431594
rs75952149
rs6682371
rs79020737
rs201608631
rs7629432
rs2635482
rs6543221
rs150681721
All
0.00
0.05
0.10
0.15
0.20
MR leave−one−out sensitivity analysis for
'Autism Spectrum Disorder' on 'Phascolarctobacterium sp003150755'
IV Strength
Strong
NA
as.factor(tot)
0.01
1
