# Supplementary material for: Multi-omics reveals cross-tissue regulatory mechanisms of autism risk loci via gut microbiota-immunity-brain axis
Source: AMB Express. 2025 Oct 29;15:161. doi: 10.1186/s13568-025-01969-4 (PMC12572420; doi:10.1186/s13568-025-01969-4)
Supplement: Supplementary file 2 — Supplementary Material 2 [file 13568_2025_1969_MOESM2_ESM.zip › Revised supplementary materials/6 Inverse MR analysis results/plot/leaveoneout_or_RUG472.pdf]

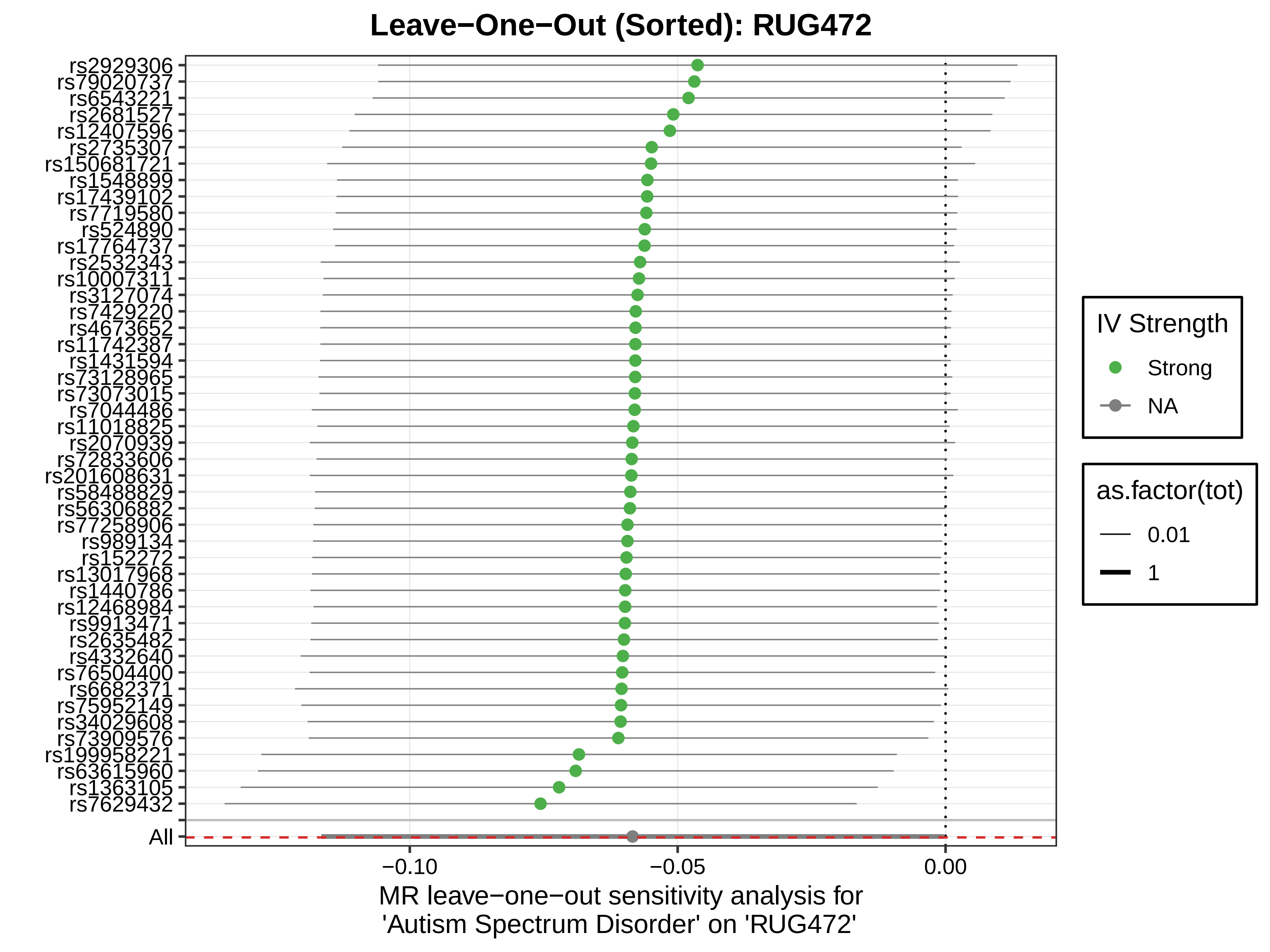

Leave−One−Out (Sorted): RUG472
rs2929306
rs79020737
rs6543221
rs2681527
rs12407596
rs2735307
rs150681721
rs1548899
rs17439102
rs7719580
rs524890
rs17764737
rs2532343
rs10007311
rs3127074
rs7429220
rs4673652
rs11742387
rs1431594
rs73128965
rs73073015
rs7044486
rs11018825
rs2070939
rs72833606
rs201608631
rs58488829
rs56306882
rs77258906
rs989134
rs152272
rs13017968
rs1440786
rs12468984
rs9913471
rs2635482
rs4332640
rs76504400
rs6682371
rs75952149
rs34029608
rs73909576
rs199958221
rs63615960
rs1363105
rs7629432
All
−0.10
−0.05
0.00
MR leave−one−out sensitivity analysis for
'Autism Spectrum Disorder' on 'RUG472'
IV Strength
Strong
NA
as.factor(tot)
0.01
1
