# Supplementary material for: Multi-omics reveals cross-tissue regulatory mechanisms of autism risk loci via gut microbiota-immunity-brain axis
Source: AMB Express. 2025 Oct 29;15:161. doi: 10.1186/s13568-025-01969-4 (PMC12572420; doi:10.1186/s13568-025-01969-4)
Supplement: Supplementary file 2 — Supplementary Material 2 [file 13568_2025_1969_MOESM2_ESM.zip › Revised supplementary materials/6 Inverse MR analysis results/plot/leaveoneout_or_Spirillospora.pdf]

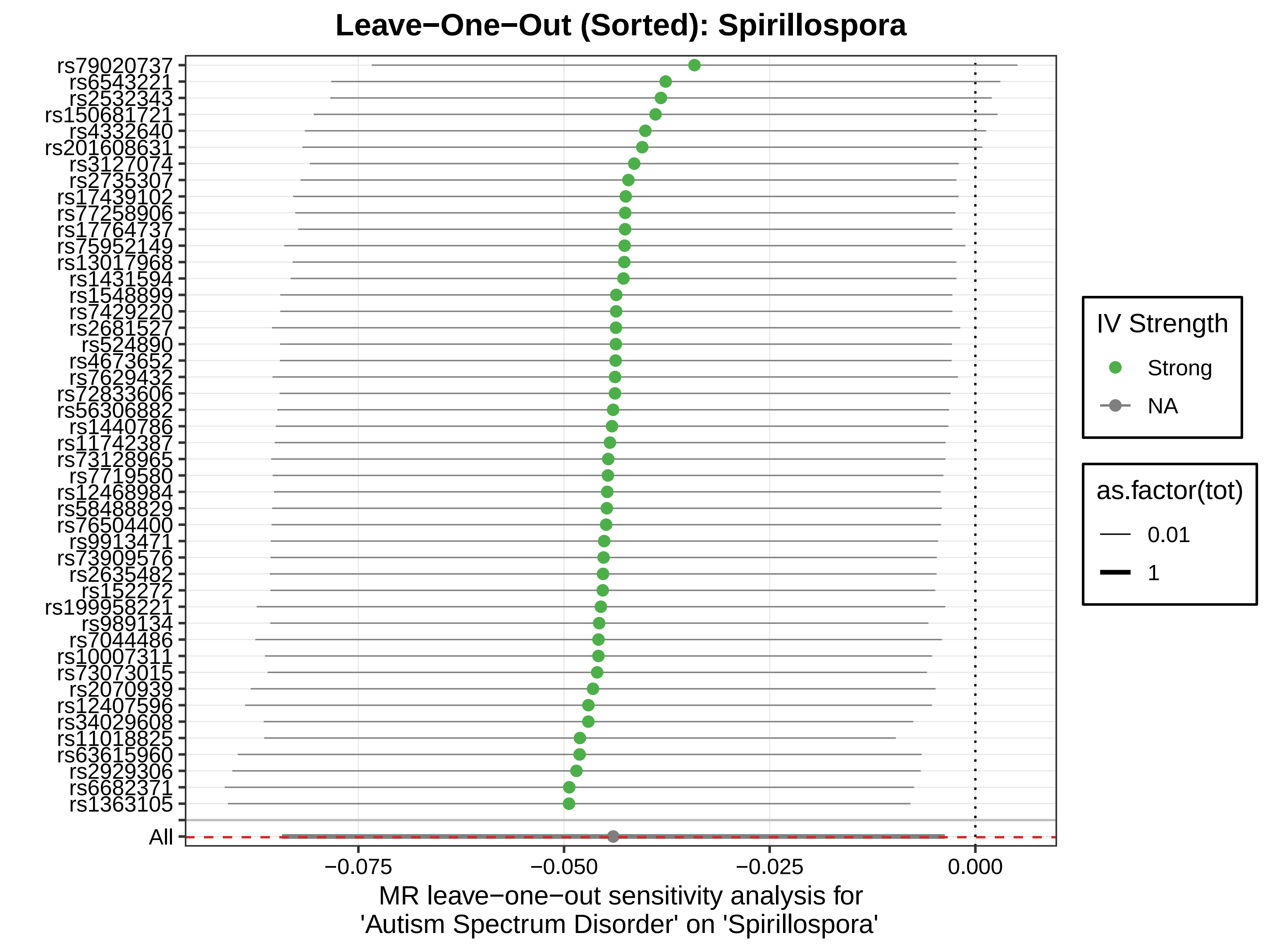
Leave−One−Out (Sorted): Spirillospora
rs79020737
rs6543221
rs2532343
rs150681721
rs4332640
rs201608631
rs3127074
rs2735307
rs17439102
rs77258906
rs17764737
rs75952149
rs13017968
rs1431594
rs1548899
rs7429220
rs2681527
rs524890
rs4673652
rs7629432
rs72833606
rs56306882
rs1440786
rs11742387
rs73128965
rs7719580
rs12468984
rs58488829
rs76504400
rs9913471
rs73909576
rs2635482
rs152272
rs199958221
rs989134
rs7044486
rs10007311
rs73073015
rs2070939
rs12407596
rs34029608
rs11018825
rs63615960
rs2929306
rs6682371
rs1363105
All
−0.075
−0.050
−0.025
0.000
MR leave−one−out sensitivity analysis for
'Autism Spectrum Disorder' on 'Spirillospora'
IV Strength
Strong
NA
as.factor(tot)
0.01
1
