# Supplementary material for: Multi-omics reveals cross-tissue regulatory mechanisms of autism risk loci via gut microbiota-immunity-brain axis
Source: AMB Express. 2025 Oct 29;15:161. doi: 10.1186/s13568-025-01969-4 (PMC12572420; doi:10.1186/s13568-025-01969-4)
Supplement: Supplementary file 2 — Supplementary Material 2 [file 13568_2025_1969_MOESM2_ESM.zip › Revised supplementary materials/6 Inverse MR analysis results/plot/leaveoneout_or_UBA1777 sp900316255.pdf]

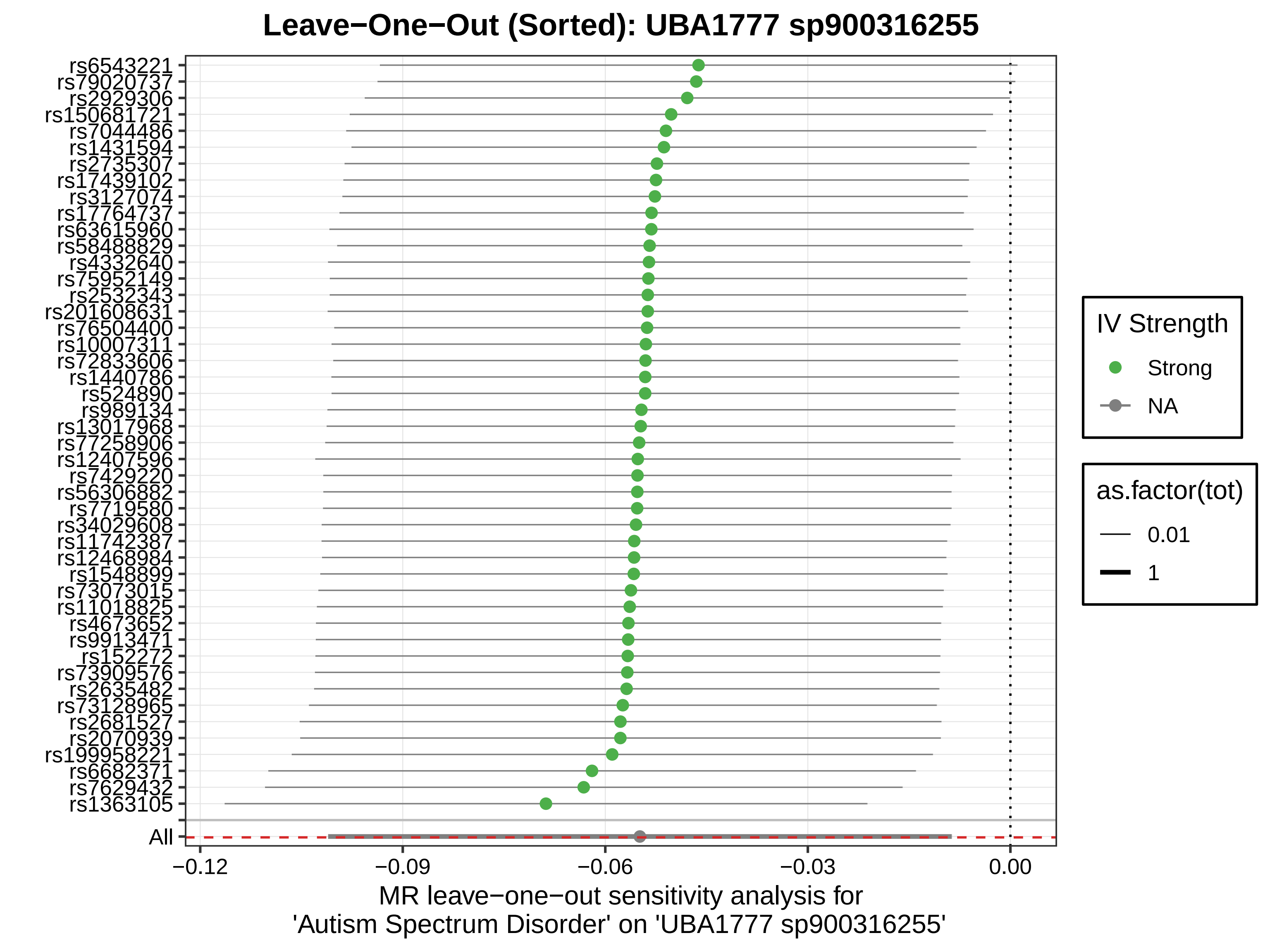

Leave−One−Out (Sorted): UBA1777 sp900316255
rs6543221
rs79020737
rs2929306
rs150681721
rs7044486
rs1431594
rs2735307
rs17439102
rs3127074
rs17764737
rs63615960
rs58488829
rs4332640
rs75952149
rs2532343
rs201608631
rs76504400
rs10007311
rs72833606
rs1440786
rs524890
rs989134
rs13017968
rs77258906
rs12407596
rs7429220
rs56306882
rs7719580
rs34029608
rs11742387
rs12468984
rs1548899
rs73073015
rs11018825
rs4673652
rs9913471
rs152272
rs73909576
rs2635482
rs73128965
rs2681527
rs2070939
rs199958221
rs6682371
rs7629432
rs1363105
All
−0.12
−0.09
−0.06
−0.03
0.00
MR leave−one−out sensitivity analysis for
'Autism Spectrum Disorder' on 'UBA1777 sp900316255'
IV Strength
Strong
NA
as.factor(tot)
0.01
1
