# Supplementary material for: Multi-omics reveals cross-tissue regulatory mechanisms of autism risk loci via gut microbiota-immunity-brain axis
Source: AMB Express. 2025 Oct 29;15:161. doi: 10.1186/s13568-025-01969-4 (PMC12572420; doi:10.1186/s13568-025-01969-4)
Supplement: Supplementary file 2 — Supplementary Material 2 [file 13568_2025_1969_MOESM2_ESM.zip › Revised supplementary materials/6 Inverse MR analysis results/plot/leaveoneout_or_UBA7703.pdf]

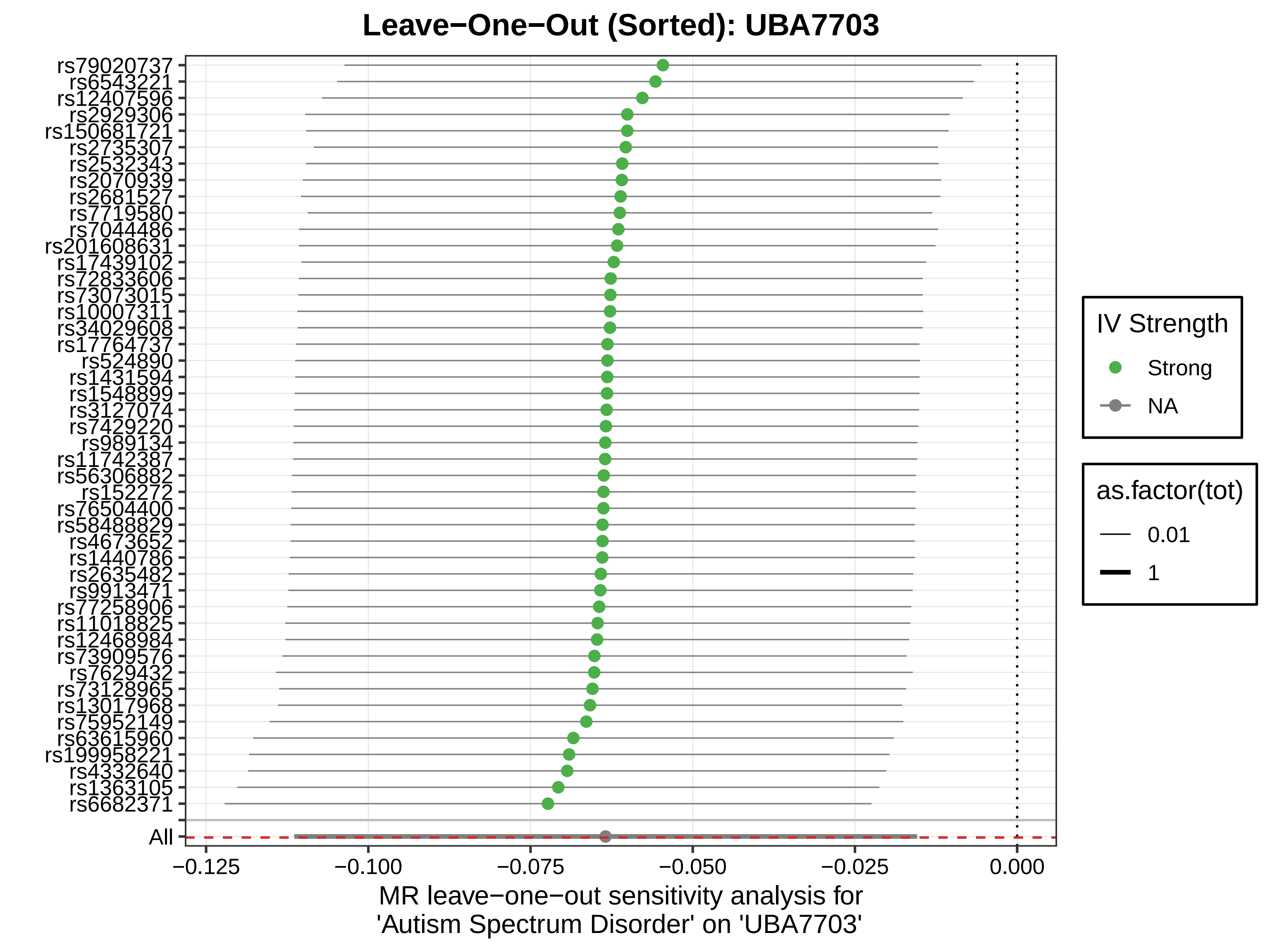

Leave−One−Out (Sorted): UBA7703
rs79020737
rs6543221
rs12407596
rs2929306
rs150681721
rs2735307
rs2532343
rs2070939
rs2681527
rs7719580
rs7044486
rs201608631
rs17439102
rs72833606
rs73073015
rs10007311
rs34029608
rs17764737
rs524890
rs1431594
rs1548899
rs3127074
rs7429220
rs989134
rs11742387
rs56306882
rs152272
rs76504400
rs58488829
rs4673652
rs1440786
rs2635482
rs9913471
rs77258906
rs11018825
rs12468984
rs73909576
rs7629432
rs73128965
rs13017968
rs75952149
rs63615960
rs199958221
rs4332640
rs1363105
rs6682371
All
−0.125
−0.100
−0.075
−0.050
−0.025
0.000
MR leave−one−out sensitivity analysis for
'Autism Spectrum Disorder' on 'UBA7703'
IV Strength
Strong
NA
as.factor(tot)
0.01
1
