# Supplementary material for: Multi-omics reveals cross-tissue regulatory mechanisms of autism risk loci via gut microbiota-immunity-brain axis
Source: AMB Express. 2025 Oct 29;15:161. doi: 10.1186/s13568-025-01969-4 (PMC12572420; doi:10.1186/s13568-025-01969-4)
Supplement: Supplementary file 2 — Supplementary Material 2 [file 13568_2025_1969_MOESM2_ESM.zip › Revised supplementary materials/6 Inverse MR analysis results/plot/legends.docx]

Leave-one-out plot: Sensitivity analysis plot showing the trend in Mendelian randomisation effects after removing individual SNPs based on the gut microbiota abundance instrumental variable set. y-axis: names of SNPs removed one by one; x-axis: effect estimates of exposure → outcome after removing the SNP; red dashed line: MR effect of the entire sample (without removing any SNPs); black dots (as.factor(0.01)), red squares (as.factor(1)): effect points marked with different plotting parameters.

Scatter plot: Plot showing the distribution of SNP effects on gut microbiota abundance and ASD, and the causal effect fitting trends of five MR methods: different colours represent different methods.

Funnel plot: Plot comparing inverse variance-weighted MR methods, showing the distribution of effect estimates (β₍_VW_₎, x-axis) and effect precision (1/SE, y-axis; where 1/SE indicates higher precision of effect estimates), to illustrate the distribution characteristics of instrumental variable effects.

Forest plot: Plot integrating the 'individual SNP independent effects' and 'aggregated effects of multiple SNPs' to quantitatively present causal associations. y-axis: names of SNPs included in the analysis one by one; x-axis: MR effect values; black scatter points + horizontal line segments: effect estimates and their 95% confidence intervals when a single SNP is used as an instrumental variable; bottom red horizontal line (All - Inverse variance weighted, All - MR Egger): combined effects of multiple instrumental variables after integrating all SNPs.
