# Supplementary material for: Multi-omics reveals cross-tissue regulatory mechanisms of autism risk loci via gut microbiota-immunity-brain axis
Source: AMB Express. 2025 Oct 29;15:161. doi: 10.1186/s13568-025-01969-4 (PMC12572420; doi:10.1186/s13568-025-01969-4)
Supplement: Supplementary file 2 — Supplementary Material 2 [file 13568_2025_1969_MOESM2_ESM.zip › Revised supplementary materials/6 Inverse MR analysis results/plot/scatter_or_Aureimonas.pdf]

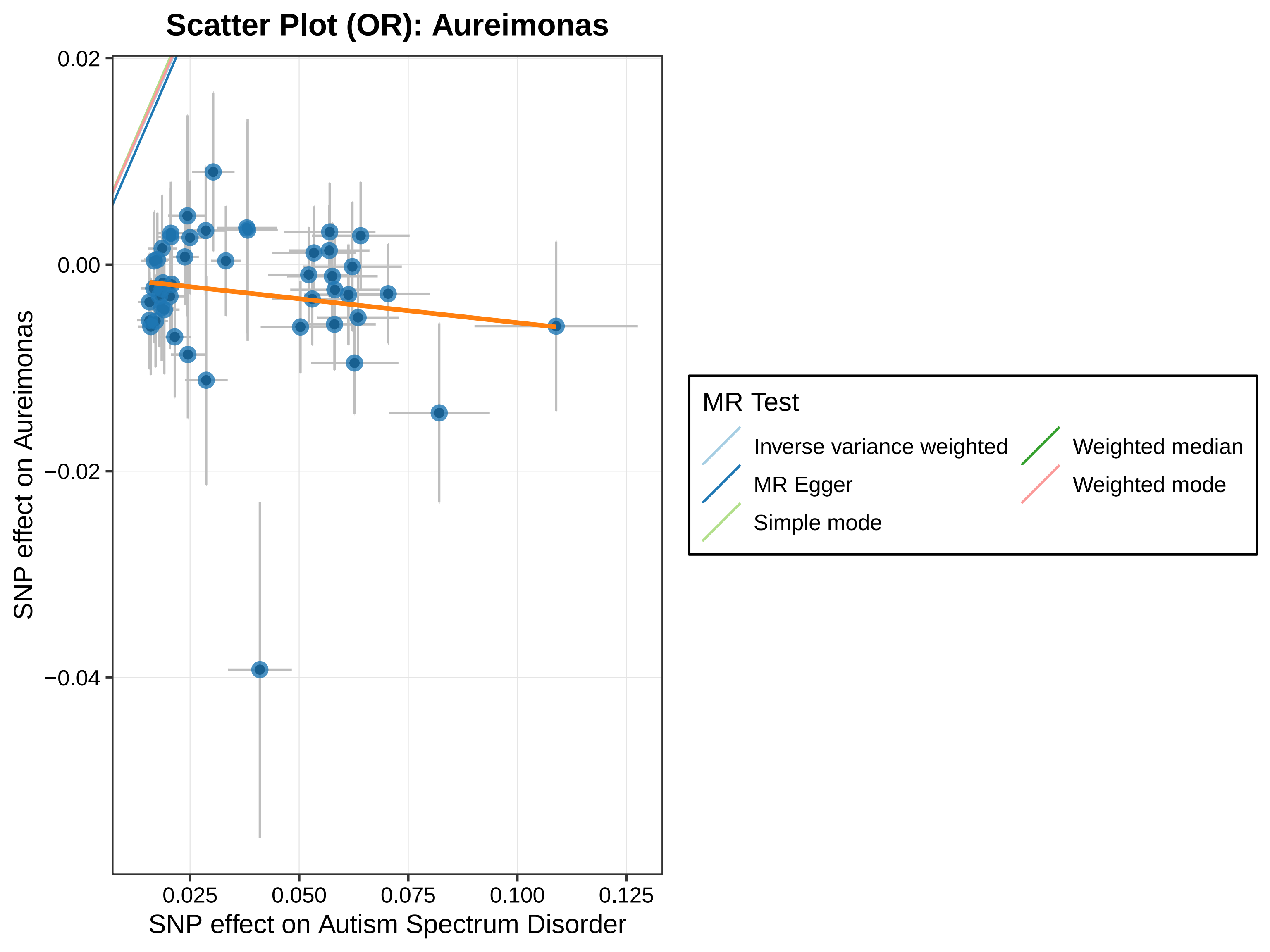

Scatter Plot (OR): Aureimonas
0.02
0.00
−0.02
−0.04
0.025
0.050
0.075
0.100
0.125
SNP effect on Aureimonas
SNP effect on Autism Spectrum Disorder
MR Test
Inverse variance weighted
MR Egger
Simple mode
Weighted median
Weighted mode
