# Supplementary material for: Multi-omics reveals cross-tissue regulatory mechanisms of autism risk loci via gut microbiota-immunity-brain axis
Source: AMB Express. 2025 Oct 29;15:161. doi: 10.1186/s13568-025-01969-4 (PMC12572420; doi:10.1186/s13568-025-01969-4)
Supplement: Supplementary file 2 — Supplementary Material 2 [file 13568_2025_1969_MOESM2_ESM.zip › Revised supplementary materials/7 Common SNP SMR results Plot/ENSG00000091127_locusplot.pdf]

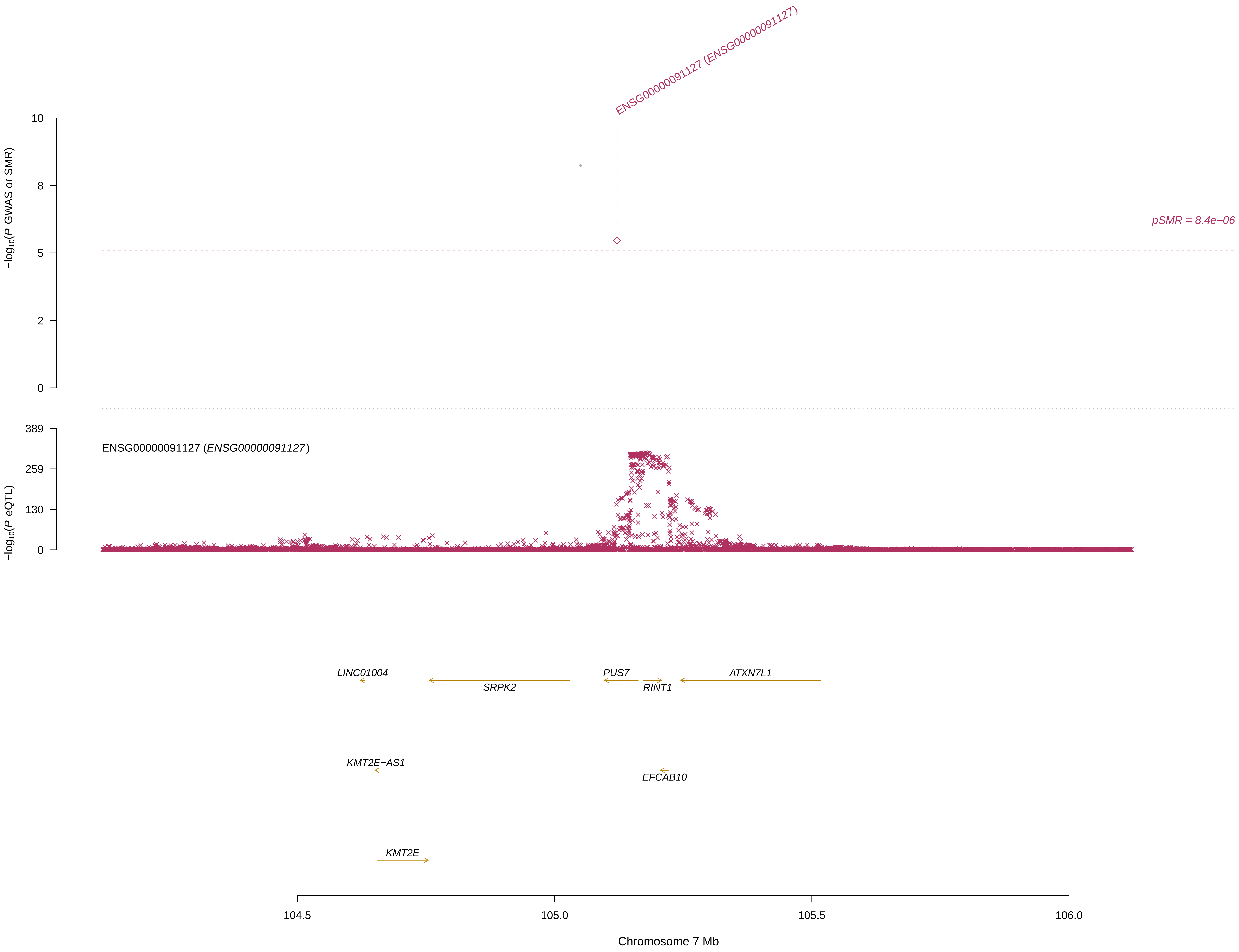
ENSG00000091127 (ENSG00000091127)
pSMR = 8.4e−06
−log10(P GWAS or SMR)
10
8
5
2
0
389
259
130
0
ENSG00000091127 (ENSG00000091127)
−log10(P eQTL)
LINC01004
SRPK2
PUS7
RINT1
ATXN7L1
KMT2E−AS1
EFCAB10
KMT2E
104.5
105.0
105.5
106.0
Chromosome 7 Mb
