# Supplementary material for: Multi-omics reveals cross-tissue regulatory mechanisms of autism risk loci via gut microbiota-immunity-brain axis
Source: AMB Express. 2025 Oct 29;15:161. doi: 10.1186/s13568-025-01969-4 (PMC12572420; doi:10.1186/s13568-025-01969-4)
Supplement: Supplementary file 2 — Supplementary Material 2 [file 13568_2025_1969_MOESM2_ESM.zip › Revised supplementary materials/7 Common SNP SMR results Plot/ENSG00000124508_locusplot.pdf]

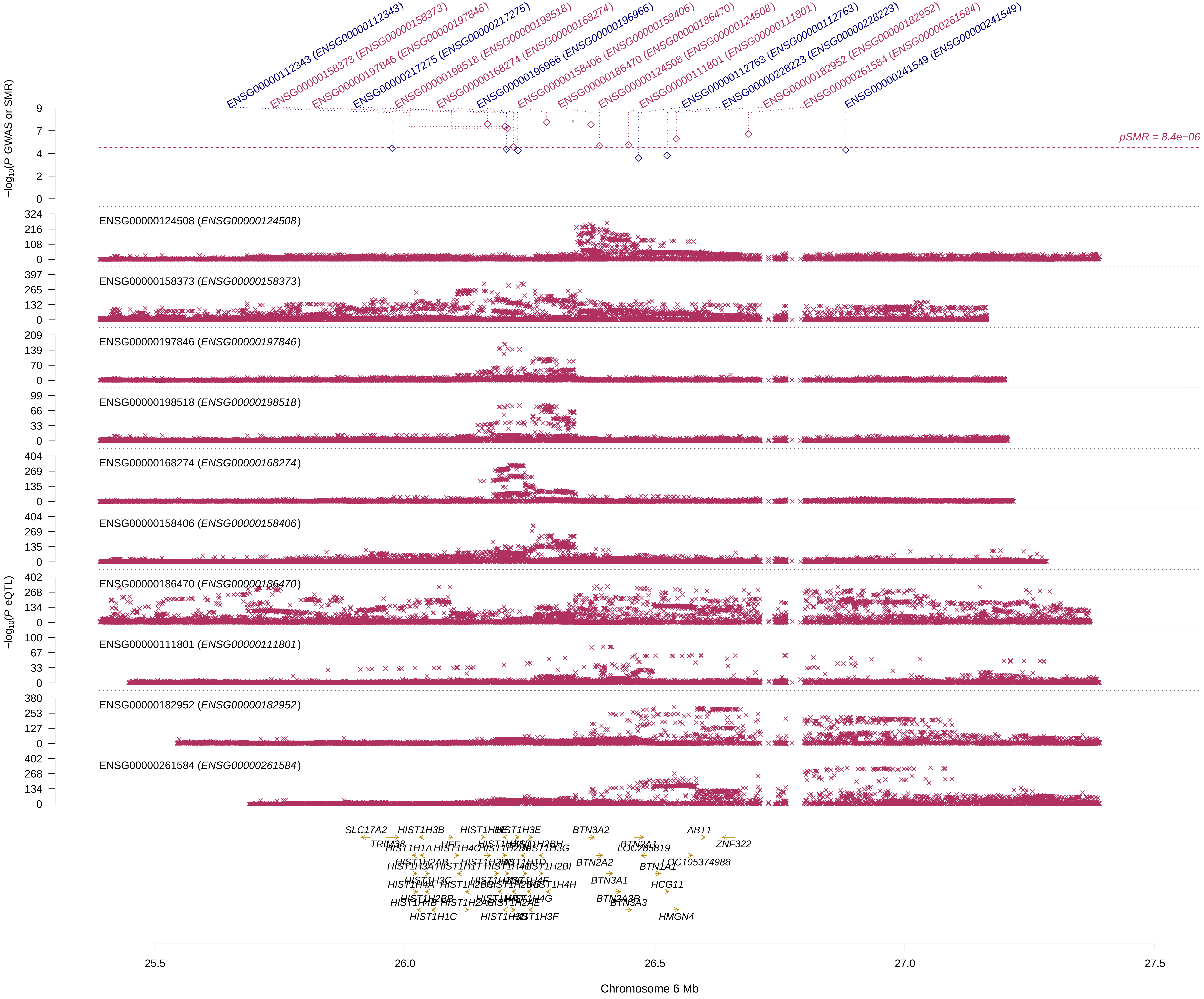

ENSG00000112343 (ENSG00000112343)
ENSG00000158373 (ENSG00000158373)
ENSG00000197846 (ENSG00000197846)
ENSG00000217275 (ENSG00000217275)
ENSG00000198518 (ENSG00000198518)
ENSG00000168274 (ENSG00000168274)
ENSG00000196966 (ENSG00000196966)
ENSG00000158406 (ENSG00000158406)
ENSG00000186470 (ENSG00000186470)
ENSG00000124508 (ENSG00000124508)
ENSG00000111801 (ENSG00000111801)
ENSG00000112763 (ENSG00000112763)
ENSG00000228223 (ENSG00000228223)
ENSG00000182952 (ENSG00000182952)
ENSG00000261584 (ENSG00000261584)
ENSG00000241549 (ENSG00000241549)
−log10(P GWAS or SMR)
pSMR = 8.4e−06
ENSG00000124508 (ENSG00000124508)
ENSG00000158373 (ENSG00000158373)
ENSG00000197846 (ENSG00000197846)
ENSG00000198518 (ENSG00000198518)
ENSG00000168274 (ENSG00000168274)
ENSG00000158406 (ENSG00000158406)
ENSG00000186470 (ENSG00000186470)
ENSG00000111801 (ENSG00000111801)
ENSG00000182952 (ENSG00000182952)
ENSG00000261584 (ENSG00000261584)
−log10(P eQTL)
SLC17A2
TRIM38
HIST1H3B
HFE
BTN3A2
BTN2A1
ABT1
ZNF322
LOC285819
BTN2A2
BTN1A1
LOC105374988
BTN3A1
HCG11
BTN2A3P
BTN3A3
HMGN4
HIST1H1C
HIST1H3F
25.5
26.0
26.5
27.0
27.5
Chromosome 6 Mb
