# Supplementary material for: Multi-omics reveals cross-tissue regulatory mechanisms of autism risk loci via gut microbiota-immunity-brain axis
Source: AMB Express. 2025 Oct 29;15:161. doi: 10.1186/s13568-025-01969-4 (PMC12572420; doi:10.1186/s13568-025-01969-4)
Supplement: Supplementary file 2 — Supplementary Material 2 [file 13568_2025_1969_MOESM2_ESM.zip › Revised supplementary materials/7 Common SNP SMR results Plot/ENSG00000157578_locusplot.pdf]

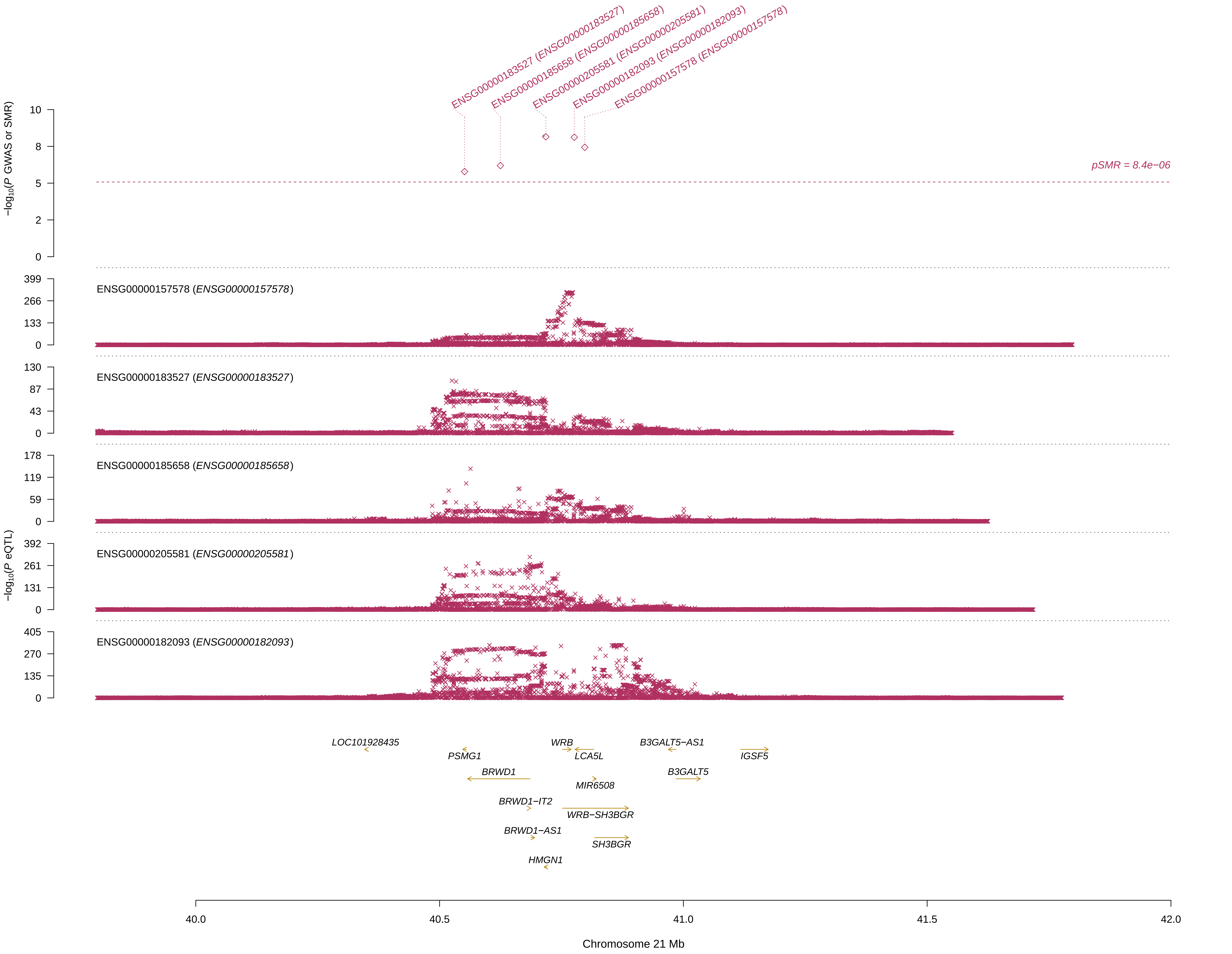
ENSG00000183527 (ENSG00000183527)
ENSG00000185658 (ENSG00000185658)
ENSG00000205581 (ENSG00000205581)
ENSG00000182093 (ENSG00000182093)
ENSG00000157578 (ENSG00000157578)
−log10(P GWAS or SMR)
10
8
5
2
0
pSMR = 8.4e−06
ENSG00000157578 (ENSG00000157578)
399
266
133
0
ENSG00000183527 (ENSG00000183527)
130
87
43
0
ENSG00000185658 (ENSG00000185658)
178
119
59
0
−log10(P eQTL)
ENSG00000205581 (ENSG00000205581)
392
261
131
0
ENSG00000182093 (ENSG00000182093)
405
270
135
0
LOC101928435
PSMG1
BRWD1
BRWD1−IT2
BRWD1−AS1
HMGN1
WRB
LCA5L
MIR6508
WRB−SH3BGR
SH3BGR
B3GALT5−AS1
B3GALT5
IGSF5
40.0
40.5
41.0
41.5
42.0
Chromosome 21 Mb
