# Supplementary material for: Multi-omics reveals cross-tissue regulatory mechanisms of autism risk loci via gut microbiota-immunity-brain axis
Source: AMB Express. 2025 Oct 29;15:161. doi: 10.1186/s13568-025-01969-4 (PMC12572420; doi:10.1186/s13568-025-01969-4)
Supplement: Supplementary file 2 — Supplementary Material 2 [file 13568_2025_1969_MOESM2_ESM.zip › Revised supplementary materials/7 Common SNP SMR results Plot/ENSG00000158406_locusplot.pdf]

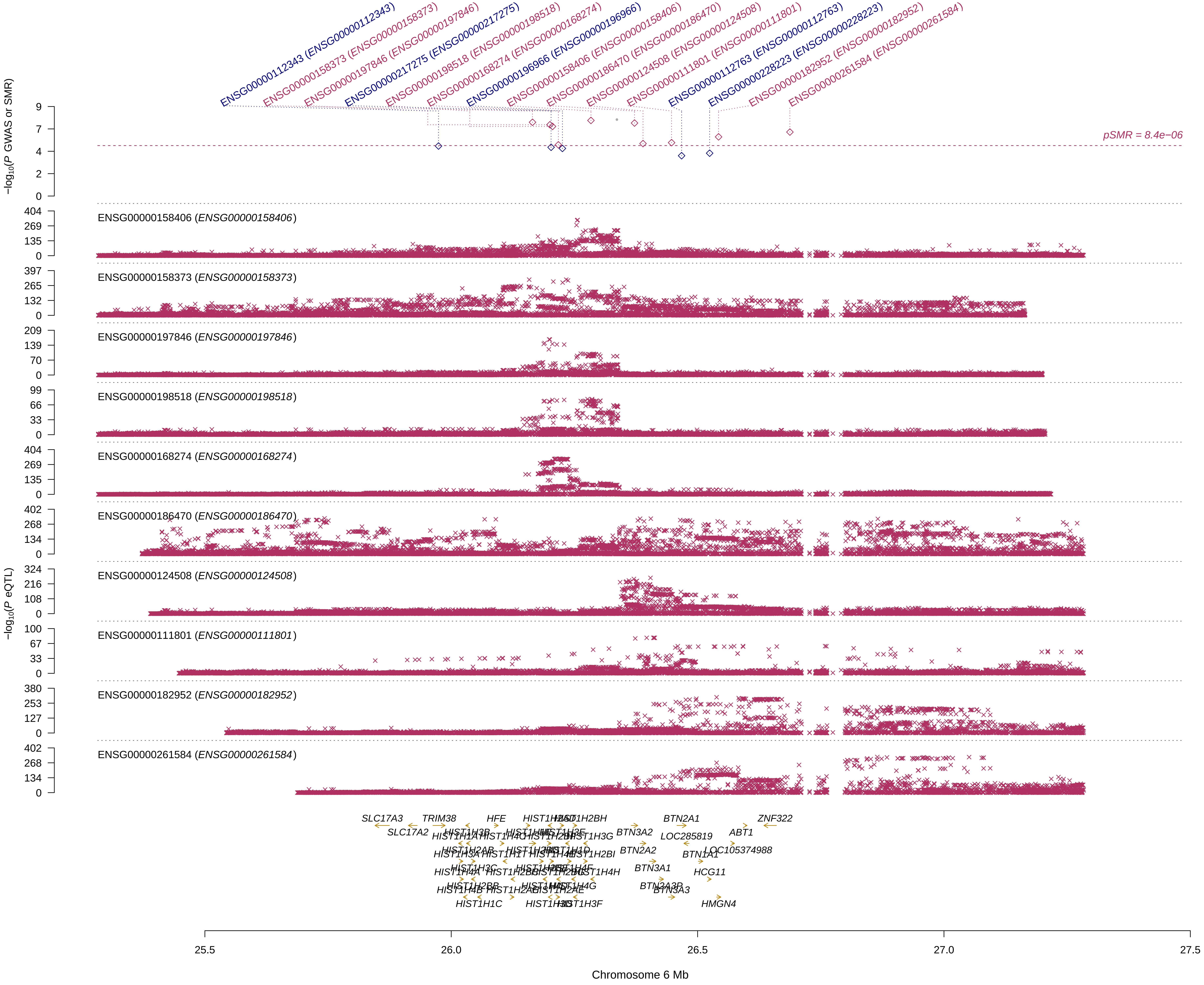

ENSG00000112343 (ENSG00000112343)
ENSG00000158373 (ENSG00000158373)
ENSG00000197846 (ENSG00000197846)
ENSG00000217275 (ENSG00000217275)
ENSG00000198518 (ENSG00000198518)
ENSG00000168274 (ENSG00000168274)
ENSG00000196966 (ENSG00000196966)
ENSG00000158406 (ENSG00000158406)
ENSG00000186470 (ENSG00000186470)
ENSG00000124508 (ENSG00000124508)
ENSG00000111801 (ENSG00000111801)
ENSG00000112763 (ENSG00000112763)
ENSG00000228223 (ENSG00000228223)
ENSG00000182952 (ENSG00000182952)
ENSG00000261584 (ENSG00000261584)
−log10(P GWAS or SMR)
pSMR = 8.4e−06
ENSG00000158406 (ENSG00000158406)
ENSG00000158373 (ENSG00000158373)
ENSG00000197846 (ENSG00000197846)
ENSG00000198518 (ENSG00000198518)
ENSG00000168274 (ENSG00000168274)
ENSG00000186470 (ENSG00000186470)
ENSG00000124508 (ENSG00000124508)
ENSG00000111801 (ENSG00000111801)
ENSG00000182952 (ENSG00000182952)
ENSG00000261584 (ENSG00000261584)
−log10(P eQTL)
SLC17A3
TRIM38
HFE
BTN2A1
ZNF322
SLC17A2
BTN3A2
LOC285819
ABT1
BTN2A2
LOC105374988
BTN1A1
BTN3A1
HCG11
HIST1H4H
HMGN4
HIST1H1C
HIST1H3F
Chromosome 6 Mb
25.5
26.0
26.5
27.0
27.5
