# Supplementary material for: Multi-omics reveals cross-tissue regulatory mechanisms of autism risk loci via gut microbiota-immunity-brain axis
Source: AMB Express. 2025 Oct 29;15:161. doi: 10.1186/s13568-025-01969-4 (PMC12572420; doi:10.1186/s13568-025-01969-4)
Supplement: Supplementary file 2 — Supplementary Material 2 [file 13568_2025_1969_MOESM2_ESM.zip › Revised supplementary materials/7 Common SNP SMR results Plot/ENSG00000182093_locusplot.pdf]

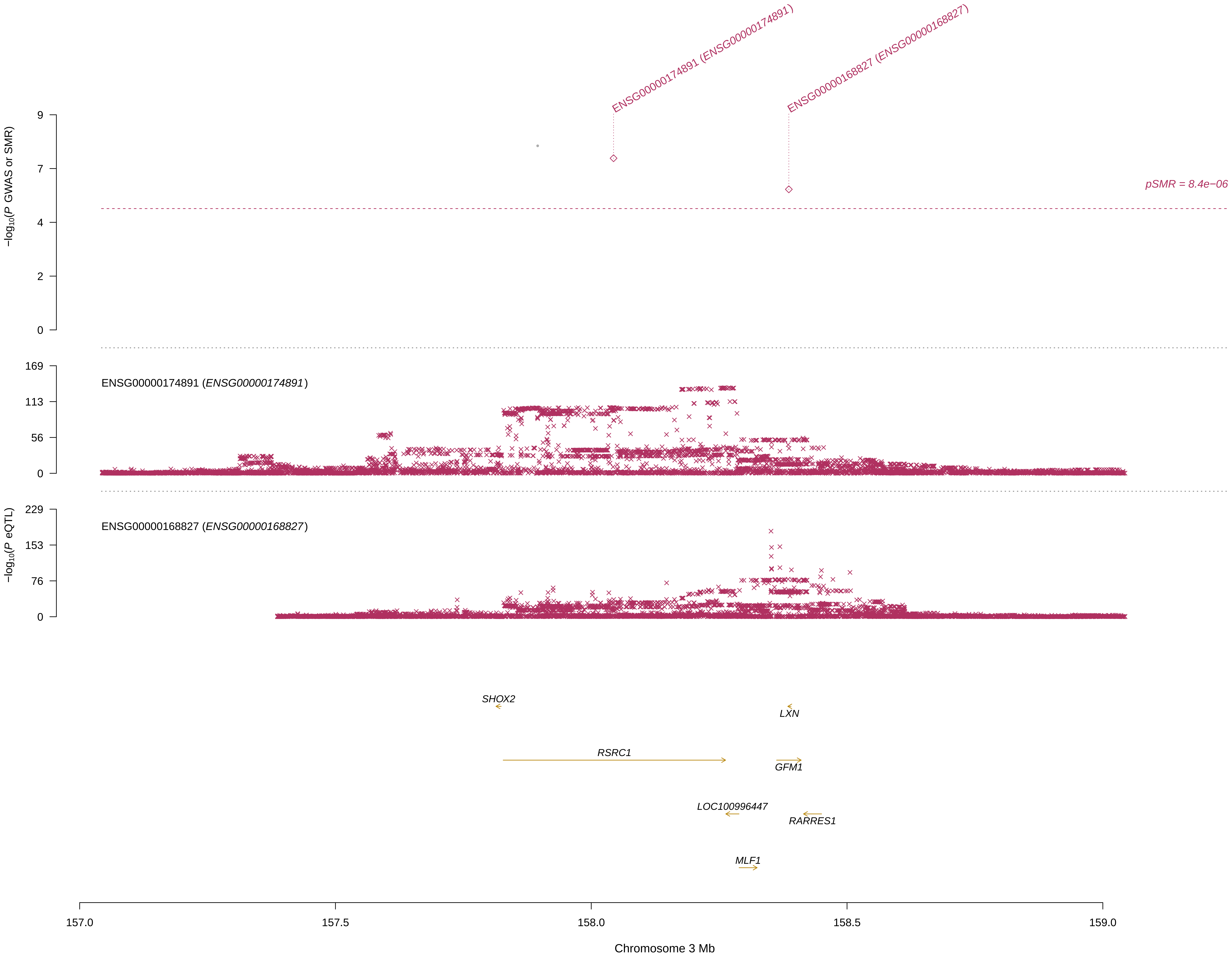

ENSG00000174891 (ENSG00000174891)
ENSG00000168827 (ENSG00000168827)
pSMR = 8.4e−06
−log10(P GWAS or SMR)
9
7
4
2
0
169
113
56
0
ENSG00000174891 (ENSG00000174891)
229
153
76
0
ENSG00000168827 (ENSG00000168827)
−log10(P eQTL)
SHOX2
LXN
RSRC1
GFM1
LOC100996447
RARRES1
MLF1
157.0
157.5
158.0
158.5
159.0
Chromosome 3 Mb
