# Supplementary material for: Multi-omics reveals cross-tissue regulatory mechanisms of autism risk loci via gut microbiota-immunity-brain axis
Source: AMB Express. 2025 Oct 29;15:161. doi: 10.1186/s13568-025-01969-4 (PMC12572420; doi:10.1186/s13568-025-01969-4)
Supplement: Supplementary file 2 — Supplementary Material 2 [file 13568_2025_1969_MOESM2_ESM.zip › Revised supplementary materials/7 Common SNP SMR results Plot/ENSG00000185658_locusplot.pdf]

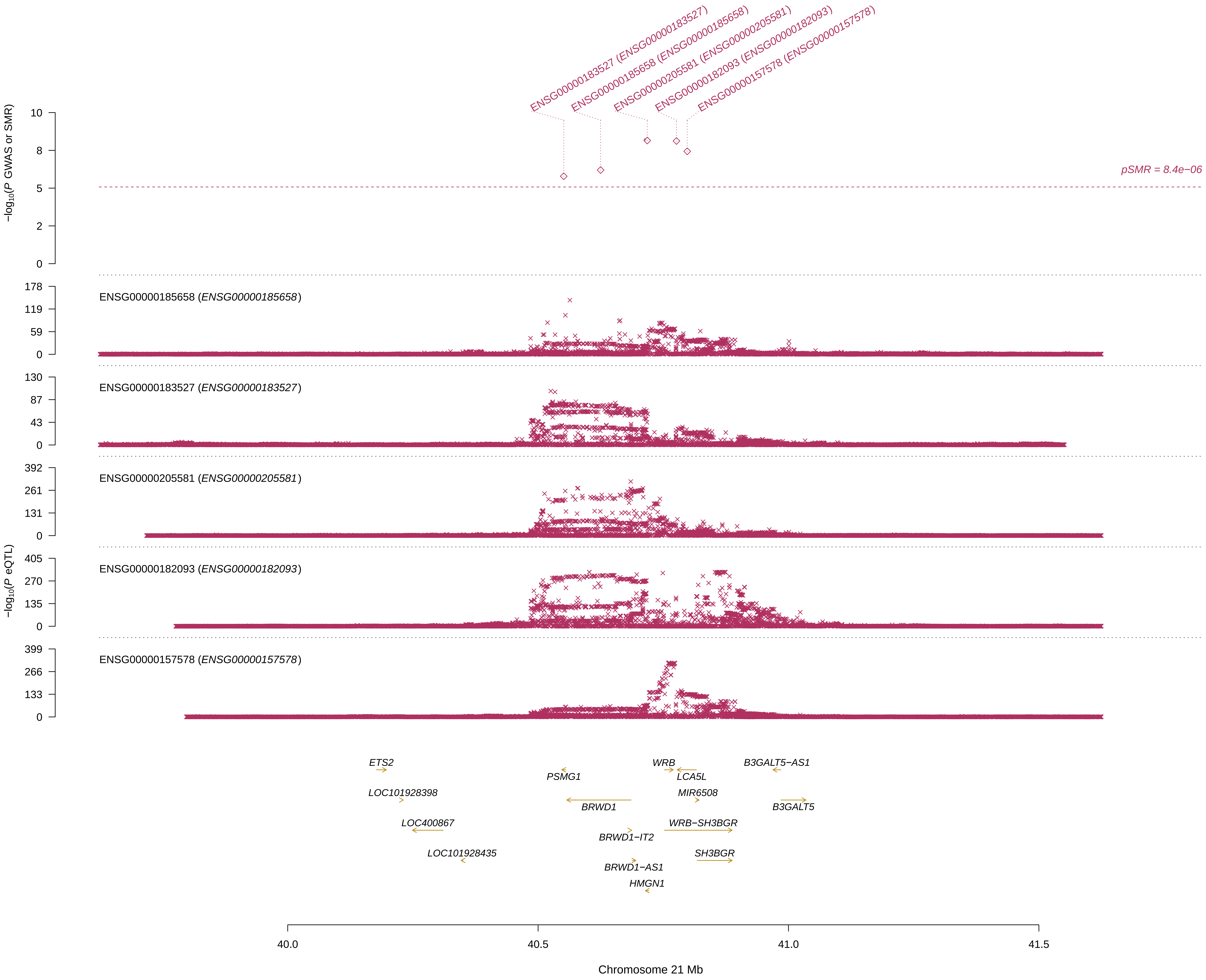

ENSG00000183527 (ENSG00000183527)
ENSG00000185658 (ENSG00000185658)
ENSG00000205581 (ENSG00000205581)
ENSG00000182093 (ENSG00000182093)
ENSG00000157578 (ENSG00000157578)
pSMR = 8.4e−06
−log10(P GWAS or SMR)
−log10(P eQTL)
ENSG00000185658 (ENSG00000185658)
ENSG00000183527 (ENSG00000183527)
ENSG00000205581 (ENSG00000205581)
ENSG00000182093 (ENSG00000182093)
ENSG00000157578 (ENSG00000157578)
ETS2
PSMG1
WRB
LCA5L
B3GALT5−AS1
LOC101928398
MIR6508
BRWD1
B3GALT5
LOC400867
WRB−SH3BGR
BRWD1−IT2
LOC101928435
SH3BGR
BRWD1−AS1
HMGN1
40.0
40.5
41.0
41.5
Chromosome 21 Mb
