# Supplementary material for: Multi-omics reveals cross-tissue regulatory mechanisms of autism risk loci via gut microbiota-immunity-brain axis
Source: AMB Express. 2025 Oct 29;15:161. doi: 10.1186/s13568-025-01969-4 (PMC12572420; doi:10.1186/s13568-025-01969-4)
Supplement: Supplementary file 2 — Supplementary Material 2 [file 13568_2025_1969_MOESM2_ESM.zip › Revised supplementary materials/7 Common SNP SMR results Plot/ENSG00000198518_locusplot.pdf]

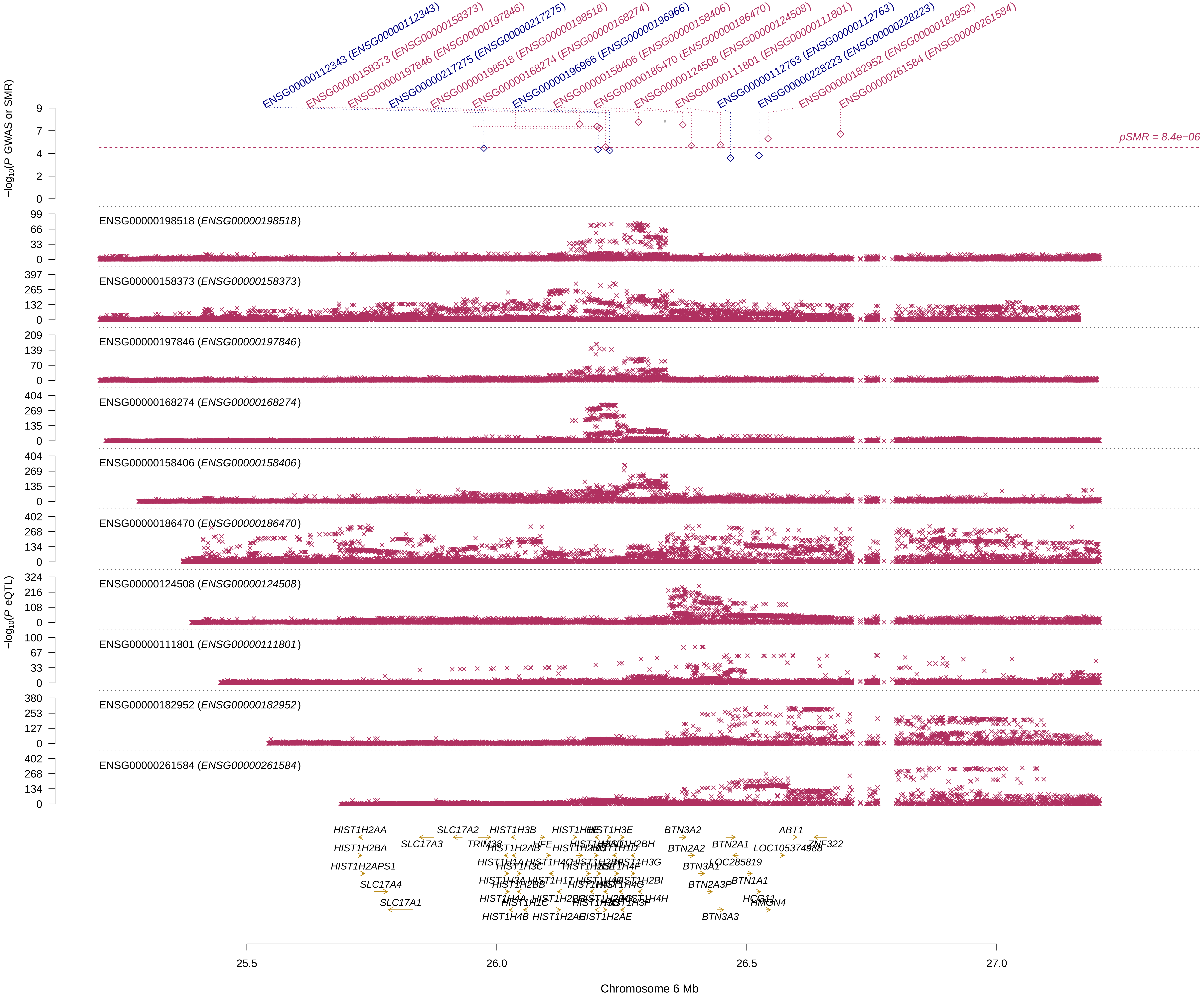

ENSG00000112343 (ENSG00000112343)
ENSG00000158373 (ENSG00000158373)
ENSG00000197846 (ENSG00000197846)
ENSG00000217275 (ENSG00000217275)
ENSG00000198518 (ENSG00000198518)
ENSG00000168274 (ENSG00000168274)
ENSG00000196966 (ENSG00000196966)
ENSG00000158406 (ENSG00000158406)
ENSG00000186470 (ENSG00000186470)
ENSG00000124508 (ENSG00000124508)
ENSG00000111801 (ENSG00000111801)
ENSG00000112763 (ENSG00000112763)
ENSG00000228223 (ENSG00000228223)
ENSG00000182952 (ENSG00000182952)
ENSG00000261584 (ENSG00000261584)
−log10(P GWAS or SMR)
pSMR = 8.4e−06
ENSG00000198518 (ENSG00000198518)
ENSG00000158373 (ENSG00000158373)
ENSG00000197846 (ENSG00000197846)
ENSG00000168274 (ENSG00000168274)
ENSG00000158406 (ENSG00000158406)
ENSG00000186470 (ENSG00000186470)
ENSG00000124508 (ENSG00000124508)
ENSG00000111801 (ENSG00000111801)
ENSG00000182952 (ENSG00000182952)
ENSG00000261584 (ENSG00000261584)
−log10(P eQTL)
HIST1H2AA
HIST1H2BA
HIST1H2APS1
SLC17A4
SLC17A1
SLC17A3
SLC17A2
TRIM38
HIST1H3B
HIST1H2AB
HFE
HIST1H3C
HIST1H1A
HIST1H4C
HIST1H3A
HIST1H1T
HIST1H2BB
HIST1H4A
HIST1H1C
HIST1H2BC
HIST1H4B
HIST1H2AC
HIST1H3E
HIST1H2BH
HIST1H1D
HIST1H3G
HIST1H4F
HIST1H2BI
HIST1H4G
HIST1H4H
HIST1H3F
HIST1H2AE
BTN3A2
BTN2A2
BTN3A1
BTN2A3P
BTN3A3
BTN2A1
LOC285819
BTN1A1
HCG11
HMGN4
ABT1
LOC105374988
ZNF322
25.5
26.0
26.5
27.0
Chromosome 6 Mb
