# Supplementary material for: Multi-omics reveals cross-tissue regulatory mechanisms of autism risk loci via gut microbiota-immunity-brain axis
Source: AMB Express. 2025 Oct 29;15:161. doi: 10.1186/s13568-025-01969-4 (PMC12572420; doi:10.1186/s13568-025-01969-4)
Supplement: Supplementary file 2 — Supplementary Material 2 [file 13568_2025_1969_MOESM2_ESM.zip › Revised supplementary materials/7 Common SNP SMR results Plot/ENSG00000228223_locusplot.pdf]

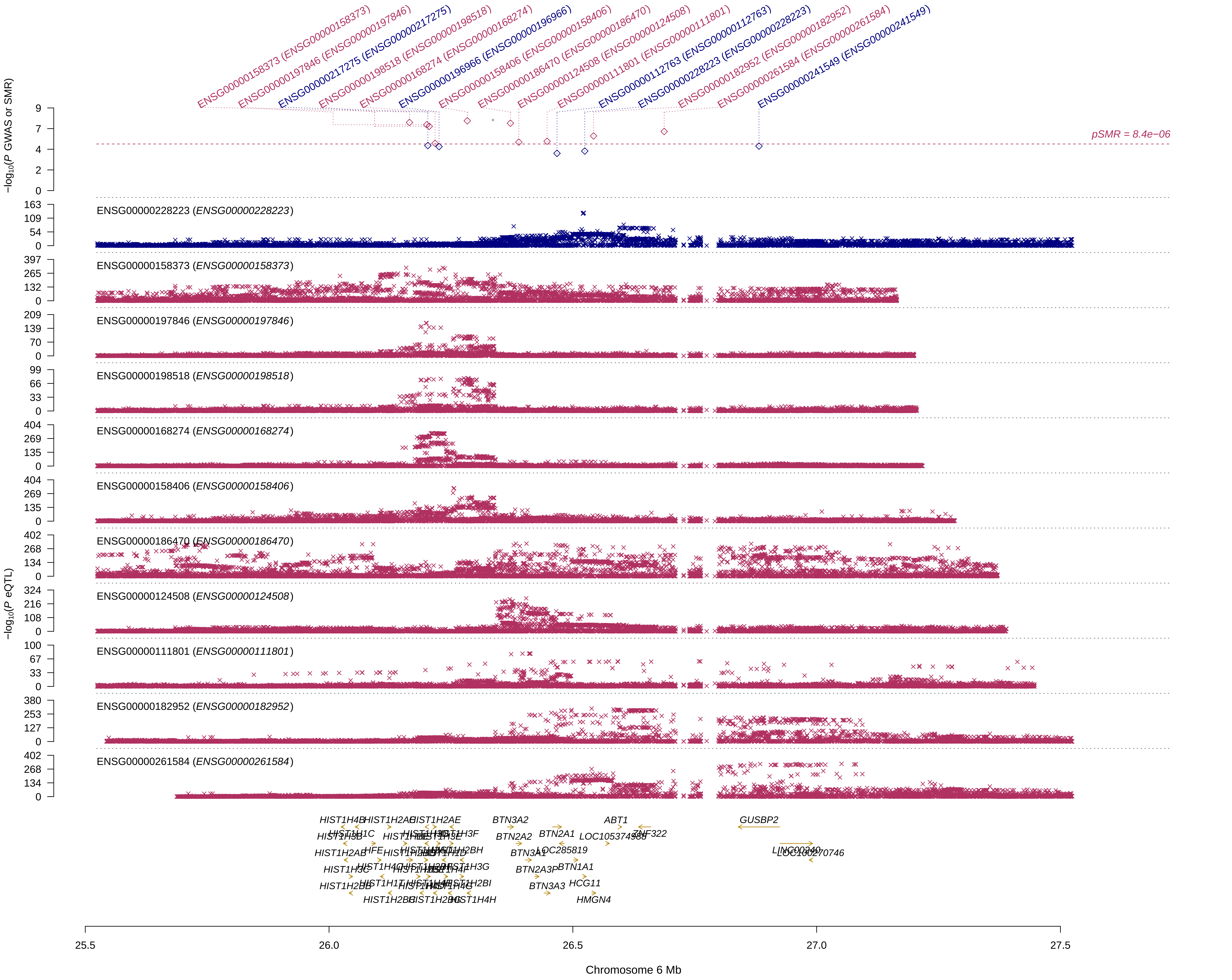

ENSG00000158373 (ENSG00000158373)
ENSG00000197846 (ENSG00000197846)
ENSG00000217275 (ENSG00000217275)
ENSG00000198518 (ENSG00000198518)
ENSG00000168274 (ENSG00000168274)
ENSG00000196966 (ENSG00000196966)
ENSG00000158406 (ENSG00000158406)
ENSG00000186470 (ENSG00000186470)
ENSG00000124508 (ENSG00000124508)
ENSG00000111801 (ENSG00000111801)
ENSG00000112763 (ENSG00000112763)
ENSG00000228223 (ENSG00000228223)
ENSG00000182952 (ENSG00000182952)
ENSG00000261584 (ENSG00000261584)
ENSG00000241549 (ENSG00000241549)
−log10(P GWAS or SMR)
pSMR = 8.4e−06
−log10(P eQTL)
ENSG00000228223 (ENSG00000228223)
ENSG00000158373 (ENSG00000158373)
ENSG00000197846 (ENSG00000197846)
ENSG00000198518 (ENSG00000198518)
ENSG00000168274 (ENSG00000168274)
ENSG00000158406 (ENSG00000158406)
ENSG00000186470 (ENSG00000186470)
ENSG00000124508 (ENSG00000124508)
ENSG00000111801 (ENSG00000111801)
ENSG00000182952 (ENSG00000182952)
ENSG00000261584 (ENSG00000261584)
HIST1H4B
HIST1H2AE
BTN3A2
ABT1
GUSBP2
HIST1H1C
HIST1H3F
BTN2A2
BTN2A1
ZNF322
HIST1H2AB
HFE
HIST1H2BH
LOC285819
BTN3A1
HIST1H3G
BTN1A1
BTN2A3P
HIST1H3C
HIST1H2BB
HIST1H2BI
BTN3A3
HCG11
HMGN4
HIST1H4H
LOC100270746
25.5
26.0
26.5
27.0
27.5
Chromosome 6 Mb
