# Supplementary material for: Multi-omics reveals cross-tissue regulatory mechanisms of autism risk loci via gut microbiota-immunity-brain axis
Source: AMB Express. 2025 Oct 29;15:161. doi: 10.1186/s13568-025-01969-4 (PMC12572420; doi:10.1186/s13568-025-01969-4)
Supplement: Supplementary file 2 — Supplementary Material 2 [file 13568_2025_1969_MOESM2_ESM.zip › Revised supplementary materials/7 Common SNP SMR results Plot/ENSG00000261584_locusplot.pdf]

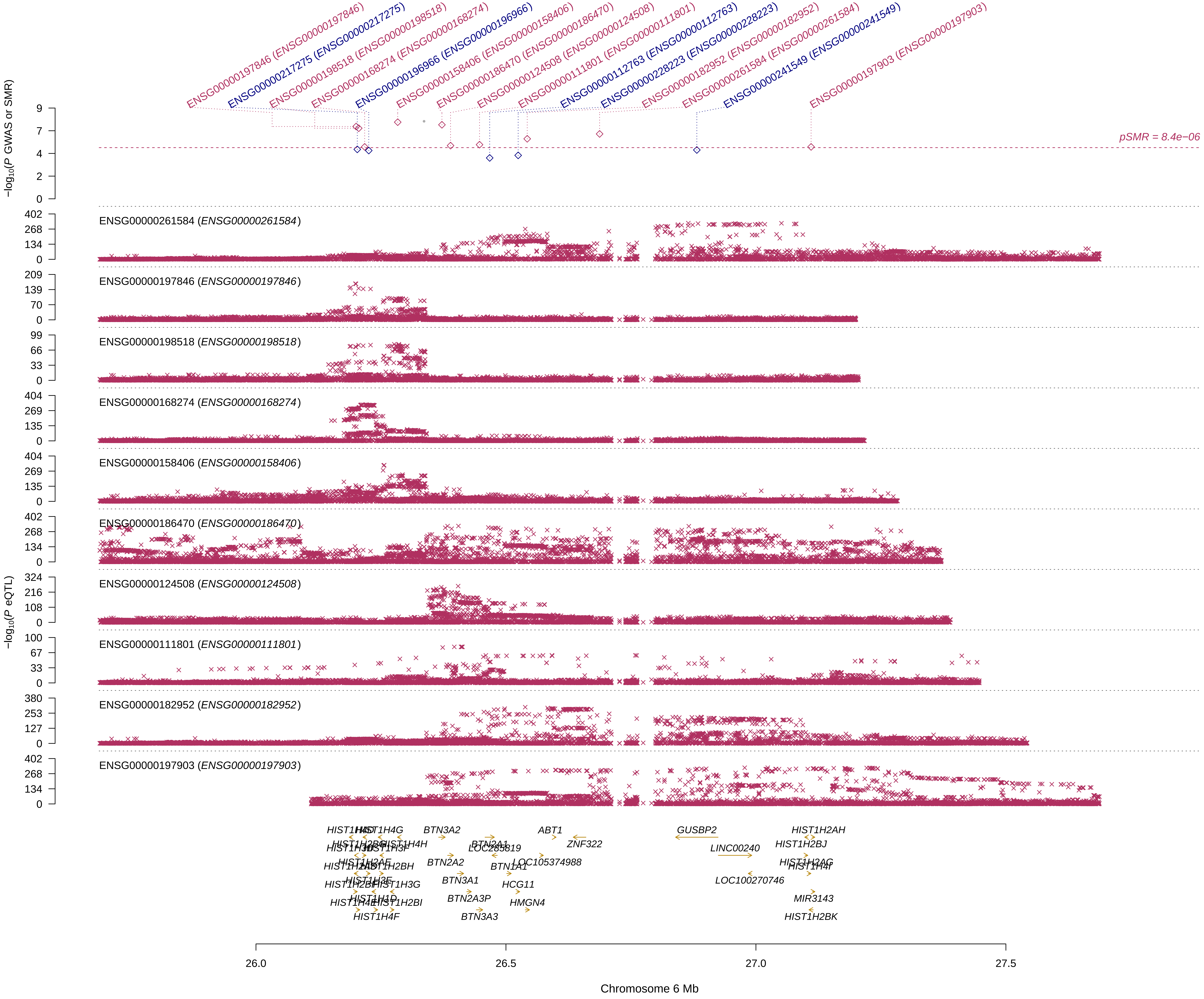

ENSG00000197846 (ENSG00000197846)
ENSG00000217275 (ENSG00000217275)
ENSG00000198518 (ENSG00000198518)
ENSG00000168274 (ENSG00000168274)
ENSG00000196966 (ENSG00000196966)
ENSG00000158406 (ENSG00000158406)
ENSG00000186470 (ENSG00000186470)
ENSG00000124508 (ENSG00000124508)
ENSG00000111801 (ENSG00000111801)
ENSG00000112763 (ENSG00000112763)
ENSG00000228223 (ENSG00000228223)
ENSG00000182952 (ENSG00000182952)
ENSG00000261584 (ENSG00000261584)
ENSG00000241549 (ENSG00000241549)
ENSG00000197903 (ENSG00000197903)
−log10(P GWAS or SMR)
pSMR = 8.4e−06
−log10(P eQTL)
ENSG00000261584 (ENSG00000261584)
ENSG00000197846 (ENSG00000197846)
ENSG00000198518 (ENSG00000198518)
ENSG00000168274 (ENSG00000168274)
ENSG00000158406 (ENSG00000158406)
ENSG00000186470 (ENSG00000186470)
ENSG00000124508 (ENSG00000124508)
ENSG00000111801 (ENSG00000111801)
ENSG00000182952 (ENSG00000182952)
ENSG00000197903 (ENSG00000197903)
HIST1H4G
BTN3A2
ABT1
ZNF322
GUSBP2
HIST1H2AH
HIST1H4H
BTN2A1
LOC285819
LINC00240
HIST1H2BJ
HIST1H3F
HIST1H2BH
BTN2A2
LOC105374988
BTN1A1
HIST1H2AG
HIST1H4I
BTN3A1
HCG11
LOC100270746
HIST1H3G
BTN2A3P
HMGN4
MIR3143
HIST1H2BI
HIST1H4F
BTN3A3
HIST1H2BK
Chromosome 6 Mb
