# Supplementary material for: Multi-omics reveals cross-tissue regulatory mechanisms of autism risk loci via gut microbiota-immunity-brain axis
Source: AMB Express. 2025 Oct 29;15:161. doi: 10.1186/s13568-025-01969-4 (PMC12572420; doi:10.1186/s13568-025-01969-4)
Supplement: Supplementary file 2 — Supplementary Material 2 [file 13568_2025_1969_MOESM2_ESM.zip › Revised supplementary materials/7 Common SNP SMR results Plot/legends.docx]

SMR locus plot: x-axis: The term "chromosomal physical location" is used to denote the genomic interval in which the target gene is located; The left vertical axis (Y_1_ axis) is defined as follows: log_10_(P GWAS or SMR) is a metric employed to ascertain the significance level of GWAS or SMR analysis, a greater value indicates a smaller P-value and stronger significance; The right vertical axis (Y_2_ axis) is defined as follows: The -log_10_(P eQTL) metric is employed to denote the statistical significance of eQTL analysis, which is utilised to evaluate the strength of the correlation between gene expression and SNPs; The distribution of other gene names along the horizontal axis is indicative of the distribution characteristics of gene clusters, aiding in the identification of functionally significant regions of genetic association.
